# Proceedings of the 31st European Paediatric Rheumatology Congress: part 1

**DOI:** 10.1186/s12969-024-01004-z

**Published:** 2024-09-09

## PReS 2024 Abstract Submission

### Autoinflammatory diseases

#### OR01 Assessment of ADA2 activity levels: report from the Italian study group on DADA2

##### Alessia Cafaro^1^, Roberta Caorsi^2^, Enrico Drago^3^, Alice Grossi^4^, Sebastiano Barco^1^, Chiara Conti^3^, Flippo Maestrini^5^, Maurizio Miano^6^, Francesca Fioredda^6^, Maria Carla Giarrattana^6^, Federica Brazaghi^7^, Michela Lupia^6^, Stefano Volpi^2, 3^, Riccardo Papa^2^, Francesca Schena^2^, Alessandra Mortellaro^7^, Isabella Ceccherini^4^, Alessandro Aiuti^7^, Carlo Dufour^6^, Giuliana Cangemi^1^, Marco Gattorno^2^ and Italian study group on DADA2

###### ^1^Central Laboratory of Analysis; ^2^Rheumatology and Autoinflammatory Diseases, IRCCS Istituto Giannina Gaslini; ^3^DINOGMI, University of Genova; ^4^Genetics of Rare Diseases, IRCCS Istituto Giannina Gaslini; ^5^University of Genova; ^6^Hematology, IRCCS Istituto Giannina Gaslini, Genova; ^7^IRCCS Ospedale San Raffaele, Milano, Italy

####### **Correspondence:** Roberta Caorsi

*Pediatric Rheumatology 2024*, **22(2):**PReS24-ABS-1499

**Introduction:** Adenosine Deaminase 2 deficiency (DADA2) is a rare monogenic autoinflammatory disease resulting from loss-of-function mutations in ADA2. Functional assays are crucial for early diagnosis. In 2021, we introduced a liquid chromatography-tandem mass spectrometry (LC-MS/MS) method to assess ADA2 activity from dried plasma spot (DPS) [1].

**Objectives:** To define cut-offs of ADA2 activity in the normal population and to assess the test’s utility in a large multicentre real-life cohort of patients with suspected DADA2.

**Methods:** At first, ADA2 activity was tested in 17 patients with genetically confirmed DADA2, 23 clinically healthy and genetically confirmed carriers, and 132 healthy donors. The test was then performed in 19 new diagnoses of DADA2 later confirmed genetically, 4 carriers subsequently confirmed genetically, 195 patients with other related conditions. Receiver Operating Curves (ROC) analysis evaluated the diagnostic performance of ADA2 activity in DPS. Spearman correlation coefficients were employed to investigate the relations between ADA2 activity in DPS and age. Significance was determined at a threshold of P<0.05 for all analyses, with two-tailed tests utilized.

**Results:** ADA2 activity in DPS effectively discriminated patients with DADA2 from carriers (AUC=0.946, P<0.001) and carriers from patients with suspected but non-DADA2 conditions (AUC=0.890, P<0.001) with high sensitivity and specificity. A significant inverse correlation existed between DADA2 activity in DPS and age (P<0.0001). The ADA2 activity cut-off values in DPS were identified as follows: ≤ 0.09 mU/mL for patients with DADA2, 0.10 – 0.39 mU/mL for carriers, and ≥ 0.40 mU/mL for subjects with normal levels. All DADA2 patients with two pathogenic variants in the ADA2 gene exhibited ADA2 activity ≤ 0.09 mU/mL. For three patients, with a mild phenotype, exhibiting a pathogenic variant along with a Variant of Uncertain Significance (VUS) in the ADA2 gene, carrier-like ADA2 activity [2] was observed. An overlap in ADA2 activity levels was observed between carriers and healthy subjects.

**Conclusion:** The LC-MS/MS ADA2 activity test due to its high sensitivity and specificity is able to discriminate DADA2 patients from carriers and healthy subjects and to identify carriers of DADA2 variants with different pathogenic impact.

**Patient Consent**

Yes, I received consent

The study was supported by a Grant of the Italian Ministry of Health (Finalizzata 2019).

**Disclosure of Interest**

None Declared

**References**

1. Cafaro A, A Novel LC – MS / MS-Based Method for the Diagnosis of ADA2 Deficiency from Dried Plasma Spot Molecules. 2021 21;26(18):5707.
2. Jee H, Comprehensive analysis of ADA2 genetic variants and estimation of carrier frequency driven by a function-based approach. J Allergy Clin Immunol. 2022 149(1):379-387

### Autoinflammatory diseases

#### OR02 Investigation of CD4+ T and innate lymphoid cell subtypes in patients with mevalonate kinase deficiency

##### Busra Seniz Demir^1,2^, Mohammad Ahmad Houran^1,2^, Sümeyra Ozdemir Çiçek^3^, Fatih Kardaş^4^, Hatice Güneş^4^, Rabia M. Kışla Ekinci^5^, Betül Sözeri^6^, Ayşenur Paç Kısaarslan^3^, Ahmet Eken^2,7^

###### ^1^Department of Medical Biology, Erciyes University Intitute of Health Sciences; ^2^Erciyes University Genome And Stem Cell Centre; ^3^Department of Pediatric Rheumatology; ^4^Department of Pediatric Metabolism, Erciyes University Faculty of Medicine, Kayseri; ^5^Department of Pediatric Rheumatology, Cukurova University Medical Faculty, Adana; ^6^Department of Pediatric Rheumatology, University of Health Sciences Umraniye Training and Reasarch Hospital, Istanbul; ^7^Department of Medical Biology, Erciyes University Faculty of Medicine, Kayseri, Türkiye

####### **Correspondence:** Busra Seniz Demir

*Pediatric Rheumatology 2024*, **22(2):**PReS24-ABS-1671

**Introduction:** Mevalonate kinase deficiency (MKD) is an autoinflammatory disease with an autosomal recessive inheritance caused by a mutation in the mevalonate kinase enzyme gene. Mevalonate Kinase enzyme contributes to the conversion of Mevalonate-5-Phosphate, an intermediate metabolite, which is essential for synthesis of main proteins involved in cholesterol biosynthesis, ubiquinones production, and protein prenylation. Existing research supports the idea that mevalonate metabolism, in particular, negatively influences the frequency of regulatory T cells (Treg) and T-helper 2 cells (Th2). Furthermore, mevalonate metabolism also has an impact on cell size and proliferation. Changes caused by MVK deficiency in macrophages/ antigen-presenting cells have been questioned in MKD patients until now. Despite the significant role of T cells in metabolism, the changes caused by *MVK* deficiency in T cells in MKD patients and their contribution to attacks and immunological disorders have not yet been studied.

**Objectives:** Our study aims to investigate the changes in CD4+ T and Innate Lymphoid Cells (ILCs) which are counterparts of T lymphocytes, immunobiology resulting from mutations that lead to *MVK* deficiency in MKD patients. We hypothesized that individuals with MVK deficiency may induce certain changes, particularly in the development of Th17 and Treg cells.

**Methods:** We categorised the patients as HIDS MKD patients in attack and HIDS patients in remission, paediatric patients using statins that simultaneously inhibit mevalonate metabolism and cholesterol biosynthesis, and healthy controls. We collected peripheral blood samples and isolated peripheric mononuclear blood cells (PBMC) from these patients and healthy volunteers. We collected serum samples from blood and quantified them using a LEGENDplex™ HU Th Cytokine Panel (12-plex) w/ VbP V02 panel. 1x10^6^ PBMC were investigated for CD4+ T cells phenotypes; Th1, Th2, Th17 and Treg cells. We stained the cell surfaces directly with ILC-specific antibodies.

**Results:** There was no change in total ILCs, Th1 and Th17 cells, a significant decrease in Th2 cells and a significant increase in Treg cells in MVK patients. TCR-mediated T cell proliferation and activation were impaired in MVK patients.

**Conclusion:** In patients with MVK deficiency, alterations in T cell phenotypes and T cell responses were observed, in particular a reduced type 2 immune response. These results support that MVK deficiency leads to defects in CD4+ T cell subtypes and T cell activation and proliferation in general, and shed light on the broad spectrum of immunodeficiency defects in the adaptive immune system of HIDS patients.

**Date of birth::** 06.08.1997

**Patient Consent**

Yes, I received consent

**Disclosure of Interest**

None Declared

**References**

1. Thurnher M, Gruenbacher G. T Lymphocyte Regulation by Mevalonate Metabolism.Sci Signal, 2015:8(370);4-14.
2. Yagi Y, Kuwahara M, Suzuki J, Imai Y, Yamashita M. Glycolysis and Subsequent Mevalonate Biosynthesis Play an Important Role in Th2 Cell Differentiation. Biochemical and Biophysical Research Communications, 2020:530(2);355-361.
3. Ntolkeras G, Barba C, Mavropoulos A, et al. On the immunoregulatory role of statins in multiple sclerosis: the effects on Th17 cells. Immunol Res., 2019;67(45):310-324.
4. Manthiram K, Edwards KM, Long SS. “Prolonged, Recurrent, and Periodic Fever Syndromes.” In Principles and Practice of Pediatric Infectious Diseases, 2018:5;117-128.

### Juvenile dermatomyositis

#### OR03 A mitochondrial gene signature to stratify juvenile dermatomyositis patient groups for more targeted treatments

##### Meredyth G. L. Wilkinson^1,2,3^, Aris E. Syntakas^1,2^, Restuadi Restuadi^1,2,3^, Huong D. Nguyen^1,2^, Melissa M. Kartawinata^1,2^, Dario Cancemi^1,2^, Vicky Alexiou^1,2^, Lucy R. Marshall^1,2^, Bethany R. Jebson^1,2^, Elizabeth Ralph^1,2^, Hannah Peckham^2^, Anna Radziszewska^2^, Wei-Yu Lin^4^, Georg W. Otto^3,5^, George Hall^3,5^, Daniel Kelberman^3,5^, Sergi Castellano^3,5^, Coziana Cuirtin^2^, Simon Eaton^3,6^, Claire T. Deakin^1,2,3,7^, Elizabeth C. Rosser^2,8^, Chris Wallace^4,9^, Lucy R. Wedderburn^1,2,3^

###### ^1^Infection, Immunity and Inflammation Research and Teaching Department, UCL Great Ormond Street Institute of Child Health; ^2^Centre for Adolescent Rheumatology Versus Arthritis at UCL UCLH and GOSH, University College London; ^3^NIHR Biomedical Research Centre at GOSH, London; ^4^MRC Biostatistics Unit, University of Cambridge, Cambridge; ^5^Genetics and Genomic Medicine Research & Teaching Department; ^6^Developmental Biology and Cancer Research & Teaching Department, UCL Great Ormond Street Institute of Child Health, London, United Kingdom; ^7^School of Population Health, University of New South Wales, Sydney, Australia; ^8^Centre for Rheumatology research, University College London, London; ^9^Cambridge Institute of Therapeutic Immunology and Infectious Disease (CITIID), University of Cambridge, Cambridge, United Kingdom

####### **Correspondence:** Meredyth G. L. Wilkinson

*Pediatric Rheumatology 2024*, **22(2):**PReS24-ABS-1639

**Introduction:** Juvenile dermatomyositis (JDM) is a rare childhood autoimmune myositis, typically presents with proximal muscle weakness and skin manifestations. JDM is characterised by abnormal interferon (IFN) type I signalling and mitochondrial abnormalities contributing to the disease pathogenesis. There is a need for better treatments, with novel therapeutics targeting IFN and mitochondria pathways, being the clear candidates.

**Objectives:** This study aimed to define and validate a JDM mitochondrial gene signature and investigate how this signature correlates with disease activity. Establish the signature as a tool to improve the understanding of what drives JDM inflammation, support individualised selection of treatments and new target discovery.

**Methods:** Peripheral blood mononuclear cell (PBMC) samples were obtained from treatment-naïve, early-treatment and on-treatment JDM and age/sex-matched child healthy controls (controls). RNA-sequencing (RNAseq) was performed from total PBMC and sorted CD14^+^ monocytes. The dataset comprised JDM pre-treatment (*n*=33), early-treatment (*n*=5), on-treatment (*n*=10) and controls (*n*=19). Differentially expressed genes (DEG) between conditions were analysed using EdgeR. Factor analysis was used to model interrelationships across genes to identify common and unique genes.

**Results:** Validation of our previously published RNAseq results in a new, larger cohort of JDM patients identified an overlapping gene signature which encapsulated 37 genes from the mitochondrial gene ontology term. By using unsupervised, hierarchical clustering, a clear separation was observed in the normalised gene counts for the defined 37 mitochondrial gene set (MGS) between the four different groups, JDM treatment naïve (*n*=26), early-treatment (*n*=4, <2 months on treatment), on-treatment (*n*=8, average time on treatment = 14 months (range=4.3-32 months)) and controls (*n*=19). Factor analysis was performed on the 37-gene MGS to model interrelationships among individual genes. While certain groups of genes were found to have shared variance, we identified a set of 18 genes with unique contributions to the overall MGS. Calculating a factor score for each sample, we showed that mitochondrial dysfunction is significantly abnormal in JDM treatment naïve patients and it is still abnormal at early/later on-treatment timepoints even, relative to controls. This demonstrated that current treatment does not resolve this pathological mitochondrial signature even in those patients that have improved disease. This finding was observed in the PBMC RNAseq data as well. We found significant positive correlation between the MGS factor score derived from JDM treatment naïve monocytes with the Manual Muscle Test (MMT8) score (*p*=0.0007, R^2^=0.4841). These data suggest that the signature could have strong clinical utility for biomarker development in blood.

**Conclusion:** This study identified and validated a dysregulated mitochondrial signature in treatment naïve JDM CD14+ monocytes, further validated in PBMCs, which positively correlated with muscle weakness by MMT8 score tool. This signature could have clinical implications as a biomarker of mitochondrial health in JDM, potentially useful for patient treatment optimisation.

**Trial registration identifying number:** Not Applicable

**Patient Consent**

Yes, I received consent

**Disclosure of Interest**

None Declare

### Macrophage activation syndrome

#### OR04 Evolution of lung inflammation in a mouse model of chronic/recurrent macrophage activation syndrome

##### Richard Chhaing^1^, Natsumi Inoue^1^, Jana Latayan^1^, Grant S. Schulert^1,2^

###### ^1^Cincinnati Children’s Hospital Medical Center; ^2^Pediatrics, University of Cincinnati College of Medicine, Cincinnati, United States

####### **Correspondence:** Grant S. Schulert

*Pediatric Rheumatology 2024*, **22(2):**PReS24-ABS-1692

**Introduction:** Systemic juvenile idiopathic arthritis-associated lung disease (SJIA-LD) is a life-threatening complication and associated in >80% of cases with macrophage activation syndrome (MAS); however, the underlying disease mechanisms driving lung inflammation are unknown. We have previously shown that the TLR9-stimulated mouse model of MAS demonstrates IFNγ-driven lung inflammation replicating several key features of SJIA-LD.

**Objectives:** To determine the evolution of lung inflammation in a TLR9-stimulated mouse model of chronic/recurrent MAS.

**Methods:** C57BL/6J mice were sequentially intraperitoneal (IP) injected 5 times with ODN1826 (CpG) over 10 days, with 10 days following cycles, yielding mice with acute MAS (1 cycle of CpG), recovery (10 days after one cycle), or chronic/recurrent MAS (3 cycles of CpG). Blood, Bronchoalveolar lavage (BAL), lung and spleen were collected. Somalogic proteomic data from BAL samples was analyzed with a log2 fold change >1 and P ≤ 0.05. Lungs were digested, stained and analyzed for flow cytometry using Aurora.

**Results:** Compared to mice with acute MAS, mice with three sequential episodes of MAS (chronic/recurrent MAS) demonstrated largely similar findings of anemia, thrombocytopenia, lymphopenia, splenomegaly, and serum and BAL cytokine elevations. In order to quantify changes in lung monocyte and macrophage populations, we examined whole lung digests using Flow cytometry. During acute MAS we found significantly higher proportions of neutrophils, interstitial macrophages, and less mature monocytes and macrophages in the lungs. During MAS recovery however, most cell populations normalized except for a persistent increase in Ly6C- monocytes and macrophages. With chronic/recurrent MAS, we observed more marked alterations in the myeloid compartment, including significant decrease in Ly6C-/CD11bLo/SiglecF+ alveolar macrophages and corresponding increase in Ly6C+/CD11b+/SiglecF- macrophages, as well as increases in both CD11b+ conventional dendritic cells (DCs) and CD103+ pulmonary DCs. In order to determine how the alveolar inflammatory environment changed during chronic/recurrent MAS, we performed proteomics from BAL fluid using Somalogic. Compared to control (PBS-treated mice), in mice with chronic/recurrent MAS we identified 119 proteins with increased abundance and 91 proteins with decreased abundance. These included significantly increased BAL levels of IL-1A, IL-16, IL-18BP, IL-33, and TGFA. Interestingly, most proteins with increased abundance (72%) were not significantly different during acute MAS. Using GO-Elite, we found Complement and Coagulation Cascade to be the most highly enriched KEGG pathway among proteins with increased abundance in chronic/recurrent MAS (z-score 9.37, adjusted *p*=9.14x10-5), including C3, CFI, F10, FGG, SERPINC1, SERPIND1, and VWF.

**Conclusion:** In a mouse model of chronic/recurrent MAS, we see persistent alternations in lung myeloid populations including replacement of Ly6C-/CD11bLo/SiglecF+ alveolar macrophages with Ly6C+/CD11b+/SiglecF- macrophages, and increases in DC populations. We also see increasing levels of both pro-inflammatory and pro-fibrotic cytokines as well as activation of the complement and coagulation pathways in alveolar fluid. Together, this work supports persistent alternations in the lung inflammatory environment driven by chronic/recurrent MAS, with important implications for the development of SJIA-LD.

**Patient Consent**

Not applicable (there are no patient data)

**Disclosure of Interest**

R. Chhaing: None Declared, N. Inoue: None Declared, J. Latayan: None Declared, G. Schulert Grant / Research Support with: IpiNovyx, Consultant with: SOBI, Boehringer Ingelheim

### Vasculitides

#### OR05 Pathogenic role of inflammasome activation and interleukin-1β overproduction in Kawasaki disease

##### Shuya Kaneko^1^, Asami Shimbo^1^, Maho Hatano^1^, Futaba Miyaoka^1^, Yuko Hayashi^1^, Hitoshi Irabu^1^, Yuko Akutsu^1^, Masaaki Mori^2^, Masaki Shimizu^1^

###### ^1^Department of Pediatrics and Developmental Biology; ^2^Department of Lifetime Clinical Immunology, Tokyo Medical and Dental University, Tokyo, Japan

####### **Correspondence:** Shuya Kaneko

*Pediatric Rheumatology 2024*, **22(2):**PReS24-ABS-1621

**Introduction:** Kawasaki disease (KD) is an acute self-limited systemic vasculitis with a predilection for coronary arteries that occurs in young children. Although activation of innate immune cells and excessive production of inflammatory cytokines are thought to be relevant to the pathogenesis of KD, the pathological mechanisms of vasculitis in KD are obscure.

**Objectives:** To investigate the activation profile of inflammatory molecules in innate immune cells and vascular endothelial cells from the view of inflammation-immune cells-vascular crosstalk.

**Methods:** Thirty-eight patients with KD were enrolled in this study. Transcription levels of whole blood RNA were comprehensively analyzed using the RT-qPCR array kit regarding inflammasome-associated molecules. Serum cytokine levels were quantified with the Luminex assay. In addition, we developed a new in vitro cell stimulation assay that serves as a vasculitis model by co-culturing monocytes differentiated from iPS cells derived from healthy individuals (iPSC-Monocytes), human coronary artery endothelial cells (HCAEC), and serum from KD patients or healthy controls (HCs). The changes in RNA signatures in iPSC-Monocytes and HCAEC were analyzed with RT-qPCR.

**Results:** Transcription levels of 10 inflammasome-associated molecules including *IL1B, NLRC4, AIM2, CASP1, CASP5, CARD6, MYD88, NLRP12, NAIP,* and *TNFSF14* in whole blood RNA were increased in the acute phase of KD patients compared to those of HCs. The transcription levels of these gene expressions decreased in the convalescent phase. No differences in gene expressions were revealed between the good-responsive group and the poor-responsive group to initial treatments for KD. Serum IL-1β levels were increased in patients with KD, especially in patients complicated with coronary artery lesions. With the in vitro vasculitis model, transcriptions of *IL1B* and *TNF* in monocytes and *IL1B*, *VEGF,* and *ANGPT2*, which reflect vasculitis/angiogenesis, in HACEC were increased in the KD groups rather than those in the HC groups. On the other hand, the transcription level of *ANGPT1* was decreased in the KD groups.

**Conclusion:** Inflammasome activation and excessive production of IL-1β might play an important role in the pathogenesis of vasculitis in KD.

**Patient Consent**

Yes, I received consent

**Disclosure of Interest**

None Declared

### New diseases

#### OR06 Haploinsufficiency of PTPN2 and early systemic autoimmunity: from Evans syndrome to systemic lupus

##### Marie Jean-Pierre^1^, Jade Cognard^2^, Maud Tusseau^2^, Quentin Riller^1^, Linh-Chi Bui^3^, Jeremy Berthelet^3^, Audrey Laurent^4^, Etienne Crickx^5^, Marianna Parlato^6^, Marie-Claude Stolzenberg^6^, Felipe Suarez^5^, Guy Leverger^5^, Nathalie Aladjidi^7^, Sophie Collardeau-Frachon^4^, Christine Pietrement^8^, Fernando Rodrigues-Lima^3^, Marion Malphettes^5^, Thierry Walzer^2^, Frédéric Rieux-Laucat^9^, Alexandre Belot^2, 4^, Anne-Laure Mathieu^2^

###### ^1^Imagine Institute, Paris; ^2^CIRI, Lyon; ^3^CNRS, Paris; ^4^CHU Lyon, Lyon; ^5^APHP, ^6^INSERM, Paris; ^7^CHU Bordeaux, Bordeaux; ^8^CHU Reims, Reims; ^9^INSERM, Imagine Institute, Paris, France

####### **Correspondence:** Jade Cognard

*Pediatric Rheumatology 2024*, **22(2):**PReS24-ABS-1186

**Introduction:** The immune system is tasked with deploying effective defenses against infections while simultaneously maintaining tolerance towards self-components. This delicate balance is maintained by a set of mechanisms that negatively regulate immune cell activation pathways. Among these, the phosphatase PTPN2, which modulates the JAK/STAT pathway pivotal in the signaling of various cytokines, has been implicated in the onset of autoimmunity (1–3).

**Objectives:** We sought to understand the impact of *PTPN2* variants on the clinical phenotype and immune responses, thereby gaining a better understanding of the role of this key phosphatase in pathophysiology.

**Methods:** Whole exome analysis of patients with pediatric-onset systemic lupus erythematosus or Evans syndrome has led to the identification of six new mutations in *PTPN2*. *In vitro* analyses including cytokine reporter, phosphatase assays and flow cytometry have enabled to characterize how these mutations affect cellular functions and signaling pathways.

**Results:** All identified heterozygous mutations resulted in the loss of regulatory function of PTPN2. This occurred either through loss of expression or changes in its phosphatase activity, leading to hyperactivation of the JAK/STAT pathway and hyperproliferation of patient T cells upon cytokine’s stimulation. Furthermore, patients exhibited high serum levels of various inflammatory cytokines, mimicking the profile observed in individuals with gain-of-function mutations in various STAT factors. Flow cytometry analysis of patient cells revealed typical alterations associated with autoimmunity, such as expansion of CD11c+ B lymphocytes, also known as “age-associated B cells”, follicular helper T lymphocytes and classical monocytes. The clinical phenotype observed varied depending on the mutation’s localization, with incomplete penetrance among relatives, but all patients had positive antinuclear autoantibodies, antiplatelet antibodies, and a positive Coombs test.

**Conclusion:** We report six new monoallelic variants of *PTPN2* in six unrelated families. They were variably associated with the development of systemic lupus in one family and Evans syndrome in five families, thus extending the spectrum of clinical manifestations associated with *PTPN2* deficiency. All six variants lead to functional defects in PTPN2, either through loss of expression or alterations in its activity resulting in hyperactivation of JAK/STAT pathways. These findings support the notion that loss of function of negative regulators of cytokine pathways can lead to a wide range of autoimmune manifestations and that *PTPN2* is playing a pivotal role as a regulator of the immune system. Haploinsufficiency of PTPN2 may constitute a new subset of autoimmune diseases, with clinical expression potentially influenced by other modifying or epigenetic factors, many of which are yet to be discovered. Identifying and understanding the mechanisms of action of these variants allows for the proposition of targeted therapies to affected individuals.

**Patient Consent**

Yes, I received consent

**Disclosure of Interest**

None Declared

**References**

1. Thaventhiran et al. PMID: 32499645, Nature. 2 juill 2020;583(7814):90‑5
2. Parlato M et al. PMID: 32721438, Gastroenterology. nov 2020;159(5):1968-1971.e4
3. Awwad J et al. PMID : 37537852, J Genet. 15 juill 2023;102(2):37

### Systemic lupus erythematosus and antiphospholipid syndrome

#### OR07 Deciphering the intricacies of Childhood-Onset SLE (CSLE) through high-dimensional data analysis: augmenting theragnosis with an immunological-based diagnostic model that unveils mechanistic insights

##### Katherine Nay Yaung^1,2^, Joo Guan Yeo^1,2,3^, Martin Wasser^1^, Su Li Poh^1^, Kai Liang Teh^3^, Lena Das^3^, Yun Xin Book^3^, Nicholas Kim Huat Khoo^1^, Zoe Mei Yun Tay^1^, Rachel Cheong^1,3^, Judith Ju Ming Wong^1,3^, Thaschawee Arkachaisri^2,3^, Salvatore Albani^1,2,3^

###### ^1^Translational Immunology Institute, SingHealth Duke-NUS Academic Medical Centre; ^2^Duke-NUS Medical School; ^3^KK Women’s and Children’s Hospital, Singapore, Singapore

####### **Correspondence:** Katherine Nay Yaung

*Pediatric Rheumatology 2024*, **22(2):**PReS24-ABS-1398

**Introduction:** Childhood-onset systemic lupus erythematosus (cSLE) presents diagnostic challenges due to its heterogeneous nature, representing an unmet need. To capture this inherent heterogeneity, a diagnostic model which uncovers underlying mechanistic immune perturbations can provide valuable theragnostic insights for SLE.

**Objectives:** We aim to resolve this immunopathogenic complexity with high-dimensional mass cytometry (CyTOF) to study the lupus immunome. Using machine learning, we developed a diagnostic model which utilized mechanistically relevant cell clusters distinguishing patients from controls and was validated with an independent cSLE cohort.

**Methods:** The discovery cohort examined peripheral blood mononuclear cells (PBMCs) from 26 cSLE patients (53 timepoints, median age: 14 years) and 17 age-matched healthy controls using a 43-marker CyTOF panel. Quality check, cell clustering and phenotypic annotation were done using our unsupervised Extended Poly-dimensional Immunome Characterisation (EPIC) pipeline.^1^ Machine learning algorithms [linear SVM, PLS-DA, random forest (RF) using MetaboAnalyst 6.0] identified predictive cell clusters for cSLE to ensure model robustness. Cell frequencies are shown as percentages of total CD45^+^ PBMCs with median and interquartile range (IQR), with statistical significance set at p<0.05 (Mann-Whitney U). Validation was conducted on a new cohort of 18 cSLE patients (median age: 11.5) and 23 age-matched controls using the same CyTOF panel.

**Results:** Multiple immune cell derangements were noted between cSLE and healthy in the discovery cohort. 67 unique cell clusters were derived, of which 26 were significantly different. All 67 cell clusters were used to map the validation cohort. Mapping was automated based on previous expert annotation of the derived cell clusters from unsupervised analysis of discovery data. Validation cohort clusters mirrored accurately the phenotypes of the discovery cohort cell populations. Interestingly, there were increased memory T_regs_ in cSLE (cSLE vs. healthy: 1.16 [0.79-1.92]% vs. 0.48 [0.32- 0.80]%, p<0.0001) but no changes in naive T_regs_. Memory T_reg_-like populations (such as CD3^+^CD4^+^CD45RO^+^CD25^-^Foxp3^+^CTLA^+^) were also higher in cSLE than healthy (2.75 [1.90-4.36] vs. 1.28 [0.83-1.78], p<0.0001). Concurrently, CD8^+^CD45RA^+^BAFF^+^ T cells were raised in cSLE (8.91 [6.78-11.4] vs. 3.22 [2.79-4.86], p<0.0001). These populations, in addition to other unique cell clusters, were used to build a cSLE classification model. Ten immunological features were selected through collective ranking of the most important features generated by machine learning algorithms on discovery cohort data. The average accuracy of discriminating cSLE and healthy based on 100 cross validations (with linear SVM, PLS-DA and RF algorithms) in the discovery cohort is 85.4% (median), (IQR 83.3-85.6%). Sensitivity for the validation cohort is 88.9% (83.3-100%). Further studies will quantitate autoantibody titers (anti-c1q/-nucleosome/-dsDNA/-Smith) for comparison.

**Conclusion:** High-dimensional and machine learning methods were used to craft an immunological classifier for discriminating cSLE from healthy. The ten selected features reflect broad changes in the lupus immunome: perturbed immunoregulation, cytokine production and reduced immune activation threshold. This aids our understanding of SLE immunopathogenesis and can reinforce current diagnostic criteria.

**Patient Consent**

Not applicable (there are no patient data)

**Disclosure of Interest**

None Declared

**Reference**

1. Yeo JG *et al.* The EPIC web-based reference and discovery tool for cytometry data. Nature Biotechnology. 2020 Jun 1;38(6):679-84.

### Treat-to-target

#### OR08 Comparative efficacy of paediatric and adult SLE treat-to-target goals in preventing severe flares and damage accrual

##### Chandni Sarker^1^, Andrea L. Jorgensen^2^, Michael W. Beresford^1,3^, Eve M. D. Smith^1,3^ and on behalf of the UK JSLE Cohort Study

###### ^1^Department of Women’s & Children’s Health, Institute of Life Course and Medical Sciences, University of Liverpool; ^2^Department of Health Data Science, Institute of Population Health, University of Liverpool; ^3^Department of Paediatric Rheumatology, Alder Hey Children’s NHS Foundation Trust Hospital, Liverpool, United Kingdom

####### **Correspondence:** Chandni Sarker

*Pediatric Rheumatology 2024*, **22(2):**PReS24-ABS-1115

**Introduction:** The Paediatric Rheumatology European Society (PReS) has endorsed Childhood Lupus (cSLE) treat-to-target (T2T) definitions include childhood Lupus Low Disease Activity State (cLLDAS), cSLE clinical remission on (cCR) and off-corticosteroids (cCR-0). These targets feature weight-based corticosteroid dosing to avoid inappropriately high corticosteroid doses in children. Additionally, the stricter second remission target, cCR-0, aims for steroid discontinuation.

**Objectives:** To compare cSLE targets (cLLDAS, cCR, cCR-0) with adult-onset SLE (aSLE) targets (LLDAS, DORIS 2021 Remission) for attainability and protection against severe flares and new damage.

**Methods:** Analysis included UK JSLE Cohort Study patients (*n*=430), <18 years at diagnosis, with ≥4 ACR criteria for SLE. Attainability and time to targets were described, with Wilcoxon signed-rank tests used to test for differences in time to target attainment between cSLE and aSLE targets. Association between attainment of cSLE- and aSLE-specific targets and both new damage and severe flare was explored using Prentice-Williams-Peterson (PWP) gap-time models. Severe flare was defined by a BILAG score A or B in any organ domain. New damage was defined by an increase of SDI score by ≥1 unit. Student’s t-tests for dependent samples were conducted to compare the hazard ratios (HRs) obtained from the PWP gap-time models for cSLE versus aSLE targets.

**Results:** A comparable number of patients were found to attain cLLDAS (*n*=290, 67%) and LLDAS (*n*=293, 68%), cCR (*n*=249, 58%) and DORIS 2021 Remission (*n*=261, 61%). Median time-to-target (months) was significantly faster for aSLE-specific targets (LLDAS: 17.4, DORIS 2021 Remission: 19.3) compared to cSLE-specific targets (cLLDAS: 18.4, cCR: 20.4, cCR-0: 23.4, all *p*<0.001). Childhood SLE targets were less attainable than aSLE targets: cCR 796 visits vs DORIS remission 848 visits, with corticosteroid dosage representing the main barrier to paediatric-specific target attainment. All cSLE and aSLE target attainment led to a comparable reduction in the hazards of severe flare and new damage. For example, the hazards of severe flare when either cLLDAS and LLDAS were attained reduced by around 82% (cLLDAS: HR 0.18, CI 0.14, 0.23; LLDAS: HR 0.18, CI 0.14, 0.24, *p*>0.05). The hazards of new damage was comparable with cLLDAS (HR 0.22, CI 0.11, 0.44), and LLDAS attainment (HR 0.24, CI 0.13, 0.46, *p*>0.05). Similarly, the hazards of severe flare and new damage was comparable when cCR and DORIS 2021 Remission were attained (all *p*>0.05).

**Conclusion:** The study highlighted that cSLE and aSLE targets performed comparably in reducing severe flare and new damage, with cSLE targets preventing inappropriate target attainment.

**Date of birth::** novembre 2

**Patient Consent**

Yes, I received consent

**Disclosure of Interest**

None Declared

### Psycho-social aspects and rehabilitation

#### OR09 Predictors of cognitive outcome in children and adolescents with Multisystem Inflammatory Syndrome (MIS-C)

##### David Gosar^1^, Mojca Zajc Avramovič^2^, Nina Emeršič^2^, Damjan Osredkar^1^, Tadej Avčin^2,3^

###### ^1^Department of Child, Adolescent and Developmental Neurology; ^2^Department of Allergology, Rheumatology and Clinical Immunology, University Children’s Hospital Ljubljana; ^3^Faculty of Medicine, Univesity of Ljubljana, Ljubljana, Slovenia

####### **Correspondence:** David Gosar

*Pediatric Rheumatology 2024*, **22(2):**PReS24-ABS-1391

**Introduction:** Few studies exist regarding the psychological outcome of MIS-C, mostly showing specific deficits in the domain of working memory (Otten et al, 2023; Rollins et al., 2023). None included predictors of outcome in specific cognitive domains.

**Objectives:** We sought to assess the cognitive profile of MIS-C patients and relate cognitive outcome to clinical and biochemical markers of disease severity.

**Methods:** At our center neuropsychological assessment was performed in 60/78 of MIS-C patients (78%) between the ages of 2 and 18 (M = 9.81 yr) six months post-discharge (SD = 3.5 months) and in 40 participants from a control group (M = 10.89 yr). When comparing standardized scores across cognitive domains (verbal and visuo-spatial ability, speed of information processing, working memory, executive functioning, verbal and non-verbal learning) between groups using Bayesian regression, we used entropy weighting to take into account demographic factors. Within the clinical group we also examined the predictive power of clinical and biochemical markers during the acute phase of the disease (peak CRP, D-dimer, Troponin, ProBNP, Ferritin, IL-6 and lowest Hb and albumin levels) for predicting the cognitive outcome within the MIS-C group across cognitive domains, with and without controlling for age, gender, BMI and depressive symptoms.

**Results:** Patients with MIS-C displayed a cognitive profile with at least moderate reductions (Cohen *d* > 0.50) in the domains of verbal and non-verbal working memory, verbal learning, speed of information processing and verbal ability. Reduced Hb and albumin levels predicted deficits in working memory and speed of information processing, while the latter were also predicted by higher levels of troponin. Higher peak troponin and proBNP levels also predicted poored executive functioning, while deficits in verbal learning and verbal recall were more closely related to higher levels of D-dimer and lower peak levels of IL-6. Finally, longer duration of disease, as measured by the time required for troponin and proBNP normalization, predicted greater verbal learning and executive functioning deficits.

**Conclusion:** A significant proportion of MIS-C patients has deficits in specific cognitive domains, associated with markers of disease severity. We hope our findings provide additional impetus for research on the different mechanism behind these documented cognitive deficits as proposed by Pan et al. (2024).

**Patient Consent**

Yes, I received consent

**Disclosure of Interest**

None Declared

**References**

1. Rollins, C. K., Calderon, J., Wypij, D., Taylor, A. M., Davalji Kanjiker, T. S., Rohde, J. S., Maiman, M., Zambrano, L. D., Newhams, M. M., Rodriguez, S., Hart, N., Worhach, J., Kucukak, S., Poussaint, T. Y., Son, M. B. F., Friedman, M. L., Gertz, S. J., Hobbs, C. V., Kong, M., Maddux, A. B., … Overcoming COVID-19 Investigators (2023). Neurological and Psychological Sequelae Associated With Multisystem Inflammatory Syndrome in Children. *JAMA network open*, *6*(7), e2324369. 10.1001/jamanetworkopen.2023.24369.
2. Pan T, Gallo ME, Donald KA, Webb K, Bath KG. Elevated risk for psychiatric outcomes in pediatric patients with Multisystem Inflammatory Syndrome (MIS-C): A review of neuroinflammatory and psychosocial stressors. Brain Behav Immun Health. 2024 Mar 18;38:100760. 10.1016/j.bbih.2024.100760. PMID: 38586284; PMCID: PMC10992702.
3. Otten, M. H., Buysse, C. M. P., Buddingh, E. P., Terheggen-Lagro, S. W. J., von Asmuth, E. G. J., … Dulfer, K. (2023). Neurocognitive, Psychosocial, and Quality of Life Outcomes After Multisystem Inflammatory Syndrome in Children Admitted to the PICU. *Pediatric critical care medicine : a journal of the Society of Critical Care Medicine and the World Federation of Pediatric Intensive and Critical Care Societies*, *24*(4), 289–300. 10.1097/PCC.0000000000003180.

### Macrophage activation syndrome

#### OR10 Traditional laboratory parameters and IFN-G related biomarkers for diagnosis and management of MAS

##### Arianna De Matteis^1^, Manuela Pardeo^1^, Ivan Caiello^2^, Valentina Matteo^2^, Denise Pires Marafon^1^, Giulia Marucci^1^, Emanuela Sacco^1^, Francesca Minoia^3^, Francesco Licciardi^4^, Angela Miniaci^5^, Ilaria Maccora^6^, Clotilde Alizzi^7^, Giusi Prencipe^2^, Fabrizio De Benedetti^1^, Claudia Bracaglia^1^

###### ^1^Division of Rheumatology, ERN-RITA center; ^2^Laboratory of Immuno-Rheumatology, ERN-RITA center, IRCCS Ospedale Pediatrico Bambino Gesù, Rome; ^3^Pediatric Rheumatology, Fondazione IRCCS Ca’ Grande Ospedale Maggiore Policlinico, Milan; ^4^Department of Pediatrics and Infectious Diseases, School of Medicine, University of Turin, Regina Margherita Children’s Hospital, Turin; ^5^Department of Pediatrics, University of Bologna, S. Orsola-Malpighi Hospital, Bologna; ^6^Pediatric Rheumatology Unit, Meyer Children’s University Hospital, Florence; ^7^University Department Pro.Sa.M.I. “G. D’Alessandro”, University of Palermo, Palermo, Italy

####### **Correspondence:** Claudia Bracaglia

*Pediatric Rheumatology 2024*, **22(2):**PReS24-ABS-1669

**Introduction:** Macrophage activation syndrome (MAS) is a life-threatening complication of Still’s disease (SD). IFNϒ plays a major role in the pathogenesis of MAS. Early diagnosis is still challenging and no prognostic factors have been identified

**Objectives:** To evaluate diagnostic and prognostic value of traditional laboratory parameters (TLP) of hyperinflammation (e.g. ferritin, cell count, transaminases, LDH) and of the IFNϒ related biomarkers (RB) (IL-18, CXCL9 and neopterin) in patients with MAS in the context of SD.

**Methods:** We enrolled 65 children (41 with MAS and 24 with SD). In SD group TLP and IFNϒ RB were collected during active disease (T0). In MAS group TLP and IFNϒ RB were collected at T0 and 5-15 days from T0 (T1).

**Results:** As expected, at T0 ferritin, platelet count (PLT), AST, triglycerides, fibrinogen, lymphocyte count, LDH, CXCL9, IL-18 and neopterin were significantly higher in MAS than SD. Receiving operating characteristic (ROC) curve for each IFNϒ RB were able to discriminate MAS from active SD (p<0.0001). We identified cut-off values with best sensitivity (se) and specificity (sp). We designed the MAS clinical severity score (MCSS) based on dose and duration of glucocorticoids (GCs), on the treatments used, the length of hospitalization, the access to intensive care unit and death. MCSS ranged from 0 to 8. The MAS group was stratified in 71% with mild MAS (0-4) and 29% with severe MAS (5-8). At T0 higher ferritin, AST and LDH levels, lower PLT and fibrinogen and higher IFNϒ RB levels were associated with severe MAS. ROC curves of ferritin, PLT, AST, fibrinogen, LDH, IL18, CXCL9 and neopterin discriminate severe from mild disease. A prognostic score based on baseline values (CXCL9 >1750 pg/ml and any 2 of the following: PLT <250 x10^9/liter or ferritin >4500 ng/ml or fibrinogen < 340 mg/dl or LDH > 1200 U/L) identified severe MAS with a se of 100% and a sp of 74%. The trend from T0 to T1 showed a significant reduction of IFNϒ RB. Noteworthily, at T1 (5-15 days from baseline) only higher levels of ferritin (*p*=0.02) and CXCL9 (p<0.0001) were significantly associated with longer time to achieve MAS remission. For Kaplan Meier survival analysis we chose for ferritin a cut-off of 684 ng/ml (as per 2016 MAS criteria) and for CXCL9 of 830 pg/ml (the value with the best diagnostic se and sp). The probability of achieving MAS remission within 2 months with CXCL9 levels <830 pg/ml was 9.4 times higher than with CXCL9 levels >830 pg/ml (CI 95 1.78-40.46; *p* = 0.01).

**Conclusion:** Lymphocyte count, LDH and IFNϒ related biomarkers may be helpful for the diagnosis of MAS, but their integration in the current criteria require additional analyses. TLP and IFNϒ related biomarkers are different in patients with severe compared to those with mild MAS. CXCL9 levels at baseline and at T1 are able to predict MAS severity and time to MAS remission .

**Trial registration identifying number:** NA

**Patient Consent**

Yes, I received consent

**Disclosure of Interest**

A. De Matteis: None Declared, M. Pardeo: None Declared, I. Caiello: None Declared, V. Matteo: None Declared, D. Pires Marafon: None Declared, G. Marucci: None Declared, E. Sacco: None Declared, F. Minoia: None Declared, F. Licciardi: None Declared, A. Miniaci: None Declared, I. Maccora: None Declared, C. Alizzi: None Declared, G. Prencipe: None Declared, F. De Benedetti Grant / Research Support with: Abbvie, Novimmune, Novartis, Roche, Sanofi-Aventis, Sobi, Regeneron, Elixiron and Zydus, C. Bracaglia Consultant with: Sobi and Novartis, Speaker Bureau with: GSK

### Macrophage activation syndrome

#### OR11 Performance of the 2016 criteria in recognizing macrophage activation syndrome in multisistem inflammatory syndrome in children (MIS-C): data from the hyper-PED-COVID registry

##### Federica Lucioni^1^, Roberta Caorsi^2^, Alessandro Consolaro^3, 4^, Francesca Bovis^4^, Camilla Speziani^4^, Betul Sozeri^5^, Kadir Ulu^5^, Lucio Verdoni^6^, Gabriele Simonini^7^, Alenka Gagro^8^, Kamrul Lalila^9^, Taciana de Albuquerque Pedrosa Fernandez^10^, Francesco La Torre^11^, Ana Victoria Villareal Trevino ^12^, Sarka Fingerhutova^13^, Ekaterina Alexeeva^14^, Nicolino Ruperto^3, 15^, Angelo Ravelli^3, 4^, Marco Gattorno^3^, Francesca Minoia^1^

###### ^1^Fondazione IRCCS Ca’ Granda Ospedale Maggiore Policlinico, Milano; ^2^IRCCS Istituto Giannina Gaslini; ^3^IRCCS Istituto Giannina Gaslini; ^4^Università degli studi di Genova, Genoa, Italy; ^5^Department of Pediatric Rheumatology, Health Sciences University, Umraniye Training and Research Hospital, Istanbul, Türkiye; ^6^ASST Papa Giovanni XXIII, Bergamo; ^7^IRCCS Meyer Children’s Hospital, florence, Italy; ^8^Children’s Hospital Zagreb, Zagreb, Croatia; ^9^Bangabandhu Sheikh Mujib Medical University (BSMMU), Dhaka, Bangladesh; ^10^Paediatric, Hospital das Clínicas - Botucatu Medicine University, UNESP,, Botucatu, Brazil; ^11^Ospedale Pediatrico Giovanni XXIII , Bari, Italy; ^12^Hospital Regional Materno Infantil de Alta Especialidad,, Guadalupe, Mexico; ^13^General University Hospital and 1st Faculty of Medicine, Charles University, Prague, Czech Republic; ^14^The Federal State Autonomous Institution “National Medical Research Center for Children’s Health” of the Ministry of Health of the Russian Federation, Moscow, Russian Federation; ^15^IRCCS Istituto Giannina Gaslini, Pediatric Rheumatology International Trial Organization, Genoa, Italy

####### **Correspondence:** Federica Lucioni

*Pediatric Rheumatology 2024*, **22(2):**PReS24-ABS-1711

**Introduction:** Macrophage activation syndrome (MAS) has been reported as a complication of Multisystem Inflammatory Syndrome in Children (MIS-C) in up to 20-50% of cases. As clinical and laboratory features of MIS-C partially overlap with MAS, diagnosis may be challenging. The 2016 classification criteria for MAS in systemic juvenile idiopathic arthritis (sJIA) have been largely used to diagnose MAS in MIS-C; however, their performance has never been evaluated in MIS-C and no specific diagnostic criteria for MAS in MIS-C exist.

**Objectives:** To evaluate the performance of 2016 classification criteria in recognizing MAS in patients with MIS-C.

**Methods:** The HyperPED-COVID is the largest international registry of patients with MIS-C. In the case reported form, clinicians were asked to specify if MIS-C patients developed MAS and to provide clinical and laboratory data at MIS-C and MAS onset. Chi-square, Fisher and Mann-Whitney test were used, as appropriate, to compare patients with and without MAS. Sensitivity (SE), specificity (SP) and area under the curve (AUC) were calculated to test the performance of the 2016 criteria at MAS onset and a multivariate analysis was performed to evaluate strength of association between variables and the diagnosis of MAS.

**Results:** Currently, data regarding 1019 patients with MIS-C were collected in the HyperPED-COVID registry; in 82 cases (8.0%) a diagnosis of MAS was made by the caring physician. Patients with MAS were older (9.0 vs 7.9 years, p 0.022), with a longer disease duration (9 vs 5 days, p <.001) and a higher rate of myocardial dysfunction (40% vs 19%, p <.0001) at MIS-C onset. Lymphadenopathy, hepatomegaly and splenomegaly were more frequently reported in MAS than in non-MAS patients, albeit in low percentages (16%, 28% and 18%, respectively). At MAS onset, patients presented higher levels of ferritin (1446 vs 403 ng/ml, p <.0001), triglycerides (235 vs 186 mg/dl, p <.0001), liver enzymes (AST 60 vs 35 U/l, p <.0001) and lower platelet counts (133 vs 193 x 10^9^/l, p <.0001) and fibrinogen (463 vs 543 mg/dl, p 0.004) compared to acute MIS-C. The 2016 criteria had a SE of 0.80, a SP of 0.83 and an AUC of 0.81 (p<.0001) in recognizing MAS from acute MIS-C. Ferritin and triglycerides resulted the strongest variables associated with the diagnosis of MAS in multivariate analysis. Despite a more aggressive treatment, especially with steroids (99% vs 80%, p <.0001) and anakinra (40% vs 9%, p <.0001), patients with MAS required more frequently a circulatory support (40 % vs 25%, p 0.005), with higher mortality (3.7% vs 0.6%, p 0.003).

**Conclusion:** MAS is associated with older age, longer disease duration and a more severe MIS-C phenotype and can further complicate the course of MIS-C, increasing morbidity and mortality. MAS prompt recognition is crucial and the 2016 criteria could represent a valid aid in clinical practice.

**Patient Consent**

Yes, I received consent

**Disclosure of Interest**

None Declared

### Miscellaneous rheumatic diseases

#### OR12 A rare disease with many faces: a multicenter registry of IGG4-related disease in children

##### Ummusen Kaya Akca^1^, Hulya Kose^2^, Tuba Kurt^3^, Kadir Ulu^4^, Vafa Guliyeva^5^, Gulsah Kılbas^6^, Ceyda Arslanoglu^7^, Deniz Gezgin Yildirim^8^, Selcan Demir^9^, Sezgin Sahin^10^, Aysenur Pac Kısaarslan^7^, Belde Kasap Demir^11^, Hafize E. Sonmez^12^, Oya Koker^13^, Gozde K. Yardimci^14^, Mustafa Ekici^14^, Sara S. Kilic^2^, Banu Celikel Acar^3^, Betul Sozeri^4^, Nuray Aktay Ayaz^5^, Selcuk Yuksel^6^, Sevcan A. Bakkaloglu^8^, Ozgur Kasapcopur^10^, Emine A. Ayhan^15^, Omer Karadag^14^, Seza Ozen^1^, Yelda Bilginer^1^

###### ^1^Department of Pediatric Rheumatology, Hacettepe University Faculty of Medicine, Ankara; ^2^Department of Pediatric Immunology and Rheumatology, Uludag University Faculty of Medicine, Bursa; ^3^Department of Pediatric Rheumatology, Ankara City Hospital, University of Health Sciences, Ankara; ^4^Department of Pediatric Rheumatology, Umraniye Training and Research Hospital, University of Health Sciences; ^5^Department of Pediatric Rheumatology, Istanbul University, Istanbul Faculty of Medicine, Istanbul; ^6^Department of Pediatric Rheumatology, Pamukkale University Faculty of Medicine, Denizli; ^7^Department of Pediatric Rheumatology, Erciyes University Faculty of Medicine, Kayseri; ^8^Department of Pediatric Rheumatology, Gazi University Faculty of Medicine, Ankara; ^9^Department of Pediatric Rheumatology, Erzurum Regional Education and Research Hospital, Erzurum; ^10^Department of Pediatric Rheumatology, Istanbul University Cerrahpasa Faculty of Medicine , Istanbul; ^11^Department of Pediatric Nephrology and Rheumatology, Izmir Katip Celebi University Faculty of Medicine, Izmır; ^12^Department of Pediatric Rheumatology, Kocaeli University Faculty of Medicine, Kocaeli; ^13^Department of Pediatric Rheumatology, Marmara University Faculty of Medicine, Istanbul; ^14^Department of Rheumatology; ^15^Department of Pathology, Hacettepe University Faculty of Medicine, Ankara, Türkiye

####### **Correspondence:** Ummusen Kaya Akca

*Pediatric Rheumatology 2024*, **22(2):**PReS24-ABS-1636

**Introduction:** IgG4-related disease (IgG4-RD) is a fibroinflammatory disease, affecting almost any organ. Given its rarity and the paucity of pediatric clinical trials, our knowledge of the diagnosis and management is mainly based on adult experience and reports of case series in children.

**Objectives:** We aimed to report the characteristics of pediatric IgG4-RD through a multicenter registry, to assess disease clusters, and to evaluate the performances of the 2019 American College of Rheumatology and European League Against Rheumatism (ACR/EULAR) classification criteria and the 2020 revised comprehensive diagnostic (RCD) criteria in this cohort.

**Methods:** Data of IgG4-RD patients in 13 pediatric rheumatology centers were recorded to a web-based registration system. The diagnosis of IgG4-RD was made according to the 2011 comprehensive diagnostic criteria. The clinical phenotypes, disease subsets, and sensitivities of the criteria sets were also investigated.

**Results:** Thirty-five children (19 females and 16 males) with IgG4-RD were enrolled. The median age at diagnosis and median follow-up time were 13.3 (25p-75p; 9.9-15.2) years and 1.8 (25p-75p; 0.6-3.4) years, respectively. The most common organ involvement was ocular findings in 21 (60%) patients, followed by lymph nodes in 12 (34.3%), musculoskeletal system in 12 (34.3%), neurological system in 9 (25.7%), pancreatic and biliary tract in 8 (22.9%) and lacrimal and major salivary glands in 8 (22.9%) patients. Sixteen patients (45.7%) were classified as having proliferative subtype whereas 19 (54.3%) had fibrotic subtype. We identified three clusters in our study cohort: those with eye involvement (*n*=11, 31.4%), those with eye involvement and neurological findings (*n*=15, 42.9%), and those with pancreato-hepatobiliary disease and lymph node involvement (*n*=9, 25.7%). Serum IgG4 levels were high in 19 out of 28 patients (67.8%). Biopsy was performed in 31 patients (88.6%) in diagnostic process and 27 of them (87.0%) had findings associated with IgG4-RD. Dense lymphocytic infiltrate and storiform fibrosis were the most prominent histopathological findings. The sensitivities of the 2019 ACR/EULAR classification criteria and the 2020 RCD criteria were 5.7% and 88.5%, respectively.

All patients except one received corticosteroid treatment, and azathioprine was the most preferred drug as a steroid-sparing agent. Complete response was observed in 33.3% of the patients, partial response in 23.3%, and stable disease in 6.6%. Relapse occurred in 11 patients (31.4%).

**Conclusion:** IgG4-RD has a wide variety of clinical manifestations, however in children the most common presentation was orbital involvement. The 2020 RCD criteria had a better performance whereas the 2019 ACR/EULAR classification criteria performed poorly in pediatric patients.

**Date of birth::** janvier 01

**Patient Consent**

Yes, I received consent

**Disclosure of Interest**

None Declared

### Vasculitides

#### OR13 The Kiwi registry: clinical and demographic characteristics of children with Kawasaki disease

##### Maria Vincenza Mastrolia^1,2^, Vignesh Pandiarajan^3^, Marco Cattalini^4^, Andrea Taddio^5^, Stefania Vergnano^6^, Jordi Anton^7^, Watchareewan Sontichai^8^, Betul Sozeri^9^, Seza Ozen^10^, Silvia Rosina^11^, Judith Sánchez-Manubens^12^, Ruby Haviv^13^, Priyankar Pal^14^, Adriana Rodrigues Fonseca^15^, Alenka Gagro^16^, Arvind Bagga^17^, Lucio Verdoni^18^, Davide Montin^19^, Maria Cristina Maggio^20^, Marco Garrone^21^, Elisa Patrone^21^, Nicolino Ruperto^21^, Gabriele Simonini^1, 2^ and for the Pediatric Rheumatology European Society (PReS) and the Paediatric Rheumatology International Trial Organization (PRINTO)

###### ^1^Meyer Children’s Hospital IRCCS, Rheumatology Unit, ERN ReCONNET center; ^2^University of Florence, NEUROFARBA Department, Firenze, Italy; ^3^Postgraduate Institute of Medical Education and Research, Pediatric Allergy Immunology Unit, Department of Pediatrics, Chandigharh, India; ^4^University of Brescia and Spedali Civili di Brescia, Pediatric Clinic, Brescia; ^5^IRCCS “Burlo Garofolo”, University of Trieste, Institute for Maternal and Child Health, Trieste, Italy; ^6^Bristol Royal Hospital for Children, Department of Pediatric Rheumatology, Bristol, United Kingdom; ^7^Universitat de Barcelona, Hospital Sant Joan de Déu, Department of Pediatric Rheumatology, Barcelona, Spain; ^8^Faculty of Medicine, Chiang Mai University, Division of Rheumatology, Department of Pediatrics, Chiang Mai, Thailand; ^9^Department of Pediatric Rheumatology, Health Sciences University, Umraniye Training and Research Hospital, Instanbul; ^10^Hacettepe University, Department of Pediatric Rheumatology, Ankara, Türkiye; ^11^IRCCS Istituto Giannina Gaslini, UOC Reumatologia e Malattie Autoinfiammatorie, Genoa, Italy; ^12^Reumatologia Pediàtrica - Servei de Medicina Pediàtrica, Hospital Parc Taulí de Sabadell, Barcelona, Spain; ^13^Meir Medical Center, Pediatric Rheumatology Unit, Department of Pediatrics, Kfar Saba, Israel; ^14^Department of Pediatric Rheumatology, Institute of Child Health, Kolkata, India; ^15^Instituto de Puericultura e Pediatria Martagao Gesteira (IPPMG), Universidade Federal do Rio de Janeiro, Rio de Janeiro, Brazil; ^16^Department of Pediatrics, Department of pulmology, allergology, clinical immunology and rheumatology, Children’s Hospital Zagreb, Zagreb, Croatia; ^17^Division of Pediatric Rheumatology - Department of Pediatrics, All India Institute of Medical Sciences, New Delhi, India; ^18^Hospital Papa Giovanni XXIII, Paediatric Department, Bergamo; ^19^Immunology and Rheumatology Unit, Regina Margherita Children Hospital, Turin; ^20^Children Hospital “G. Di Cristina”, Rheumatology Unit, Palermo; ^21^UOSID Trial Center, Rheumatology Unit, PRINTO, IRCCS Giannina Gaslini Institute, Genoa, Italy

####### **Correspondence:** Maria Vincenza Mastrolia

*Pediatric Rheumatology 2024*, **22(2):**PReS24-ABS-1299

**Introduction:** 10-20% of Kawasaki disease (KD) patients are resistant to intravenous immunoglobulins (IVIg). Previous studies on different scoring scales developed by Kobayashi, Egami and Sano had shown a godd sensitivity (77–86%) and specificity (67–86%) in predicting IVIg unresponsiveness in Japanese KD populations^1^. Their predictive value was not confirmed in European, American and other Asian populations. The Kawanet group have proposed a score providing good sensitivity (77%) and acceptable specificity (60%) in a not-Asian KD population^2^.

**Objectives:** The Comparison and performance of Kobayashi and Kawanet IVIg resistance scores in a multi-centric European and North Indian cohort of KaWasaki dIsease, the KIWI study, has the aim to analyze demographic, clinical, biological and echocardiographic variables associated with IVIg resistance and to assess differences in clinical and treatment outcomes in Caucasian and Asian cohorts of KD children.

**Methods:** This is a retrospective-prospective, observational, international and multicenter study of patients with KD according to the American Heart Association (AHA) criteria. The KIWI study was awarded the 2020 PRINTO/PReS grant, enrollment span from April 2022 to January 2024. Data deriving from KD patients diagnosed from January 1^st^, 2015, were collected retrospectively by revising medical charts, new KD diagnosis were collected prospectively during the study period.

**Results:** 612 patients were enrolled with complete data as of January 31st, 2024, from 19 pediatric Rheumatology Units. 372 were male (60.7%), with a median age of 2.4 years. 419 patients (66.0%) were Caucasian, 103 (16.8%) Asian, 19 (3.1%) African and 15 (2.5%) Latin-American. 348 patients (56.9%) presented with complete KD, 234 (38.2%) with incomplete KD, and 19 (3.1%) children reported atypical features. Coronary abnormalities (ectasia) were observed in 90 patients (14.7%), aneurysms in 120 (19.6%) during the disease course. 10 patients (1.6%) developed macrophage activation syndrome (MAS), while 11 (1.8%) presented with Kawasaki disease shock syndrome (KDSS). Mucocutaneous signs were reported in 600 children (98.0%), musculoskeletal signs in 77 (12.6%), lymphadenopathy was observed in 367(60.0%). Gastrointestinal system involvement was recorded in 275 patients (44.9%), and neurological manifestations in 43 cases (7.0%). 595 patients (97.2%) received an IVIg dose at onset,154 (25.2%) received glucocorticoid therapy as first-line treatment. The rate of IVIg resistance in the study population was 21.9% (134). Second-line therapy was administered to 56 patients (41.8%) with glucocorticoids, 15 (11.2%) with anakinra, 11 (8.2%) with infliximab, 4 (3.0%) with cyclosporine, and 1 (0.8%) with tocilizumab. A full recovery was observed in 568 patients (92.8%), while 44 children (7.2%) experienced sequelae. No deaths were reported.

**Conclusion:** Our preliminary results confirm an IVIG resistance rate of 20%. Almost 3% of patients experienced MAS or KDSS. Glucocorticoids were the most commonly used second-line therapy, anakinra represented the most frequently administered biologic drug.

**Trial registration identifying number:** NCT06305611

**Patient Consent**

Yes, I received consent

**Disclosure of Interest**

None Declared

**References**

1. Kobayashi T, et al. Prediction of intravenous immunoglobulin unresponsiveness in patients with Kawasaki disease. Circulation 2006.
2. Piram M, et al. Defining the risk of first intravenous immunoglobulin unresponsiveness in non-Asian patients with Kawasaki disease. Sci Rep. 2020

### Vasculitides

#### OR14 Neonatal-onset vasculitis driven by pathogenic variants in Haematopoietic Cell Kinase (HCK) - a report of two families and a novel mutation

##### Fiona Price-Kuehne^1^, Alice Burleigh^1^, Ebun Omoyinmi^1^, Ying Hong^1^, Gulshan Malik^2^, Gabriela Petrof^3^, Despina Eleftheriou^1,4^, Paul Brogan^1,4^

###### ^1^Infection, Immunity and Inflammation, University College London Institute of Child Health, London; ^2^Paediatrics, Royal Aberdeen Children’s Hospital, Aberdeen; ^3^Dermatology; ^4^Rheumatology, Great Ormond Street Hospital for Children NHS Trust, London, United Kingdom

####### **Correspondence:** Fiona Price-Kuehne

*Pediatric Rheumatology 2024*, **22(2):**PReS24-ABS-1309

**Introduction:** Haematopoietic cell kinase (HCK) is a member of the src-family of tyrosine kinases and is expressed in cells of lymphoid and myeloid lineage. In 2021, a single case report of a mutation in *HCK* leading to pulmonary and cutaneous vasculitis was published (1). We now report on 3 further cases including a family with a novel mutation.

**Objectives:** To determine the cause of severe neonatal-onset vasculitis in 3 cases from 2 unrelated kindreds and to highlight the importance of re-analysis of genetic sequencing in unsolved cases.

**Methods:** Retrospective case note review and whole exome and Sanger sequencing.

**Results:** Proband A, born to unrelated healthy parents, presented with rash at 4 hours of life. Biopsy showed leukocytoclastic vasculitis, the rash persisted, and by 2 years of age she developed pulmonary haemorrhage and splenomegaly. Inflammatory markers were normal and there was no response to immunosuppressive treatment including prednisolone and trials of: ciclosporin; tacrolimus; colchicine; hydroxychloroquine; mycophenolate mofetil; anakinra; adalimumab; and baricitinib. Initial gene panel and later whole-exome sequencing revealed no genetic cause. However, reanalysis using an updated bioinformatics pipeline and referencing a new publication (1), identified a de novo heterozygous *HCK* mutation (c.C1545A; p.Y515X) prompting referral for allogeneic hematopoietic stem cell transplantation.

Proband B and his father presented with rash on the first day of life. Biopsy showed leukocytoclastic vasculitis and the rash recurred through infancy but they did not develop lung disease. Whole exome sequencing of Proband B at the time revealed no genetic cause. However, reanalysis in light of the newly discovered HCK-vasculitis revealed that he and his father also carried a novel rare heterozygous *HCK* mutation (c.A1565T; p.Y522F).

**Conclusion:** The leukocyte signalling molecule HCK relies on an inhibitory tyrosine (Y522) in the c-terminal tail to prevent inappropriate pro-inflammatory signalling. The nonsense mutation in Proband A causes loss of this crucial inhibitory tyrosine, while Family B have a missense mutation causing the inhibitory tyrosine to be replaced with phenylalanine. Since Family B do not have lung involvement and the rash in the father resolved in childhood, we hypothesise that missense mutations in HCK are less deleterious.

In conclusion, we report the second case of the newly-described monogenic HCK-vasculitis, and report a further family with a novel missense mutation driving a self-resolving cutaneous vasculitis. We also highlight the importance of re-analysing genetic sequencing data in unsolved cases. This is especially pertinent in the field of autoinflammation and rare disease where new disease-gene discoveries are made on a year-to-year basis.

**Patient Consent**

Yes, I received consent

**Disclosure of Interest**

None Declared

**Reference**

1. Kanderova V, et al. Early-onset pulmonary and cutaneous vasculitis driven by constitutively active SRC-family kinase HCK. J Allergy Clin Immunol.2022 Apr;149(4):1464-1472.

### JIA (oligo, poly, psoriatic)

#### OR15 Accelerometer-measured movement behaviours among patients with juvenile idiopathic arthritis compared to controls: results of the multicentre actimon study

##### Florian Milatz^1^, Lisa Voigt^2^, Jens Klotsche^1^, Tilmann Kallinich^1,3^, Ralf Trauzeddel^4^, Daniel Windschall^5,6^, Sandra Hansmann^7^, Nadja Baumeister^8^, Johannes-Peter Haas^8^, Moritz Klaas^9^, Hermann J. Girschick^9^, Joachim Peitz-Kornbrust^10^, Alexander Burchartz^11^, Kirsten Minden^1,3^

###### ^1^Deutsches Rheuma-Forschungszentrum Berlin, Berlin; ^2^University Medicine Greifswald, Greifswald; ^3^Charité - Universitätsmedizin Berlin; ^4^Helios Klinikum Berlin-Buch, Berlin; ^5^St. Josef-Stift Sendenhorst, Sendenhorst; ^6^Universität Halle-Wittenberg, Halle; ^7^University of Tuebingen, Tuebingen; ^8^German Centre for Paediatric and Adolescent Rheumatology, Garmisch-Partenkirchen; ^9^Vivantes Klinikum Friedrichshain, Berlin; ^10^Asklepios Kinderklinik Sankt Augustin, Sankt Augustin; ^11^Karlsruhe Institute of Technology, Karlsruhe, Germany

####### **Correspondence:** Florian Milatz

*Pediatric Rheumatology 2024*, **22(2):**PReS24-ABS-1146

**Introduction:** Accurately assessing young peoples’ many spontaneous and impulsive movements in everyday life is a major challenge, but essential for deriving targeted interventions to promote health-enhancing lifestyles. Accelerometers can tackle these challenges; however, to date research on device-measured physical activity (PA) and sedentary behaviour (SB) in JIA patients is limited.

**Objectives:** To examine JIA patients’ movement behaviour in comparison to that of population controls, taking into account internationally recommended criteria and validated cut-points.

**Methods:** JIA patients aged 10 to 20 years recruited within the German multicentre ActiMON study were instructed to wear an ActiGraph accelerometer (Model wGT3X-BT) on the hip for seven consecutive days during waking hours. Data collection and processing were done according to international criteria (e.g. valid wear time ≥ 5 days incl. ≥ 1 weekend day with ≥ 8 hours each) before classifying movements into SB, light PA (LPA), moderate (MPA) and vigorous PA (VPA) [1]. Device-measured data were linked to clinical data from the National Paediatric Rheumatological Database (NPRD). Age- and sex-matched controls were drawn from the nationwide representative MoMo Study, in which the same study protocol and accelerometer processing criteria were used [2]. Analyses were performed under consideration of compositional data analysis (CoDA) approaches to account for relative amounts of movement behaviours in relation to accelerometer wear time.

**Results:** 126 age- and sex-matched pairs fulfilled wear time criteria (mean age 15.0 ± 2.1 years, female 67%, patients’ disease duration 8 ± 4 years, cJADAS-10 2.4 ± 3.0, oligoarthritis 46%, valid wear days 6.6 ± 0.6, mean wear time 14.3 ± 2.0 hours per day). Patients’ body mass index (21.1 ± 4.1), weight (58.5 ± 15.0 kg) and height (166 ± 10 cm) did not differ significantly from those of controls. Overall, 24% of patients and 8% of controls achieved the average amount of ≥60 minutes moderate to vigorous PA per day recommended by the WHO. Relative to their wearing time, patients spent on average more time in SB (86% vs. 78%, *p*<0.001) and VPA (4% vs. 3%, *p*<0.001), but less time in LPA (8% vs. 17%, *p*<0.001) than controls. No differences were found for MPA (2% vs. 2%, *p*=0.230). Time-use composition of movement behaviour did not differ significantly between weekdays and weekends in either group. Differences in composition of time patients spent in movement behaviours were found for gender (*p*=0.040), age (*p*=0.005) and level of fatigue (*p*=0.021), with females, late adolescents and patients with higher fatigue scores being less physically active.

**Conclusion:** Although proportionally more JIA patients achieved the WHO recommended minimum level of PA compared to healthy controls, the majority remained insufficiently physically active while accumulating substantially more time in SB than controls. Targeted interventions to compensate for unfavourable movement behaviours appear to be urgently needed in this patient population.

ActiMON as part of the research network TARISMA was funded by the Federal Ministry of Education and Research (01EC1902F).

**Trial registration identifying number:** DRKS00022258

**Patient Consent**

Not applicable (there are no patient data)

**Disclosure of Interest**

None Declared

**References**

1. Romanzini M, et al. Calibration of ActiGraph GT3X, Actical and RT3 accelerometers in adolescents. Eur J Sport Sci. 2014;14(1):91-9.
2. Burchartz A, et al. Measurement of physical activity and sedentary behavior by accelerometry among a nationwide sample from the KiGGS and MoMo Study: Study Protocol. JMIR Res Protoc 2020;9:e14370.

### Scleroderma and related syndromes

#### OR16 Significant clinical improvement with autologous stem cell transplantation for refractory juvenile-onset systemic sclerosis

##### Kathryn Torok^1^, Paulina Horvei^2^, Jonathan Li^3^, Franziska Rosser^4^, Kirsten Rose-Felker^5^, Vibha Sood^6^, Adam Olsen^7^, Nicole Hogue^2^, Vickie Vandergrift^8^, Lauren Farver^9^, Devin Mcguire^10^, Haley Havrilla^8^, Jessie Alexander^11, 12^, Shawna McIntyre^2^, Paul Szabolcs^2^

###### ^1^Pediatric Rheumatology, University of Pittsburgh, UPMC Children’s Hospital of Pittsburgh; ^2^Pediatric Bone Marrow Transplant and Cellular Therapies; ^3^Pediatric and Internal Medicine; ^4^Pediatric Pulmonology; ^5^Pediatric Cardiology; ^6^Pediatric Gastroenterology; ^7^Pediatric Radiation Oncology; ^8^Pediatric Rheumatology; ^9^Pediatric Physical Therapy; ^10^Pediatric Behavioral Health, University of Pittsburgh, Pittsburgh; ^11^Pediatric Bone Marrow Transplant and Cellular Therapies, Stanford Children’s Hospital, Palo Alto; ^12^Pediatric Bone Marrow Transplant and Cellular Therapies, University of Pittsburgh, UPMC Children’s Hospital of Pittsburgh, Pittsburgh, United States

####### **Correspondence:** Jonathan Li

*Pediatric Rheumatology 2024*, **22(2):**PReS24-ABS-1378

**Introduction**

Juvenile-onset systemic sclerosis (jSSc) carries a significant morbidity and mortality among the pediatric rheumatologic diseases and effective therapies are limited. Autologous Stem Cell Transplant (ASCT) has been a promising treatment option in adult SSc, but data is limited in jSSc.

**Objectives**

Herein, we report the outcomes of the first seven jSSc patients after receiving ASCT at our center.

**Methods**

Patients with severe and/or progressive jSSc were referred to our multidisciplinary pediatric scleroderma center, and if deemed appropriate and eligible by the care team were enrolled in our SSc ASCT protocol (NCT03630211). Clinical outcomes and patient reported measures were collected at baseline (pre-ASCT) and at routine intervals post-ASCT.

**Results**

Seven jSSc onset patients were eligible and received ASCT. They all had disease refractory to >3 DMARDs and met criteria for ASCT by severity and/or continued activity of lung, skin or musculoskeletal disease. Two patients had pulmonary arterial hypertension (an exclusion for some SSc protocols). Most patients were female (70%), non-Hispanic (63%), and Black (57%). Mean age of disease onset was 12.9 ± 3.0 years and mean age of ASCT was 18.3 ± 1.9 years. The majority had diffuse cutaneous disease, average mRSS of 22 (+10) with features of overlap (myositis and arthritis). Lung function was impacted in all, either from interstitial lung disease and/or myopathy with a mean baseline FVC of 65% (+ 23%) and DLCO 52% (+ 15%). GI involvement was moderate to severe, with average LES manometry of 12.8 mmHg (+ 6), below normal pressure.

Overall conditioning was tolerated well and on average neutrophils engrafted on day 13 (± 6 days) and platelets engrafted day 22 (± 7 days) post-ASCT. All 7 patients had 3 month time point data collection at the time of this report. The mRSS decreased by 70% on average for all patients at the 3 month post-ASCT visit and those with 1 year follow-up (*n*=5 patients) it showed a 90% decrease from baseline (average 22 to 3). Improvement in oral aperture, finger to palm distance and general joint mobility and associated C-HAQ scores paralleled these improvements in skin score. Lung function was stable to improved and GI outcomes were improved. Patient overall Scleroderma Health Assessment Questionnaire (S-HAQ) VAS of overall Disease impact decreased significantly from an average of 1.74 (+ 0.82 ) to 0.38 (+ 0.45), where a lower score signifies less interference of the disease with everyday life.

**Conclusion**

Among our cohort of refractory jSSc patients with moderate-severe disease, ASCT is a safe and effective intervention that provided sustained global disease modifying improvement after transplant across many organ systems and importantly in patient quality of life.

**Trial registration identifying number:** Autologous Stem Cell Transplantation in Patients With Systemic Sclerosis - Full Text View - ClinicalTrials.gov

NCT03630211

**Patient Consent**

Yes, I received consent

**Disclosure of Interest**

None Declared

### Systemic JIA

#### OR17 EULAR/PReS recommendations for the diagnosis and management of still’s disease, comprising systemic juvenile idiopathic arthritis and adult-onset still’s disease

##### Bruno Fautrel^1,2^, Stephane Mitrovic ^1^, Arianna de Matteis^3^, Sara Bindoli^4^, Jordi Anton^5^, Alexandre Belot^6^, Claudia Bracaglia^3^, Tamas Constantin^7^, Lorenzo Dagna^8^, Alessandro De Bartolo^9^, Eugen Feist^10^, Dirk Foell^11^, Marco Gattorno^12^, Sophie Georgin-Lavialle^13^, Roberto Giacomelli^14^, Alexei Grom^15^, Yvan Jamilloux^16^, Katerina Laskari^17^, Calin Lazar^18^, Francesca Minoia^19^, Peter A. Nigrovic^20^, Seza Ozen^21^, Pierre Quartier^22^, Piero Ruscitti^23^, Erdal Sag^21^, Sinisa Savic^24^, Marie-Elise Truchetet^25^, Sebastiaan J. Vastert^26^, Tanita Wilhelmer^27^, Carine Wouters^28^, Fabrizio De benedetti^3^ and on behalf of the EULAR/PReS QoC011 Task Force

###### ^1^Sorbonne Université; ^2^Pitié-Salpêtrière Hospital, Paris, France; ^3^Ospedale Bambino Gesù, Roma; ^4^Padova University, padova, Italy; ^5^Hospital Sant Joan de Déu, barcellona, Spain; ^6^Hospices Civils de Lyon, Lyon, France, Lyon, France; ^7^Semmelweis University, Budapest, Hungary; ^8^IRCCS San Raffaele Hospital; ^9^Patient representative , Milano, Italy; ^10^Helios Department for Rheumatology and Clinical Immunology, Vogelsang-Gommern; ^11^University Children’s Hospital Muenster, mìMunster, Germany; ^12^IRCCS Istituto G. Gaslini, Genova, Italy; ^13^Sorbonne University, Paris, France; ^14^Fondazione Policlinico Universitario, Campus Bio-Medico, Roma, Italy; ^15^Cincinnati Children’s Hospital Medical Center, Cincinnati, United States; ^16^University Hospital Croix-Rousse, Hospices Civils de Lyon, Lyon, France; ^17^National & Kapodistrian University of Athens Medical School, Athens, Greece; ^18^University of Medicine and Pharmacy Iuliu Hatieganu, Cluj-Napoca, Romania; ^19^Fondazione IRCCS Ca’ Granda Ospedale Maggiore Policlinico, Milano, Italy; ^20^Boston Children’s Hospital, Boston, United States; ^21^Hacettepe University, ankara, Türkiye; ^22^Necker-Enfants Malades hospital, Paris, France; ^23^University of L’Aquila, L’Aquila, Italy; ^24^University of Leeds, Leeds, United Kingdom; ^25^Université de Bordeaux, Bordeaux, France; ^26^Wilhelmina Children’s Hospital, Utrecht, Netherlands; ^27^Patient representative , Dornbirn, Austria; ^28^UZ Leuven, Leuven, Belgium

####### **Correspondence:** Fabrizio De benedetti

*Pediatric Rheumatology 2024*, **22(2):**PReS24-ABS-1721

**Introduction:** Systemic juvenile idiopathic arthritis (sJIA) and adult-onset Still’s disease (AOSD) were described as the same disease continuum. However, subsequently, an arbitrary cut-off fo 16 years of age at onset has been applies. The two entities share common features such as the four major symptoms: recurrent spiking fever, skin rash, arthralgia and/or arthritis, and high levels of inflammation. Despite being progressively considered the same disease, a common approach for diagnosis and management is still missing.

**Objectives:** To establish the first consensus recommendations for the diagnosis and management of children and adults with Still’s disease.

**Methods:** In May 2022, EULAR and PReS endorsed a proposal for a joint task force (TF) to develop recommendations for the diagnosis and management of sJIA and AOSD. The TF agreed during a first meeting to address four topics: similarity between sJIA and AOSD, diagnostic biomarkers, therapeutic targets and strategies, and complications including macrophage activation syndrome (MAS). Systematic literature reviews were conducted accordingly.

**Results**

The TF based their recommendations on four overarching principles that highlighted notably that sJIA and AOSD are one disease, to be designated by one name, Still’s disease, and that T2T has to be applied and the need for prompt detection and rapid treatment of MAS. Fourteen specific recommendations were issued. In the absence of a COMMON validated set of diagnostic/classification criteria fro children and adult with SD, the task force provided operational definition of key diagnostic features and highlighted that arthritis is not necessary for the diagnosis. Two therapeutic targets were defined: clinically inactive disease (CID) and remission, i.e., CID maintained for at least 6 months. The optimal therapeutic strategy relies on early use of IL-1 or IL-6 inhibitors associated to short duration glucocorticoid (GC). A treatment algorithm is proposed. MAS treatment should rely on high-dose GCs, IL-1 inhibitors, ciclosporin and IFNγ inhibitors. A specific concern rose recently with cases of severe lung disease in children with Still’s disease, for which T cell directed immunosuppressant are suggested. The recommendations emphasized the key role of expert centers for difficult-to-treat patients. All overarching principles and recommendations were agreed by over 80% of the TF experts with a high level of agreement.

**Conclusion:** These recommendations are the first consensus for the diagnosis and management of children and adults with Still’s disease.

**Patient Consent**

Not applicable (there are no patient data)

**Disclosure of Interest**

None Declared

### Systemic JIA

#### OR18 Biomarker-guided treatment-and-stop-strategy for recombinant il-1receptor antagonist (ANAKINRA) in patients with systemic juvenile idiopathic arthritis

##### Remco Erkens^1,2^, Gerda Den Engelsman^1^, Sytze De Roock^1,2^, Dörte Hamann^2^, Dieneke Schonenberg-Meinema^3^, Merlijn Van den Berg^3^, Mariken Gruppen^3^, Wineke Armbrust^4^, Elizabeth Legger^4^, Sylvia Kamphuis^5^, Marleen Verkaaik^5^, Ellen Schatorjé^6^, Esther Hoppenreijs^6^, Thomas Vogl^7^, Johannes Roth^7^, Petra Hissink Muller^8^, Joost Swart^1^, Marc Jansen^1^, Jorg Van Loosdregt^2^, Bas Vastert^1,2^

###### ^1^Division of Pediatric Rheumatology and Immunology, Wilhelmina Children’s Hospital; ^2^Center for Translational Immunology, University Medical Center Utrecht, Utrecht; ^3^Division of Pediatric Rheumatology and Immunology, Amsterdam University Medical Center, Amsterdam; ^4^Division of Pediatric Rheumatology and Immunology, University Medical Center Groningen, Groningen; ^5^Division of Pediatric Rheumatology and Immunology, Erasmus Medical Center, Rotterdam; ^6^Division of Pediatric Rheumatology and Immunology, University Medical Center Radboud, Nijmegen, Netherlands; ^7^Institute of Immunology, University of Münster, Münster, Germany; ^8^Division of Pediatric Rheumatology and Immunology, Leiden University Medical Center, Leiden, Netherlands

####### **Correspondence:** Bas Vastert

*Pediatric Rheumatology 2024*, **22(2):**PReS24-ABS-1686

**Introduction:** Early initiation of treatment with the recombinant Interleukin 1 receptor antagonist (IL-1Ra), anakinra, in new-onset steroid naïve systemic Juvenile Idiopathic Arthritis (sJIA) patients is safe and results in quick resolution of fever and systemic inflammation and remarkably high response rates in the majority of patients[1-2]. Although anakinra is very effective and safe it is relatively expensive, not without side effects and the daily burden of injections can be high. Therefore, we previously investigated a taper-and-stop strategy for anakinra treatment in sJIA where patients with clinically inactive disease (CID) at time point ~3 months after the start of anakinra were tapered to alternate day administration and subsequently stopped. This resulted in around 50% of patients in remission without medication after 1 year[1,2]. Retrospectively, the patients that relapsed in that cohort showed significantly higher levels of IL-18 at time point 3 months than the patients with a successful taper and stop attempt. Consequently biomarker guidence has the potential to improve the taper and stop strategy.

**Objectives:** Here we describe the first results of the Dutch multi-center, prospective, intervention study aiming to develop a safe and personalized ‘taper and stop strategy’ for sJIA patients with a complete response with 1^st^ line use of rIL-1RA after 3 months of treatment.

**Methods:** The study consisted of an open-label lead-in part, in which patients with new-onset, biological and steroid naïve sJIA, from 6 centers in the Netherlands were included. SJIA diagnosis was in accordance with the criteria proposed by Martini *et al* [3]. Serum levels of IL-18 were measured by Luminex technology in the diagnostic laboratory of the UMC Utrecht, using standard operating protocols for processing and shipment of samples[1]. All patients received anakinra as first-line therapy in accordance with the Dutch national protocol with frequent follow-up and sampling. Only patients that had a good initial clinical response and achievement of CID at T=3 months after start on rIL-1RA mono-therapy were allowed to enter the intervention part of the study: these patients were then assessed every month for both clinical response and measurement of IL-18. Patients with IL-18 <1200 pg/mL were switched to an alternate day regimen and the rIL-1RA was subsequently stopped 1 month later. If the IL-18 remained above the threshold of 1200 pg/mL, rIL-1RA was continued in a daily dose. Patients with CID at t=9 months after start of treatment were tapered (1 month alternate day) and stopped regardless of IL-18 levels. Patients were followed up to 2 years after start of treatment.

**Results:** In total, 67 patients were enrolled in the study of which 44 showed CID on rIL-1RA monotherapy at t=3 months, allowing entry in the intervention phase of the trial. The median age at the start of treatment was 9 years (range 0.8-16.5) and 56% were female. The 23 patients that were not enrolled in the intervention phase, due to active disease while on rIL-1RA, due to the start of concomitant steroids or to a switch of therapy, were prospectively followed up for 2 years. In total, 6 patients developed MAS before the taper and stop phase, 9 had continued or relapsing disease, 5 experienced adverse events of the therapy and 3 were unable to continue on anakinra due to the burden of injections. There was no significant difference in the clinical and laboratory characteristics between the intervention (CID) and the non-intervention (more refractory course up to 3 months) group at t=0 except for the percentage of females, 43% and 79% respectively (*p*=0.004). In the intervention group, 75% (33/44) of sJIA patient were successfully tapered and stopped, with a sustained clinical response at t=12 months. This is significantly (*p*=0,043) better than the percentage of successful taper and stop in our historic cohort (47%, 7/15)[1].

**Conclusion:** Our prospective, multicenter intervention trial shows that IL-18 with a cut-of value of 1200 pg/mL is helpful in successful tapering and stop of rIL-1RA in well-responding patients.

**Patient Consent**

Yes, I received consent

**Disclosure of Interest**

R. Erkens: None Declared, G. Den Engelsman: None Declared, S. De Roock: None Declared, D. Hamann: None Declared, D. Schonenberg-Meinema: None Declared, M. Van den Berg: None Declared, M. Gruppen: None Declared, W. Armbrust: None Declared, E. Legger: None Declared, S. Kamphuis: None Declared, M. Verkaaik: None Declared, E. Schatorjé: None Declared, E. Hoppenreijs: None Declared, T. Vogl: None Declared, J. Roth: None Declared, P. Hissink Muller: None Declared, J. Swart: None Declared, M. Jansen: None Declared, J. Van Loosdregt: None Declared, B. Vastert Grant / Research Support with: SOBI, Consultant with: SOBI and Novartis

**References**

1. PMID: 24757154
2. PMID: 30848528
3. PMID: 30275259

### Uveitis

#### OR19 Effectiveness and safety of baricitinib for the treatment of juvenile idiopathic arthritis associated uveitis or chronic anterior antinuclear antibody positive uveitis in children

##### Athimalaipet Ramanan^1^, Catherine Guly^2^, Gabriele Simonini^3,4^, Stuart Keller^5^, Priyanka Sen^5^, Thorsten Holzkaemper^5^, Pierre Quartier^6,7,8^

###### ^1^University of Bristol; ^2^University Hospitals Bristol and Weston NHS Foundation Trust, Bristol, United Kingdom; ^3^Meyer Children’s Hospital IRCCS; ^4^University of Florence, Florence, Italy; ^5^Eli Lilly and Company, Indianapolis, United States; ^6^Pediatric Immunology, Necker-Enfants Malades Hospital, Assistance Publique-Hopitaux de Paris; ^7^IMAGINE Institute, Necker-Enfants Malades Hospital, Assistance Publique-Hopitaux de Paris; ^8^Université de Paris, Paris, France

####### **Correspondence:** Athimalaipet Ramanan

*Pediatric Rheumatology 2024*, **22(2):**PReS24-ABS-1294

**Introduction:** Baricitinib (BARI) could target multiple cytokine pathways associated with juvenile idiopathic arthritis associated uveitis (JIA-U) and antinuclear antibody (ANA)-positive uveitis, providing a novel therapeutic approach.

**Objectives:** Evaluating the efficacy and safety of BARI on paediatrics with active JIA-U or chronic anterior ANA-positive uveitis, with an inadequate response to methotrexate (MTX) or biologic disease modifying antirheumatic drugs (bDMARDs) and on topical corticosteroid eye drops at a stable dose.

**Methods:** The open-label, active-controlled, Phase 3 trial, was conducted on children aged 2-<18 years. Patients received 2mg (2-<9 years) or 4mg (9-<18 years) oral BARI once daily. A reference patient group received adalimumab (ADA, subcutaneous injection) every 2 weeks (20/40mg based on weight). Primary efficacy endpoint: proportion of responders at Week 24 (W24), defined by Standardization of Uveitis Nomenclature criteria as a 2-step decrease in inflammation (anterior chamber cells) or decrease to zero through W24 in the eye most severely affected at baseline. A Bayesian analysis was performed for the primary endpoint, based on pre-specified success criteria (the posterior probability of the treatment response rate exceeding 57% is at least 80%).

**Results:** BARI (*N*=24) vs ADA group (*N*=5): mean age 11.6 vs 6.6 years, males 58.3% vs 100%, White 100% (data missing for 4) vs 80%, not Hispanic/Latino 52.2% vs 80%. The primary endpoint of the study was not met. BARI group: 33% patients achieved a response at W24, resulting in 1.03% posterior probability of a response rate of >57%. The treatment-emergent adverse events (TEAEs) were consistent with the established safety profile in other BARI indications in paediatrics and adults. BARI group: 83.3% patients reported with at least 1 TEAE (overall, 41.7% mild, 29.2% moderate and 12.5% severe).

**Conclusion:** The primary endpoint of the study was not met. The data provides additional information for the treatment of children with JIA uveitis refractory to both MTX and bDMARDs. Also, BARI safety profile in this study was consistent with previous studies in children and adults with other diseases, with most TEAEs being mild/moderate.

**Patient Consent**

Not applicable (there are no patient data)

**Disclosure of Interest**

None Declared

### JIA (oligo, poly, psoriatic)

#### OR21 Harnessing the window of opportunity in childhood arthritis: results from the UCAN CAN-DU study

##### Jelleke B. De Jonge^1,2^, Sytze de Roock^1^, Sebastiaan J. Vastert^1,2^, Rae S.M. Yeung^3^, Deborah Marshall^4^, Joost F. Swart^1^, Susanne M. Benseler^5,6^ and on behalf of the UCAN CAN-DU consortia

###### ^1^Department of Pediatric Rheumatology, Division of Paediatrics, University Medical Center Utrecht, Wilhelmina Children’s Hospital; ^2^Center for Translational Immunology, University Medical Center Utrecht, Utrecht University, Utrecht, Netherlands; ^3^Division of Rheumatology, Department of Paediatrics, Immunology and Institute of Medical Science, The Hospital for Sick Children, University of Toronto, Toronto, Ontaria; ^4^Department of Community Health Services, McCaig Institute for Bone an Joint Health, O’Brien Institute for Public Health, University of Calgary; ^5^Alberta Children’s Hospital Research Institute, University of Calgary; ^6^Division of Rheumatolgoy, Department of Pediatrics, Alberta Children’s Hospital, Cumming School of Medicine, University of Calgary, Calgary, Canada

####### **Correspondence:** Jelleke B. De Jonge

*Pediatric Rheumatology 2024*, **22(2):**PReS24-ABS-1750

**Introduction:** Childhood arthritis (Juvenile Idiopathic Arthritis, JIA) is the most common chronic rheumatic disease carrying a dramatic individual and societal burden. Biologic therapies can effectively target inflammatory pathways, control joint inflammation, and prevent disability. Recent studies suggested a window of opportunity, in which early precision treatment start may result in rapid and sustained remission.

**Objectives:** To determine the timing of starting biologic therapies for childhood arthritis across two countries, compare practice patterns, and analyse their impact on achieving a state of inactive disease.

**Methods:** The international UCAN CAN-DU study prospective enrols children with arthritis across Canada (CAN) and the Netherlands (DU) since 2018. This nested cohort study included consecutive, biologic-naïve non-systemic patients at start of biologics and observed for six months. Baseline characteristics including demographics, ILAR JIA subtype, active joint counts and extraarticular features were captured, as well as biologic pathway and selected biologic therapies. Time to biologic treatment was defined as time from symptom onset to the start date of the first biologic. Outcome: Inactive disease at six months defined as evidence of no active joints while maintaining in the initial biologic treatment. Analysis: descriptive statistics, comparative analyses.

**Results:** A total of 188 children from 13 centres were included; 75% were Dutch patients. Overall, these were 119 girls (63%), median age was 9.6 years (4.1,12.6). JIA subtype per country: CAN - 45% poly, 32% ERA, 22% oligo/psoriatic versus DU - 43% poly, 19% ERA, 37% oligo/psoriatic. The predominantly targeted biological pathway was TNFα in both countries (96%). Significant differences were found for generic medication versus biosimilar between countries, with higher biosimilar use in CA (p<0.01). Median time to biologics start: CAN – 93 weeks (IQR: 49-316) versus DU – 73 weeks (IQR: 30-179). Early biologics centres (only centres with >10 patients included) started at 34 weeks (IQR: 22-93) late centres at 131 weeks (IQR: 59-259). Window of opportunity: The rate of inactive disease was the highest in children receiving biologics early (<6 months, 78%) compared to intermediate (6-12 months, 63%) and late biologics starters (12-24 months, 56%).

**Conclusion:** Childhood arthritis has a window of opportunity. Early start of targeted biologic therapies controls joint inflammation in 4 out of 5 children with arthritis at six months. Potential individual and treatment associated risk factors and biologic signatures need to be determined to further advance the effectiveness of precision therapies.

**Patient Consent**

Not applicable (there are no patient data)

**Disclosure of Interest**

J. De Jonge: None Declared, S. de Roock: None Declared, S. Vastert: None Declared, R. S.M. Yeung Consultant with: Consulting fees from Novartis and Lily outside the submitted work, D. Marshall: None Declared, J. Swart: None Declared, S. Benseler: None Declared

### JIA (oligo, poly, psoriatic)

#### OR22 A multicenter study of musculoskeletal manifestations in children with inflammatory bowel diseases (Gastroreum study)

##### Federico Diomeda^1^, Manuela Taurisano^2^, Rossella Greco^3^, Silvia Greco^4^, Eleonora Prete^5^, Romina Gallizzi^6^, Alessandra Spagnolo^6^, Marco Gattorno^7^, Chiara Longo^8^, Serena Arrigo^8^, Silvia Magni Manzoni^9^, Fabrizio De Benedetti^9^, Giusyda Tarantino^9^, Davide Montin^10^, Ilaria Battagliere^10^, Gabriele Simonini^11^, Edoardo Marrani^11^, Giovanni Conti^12^, Cecilia Lugarà^12^, Emanuela Del Giudice^13^, Riccardo Lubrano^13^, Giovanni Filocamo^14^, Arianna Petrillo^14^, Rosaria Celano^14^, Fortunata Civitelli^15^, Elisabetta Cortis^15^, Francesca Ardenti Morini^15^, Angela Miniaci^16^, Serena Pastore^17^, Pierandrea Elefante^18^, Francesco La Torre^19^, Bianca Lattanzi^20^, Angela Mauro^21^, Lorenzo Mambelli^22^, Patrizia Barone^23^, Alma Nunzia Olivieri^24^, Maria Francesca Gicchino^24^, Gianluca Vergine^25^, Valeria Dell’Omo^25^, Maria Cristina Maggio^26^, Francesca Cavataio^26^, Ilenia Floretta^27^, Simona Campus^28^, Luca Cantarini^29^, Emanuela Sacco^30^, Michela Cappella^31^, Francesca Bovis^32^, Angelo Ravelli^33,34^, Adele Civino^1^ on behalf of Italian Society of Pediatric Rheumatology (ReumaPed)

###### ^1^UOSD Reumatologia e Immunologia Pediatrica, Ospedale “Vito Fazzi”; ^2^Università del Salento, Lecce; ^3^Università degli Studi di Bari “Aldo Moro”, Bari; ^4^Università degli Sudi di Chieti; ^5^AO “Cardinale Panico”, Lecce; ^6^UOC Pediatria Specialistica e Malattie Rare, Azienda Ospedaliera “Renato Dulbecco”, Catanzaro; ^7^Clinica Pediatrica e Reumatologia; ^8^Gastroenterologia pediatrica ed Endoscopia digestiva, IRCCS Istituto “G. Gaslini”, Genova; ^9^UOC Reumatologia , IRCCS Ospedale Pediatrico “Bambino Gesù”, Roma; ^10^SC Pediatria Specialistica, AOU “Città della Salute e della Scienza”, Torino; ^11^Rheumatology Unit, ERN ReCONNET center, Meyer Children’s Hospital IRCCS, Firenze; ^12^UO Nefrologia e Reumatologia Pediatrica, AOU Policlinico “G. Martino”, Messina; ^13^UOC Pediatria e Neonatologia, Ospedale “Santa Goretti”, Latina; ^14^Pediatria Immunoreumatologia, Fondazione IRCCS “Ca’ Granda” Ospedale Maggiore Policlinico, Milano; ^15^UOC Pediatria, Ospedale “Sant’Eugenio”, Roma; ^16^Pediatric Unit, IRCCS Azienda Ospedaliero-Universitaria, Bologna; ^17^SS Reumatologia, IRCCS Materno Infantile “Burlo Garofolo”; ^18^Dipartimento di Scienze Mediche, Chirurgiche e della Salute, Università degli Studi di Trieste, Trieste; ^19^UOC Pediatria Ospedaliera, Ospedale Pediatrico “Giovanni XXIII”, Bari; ^20^SOD Pediatria, AOU “Ospedali Riuniti”, Ancona; ^21^Dipartimento di Medicina dell’Infanzia e dell’Età Evolutiva, ASST “Fatebenefratelli-Sacco”, Milano; ^22^UOC Pediatria, Ospedale “Santa Maria delle Croci”, Ravenna; ^23^UOSD Pediatria ad indirizzo reumatologico, AOU Policlinico San Marco, Catania; ^24^AOU dell’Università degli studi della Campania “Luigi Vanvitelli”, Napoli; ^25^UOC Pediatria, Ospedale “Infermi”, Rimini; ^26^Clinica Pediatrica, Ospedale dei Bambini “G. Di Cristina”, Palermo; ^27^UO Pediatria, APSS di Trento, Trento; ^28^SC Clinica Pediatrica e Malattie Rare, Ospedale Pediatrico Microcitemico “A. Cao”, Cagliari; ^29^UOC Reumatologia, Policlinico “Santa Maria alle Scotte”, Siena; ^30^UOC Pediatria, Ospedale “Casa Sollievo della Sofferenza”, San Giovanni Rotondo; ^31^UOSD Reumatologia Pediatrica e dell’Adolescenza, Arcispedale “Santa Maria Nuova”, Reggio Emilia; ^32^Department of Health Sciences (DISSAL), Università di Genova; ^33^Direzione Scientifica, IRCCS Istituto “Giannina Gaslini”; ^34^Dipartimento di Neuroscienze, Riabilitazione, Oftalmologia, Genetica e Scienze Materno Infantili (DINOGMI), Università di Genova, Genova, Italy

####### **Correspondence:** Federico Diomeda

*Pediatric Rheumatology 2024*, **22(2):**PReS24-ABS-1455

**Introduction:** Musculoskeletal (MSK) manifestations are frequent in children with inflammatory bowel diseases (IBD) and may be the presenting clinical feature. However, the frequency and characteristics of MSK symptoms in pediatric-onset IBD (p-IBD) have been seldom investigated.

**Objectives:** To describe the clinical features and outcomes of children with IBD-associated MSK manifestations.

**Methods:** This study retrospectively evaluated the characteristics of p-IBD patients who presented with MSK complaints at 25 Italian pediatric rheumatology centers between 2010 and 2022. A chi-square test was used to assess the association between clinical features and the presence of active arthritis.

**Results:** 180 patients with p-IBD and MSK symptoms were enrolled: 56.7% were males and 61.7% had Crohn’s disease (CD), 30.5% ulcerative colitis (UC), and 7.8% unclassified-IBD (u-IBD). The median age at IBD diagnosis was 12 years (6.6-17.5) and 11 patients (6.1%) had early onset-IBD (i.e.< 6 years). In about half of patients MSK symptoms had their onset before IBD diagnosis, with a median time lag of 12.5 months (1.2-41.7). In more than 80% of these patients, at least one sign suggesting IBD (diarrhea, abdominal pain, weight loss, growth failure, recurrent oral aphthosis, anemia, hypoalbuminemia, positive family history of IBD) was present at onset of MSK symptoms. Of the 180 patients, 131 (72.8%) had arthralgia,125 (69.4%) arthritis, 46 (25.6%) inflammatory back pain, 20 (11.1%) enthesitis, and 10 (5.6%) dactylitis. Of the 125 patients with arthritis, 77% had peripheral arthritis, 14.4% axial involvement, defined as radiographic sacroiliitis, and 9% combined axial and peripheral arthritis. Peripheral joint disease was oligoarticular in 76% of patients, with the most frequently involved joints being the knee (50.5%), ankle (36.4%), and hip (19.6%). Other extra-intestinal manifestations included recurrent oral aphthosis (11.1%), erythema nodosum (6.6%), chronic non-bacterial osteomyelitis (5.6%), psoriasis (5%), uveitis (3.9%), sclerosing cholangitis (3.4%), autoimmune hepatitis (1.7%), and pyoderma gangrenosum (1%). At 1-year follow-up, 77.3% of patients who presented with arthritis had remission of joint disease, 19.7% had active IBD without active arthritis, and 6.6% had both active arthritis and active IBD. Persistence of arthritis activity appeared to be associated with the presence of psoriasis (*p*=0.042) and ANA positivity (*p*=0.043).

**Conclusion:** Most of our patients with p-IBD and MSK manifestations presented with peripheral oligoarthritis involving knee and ankle joints. In about half of the patients, the diagnosis of IBD was made long after onset of MSK manifestations, despite the presence of symptoms suggesting IBD at the time of occurrence of MSK complaints. Arthritis had a benign course in most patients as only a minority had persistently active arthritis at 1-year follow-up visit.

**Patient Consent**

Yes, I received consent

**Disclosure of Interest**

None Declared

### JIA (oligo, poly, psoriatic)

#### OR23 Serum biomarkers associated with baricitinib response in JIA patients

##### Venkatesh Krishnan^1^, Stuart Y. Keller^1^, A.V. Ramanan^2^, Christine Chew^2^, Rona Wang^1^, Jonathan Sims^1^, ChingYun Chang^1^, Ernst R. Dow^1^

###### ^1^Eli Lilly and Company, Indianapolis, United States; ^2^Bristol Pediatric Rheumatology, Bristol, United Kingdom

####### **Correspondence:** A.V. Ramanan

*Pediatric Rheumatology 2024*, **22(2):**PReS24-ABS-1096

**Introduction:** In the phase 3, randomized, double-blind, placebo-controlled, withdrawal, global efficacy, and safety trial, JUVE-BASIS, baricitinib (BARI) treatment resulted in robust improvement in clinical response (Juvenile Arthritis Disease Activity Score 27 joints [JADAS-27]) in patients with juvenile idiopathic arthritis (JIA) after a 12-week open-label lead-in period, which was sustained over the randomized assignment period.^1^

**Objectives:** Enrolled patients (aged 2 to <18 years) with polyarticular JIA (positive or negative for rheumatoid factor), extended oligoarticular JIA, or enthesitis-related arthritis were investigated for longitudinal serum biomarker changes between Week 0 (baseline) and Week 12.

**Methods:** A total of 168 serum samples were analyzed (84 patients) using the Olink Explore 3072 panel at baseline and Week 12. All patients in these analyses provided informed consent for this exploratory biomarker study. BARI-mediated pharmacodynamic changes in serum protein markers were measured as changes from baseline to Week 12 derived from a mixed model with repeated measurement, with cutoffs for exploratory purpose at 1.3-fold change and at 0.1 of p-value for Week 12 vs baseline. Pearson correlation of the change in serum biomarkers and JADAS-27 scores comparing baseline to Week 12 were reported, with a cutoff at 0.2 correlation. The proportional change from baseline for serum markers were also categorically identified across 3 response sub-sets of JIA patients: Juvenile Idiopathic Arthritis-American College of Rheumatology (JIA-ACR) <30% (non-responders), JIA-ACR 30%–70% (responders), and JIA-ACR 70%–100% (super-responders).

**Results:** The serum biomarkers IL-6 (fold-change −1.81*), MMP-3 (−1.58*), FGL1 (−1.43*), CCL7 (−1.35*), and CCL18 (−1.31*) were significantly lower than baseline and MYOC (1.41*) and EPO (1.71*) were significantly higher than baseline following 12 weeks of treatment with BARI for all patients, and these changes were correlated with the change in JADAS-27 score. For the subset of patients with JIA-ACR <30%, no significant differences in these serum biomarkers were observed between baseline and Week 12. For patients with JIA-ACR 30%–70% (responders), significant reductions were seen in MMP-3 (fold-change −1.65), FGL1 (−1.44), and CCL18 (−1.33*) and significant increase in EPO (1.83*). For the subset of patients with JIA-ACR >70% (super-responders), all biomarkers showed significant change from baseline (IL-6 fold-change −2.26*; MMP-3 −1.80*; FGL1 −1.47*; CCL7 −1.52*; CCL18 −1.35*; MYOC 1.40*; and EPO 1.61*). The biomarkers that correlate with clinical response suggest a pronounced role for macrophage activation (CCL7, CCL18, IL-6) and matrix regulation (MMP-3).

* adjusted p-value<0.05

**Conclusion:** This is the first study measuring serum protein markers in the context of an intervention trial with BARI in JIA patients. Notably, the association of such biomarker changes with clinical response and the categorical analyses of JIA-ACR response may allow a physician to measure such markers in patients to help reaffirm the clinical utility of BARI in JIA patients.

**Trial registration identifying number:** ClinicalTrials.gov, NCT03773978

**Patient Consent**

Not applicable (there are no patient data)

**Disclosure of Interest**

V. Krishnan Shareholder with: Eli Lilly and Company, Employee with: Eli Lilly and Company, S. Keller Shareholder with: Eli Lilly and Company, Employee with: Eli Lilly and Company, A. Ramanan Grant / Research Support with: Eli Lilly and Company, Consultant with: Eli Lilly and Company, AbbVie, GlaxoSmithKline, Novartis, Pfizer, Roche, Sobi, and UCB, C. Chew: None Declared, R. Wang Shareholder with: Eli Lilly and Company, Employee with: Eli Lilly and Company, J. Sims Shareholder with: Eli Lilly and Company, Employee with: Eli Lilly and Company, C. Chang Shareholder with: Eli Lilly and Company, Employee with: Eli Lilly and Company, E. Dow Shareholder with: Eli Lilly and Company, Employee with: Eli Lilly and Company

**Reference**

1. Ramanan AV, Quartier P, Okamoto N, et al. Baricitinib in juvenile idiopathic arthritis: An international, phase 3, randomised, double-blind, placebo-controlled, withdrawal, efficacy, and safety trial. Lancet. 2023;402:555-70.

### Juvenile dermatomyositis

#### OR24 Exploring the interferon signature as a biomarker for disease activity and organ damage in juvenile dermatomyositis

##### Helena Codes-Méndez^1^, Senne Cuyx^2^, Afroditi Barmpakou^2^, Elena Moraitis^2^, Sandrine Compeyrot-Lacassagne^2^, Muthana Al-Obaidi^2^, Charalampia Papadopoulou^2^

###### ^1^Rheumatology, Hospital de la Santa Creu i Sant Pau, Barcelona, Spain; ^2^Paediatric Rheumatology, Great Ormond Street Hospital for Children NHS Foundation Trust, London, United Kingdom

####### **Correspondence:** Helena Codes-Méndez

*Pediatric Rheumatology 2024*, **22(2):**PReS24-ABS-1311

**Introduction:** Juvenile dermatomyositis (JDM) is a systemic autoimmune disease with a prominent up-regulation of interferon (IFN) signaling system. To date, no validated markers for assessing disease activity have been identified.

**Objectives:** To analyze the IFN signature in patients with JDM and evaluate its potential as biomarkers for disease activity and organ damage, including manifestations in the skin, muscle, joints, lung, and gastrointestinal (GI) tract.

**Methods:** Retrospective study on a cohort of 69 JDM patients from the largest paediatric rheumatology department in the UK between 03/2020 and 05/2024. IFN type I/II pathways were assessed by RT-PCR quantitation^1^. Disease activity assessment included the evaluation of muscle strength (CMAS/MMT8), skin (modified DAS), and the physician’s global assessment. Statistical analysis included Mann-Whitney tests and Spearman’s correlations to explore associations between disease activity, organ involvement, and expression levels of each IFN gene. Statistical significance was set at *p*<0.05.

**Results:** A total of 150 blood samples were obtained from 69 patients (48 females), with a median age at diagnosis of 7(1-13) years. All presented muscle and skin involvement at disease onset. GI involvement (dysphagia) was observed in 18 patients, while 13 presented interstitial lung disease (ILD). Longitudinal follow-up data were available for 41 patients.

The most prevalent myositis-specific autoantibodies were TIFγ(18.3%), NXP2(12.6%), MDA5(12.6%), and Mi2(8.4%).

Patients with active muscle involvement presented higher expressions of IFI27 (p<0.001), IFI44L (p<0.001), IFIT1 (p<0.001), IFNB1 (*p*=0.02), RSAD2 (p<0.001), SIGLEC1 (p<0.001), CXCL10 (*p*=0.04), and IL-18 (*p*=0.004).

Arthritis was associated with elevated IFI27 (*p*=0.004), IFI44L (*p*=0.01), SIGLEC1 (*p*=0.03), and CXCL9 (*p*=0.04).

Patients with active skin disease presented higher expressions of IFI27 (p<0.001), IFI44L (p<0.001), IFIT1 (p<0.001), RSAD2 (p<0.001), SIGLEC1 (p<0.001), and CXCL10 (*p*=0.02). Calcinosis reached statistical significance for IFIT1 (*p*=0.03), RSAD2 (*p*=0.02) and IL-18 (*p*=0.02); whereas patients with V-sign, mucous membrane lesions and subcutaneous oedema had higher expressions of CXCL9 (*p*=0.005, 0.03, 0.03; respectively). No significant association was found with skin ulcerations.

Patients with ILD presented higher expressions of IFI27 (*p*=0.04) and CXCL9 (*p*=0.01), while those with dysphagia had elevated IFI27 (*p*=0.02), IFIT1 (*p*=0.02), SIGLEC1 (*p*=0.01).

Baricitinib was initiated in 11 patients due to refractory disease activity. Among them, 4 experienced normalization of IFN levels and achieved clinical remission. The remaining patients have shown promising initial responses, though it is premature to fully evaluate outcomes due to the recent treatment initiation.

**Conclusion:** Our study demonstrates hyperactivation of the IFN signaling systems in JDM patients. Both IFN I/II regulated transcripts were associated with muscle and skin disease activity. Our findings indicate that calcinosis is associated with RSAD2 and IFIT1, as well as IL-18, whereas skin ulceration did not show any association. Notably, ILD was associated with IFI27 and CXCL9. Patients with dysphagia exclusively exhibited hyperactivation of IFN-I signaling system.

The serum IFN gene signature shows promise as a biomarker in JDM. Further studies are needed to confirm these results.

**Patient Consent**

Yes, I received consent

**Disclosure of Interest**

None Declared

**Reference**

1. Papadopoulou C, et al. Janus kinase 1/2 inhibition with baricitinib in the treatment of JDM. Brain. 2019 Mar.

### Psycho-social aspects and rehabilitation

#### OR25 “I have never spoken to anyone about this before”: giving fathers an opportunity to share their experiences

##### Klaudia Kupiec^1^, Elizabeth Bichard^1^, Polly Livermore^2^

###### ^1^Great Ormond Street Hospital for Children NHS Foundation Trust; ^2^Great Ormond Street Hospital for Children NHS Foundation Trust BRC, London, United Kingdom

####### **Correspondence:** Klaudia Kupiec

*Pediatric Rheumatology 2024*, **22(2):**PReS24-ABS-1648

**Introduction:** Fathers of children and young people with Juvenile Idiopathic Arthritis have been shown to experience different thoughts and emotions compared to mothers. These different perspectives are important when considering the completion of patient reported outcomes in clinical appointments and as part of research protocols. However, our work has also shown that providing an opportunity for fathers to share their perspective of raising a child or young person with a rheumatological condition is cathartic and provides a release for fathers to talk openly without worry about sharing how they feel. Health Care Professionals can consider these differences and aide parents with strategies to strengthen their partnership in their child’s healthcare. This is the first known piece of work to involve fathers of a range of paediatric rheumatological conditions.

**Objectives:** The IMPACT study is a large four phased UK wide study to design, develop and test a technology intervention to support parents of children and young people with rheumatological conditions. The study follows Experience Based Co-Design Methodology where the patients and their families help shape the study from design through to dissemination. Understanding their viewpoints is critical to ensure the technology we create is useful and usable by parents.

**Methods:** As part of the first phase of this study we invited children, young people, young adults, parents and healthcare professionals to share with us their experiences and perspectives in focus groups. We wanted to understand their particular barriers and facilitators to good care for all paediatric rheumatological diseases, at all ages. The child and young person groups talked through a vignette to save each child needing to feel they needed to share their own experiences. The other groups had minimal structure and were led by both the research team and in most instances a member of the wide Steering Group, made up of children, young people, parents and health care professionals. Reflexive thematic analysis by Braun and Clark was used to code the data.

**Results:** We conducted 24 focus groups with 158 participants between 19^th^ Dec 2023 and 18^th^ April 2024. By chance some of the parent groups ended up with just mother participation, we therefore purposefully encouraged the participation of fathers to form a father only group. We ended up running two of these groups with a total of 14 fathers involved and included a range of rheumatological conditions (JIA = 6, JDM=3, CRMO=1, PFAPA=1). Themes arising from the fathers included: Wanting control, Being the bad parent, Their role in the healthcare system, Communication difficulties and Loneliness.

**Conclusion:** Fathers felt that they often have a different role to play to the child’s mother and that when this works well, the roles can contemplate each other. However, fathers also felt that their responsibilities were often challenging, such as for example when they are expected to cause weekly pain through injection administration as the other parent wants to be the comforter. They also talked about a loss of control from not knowing as much as their partner and the worry that this could have on their child’s care for example by recalling incorrect medications – some described this as feeling this was a ‘test’. This work highlights the need to consider the fathers perspectives and provide strategies for families to consider how to improve communication and work together for the best interests of their child. Fathers also commented that they had never shared their reflections with anyone before and how beneficial they found sharing this within the safety of the fathers group.

**Patient Consent**

Yes, I received consent

**Disclosure of Interest**

None Declared

### Pain, fatigue, disease experience and quality of life

#### OR26 Empowering pediatric rheumatic youngsters: a journey through pain awareness insights from an online webinar

##### Asena Yekdaneh^1,2^, Nilay Arman^3^, Yusuf Açıkgöz^4^, Figen Çakmak^5^, Nuray Aktay Ayaz^6^

###### ^1^Vocational School of Health Services Physiotherapy English Program, Fenerbahce University; ^2^Institute of Graduate Studies Physiotherapy and Rehabilitation Doctorate Program; ^3^Faculty of Health Sciences Department of Physiotherapy and Rehabilitation; ^4^Institute of Graduate Studies Physiotherapy and Rehabilitation Master of Science Program, Istanbul University-Cerrahpaşa; ^5^Pediatric Rheumatology, Başakşehir Çam and Sakura City Hospital; ^6^Istanbul Faculty of Medicine, Department of Pediatric Rheumatology, Istanbul University, Istanbul, Türkiye

####### **Correspondence:** Asena Yekdaneh

*Pediatric Rheumatology 2024*, **22(2):**PReS24-ABS-1493

**Introduction:** Pediatric rheumatic diseases (PRDs) encompass conditions with unpredictable prognoses, leading to impairments and negatively impacting functioning and quality of life (QoL) (1). Chronic pain, common in PRDs, can significantly limit physical activity and QoL. Biopsychosocial models in pediatrics recognize that chronic pain perception and associated limitations are influenced by physiological and psychological processes (2). Comprehensive assessments are emphasized to identify factors affecting pain, fear of pain, participation, and to provide chronic pain awareness education for children and adolescents (3). Despite the recognized importance of pain awareness, self-management, and education in adults and PRDs, there’s a lack of interactive educational programs specifically targeting pain awareness in PRDs.

**Objectives:** The aim of this study was to question the effects of a webinar on pain in patients with PRD on pain awareness.

**Methods:** Twenty-two children and adolescents aged 12-18 years with PRDs were included, consisting of 18 girls and 4 boys. Among them, 4 had Familial Mediterranean Fever, 17 had Juvenile Idiopathic Arthritis, and 1 had Juvenile Fibromyalgia. An interactive webinar via Zoom, comprising a single 30-minute session spread over 2 days within a week, was organized based on participants’ preferred timings. Pain awareness levels were assessed using Google Forms with 11 questions before and after the webinar. The webinar provided informative presentations on pain awareness and coping methods. Following the webinar, the same questions were presented as a game-competition using the “Quizizz platform”. Participants were also verbally asked about their satisfaction levels during and after the webinar.

**Results:** The mean age of participants with PRDs were 13.8 years. Before the webinar, 21 of them perceived pain as a negative sensation, and 8 of them believed they couldn’t cope with pain in the long term. After the webinar, these numbers changed to 14 and 5, respectively. Additionally, the number of participants preferring physical activity as a coping method for pain increased from 14 to 21. Awareness of stress levels affecting pain increased from 16 participant to 21. Furthermore, 18 participants expressed a desire for the educational webinar to be organized again. Lastly, while only 17 participants considered pain to be a shareable emotion before the webinar, all children held this view after the webinar.

**Conclusion:** The preliminary findings of this study indicate that a single educational webinar session can influence pain awareness levels in PRDs. After the webinar, children and adolescents show an enhanced understanding of managing their chronic pain and acquiring coping mechanisms. Moreover, we propose that interactive webinars designed for this purpose can effectively address barriers to participation and provide motivational reinforcement for PRD patients. Encouraged by the positive reception from the participants, we advocate for the integration of physiotherapists and other healthcare professionals working with this patient group in comprehensive educational programs aimed at mitigating the long-term symptoms of PRDs.

**Date of birth:** 29.12.1994

**Patient Consent**

Yes, I received consent

**Disclosure of Interest**

None Declared

**References**

1. Frye, W.S.,&Milojevic,D. (2022). The Role of Psychology in Pediatric Rheumatic Diseases. Pediatric clinics of North America, 69(5), 965–974.
2. Cunningham, N.R.,&Kashikar-Zuck, S. (2013). Nonpharmacological treatment of pain in rheumatic diseases and other musculoskeletal pain conditions. Current rheumatology reports, 15(2), 306.
3. Asmundson GJG, Noel M, Petter M, et al. (2012). Pediatric fear-avoidance model of chronic pain: Foundation, application and future directions. Pain Research and Management 17: 397–405.

### Macrophage activation syndrome

#### PT01 Current treatment of macrophage activation syndrome worldwide: the metaphor project, a PReS/PRINTO real-life international survey

##### Francesca Minoia^1^, Francesco Baldo^1,2^, Remco G. A. Erkens^3^, Claudia Bracaglia^4^, Dirk Foell^5^, Marco Gattorno^6^, Marija Jelusic^7^, Jordi Anton^8^, Paul Brogan^9^, Scott Canna^10^, Shanmuganathan Chandrakasan^11^, Randy Q. Cron^12^, Fabrizio De Benedetti^4^, Alexei Grom^13^, Merav Heshin-Bekenstein ^14^, AnnaCarin Horne^15^, Raju Khubchandani ^16^, Mao Mizuta^17^, Seza Ozen^18^, Pierre Quartier^19^, Angelo Ravelli^6^, Masaki Shimizu^20^, Grant Schulert^13^, Christiaan Scott^21^, Nicola Ruperto^6^, Rashmi Sinha^22^, Joost Swart^3^, Sebastiaan Vastert^3^ and on behalf of the PReS MAS/sJIA Working Party and Pediatric Rheumatology International Trial Organization

###### ^1^Fondazione IRCCS Ca’ Granda Ospedale Maggiore Policlinico; ^2^ASST Gaetano Pini, Milan, Italy; ^3^University Medical Center , Utrecht, Netherlands; ^4^IRCCS Ospedale Pediatrico Bambino Gesù, Rome, Italy; ^5^University Hospital Muenster, Muenster, Germany; ^6^IRCCS Istituto Giannina Gaslini, Genoa, Italy; ^7^University Hospital Centre , Zagreb, Croatia; ^8^Hospital Sant Joan de Déu, Barcelona, Spain; ^9^Great Ormond Street Hospital for Children, London, United Kingdom; ^10^Children’s Hospital of Philadelphia, Philadelphia; ^11^Aflac Cancer and Blood Disorders Center Children’s Healthcare , Atlanta; ^12^University of Alabama at Birmingham, Birmingham; ^13^Cincinnati Children’s Hospital, Cincinnati, United States; ^14^Dana Dwek Children’s Hospital, Tel Aviv, Israel; ^15^Karolinska University Hospital, Stockholm, Sweden; ^16^SRCC Childrens Hospital, Mumbai, India; ^17^Hyogo Prefectural Kobe Children’s Hospital, Kobe, Japan; ^18^Department of Pediatric Rheumatology, Hacettepe University, Ankara, Türkiye; ^19^Université Paris-Cité, Paris, France; ^20^Graduate School of Medical and Dental Sciences, Tokio, Japan; ^21^University of Ottawa, Ottawa, Canada; ^22^Systemic JIA Foundation, Cincinnati, United States

####### **Correspondence:** Francesca Minoia

*Pediatric Rheumatology 2024*, **22(2):**PReS24-ABS-1597

**Introduction:** Despite significant improvement in its management, macrophage activation syndrome (MAS) treatment is still not standardized, due to lack of robust evidence and differences in access to medications. This holds especially true for MAS related to rheumatologic conditions other than systemic juvenile idiopathic arthritis (sJIA)

**Objectives:** To capture the current therapeutic approaches to MAS worldwide and to evaluate major unmet needs and treatment challenges.

**Methods:** In context of the METAPHOR project, a PReS/PRINTO initiative to optimize treatment in sJIA and MAS, a survey on MAS treatment was developed. Topics addressed were based on a systematic literature review, and selected by a panel of 22 experts, including 1 patient representative and 2 pediatric hematologists. The survey consisted of: a) demographic section, including country of practice; b) clinical section, investigating real-life approaches to MAS; c) patient focused section, developed with patient representatives and exploring major unmet needs. Physicians part of the PReS/PRINTO network and of the Histiocyte Society were invited to anonymously complete the web-survey from Oct 5^th^ to Dec 15^th^2023

**Results:** A total of 287 replies from 64 countries worldwide were collected. Respondents were mostly pediatric rheumatologists (90%), while 10% were pediatric hematologists. Methylprednisolone (MPN) pulses were the commonest glucocorticoid (GC) in all subtypes of MAS. In addition to GC, ciclosporin (CsA) and anakinra were the cornerstones of treatment in sJIA-MAS, used by 91% and 74% of physicians, respectively. Anakinra was the most selected agent beside GC as 1^st^ line, and only 2% of respondents indicated not to consider the use anakinra in sJIA-MAS. However, access to anakinra is a major gap across countries: almost all physicians would use it in North America and Western Europe (1^st^ line for 66 and 50% of respondents, respectively) but it is still unavailable for more than 65% and 70% of physicians in Asia and South America. Systemic erythematosus lupus (SLE)-associated MAS represents the condition with the highest heterogeneity of therapeutic approaches across centres and countries. Besides MPN, which was largely the 1^st^ line choice worldwide (98%), 58% of physicians would use anakinra as 1^st^ line in North America and 63% as 1^st^/2^nd^ line in Western Europe, while CsA and immunoglobulin would be the medications of choice in all the other countries. Etoposide was globally the agent most frequently selected as 3^rd^ line/rescue, followed by JAK-inhibitors, mainly ruxolitinib. Emapalumab was potentially chosen as 2^nd^/3^rd^ line treatment across all subtypes of MAS; however, its access is still drastically limited in all countries, except North America and partially Western Europe

**Conclusion:** A wide heterogeneity in the approach to MAS still exists, with relevant discrepancies in its management worldwide. An international effort is needed to optimize therapeutic options, reduce gaps in access to medications and harmonize treatment

**Patient Consent**

Not applicable (there are no patient data)

**Disclosure of Interest**

F. Minoia Consultant with: SOBI, Novartis, F. Baldo: None Declared, R. Erkens: None Declared, C. Bracaglia Consultant with: SOBI, Novartis, Speaker Bureau with: GSK, D. Foell Consultant with: Novartis, SOBI and Boehringer., M. Gattorno: None Declared, M. Jelusic: None Declared, J. Anton: None Declared, P. Brogan: None Declared, S. Canna: None Declared, S. Chandrakasan: None Declared, R. Cron Consultant with: Abbvie, Pfizer and SOBI, F. De Benedetti: None Declared, A. Grom Grant / Research Support with: Novartis, SOBI and Kiniksa, M. Heshin-Bekenstein : None Declared, A. Horne Speaker Bureau with: SOBI, Novartis, R. Khubchandani : None Declared, M. Mizuta: None Declared, S. Ozen Consultant with: SOBI, Novartis, P. Quartier: None Declared, A. Ravelli: None Declared, M. Shimizu: None Declared, G. Schulert Grant / Research Support with: IpiNovyx, Consultant with: SOBI and Boehringer Ingelheim, C. Scott: None Declared, N. Ruperto: None Declared, R. Sinha: None Declared, J. Swart: None Declared, S. Vastert: None Declared

### Macrophage activation syndrome

#### PT02 Dynamics of neutrophil activation in the TLR-9-induced mouse model of macrophage activation syndrome

##### Natsumi Inoue^1^, Kairavee Thakkar^2,3^, Richard Chhaing^1^, Thuy Do^1^, Nathan Salomonis^2,4^, Grant Schulert^1,4^

###### ^1^Division of Rheumatology; ^2^Division of Biomedical Informatics, Cincinnati Children’s Hospital Medical Center; ^3^Department of Pharmacology and Systems Physiology; ^4^Department of Pediatrics, University of Cincinnati College of Medicine, Cincinnati, United States

####### **Correspondence:** Natsumi Inoue

*Pediatric Rheumatology 2024*, **22(2):**PReS24-ABS-1074

**Introduction:** Macrophage activation syndrome (MAS) is a potentially life-threatening hyperinflammatory cytokine storm syndrome that can complicate rheumatic diseases, including systemic juvenile idiopathic arthritis (SJIA). While neutrophilia and neutrophil activation is a key feature of SJIA, MAS is associated with neutropenia. However, the functional contribution and the dynamics of neutrophils in MAS are largely unexplored.

**Objectives:** The objective of this study is to define the features of neutrophil activation in the repeated TLR9-induced model of experimental MAS.

**Methods:** Wild type C57BL/6 mice six to ten weeks of age were intraperitoneally injected with 50 μg of CpG ODN1826 five times in 10 days. Mice were sacrificed one day after the last injection (acute MAS) or after 21 days (resolution). Neutrophils were isolated from whole blood using magnetic negative selection for single-cell RNA sequencing (scRNA-seq) and cytokine release assays. For scRNA-seq, isolated neutrophils were processed with 10X Genomics and Illumina Novaseq platform. Following sequencing, libraries were processed in Cell Ranger followed by downstream integrated analysis in Seurat 3 to determine cell types across 3 conditions. Differential expression and gene regulatory prediction analyses were performed using the software cellHarmony (adjp<0.05 and fold change >1.2). Cytokines were determined from culture supernatant after 20 hours of incubation with or without LPS stimulation (100ng/ml) by Milliplex Multiplex kits (MilliporeSigma).

**Results:** During experimental MAS, pancytopenia was observed, which largely normalized at resolution, except for neutropenia. Total peripheral neutrophil counts trended lower during acute MAS and were significantly lower at resolution compared with control (697/μl vs 963/μl, *p*=0.02). Immature neutrophils (CXCR2+CD101-) were markedly and significantly increased during acute MAS (14.3 % vs control 1.4 %, *p*=0.03).

To define their cellular diversity, we performed scRNA-seq of isolated peripheral blood neutrophils from control mice, during acute MAS, and resolution. After filtering contaminants, we analyzed 10,480 cells from controls, 8,278 cells from acute MAS, and 11,001 cells from resolution mice. We identified six distinct neutrophil cell populations, representing both immature and mature neutrophils. The mature cells included aged populations and populations enriched with interferon-stimulated genes (ISG). In acute MAS, we observed increased proportions of immature neutrophils (9.6 % vs 4.5 % in control) and ISG high neutrophils (15.5 % vs 11.6 % in control). Compared to control neutrophils, genes associated with interferon alpha and/or beta signaling were significantly upregulated in most populations (adjp <0.0001). Notably, *CD274* (*PD-L1*) was upregulated in MAS and upregulated PD-L1 expression on neutrophils was confirmed with flow cytometry, suggesting neutrophil exhaustion and a potential suppressive effect on T cell activation. IL-1 related genes including *IL1b* were downregulated throughout acute MAS to resolution.

Functionally, purified peripheral blood neutrophils demonstrated no significant differences in baseline secretion of inflammatory cytokine/chemokines. However, upon LPS stimulation, both IL-6 and KC release were significantly suppressed in neutrophils from both acute MAS and resolution to control.

**Conclusion:** In the repeated TLR-9-induced model of MAS, we observed neutropenia with increased immature populations, IFN driven activation with upregulated *PD-L1* during MAS, and prolonged downregulation of *IL1b* transcription and suppressed release of inflammatory mediators. Together, these results suggest that neutrophils may have dual roles as both pro-inflammatory and persistent suppressive functions following MAS. Functional assessment of neutrophils is required to clarify neutrophil contributions to MAS pathophysiology.

**Patient Consent**

Not applicable (there are no patient data)

**Disclosure of Interest**

None Declared

### Systemic JIA

#### PT03 Efficacy of MAS825, a bispecific IL-1 and IL-18 neutralizing antibody, in refractory still’s disease with recurrent macrophage activation syndrome and lung involvement

##### Andrea Ippoliti, Manuela Pardeo, Arianna De Matteis, Matteo Trevisan, Arturo Maria Greco, Giusi Prencipe, Fabrizio De Benedetti, Claudia Bracaglia

###### Ospedale Pediatrico Bambino Gesù, Roma, Italy

####### **Correspondence:** Andrea Ippoliti

*Pediatric Rheumatology 2024*, **22(2):**PReS24-ABS-1397

**Introduction:** Still’s disease (SD) represents a clinical challenge due to the multifaceted systemic inflammation and to the predisposition to severe complications including macrophage activation syndrome (MAS) and lung disease (LD). Patients with refractory SD frequently require long-term immunosuppressive treatment with potential side effects. MAS825, a bispecific monoclonal antibody targeting IL-1b and IL-18, is a potential therapeutic option for refractory SD, particularly with recurrent MAS and LD, as these patients have very high levels of IL-18.

**Objectives:** To describe 4 patients with refractory SD complicated by MAS and LD treated with MAS825 in the context of a compassionate use program.

**Methods:** IL-18 levels were measured by ELISA.

**Results:** 3 out of 4 patients were female, the mean age at disease onset was 2,4 yrs (range 0,5-6,4 yrs) and at the start of MAS825 was 5 yrs (range 1,9-8,7 yrs). The mean follow-up was 14,5 months (range 9,2-23,1 months). MAS825 was administered intravenously at the dose of 10 mg/kg every 2 weeks in addition to glucocorticoid (GC) at the dose ranging from 0.3-1 mg/kg/day in 3 patients, to mycophenolate mofetil (MMF) in 3 and cyclosporine-A (CYA) in 1. All four patients showed a good response to MAS825 with rapid improvement of clinical systemic features and normalization of inflammatory parameters. IL-18 levels at baseline (10965, 37500,199015, 60568 pg/ml respectively), decreased after 6 months (2483, 23858, 11639, 26516 pg/ml respectively) and normalized in two patients after 7 months (< 4800 pg/ml). GC treatment was tapered in 2 patients to 0,2 mg/kg/day and was stopped in 1 patient after 3 months. MMF and CYA were maintained during MAS825 therapy.

None of the patients experienced further MAS episodes. All the patients presented interstitial lung disease (ILD) diagnosed by high resolution chest computed tomography (HRCT) and characterized by septal thickening in all of them, peri-bronchovascular thickening in 2, ground glass opacities in 2 and peripheral consolidations in 1. HRCT, repeated after 6 months, showed a significant improvement of ILD, with reduction of the consolidation areas, and of the fibrotic changes.

None of the patients experienced adverse events during treatment with MAS825. One patient discontinued MAS825 after 1 year of treatment for inadequate control of joint symptoms that required repetitive intra-articular glucocorticoid injections. Despite she experience optimal control of systemic symptoms, ILD and no MAS recurrences.

**Conclusion:** Our case series suggest that simultaneous neutralization of IL-1β and IL-18 may represent a promising therapeutic approach in refractory SD with recurrent MAS and ILD.

**Patient Consent**

Yes, I received consent

**Disclosure of Interest**

None Declared

### Systemic JIA

#### PT04 Clinical and proteomic predictors of IL-1 inhibition response in patients with systemic juvenile idiopathic arthritis

##### Emma Longoni^1^, Selma C. Sheffler-Mendoza^1^, Federica Penco^1^, Enrico Drago^1^, Martina Bartolucci^2^, Silvia Rosina^1^, Clara Malattia^1^, Alessandro Consolaro^1^, Stefania Viola^1^, Riccardo Papa^1^, Caterina Matucci-Cerinic^1^, Valentina Natoli^1^, Andrea Petretto^2^, Angelo Ravelli^2^, Roberta Caorsi^1^, Marco Gattorno^1^

###### ^1^Unit of Rheumatology and Autoinflammatory diseases; ^2^Core Facilities and Scientific Direction, IRCCS G. Gaslini, Genova, Italy

####### **Correspondence:** Emma Longoni

*Pediatric Rheumatology 2024*, **22(2):**PReS24-ABS-1360

**Introduction:** Systemic Juvenile Idiopathic Arthritis (sJIA) is a complex disease where interleukin-1 (IL-1) plays a pathogenetic key-role, which led to the use of IL-1 inhibitors. Although many patients have dramatic response, not all patients were responsive to IL-1 inhibition.

**Objectives:** To characterize clinical and biological predictors of the effectiveness of IL-1 inhibitors, to identify patients with sJIA who are more likely to respond to IL-1 blockade.

**Methods:** We conducted a retrospective review of 84 consecutive patients with sJIA followed at “*G. Gaslini Institute*” treated with IL-1 inhibitors between 2004 and 2023. Response to IL-1 inhibitors was evaluated at 12 months and at the last follow-up visit and defined as clinically inactive disease (CID) without steroid treatment (complete response), maintenance of anti-IL-1 treatment without achievement of CID (partial response), withdrawal of anti-IL-1 treatment for lack of response (no-response). Plasma samples were analysed at the time of anti-IL-1 treatment by liquid chromatography tandem mass spectrometry using an Exploris 480 mass spectrometer coupled to the Evosep One chromatographic system in 30 patients (9 in CID, 10 partial responders, 11 non-responders).

**Results:** 84 patients with sJIA were followed at least for 12 months after IL-1 inhibitors were started: 81 patients received anakinra, 3 canakinumab, with a different combination of other treatments. At 12 months follow-up 36/84 (42.9%) patients had complete response to anti-IL-1 therapy and were in CID, 29/84 (34.5%) had partial response, and 19/84 (22.6%) withdrawn IL-1 inhibitors because non-responders. Three patients were in CID off therapy. The comparison between these three groups showed significant differences in disease duration before starting anti-IL1 therapy, proportion of patients with arthritis, and number of affected joints at baseline. No differences were found in baseline therapy between the three groups in terms of glucocorticoids or nonbiologic disease modifying antirheumatic drugs (DMARDs). The median follow-up period was 7.4 years. At the last follow-up, 39/65 patients that displayed a complete or partial response to IL-1 blockers at months 12 were in CID, 3/65 were partial responders, 23/65 became non-responders. Proteomic analysis identified 583 proteins. T-Tests highlighted the differentially expressed proteins between different groups. Significantly dysregulated proteins were identified by comparing complete responder patients and non-responders to anti-IL-1 therapy and age-matched healthy controls. Multivariate analysis identified clinical and proteomic variables predictive for a complete response.

**Conclusion:** The study confirmed the variability of response to anti-IL-1 in sJIA. An early intervention prompted to the higher probability of achieve CID in the first 12 months. The proteomic analysis could be integrated with clinical variables, could help to differentiate patients’ response to anti-IL-1 therapy and identify different disease clusters.

**Patient Consent**

Not applicable (there are no patient data)

**Disclosure of Interest**

None Declared

### Systemic JIA

#### PT05 A potential role of long-lasting IL-18 stimulation in the susceptibility for macrophage activation syndrome in patients with systemic juvenile idiopathic arthritis

##### Greta Rogani^1,2^, Remco Erkens^1,2^, Marein Putmans^1^, Rianne Scholman^1^, Jorg Van Loosdregt^1^, Bas Vastert^1,2^

###### ^1^Center for Translational Immunology, University Medical Center Utrecht; ^2^Division of Pediatric Rheumatology and Immunology, Wilhelmina Children’s Hospital , Utrecht, Netherlands

####### **Correspondence:** Greta Rogani

*Pediatric Rheumatology 2024*, **22(2):**PReS24-ABS-1696

**Introduction:** Macrophage Activation Syndrome (MAS) is a pathologic condition of immune hyperactivation, which occurs in 10-30% of cases of systemic Juvenile Idiopathic Arthritis (sJIA) and represents one of its major cause of mortality. Although targeted biological treatment such as interleukin (IL)-1 or IL-6 blockade is highly effective in inducing clinical inactive disease in most sJIA patients, long term follow-up studies show no reduction in the incidence of MAS^1^. The immunological connection between these two disorders is still incompletely understood. Recently, a population of highly proliferative CD8+ T cells, characterized by the surface activation markers CD38 and HLA-DR, has been identified to be expanded and to act as the main source of interferon-gamma in MAS patients^2^. Although these cells have been thoroughly characterized in MAS, only limited data are available in the disease course of sJIA in general, and the mechanism which leads to their expansion has not yet been elucidated.

**Objectives:** The aim of this research was to evaluate this subset of CD8+ T cells longitudinally in patients with sJIA at different stages of disease. In addition, we sought to identify factors which drive the increase of these cells *in vitro*.

**Methods:** We quantified the percentage of CD38+ HLA-DR+ CD8+ T cells in peripheral blood mononuclear cells (PBMC) in paired samples of patients with active sJIA (*n*=7), inactive sJIA (*n*=6), MAS (*n*=3) and healthy donors (HD) (*n*=5), using flowcytometry. Then, we cultured PBMC and MACS-isolated CD8+ T cells of HD with different stimulants (IL-6, IL-18, IL-6+IL-18, IFNα+IL15, TLR 7-3-9 agonists). The percentage of CD38+ HLA-DR+ CD8+ induction was evaluated over a duration of 7 days.

**Results:** In patients with active sJIA the percentage of CD38+ HLA-DR+ CD8+ cells was significantly increased compared to both HD and patients in remission. Similarly to what was previously reported, the percentage of these CD38+ HLA-DR+ CD8+ cells was even more increased in patients with MAS. Our *in vitro* data confirmed a previous observation that stimulation with IFNα+IL15 induces these activated CD8+ cells after a short-term stimulation of 48 hours. However, at day 7 (representing long term stimulation), IL18 was also able to induce high percentage of CD38+ HLA-DR+ CD8+ cells. The induction of CD38+ HLA-DR+ CD8+ T cells by INFα+IL15 and IL18 over time was comparable between PBMCs and isolated CD8, suggesting a possible direct role of IL-18 stimulation on CD8+ cells.

**Conclusion:** We observed that CD38+ HLA-DR+ CD8+ cells, which have specifically been linked to MAS patients, are also increased in sJIA patients with active disease, representing a potential biological connection between the two disorders. Moreover, the *in vitro* effect of longer lasting IL18 stimulation, known to be high in the plasma of sJIA patients and even higher in MAS patients, induced significantly high percentages of these CD8+ cells. Therefore IL18 might contribute in the expansion of this cell population and, therefore, in the susceptibility for this potentially fatal complication.

**Patient Consent**

Yes, I received consent

**Disclosure of Interest**

G. Rogani: None Declared, R. Erkens: None Declared, M. Putmans: None Declared, R. Scholman: None Declared, J. Van Loosdregt: None Declared, B. Vastert Grant / Research Support with: SOBI, Consultant with: SOBI and Novartis

**References**

1. Erkens R et Al. Arthritis Rheumatol. 2024 Jan;76(1):119-129
2. Huang Z et Al. J Clin Invest. 2023 Nov 15;133(22):e165616

### Systemic JIA

#### PT06 IL-18 levels can differentiate between SJIA and childhood malignancies, aiding in the diagnostic workup for SJIA

##### Arjen Leek^1^, Mao Mizuta^2^, Jurrian de Kanter^3^, Rianne Scholman^4^, Valerie de Haas^3^, Friederike Meyer-Wentrup^3^, Lieve Tytgat^3^, Stefan Nierkens^4^, Julia Drylewicz^4^, Eveline Delemarre^4^, Jorg van Loosdregt^4^, Sebastiaan Vastert^1, 4^

###### ^1^Pediatric Rheumatology, University Medical Center Utrecht, Utrecht, Netherlands; ^2^Pediatric Rheumatology, Prefectural Kobe Children’s Hospital, Kobe, Japan; ^3^Prinses Máxima Centrum for Pediatric Oncology; ^4^Center for Translation Immunology, University Medical Center Utrecht, Utrecht, Netherlands

####### **Correspondence:** Arjen Leek

*Pediatric Rheumatology 2024*, **22(2):**PReS24-ABS-1703

**Introduction:** Systemic JIA (sJIA) exhibits a predominantly autoinflammatory signature and therefore differs markedly from the other JIA subtypes. It is characterized at onset by a quotidian spiking fever, a marked acute phase response, skin rash and enlargement of liver/spleen/lymph nodes. These key features unfortunately are not very specific, so the differential diagnosis of sJIA is broad. An additional challenge is the absence of arthritis during the first months of the disease in about 30% of patients. Therefore, the sJIA phenotype may initially overlap considerably with childhood malignancies, often resulting in a possible diagnostic delay or additional diagnostic procedures such as bone marrow aspiration or PET CT scans in order to rule out malignancy.

**Objectives:** To evaluate the potential of using inflammatory cytokines and chemokines as potential diagnostic biomarkers to differentiate between sJIA and malignancy at onset of disease.

**Methods:** In collaboration with the national pediatric oncology center (Princess Maxima Center) in the Netherlands, we assembled a cohort of patients (as part of prospective cohort studies / biobank studies) sampled at diagnosis before the start of treatment with the following diagnoses: sJIA (*n*=34, of which *n*=9 presenting without arthritis) and childhood malignancies (precursor B-ALL *n*=24, T-ALL *n*=14, AML *n*=12, Hodgkin’s lymphoma *n*=20, non-Hodgkin lymphoma *n*=4, neuroblastoma *n*=15). Clinical and laboratory data were retrospectively extracted from patient files. In total, 17 inflammation associated cytokines and chemokines were measured in serum and plasma using a multiplex immunoassay based on Luminex technology and additionaly the same serum/plasma samples were used for the measurement of 92 protein biomarkers using the OLINK high-multiplex immunoassay “immuno-oncology” panel.

**Results:** All sJIA patients exhibited (spiking) fever in the two weeks leading up to diagnosis, compared to *45*% of malignancy patients (ranging from *72*% in B-ALL to 25% in Hodgkin’s lymphoma). Arthritis or arthralgia, defined as either joint pain or reduced range of motion, was present in 73.5% of sJIA patients compared to 27.5% of malignancy (ranging from 52% in B-ALL to 7.1% in T-ALL). Skin rash was most prevalent in sJIA (85.3% of patients at admittance, compared to 4.4% of malignancy patients). Mean IL-18 levels were significantly elevated in sJIA when compared to malignancy. We evaluated different cut-off values for IL-18 in ROC curves. A value of 1500pg/ml performed best, with a sensitivity of 0.94 and a specificity of 0.83. The OLINK panel (semi-quantitative analysis) suggested several other potential biomarkers, amongst others IL-8, CXCL10, MCP3 and TWEAK. The addition of IL-8 levels (Luminex) slightly improved the model’s performance.

**Conclusion:** We hereby validate the value of IL-18 as a diagnostic biomarker in sJIA, differentiating between sJIA and childhood malignancy. A cut off value of 1500pg/ml in our lab showed the highest AUC in a ROC curve. Our data suggest that patients evaluated for sJIA in a diagnostic work up could reliably be diagnosed with sJIA when having IL-18 levels > 1500pg/ml. For patients with IL-18 levels lower than 1500pg/mL, we suggest to consider a bone marrow aspiration and/or PET CT to rule out malignancy.

**Patient Consent**

Not applicable (there are no patient data)

**Disclosure of Interest**

A. Leek: None Declared, M. Mizuta: None Declared, J. de Kanter: None Declared, R. Scholman: None Declared, V. de Haas: None Declared, F. Meyer-Wentrup: None Declared, L. Tytgat: None Declared, S. Nierkens: None Declared, J. Drylewicz: None Declared, E. Delemarre: None Declared, J. van Loosdregt: None Declared, S. Vastert Grant / Research Support with: Sobi , Consultant with: Sobi / Novartis

### Systemic JIA

#### PT07 Still’s disease associated lung disease: data from the European registry

##### Claudia Bracaglia^1^, Francesca Minoia^2^, Christoph Kessel^3^, Sebastiaan Vastert^4^, Manuela Pardeo^1^, Alessia Arduini^1^, Sarka Fingerhutova^5^, Irina Nikishina^6^, Ozge Basaran^7^, Nural Kiper^7^, Konstantin Belozerov^8^, Mikhail Kostik^8^, Sezgin Sahin^9^, Mia Glerup^10^, Roberta Caorsi^11^, AnnaCarin Horne^12^, Giovanni Filocamo^2^, Helmut Wittkowski^3^, Marija Jelusic^13^, Jordi Anton^14^, Samira Khaldi-Plassart^15^, Alexandre Belot^15^, Gerd Horneff^16^, Seraina Palmer Sarott^17^, Elvira Cannizzaro Schneider^17^, Lampros Fotis^18^, Isabelle Kone-Paut^19^, Ozgur Kasapcopur^9^, Pavla Dolezalova^5^, Seza Ozen^7^, Angelo Ravelli^20^, Fabrizio De Benedetti^1^ and MAS/sJIA working party

###### ^1^Division of Rheumatology, Ospedale Pediatrico Bambino Gesù IRCCS, Roma; ^2^Fondazione IRCCS Ca’ Grande Ospedale Maggiore Policlinico, Milan, Italy; ^3^Department of Pediatric Rheumatology & Immunology, WWU Medical Center (UKM), Münster, Germany; ^4^Pediatric Rheumatology & Immunology, University Medical Center Utrecht, Utrecht, Netherlands; ^5^Centre for Paediatric Rheumatology and Autoinflammatory Diseases, Department of Paediatrics and Inherited Metabolic Disorders, 1st Faculty of Medicine, Charles University and General University Hospital in Prague, Prague, Czech Republic; ^6^V.A. Nasonova Research Institute of Rheumatology, Moscow, Russian Federation; ^7^Department of Pediatrics, Hacettepe University, Ankara, Türkiye; ^8^Saint-Petersburg State Pediatric Medical University, Saint-Petersburg, Russian Federation; ^9^Department of Pediatric Rheumatology, Cerrahpasa Medical School, Istanbul University-Cerrahpasa, Istanbul, Türkiye; ^10^Department of Pediatrics, Aarhus University Hospital, Aarhus, Denmark; ^11^Department of Pediatrics and Rheumatology, IRRCS Istituto G. Gaslini, Genova, Italy; ^12^Department of Pediatric Rheumathology, Karolinska University Hospital and Department of Pediatrics, Karolinska Institute, Stockholm, Sweden; ^13^Department of Pediatrics, University of Zagreb School of Medicine, University Hospital Centre Zagreb, Zagreb, Croatia; ^14^Pediatric Rheumatology, Hospital Sant Joan de Déu, Universitat de Barcelona, Barcelona, Spain, ^15^Pediatric Nephrology, Rheumatology, Dermatology Unit, Hôpital Femme Mère Enfant, Hospices Civils de Lyon, Lyon, France; ^16^Pediatrics, Asklepios Clinic Sankt Augustin, Sankt Augustin, Germany; ^17^Department of Pediatric Rheumatology, University Children’s Hospital Zurich, Zurich, Switzerland; ^18^Pediatric Rheumatology Division, 3rd Department of Pediatrics, National and Kapodistrian University of Athens, “ATTIKON” General University Hospital, Athens, Greece; ^19^Pediatric rheumatology department, CEREMAIA Bicêtre hospital, APHP, Le Kremlin Bicêtre University of Paris Saclay, Paris, France; ^20^IRRCS Istituto Giannina Gaslini and Università degli Studi di Genova, Genova, Italy

####### **Correspondence:** Claudia Bracaglia

*Pediatric Rheumatology 2024*, **22(2):**PReS24-ABS-1276

**Introduction:** Lung disease (LD) is a new emerging severe life-threatening complication of Still’s disease (SD). Patients with SD complicated by LD are increasingly being reported, essentially only from North American. Frequency and features of SD-LD in Europe have never been reported.

**Objectives:** To evaluate the burden of SD associated LD in Europe.

**Methods:** Patients with diagnosis of SD-LD, followed in European paediatric rheumatology centres were identified through a survey sent to the members of the MAS/SJIA Working Party.

**Results:** Data from 58 SD-LD patients, diagnosed in 19 European paediatric rheumatology centres between 2007 and 2024, were collected. 56 patients were White-Caucasian and 2 African American; 37 were female. The median age at SD onset was 7 years and LD occurred after a median time of 3 years. Only 3 patients (5%) had trisomy 21. 88% (51) of the patients had at least one episode of MAS, 23 (45%) of whom had MAS at SD onset and 30 (59%) at time of LD diagnosis; 37 (72%) patients had more than 1 MAS episode. IL-18 levels, measured in 24 (41%) patients, were markedly elevated at time of SD onset and of LD diagnosis (median of 70467 and 89714 pg/ml respectively). 23 (40%) patients experienced drug adverse reaction to a cytokine inhibitor: 15 to tocilizumab, 6 to anakinra and 2 to both. To note, 10 patients (17%) did not receive IL-1 or IL-6 inhibitor before LD developed. Eosinophils count was measured in 52 (89%) patients, 10 (19%) of whom had values >1,000 cell/mmc at time of SD onset and 15 (29%) had values >1,000 cell/mmc at time of LD diagnosis. The HLA-DRB1 was searched in almost half of the patients (48%), and the HLA-DRB1*15 allele was present in 18/28 (64%) patients. After LD diagnosis 51 (88%) patients received intravenous or oral glucocorticoids and the additional treatments were very heterogeneous in the entire cohort. 6 patients underwent hematopoietic stem cell transplantation. LD worsened in 14 patients, of whom 8 developed hypoxia and 6 required O2 supplementation. Half of the patients (29, 50%) required ICU admission. As of now, 11 (22%) patients died.

**Conclusion:** SD-LD patients are also present in Europe. Prompt recognition is crucial and new therapeutic strategies are needed to reduce the risk and improve the outcome of this complication.

**Patient Consent**

Yes, I received consent

**Disclosure of Interest**

C. Bracaglia Consultant with: Sobi, Novartis, Speaker Bureau with: GSK, F. Minoia Consultant with: Sobi, C. Kessel Grant / Research Support with: Novartis, Consultant with: Novartis, Speaker Bureau with: Sobi, S. Vastert: None Declared, M. Pardeo: None Declared, A. Arduini: None Declared, S. Fingerhutova: None Declared, I. Nikishina Speaker Bureau with: Pfizer, Roche, Novartis, Pfizer, Sobi, MSD, R-Pharm, Ipsen, O. Basaran: None Declared, N. Kiper: None Declared, K. Belozerov: None Declared, M. Kostik: None Declared, S. Sahin: None Declared, M. Glerup: None Declared, R. Caorsi Consultant with: Sobi, Novartis, A. Horne: None Declared, G. Filocamo Consultant with: Sobi, H. Wittkowski: None Declared, M. Jelusic: None Declared, J. Anton Grant / Research Support with: Sobi, Novimmune, Novartis, Abbvie, Pfizer, GSK, Roche, Amgen, Lilly, BMS, Sanofi., Consultant with: Sobi, Novimmune, Novartis, Pfizer, GSK., Speaker Bureau with: Sobi, Novimmune, Novartis, GSK, Pfizer., S. Khaldi-Plassart: None Declared, A. Belot Consultant with: SOBI, Novartis, Roche, Pfizer, G. Horneff Grant / Research Support with: MSD, Novartis, Roche, Speaker Bureau with: Abbvie, Chugai, Lilly, Sanofi, Novartis, Pfizer , S. Palmer Sarott: None Declared, E. Cannizzaro Schneider: None Declared, L. Fotis: None Declared, I. Kone-Paut: None Declared, O. Kasapcopur: None Declared, P. Dolezalova: None Declared, S. Ozen Consultant with: Sobi, Novartis, Speaker Bureau with: Sobi, Novartis, A. Ravelli Consultant with: Abbvie, Alexion, Galapagos, Novartis, Pfizer, SOBI, F. De Benedetti Consultant with: Abbvie, Sobi, Novimmune, Novartis, Roche, Pfizer

### Systemic JIA

#### PT08 fuel maladaptive immune responses in the joints of systemic JIA patients

##### Johannes Dirks^1,2^, Claudia Bracaglia^3^, Jonas Fischer^1^, Giusi Prencipe^3^, Hermann Girschick^4^, Manuela Pardeo^3^, Christoph Kessel^5^, Henner Morbach^1^

###### ^1^Department of Pediatrics, Pediatric Immunology and Rheumatology, University Hospital Würzburg, Würzburg; ^2^Site Hamburg-Lübeck-Borstel-Riems, German Center for Infection Research, Hamburg, Germany; ^3^Division of Rheumatology, Ospedale Pediatrico Bambino Gesù, Rome, Italy; ^4^Children’s Hospital, Vivantes Klinikum im Friedrichshain, Berlin; ^5^Department of Pediatric Rheumatology and Immunology, University Children’s Hospital Muenster, Münster, Germany

####### **Correspondence:** Johannes Dirks

*Pediatric Rheumatology 2024*, **22(2):**PReS24-ABS-1271

**Introduction:** Systemic juvenile idiopathic arthritis (sJIA) differs from other forms of JIA by systemic features of inflammation, often preceding the onset of arthritis. While presumed to ensue from an autoinflammatory pathogenesis, the ‘biphasic hypothesis’ suggests that the inflammatory milieu during early sJIA may skew pathogenic T cell responses, that perpetuate arthritis in the chronic phase. This hypothesis is supported by augmented polarization of circulating naïve CD4+ T cells towards peripheral T helper (T_PH_) cells in sJIA patients^1^. T_PH_ cell play pivotal roles in many autoimmune diseases, and their expansion has been documented in inflamed joints of antinuclear antibody positive oligo-/poly- JIA (ANA+ JIA)^2^.

**Objectives:** In this study, we aimed at unraveling the cellular phenotype and the clonal evolution of CD4^+^ T cells in the inflamed joints of sJIA patients during the chronic phase. Specifically, we set out to investigate whether T_PH_ cell expansion occurs in the joints of sJIA patients, and whether these T_PH_ cells might differ from those found in ANA+ JIA on a molecular level.

**Methods:** Deep-phenotyping of synovial fluid (SF) CD4^+^ T cells by flow cytometry and single cell RNA- and V(D)J-sequencing (10X Genomics) of SF CD4+ T cells from sJIA patients (*n*=5) and ANA+ JIA patients (*n*=4).

**Results:** Flow cytometric analysis revealed high frequencies of SF PD-1^hi^HLA-DR^+^CD161^+^T_PH_ cells in sJIA patients, similar to ANA+ oligo/poly-JIA patients but significantly higher than in other forms of childhood arthritis. These cells were characterized by IL-21 or CXCL13 expression together with intermediate levels of IFN-γ production. In scRNA-seq, sJIA SF CD4+ T cells revealed a higher abundance of cells assigned to T_PH_ or Treg clusters and showed significantly upregulated transcriptomic features of a T_PH_ phenotype compared to ANA+ JIA patients. Integrating T cell receptor (TCR) repertoire analysis revealed an unexpected high level of clonal expansion in sJIA CD4^+^ T cells particularly among cells with a T_PH_ phenotype. Comparing clonal connections between clusters identified a higher plasticity in sJIA compared to ANA+ JIA patients. Finally, tracking of clonotypes in two sJIA patients with follow-up samples available, uncovered persisting clones preferentially in T_PH_ and Treg clusters. Application of cytokine signaling signatures to the transcriptomic scRNA-seq data revealed significant differences between ANA+ JIA and sJIA samples and correlations to differential treatment.

**Conclusion:** This comprehensive analysis, integrating cytokine expression, transcriptional profiling and TCR repertoire assessment of SF CD4+ T cells at the single cell level, uncovers mutually pronounced involvement of sustained and dysregulated adaptive immune responses at the site of inflammation during the chronic phase of sJIA. Notably, T_PH_ cells emerge as pivotal drivers of the ‘maladaptive’ T helper cell response observed in sJIA patients.

**Patient Consent**

Yes, I received consent

**Disclosure of Interest**

None Declared

**References**

1. Kuehn J, ... Kessel C. Arthritis Rheumatol. 2023. 10.1002/art.42409.
2. Fischer J, Dirks J, ..., Morbach H. Arthritis Rheumatol. 2022. 10.1002/art.41913.

### COVID-19 (Coronavirus)

#### PT09 Development and initial validation of preliminary criteria of macrophage activation syndrome in multisystem inflammatory syndrome associated with COVID-19 in children

##### Ilia Avrusin^1^, Ludmila Bregel^2,3^, Liudmila Firsova^1^, Olesya Efremova^3^, Alla Vilnits^1,4^, Olga Kalashnikova^1^, Vyacheslav Chasnyk^1^, Mikhail Kostik^1^

###### ^1^Saint Petersburg State Pediatric Medical University, Saint Petersburg; ^2^Irkutsk State Medical Academy of Postgraduate Education, branch of Russian Medical Academy of Continuous Professional Education; ^3^Irkutsk Regional Children’s Clinical Hospital, Irkutsk; ^4^Pediatric Research and Clinical Center for Infection Diseases, Saint Petersburg, Russian Federation

####### **Correspondence:** Ilia Avrusin

*Pediatric Rheumatology 2024*, **22(2):**PReS24-ABS-1550

**Introduction:** Multisystem inflammatory syndrome associated with COVID-19 in children (MIS-C) is a rare, serious disease which affects almost all organs and systems. Macrophage activation syndrome (MAS) seems to be to be one of the most important factors determining the severity of the course of MIS-C, and often requires hospitalization to intensive care unit (ICU), due to its association with unfavorable prognosis for patients. There are no specific validated criteria for MAS in MIS-C patients, and the existing criteria for hemophagocytic syndromes, such as HLH-2004, MAS-2005, and MAS-2016 (Ravelli et. al.) are not optimal for evaluating hemophagocytosis in MIS-C.

**Objectives:** The aim of the study was to develop the MIS-C-specific criteria for early diagnosis of MAS.

**Methods:** The retrospective multicenter cohort study included 166 patients with MIS-C. Two of the most experienced independent experts in the field selected MAS cases based on their expertise. The patients were divided into 3 groups: MAS group (*n*=19), without MAS (*n*=78), and probable MAS (*n*=67), which was identified if there was no consensus among the experts. The third group was excluded from the analysis. Variables which allowed to differentiate MAS were used to develop and validate the diagnostic score.

**Results:** Patients with MAS were significantly older (9 years 8 months vs. 6 years 4 months, p<0.0008), and had higher prevalence of such clinical signs as edematous syndrome (86.7 vs. 31.6%, p<0.0006), hypotension and/or shock (68.4 vs. 23.1% p<0.0001), splenomegaly (94.1 vs. 26.9% p<0.000003), CNS involvement (83.3 vs. 40.8%, p<0.005). Among laboratory parameters thrombocytopenia (94.7 vs. 18%, p<0.000001), hypoalbuminemia (25.0 vs. 30.2 g/l, p<0. 0000001), hypertriglyceridemia (3.6 vs. 2.0 mmol/L, p<0.003) were more often observed in MAS-MIS-C patients. They also had higher levels of inflammatory biomarkers such as ferritin (100 vs. 63.4%, p<0.0000001), CRP (23.2 vs. 9.9 mg/dL, p<0.00007), troponin (117.7 vs. 2.0 pg/mL, p<0.001), as well as AST (68.7 vs. 38.0 IU/l, p<0.0000001), and ALT (62.0 vs. 30.1 IU/l, p<0.0003). There were signs of coagulation abnormalities: fibrinogen (2.7 vs. 5.5 g/L, p<0.000002), and D-dimer (3460 vs. 944 ng/mL, p<0.000008) in the MAS-MIS-C group. We selected continuous and categorical variables with statistical significance, and analysis of sensitivity and specificity with OR calculation was done to find the 21 main predictor of MAS in MIS-C.

Two of them were used to develop the criteria: ferritin > 469 ng/L (65 points), platelets < 114 x 109/L (35 points), were included in the diagnostic score (*p*=5x10-31).

**Conclusion:** The obtained diagnostic criteria allow differentiation of MAS in MIS-C patients with a sensitivity of 100% and specificity of 94.9% and can be used, along with other diagnostic tests and procedures.

**Patient Consent**

Not applicable (there are no patient data)

**Disclosure of Interest**

None Declared

### Immunoregulation and basic science

#### PT10 Ferroptosis in SJIA and MIS-C: strike while the iron is hot

##### Freya Huijsmans^1^, Lysa Langevin^2^, Jorg van Loosdregt^1^, Bas J. Vastert^1^, Rae S. M. Yeung^2,3,4,5^

###### ^1^Pediatric Rheumatology and Immunology, UMC Utrecht, Utrecht, Netherlands; ^2^Cell Biology Program, The Hospital for Sick Children; ^3^Immunology, University of Toronto; ^4^The Institute of Medical Science; ^5^Division of Rheumatology, Department of Pediatrics, The Hospital for Sick Children, Toronto, Canada

####### **Correspondence:** Freya Huijsmans

*Pediatric Rheumatology 2024*, **22(2):**PReS24-ABS-1753

**Introduction:** Systemic Juvenile Idiopathic Arthritis (sJIA) and Multisystem inflammatory syndrome in children (MIS-C) are both hyperinflammatory syndromes of childhood that share clinical features and immunobiology. For both entities inflammasome activation is involved, highlighted by expression of IL-1β and IL-18. Ferroptosis has been described to be a very immunogenic form of cell death, which is distinct from conventional cell death modes as it uniquely hinges on dysregulated iron metabolism and is characterized by unrestricted lipid peroxidation of phospholipids, resulting in plasma membrane rupture and release of damage-associated molecular pattern signals (DAMPs). There is growing evidence that ferroptosis is involved in the activation of several inflammatory pathways, including the inflammasome and STING. Correspondingly, ferroptosis has been implicated in the pathogenesis of COVID-19 and inflammatory arthritis.

**Objectives:** To determine whether the ferroptosis pathway is activated in patients with sJIA and MIS-C and is involved in the pathogenesis of these hyperinflammatory syndromes.

**Methods:** We measured lipid peroxidation and assessed the expression of ferroptosis-associated genes in patient samples. Malondialdehyde (MDA) was used as a readout of ferroptosis as it is the final product of lipid peroxidation. MDA levels were measured in plasma of 52 MIS-C patients. Moreover, RNA sequencing was performed on neutrophils, monocytes, CD4 and CD8 T cells from patients with active sJIA (*n*=3-7) and healthy controls (*n*=3-9).

**Results:** MDA levels were highly elevated in our pre-treatment MIS-C cohort (*n*=35, median 177.9 nmol/ml) and were undetectable in 14 out of 19 (74%) post-treatment samples. Moreover, MDA levels were significantly higher in MIS-C patients with aneurysm formation, compared to patients with no aneurysms (median 248.81 versus 159.84; *p* = 0.0080) and correlated positively with coronary artery Z-scores (Spearman r 0.40, *p*=0.0180). Furthermore, gene set enrichment analysis showed that the expression of genes associated with the ferroptosis pathway is significantly enriched in neutrophils, monocytes, CD4 and CD8 cells of active sJIA patients compared to healthy controls.

**Conclusion:** Our findings provide new insights into the pathobiology of sJIA and MIS-C and indicate the involvement of the ferroptosis pathway. Obtaining a better understanding of the interplay between ferroptosis, inflammatory pathways, and other types of cell death is important as this could provide novel avenues for clinical treatment strategies of hyperinflammatory syndromes in children.

**Patient Consent**

Not applicable (there are no patient data)

**Disclosure of Interest**

F. Huijsmans: None Declared, L. Langevin: None Declared, J. van Loosdregt: None Declared, B. Vastert Grant / Research Support with: SOBI, Consultant with: Novartis and SOBI, R. Yeung: None Declared

### Systemic lupus erythematosus and antiphospholipid syndrome

#### PT11 Unveiling the largest cohort of monogenic lupus in Oman: insights from DNASE1L3 Mutations

##### Reem Abdwani^1^, Mahadev Jetho Mal^2^, Eman Al Masroori^2^, Safiya Al Abrawi^3^

###### ^1^College of Medicine, Sultan Qaboos University; ^2^Child Health, University Medical City; ^3^Child Health, Royal Hospital, Muscat, Oman

####### **Correspondence:** Reem Abdwani

*Pediatric Rheumatology 2024*, **22(2):**PReS24-ABS-1192

**Introduction:** Monogenic lupus in children is a rare but significant form of lupus where the disease is caused by mutations in single genes, in contrast to the more common multifactorial forms of lupus. To date, more than 30 genes have been identified that can cause monogenic forms of lupus [1]. Monogenic lupus caused by DNASE1L3 deficiency leads to the accumulation of extranuclear nucleic acids, which can be recognized by toll-like receptors, causing type I interferon production, or leading to the production of autoantibodies and immune complex-related tissue damage [2].

**Objectives:** In this study, we aim to describe the largest cohort of monogenic lupus caused by DNASE1L3 yet reported describing its phenotypic characteristic and outcome.

**Methods:** We performed a multicenter retrospective cohort using data from all cSLE patients followed in the pediatric rheumatology tertiary centers in the Sultanate of Oman. We included patients diagnosed with cSLE diagnosed before the age of 13 years with genetically confirmed DNASE1L3 mutation. All patients fulfilling the 1997 ACR and 2012 SLICC classification criteria with at least 6 months of follow-up.

**Results:** We recruited 33 patients from 15 families with confirmed homozygous 1-bp deletion in DNASE1L3 (NM_004944.2): c.643delT (p.Trp215GlyfsX2). The number of patients affected by DNASLE1L3 mutation in each family, ranged from 1-5. The median age of disease onset was 4 years, with *n*=55% of cohort presenting before the age of 5 years. There were more boys (*n*=20) than girls (*n*=13) affected. The most common clinical features were arthritis (82%) and nephritis (60%), urticarial vasculitis (79%), fever (49%) and conjunctivitis (25%). The SLEDAI score on presentation 13.3 ± 3.64 (SD). Aggressive treatment with Azathioprine *n*=15 (46%), MMF *n* = 20 (60%), Cyclophosphamide *n*= 8 (24%) and Rituximab *n*= 6 (18%) were used. Disease damage was identified in *n*=14 (42%) of patients with mean damage index score of 2.14 ± 1.12 (SD). Table 1,2,3 & 4 compares monogenic (DNASE1L3) cSLE vs non familial cSLE cohort from Oman in terms of demographic features, clinical manifestation, SLEDAI score, damage index score, laboratory features and treatment.

**Conclusion:** Given the early onset, aggressive nature and refractory natures of cSLE caused by DNASE1L3 mutation, further research aimed at targeted therapies that could restore the function of DNASE1L3 or compensate for its deficiency is warranted.

**Patient Consent**

Not applicable (there are no patient data)

**Disclosure of Interest**

None Declared

**References**

1. Omarjee O, Picard C, Frachette C, Moreews M, Rieux-Laucat F, Soulas-Sprauel P, Viel S, Lega JC, Bader-Meunier B, Walzer T, Mathieu AL, Cimaz R, Belot A. Monogenic lupus: Dissecting heterogeneity. Autoimmun Rev. 2019;18(10):102361
2. Gerovska D, Araúzo-Bravo MJ. Systemic Lupus Erythematosus Patients with DNASE1L3·Deficiency Have a Distinctive and Specific Genic Circular DNA Profile in Plasma. Cells. 2023;12(7):1061.

### Systemic lupus erythematosus and antiphospholipid syndrome

#### PT12 Contributors to damage accrual in Childhood-onset Systemic Lupus Erythematosus (CSLE): influence of corticosteroids and disease activity

##### Maria Hanif^1^, Chandni Sarker^1^, Michael W. Beresford^1, 2^, Eve M. D. Smith^1, 2^ and on behalf of the UK JSLE Cohort Study

###### ^1^Institute of Life Course and Medical Sciences; ^2^Alder Hey Children’s NHS Foundation Trust Hospital, Liverpool, United Kingdom

####### **Correspondence:** Maria Hanif

*Pediatric Rheumatology 2024*, **22(2):**PReS24-ABS-1087

**Introduction:** Childhood-onset Systemic Lupus Erythematosus (cSLE) is a chronic autoimmune disorder that can cause early organ damage, making it crucial to identify predictors of damage.

**Objectives:** To identify independent predictors of damage in patients stratified by levels of disease activity and treatment.

**Methods:** Analysis included UK JSLE Cohort Study participants diagnosed ≤18 years and meeting ≥4 ACR 1997 SLE classification criteria. New damage was defined as an increase in ≥1 unit in the SLICC SDI score. Univariable and multivariable Prentice-Williams-Peterson gap-time models investigated how demographics, time-adjusted mean prednisolone dose, methylprednisolone exposure, time-adjusted mean Physicians Global Assessment (PGA) score, baseline organ damage and average disease activity (time-adjusted average SLEDAI-2K score, AMS) influenced the hazards of new damage. Analyses were performed across the entire cohort and four subgroups: (1) minimal activity (AMS≤2), (2) low activity (AMS≤4), (3) moderate-high activity (AMS>4), and (4) those with no corticosteroids during follow-up.

**Results:** 430 patients were included with a median of 10 visits/patient, with 66/410 patients displaying organ damage at first visit and 99/430 accruing further damage. Baseline organ damage predicted further damage in patients with no corticosteroids (HR 3.64 CI 1.83-7.24, p<0.001) and in those with minimal disease activity (HR 1.33 CI 1.78-8.08, *p*=0.001) during follow-up. For those in low disease activity during follow-up, methylprednisolone exposure and time-adjusted mean PGA score were associated with damage accrual (HR 2.61 CI 1.04-6.53 and HR 3.41 CI 1.52-7.67 respectively, both p<0.05). Methylprednisolone exposure, time-adjusted mean PGA score and AMS score were all significant predictors of damage in the whole cohort (HR 2.20 CI 1.33-3.62, HR 2.87 CI 1.48-5.56 and HR 1.13 CI 1.03-1.24 respectively, all p<0.05) and the moderate-high disease activity subgroup (HR 2.29 CI 1.31-4.00, HR 2.66 CI 1.20-5.87 and HR 1.15 CI 1.03-1.29 respectively, all p<0.05).

**Conclusion:** Methylprednisolone exposure is a significant, modifiable risk factor for damage in cSLE, warranting further research to optimise paediatric dosage regimens. Baseline organ damage predicts occurrence of further damage, underscoring the need for early specialist referral and optimising initial treatment. In patients with an AMS>4, a 1 unit increase in SLEDAI raises damage risk by 15%. This correlation was absent in the AMS≤4 subgroup, indicating maintenance of low disease activity through T2T strategies could mitigate damage accrual.

**Date of birth:** 11/24/2001

**Patient Consent**

Yes, I received consent

**Disclosure of Interest**

None Declared

### Systemic lupus erythematosus and antiphospholipid syndrome

#### PT13 Anti-CD19 CAR-T cell therapy for refractory Childhood-onset Systemic Lupus Erythematosus (CSLE) with severe lung and renal involvement

##### Claudia Bracaglia^1^, Pietro Merli^2^, Patricia Moran-Alvarez^1^, Francesca Del Bufalo^2^, Virginia Messia^1^, Rebecca Nicolai^1^, Emiliano Marasco^1^, Mattia Algeri^2^, Concetta Quintarelli^2^, Chiara Rosignoli^2^, Maria Giuseppina Cefalo^2^, Marco Becilli^2^, Francesca Diomedi Camassei^3^, Linda Hanssens^4^, Francesco Emma^5^, Fabrizio De Benedetti^1^, Franco Locatelli^2,6^

###### ^1^Division of Rheumatology; ^2^Department of Hematology/Oncology, Cell and Gene Therapy; ^3^Pathology Unit, Ospedale Pediatrico Bambino Gesù IRCCS, Roma, Italy; ^4^Miltenyi Biomedicine, Bergisch-Gladbach, Germany; ^5^Division of Nephrology, Ospedale Pediatrico Bambino Gesù IRCCS; ^6^Catholic University of the Sacred Heart, Roma, Italy

####### **Correspondence:** Claudia Bracaglia

*Pediatric Rheumatology 2024*, **22(2):**PReS24-ABS-1198

**Introduction:** cSLE has a more severe clinical course, higher frequency of major organ involvement and lower survival rates compared to adult SLE. Anti-CD19 CAR-T cells have shown excellent efficacy and tolerability in few adults and one adolescent with SLE.

**Objectives:** To report two cases of refractory cSLE with severe lung and renal involvement successfully treated with CAR-T cells.

**Methods:** A single infusion of fresh, autologous, second-generation anti-CD19 CAR-T product (lentivirus) manufactured on the Prodigy device was administered (1x10^6^ CAR-T cells/kg) after lymphodepletion with cyclophosphamide (CYC), (1000 mg/m^2^) and fludarabine (90 mg/m^2^).

**Results:** Case 1. 15-year-old female with haematological involvement, interstitial lung disease (ILD) with pulmonary hypertension (PH), class II/V lupus nephritis (LN), pericarditis. She failed multiple treatments, including high-dose glucocorticoids (GC), MMF, RTX, and CYC. She became GC dependent and had 2 severe systemic infections, requiring prolonged hospitalization.

For persistent disease activity (SLEDAI=22) and vital organ involvement, CAR-T cells were administered. Immunosuppression was withdrawn before CAR-T. She presented G1 CRS, anaemia (G2) and neutropenia (G3). No infection or neurotoxicity were observed. Once infused, CAR-T cells expanded (peak at d12=52.41 cells/mL) with B-cell depletion at d7. SLEDAI normalized at m3 with sustained drug-free remission at m11. Improvement of LD documented by HRCT and of PH allowing discontinuation of bosentan and sildenafil occurred. Complement normalized at wk 6. ANA and anti-dsDNA decreased progressively, while proteinuria normalized at wk 4. Renal biopsy at m6 showed absence of complement and IgG deposits. B-cell recovery occurred at m4, without SLE flare.

Case 2. 16-year-old female with haematological involvement, class V LN with tubulointerstitial nephritis, ILD and optic neuritis. She had 2 life-threating episodes of diffuse alveolar haemorrhage (DAH) despite several treatments (plasma exchange, high-dose GC, CYC and MMF). Due to long-term use of high-dose GC, she developed hypertension, GC-associated diabetes, avascular necrosis, and severe systemic Aspergillus infection.

For persistent disease activity (SLEDAI=38) CAR-T cells were administered after withdrawal of immunosuppression. She presented G1 CRS. SLEDAI normalized at m5. She had no new DAH episodes and LD improved at HRCT. C3/C4, ANA and anti-dsDNA normalized at m1, while proteinuria normalized at m5. B-cell aplasia is still present at last follow-up, without SLE flare.

**Conclusion:** In these 2 cSLE patients, with severe lung and renal involvement, refractory to multiple treatments, B-cell depletion and drug-free remission were rapidly achieved after a single infusion of CAR-T cells. Long-term efficacy needs to be assessed.

**Patient Consent**

Yes, I received consent

**Disclosure of Interest**

C. Bracaglia Consultant with: Sobi, Novartis, Speaker Bureau with: GSK, P. Merli Consultant with: Sobi, P. Moran-Alvarez: None Declared, F. Del Bufalo: None Declared, V. Messia: None Declared, R. Nicolai: None Declared, E. Marasco: None Declared, M. Algeri: None Declared, C. Quintarelli: None Declared, C. Rosignoli: None Declared, M. G. Cefalo: None Declared, M. Becilli: None Declared, F. Diomedi Camassei: None Declared, L. Hanssens Employee with: Miltenyi Biomedicine, F. Emma: None Declared, F. De Benedetti Consultant with: Abbvie, Apollo, Elixiron, Kiniksa, Novartis, Roche, Pfizer, Sanofi and Sobi, F. Locatelli Consultant with: AMGEN, Jazz Pharmaceutical, Novartis, Miltenyi, Gilead, Sanofi, Sobi, Vertex, Speaker Bureau with: AMGEN, Jazz Pharmaceutical, Novartis, Miltenyi, Gilead, Sanofi, Sobi, Vertex

**Reference**

1. Mackensen A, et al. Anti-CD19 CAR T cell therapy for refractory systemic lupus erythematosus. Nat Med. 2022 Oct;28(10):2124-2132. 10.1038/s41591-022-02017-5.

### Systemic lupus erythematosus and antiphospholipid syndrome

#### PT14 A feasibility study: performing fitness testing in JSLE routine clinical MDT clinic

##### Verna J. Cuthbert, Hanna Lythgoe

###### Paediatric Rheumatology, Manchester Childrens’ Hospital, Manchester, United Kingdom

####### **Correspondence:** Verna J. Cuthbert

*Pediatric Rheumatology 2024*, **22(2):**PReS24-ABS-1310

**Introduction:** Juvenile-onset systemic lupus erythematosus (JSLE) is a chronic inflammatory condition with known associated adverse long term cardiovascular outcomes. It is also well established that patients with inflammatory conditions in childhood are less fit compared with their peers. It remains unclear if addressing cardiovascular risk factors such as reduced fitness in childhood improves long-term cardiovascular outcome in SLE. Improving the long-term cardiovascular outcomes in SLE remains crucial and therefore we have introduced fitness testing in the JSLE clinic.

**Objectives:** This pilot project seeks to explore the feasibility of routine fitness testing in our JSLE MDT clinic at Manchester children’s Hospital, and patients and parents’ perspectives. Additionally, Tertiary Paediatric Rheumatology centres in the UK have been surveyed to gain further understanding of what fitness testing is currently being carried out.

**Methods:** The Kasch pulse recovery (KPR) test has been used to measure paediatric fitness (1). The KPR is a sub-maximal exercise test suitable for our SLE population. It is safe, easy to use, free, quick to do in clinic and only requires minimal equipment usually found in a clinic setting: HR monitor, timer, Step (30 cm), and a metronome, (which can be accessed via a free app on phone). It can be carried out in a clinic room setting in outpatients. The KPR test consists of climbing a 0.3m step at a rate of 24 steps up and down per minute defined by using a metronome. The HR is monitored during the test and during recovery. There is limited normative data available for an under 18 population. The Kasch fitness test is carried out within routine MDT SLE clinic by the physiotherapist. The test takes approximately 5 mins to carry out. Comparison with age matched healthy peers helps to stimulate discussion re health benefits of exercise in clinic. Patient and parent feedback was sought using paper/online forms. Healthcare professionals were surveyed using an online questionnaire.

**Results:** Testing has been performed in 32 JSLE patients to date. Initial patient and parent feedback suggests that it is an acceptable test for them. Preliminary results from the healthcare professional survey (*n*=7) found that only 2 centres are currently using fitness testing in certain inflammatory patient groups. 4 centres think it is important, 2 not sure and 1 centre did not think measuring cardiorespiratory fitness was important.

Barriers to using fitness testing given as: *insufficient equipment, limited time or not thought as relevant.*

**Conclusion:** Initial survey results show a sparse use of fitness testing for paediatric rheumatology patients nationally in the UK, however there is interest in such an outcome measure. Practical considerations for such testing are important in clinical areas. More detailed feedback data from patients and families using semi-structured interviews is currently ongoing. We hope to generate further discussion for use of fitness testing in JSLE and other paediatric rheumatology populations.

**Patient Consent**

Not applicable (there are no patient data)

**Disclosure of Interest**

None Declared

**Reference**

1. Jankowske M et al. Pediatr Cardiol 36:27-32

### Systemic lupus erythematosus and antiphospholipid syndrome

#### PT15 Alternative allele counts associate with ancestry, age at disease onset, organ involvement and disease severity in juvenile-onset systemic lupus erythematosus

##### Valentina Natoli^1,2^, Amandine Charras^1^, Megan Hasoon^3,4^, Eva Caamano-Gutierrez^3,4^, Eve M. Smith^1,5^, Michael W. Beresford^1,5^, Christian M. Hedrich^1,5^ and UK JSLE Cohort Study

###### ^1^Department of Women’s & Children’s Health, Institute of Life Course and Medical Sciences, University of Liverpool, Liverpool, United Kingdom; ^2^Dipartimento di Neuroscienze, Riabilitazione, Oftalmologia, Genetica e Scienze Materno-infantili, Università degli Studi di Genova, Genoa, Italy; ^3^Computational Biology Facility, Liverpool Shared Research Facilities, Faculty of Health and Life Sciences; ^4^Institute of Systems, Molecular and Integrative Biology, University of Liverpool; ^5^Department of Paediatric Rheumatology, Alder Hey Children’s NHS Foundation Trust Hospital, Liverpool, United Kingdom

####### **Correspondence:** Valentina Natoli

*Pediatric Rheumatology 2024*, **22(2):**PReS24-ABS-1092

**Introduction:** Systemic Lupus Erythematosus (SLE) is a systemic autoimmune/inflammatory condition characterized by extremely variable clinical presentations. While across age groups, genetic determinants play a key pathomechanistic role, they are likely to have a more pronounced role in juvenile-onset (jSLE) when compared to adult-onset SLE patients, contributing to earlier disease-onset, more severe phenotypes and less favorable outcomes.

**Objectives:** This study investigated the relationship between alternative allele counts (AAC), and age at disease onset, sex, ancestry, organ involvement and clinical severity in a multi-ethnic jSLE cohort from the UK. It furthermore explored associations between gene-level alternative allele scores (GAAS), organ involvement and disease severity.

**Methods:** 315 jSLE patients from the UK JSLE Cohort Study were studied using a panel sequencing approach covering 62 genes/genomic regions. Demographic and clinical features, along with disease activity (pBILAG-2004 and SLEDAI) and damage index (SLICC-SDI) scores were recorded. AAC were generated by counting the number of alternative alleles within a SNP and multiplying the AAC by their *in silico* predicted functional impact of that SNP on gene function (low or modifier=1, moderate=2, high=3) and then summed across all SNPs to give a patient level AAC. GAAS was calculated, similarly to the AAC, by counting the number of alternative alleles and multiplying by functional impact, and then summed across SNPs within a gene, to give a GAAS for each gene and patient. A generalised linear model, adjusted for ethnicity, sex and family history, was used to assess their relationship with organ involvement and severity.

**Results:** A (weak) inverse correlation between age at diagnosis and AAC was observed (R=-0.15, *p*=0.01), when ethnicity was not considered. Notably, the inverse correlation between age at disease-onset and AAC across ethnicities was primarily influenced by South-Asian patients (R=-0.28, p<0.001).

Across the whole cohort, constitutional (p<0.001), renal (p=0.001), haematological (p=0.001) and neuropsychiatric (p=0.03) involvement were significantly associated with AAC.

When examining cumulative GAAS across ethnicities, higher scores were recorded in patients of Black African/Caribbean ancestry when compared to White Caucasians, and East and South Asian participants (p<0.001). GAAS associated significantly with some clinical variables, including severity of neuropsychiatric (*ACP5, TYK2* and *RNASEH2A*, p<0.001; RASGRP3, p=0.04) and renal (*ACP5*, *ITGAM*, and *LYN*, p<0.001; *TNFAIP3*, p=0.007) involvement.

**Conclusion:** Genetic variability contributes to early disease expression, especially in South-Asian jSLE patients. Some GAAS associate with specific organ involvement and severity across ethnicities. Observations from this study may allow for future genetic risk assessment and patient stratification towards individualized treatment and care.

**Patient Consent**

Yes, I received consent

**Disclosure of Interest**

V. Natoli: None Declared, A. Charras: None Declared, M. Hasoon: None Declared, E. Caamano-Gutierrez: None Declared, E. Smith: None Declared, M. Beresford: None Declared, C. Hedrich Grant / Research Support with: Unrestricted research funding from Merck to study lupus nephritis

### Systemic lupus erythematosus and antiphospholipid syndrome

#### PT16 Identifying cross-registry commonalities to facilitate international pediatric antiphospholipid syndrome research

##### Selcan Demir^1^, Mojca Z. Avramovič^2^, Elizabeth Sloan^3^, Jheel Pandya^4^, Doruk Erkan^5^, Seza Ozen^6^, Tadej Avcin^2^

###### ^1^Department of Pediatric Rheumatology, Eskisehir Osmangazi University, Eskisehir, Türkiye; ^2^Department of Allergology, Rheumatology and Clinical Immunology, University Medical Centre Ljubljana, Ljubljana, Slovenia; ^3^Department of Pediatric Rheumatology, University of Texas Southwestern Medical Center, Dallas; ^4^Department of Pediatric Rheumatology; ^5^Department of Rheumatology, Hospital for Special Surgery, New York, United States; ^6^Department of Pediatric Rheumatology, Hacettepe University, Ankara, Türkiye

####### **Correspondence:** Selcan Demir

*Pediatric Rheumatology 2024*, **22(2):**PReS24-ABS-1661

**Introduction:** Antiphospholipid syndrome (APS) is a systemic autoimmune disease defined as the development of venous and/or arterial thromboses with persistently positive antiphospholipid antibodies. Although pediatric APS is rare, it is associated with significant morbidity and mortality and evidence-based data regarding its diagnosis and management are limited. Much of the current knowledge on pediatric APS is from a 2009 review of 121 cases through the Ped-APS Registry which was initiated as a joint project of the European aPL Forum and the Juvenile SLE Working Group of PReS. In North America, CARRA hosts the largest ongoing observational registry of children with SLE and APS, though limited data have been published on the subset with APS. The recently introduced 2023 ACR-EULAR APS classification criteria, developed for adult patients, have significantly higher specificity compared to the revised Sapporo criteria and provide a new basis for future APS research. However, there is still a lack of pediatric-specific APS criteria and current classification criteria were only validated for adult population studies.

**Objectives:** To develope an international data set to facilitate international registry based pediatric APS research.

**Methods:** We initiated by comparing data fields from existing European and North American registries, including the Ped-APS Registry and the CARRA Lupus-APS Subgroup Registry. This comparative analysis aimed to identify commonalities and discrepancies in data collection practices across registries. Additionally, we conducted a systematic literature review on Pubmed to identify relevant studies focusing on pediatric APS between 2003-2024. In the second phase of our study, we plan to utilize the Delphi methodology and convene a consensus meeting among experts to define core clinical and laboratory data fields that should be collected and harmonized between international registries for pediatric APS.

**Results:** A comprehensive literature search was conducted and yielded 2,105 articles. Of these,1607 articles were excluded for the following reasons (reasons determined a priori): 25 articles because we were not able to retrieve them, 123 articles because they were not in English, 453 articles that were about pregnancy/obstetric APS, 519 articles that included adults without sufficient data on pediatric patients and 487 articles that were not relevant. We included 498 articles, out of which 311 were case reports/case series with a maximum of 10 patients included. Resulting from this, we have identified 325 candidate items for the registry. Of them, 102 from Pediatric APS Registry and CARRA registry review, 99 from the recently published adult classification criteria, and 124 from the systematic literature review. We prepared a list of candidate items for Delphi questionnaire for the following areas: demographics, comorbidities, family history, laboratory testing, involved systems, anticoagulation, antiaggregant and immunosuppressive treatment.

**Conclusion:** Through systematic evaluation of existing data sources, literature review, and preparation for Delphi and expert consensus, we are laying a solid foundation for the development of a robust and collaborative registry for pediatric APS. This global dataset will facilitate international collaboration and research efforts to improve outcomes for children with APS worldwide.

Research Support: CARRA-PReS Collaborative Award

**Date of birth:** septembre

**Patient Consent**

Not applicable (there are no patient data)

**Disclosure of Interest**

None Declared

### Systemic lupus erythematosus and antiphospholipid syndrome

#### PT17 Involvement of type 1 interferon in anti-nuclear antibody-associated childhood immune thrombocytopenic purpura: a pre-lupus condition

##### Jerome Granel^1,2,3^, Helder Fernandes^1,2^, Pascal Pillet^1,3^, Olivier Richer^1,3^, Johanna Clet^1,3^, Elodie Wojciechowski^4^, Patrick Blanco^4^, Alexandre Belot^5^, Nathalie Aladjidi^1,2^

###### ^1^Paediatric Clinical Immunology, Pellegrin Hospital, CIC1401, INSERM CICP, ^2^Centre de Référence National des Cytopénies Auto-immunes de l’Enfant (CEREVANCE), ^3^Centre de Référence des Rhumatismes inflammatoires et maladies Auto-Immunes Systémiques rares de l’Enfant (RAISE), ^4^Immunology laboratory, Bordeaux University Hospital, Bordeaux, ^5^Pediatric Rheumatology, Nephrology and Dermatology Department, Hospices Civils de Lyon, Lyon, Lyon, France

####### **Correspondence:** Jerome Granel

*Pediatric Rheumatology 2024*, **22(2):**PReS24-ABS-1379

**Introduction:** Anti-nuclear antibody-associated immune thrombocytopenic purpura (ITP-ANA+) is a pre-lupus condition, called incomplete lupus erythematous (ILE), as 20% of children will develop systemic lupus erythematosus (SLE) (1). In adults with ILE, elevation of interferon score (IS), measuring the expression of a panel of type 1 interferon-stimulated genes, is associated with progression to SLE (2) and hydroxychloroquine could prevent progression to SLE by reducing type 1 interferon signaling (3).

**Objectives:** Description of the IS in childhood-onset ITP-ANA+.

**Methods:** Thirty-four children followed at the Bordeaux University Hospital had an IS evaluation in the Immunology Department of the Lyon University Hospital in 2022-2023. A score was positive if > 2.3.

**Results:** Eleven of the 34 children had ITP-ANA+, 9 were girls and median age at ITP diagnosis was 13 years (1.1-15.7). At IS evaluation, ITP was acute (n=2), persistent (n=3) or chronic (n=6). IS was positive in 9/11 (82%) children with a median score of 18 (1.2-68). This median IS was higher than in 2 children with ITP-ANA- (2.4 (1.5-3.3)), and than in 11 children with juvenile arthritis (1.9 (0.9-6.5), p=0.004), but not different than in 10 children with SLE (35 (2.1-90), p=0.3). Among ITP-ANA+ children, IS was significantly higher in patients with ITP diagnosed after the age of 11 (n=7, p=0.02), within the first year after ITP diagnosis (n=5, p=0.02), in the presence of anti-SSA (n=5, p=0.02), and in the absence of a treatment by hydroxychloroquine (n=5, p=0.048). Finaly, hydroxychloroquine reduced the IS in 3/3 children with available pre- and post-treatment scores: from 30, 51, and 68, to 1.1, 20, and 34, respectively (p=0.002).

**Conclusion:** These results suggest the involvement of the type 1 interferon signaling in the pathophysiology of childhood ITP-ANA+. Further studies are needed to define the variables associated with this IS and progression from ILE to SLE, and to use it for early treatment by hydroxychloroquine.

**Patient Consent**

Yes, I received consent

**Disclosure of Interest**

None Declared

**References**

1. Granel J, Fernandes H, Bader-Meunier B, Guth A, Richer O, Pillet P, et al. Antinuclear antibodies associated auto-immune cytopenia in childhood is a risk factor for systemic lupus erythematosus. Blood. 16 janv 2024;blood.2023021884.
2. Md Yusof MY, Psarras A, El-Sherbiny YM, Hensor EMA, Dutton K, Ul-Hassan S, et al. Prediction of autoimmune connective tissue disease in an at-risk cohort: prognostic value of a novel two-score system for interferon status. Ann Rheum Dis. oct 2018;77(10):1432‑9.
3. Olsen NJ, Karp DR. Hydroxychloroquine: An Old Drug With New Tricks. J Rheumatol. juin 2021;48(6):796‑8.

### Systemic lupus erythematosus and antiphospholipid syndrome

#### PT18 The usefulness of white blood cell inflammatory parameters in children with systemic lupus erythematosus

##### Butsabong Lerkvaleekul^1^, Nongnuch Sirachainan^1^, Duantida Songdej^1^, Karan Paisooksantivatana^2^, Pawadee Chinudomwong^2^, Soamarat Vilaiyuk^1^

###### ^1^Department of Pediatrics; ^2^Department of Pathology, Faculty of Medicine Ramathibodi Hospital, Bangkok, Thailand

####### **Correspondence:** Butsabong Lerkvaleekul

*Pediatric Rheumatology 2024*, **22(2):**PReS24-ABS-1141

**Introduction:** Systemic lupus erythematosus (SLE) is a chronic inflammatory disease involving dysregulation of innate and adaptive immune cells. Since neutrophils and lymphocytes play a crucial role in systemic inflammation, white blood cell inflammatory parameters that can be incorporated into the routine differential blood count might be used in clinical practice to assess disease activity in children with SLE.

**Objectives:** To evaluate the relation among white blood cell inflammatory parameters, inflammatory markers, including erythrocyte sedimentation rate (ESR), c-reactive protein (CRP), and procalcitonin (PCT), and disease activity in SLE.

**Methods:** Sixty-seven SLE patients (36 active and 31 inactive status) and 112 aged match healthy controls (HC) were enrolled. White blood cell inflammatory parameters describing activated cell populations such as neutrophil reactivity intensity (NEUT-RI), neutrophil granularity intensity (NEUT-GI), the width of dispersion of neutrophil complexity (NE-WX), the width of dispersion of neutrophil fluorescence (NE-WY), the width of dispersion of neutrophil size (NE-WZ), reactive lymphocytes (RE-LYMP), antibody-synthesizing lymphocytes (AS-LYMP), and immature granulocytes (IG) were evaluated by the XN-series hematology analyzer (Sysmex Corp., Japan). Demographic data and laboratory parameters were also collected. We used the SLE disease activity index 2000 (SLEDAI-2K) and physician global assessment (PGA) on a 0–3 visual analog scale to measure disease activity. Inactive disease was defined as clinical SLEDAI-2K (cSLEDAI-2K), excluding anti-double stranded DNA (anti-dsDNA) and complement, equal to zero, regardless of medication.

**Results:** The median values of NEUT-RI, NE-WX, NE-WY, NE-WZ, %RE-LYMP, %AS-LYMP, and %IG were significantly higher in active SLE than in HC. In addition, the median values of %IG, NE-WX, and NE-WY were substantially higher in active SLE compared to inactive SLE. The percentage of IG was the white blood cell inflammatory parameter that correlated the best with ESR (r_s_=0.56, p<0.0001), PCT (r_s_=0.51, p<0.0001), and CRP (r_s_=0.40, p=0.001). Regarding SLE disease activity, ESR was the best correlated with disease activity measured by SLEDAI-2K (r_s_=0.69, p<0.0001) and PGA (r_s_=0.69, p<0.0001), followed by PCT (SLEDAI-2K r_s_=0.62, p<0.0001; PGA r_s_=0.68, p<0.0001), %IG (SLEDAI-2K r_s_=0.53, p<0.0001; PGA r_s_=0.61, p<0.0001), and CRP (SLEDAI-2K r_s_=0.49, p<0.0001; PGA r_s_=0.60, p<0.0001). Next, we assessed the performance of white blood cell inflammatory parameters and inflammatory markers to differentiate the active and inactive SLE status. ESR demonstrated the best performance (AUC 0.95, 95 CI 0.89, 1.00), followed by %IG (AUC 0.91, 95% CI 0.83, 0.98), CRP (AUC 0.86, 0.77, 0.95), PCT (AUC 0.85, 95% CI 0.74, 0.96).

**Conclusion:** The percentage of IG was the best white blood cell inflammatory parameter to evaluate disease activity in children with SLE. There was a moderate to strong correlation among %IG, ESR, PCT, CRP, and disease activity assessed by SLEDAI-2K and PGA. The IG percentage can complement the inflammatory markers, specifically ESR, PCT, and CRP, in monitoring disease activity in SLE. Implementing the percentage of IG into clinical practice might be useful for monitoring disease activity in SLE.

**Patient Consent**

Not applicable (there are no patient data)

**Disclosure of Interest**

B. Lerkvaleekul Grant / Research Support with: The study was funded by grants from Sysmex Asia Pacific. Interpretations and results are entirely the responsibility of the authors., N. Sirachainan: None Declared, D. Songdej: None Declared, K. Paisooksantivatana: None Declared, P. Chinudomwong: None Declared, S. Vilaiyuk: None Declared

### Systemic lupus erythematosus and antiphospholipid syndrome

#### PT19 Rare turner syndrome and lupus coexistence with insights from DNA methylation patterns

##### Gülşah Kavrul Kayaalp^1^, Desiré Casares-Marfil^2^, Sezgin Şahin^3^, Özgür Kasapçopur^3^, Betül Sözeri^4^, Nuray Aktay Ayaz^1^, Amr H. Sawalha^5^

###### ^1^Division of Pediatric Rheumatology, Department of Pediatrics, Istanbul University, Istanbul, Türkiye; ^2^Division of Rheumatology, Department of Pediatrics, University of Pittsburgh, Pittsburgh, United States; ^3^Division of Pediatric Rheumatology, Department of Pediatrics, Istanbul University-Cerrahpaşa; ^4^Division of Pediatric Rheumatology, Department of Pediatrics, University of Health Sciences, Umraniye Research and Training Hospital, Istanbul, Türkiye; ^5^Departments of Pediatrics, Medicine, and Immunology & Lupus Center of Excellence, University of Pittsburgh, Pittsburgh, United States

####### **Correspondence:** Gülşah Kavrul Kayaalp

*Pediatric Rheumatology 2024*, **22(2):**PReS24-ABS-1289

**Introduction:** Systemic lupus erythematosus (SLE or lupus) is a complex autoimmune disease affecting multiple organs. Although the etiology and pathogenesis have not been fully elucidated, the influence of X chromosome dosage has been suggested. Besides the higher prevalence of lupus in women, individuals with Klinefelter syndrome (47, XXY) exhibit a heightened susceptibility compared to other men. Additionally, the occurrence of SLE alongside Turner syndrome is exceedingly rare.

**Objectives:** In this study, we aim to present a rare case of a patient diagnosed with mosaic Turner syndrome and subsequently presenting with juvenile-onset SLE, analyze DNA methylation patterns in this patient and compare with age-matched female SLE controls.

**Methods:** The study included the patient diagnosed with juvenile-onset SLE and Turner syndrome, as well as four juvenile-onset SLE patients matched for age, sex, ethnicity, and treatment. All patients fulfilled the 1997 revised ACR classification criteria for SLE. DNA was isolated from peripheral blood mononuclear cells and genome-wide DNA methylation patterns were determined using the Illumina Infinium MethylationEPIC v2.0 BeadChip array. Beta values were adjusted by cell type composition differences, and adjusted beta values were used to assess differences of DNA methylation levels of the SLE patient with Turner syndrome and control SLE patients.

**Results: Case:** A 9-year-old female patient presented to the pediatric clinic due to short stature. Upon examination, short neck, short 4th metacarpals, cubitus valgus, and widely spaced nipples were observed. Karyotype analysis revealed a chromosomal pattern of 45,X/46,X,i(X)(q10), leading to the diagnosis of mosaic Turner syndrome. The patient presented to the pediatric rheumatology clinic at the age of 11 with joint pain, malar and discoid rash, photosensitivity, oral ulcers, and a positive ANA test with a titer of 1:160, and diagnosed with juvenile-onset SLE.

Methylation analysis: DNA methylation patterns were analyzed in this patient and compared with age-matched female SLE controls, revealing higher methylation levels in interferon-regulated genes previously shown to be hypomethylated in SLE (p-value = 0.018). Four hundred CpG regions showing the greatest differences in methylation levels between SLE patients with and without Turner syndrome were investigated. Enrichment analysis of the genes annotated with these regions revealed significant enrichment in neurological pathways.

**Conclusion:** These data provide a mechanistic link between a gene-dose effect from the X-chromosome and the lupus-defining epigenotype. We hypothesize that the attenuated demethylation in interferon-regulated genes might provide a protective effect explaining the rarity of SLE in Turner syndrome. Further research is needed to determine whether *MECP2* or other X chromosome genes are involved in this conclusion.

**Date of birth:** Octobre 27

**Patient Consent**

Yes, I received consent

**Disclosure of Interest**

None Declared

### Systemic lupus erythematosus and antiphospholipid syndrome

#### PT20 Evaluation of neonatal lupus syndrome and babies born to mothers with lupus: a multicentre study

##### Esra Esen^1^, Sümeyra Özdemir^1^, Ayşenur P. Kısaarslan^1^, Mustafa Çakan^2^, Gülşah Doğrusadık^3^, Burcu Bozkaya^4^, Özge Baba^5^, Seher Şener^6^, Selcan Demir^7^, Nihal Şahin^8^, Nihal Karaçayır^9^, Betül Sözeri^3^, Mukaddes Kalyoncu^5^, Yelda Bilginer^6^, Sevcan Bakkaloğlu^9^, Hakan Poyrazoğlu^1^

###### ^1^Paediatric Rheumatology, Erciyes University, Kayseri; ^2^Paediatric Rheumatology, Zeynep Kamil Women’s and Children’s Diseases Training and Research Hospital; ^3^Paediatric Rheumatology, University of Health Sciences Ümraniye Training and Research Hospital, İstanbul; ^4^Paediatric Rheumatology, Samsun Training and Research Hospital, Samsun; ^5^Paediatric Rheumatology, Karadeniz Technical University , Trabzon; ^6^Paediatric Rheumatology, Hacettepe University , Ankara; ^7^Paediatric Rheumatology, Eskisehir Osmangazi University , Eskişehir; ^8^Paediatric Rheumatology, Kocaeli University , Kocaeli; ^9^Paediatric Rheumatology, Gazi University, Ankara, Türkiye

####### **Correspondence:** Esra Esen

*Pediatric Rheumatology 2024*, **22(2):**PReS24-ABS-1814

**Introduction:** Neonatal lupus erythematosus (NLE) is a rare and acquired autoimmune disease caused by maternal autoantibodies crossing the placenta. Autoantibodies related to NLE are anti-SSA/Ro, anti-SSB/La and anti-RNP antibodies.

**Objectives:** The aim is to evaluate the national data of NLE.To compare neonatal lupus syndrome and babies born to mothers with lupus who do not fulfil the diagnostic criteria for neonatal lupus syndrome.

**Methods:** In our study, we collected data on infants diagnosed with neonatal lupus syndrome and infants born to mothers diagnosed with SLE or SS who did not meet the diagnostic criteria for NLE.Neonatal lupus syndrome was defined as infants who were positive for at least one of the maternally transmitted anti-SSA, anti-SSB and anti-RNP antibodies and had systemic findings. Infants with negative antibodies and no systemic involvement were defined as baby born to a mother with lupus who did not develop neonatal lupus syndrome.Clinical and laboratory characteristics of the mothers and infants and the treatments they received were analysed.

**Results:** A total of 38 patients from 9 centres were included in the study.Of these, 18 had a diagnosis of NLE and 20 babies born to a mother with lupus. There were 37 patients whose mothers had a diagnosis of rheumatic disease at birth.In one patient, fetal hydrops was detected during pregnancy and the mother was diagnosed with Sjögren’s syndrome.The infants with neonatal lupus syndrome had mean gestational age of 37+2 (± 2.1) weeks and mean birth weight of 2899.71(± 476.237) grams.Anti-SSA was positive in 11(61.1%), anti-SSB in 5(27.8%) and anti-RNP in 2(11.1%) of the babies with neonatal lupus syndrome. System involvement in patients with NLE was cardiac in 9(50%), skin in 7(38.9%), haematological in 8(44.4%), hepatobiliary in 4(22.2%) and neurological in 2(11.1%).Treatment (medical/surgical) was required in 9 patients.Fetal bradycardia was noted in 6 of 9 patients with cardiac involvement in the prenatal period.In 4 of these patients, a pacemaker was implanted in the postnatal period because of complete AV block.The babies born to mothers with lupus who did not fulfil the diagnostic criteria for neonatal lupus syndrome had mean gestational age of 38(29-39) weeks and mean birth weight of 3150 (900-3700) grams.Five mothers were positive for anti-SSA antibodies.Anatomical abnormalities were observed on routine echocardiography in 9 asymptomatic babies born to mothers with lupus. Patent foramen ovale was observed in 7 patients and the anatomical defects resolved during follow-up. One baby born to a mother with lupus underwent PDA closure surgery. There were 2 patients with transient transaminase elevation. One patient had thrombocytopenia and neurological findings (intracranial haemorrhage) were observed in this patient. No baby born to a mother with lupus had cutaneous findings. In logistic regression analysis, maternal anti-SSA positivity [OR: 36.000 (95% CI: 3.692-351.002), p=0.002] and maternal anti-SSB positivity [OR: 18.286 (95% CI: 1.907-175.347, p=0.012] increased the development of neonatal lupus syndrome.

**Conclusion:** In our study, cardiac involvement was most common in patients with NLE, followed by haematological, skin, hepatobiliary and neurological involvement.Maternal anti-SSA or anti-SSB positivity was found to increase the risk of developing NLE.

**Date of birth:** août 27, Y

**Patient Consent**

Yes, I received consent

**Disclosure of Interest**

None Declared

### Autoinflammatory diseases

#### PT21 Understanding the mechanisms of hematologic and immunologic abnormalities by analyzing bone marrow of DADA2 patients

##### Francesca Schena^1^, Michela Lupia^2^, Roberta Caorsi^1^, Federica Barzaghi^3^, Mariacarla Giarratana^2^, Anna Corcione^1^, Federica Penco^1^, Paola Terranova^2^, Alessandro Aiuti^4^, Alessandra Mortellaro^4^, Alessia Cafaro^5^, Giuliana Cangemi^5^, Alice Grossi^6^, Elisabetta Traggiai^7^, Isabella Ceccherini^6^, Maurizio Miano^2^, Marco Gattorno^1^, Carlo Dufour^2^

###### ^1^UOC Reumatologia e Malattie Autoinfiammatorie; ^2^Haematology Unit, IRCCS Istituto Giannina Gaslini, Genoa; ^3^Pediatric Immunohematology and Bone Marrow Transplantation Unit; ^4^San Raffaele Telethon Institute for Gene Therapy, IRCCS San Raffaele Scientific Institute, Milan; ^5^Chromatography and Mass Spectrometry Section, Central Laboratory of Analysis; ^6^Genetic Unit, IRCCS Istituto Giannina Gaslini, Genoa, Italy; ^7^NIBR Biologics Center (NBC), Novartis Institutes for BioMedical Research, Basel, Switzerland

####### **Correspondence:** Francesca Schena

*Pediatric Rheumatology 2024*, **22(2):**PReS24-ABS-1320

**Introduction:** Deficiency of adenosine deaminase 2 (DADA2) is a monogenic autoinflammatory syndrome caused by biallelic mutations in the ADA2 gene. It is an autoinflammatory disease characterized by early-onset inflammatory vasculopathy, strokes and immunodeficiency. Haematologic manifestations occur early with hypogammaglobulinemia, lymphopenia, pure red cell aplasia or pancytopenia. The diagnosis of DADA2 is confirmed by decreased enzymatic activity of ADA2 and genetic testing. Treatment can be challenging. TNFαinhibitors are helpful to control inflammatory symptoms. Hematopoietic stem cell transplant may be needed to treat refractory cytopenia.

**Objectives:** The aim of this study is to characterize the mechanisms of bone marrow damage in DADA2 patients.

**Methods:** BM samples from DADA2 patients and healthy donors (HD) were analyzed for different in-vitro assays.

Fresh bone marrow mononuclear cells (BMMNCs) were cultured in semisolid media to perform the colony forming unit assay (CFU). Assays were performed in presence of anti-TNFα, human recombinant ADA2 or eltrombopag. After 14 days CFU colonies were scored. Pro-inflammatory cytokines in the BM plasma were measured by flow cytometry bead array.

Apoptosis assay was performed ex vivo and after stimulation with ADA2 on HD and patients BMMNCs.

**Results:** DADA2 patients show a defect of proliferation and maturation of progenitors compared to healthy donor (HD). Our data show that the addition of anti-TNFa to cultures stimulates significantly the growth of myeloid bone marrow progenitors in patients with severe bone marrow damage. The recombinant ADA2 also stimulates the growth of erythroid bone marrow progenitor cells. A slight stimulatory effect was observed in the presence of Eltrombopag.

TNFα marrow plasma levels were significantly higher in DADA2 patients compared to HD. No differences were observed in BM plasma IFNγ levels.

**Conclusion:** Our study shows that the BM of DADA2 patients is characterized by an inflammatory milieu. This may play a role in the pathophysiology of DADA2.

Preliminary data suggest that DADA2 B cells show a greater apoptosis than normal cells.

To better understand the mechanisms of BM damage and to develop new, alternative therapeutic approaches, further studies are needed

**Patient Consent**

Yes, I received consent

**Disclosure of Interest**

None Declared

### Autoinflammatory diseases

#### PT22 Interferon-gamma hyperresponsiveness may explain why FMF patients do not develop MAS

##### Ozge Basaran^1^, Erdal Sag^1,2^, Elif Arslanoglu Aydın^3^, Busra Aydin^4^, Kubra N. Tasdemir^4^, Elif Celikel^5^, Yagmur Bayındır^1^, Semanur Özdel^3^, Yelda Bilginer^1^, Alexei A. Grom^6^, Seza Ozen^1,4^

###### ^1^Pediatric Rheumatology, Hacettepe University; ^2^Pediatric Rheumatology Unit, Translational Medicine Laboratories, Hacettepe University; ^3^Pediatric Rheumatology, University of Health Sciences, Etlik City Hospital; ^4^Pediatric Rheumatology Unit, Translational Medicine Laboratories, Hacettepe University; ^5^Pediatric Rheumatology, University of Health Sciences, Ankara City Hospital, Ankara, Türkiye; ^6^Pediatric Rheumatology, Cincinnati Children’s Hospital Medical Center, University of Cincinnati, Ohio, United States

####### **Correspondence:** Ozge Basaran

*Pediatric Rheumatology 2024*, **22(2):**PReS24-ABS-1152

**Introduction:** Macrophage activation syndrome (MAS) is a life-threatening complication of systemic Juvenile Idiopathic Arthritis caused by excessive activation and proliferation of T cells and macrophages leading to a cytokine storm. IFN-ɣ and IL-18 have emerged as critical cytokines. Additionally, the hyperresponsiveness of monocytes and macrophages to IFN-ɣ may be an important factor that further amplifies MAS-associated hyperinflammation. This feature may also explain the strong association between SJIA and MAS. Familial Mediterranean Fever (FMF), an autosomal recessive disorder, typically presents with recurrent episodes of fever due to mutations in the MEFV gene. Despite the intense inflammatory response observed in FMF, the occurrence of MAS in these patients remains notably low.

**Objectives:** In this study, we aimed to compare in vitro the responsiveness of PBMCs to IFN-ɣ between SJIA/MAS and FMF patients.

**Methods:** Five patients diagnosed with MAS associated with systemic juvenile idiopathic arthritis (sJIA) and five FMF patients were enrolled. Peripheral blood mononuclear cells (PBMCs) were collected at the beginning and after the resolution of MAS or FMF flare. Four healthy individuals were enrolled as controls. PBMCs were in vitro stimulated with IFN-ɣ for 45 minutes. The levels of IFN-induced chemokine (C-X-C) motif ligand 9(CXCL9), CXCL10 and interleukin (IL) 18 levels in the supernatants were assessed before after the simulation via cytometric bead array.

**Results:** At baseline, in vitro cultured PBMCs from MAS patients were producing higher levels of CXCL9 compared to the samples from FMF patients obtained at the time of a flare. These differences were further increased after the stimulation with IFN-ɣ. Although the IFN-ɣ stimulation did mildly upregulate production of IL-18 in MAS (but not FMF patients), the overall production of IL-18 was comparable to healthy controls. This was in a sharp contrast to highly increased levels of total IL18 in the serum of MAS patients measured at the time of sample collection.

**Conclusion:** The study findings suggest that the exaggerated responsiveness to IFN-ɣ distinguishes SJIA/MAS patients from the patients in FMF flare. This feature may explain low rates of MAS in FMF. The dissociation between relatively low production of IL-18 by PBMCs in vitro and strikingly high serum levels in MAS/SJIA patients in vivo, suggests that this cytokine may be produced by predominantly non-hematopoietic cells. This study provides insights into the immunological mechanisms underlying MAS and FMF, paving the way for future investigations in this area.

**Date of birth:** 01.08.1981

**Patient Consent**

Yes, I received consent

**Disclosure of Interest**

None Declared

**References**

1. Schulert GS, Grom AA. Pathogenesis of macrophage activation syndrome and potential for cytokine- directed therapies. Annu Rev Med. 2015;66:145-59.
2. Ozen S. Update in familial Mediterranean fever. Curr Opin Rheumatol. 2021;33(5):398-402.
3. Bracaglia C, de Graaf K, Pires Marafon D, Guilhot F, Ferlin W, Prencipe G, et al. Elevated circulating levels of interferon-gamma and interferon-gamma-induced chemokines characterize patients with macrophage activation syndrome complicating systemic juvenile idiopathic arthritis. Ann Rheum Dis. 2017;76(1):166-72.

### Autoinflammatory diseases

#### PT23 Transcriptomic landscape of familial Mediterranean fever; a translational study

##### Erdal Sag^1,2^, Gozde Imren^3^, Ozge Basaran^1^, Busra Aydin^2^, Nur K. Tasdemir^2^, Dilara Unal^1^, Hulya Emreol^1^, Beren Karaosmanoglu^3^, Yelda Bilginer^1^, Ekim Z. Taskiran^3^, Seza Ozen^1, 2^

###### ^1^Department of Pediatric Rheumatology; ^2^Translational Medicine Laboratories, Pediatric Rheumatology Unit; ^3^Department of Medical Genetics, Hacettepe University, Ankara, Türkiye

####### **Correspondence:** Erdal Sag

*Pediatric Rheumatology 2024*, **22(2):**PReS24-ABS-1426

**Introduction:** Familial Mediterranean Fever (FMF) is the most common monogenic autoinflammatory disease, characterized by recurrent attacks of inflammation. Although it is an autosomal recessive disease, some patients develop disease phenotype even with only 1 MEFV mutations. Furthermore, about 3-5% of the patients do not respond adequately to colchicine treatment.

**Objectives:** We investigated the transcriptomic profile of FMF patients with different phenotypes and genotypes to figure out the possible differential signatures and diagnostic pathways.

**Methods:** RNAs derived from peripheral blood mononuclear cells (PBMCs) of 20 FMF patients, all meeting Eurofever criteria and on colchicine treatment (colchicine resistant with homozygous exon 10 mutations n=5; colchicine responsive with homozygous exon 10 mutations n=5; compound heterozygous with VOUS n=5; heterozygous exon 10 mutations n=5) were studied. Samples from 10 healthy controls (heterozygous MEFV carriers without clinical features n=5; healthy none MEFV mutation carriers n=5) were included as the healthy group. RNA-sequencing was performed on Illumina NextSeq550 with Qiaseq library kits. CLC software was used for data analysis. We first compared the transcriptomic profiles between patients expressing the FMF phenotype and healthy individuals. Later on, we analyzed the differences between FMF patients with heterozygous mutations and the ones without FMF phenotype but are heterozygous carriers.

**Results:** When we compared FMF patients with healthy individuals, we encountered 157 differently expressed genes overall. Among those genes, 104 of them were down-regulated in FMF patients while 53 of them were upregulated. We then analyzed the pathways. Interestingly, genes related with inflammatory response, IFN type 2 pathway, adaptive immune system pathway, IL-10 and IL-6 were downregulated. On the other hand, genes related with ubiquitination, apoptosis, heat shock proteins, IL-23, tubulin proteins, FOS-B and LAT pathway were upregulated. We than compared the differences between FMF carriers with the FMF phenotype to those without any features (healthy). We have seen a gradual difference with a similar signature showing these proteins might be useful for differentiating patients from healthy carriers. Among those genes SPP1 (Osteopontin) has been the most powerful one to help to discriminate the FMF patients.

**Conclusion:** The immune system pathways seem exhausted in FMF patients, which might explain the downregulation of these pathways and upregulation of LAT, involved in exhaustion. Another important gene discriminating colchicine resistant group was FOS-B (together with Rho-GTPase) which is also an important proinflammatory transcription factor. Osteopontin (SPP1) and FOS-B might be an important biomarker to discriminate the FMF phenotype requiring treatment. We are continuing further analysis and confirmation studies.

**Acknowledgement**

First two authors contributed equally.

This study was supported by Scientific and Technological Research Council of Turkey (TUBITAK) under the Grant Number 120C127 as part of 2247-A National Outstanding Researchers Program. The authors thank to TUBITAK for their supports.

**Patient Consent**

Yes, I received consent

**Disclosure of Interest**

None Declared

### Autoinflammatory diseases

#### PT24 Personalized medicine for systemic autoinflammatory diseases: the European multicenter persaids project

##### Marco Gattorno^1^, Paolo Uva^1^, Camilla Speziani^1^, Sabrina Fühner^2^, Helmut Wittkowski^2^, Yulia Rodina^3^, Gabriel Vignolle^4^, Andrea Petretto^1^, Ezgi Deniz Batu^5^, Seher Şener^5^, Fuat Akal^5^, Juantxo Arostegui^6^, Sergio Decherchi^3^, Dirk Föll^2^, Klemens Vierlinger^4^, Isabella Ceccherini^1^, Seza Ozen^5^

###### ^1^IRCCS Istituto Giannina Gaslini, Genoa, Italy; ^2^University of Münster, Münster, Germany; ^3^Fondazione Istituto Italiano di Tecnologia, Genoa, Italy; ^4^Austrian Institute of Technology Gmbh, Wien, Austria; ^5^Hacettepe University Faculty of Medicine, Ankara, Türkiye; ^6^Fundacio clinic per a la recerca biomedica, Barcelona, Spain

####### **Correspondence:** Marco Gattorno

*Pediatric Rheumatology 2024*, **22(2):**PReS24-ABS-1466

**Introduction:** Systemic autoinflammatory diseases (SAIDs) are a rapidly growing number of rare conditions with monogenic or polygenic/multifactorial etiology, which cause deregulation of mechanisms controlling innate immune responses. While specific monogenic SAIDs may benefit from personalized medicine approaches, “undefined” SAIDs (uSAIDs) are still lacking molecular testing and specific treatments.

**Objectives:** To improve the classification, diagnosis and prognosis of uSAIDs and to support the discovery of personalized therapies through the development of multi-omics signatures. To develop tools for SAID diagnosis and management in clinical practice.

**Methods:** We have undertaken a project aimed at analyzing available data and producing new data from a total of 200 SAIDs and uSAIDs, by integrating structured clinical data, multi-omics approaches (genomics, transcriptomics, proteomics, metabolomics, lipidomics, epigenomics, immunomics and inflammatory panel) and Artificial Intelligence technologies. Both supervised and unsupervised learning algorithms have been used to build models.

**Results:** Clinical and omics data have been collected and harmonized through a uniform semantic schema implemented in dedicated instance of the MOLGENIS database. Biological samples (DNA, RNA and sera) from patients recruited from three clinical centers were used by five centers to generate data from genomics, transcriptomics, proteomics, metabolomics, lipidomics, epigenomics, immunomics and OLINK inflammation panel. Preliminary results using hierarchical clustering followed by differential expression analysis and supervised Machine Learning approaches on individual omics showed relevant stratification between different SAIDs and uSAIDs and highlighted the presence of differentially expressed disease-associated markers that can provide insight into which biological processes or pathways differ between patient groups.

**Conclusion:** Omics-based clustering of yet unclassified SAIDs, already feasible using single omics datasets, will further increase in definition in the multi-omics analysis phase where integrated datasets can reveal novel associations between biological markers and disease phenotypes of relevance in clinical decision-making.

**Patient Consent**

Not applicable (there are no patient data)

**Disclosure of Interest**

None Declared

### Autoinflammatory diseases

#### PT25 Genetically defined systemic autoinflammatory diseases in pediatric patients with Behçet’s disease – a single center experience

##### Shaoling Zheng^1^, Xuechan Huang^1^, Yukai Huang^1^, Pui Yuen Lee^2^, Qing Zhou^3^, Tianwang Li^1^

###### ^1^Department of Rheumatology and Immunology, Guangdong Second Provincial General Hospital, Guangzhou, China; ^2^Allergy andRheumatoid Immunity, Boston Children Hospital, Boston, United States; ^3^Life Sciences Institute, Zhejiang University, Hangzhou, China

####### **Correspondence:** Shaoling Zheng

*Pediatric Rheumatology 2024*, **22(2):**PReS24-ABS-1083

**Introduction:** Behçet’s syndrome (BS) is a rare systemic inflammatory conditionwhich can cause a variable vasculitis, affecting both arteries of differentsizes and veins. Juvenile cases with age of onsetbefore 16 years of age account for around 2.7–17.6% of reported cases. Some of this variation relates to whether studies define juvenilecases as disease onset or diagnosis before 16 years of age. Because of the heterogeneity of presentation, the differential is wideand. Some of them will be diagnosis systemic autoinflammatory diseases by genetically defined such as HA20, RELA, GATA2, periodic fever, aphthous stomatitis, pharyngitis, and cervical adenitis(PFAPA), cyclical neutropenia and autoinflammatory mimics.

**Objectives:** The aim of this study is to characterize genetically defined SAID in a cohort of children with BD.

**Methods:** We studied 31 cases of pediatric BD treated in our center from 2019 to 2024. All cases fulfilled the 2015 classification criteria for paediatric BD [1]. Whole exome sequencing was performed to identify potential genetic causes of disease. Clinical characteristics of cases with or without genetically defined SAID were compared.

**Results:** We performed whole exome sequencing on 31 patients with pediatric BD and identified 7 cases with genetic findings associated with SAID (SAID+ group). Six patients possessed pathogenic or likely pathogenic germline variants in RELA, TNFAIP3 (encoding A20), and GATA2, compatible with the diagnoses of RELA haploinsufficiency (3 cases), haploinsufficiency of A20 (HA20; 2 cases), and GATA2 deficiency (1 case), respectively. One patient was found to have trisomy 8.

Three of the 7 cases exhibited parentally inherited variants (2 case with RELA haploinsufficiency and 1 case with HA20) while the remainder possessed de novo variants. Compared to pediatric BD patients without genetically determined SAID (SAID- group; n = 24), the SAID+ group exhibited earlier disease onset (3.55±3.33 vs. 7.38±4.26, P=0.034) and greater prevalence of recurrent fever episodes, intestinal involvement, and hematologic abnormalities. Oral ulceration affected all patients and was the initial symptom in the majority of cases in both groups. Laboratory studies revealed higher levels of C-reactive protein (44.92±36.08 vs. 7.47±8.58, P=0.0035), erythrocyte sedimentation rate (65.57±23.03 vs. 28.47±23.45, P=0.0023), and multiple cytokines including IL-8, IL-6, IL-10, and IL-2. Corticosteroid treatment was commonly utilized in both groups while the use of biologic DMARDs was more common in the SAID+ group (85.71% vs. 33.33% in the SAID- group, P=0.028). SAID+ patients were treated with adalimumab (3 cases), etanercept (3 cases), tocilizumab (1 case), and Infliximab (2 case).

**Conclusion:** Genetic evaluation of pediatric-onset BD is important as genetically defined SAID account for a substantial proportion of cases. Pediatric BD patients with SAID display greater levels of systemic inflammation and require more aggressive immunosuppressive therapy.

**Date of birth:** novembre 1

**Patient Consent**

Yes, I received consent

**Disclosure of Interest**

None Declared

**Reference**

1. Koné-Paut I, Fahrad Shahram, Martha Darce-Bello, et al. Consensus classification criteria for paediatricBehçet’s disease from a prospective observational cohort: PEDBD. Ann Rheum Dis 2015;0:1–7.

### Autoinflammatory diseases

#### PT26 Deficiency of adenosine deaminase 2: a large national multicenter cohort

##### Zeynep Balik^1^, Umit Gul^2^, Rabia Miray Kisla Ekinci^3^, Eray Tunce^4^, Eda Kayhan^5^, Aydan Yekeduz Bulbul^5^, Elif Celikel^6^, Aysenur Dogru^7^, Bahar Demirbas^8^, Seher Sener^9^, Hatice Adiguzel Dundar^10^, Ozge Baba^11^, Sezgin Sahin^2^, Halide Ozge Basaran^1^, Ezgi Deniz Batu^1^, Mukaddes Kalyoncu^11^, Aysenur Pac Kisaarslan^5^, Banu Celikel Acar^6^, Nuray Aktay Ayaz^7^, Betul Sozeri^4^, Yelda Bilginer^1^, Ozgur Kasapcopur^2^, Seza Ozen^1^

###### ^1^Department of Pediatric Rheumatology, Hacettepe University, Ankara; ^2^Department of Pediatric Rheumatology, Istanbul University Cerrahpasa Medical Faculty, Istanbul; ^3^Department of Pediatric Rheumatology, Cukurova University, Adana; ^4^Department of Pediatric Rheumatology, Umraniye Training and Research Hospital, Istanbul; ^5^Department of Pediatric Rheumatology, Erciyes University, Kayseri; ^6^Department of Pediatric Rheumatology, Ankara Bilkent City Hospital, Ankara; ^7^Department of Pediatric Rheumatology, Istanbul University Istanbul Medical Faculty, Istanbul; ^8^Department of Pediatric Rheumatology, Etlik City Hospital, Ankara; ^9^Department of Pediatric Rheumatology, Adana City Hospital, Adana; ^10^Department of Pediatric Rheumatology, Tepecik Training and Research Hospital, Izmir; ^11^Department of Pediatric Rheumatology, Karadeniz Technical University, Trabzon, Türkiye

####### **Correspondence:** Zeynep Balik

*Pediatric Rheumatology 2024*, **22(2):**PReS24-ABS-1716

**Introduction:** The deficiency of adenosine deaminase 2 (DADA2) is an autosomal recessively inherited autoinflammatory disease. Disease manifestations could be separated into three major phenotypes: inflammatory/vascular, immune dysregulation, and hematologic, however, most patients present with significant overlap between these three phenotype groups. Treatment responses may vary between groups.

**Objectives:** This study aimed to evaluate the clinical findings, treatments and treatment responses of DADA2 patients in a large national multicenter cohort.

**Methods:** Until 2024, the patients diagnosed with DADA2 who were followed up in 11 different centers in Turkey were included in the study. The patients’ demographics, clinical findings, treatments and treatment responses were assessed.

**Results:** A total of 89 patients (46 female, 43 male) were included in the study. The median age at symptom onset was 6.5 years, diagnosis was 10.3 years. The median duration from symptom onset to diagnosis was 3.0 years. Sixty-one (68.5%) patients had a history of parental consanguinity and 38 (42.7%) patients had a family history of DADA2. Two (2.2%) patients were asymptomatic, they were diagnosed after screening for DADA2 in their siblings. The most common clinical findings were skin(n=71, 79.8%), constitutional (n=69,77.5%) and musculoskeletal (n=61, 68.5%) findings, respectively. Hematologic abnormalities were found in 43 (48.3%) patients while immunologic abnormalities were found in 38 (42.7%) patients. Eighteen patients (20.2%) had systemic vasculitis. Forty-eight (53.9%) patients were initially diagnosed with other rheumatological diseases, and the most common diagnoses were polyarteritis nodosa and familial Mediterranean fever. The median PVAS at the diagnosis was 4. The most commonly used treatments before diagnosis were steroids (47.2%), colchicine (33.7%), methotrexate (12.4%) and azathioprine (12.4%) while after diagnosis almost all received anti-tumor necrosis factor (anti-TNF), and some were also on steroids (30.3%) and colchicine (13.5%). By anti-TNF therapy were constitutional and gastrointestinal symptoms improved. Hematopoietic stem cell transplantation was performed in one patient with the diagnosis of Diamand Blackfan anemia before the diagnosis of DADA2, two patients due to severe hematological involvement and one patient with severe neutropenia. At the last follow-up, 63 (71.6%) patients were in complete remission while 17 (19.3%) patients were in partial remission. Five (5.6%) patients died due to resistant disease.

**Conclusion:** The clinical findings of DADA2 are very variable and delay in diagnosis is very common. Childhood polyarteritis nodosa patients should also be evaluated for DADA2 in this respect. Early diagnosis and effective treatment may improve disease course.

**Date of birth::** septembre

**Patient Consent**

Yes, I received consent

**Disclosure of Interest**

None Declared

**References**

1. Li GM, Han X, Wu Y, Wang W, Tang HX, Lu MP, et al. A Cohort Study on Deficiency of ADA2 from China. J Clin Immunol. 2023;43(4):835-45
2. Barron KS, Aksentijevich I, Deuitch NT, Stone DL, Hoffmann P, Videgar-Laird R, et al. The Spectrum of the Deficiency of Adenosine Deaminase 2: An Observational Analysis of a 60 Patient Cohort. Front Immunol. 2021;12:811473.

### Autoinflammatory diseases

#### PT27 Genes involved into interleukin or interferon signalling pathways are differentially expressed in COVID19 associated multisystem inflammatory syndrome in children at single cell level

##### Barbara Jenko Bizjan^1,2^, Robert Sket^1,2^, Tine Tesovnik^1,2^, Jernej Kovac^1,2^, Marusa Debeljak^1,2^, Mojca Z. Avramovic^2,3^, Nina Emersic^2,3^, Klementina Crepinsek^1^, Barbara Slapnik^1^, Blaz Vrhovsek^1^, Tadej Avcin^2,3^

###### ^1^Clinical Institute of Special Laboratory Diagnostics, University Children’s Hospital, UMC; ^2^Faculty of Medicine, University of Ljubljana; ^3^Department of Allergology, Rheumatology and Clinical Immunology, University Children’s Hospital, UMC, Ljubljana, Slovenia

####### **Correspondence:** Barbara Jenko Bizjan

*Pediatric Rheumatology 2024*, **22(2):**PReS24-ABS-1132

**Introduction:** Children infected with severe acute respiratory syndrome coronavirus 2 (SARS-CoV-2) usually present minimal symptoms or are asymptomatic. Nevertheless, a subset of children 2-6 weeks after the initial SARS-CoV-2 infection develops a postinfectious SARS-CoV-2-related multisystem inflammatory syndrome in (MIS-C).

**Objectives:** The purpose of our project is to characterize the complexity of cell populations and capture cellular heterogeneity to uncover the regulatory networks that are disrupted during MIS-C flare with simultaneous profiling of gene expression and open chromatin regions. Moreover, we are exploring gene regulatory interactions driving inflammation in MIS-C.

**Methods:** Samples of peripheral blood mononuclear cells from patients with MIS-C diagnosed at the University Children’s Hospital, University Medical Center Ljubljana, were collected during the initial presentation before any treatment and at 6-12 months in remission. To enable simultaneous profiling of epigenomic landscape and gene expression from the same nuclei, we are using Chromium Next GEM Single Cell Multiome ATAC + Gene Expression kit from 10X Genomics.

**Results:** We included 20 MIS-C patients with MIS-C from whom we collected paired blood samples during the initial presentation before treatment and at 6-12 months in remission. Samples in two time points from 10 patients, with the most viable cell count prior cryopreservation were included into single cell multiomic experiment. After low cells quality filtering and doublets removal, we had 89154 cells, where 51312 were at the time point of 6-12 months in remission and 37842 at initial disease presentation before any treatment. On average 5131 cells (SD=1626,2) per patient remained in analyses at the time of remission state and 3784,2 per patient at initial disease presentation time point. Genes that were differentially expressed (DE) in at least 6 patients per cell type were selected for gene set enrichment analysis. The higest number of DE genes was found in CD4 Naive cells (n=70), while CD4 TCM and CD8 Naive had 33 DE genes, B naive 15, NK cells 12, and finally CD8 TEM 4 and B intermediate 3. Interferon Alpha/Beta Signaling, Interferon Gamma Signaling, nterleukin-6 Family Signaling and Interleukin-4 And Interleukin-13 Signaling were significantelly enriched most cell types with DE signature. Interferon Alpha/Beta Signaling and Interferon Gamma Signaling were significantelly enriched in B naive (p.adjust=0.001), CD4 naive (p.adjust<0.001), CD4 TCM (p.adjust=0.005) as well as in NK cells (p.adjust=0.03), while Interleukin-6 Family Signaling and Interleukin-4 And Interleukin-13 Signaling were significantly enriched in CD8 Naive (p.adjust<0.05), CD4 TCM (p.adjust<0.001) and in CD4 Naive (p.adjust<0.001) cells.

**Conclusion:** The results of this project are expected to enlighten the underlying pathophysiology of MIS-C flare and thus support clinical decision on more targeted treatment. The identified disrupted networks during MIS-C flare could lead the way to establish an early diagnosis and improve long-term outcome, including prevention of myocardial and neuropsychological impairment.

**Date of birth:** décembre 3

**Patient Consent**

Yes, I received consent

**Disclosure of Interest**

None Declared

### Autoinflammatory diseases

#### PT28 Cluster definition in pediatric SAPHO syndrome according to skin manifestations: results from a national multicentric study

##### Caterina Matucci-Cerinic^1,2^ on behalf of REUMAPED, Anna Attico^3^, Clara Malattia^1, 2^, Alessandro Consolaro^1, 2^, Silvia Rosina^2^, Luciana Breda^4^, Saverio La Bella^4^, Marco Cattalini^5^, Francesca Ricci^5^, Giovanni Conti^6^, Adele Civino^7^, Letizia Baldini^8^, Francesco Licciardi^8^, Antonella Insalaco^9^, Francesco La Torre^10^, Serena Pastore^11^, Giovanni Filocamo^12^, Gisella Beatrice Beretta^12^, Francesca Biscaro^13^, Angela Miniaci^14^, Gabriele Simonini^15, 16^, Edoardo Marrani^15^, Angela Pistorio^17^, Stefano Volpi^1, 2^, Roberta Caorsi^1, 2^, Nicolino Ruperto^18^, Gianmaria Viglizzo^19^, Marco Gattorno^2^ and REUMAPED

###### ^1^Department of Neurosciences, Rehabilitation, Ophthalmology, Genetics, Maternal andChild Health, University of Genoa; ^2^Rheumatology and Autoinflammatory diseases, IRCCS Istituto Giannina Gaslini, Genoa; ^3^Dept of Medical sciences, Pediatric Section, University Hospital of Ferrara, ferrara; ^4^Dipartimento di Pediatria, Università “G. D’Annunzio” di Chieti-Pescara, Chieti; ^5^Clinica Pediatrica, Università degli Studi di Brescia e ASST Spedali Civili, ERN RITA Centre, Brescia; ^6^AOU Policlinico G Martino, Unità di Nefrologia e Reumatologia pediatrica, Messina; ^7^Reumatologia e Immunologia pediatrica, Ospedale Vito Fazzi, Lecce; ^8^Regina Margherita Children’s Hospital, Divisione di Immunoreumatologia Pediatrica , Torino; ^9^IRCCS Ospedale Pediatrico Bambino Gesù, Division of Rheumatology, ERN-RITA center, Roma; ^10^Servizio di Reumatologia Pediatrica, UOC Pediatria, Ospedale Pediatrico Giovanni XXIII, Università di Bari, Bari; ^11^Istituto Salute Materno-infantile, “IRCCS Burlo Garofolo”, Trieste; ^12^Fondazione IRCCS Cà Granda Ospedale Maggiore Policlinico, Reumatologia Pediatrica, Milano; ^13^Ospedale civile di Treviso, Treviso; ^14^Pediatric Unit, IRCCS Azienda Ospedaliero-Universitaria di Bologna, Bologna; ^15^Meyer Children’s Hospital IRCCS, Rheumatology Unit, ERN ReCONNET center; ^16^University of Florence, NEUROFARBA department, Firenze; ^17^Biostatistics Unit; ^18^Gaslini Trial Center; ^19^Dermatology Department, IRCCS Istituto Giannina Gaslini, Genoa, Italy

####### **Correspondence:** Caterina Matucci-Cerinic

*Pediatric Rheumatology 2024*, **22(2):**PReS24-ABS-1067

**Introduction:** Pediatric SAPHO(pSAPHO)is a rare autoinflammatory disease characterized by chronic non-bacterial osteitis (CNO) asssociateed to different dermatologic manifestations (Palmo-plantar pustulosis (PPP),psoriasis vulgaris(PV), acne,hidradenitis suppurativa (HS),pyoderma gangrenosum(PG)).pSAPHO has been clustered upon skin manifestations in an acne-HS-PG group(male prevalence,disease onset in pubertal age with skin manifestations refractory to multiple treatments)and a PPP-PV group(female prevalence,disease onset in prepubertal age with osteoarticular manifestations)(1)

**Objectives:** to confirm the presence of different pSAPHO clusters according to skin manifestations in an italian multicenter cohort, compared to CNO patients without skin disease

**Methods:** The data of pSAPHO enrolled in the Eurofever Registry,were retrospectively analysed.Patients were divided into an acne-HS-PG and a PPP-PV group and were compared to a CNO group without skin manifestations.Comparison of frequencies between groups was performed by the means of the Chi-square test or by the Fischer’s Exact test

**Results:** 54 pSAPHO with skin manifestations(35 acne-HS-PG and 19 PPP-PV)were enrolled and compared to 167 CNO.In the Acne-HS-PG group,82.9% were males,with disease onset in pubertal age (median 13.3 years)characterised by skin manifestations,with the appearance of osteoarticular symptoms in the following year.In the PPP-PV group, 84.2% were females,with disease onset with osteoarticular manifestations in prepubertal years(median 10.2 years),followed by skin manifestations in the following year. In CNO,there were no gender differences,median age at disease onset 9.5 years.A significant difference was found between the 3 groups in terms of sex(p< 0.0001), and age at disease onset (p<0.0001).All patients showed long bones involvement,while an axial pattern(sterno-clavicular-spine-sacroiliacs)was more frequent in the acne-HS-PG (p<0.01), and a mandibular involvement in the PPP-PV (p<0.01).In CNO,NSAIDs(84%,p<0.001),methotrexate(20%), and salazopyrin(30%) were more frequently used,48.5%required bisphosphonates(BP), and 16%biologic therapy.In PPP-PV 50% required BP,20% MTX and 30% salazopyrin,in 60% biologic therapy was added for CNO control, while skin responded well to topical therapies or DMARDs.In acne-HS-PG,steroid use was prevalent(54.3%,p<0.001)and biological therapy was needed in 80% to control refractory skin disease (combination Adalimumab/MTX more efficacious)

**Conclusion:** The data confirm,in a large multi-center national study, the presence of 2 disease clusters of pSAPHO based on skin manifestations:an acne-HS-PG group(male predominance with pubertal disease onset with skin manifestations refractory to treatments), and a PPP-PV group(female predominance with prepubertal disease onset with osteoarticular manifestations), which was similar to CNO group. These clusters need a different therapeutic approach according to skin manifestations, and highlight the need of a new disease classification.

**Patient Consent**

Yes, I received consent

**Disclosure of Interest**

None Declared

**Reference**

1. Semin Arthritis Rheum. 2023:63:152277

### Autoinflammatory diseases

#### PT29 Cross-national biologic treatment guidelines for familial Mediterranean fever: a comparative analysis

##### Muserref Kasap Cuceoglu^1^, Tanja Hinze^2^, Helmut Wittkowski^3^, Fatih Haslak^4^, Anna Mamutova^5^, Nimet Oner^6^, Yonatan Butbul Aviel^7^, Veronique Hentgen^8^

###### ^1^Department of Pediatric Rheumatology, Hacettepe University, Ankara, Türkiye; ^2^Clinic of Paediatric and Adolescent Rheumatology, St.-Josef-Stift Sendenhorst, Northwest German Center for Rheumatology, Sendenhorst; ^3^Department of Pediatric Rheumatology and Immunology, University Hospital Münster, Münster, Germany; ^4^Department of Pediatric Rheumatology, İstanbul University-Cerrahpasa, İstanbul, Türkiye; ^5^Department of Pediatric Rheumatology, National Medical Research Centre for Children’s Health, Moscow, Russian Federation; ^6^Department of Pediatric Rheumatology, Department of Pediatrics, Ankara City Hospital, University of Health Sciences, Ankara, Türkiye; ^7^Pediatric Rheumatology Service, Ruth Rappaport Children’s Hospital, Haifa, Israel; ^8^Department of General Paediatrics, French Reference centre for Autoinflammatory diseases and amyloidosis (CEREMAIA), Versailles Hospital, Le Chesnay, France

####### **Correspondence:** Fatih Haslak

*Pediatric Rheumatology 2024*, **22(2):**PReS24-ABS-1339

**Introduction:** Familial Mediterranean fever (FMF), the most common genetic autoinflammatory disease (AID), exhibits a distinct geographic distribution predominantly in populations of the eastern Mediterranean.(1) While colchicine stands as the cornerstone therapy for FMF, 5-10% of patients exhibits colchicine resistance or intolerance, necessitating alternative treatments such as biological disease-modifying anti-rheumatic drugs (bDMARDs), particularly interleukin-1 (IL-1) inhibitors.(2)

**Objectives:** This study explores the accessibility and reimbursement policies concerning IL-1 inhibitors for the treatment of FMF across various countries. Additionally, it aims to compare the official national guidelines utilized in different regions globally.

**Methods:** This study is a part of the Clinical Practice Strategies (CLiPS) project that aims to collect real life clinical practice strategies from worldwide physicians dealing different medical topics with the Juvenile Inflammatory Rheumatism - Clinical Practice Strategies (JIR-CliPS) Action falling under its umbrella. The JIR-CliPS is a project that aims to collect real life clinical practice strategies from worldwide physicians dealing with five different medical topics. One of the five topics is the use of bDMARDs for treatment of AID.

We initially collected data on access to bDMARDs and reimbursement policies by country through a questionnaire distributed to adults and pediatric rheumatologists, as well as experts who completed the CLiPS questionnaire. Subsequently, we contacted experts from countries that did not respond to the questionnaire, asking the same questions via email.

In addition, we conducted a comprehensive search for national recommendations on FMF treatment using the PubMed/MEDLINE database and Google search, without language restrictions. Finally, we conducted an analysis of standardized recommendations, focusing on variations between countries in defining colchicine resistance, colchicine intolerance, colchicine-related adverse events, indications for biologic use, and the availability of IL-1 inhibitors.

**Results:** We examined 31 countries and obtained national guidelines for 11 of them. Among these, articles were published in PubMed for five countries (Brazil, Egypt, Iran, Germany, France). Two guidelines (from The Netherlands and Turkey) were received via email from experts who completed the CLiPS questionnaire. For the remaining four countries (England, Israel, Belgium, Spain), we accessed the guidelines in the respective national language using Google search. Furthermore, representatives from five countries (Italy, Portugal, Slovenia, Switzerland, Armenia) indicated the utilization of the EULAR 2016 guideline (3).

Anakinra treatment was available for colchicine- resistant FMF treatment in 21 countries, with 14 of them covered by National Health Insurance reimbursement. Canakinumab treatment was accessible in 17 countries, with reimbursement coverage in 14 of them.

Reimbursement conditions varied across many countries, with seven countries (Belgium, England, Spain, Lithuania, Romania, Brazil, Turkey) having additional indications for restricted prescription of biological drugs in FMF patients.

**Conclusion:** This study underscores the heterogeneous landscape of prescribing IL-1 inhibitors for FMF globally, reflecting variations in national guidelines, medication availability, and reimbursement policies. Standardized guidelines incorporating clear definitions of treatment indications and harmonized access to therapeutic options are warranted to optimize patient care and outcomes.

**Date of birth:** 5/21/1995 1

**Patient Consent**

Not applicable (there are no patient data)

**Disclosure of Interest**

None Declared

**References**

1. Russo RAG, Brogan PA. Monogenic autoinflammatory diseases. Rheumatol (United Kingdom). 2014;53(11):1927–39.
2. Ehlers L, Rolfes E, Lieber M, Müller D, Lainka E, Gohar F, et al. Treat-to-target strategies for the management of familial Mediterranean Fever in children. Pediatr Rheumatol. 2023;21(1).
3. Ozen S, Demirkaya E, Erer B, Livneh A, Ben-Chetrit E, Giancane G, et al. EULAR recommendations for the management of familial Mediterranean fever. Ann Rheum Dis. 2016;75(4):644–51.

### Autoinflammatory diseases

#### PT30 Long-term follow-up and outcomes of colchicine discontinuation in patients with familial Mediterranean fever

##### Selen Duygu Arık, Özlem Akgün, Gülşah Kavrul Kayaalp, Nuray Aktay Ayaz

###### Division of Pediatric Rheumatology, Istanbul University Istanbul Faculty of Medicine, Istanbul, Türkiye

####### **Correspondence:** Selen Duygu Arık

*Pediatric Rheumatology 2024*, **22(2):**PReS24-ABS-1728

**Introduction:** Familial Mediterranean Fever (FMF) is a monogenic autoinflammatory disease with frequent attacks in Turkey. Colchicine is the main drug to prevent attacks in FMF patients and the main goal is to reduce clinical and subclinical inflammation to prevent long-term complications.

**Objectives:** The aim of this study is to present the characteristics and follow-up results of children diagnosed with FMF, with at least one genetic mutation in the MEFV gene, who were treated with colchicine for a period of time during the course of the disease and who were discontinued colchicine during follow-up

**Methods:** The medical files of all children with FMF who were followed up regularly every 3-6 months at one Pediatric Rheumatology Clinic center were retrospectively analyzed. Colchicine was discontinued by pediatric rheumatologists if there was no family history of amyloidosis, no attacks during 6 months of colchicine treatment, no homozygous or compound heterozygous exon 10 MEFV mutations, and no proteinuria. Patients who did not need colchicine after colchicine discontinuation were classified as Group 1 and patients who restarted colchicine treatment were classified as group 2. Both groups 1 and 2 were compared in terms of demographic characteristics, clinical findings, and MEFV mutations.

**Results:** In a cohort of 2300 FMF patients, 100 patients who discontinued colchicine treatment were included. Of the entire cohort, 64% were male. Median age at symptom onset and median age at diagnosis were 5.4 (min-max: 0.2-13.7) and 6.7 (min-max: 1.2-16.1) years, respectively. The median disease duration was 9.1 (min-max: 1.6-18.7) years. The median follow-up period after colchicine discontinuation was 5.1 (min-max: 0.2-18.4) years. Colchicine treatment was discontinued by physician’s decision in 63 patients and voluntarily in 37 patients. During follow-up, 78 patients continued to follow-up without medication, while 22 patients restarted colchicine treatment due to clinical attacks and/or elevated acute phase reactants and proteinuria. There was no significant difference between the two groups in terms of baseline clinical characteristics and the appropriateness of colchicine initiation and discontinuation dose according to age. Also there was no significant difference between the two groups in terms of age at symptom onset, age at diagnosis, duration of colchicine treatment and duration of disease follow-up. The duration of the attack-free period before discontinuation of colchicine treatment was analyzed in groups 1 and 2 and no significant difference was found (p:0.77). When the clinical findings of 20 patients who restarted colchicine treatment were evaluated, it was observed that 55% of the patients had abdominal pain and arthralgia, 50% had fever and 5% had chest pain before restarting. However, no patient had arthritis or erysipelas-like erythema.

When the two groups were compared for the presence of MEFV mutations, no significant difference was found between the two groups in terms of carrying exon 10 mutations. The MEFV mutation carriage observed in the total cohort consisted of heterozygous mutations of M694V (40%), E148Q (30%) and V726A (16%) alleles in order of frequency.

**Conclusion:** This study is one of the few long-term studies evaluating the discontinuation of colchicine treatment in childhood AAA patients. It is known that there are no proven data and/or guidelines on the discontinuation of colchicine treatment in childhood. Long-term and large studies on this subject are needed in the following years.

**Date of birth:** octobre 30

**Patient Consent**

Not applicable (there are no patient data)

**Disclosure of Interest**

None Declared

### JIA (oligo, poly, psoriatic)

#### PT31 The discrepancy between physician global assessment and the parent/patient well-being evaluation may have two souls

##### Francesca Ridella^1^, Roberta Naddei^2^, Francesca Bovis^3^, Martina Maccario^4^, Marco Burrone^1,4^, Silvia M. Orsi^1^, Silvia Rosina^4^, Valentina Natoli^1,4^, Caterina Matucci-Cerinic^1,4^, Clara Malagon^5^, Amparo Ibanez^6^, Olga Arguedas^7^, Cristina Herrera Mora^8^, Nicola Ruperto^4,9^, Angelo Ravelli^10^, Marco Gattorno^4^, Alessandro Consolaro^1,4^

###### ^1^Dipartimento di Neuroscienze, Riabilitazione, Oftalmologia, Genetica e Scienze Materno Infantili (DINOGMI), University of Genova, Genova; ^2^Department of Traslational Medical Sciences, University of Naples, Napoli; ^3^Department of Health Sciences (DISSAL), Section of Biostatistics, University of Genova; ^4^UOC Rheumatology and Autoinflammatory diseases, IRCCS Giannina Gaslini Institute, Genova, Italy; ^5^Clinica Infantil Colsubsidio, Hospital Universitario Simon Bolivar, Bogota’, Colombia; ^6^Rheumatology, National Institute Salud del Nino, Lima, Peru; ^7^Immunology, Hospital Nacional De Ninos Dr.Carlos Saenz Herrera, San Jose’, Costa Rica; ^8^Reumatologia, Hospital de Ninos Roberto Gilbert Elizalde, Guayaquil, Ecuador; ^9^Paediatric Rheumatology INternational Trials Organisation (PRINTO); ^10^Direzione Scientifica, IRCCS Giannina Gaslini Institute, Genova, Italy

####### **Correspondence:** Francesca Ridella

*Pediatric Rheumatology 2024*, **22(2):**PReS24-ABS-1745

**Introduction:** Parent/child-reported outcomes (PCROs) are crucial for assessing the perception of rheumatic disease course and therapeutic effectiveness, particularly in Juvenile Idiopathic Arthritis (JIA). The parent/patient global evaluation or well-being visual analogue scale (WB-VAS) is widely used in JIA patients, yet discrepancies between physician and parent/patient evaluations have been described.

**Objectives:** The study aimed to evaluate which variables might determine the differences between physician assessment of inactive disease and the parent/patient perception of well-being.

**Methods:** We analyzed data from a large multinational sample of JIA patients from the Epidemiology Treatment and Outcome of Childhood Arthritis (EPOCA) study. Demographic factors, socioeconomic status, education level, JIA subtype, and various patient-reported outcomes (PROs) were examined. We only included patients with a Physician Global Assessment (PGA) score of 0 and we divided them in two groups, according to their answer to the WB-VAS score: group 1 reported WB-VAS ≤ 1 and group 2 reported WB-VAS >1. We then checked with factor analysis if items that were different between the two groups could be reduced into a smaller set of factors.

**Results:** Results from 3537 patients revealed two groups based on WB-VAS score: 2862 with WB-VAS ≤ 1 and 675 with WB-VAS > 1. Socioeconomic status and education did not significantly differ between the groups: lower socioeconomic status was noted in 17.6% and 18.1% of families, intermediate in 70.5% and 71%, and higher in 11.9% and 10.8%, respectively; education levels were similarly distributed. No significant difference was observed in JIA subtype distribution. Patients with higher WB-VAS scores were slightly older at disease onset (6.4 and 5.6 years); they reported more pain (VAS pain mean 2.4 vs 0.3 in group 1), morning stiffness (42.4% vs 8% of patients in group 1), joint inflammation (1.4 vs 0.2 number of proxy-assessed active joints in group 1), medication side effects (42.9% vs 21% of patients in group 1), and had greater functional impairment (3 vs 0.5 mean score in the JIA Assessment of Functionality Score) and lower health-related quality of life (6.4 vs1.6 mean score in the JIA Quality of Life Score). Exploratory factor analysis identified two key factors: “Joint symptoms” (factor 1) and “Disease burden” (factor 2). The variable with the strongest association to the underlying latent variable factor 1, is VAS pain. Factor 1 also correlated with joint inflammation signs, morning stiffness, and disease activity, while factor 2 correlated with functional ability, Health-Related Quality of Life (HRQoL), medication side effects, and school problems.

**Conclusion:** In conclusion, the study highlighted factors contributing to discrepancies between physician assessments and parent/patient perceptions of well-being in JIA. Our data suggests that the disagreement can be due either to a different perception of the disease status, or to the fact that WB-VAS measures a broader domain than disease activity

**Patient Consent**

Yes, I received consent

**Disclosure of Interest**

None Declared

### JIA (oligo, poly, psoriatic)

#### PT32 Seeking for predictors of inactive disease in juvenile idiopathic arthritis with artificial intelligence

##### Ana Isabel Rebollo-Gimenez^1,2^, Davide Cangelosi^3^, Francesca Ridella^4^, Silvia Maria Orsi^4^, Elena Aldera^4^, Valentina Natoli^4^, Silvia Rosina^1^, Esperanza Naredo^5^, Angelo Ravelli^6^

###### ^1^Pediatric and Rheumatology Clinic, IRCCS Istituto Giannina Gaslini, Genoa, Italy; ^2^Rheumatology, Universidad Autónoma de Madrid. Hospital Beata María Ana, Madrid, Spain; ^3^Clinical bioinformatics unit; ^4^Università Degli Studi Di Genova, Dipartimento di Neuroscienze, Riabilitazione, Oftalmologia, Genetica e Scienze Materno-Infantili, IRCCS Istituto Giannina Gaslini, Genoa, Italy; ^5^Department of Rheumatology, Bone and Joint Research Unit, Hospital Universitario Fundación Jiménez Díaz, IIS Fundación Jiménez Díaz, and Universidad Autónoma de Madrid, Madrid, Spain; ^6^Scientific Direction, IRCCS Istituto Giannina Gaslini, Genoa, Italy

####### **Correspondence:** Ana Isabel Rebollo-Gimenez

*Pediatric Rheumatology 2024*, **22(2):**PReS24-ABS-1722

**Introduction:** The achievement of inactive disease (ID) is the goal of contemporary treatment strategies in all patients with juvenile idiopathic arthritis (JIA). Complete disease quiescence is regarded as the ideal therapeutic goal as its attainment helps to minimize pain and disability associated with active disease, prevent articular and extraarticular damage, and improve the quality of life of children and their families. Artificial intelligence is a powerful tool that is well suited to identify the variables that are of foremost relevance to predict a desired outcome.

**Objectives:** The aim of our study is to seek for the disease parameters that have the best ability to predict the achievement of the state of ID at 24 months using artificial intelligence methods.

**Methods:** The clinical charts of all consecutive patients with JIA by ILAR criteria who were first seen at study centre within 6 months after disease onset between 2007 and 2019 and had follow-up visits at 6, 12, 18, and 24 months after initial evaluation were reviewed retrospectively. Demographic parameters, clinical features (including physician-centred and parent-reported outcomes), laboratory tests and prescribed therapy were collected at all visits and entered into an excel dataset. ID was defined by 2004 Wallace criteria. The Last Observation Carried Forward (LOCF) and posteriorly Baseline Observation Carried Forward (BOCF) methods were used to impute missing values. Patients in whom imputation could not be performed with these computational techniques were excluded. Data recorded at baseline and at 0-, 6- and 12-month follow-up visits were included in the analysis of prediction of achievement of ID at 24 months. The study cohort was divided into two subsets:a training set (50%) and a test set (50%). Multivariate time series forecasting was applied to the longitudinal data through the Light Gradient Boosting Machine (LightGBM) method using the Scikit-learn with mlforecast to train and test the predictive model. Features were ranked by importance to determine their relative impact on achievement of ID. Hyperparameter tuning was carried out using the optuna package. Matthews correlation coefficient (MCC) was used as a metric to assess model performance. The entire analysis procedure was implemented in Python.

**Results:** Of the 449 patients who had their initial evaluation within 6 months after disease onset at study centre in the study period, 294 had longitudinal assessments available. 147 patients were randomly allocated to the training set and 147 to the test set. For each follow up visit 74 features were collected. By applying the mlforecast method coupled with LightGBM algorithm, we obtained 71% of MCC in the training set and 69% in the test set, which indicated a strong ability of our artificial intelligence model to predict the state of ID from the identified clinical variables at 0, 6 and 12 months. The best predictor of ID was the physician’s global assessment of disease activity (PhGA), followed by the age at onset and count of active joints. Other relevant features were acute phase reactants (erythrocyte sedimentation rate and C-reactive protein).

**Conclusion:** The PhGA over time was the strongest predictor of achievement of ID at 24 months, which highlights the importance of its regular scoring and its key role in guiding treatment decisions. The prominent importance of physician-centred outcome measures (PhGA and active joint count) and acute phase reactants in ID prediction supports the use of the Juvenile Arthritis Disease Activity Score (JADAS), which includes all these variables, in deciding treatment adaptations within treat-to-target strategies aimed to attain complete disease control.

**Patient Consent**

Not applicable (there are no patient data)

**Disclosure of Interest**

A. I. Rebollo-Gimenez: None Declared, D. Cangelosi: None Declared, F. Ridella: None Declared, S. M. Orsi: None Declared, E. Aldera: None Declared, V. Natoli: None Declared, S. Rosina: None Declared, E. Naredo: None Declared, A. Ravelli Speaker Bureau with: AbbVie, Novartis, Pfizer, Reckitt Benckiser, Alexion, Galapagos, Sobi

### JIA (oligo, poly, psoriatic)

#### PT33 Adalimumab and anti-drug antibodies in a cohort of children with juvenile idiopathic arthritis

##### Giusyda Tarantino^1^, Raffaele Simeoli^2^, Emiliano Marasco^1^, Rebecca Nicolai^1^, Angela Aquilani^1^, Bianca Maria Goffredo^2^, Silvia Magni Manzoni^1^, Fabrizio De Benedetti^1^

###### ^1^Rheumatology Division; ^2^Metabolic Disease, IRCCS Bambino Gesù Children’s Hospital, Rome, Italy

####### **Correspondence:** Giusyda Tarantino

*Pediatric Rheumatology 2024*, **22(2):**PReS24-ABS-1598

**Introduction:** Adalimumab (ADA), a fully humanized antibody against tumor necrosis factor (TNF)-α, has revolutionized treatment of patients with juvenile idiopathic arthritis (JIA). Although most of these respond within the first weeks, a minority may show loss of response (LOR) after continued exposure. Many studies demonstrate the influence of anti-adalimumab antibodies (AAA) on serum drug concentrations and clinical outcome in adults. However, little information about AAA and LOR is available for children with JIA.

**Objectives:** Firstly, to describe demographic and clinical features in a single-center cohort of JIA patients treated with ADA, grouped according to frequency (1W vs 2W), to dosage of drug administration (20 vs 40 mg) and to disease activity (from inactive ID to high HDA). Also to assess ADA levels versus AAA titer and, finally, to investigate possible correlation between LOR and AAA.

**Methods:** Records of JIA patients on ADA treatment were retrospectively reviewed with focus on medical history and ELISA (enzyme-linked immunosorbent assay) ADA/AAA levels in a 3-years-period. Children with idiopathic uveitis and systemic JIA were excluded.

**Results:** From June 2019 to January 2024 we collected 446 ADA/AAA samples in 128 JIA patients treated with ADA (a median of 3.3/pt). Of them (65% females), 62 had ANA-positive oligoarthritis, 40 RF-negative polyarthritis, 13 HLA-B27 positive enthesitis-related arthritis and 10 psoriatic. The median age at disease onset was about 3.9 years. Half of study cohort was b-DMARDS naive at ADA start, while all children were on concomitant c-DMARDs (98% on Methotrexate). Chronic recurrent uveitis was the main reason for ADA starting, followed-by tenosynovitis, bowel inflammation and spine or hip active arthritis. The median disease duration at first sampling was 3.1 (IQ 1.3-6.4). About pharmacinetics, in the study group there was an inverse correlation, previously described for adults, between ADA and AAA. Among AAA positive (>50 AU/ml) patients, higher levels of body mass (BMI) and basal protein C reactivity (CRP) were detected. About pharmacodinamics, the concentration of ADA, at the same dosing time, resulted lower in children with moderate (MDA) and high (HDA) disease activity, stratified into oligo (c-JADAS10 >4) and polyarticular forms (cJADAS10 >8.5). Finally, in 65% of these last we collected very high AAA titer (105-1230 AU/mL).

**Conclusion:** Our preliminary “real life” data showed association between occurrence of AAA and lower ADA levels. The clinical response expressed as c-JADAS10 depends firstly on certain drug exposure. A targeted risk analysis about high AAA titers and LOR incidence is still pending at our center. Monitoring of drug immunogenicity should be implemented in daily practice and become subject of future modelling studies.

**Patient Consent**

Yes, I received consent

**Disclosure of Interest**

None Declared

**References**

1. A. Marino. Anti-adalimumab antibodies in a cohort of patients with juvenile idiopathic arthritis: incidence and clinical correlations. Clin Rheumatol. 2018 May;37:1407-1411.
2. J. B. Brunelli. Anti-adalimumab antibodies kinetics: an early guide for juvenile idiopathic arthritis (JIA) switching. Clin Rheumatol 2020 Feb;39:515-521.

### JIA (oligo, poly, psoriatic)

#### PT34 Assessment of grip strength, body composition and physical performance of patients with juvenile idiopathic arthritis: could sarcopenia be possible?

##### Asya Albayrak^1,2^, Nilay Arman^3^, Irem Donmez^4^, Yusuf Acıkgoz^4^, Kubra Ucar^3^, Asena Yekdaneh^1,5^, Figen Cakmak^6^, Nuray Aktay Ayaz^7^

###### ^1^Physiotherapy and Rehabilitation Doctorate Program, Istanbul University-Cerrahpasa Institute of Graduate Studies; ^2^Faculty of Health Sciences, Department of Physiotherapy and Rehabilitation , Istanbul Kent University; ^3^Faculty of Health Sciences, Department of Physiotherapy and Rehabilitation , Istanbul University-Cerrahpasa; ^4^Physiotherapy and Rehabilitation Master of Science Program, Istanbul University-Cerrahpasa Institute of Graduate Studies; ^5^Physiotherapy English Program, Fenerbahçe University Vocational School of Health Services; ^6^Department of Pediatric Rheumatology , Basaksehir Cam ve Sakura Hospital, ^7^Department of Pediatric Rheumatology, Istanbul University Istanbul Faculty of Medicine , Istanbul, Türkiye

####### **Correspondence:** Asya Albayrak

*Pediatric Rheumatology 2024*, **22(2):**PReS24-ABS-1513

**Introduction:** Sarcopenia is described by the loss of skeletal muscle mass and reduced muscle strength or physical performance (1). Historically, sarcopenia was primarily linked with older adults; however, a decrease in muscle mass, strength, and function associated with chronic diseases has also been observed in pediatric populations (2). Juvenile idiopathic arthritis (JIA) is the most common chronic rheumatic disease of childhood, which can cause a decrease in muscle strength and physical performance, and may be associated with sarcopenia (3).

**Objectives:** The aim of this study was to evaluate grip strength, body composition, and physical performance in adolescents with JIA to identify possible sarcopenia and to compare them with healthy controls.

**Methods:** Twenty-one adolescents with JIA and 16 healthy controls aged between 12 to 17 years old were included in the study. The grip strength of the patients was assessed with the hand-held dynamometer (Kinvent Physio K-Grip), in terms of peak force (PF), mean force (MF), mean RFD, time to peak force (TPF), and fatigue parameters. Grip measurements were repeated three times for both hands. The body composition was analyzed using the Bioelectrical Impedance Analysis Device (Tanita SC240MA), and for each participant, appendicular skeletal muscle mass (ASM)/weight and muscle-to-fat ratio (MTF) were calculated. The physical performance was evaluated through the 6 Minute Walk Test (6MWT) and walking speed.

**Results:** The mean age of the children and adolescents diagnosed with JIA and healty controls included in the study was 14.71±1.79 and 15.43±0.96 years, respectively. When compared with healthy controls, grip strength measured for three repetitions for both hands in adolescents with JIA was significantly lower in terms of PF, MF, and mean RFD (p<0.05), while there was no difference in terms of TPF and fatigue (p>0.05). When body composition and physical performance results were compared, ASM/weight and MTF ratios, 6MWT and walking speed of adolescents with JIA were significantly lower compared to healthy peers (p<0.05).

**Conclusion:** The results of this study indicate that grip strength, muscle mass, and physical performance are significantly affected in adolescents with JIA compared to their healthy peers. We believe that the observed decrease in grip strength, muscle mass, and physical performance in children with JIA may suggest the potential development of sarcopenia. Therefore, we recommend a detailed evaluation of these parameters in adolescents with JIA before planning physical activity and exercise programs. Given these findings, we believe that sarcopenia in adolescents with Juvenile Idiopathic Arthritis emerges not merely as a possibility but as a critical condition that necessitates proactive, targeted interventions to mitigate its progression and optimize health outcomes. This study was supported by TÜBİTAK 1001-Scientific and Technological Research Projects Support Program with project number 121E690.

**Date of birth::** 23.10.1995

**Patient Consent**

Yes, I received consent

**Disclosure of Interest**

None Declared

**References**

1. Ooi, P. H., Thompson‐Hodgetts, S., Pritchard‐Wiart, et al. Pediatric sarcopenia: a paradigm in the overall definition of malnutrition in children? Journal of Parenteral and Enteral Nutrition, 2020;44(3),407-418.
2. Ritz, A., Lurz, E., Berger, M. Sarcopenia in children with solid organ tumors: an instrumental era. Cells, 2022;11(8),1278.
3. Kuntze, G., Nesbitt, C., Whittaker, J. L., et al. Exercise therapy in juvenile idiopathic arthritis: a systematic review and meta-analysis. Archives of Physical Medicine and Rehabilitation, 2018;99(1),178-193.

### JIA (oligo, poly, psoriatic)

#### PT35 Efficacy and safety of ixekizumab in children with active juvenile psoriatic arthritis and enthesitis related arthritis (COSPIRIT-JIA): 16-week results of a multicentre, randomised, open-label study

##### Athimalaipet Ramanan^1^, Nicolino Ruperto^2^, Ivan Foeldvari^3^, Gabriel V. Cornejo^4^, Stuart Keller^5^, Rona Wang^5^, Joana Araújo^5^, Maja Hojnik^5^, Priyanka Sen^5^, Ketan Marulkar^5^, Pierre Quartier^6,7^

###### ^1^University of Bristol, Bristol, United Kingdom; ^2^IRCCS Istituto Giannina Gaslini, France, Italy; ^3^Hamburger Zentrum für Kinder-und Jugendrheumatologie, Hamburg, Germany; ^4^CREA, Hospital México Americano, Guadalajara , Mexico; ^5^Eli Lilly and Company, Indianapolis, United States; ^6^Université Paris-Cité; ^7^Pediatric Immunology-Haematology and Rheumatology Unit, Hôpital Necker-Enfants Malades, Paris, France

####### **Correspondence:** Athimalaipet Ramanan

*Pediatric Rheumatology 2024*, **22(2):**PReS24-ABS-1243

**Introduction:** Ixekizumab (IXE), an anti-interleukin-17A monoclonal antibody, has demonstrated efficacy and safety in adults with psoriatic arthritis (PsA) and axial spondyloarthritis (axSpA).

**Objectives:** To evaluate efficacy and safety of IXE in paediatric patients(pts) with active Juvenile psoriatic arthritis (JPsA) and enthesitis-related arthritis (ERA) through week (W) 16.

**Methods:** COSPIRIT-JIA is an on-going multicentre, randomised, open-label Phase 3 study of IXE, with adalimumab (ADA) as a reference arm in pts aged 2 - <18 with active JPsA/ERA. It is conducted in 3 periods: a 16W open-label treatment (OLT) period followed by open-label extension (OLE) period proceeding to W104, followed by open-label long term extension period (LTE) out to a total of 264W. Pts received either subcutaneous IXE or ADA based on weight during the OLT and OLE period (IXE every 4 weeks: 20mg (starting dose 40mg) for pts 10 - <25kg; 40mg (starting dose 80mg) for pts 25 - 50kg and 80mg (starting dose 160mg) for pts >50kg; and ADA every 2 weeks: 20mg for pts 10 - <30kg and 40mg for pts ≥30kg). At the end of OLE, all remaining ADA pts will be transitioned to IXE or discontinued. Primary endpoint: IXE-treated pt percentage meeting the JIA American College of Rheumatology (ACR) 30 response criteria at W-16.

**Results:** There were 101 pts (IXE=81, ADA=20) in the 16-W OLT period. At baseline: 44 (43.6%) females, mean age 13.1(±3.1) years, total Psoriasis Area and Severity Index score 4.4±3.0 and Leeds Enthesitis Index 2.1± 1.1. Pts were diagnosed with JPsA (31 [30.7%]) and ERA (70 [69.3%]). At the end of OLT, 72 (88.9%) of all IXE-treated pts achieved JIA ACR30. Response rates were similar across IXE-treated bio-naïve (54 [90.0%]) and bio-experienced (18 [85.7%]) pts and across ERA (48 [88.9%]) and JPsA (24 [88.9%]) categories. In the OLT period, 81.5% IXE-treated pts presented treatment-emergent adverse events (most were mild). Serious adverse events: 3.7% IXE-treated pts. There were no new safety signals.

**Conclusion:** In paediatric pts with JPsA and ERA, efficacy of IXE was demonstrated with an overall 88.9% JIA ACR30 response rate at W16. Safety findings were consistent with the known safety profile of IXE.

**Patient Consent**

Not applicable (there are no patient data)

**Disclosure of Interest**

None Declared

### JIA (oligo, poly, psoriatic)

#### PT36 Predictive ability of the JADAS10- and cjadas10-based disease activity states for psoriatic arthritis and enthesitis-related arthritis

##### Silvia M. Orsi^1^, Marco Burrone^1,2^, Francesca Ridella^1^, Ivan Foeldvari^3^, Ekaterina Alexeeva^4^, Nuray Aktay Ayaz^5^, Inmaculada Calvo Penades^6^, Ozgur Kasapcopur^7^, Kaur Sharonjeet^8^, Hermine I Brunner^9^, Angelo Ravelli^1,10^, Nicolino Ruperto^11^, Marco Gattorno^2^, Alessandro Consolaro^1,2^ and the Paediatric Rheumatology International Trials Organisation (PRINTO) and the Pediatric Rheumatology Collaborative Study Group

###### ^1^Dipartimento di Neuroscienze, Riabilitazione, Oftalmologia, Genetica e Scienze Materno-Infantili (DiNOGMI), Università degli Studi di Genova; ^2^Rheumatology and autoinflammatory diseases Unit, IRCCS Istituto Giannina Gaslini, Genova, Italy; ^3^Competence Centre for Uveitis and scleroderma in childhood and adolescence, Hamburg Centre for Pediatric and Adolescent Rheumatology, Hamburg, Germany; ^4^Rheumatology Department, State Institution Scientific Center of Children Health of Rams Moscow, Moscow, Russian Federation; ^5^Pediatric Rheumatology Department, Istanbul University Faculty of Medicine, Istanbul, Türkiye; ^6^Pediatric Rheumatology Unit, Hospital Universitario y Politécnico la Fe, Valencia, Spain; ^7^Pediatric Rheumatology, Istanbul Universitesi-Cerrahpasa, Istanbul, Türkiye; ^8^Novartis, Basel, Switzerland; ^9^Division of Rheumatology, Cincinnati Children’s Hospital Medical Center, Cincinnati, United States; ^10^Scientific Direction; ^11^UOSID Centro Trial, IRCCS Istituto Giannina Gaslini, Genova, Italy

####### **Correspondence:** Silvia M. Orsi

*Pediatric Rheumatology 2024*, **22(2):**PReS24-ABS-1345

**Introduction:** The measurement of disease activity level is of central importance in the evaluation of the patient with juvenile idiopathic arthritis (JIA) and the need to have cutoffs for all the arthritis categories is increasingly evident. The cutoff for Juvenile Arthritis Disease Activity Score (JADAS) and its clinical version excluding the acute phase reactant (cJADAS) in RF-polyarthritis and oligoarthritis were validated and are being used in clinical trials and routine practice. We have recently demonstrated on the large multinational dataset of patients enrolled in the Epidemiology, Treatment and Outcome of Childhood Arthritis (EPOCA) study that 2021 ACR JADAS10 and cJADAS10 cutoffs to define disease activity states showed good discriminative validity in juvenile psoriatic arthritis (PsA) and enthesitis-related arthritis (ERA) (oligoarthritis cutoffs) and in RF positive polyarthritis (polyarthritis cutoffs).

**Objectives:** Aim of this study is to assess the predictive ability of the JADAS10 and cJADAS10 disease state cutoffs to separate the states of inactive disease (ID), minimal disease activity (MiDA), moderate disease activity (MoDA), and high disease activity (HDA) in children with PsA and ERA.

**Methods:** 86 JIA children aged 2 to 18 years included in the clinical trial to assess efficacy and safety of secukinumab in ERA and PsA were considered. This phase 3 trial is a randomised, double-blind, placebo-controlled, treatment-withdrawal, in which biologic-naïve patients with active disease were treated with open-label subcutaneous secukinumab for up to 8 weeks and then switched to a randomised, double-blind, placebo-controlled, withdrawal period until week 104. We compared the frequency of patients in ACR clinically inactive disease (CID) and normal functional ability (i.e. with Childhood Health Assessment Questionnaire (CHAQ) = 0) at week 104 among those who achieved or did not achieve the states of ID and MiDA at week 12. We expected that patients who achieved ID or MiDA at week 12 had a higher probability of achieving ACR CID or normal physical function at week 104.

**Results:** Of the 74 patients with ERA and PsA at week 104, 28 (37.8%) met the definition of ACR CID and 42 (56.8%) had CHAQ = 0. Of the 28 patients in ACR CID at week 104, more than 60% of patients were in JADAS10 or cJADAS10 ID at week 12; whereas of the of the 46 patients who did not achieve ACR CID less than 22% were in JADAS10 or cJADAS10 ID at week 12 (p < 0.001 for both comparisons). Of the 42 patients with normal physical function at week 104, more than 47% of patients were in JADAS10 or cJADAS10 ID at week 12; whereas of the 32 patients who lacked normal physical function fewer than 19% were in JADAS 10 or cJADAS10 ID at week 12 (p < 0.01 for both comparisons). Among patients in ACR CID and with CHAQ = 0 at week 104, the 78,6% were in MiDA at week 12 according to both JADAS10 and cJADAS10 cutoffs; whereas of the patients who didn’t achieve ACR CID and who had CHAQ > 0 less than 59% were in JADAS10 and cJADAS10 MiDA at week 12.

**Conclusion:** Both the JADAS10 and cJADAS10 cutoffs to define disease activity states validated for oligoarthritis showed also a good predictive ability in PsA and ERA. These results confirm our prior indication that the available cutoffs might be used for these categories of JIA.

**Patient Consent**

Yes, I received consent

**Disclosure of Interest**

None Declared

### JIA (oligo, poly, psoriatic)

#### PT37 Associations of movement behaviours and health-related quality of life among patients with juvenile idiopathic arthritis: a compositional data analysis

##### Florian Milatz^1^, Lisa Voigt^2^, Jens Klotsche^1^, Tilmann Kallinich^1, 3^, Ralf Trauzeddel^4^, Daniel Windschall^5, 6^, Sandra Hansmann^7^, Nadja Baumeister^8^, Johannes-Peter Haas^8^, Moritz Klaas^9^, Hermann J. Girschick^9^, Joachim Peitz-Kornbrust^10^, Kirsten Minden^1, 3^

###### ^1^Deutsches Rheuma-Forschungszentrum Berlin, Berlin; ^2^University Medicine Greifswald, Greifswald; ^3^Charité - Universitätsmedizin Berlin; ^4^Helios Klinikum Berlin-Buch, Berlin; ^5^St. Josef-Stift Sendenhorst, Sendenhorst; ^6^Universität Halle-Wittenberg, Halle; ^7^University of Tuebingen, Tuebingen; ^8^German Centre for Paediatric and Adolescent Rheumatology, Garmisch-Partenkirchen; ^9^Vivantes Klinikum Friedrichshain, Berlin; ^10^Asklepios Kinderklinik Sankt Augustin, Sankt Augustin, Germany

####### **Correspondence:** Florian Milatz

*Pediatric Rheumatology 2024*, **22(2):**PReS24-ABS-1555

**Introduction:** Recent research suggests that the composition of movement behaviours within a day can have important implications for health across the lifespan [1]. Acknowledging the co-dependence and compositional nature of physical activity (PA) and sedentary behaviour (SB), the focus is therefore now on how these behaviours relate to each other rather than viewing them in isolation when investigating their associations with health outcomes.

**Objectives:** This study examines the association between time-use composition of movement behaviours and health-related quality of life (HRQoL) among young people with JIA using compositional data analysis (CoDA) [2].

**Methods:** Movement data of JIA patients aged 10 to 20 years recruited in the German multicentre ActiMON study were collected using a hip-worn ActiGraph accelerometer (Model wGT3X-BT) on seven consecutive days during waking hours (valid wear time ≥ 5 days incl. at least 1 weekend day with ≥ 8 hours each). Data collection and processing was done according to internationally recommended criteria [3] using validated cut points for classification into SB, light PA (LPA) and moderate to vigorous PA (MVPA). HRQoL was assessed using the Pediatric Quality of Life Inventory (PedsQL 4.0), while clinical data were collected within the National Paediatric Rheumatological Database (NPRD). CoDA was used to transform time spent in SB, LPA, and MVPA into isometric log-ratio pivot coordinates before examining their association with HRQoL in confounder-adjusted multiple linear regressions.

**Results:** 126 patients fulfilled wear time criteria (mean age 15.0 ± 2.1 years, female 67%, mean disease duration 8 ± 4 years, oligoarthritis 46%, cJADAS-10 2 ± 3 years, mean PedsQL total score 84 ± 12, valid wear days 6.6 ± 0.6, daily wear time 14.3 ± 2.0 hours). Patients spent on average 86% of wear time in SB, 8% in LPA and 6% in MVPA. The overall time-use composition was associated with PedsQL total score (*p*<0.001) after adjusting for age, gender, functional ability (CHAQ) and severity of depressive symptoms (PHQ-9). Specifically, more time in MVPA relative to LPA and SB was significantly associated with higher PedsQL total score (*p*=0.041). Based on the sample’s mean composition (86% SB, 8% LPA, and 6% MVPA) an increase in MVPA of 3-5% at the expense of time in LPA or SB would be associated with an increase in PedsQL total score of about 4 points in both males and females.

**Conclusion:** Young patients’ movement behaviour composition is related to their self-reported HRQoL. This might be improved in particular by reallocating time in favour of MVPA. Applying CoDA can provide the basis to determine the optimal composition of various movement behaviours for HRQoL enhancement in JIA patients’ everyday life.

ActiMON as part of the research network TARISMA was funded by the Federal Ministry of Education and Research (01EC1902F).

**Trial registration identifying number:** DRKS00022258

**Patient Consent**

Not applicable (there are no patient data)

**Disclosure of Interest**

None Declared

**References**

1. Rollo S, Antsygina O, Tremblay MS. The whole day matters: Understanding 24-hour movement guideline adherence and relationships with health indicators across the lifespan. J Sport Health Sci. 2020 Dec;9(6):493-510.
2. Dumuid D, et al. Compositional Data Analysis (CoDA) in Time-Use Epidemiology: What, Why, How. Int J Environ Res Public Health. 2020;17:2220.
3. Migueles JH, et al. Accelerometer data collection and processing criteria to assess physical activity and other outcomes: A systematic review and practical considerations. Sports Med. 2017;47:1821-45.

### JIA (oligo, poly, psoriatic)

#### PT38 Preliminary results of the ondansetron pre-medication trial in juvenile idiopathic arthritis (OPT-JIA): a pragmatic randomized controlled trial nested in the capri registry

##### Gaëlle Chédeville^1^, Heinrike Schmeling^2^, Jean-Philippe Proulx-Gauthier^3^, Michelle Batthish^4^, Jean Jacques De Bruycker^5^, Brian M. Feldman^6^, Roberta A. Berard^7^, Roxana Bolaria^8^, Amieleena Chhabra^9^, Lily SH Lim^10^, Adam M. Huber^11^, Matthew Berkowitz^12^, Thomas Loughin^12^, Jaime Guzman^13^ and CAPRI Registry Investigators

###### ^1^Pediatrics, McGill University, Montreal; ^2^Pediatrics, University of Calgary, Calgary; ^3^Pediatrics, Universite Laval , Quebec; ^4^Pediatrics, McMaster University, Hamilton; ^5^Pediatrics, Universite de Montreal, Montreal; ^6^Pediatrics, University of Toronto, Toronto; ^7^Pediatrics, Western University, London; ^8^Pediatrics, UBC, Victoria; ^9^Pediatrics, UBC, Penticton; ^10^Pediatrics, University of Manitoba, Winnipeg; ^11^Pediatrics, Dalhousie University, Halifax; ^12^Statistics, Simon Fraser University, Burnaby; ^13^Pediatrics, UBC, Vancouver, Canada

####### **Correspondence:** Jaime Guzman

*Pediatric Rheumatology 2024*, **22(2):**PReS24-ABS-1051

**Introduction:** In children with Juvenile Idiopathic Arthritis (JIA), methotrexate (MTX) side effects such as nausea, vomiting and abdominal discomfort can lead to conditioned MTX intolerance with severe anticipatory nausea/vomiting, and avoidance behaviours (in up to 50% of children). MTX intolerance may lead to decreased quality of life, poor adherence to optimal dosing, or to stopping MTX altogether.

**Objectives:** To evaluate if routine premedication with the anti-emetic ondansetron reduces MTX intolerance and increases the proportion of children with JIA able to continue MTX, when compared to as needed ondansetron use.

**Methods:** Children with JIA ages 4-16 years participating in the Canadian Alliance of Pediatric Rheumatology Investigators (CAPRI) Registry who were starting MTX for the first time were assigned, via stratified block randomization by Canadian region and patient weight, to one of two groups: 1) routine premedication with oral ondansetron from the very first dose of MTX (intervention), or 2) ondansetron used only after developing nausea/vomiting (control). Ondansetron was prescribed as 3 doses weekly starting one hour before administration of MTX (2mg if <15Kg, 4 mg if 15-30 Kg, or 8mg if >30Kg). The primary trial endpoint was the proportion of children who remain on MTX with no intolerance one year after starting MTX. We hypothesized the primary endpoint would be observed in ≥75% patients in the intervention group, compared to 50% in the control group. Intolerance was defined as a score of ≥6 points in the MTX Intolerance Severity Score, MISS. The COVID19 pandemic slowed recruitment and it was decided to conduct the planned interim analysis with data available as of October 6, 2023. Any patient who developed intolerance or discontinued MTX within a year of starting it was considered a failure, and one-year success rates were estimated with Kaplan Meier survival analysis.

**Results:** Sixty-one children were assessed for eligibility, of which 52 were randomized and 44 contributed to the Kaplan Meier analysis of the primary outcome. Participants randomized to intervention (n=22) or control (n=30) had comparable baseline demographic and clinical characteristics, despite the imbalance in number of participants due to use of stratified blocks. After a median follow-up of 45.5 weeks (intervention) and 39 weeks (controls), we observed 7 failures in the intervention group and 8 failures in the control group with 59% and 83% of participants still on MTX at last follow-up, respectively. The Kaplan-Meier estimate of success at one year was 57% for intervention and 59% for controls, for a risk ratio of 0.91 (99% CI: 0.39, 2.08). Based on computer simulations, the probability of observing results like this if our a priori hypothesized success rates (75% and 50%, respectively) are correct is very low, at 0.055. Forty-six adverse events were reported (mostly gastrointestinal, 17 in intervention and 29 in control groups, respectively), and only one was serious (infection with hospitalization in a patient not receiving ondansetron).

**Conclusion:** This interim analysis showed very little signal to suggest prophylactic ondansetron increases the proportion of patients remaining on MTX with no intolerance one year after starting it, relative to using ondansetron after nausea or vomiting develop. Based on these results, the Data and Safety Committee deemed enrolling additional patients was unlikely to change findings. It recommended stopping recruitment into the trial and completing follow-up of all subjects already enrolled.

Funded by a competitive research grant from The Arthritis Society, Canada.

**Trial registration identifying number**: clinicaltrials.gov NCT04169828

**Patient Consent**

Not applicable (there are no patient data)

**Disclosure of Interest**

None Declared

### JIA (oligo, poly, psoriatic)

#### PT39 Early systemic treatment may reduce the frequency for temporomandibular joint involvement in children with JIA

##### Martina Albertella^1^, Marco Burrone^1,2^, Silvia Maria Orsi^1^, Francesca Ridella^1^, Ana Isabel Rebollo-Giménez^2^, Alice Gamba^1^, Lorenzo Anfigeno^3^, Maria Beatrice Damasio^3^, Clara Malattia^1, 2^, Stefania Viola^2^, Marco Gattorno^2^, Angelo Ravelli^2,4^, Alessandro Consolaro^1,2^

###### ^1^Dipartimento di Neuroscienze, Riabilitazione, Oftalmologia, Genetica e Scienze Materno-Infantili (DiNOGMI), Università degli Studi di Genova; ^2^Rheumatology and Autoinflammatory Diseases Unit; ^3^Department of Radiology; ^4^Scientific Direction, IRCCS Istituto Giannina Gaslini, Genoa, Italy

####### **Correspondence:** Silvia M. Orsi

*Pediatric Rheumatology 2024*, **22(2):**PReS24-ABS-1483

**Introduction:** In Juvenile Idiopathic Arthritis (JIA), temporomandibular joint (TMJ) involvement still represents a major source of long-term damage and reduced life quality health. No specific risk factors have been identified for this disease manifestation and it is not clear if an early aggressive intervention may prevent the TMJ disease in children with JIA.

**Objectives:** To study the frequency of TMJ involvement among patients affected with JIA, together with the investigation of clinical characteristics associated and the impact of early JIA treatment on TMJ involvement.

**Methods:** We included in the study the clinical charts of consecutive patients with oligoarthritis and RF negative polyarthritis, visited in our center in the first six months from disease onset between January 2018 and February 2020 with at least 4 years of follow-up. Only patients who received the first treatment for arthritis at the study Unit were included. TMJ involvement was assessed by magnetic resonance imaging (MRI). We compared baseline disease characteristics between children with or without TMJ involvement and the therapeutic intervention done before TMJ involvement.

**Results:** A total of 67 patients (71.6 % females, median age at JIA onset 3.5 years) with a median disease duration of 4.3 years were included in the study. The JIA category distribution was: 64.2% oligoarticular arthritis, 35.8% polyarticular arthritis. In the first six months of disease course, 91.0% of children received intraarticular corticosteroid injections (IACIs), 58.2% methotrexate, 13.4% biologic DMARDs. TMJ involvement was radiologically identified in 31/67 patients (46.3%). No significant difference was observed in the frequency of TMJ involvement based on gender, age at onset, disease duration at first intervention, JIA category, number of active joints, ANA positivity, baseline CRP and ESR. Twenty children received only IACIs until the last follow up visit or until TMJ disease was diagnosed. Of these, 16 (80%) had TMJ involvement. Of the remaining 47 patients who were treated with conventional or biological DMARDs until the last follow up visit or until TMJ disease was diagnosed, 15 (32%) had TMJ involvement (Chi-squared test P < 0.001).

**Conclusion:** TMJ involvement was common in in the first 5 years of disease course in this cohort of JIA children. No clinical risk factor for TMJ was observed at disease onset. Early treatment with systemic medication seems to protect JIA patients from these potentially severe complications.

**Patient Consent**

Yes, I received consent

**Disclosure of Interest**

None Declared

### Juvenile dermatomyositis

#### PT40 Toward a treat-to-target strategy in juvenile dermatomyositis: seeking for suitable targets and optimal timing of their achievement

##### Silvia Rosina^1^, Ana I. Rebollo-Giménez^1^, Chiara Campone ^2^, Valentina Natoli^1,2^, Alessandro Consolaro^1,2^, Francesca Bovis^3^, Angelo Ravelli^4^, Angelo Ravelli^4^

###### ^1^UOC Reumatologia e Malattie Autoinfiammatorie , IRCCS Istituto Giannina Gaslini; ^2^Dipartimento di Neuroscienze, Riabilitazione, Oftalmologia, Genetica e Scienze Materno-Infantili (DiNOGMI); ^3^Dipartimento di Scienze della Salute, Sezione di Biostatistica , Università degli Studi di Genova; ^4^Direzione Scientifica , IRCCS Istituto Giannina Gaslini , Genova, Italy

####### **Correspondence:** Silvia Rosina

*Pediatric Rheumatology 2024*, **22(2):**PReS24-ABS-1664

**Introduction:** The management of juvenile dermatomyositis (JDM) is not standardized and no widely embraced therapeutic protocols are available. Furthermore, the optimal therapeutic targets as well as the ideal timing of their achievement are not established. Defining these aspects of the therapeutic approach would be fundamental to implement the treat-to-target (T2T) strategy.

**Objectives:** To investigate the longitudinal trends of indicators of treatment effectiveness in a cohort of JDM patients, with the aim of identifying suitable targets and optimal timing of their achievement.

**Methods:** We reviewed retrospectively the charts of 44 patients diagnosed with JDM, seen at our center within 6 months after disease diagnosis and followed for ≥ 6 months. The disease course was assessed at the following time points: baseline (diagnosis) and after 1.5, 3, 6, 12, 18 and 24 months. Collected data included demographic features, muscle enzymes, and the main physician- and parent-centered JDM outcome measures. Time to skin and muscle remission, normalization of muscle enzymes, inactive disease (ID) by PRINTO modified criteria, complete clinical response (CCR), ID by JDMAI1 and JDMAI2, reduction of prednisone (PDN) dose < 0.3 mg/kg/day and < 0.1 mg/kg/day, and glucocorticoid (GC) discontinuation was calculated. Treatment response by IMACS and PRINTO criteria, as well as longitudinal changes in JDMAI1 and JDMAI2 were also evaluated.

**Results:** A total of 44 patients (median age at diagnosis 7.5 years; median time from onset to diagnosis 4.2 months) were included. All but 4 patients received high-dose GC at diagnosis, associated with methotrexate and IVIG in 64% and 20.5%, respectively. The frequency of and median time to achievement of established targets were: 82.9% and 12.0 months for skin remission; 94.7% and 5.6 months for muscle remission; 94.1% and 3.0 months for normalization of muscle enzymes; 79.5% and 13.0 months for ID by PRINTO modified criteria; 68.2% and 18.6 months for CCR; 60% and 13.0 months for ID by JDMAI1; 64% and 12.9 months for ID by JDMAI2; 100% and 11.2 months for PDN dose reduction to < 0.3 mg/kg/day; and 56.1% and 24.7 months for PDN dose reduction to < 0.1 mg/kg/day. GC were discontinued in 34.1% patients (median time not reached). IMACS minimal/moderate/major improvement was reached in 36.4%/56.8%/40.9% of patients, whereas PRINTO minimal/moderate/major improvement in 32%/76%/52%, respectively. JDMAI1 and JDMAI2 scores declined over time, especially at 12 months (mean absolute/percentage change from baseline: -14.1/-90.0% for JDMAI1, -14.7/-84.4% for JDMAI2).

**Conclusion:** Our findings provide preliminary figures derived from the real world of clinical practice that may help to define suitable targets and optimal timing of their achievement for the future introduction of the T2T strategy in JDM.

**Patient Consent**

Not applicable (there are no patient data)

**Disclosure of Interest**

None Declared

**References**

1. Rosina S, et al. Development and validation of a composite disease activity score for measurement of muscle and skin involvement in juvenile dermatomyositis. Rheumatology (Oxford) 2019;58(7):1196-1205.
2. Rosina S, et al.; Paediatric Rheumatology International Trials Organisation (PRINTO). Defining criteria for disease activity states in juvenile dermatomyositis based on the Juvenile Dermatomyositis Activity Index. RMD Open 2024;10(1):e003093.

### Juvenile dermatomyositis

#### PT41 Development and validation of a composite parent-centered disease activity score for juvenile dermatomyositis

##### Letizia Tarantola^1^, Ana I. Rebollo-Giménez^2^, Roberta Naddei^3^, Alessandro Consolaro^2^, Angela Pistorio^4^, Angelo Ravelli^4^, Silvia Rosina^2^

###### ^1^Dipartimento di Neuroscienze, Riabilitazione, Oftalmologia, Genetica e Scienze Materno-Infantili (DiNOGMI), Università degli Studi di Genova; ^2^UOC Reumatologia e Malattie Autoinfiammatorie , IRCCS Istituto Giannina Gaslini , Genova; ^3^Dipartimento di Scienze Mediche Traslazionali, Università degli Studi di Napoli Federico II, Napoli; ^4^Direzione Scientifica, IRCCS Istituto Giannina Gaslini , Genova, Italy

####### **Correspondence:** Silvia Rosina

*Pediatric Rheumatology 2024*, **22(2):**PReS24-ABS-1800

**Introduction:** Increasing attention has been recently paid to the development of parent-centered composite disease activity scores for the assessment of health status of children with rheumatic diseases.

**Objectives:** The aim of the present study was to develop and validate an entirely parent-centered composite disease activity score for JDM, named parent Juvenile DermatoMyositis Activity Index (parJDMAI).

**Methods:** The parJDMAI includes the following items: 1) parent assessment of skin disease activity (Parent Skin Scale) on a 0-5 Likert scale, by giving 1 point to each of: i) rash on eyelids, ii) rash on nose/cheeks, iii) rash on knuckles, iv) rash on trunk/arms, v) skin ulceration; 2) parent assessment of muscle disease activity (Parent Muscle Scale) on a 0-5 Likert scale, by giving 1 point to each of: i) fatigue/discomfort, ii) muscle weakness, iii) muscle pain, iv) voice change, v) difficulty swallowing; 3) parent assessment of child’s fatigue on a 0-10 VAS (0 =no fatigue; 10 = maximum fatigue); 4) parent global assessment of disease activity on a 0-10 VAS (0 = no activity; 10 = maximum activity). To give the 4 components the same weight, the scores of the Parent Skin and Muscle Scales were converted to a 0-10 scale. Thus, the total ParJDMAI score ranges from 0 to 40. Initial validation was conducted on a multicentric prospective sample of 263 patients followed in standard clinical care assessed at baseline and at a second follow-up visit. Construct validity was assessed by calculating the correlations between parJDMAI and: i) physician-centered JDM outcome measures; ii) JDMAI1 and JDMAI2 with or without the Parent’s global assessment of overall wellbeing. Validation procedures included: responsiveness to change, internal consistency, discriminant ability and factor analysis.

**Results:** Spearman’s correlations between parJDMAI and physician-centered JDM outcome measures were moderate (0.4-0.59), whereas those with JDMAI1 and JDMAI2 were strong (0.6-0.79); correlations between parJDMAI and 3item-JDMAI1 and 3item-JDMAI2 were moderate but lower than those with original (4item-) JDMAI1 and JDMAI2, as expected. Responsiveness to change was good and internal consistency was substantial (Crombach alpha>0.8). Discriminant ability was satisfactory. Factor analysis and principal component analysis confirmed the unidimensionality and internal consistency of the scale.

**Conclusion:** The parJDMAI revealed satisfactory measurement properties and is, therefore, suitable for use in clinical practice and research. The new tool should be further tested in different clinical and cultural environments.

**Patient Consent**

Not applicable (there are no patient data)

**Disclosure of Interest**

None Declared

**Reference**

1. Rosina S, Consolaro A, van Dijkhuizen P, Pistorio A, Varnier GC, Bovis F, Nistala K, Maillard S, Civino A, Tsitsami E, de Inocencio J, Jelusic M, Vojinovic J, Espada G, Makay B, Katsicas MM, Pratsidou-Gertsi P, Lazarevic D, Rao AP, Pires Marafon D, Martini A, Pilkington C, Ruperto N, Ravelli A. Development and validation of a composite disease activity score for measurement of muscle and skin involvement in juvenile dermatomyositis. Rheumatology (Oxford). 2019 Jul 1;58(7):1196-1205. 10.1093/rheumatology/key421. PMID: 30690571.

### Juvenile dermatomyositis

#### PT42 Fibroblast Growth Factor 21 (FGF21) and Growth Differentiation Factor 15 (GDF15) plasmatic levels are increased in treatment-naive Juvenile Dermatomyositis (JDM) patients and differ among MSA subgroups

##### Maria Isabella Petrone^1^, Emiliano Marasco^1^, Giusi Prencipe^1^, Ivan Caiello^1^, Valentina Matteo^1^, Luciapia Farina^1^, Fiorella Piemonte^2^, Caterina Torda^2^, Fabrizio De Benedetti^1^, Rebecca Nicolai^1^

###### ^1^Division of Rheumatology; ^2^Laboratory of Muscular and Neurodegenerative Diseases, IRCCS Ospedale Pediatrico Bambino Gesù, Rome, Italy

####### **Correspondence:** Maria Isabella Petrone

*Pediatric Rheumatology 2024*, **22(2):**PReS24-ABS-1245

**Introduction:** Recently published evidence suggests a role for mitochondrial dysfunction in idiopathic inflammatory myopathies (IIM). The mitokines GDF15 and FGF21 are induced in situations of muscle stress, particularly mitochondrial myopathies. Previous studies demonstrated that GDF15 is increased in serum and muscle of adult patients with IIM and in plasma of JDM patients.

**Objectives:** To investigate serum levels of GDF15 and FGF21 in JDM patients at diagnosis, before start of treatment, and evaluate possible correlations with clinical and laboratory findings, as well as with IFN-related biomarkers in blood (type I IFN score, CXCL10, CXCL9 and neopterin) and muscle (type I and II IFN score on muscle biopsies).

**Methods:** We collected muscle biopsy and blood samples of 24 treatment naïve JDM patients enrolled at time of diagnosis. Serum levels of FGF21, GDF15, CXCL10, CXCL9 and neopterin were analyzed by ELISA (normal values: 0-200 pg/ml, 200-1000 pg/ml, <300 pg/ml, <150 pg/ml, <1.59 ng/ml); expression of 6 IFN-induced genes (IFI27, IFI44L, IFIT1, ISG15, RSAD2, SIGLEC1) was measured by real-time PCR and used to calculate a type I IFN score in blood and a type I score in muscle (measuring the expression of the genes cited previously). A muscle type II IFN score, based on expression levels of CIITA, IFNγ and CXCL9 was also calculated. For each patient, physician’s global assessment (PGA) of disease activity VAS (Visual Analogue Scale), Childhood Myositis Assessment Score (CMAS), serum levels of creatine phosphokinase (CK, IU/l), MSA (Myositis Specific Autoantibodies) status were recorded. Correlations were determined by the Spearman’s rank correlation coefficient. Non-parametric tests were used for comparisons between 2 groups.

**Results:** 18 out of 24 patients were female (75%). Median age at disease onset was 5.6 years [IQR 4.27, 12.03] and median disease duration at diagnosis 2.4 months [IQR 1.65, 6.95]. 17 patients were positive for at least one MSA. Median FGF21 levels were increased [261 pg/ml (IQR 43.75-617.25)], as well as median GDF15 levels [1521 pg/ml (IQR 1022-2350)]. Median GDF15 levels tended to be higher in anti-NXP2pos patients (n=4) [3675 pg/ml, (IQR 2535,4645)] when compared to the anti-NXP2neg patients (n=20), [1430 pg/ml, IQR 883-1888) (p=0.053), whereas they tended to be lower in anti-MDA5 positive patients (n=3) [896 pg/ml, IQR 817-1011)] when compared to anti-MDA5 neg patients (n=21), [1704 pg/ml, IQR 1181-2562)] (p=0.08). FGF21 levels showed significant correlation with CMAS (p=0.01, rs=-0.46) and a tendency to correlate with higher muscle type I IFN score (p=0.057, rs=0.33). GDF15 levels showed significant correlation with CK levels (p=0.002, rs=0.57), PGA-VAS (p=0.02, rs=0.4), CMAS (p=0.002, rs=-0.56) and IFN score (p=0.04, rs=0.36). GDF15 also correlated with muscle type I IFN score (p=0.05, rs=0.34).

**Conclusion:** In our cohort, a significant number of JDM patients showed increased levels of FGF21 and GDF15. GDF15 levels were highest in anti-NXP2+ patients and lowest in anti-MDA5 patients. FGF21 levels correlated with CMAS and tended to correlate with muscular type I IFN score. GDF15 levels were correlated to global and muscular disease activity. Our data support the use of GDF15, and potentially of FGF21, as biomarker for IMM.

**Patient Consent**

Yes, I received consent

**Disclosure of Interest**

None Declared

**References**

1. De Paepe B et Al. Growth differentiation factor-15 as an emerging biomarker for identifying myositis. Expert Rev Clin Immunol. 2022. 10.1080/1744666X.2022.2021879.
2. Duvvuri B et Al. Growth and Differentiation Factor 15, an emerging biomarker of mitochondrial dysfunction-associated myopathies: implications for Juvenile Dermatomyositis. Rheumatology (Oxford). 2023.  10.1093/rheumatology/kead654.

### Juvenile dermatomyositis

#### PT43 Spectrum of clinical phenotypes associated with myositis-specific antibodies in juvenile idiopathic inflammatory myositis: an update from North India

##### Vignesh Pandiarajan, Suprit Basu, Aditya Dod, Ravinder Garg, Rakesh Pilania, Manpreet Dhaliwal, Saniya Sharma, Deepti Suri, Amit Rawat, Surjit Singh

###### Pediatric Clinical Immunology and Rheumatology, Department of Pediatrics, Postgraduate Institute of Medical Education and Research, Chandigarh, India

####### **Correspondence:** Vignesh Pandiarajan

*Pediatric Rheumatology 2024*, **22(2):**PReS24-ABS-1746

**Introduction:** Idiopathic Inflammatory Myositis (IIM) are a heterogenous group of disorders with distinct clinical phenotypes associated with specific myositis-specific antibodies (MSA).

**Objectives:** To evaluate the frequency, pattern, and associations of MSA in a large Indian cohort of juvenile IIM (JIIM).

**Methods:** A review of medical records of all patients diagnosed to have JIIM during the period January 1992 - April 2024 in Pediatric Rheumatology Clinic, Advanced Pediatrics Centre, Postgraduate Institute of Medical Education and Research, Chandigarh, India, was done. Case records of children with JIIM who had significant positivity for MSA by myositis immunoblot were analyzed in detail.

**Results:** Of the 166 children with JIIM, MSA immunoblot was carried out in 95 patients. Myositis antibody was positive in 71/95 (74.7%) cases, and 17 of them were positive for multiple antibodies. The most common MSA was anti-NXP2 24 (25.3%) followed by anti-MDA5 15 (15.8%) and anti-TIF-gamma 12 (12.6%). Anti-Mi2, anti-Ro52, and anti-PM-Scl positivity were found in 9 (9.5%), 10 (10.5%), and 9 (9.5%) cases, respectively. We observed 4 (4.2%) cases of anti-SAE antibody, all of them having cutaneous disease predating muscle disease, and the myositis responded briskly to immunosuppressants. Calcinosis-predominant presentation with no clinical muscle involvement at presentation was seen in 4/24 (16.6%) cases with the anti-NXP2 antibody group. While the severe and relapsing cutaneous disease is more commonly noted in the anti-TIF-gamma group, cutaneous ulcers, arthritis and interstitial lung disease (ILD) were noted at higher rates in the anti-MDA5 group. However, we have not noted an amyopathic form in anti-MDA5 JDM in our cohort.

**Conclusion:** The spectrum of MSAs and clinical phenotypes within the particular category of MSAs in our cohort varies from other reported cohorts from the Eastern and Western world. Anti-NXP2 is the most common MSA in our cohort, with 16.6% of them presenting as calcinosis-predominant clinically amyopathic form. The anti-MDA5 subgroup had high rates of arthritis presentation, and no amyopathic form was noted in this group.

**Date of birth::** juin 03, Y

**Patient Consent**

Yes, I received consent

**Disclosure of Interest**

None Declared

### Juvenile dermatomyositis

#### PT44 Dysregulation of the TLR3/IFIH1 pathway at juvenile dermatomyositis onset implicates viral infection as a disease trigger

##### Thomas R. Moreau^1,2,3^, Vincent Bondet^2^, Chloé Albert-Vega^2^, Yixiang Y. Zhu^1^, Florian Dubois^2^, Farah Rahal^2^, Françoise Donnadieu^4^, Etienne Villain^2^, Christine Bodemer^5^, Laurye-Anne Eveillard^5^, Marie-Louise Frémond^5^, Benjamin Fournier^5^, Pierre Quartier^3,5,6^, Eugénie Sarda^3,5^, Adrien Schvartz^7^, Michael White^4^, Isabelle Melki^3,6,8^, Cyril Gitiaux^3,5^, Brigitte Bader-Meunier^5,6^, Mathieu P. Rodero^1,3^, Darragh Duffy^2^

###### ^1^Laboratoire de Chimie et de Biochimie Pharmacologiques et Toxicologiques, CNRS; ^2^Translational Immunology Unit, Institut Pasteur; ^3^Université Paris-Cité; ^4^Infectious Disease Analytics and Epidemiology Unit, Institut Pasteur; ^5^Necker-Enfants Malades Hospital, AP-HP; ^6^Reference centre for inflammatory Rheumatism, AutoImmune disease and Systemic interferonopathies in childrEn (RAISE), Paris; ^7^Bicêtre Hospital, AP-HP, Le Kremlin-Bicêtre; ^8^Robert Debré Hospital, AP-HP, Paris, France

####### **Correspondence:** Thomas R. Moreau

*Pediatric Rheumatology 2024*, **22(2):**PReS24-ABS-1504

**Introduction:** We previously reported elevated plasmatic interferon alpha levels concomitant with SARS-CoV-2 infection in juvenile dermatomyositis (JDM) at onset^1^.

**Objectives:** Here we tested the hypothesis that (i) dysregulation of specific nucleic acid sensor pathways may be implicated in JDM and (ii) specific pathogens sensed by these pathways may be associated with JDM onset.

**Methods:** We prospectively recruited 18 JDM patients at diagnosis and age-matched paediatric controls. Whole blood cellular phenotyping and single-cell phosphoproteomics were performed by mass cytometry. Responses of specific nucleic acid sensors were evaluated using standardized *ex-vivo* whole blood stimulation assays (TruCulture) with Toll-like receptor (TLR) agonists Poly(I:C), ODN 2216 and R848. Cytokine secretion was quantified using Luminex and digital ELISA (Simoa) and transcriptomic signatures by bulk-RNA-sequencing. IgG levels against 20 RNA viruses were quantified by Luminex at diagnosis.

**Results:** We observed increased proportions of transitional B cells and naïve CD8 T cells, and reduced memory B cells and effector memory CD8 T cells, in JDM patients at diagnosis compared to age-matched controls. Phosphoproteome profiles within JDM patients at diagnosis did not identify a shared inflammatory pattern between patients. In contrast, they revealed heterogeneous and globally elevated inflammatory responses. Median levels of type I interferon and specific interferon induced proteins were highly elevated in JDM patients at basal state compared to controls. Transcriptomic analysis confirmed an enrichment of the interferon pathway in JDM at diagnosis. Following TLR stimulation JDM patients showed a defective response to Poly(I:C) stimulation at both proteomic and transcriptomic levels compared to controls. In contrast, responses to R848 and ODN stimulation were not different. *IFIH1,* but not *TLR3,* was up-regulated in JDM compared to controls. Antibody analysis showed that JDM patients at diagnosis had a greater history of infections with SARS-CoV-2 and Enterovirus compared to controls.

**Conclusion:** We identified a defective response to Poly(I:C) stimulation in JDM patients implicating the IFIH1 pathway. JDM patients at diagnosis had elevated seropositivity to specific RNA viruses, suggesting their potential role in triggering *IFIH1* dysregulation and JDM onset. Validation of these viral signatures by current antibody analysis in an independent cohort may provide further confirmation. Additionally, ongoing single-cell studies will help to better understand the molecular and cellular specificities that drive RNA sensing dysregulation in JDM patients.

**Date of birth:** août 07, Y

**Patient Consent**

Yes, I received consent

**Disclosure of Interest**

None Declared

**Reference**

1. Rodero, M. P. *et al.* Onset and Relapse of Juvenile Dermatomyositis Following Asymptomatic SARS-CoV-2 Infection. *J. Clin. Immunol.* **42**, 25–27 (2022).

### Juvenile dermatomyositis

#### PT45 Lung involvement in juvenile dermatomyositis: a French retrospective study

##### Capucine Durand^1^, Isabelle Melki^2^, Christine Bodemer^3^, Brigitte Bader-Meunier^1^, Laureline Berteloot^4^, Alice Hadchouel^5^

###### ^1^Pediatric Hematology-Immunology and Rheumatology Department, Necker-Enfants Malades Hospital, Assistance Publique-Hôpitaux de Paris (AP-HP); ^2^General Pediatrics, Infectious Disease and Internal Medicine Department, Hôpital Robert Debre, Assistance Publique-Hôpitaux de Paris (AP-HP); ^3^Department of Dermatology, Referral Center for Genodermatoses and Rare Skin Diseases; ^4^Pediatric Radiology Department; ^5^Pediatric Pulmonology Department, Necker-Enfants Malades Hospital, Assistance Publique-Hôpitaux de Paris (AP-HP), Paris, France

####### **Correspondence:** Capucine Durand

*Pediatric Rheumatology 2024*, **22(2):**PReS24-ABS-1323

**Introduction:** Juvenile dermatomyositis (JDM) is a rare juvenile idiopathic inflammatory myopathy. Lung involvement may be associated with JDM, and may be life threatening(1). However, little is known about its prevalence, characteristics and course during JDM.

**Objectives:** To describe the characteristics and course of lung involvement in a cohort of children with JDM.

**Methods:** Retrospective analysis of clinical, radiological and pulmonary function tests (PFTs) data of patients with JDM and lung involvement followed in the Paris referral center for Rare Paediatric Rheumatism and Systemic Autoimmune diseases (RAISE) from January 2009 to December 2022. Inclusion criteria were: i) diagnosis of JDM according to conventional clinico-pathological criteria, ii) lung involvement assessed by evaluation of pulmonary function test (PFT) and/or chest CT scan at diagnosis and iii) follow-up of at least six months after the diagnosis. The diagnosis of interstitial lung disease (ILD) was based on CT chest scan abnormalities. All CT scans were reviewed by the same paediatric radiologist (LB). Patient’s characteristics were compared using Chi-square or Fisher’s exact test.

**Results:** Among the 148 patients followed during the considered period, 87 underwent pulmonary investigations. Sixteen patients (18%) had a diagnosis of ILD. The median age at diagnosis of JDM was 10.6 years old (8,6- 12,3). Fifteen (94%) patients had MSA: 12 anti-MDA5 (75%), 4 anti-NXP2 and 1 anti-TIF1gamma antibodies respectively. At diagnosis 9/16 (56%) had respiratory symptoms (dry cough (17%) or dyspnoea on exertion (47%)). Nine patients (56%) had abnormal PFT, including 4 patients without pulmonary symptoms: 3 had both restrictive syndrome and low DLCO, 2 had isolated low DLCO and 3 had isolated restrictive syndrome. Chest scan at diagnosis showed: ground glass lesions (82%), nodules or micronodules (47%) and condensations (41%). Pulmonary lesions were always present at diagnosis of JDM except for one patient with anti-TIF1-gamma antibodies who developed ILD two years later.

Among patients with ILD there was a higher prevalence of respiratory symptoms (56% vs 13,6%, p=0.00103), a higher prevalence of MDA5 Ab (75% vs 13%, p=1.454e-05) and a higher prevalence of hospitalization in an intensive care unit (25% vs 7,6%, p =0.04948) compared to the patients with no lung involvement. Patients with ILD were treated more often with plasma exchange (37,5% vs 12%, p=0.06247) than the other patients. Other treatments were similar in the two groups : oral corticosteroids (100%), corticosteroid pulses (n=7, 43%), methotrexate (n=12, 75%), intravenous immunoglobulins (n=7, 43%) , plaquenil (n=7), jak inhibitors (n=7), rituximab (n=8, 50%), cellcept (n=6, 37,5%) and endoxan (n=2, 12,5%).

At last evaluation (median follow up: 3.1 years), 3 patients still had dyspnoea resulting from JDM. CT chest scan improved in 9 patients (56%) and remitted in two patients. All PFTs were normal at last evaluation except for 2 patients who had low DLCO (50-73%), without clinical or radiological abnormalities. One patient with anti-TIF1-gamma antibody-positive JDM died from multivisceral failure including a severe ILD.

**Conclusion:** In our cohort of patients with JDM we observe a relatively high prevalence of ILD, which occurred mainly in patients with anti-MDA5 positive JDM. Pulmonary involvement was moderate, without case of rapidly progressive interstitial lung disease. It was present at diagnosis of JDM and improved with time in most of the patients.

**Patient Consent**

Yes, I received consent

**Disclosure of Interest**

None Declared

**Reference**

1. Abu-Rumeileh S, Marrani E, Maniscalco V, Maccora I, Pagnini I, Mastrolia MV, et al. Lung involvement in juvenile idiopathic inflammatory myopathy: A systematic review. Autoimmun Rev. oct 2023;22(10):103416.

### Juvenile dermatomyositis

#### PT46 Understanding the mechanisms of B cell and interferon pathway dysregulation in juvenile dermatomyositis

##### Aris E. Syntakas^1,2^, Huong D. Nguyen^1,2^, Restuadi Restuadi^1,2,3^, Bethany R. Jebson^1,2^, Vicky Alexiou^1,2^, Lucy R. Marshall^1,2^, Elizabeth Ralph^1,2,3^, Kathryn O’Brien^2,4^, Dario Cancemi^1,2,4^, Hannah Peckham^2^, Ania Radziszewska^2^, Nina De Gruijter^2^, George T. Hall^3,5^, Sergi Castellano^3,5^, Coziana Ciurtin^2^, Claire T. Deakin^1,2,3^, Elizabeth C. Rosser^2,4^, Lucy R. Wedderburn^1,2,3^, Melissa Kartawinata^1,2^, Meredyth G. L. Wilkinson^1,2,3^ and and on behalf of the UK Juvenile Dermatomyositis Cohort and Biomarker Study (JDCBS) and the Centre for Adolescent Rheumatology Versus Arthritis at UCL, UCLH and GOSH

###### ^1^Infection, Immunity and Inflammation Research and Teaching Department, UCL Great Ormond Street Institute of Child Health; ^2^Centre for Adolescent Rheumatology Versus Arthritis at UCL UCLH and GOSH; ^3^NIHR Biomedical Research Centre at GOSH; ^4^Centre for Rheumatology Research, University College London; ^5^Genetics and Genomic Medicine Research & Teaching Department, UCL Great Ormond Street Institute of Child Health, London, United Kingdom

####### **Correspondence:** Aris E. Syntakas

*Pediatric Rheumatology 2024*, **22(2):**PReS24-ABS-1425

**Introduction:** Juvenile Dermatomyositis (JDM) is a rare childhood autoimmune disease, characterized by muscle inflammation and skin rash. Recent investigations have implicated dysregulation of the interferon (IFN) pathways and B cell involvement.

**Objectives:** This study aims to elucidate alterations in B cell populations, their association with disease severity, and their correlation with IFN pathway dysregulation in JDM patients.

**Methods:** Transcriptomic, flow cytometry, and clinical data were generated and collected from 52 JDM patients recruited from the UK Juvenile Dermatomyositis Cohort and Biomarker Study (JDCBS). Flow cytometry was used to assess B cell populations in treatment-naïve (pre-treatment, n=42) and treated (on-treatment, n=24) JDM patients, alongside age-matched healthy donors (n=35). Differential gene expression (DGE) analysis was conducted on CD19+ B cells. Genes with |LogFC| > 0.58 and adjusted p-value < 0.05 were considered differentially expressed. Pathway over-representation and gene set enrichment analysis were performed using the Enrichr and fgsea tools. An IFN score was calculated relative to the healthy controls by summing the z-scores of the differentially expressed IFN genes per patient.

**Results:** Flow cytometry analysis revealed a significant expansion of total B cells (CD19+, p < 0.0001, ANOVA) and immature B cells (CD19+ CD24hi CD38hi, p = 0.0055, Kruskal-Wallis) in pre-treatment JDM patients compared to healthy controls, which were normalized by treatment. Additionally, memory B cells were decreased (CD19+ CD24hi CD38lo, p < 0.0001, Kruskal-Wallis). A negative correlation between total B cells and the Childhood Myositis Assessment Scale (CMAS) was observed in pre-treatment JDM patients (p = 0.0034, R-squared = 0.2517), suggesting a link between B cell expansion and increased muscle weakness. DGE analysis revealed 210 DE genes in pre-treatment JDM patients, 48 of which were IFN-related. Distinct clustering of pre-treatment patients was observed based on the expression of the IFN genes. Pathway analysis of CD19+ B cells confirmed significant enrichment of IFN pathways in pre-treatment JDM patients. The IFN score positively correlated (p = 0.0352, R-squared = 0.2132) with the immature B cell population in pre-treatment patients.

**Conclusion:** This study validates our previous findings^1^, indicating a significant role of B cells in JDM pathogenesis. An expansion of the total and immature B cell populations was observed in pre-treatment JDM patients, which correlated with disease activity and was normalized with treatment. IFN gene expression was able to cluster pre-treatment JDM patients and the IFN score positively correlated with the immature B cell population, suggesting increased involvement of this specific cell subset in JDM pathogenesis. Overall, these findings offer insights into the complex nature of JDM, potentially leading to the future identification of novel biomarkers.

**Patient Consent**

Yes, I received consent

**Disclosure of Interest**

None Declared

**Reference**

1. Piper CJM, Wilkinson MGL, Deakin CT, Otto GW, Dowle S, Duurland CL, Adams S, Marasco E, Rosser EC, Radziszewska A, Carsetti R, Ioannou Y, Beales PL, Kelberman D, Isenberg DA, Mauri C, Nistala K, Wedderburn LR. CD19^+^CD24^hi^CD38^hi^ B Cells Are Expanded in Juvenile Dermatomyositis and Exhibit a Pro-Inflammatory Phenotype After Activation Through Toll-Like Receptor 7 and Interferon-α. Front Immunol. 2018 Jun 22;9:1372. 10.3389/fimmu.2018.01372. PMID: 29988398; PMCID: PMC6024011.

### Juvenile dermatomyositis

#### PT47 Swallow assessment in newly diagnosed juvenile dermatomyositis: bedside or videofluoroscopy?

##### Ria R. Nair^1^, Kristy O’Connor^2^, Aris E. Syntakas^3^, Meredyth G. Wilkinson^3,4,5^, Gabrielle Dobson^2,6^, Charalampia Papadopoulou^2,3^

###### ^1^Faculty of Medical Sciences, Newcastle University, Newcastle Upon Tyne; ^2^Great Ormond Street Hospital; ^3^Infection, Immunity and Inflammation Programme Research and Teaching Department, UCL Great Ormond Street Institute of Child Health; ^4^Centre for Adolescent Rheumatology Versus Arthritis at UCL, University College London; ^5^NIHR Biomedical Research Centre, Great Ormond Street Hospital, London; ^6^Cambridge University Hospitals, Cambridge, United Kingdom

####### **Correspondence:** Ria Rajendrakumar Nair

*Pediatric Rheumatology 2024*, **22(2):**PReS24-ABS-1322

**Introduction:** Juvenile Dermatomyositis (JDM) is characterised by small vessel inflammation leading to myopathy, including pharyngeal weakness with dysphagia. Literature suggests that dysphagia and silent aspiration are inaccurately predicted by muscle weakness(1). International recommendations advise bedside swallow assessment (BSA) or instrumental swallow assessment at JDM diagnosis(2).

**Objectives:** To explore if BSA is as effective as videofluroscopy swallow study (VFSS) in diagnosing oropharyngeal dysphagia and aspiration risk in newly diagnosed JDM patients.

**Methods:** This service evaluation was registered with the Great Ormond Street Hospital clinical audit and service evaluation department: Registration 3758. All information was anonymised. Using the electronic patient record, SlicerDicer identified all patients with a diagnosis of juvenile myositis/ juvenile dermatomyositis between April 2019 - June 2023. 23 patients were identified with new diagnoses of JDM in this period. 2 were excluded for not receiving dual swallow assessments. Retrospective case note review collated data from BSA and VFSS tests. Swallow assessments were done by speech and language therapists (SLT). The childhood eating and drinking activity scale (CEDAS) was used to compare BSA and VFSS assessments using Spearman’s Correlation. VFSS was positioned as the gold standard test for sensitivity and specificity comparing BSA to VFSS CEDAS and BSA CEDAS to VFSS penetration aspirations scale (PAS). CEDAS scores of 6 were considered normal and ≤ 5 abnormal, PAS 1-2 were considered normal and ≥ 3 abnormal.

**Results:** BSA and VFSS CEDAS had a positive correlation with a coefficient of 0.675, p<0.001 using two-tailed analysis.

7/21 patients (33%) had worse VFSS CEDAS scores compared to BSA. For 14 patients (66%), CEDAS scores were either the same in both assessments (57%) or the CEDAS score was better in VFSS (9%). Normal BSA CEDAS compared to normal VFSS CEDAS sensitivity was 45%, with a specificity of 100%. BSA CEDAS compared to PAS sensitivity was 33% with a specificity of 80%. PAS scores were made for each food/fluid consistency tested and termed using the IDDSI framework 0-7. For IDDSI 0 and 1, 5 participants scored 6 or 5 with BSA CEDAS but had abnormal PAS. 15 of the remaining patients had normal PAS scores, despite one of these being given an abnormal score of 1 with BSA CEDAS. For IDDSI 3-4, there were no abnormal results. For IDDSI 6-8, there was one abnormal result in a participant who scored 1 on the BSA CEDAS.

**Conclusion:** These patients underwent dual swallow assessments comparing BSA and VFSS in the role of diagnosing dysphagia and aspiration risk. This service evaluation shows a correlation between the assessments indicating value in both. Dysphagia and aspiration risk are difficult to quantify from general muscle strength assessments alone. VFSS provides a more detailed assessment of swallow physiology and therefore is likelier to expose aspiration and difficulties with oropharyngeal residue in patients with pharyngeal weakness. Many of the patients with worse VFSS scores had abnormalities with thin fluids that had not been apparent from BSA alone. No data was gathered on rates of aspiration pneumonia, which could have further qualified these findings. However, this sample may have been biased as abnormal assessments receive SLT guidance to manage aspiration risk.

**Patient Consent**

Not applicable (there are no patient data)

**Disclosure of Interest**

None Declared

**References**

1. McCann LJ, Garay SM, Ryan MM, Harris R, Riley P, Pilkington CA. Oropharyngeal dysphagia in juvenile dermatomyositis (JDM): an evaluation of videofluoroscopy swallow study (VFSS) changes in relation to clinical symptoms and objective muscle scores. Rheumatology. 2007 May 27;46(8):1363–6.
2. Bellutti Enders F, Bader-Meunier B, Baildam E, Constantin T, Dolezalova P, Feldman BM, et al. Consensus-based recommendations for the management of juvenile dermatomyositis. Annals of the Rheumatic Diseases [Internet]. 2016 Aug 11 [cited 2022 Feb 18];76(2):329–40.

### Juvenile dermatomyositis

#### PT48 Testing of parent-reported outcome measures for remote assessment in juvenile dermatomyositis

##### Silvia Rosina^1^, Ana I. Rebollo Giménez^1^, Letizia Tarantola^2^, Roberta Naddei^3^, Alessandro Consolaro^1^, Angela Pistorio^4^, Angelo Ravelli^4^

###### ^1^UOC Reumatologia e Malattie Infiammatorie, IRCCS Istituto Giannina Gaslini; ^2^Dipartimento di Neuroscienze, Riabilitazione, Oftalmologia, Genetica e Scienze Materno-Infantili (DiNOGMI), Università degli Studi di Genova, Genova; ^3^Dipartimento di Scienze Mediche Traslazionali, Università degli Studi di Napoli Federico II, Napoli; ^4^Direzione Scientifica, IRCCS Istituto Giannina Gaslini, Genova, Italy

####### **Correspondence:** Silvia Rosina

*Pediatric Rheumatology 2024*, **22(2):**PReS24-ABS-1111

**Introduction:** The care of patients with juvenile dermatomyositis (JDM) requires frequent assessments of disease activity over time. However, an adequately tight frequency of physicians’ visits may not be always feasible. If the disease is stable or the child is off therapy, appointments may be deferred. In this scenario, the availability of well-established parent-reported outcomes (PROs) could allow for delivering remote symptom monitoring and telehealth, new emerging technologies that may lessen the financial burden to families, reduce the pressure on healthcare systems and help improving the quality of care.

**Objectives:** Aim of the study was to evaluate correlations between PROs potentially suitable for remote disease monitoring and physician-centered tools in JDM.

**Methods:** The following data were collected from parents of 263 JDM patients at the time of a visit at study center: 1) parent assessment of skin disease activity (Parent Skin Scale) on a 0-5 Likert scale, giving 1 point to the report of each of the following: i) rash on eyelids, ii) rash on nose/cheeks, iii) rash on knuckles, iv) rash on trunk/arms, v) skin ulceration; 2) parent assessment of muscle disease activity (Parent Muscle Scale) on a 0-5 Likert scale, giving 1 point to the report of i) fatigue/discomfort, ii)

muscle weakness, iii) muscle pain, iv) voice change, v) difficulty swallowing; 3) parent assessment of child’s fatigue on a 0-10 visual analog scale (VAS); 4) parent global assessment of disease activity on a 0-10 VAS (PaGA). Correlations between these PROs and physician-centered measures (physician’s global assessment of disease activity on a 0-10 VAS - PhGA, total DAS, skin DAS, muscle DAS, physician’s assessment of skin and muscle disease activity on a 0-10 VAS, MMT8, hMC and CMAS) were calculated.

**Results:**

**Conclusion:** The fair correlations found between the tested PROs and physician-centered tools in their respective (i.e. skin or muscle) domain suggest that parents’ evaluations may serve as surrogate measures of disease activity when physician-centered assessments are not available. This finding supports the use of PROs for remote monitoring of JDM disease course over time.

**Patient Consent**

Not applicable (there are no patient data)

**Disclosure of Interest**

None Declared

### Juvenile dermatomyositis

#### PT49 JAK inhibitor use in patients with juvenile dermatomyositis, Türkiye experience

##### Veysel Çam^1^, Yağmur Bayındır^*1^, Kübra Zora^2^, Ayşenur Çavuşoğlu^3^, Çisem Yıldız^4^, Sıla Atamyıldız Uçar^5^, Eda Kayhan^6^, Melike M. Kaplan^7^, Rüya Torun^8^, Selen D. Arık^9^, Esra Bağlan^3^, Elif Çelikel^7^, Ayşenur Paç Kısaarslan^6^, Özge Başaran^1^, Erbil Ünsal^8^, Nuray Aktay Ayaz^9^, Sevcan Bakkaloğlu^4^, Betül Sözeri^5^, Sara S. Kılıç^2^, Yelda Bilginer^1^, Seza Ozen^1^

###### ^1^Pediatric Rheumatology, Hacettepe University, Ankara; ^2^Pediatric Rheumatology, Uludağ University, Bursa; ^3^Pediatric Rheumatology, Ankara Etlik City Hospital; ^4^Pediatric Rheumatology, Gazi University, Ankara; ^5^Pediatric Rheumatology, Umraniye Training and Research Hospital, İstanbul; ^6^Pediatric Rheumatology, Erciyes University, Kayseri; ^7^Pediatric Rheumatology, Ankara City Hospital, Ankara; ^8^Pediatric Rheumatology, Dokuz Eylül University, İzmir; ^9^Pediatric Rheumatology, İstanbul University, İstanbul, Türkiye

####### **Correspondence:** Yağmur Bayındır

*Pediatric Rheumatology 2024*, **22(2):**PReS24-ABS-1284

**Introduction:** JDM, which can be classically treated with methotrexate and steroids, may require the use of DMARDs or biological drugs due to findings such as refractory muscle disease, skin ulcers, lung involvement, and calcinosis. The IFN pathway is upregulated in JDM. Therefore, the JAK-STAT pathway, which leads to the transcription of IFN-stimulated genes, is a potential target in the treatment of JDM. Clinical trials of specific JAK inhibitors in adult patients are ongoing.

**Objectives:** In this multicenter study, we aimed to share our JAK inhibitor experience in a JDM.

**Methods:** 13 JDM patients from 7 Pediatric Rheumatology centers who received JAKis between July 2015 and March 2023 were retrospectively reviewed. All patients fulfilled the Bohan-Peter classification criteria. Patients aged <18 years who started Jak inhibitor treatment and had at least 6 months follow-up data were included. Indications for treatment and response to that indication, physician VAS before and after treatment, reduction in steroid dose (6. Month if available, 12. Month), side effects, need for drug change due to treatment failure were assessed. Muscle strength, if previously recorded, was assessed with the Childhood Myositis Assessment Scale (CMAS, range 0-52) and the Manual Muscle Testing (MMT) scale (range 0-80). Organ involvement such as lung, cardiac and gastrointestinal system (GIS) was evaluated as remission-stable disesase-progression. Descriptive statistics were presented as median (IQR) for continuous variables and as number and percentage for nominal/categorical variables.

**Results:** Data from 13 patients were included. Main indications were muscle weakness (n=9), calcinosis (n=9), skin ulcers (n=3), dysphagia (n=1), interstitial lung disease (ILD) (n=1) and cardiac involvement (n=1). ). Due to payment terms, all patients received Tofacitinib. All patients had a history of glucocorticoid use, 12 patients with hydroxychloroquine, 5 patients with MMF, 12 patients with Methotrexate, 11 patients with IVIG, 7 patients with anti-TNF, and 6 patients with rituximab. Along with tofacitinib, glucocorticoid was used in ten patients, IVIG in three patients, hydroxychloroquine in five patients, and mycophenolate mofetil in two patients. One patient used it as monotherapy. No significant adverse events were reported. Progressive disease in 5 of the patients remained stable after tofacitinib. Partial remission was observed in 5 patients. In the follow-up of these, treatment was discontinued due to relapse in four of them. In 3 patients, treatment was discontinued due to lack of response. Steroid dose reduced in seven patients.

**Conclusion:** Our findings suggest that Tofacitinib is a safe alternative in the treatment of JDM. However complete remission was achieved in half of the patients. Further studies should aim to define the group of patients who would benefit from Jak inhibitors.

**Date of birth:** février 15

**Patient Consent**

Not applicable (there are no patient data)

**Disclosure of Interest**

None Declared

**References**

1. Spencer, C.H., et al., Biologic therapies for refractory juvenile dermatomyositis: five years of experience of the Childhood Arthritis and Rheumatology Research Alliance in North America. Pediatr Rheumatol Online J, 2017. 15(1): p. 50.
2. Tezak, Z., et al., Gene expression profiling in DQA1*0501+ children with untreated dermatomyositis: a novel model of pathogenesis. J Immunol, 2002. 168(8): p. 4154-63.
3. Ll Wilkinson, M.G., et al., JAK inhibitors: a potential treatment for JDM in the context of the role of interferon-driven pathology. Pediatr Rheumatol Online J, 2021. 19(1): p. 146.

### Vasculitides

#### PT50 Infantile Kawasaki disease-an experience from a tertiary care hospital in Bristol

##### Ashwini Prithvi^1^, Karthik Darma^2^, Chaitra Govardhan^1^, Paulina Connolly^2^, Reina Sangaran^1^, Athimalaipet V. Ramanan^1^, Stefania Vergnano^2^, Jolanta Bernatoniene^2^, Marion R. Roderick^2^

###### ^1^Paediatric Rheumatology; ^2^Paediatric Immunology, Bristol Royal Hospital for Children, Bristol, United Kingdom

####### **Correspondence:** Ashwini Prithvi

*Pediatric Rheumatology 2024*, **22(2):**PReS24-ABS-1429

**Introduction:** Kawasaki disease (KD) is a small to medium vessel vasculitis affecting young children. KD in infants can be a diagnostic challenge due to incomplete/atypical presentations. Upto two thirds of infants with KD develop coronary artery aneurysms (CAA)^1^.

**Objectives:** To describe the clinical profile, echocardiography and outcomes in infants diagnosed with KD.

**Methods:** A retrospective observational study was conducted at Bristol Royal Hospital for Children to include children <1 year with KD between 2011-2024. Clinical features, echocardiography and outcomes were recorded and analysed using descriptive statistics. Long-term clinical outcome data were collected where available.

**Results:** Among a total of 156 children diagnosed with KD, 28 were aged under 1 and included in this study(17.95%). Mean age was 5. 8 months [2-11] with more boys affected (Male: Female= 2.1:1). Only 8 infants fulfilled criteria for complete KD; with the remaining 20 diagnosed as incomplete KD. The presenting symptoms were rash (78.57%), mucosal changes (67.86%), bilateral non-purulent conjunctivitis (64.29%), extremity changes (42.86%) and lymphadenopathy (17.86%). 1 had reactivation of Bacillus-Calmette-Guerin scar. 21/28 (75%) developed CAA. Left main coronary artery (LMCA) was the most common, followed by right coronary artery (RCA). Giant coronary aneurysms (Z score >+10) were seen in 6/28 (21.42%). Maximum coronary artery Z score was +50. Other cardiac findings included pericardial effusion-6/28 (21.42%), severe LV dysfunction (1/28) and distal RCA thrombus (1/28). All children received intravenous immunoglobulin (IVIG) and aspirin as per protocol. Additional treatment included 2^nd^ dose IVIG in 11/28 (39.29%), methylprednisolone in 11/28 (39.29%), infliximab in 3/28 (10.71%) and anakinra in 1/28.1 infant succumbed secondary to rupture of giant aneurysm. A 1 year follow up showed resolution in 12/21 (57.14%) whereas 7/21 (33.33%) had persistent CAA and 1 had recurrence. 1 child had extensive myocardial damage and underwent heart transplant with good outcome.

**Conclusion:** Infantile KD is a diagnostic challenge with incomplete presentations being more common which may result in delayed diagnosis. Infants with KD have a higher risk of developing CAA. Infantile KD should be considered in any infant with persistent fever early to avoid long term morbidity or mortality.

**Trial registration identifying number:** Not applicable

**Date of birth::** mai 03, YY

**Patient Consent**

Not applicable (there are no patient data)

**Disclosure of Interest**

None Declared

**Reference**

1. Salgado AP, Ashouri N, Berry EK, et al. High risk of coronary artery aneurysms in infants younger than 6 months of age with Kawasaki disease. J Pediatr. 2017;185:112–116.e1.

### Vasculitides

#### PT51 Organ involvement and ANCA specificity of children with Anca-Associated Vasculitis (AAV) with the ACR/EULAR classification

##### Else S. Bosman, David A. Cabral and the PedVas Investigators Network

###### Pediatrics, UBC, Vancovuer, Canada

####### **Correspondence:** David Cabral

*Pediatric Rheumatology 2024*, **22(2):**PReS24-ABS-1579

**Introduction:** Classification criteria of pediatric vasculitis are a useful tool to define groups for research purposes and is a topic of ongoing debate among experts. In 2022, the ACR/EULAR published criteria for classifying Granulomatosis with polyangiitis (GPA) and microscopic polyangiitis (MPA) in adults, although these criteria have not been validated for pediatric ANCA-associated vasculitis (AAV) patients. It is unclear if these adult criteria differentiate between disease manifestations of pediatric AAV at the time of diagnosis.

**Objectives:** To apply the ACR/EULAR classification criteria to a large cohort of pediatric AAV patients and compare the organ involvement of the different classifications to the previously used pediatric-adapted EULAR/PRINTO/PReS (EPP-EMA) classification criteria.

**Methods:** Two classification criteria (2022 ACR/EULAR for AAV versus the pediatric EPP-EMA) were computationally applied to a registry cohort of pediatric vasculitis patients, enrolled between 2008 and 2024 in the international PedVas Initiative. We compared the ANCA specificity, and the involvement frequency of different organ systems as measured by the Pediatric Vasculitis Activity Score (PVAS). Only those patients who classified for GPA, MPA, or unclassified AAV were included in the comparison. Unclassified AAV patients were defined presence of small vessel vasculitis and tested positive for ANCA but did not meet criteria for a single classification, or alternatively fulfilled criteria for both GPA and MPA.

**Results:** -GPA classification (n=279 for EPP-EMA, n=254 for ACR/EULAR) showed a similar distribution of organ involvement although the PR3 positivity increased from 73.8% to 93.7% with the ACR/EULAR method.

- Patients who classified as MPA (n=70 for EPP-EMA, n=127 for ACR/EULAR) showed slight changes in organ involvement distribution, in particular the constitutional, mucosal, and renal disease involvement increased when using the ACR/EULAR classification criteria. MPO positivity increased from 48.6% to 100%. The distribution of organ involvement of MPA is more similar to GPA when using the ACR/EULAR criteria but the relative distribution is driven by the ANCA specificity
- Using the ACR/EULAR criteria 40 patients classified as unclassified AAV (14 not fulfilling criteria for either GPA or MPA and 26 fulfilling criteria for both). Using the EPP-EMA, 129 patients did not fulfill criteria for GPA or MPA. Comparing the two classification methods resulted in significant changes in organ involvement distribution frequency among groups. The ACR/EULAR criteria resulted in an increase of 40% of ENT symptoms and 22.8% increase of renal involvement, and MPO positivity increased with 59%.

**Conclusion:** This study shows that the adult ACR/EULAR criteria increased the number of patients that are classified as MPA and reduced the number of patients with unclassifiable AAV compared the the EPP-EMA method. GPA classification had the same frequency of organ involvement between classification methods.

Unlike the EPP-EMA, in the ACR/EULAR criteria ANCA specificity (PR3/MPO) is weighted heavily and appears the main driver in differentiating GPA from MPA. This has not led to any unique insights to improve clinical differentiation (organ frequency distribution) of GPA versus MPA. Arguments of the relative merits or pitfalls of this heavy weighting apply to both adult and pediatric disease, but alignment of classification systems and integrated inclusion of categorial criteria for MPA argue for its use in pediatrics.

**Trial registration identifying number:** NCT02006134

**Patient Consent**

Yes, I received consent

**Disclosure of Interest**

None Declare.

### Vasculitides

#### PT52 Gene expression, clinical and demographic data distinguish Kawasaki disease from other inflammatory conditions in South African children

##### Timothy F. Spracklen^1,2^, Simon C. Mendelsohn^3^, Claire Butters^1^, Heidi Facey-Thomas^1^, Mzwandile Erasmus^3^, Mthawelanga Ndengane^1^, Liesl Zühlke^1,2,4^, Thomas Scriba^3^, Kate Webb^1^

###### ^1^Department of Paediatrics and Child Health; ^2^Cape Heart Institute; ^3^South African Tuberculosis Vaccine Initiative, Institute of Infectious Disease and Molecular Medicine and Department of Pathology, University of Cape Town; ^4^South African Medical Research Council, Cape Town, South Africa

####### **Correspondence:** Timothy F. Spracklen

*Pediatric Rheumatology 2024*, **22(2):**PReS24-ABS-1629

**Introduction:** Diagnosis of Kawasaki Disease (KD) remains a challenge due to difficulty in distinguishing it from similar paediatric inflammatory disorders. Studies in patients of European ancestry have shown the clinical utility of polygenic scores in identifying KD, but no such study has been conducted in children with KD from Africa.

**Objectives:** To determine the discriminatory capacity of gene expression, demographic and clinical data for KD, compared to other presenting inflammatory conditions in Cape Town, South Africa.

**Methods:** RNA was collected from 13 pre-treatment children with KD, 42 children with multisystem inflammatory syndrome in children (MIS-C), 44 controls with other inflammatory conditions, and 74 healthy non-inflammatory paediatric controls. Expression of 80 genes with broad relevance to inflammation, immunopathology, immune regulation, and type I interferon response, was determined by real-time quantitative PCR. Differentially expressed genes (DEGs) were identified through nonparametric pairwise comparisons between experimental groups, adjusted by Holms correction. Receiver operating curve analysis was used to assess the discriminatory capacity of each gene for KD or MIS-C compared to controls. Discriminatory capacity of polygenic scores (the mean normalised expression values for each transcript of interest), clinical and demographic factors were similarly assessed.

**Results:** Children with KD were younger than those with MIS-C (p = 0.0061) and the inflammatory controls (p = 0.0049). Both KD and MIS-C were characterised by the presence of conjunctivitis, rash and tachycardia at baseline. A total of 32 DEGs were identified in KD compared to healthy children, but only two transcripts were up-regulated in KD compared to the inflammatory controls. A multi-factor score consisting of *CASP5* and *TREM1* expression, presence of conjunctivitis, and age could reliably differentiate KD from inflammatory controls (AUC 95.4%; 95% CI: 89.8-100%). This score did not perform as well in distinguishing KD from MIS-C (AUC 69.1%; 95% CI: 51.2-87.1%); however, a two-gene score of *IL27* and *SOCS1* expression could distinguish these disease groups (AUC 89.5%; 95% CI: 76.6-100%).

**Conclusion:** These data suggest that patient demographic and clinical data can be incorporated into a diagnostic algorithm for KD and MIS-C that includes gene expression of four key genes. This has the potential to greatly improve diagnosis of KD in African children. Notably, our results do not replicate recently described gene signatures of KD and MIS-C in children of European ethnicity, partly due to the use of a limited 80 gene panel in this study. Nevertheless, this highlights the importance of conducting work of this nature in underrepresented patient populations.

**Date of birth:** septembre

**Patient Consent**

Yes, I received consent

**Disclosure of Interest**

None Declared

### Vasculitides

#### PT53 A genetic insight to the etiopathogenesis of Kawasaki disease- a real time PCR based study of 15 genes in north Indian children

##### Abarna Thangaraj, Rakesh Kumar Pilania, Vibhu Joshi, Kanika Arora, Amit Kumar, Taranpreet Kaur, Priyanka Srivastava, Amit Rawat, Surjit Singh

###### Pediatrics, Postgraduate Institute of Medical Education and Research , Chandigarh, India

####### **Correspondence:** Abarna Thangaraj

*Pediatric Rheumatology 2024*, **22(2):**PReS24-ABS-1447

**Introduction:** The pathogenesis of KD is still an enigma even after 5 decades. In an in-silico analysis from our institute, we found that 15 genes (*IL1B, S100A12, MMP9, ITGAM, TLR2, PRF1, CD44, TLR8, TREM1, UBB, IL7R, FCER1G, CXCL8, SPI1*and *FCGR1A*) were associated with pathogenies of KD. In this study, we analysed gene expression of these 15 genes by real-time- polymerase chain reaction (RT-PCR) method.

**Objectives:** To study the expression of genes *IL1B, S100A12, MMP9, ITGAM, TLR2, PRF1, CD44, TLR8, TREM1, UBB, IL7R, FCER1G, CXCL8, SPI1and FCGR1A*in children with KD

To analyse the genetic expressions of these 15 genes in children with KD versus controls. Gene expression of the 15 genes was compared pre and post-IVIG treatment, the gene expressions were compared with disease controls (systemic lupus erythematosus) and in KD patients with and without coronary artery aneurysms (CAA)

**Methods:** The study was conducted from July 2022-December 2023. We enrolled 34 patients with KD, (samples were taken before and after treatment with IVIG), 9 healthy controls and 4 disease controls. We used TaqMann assay for doing RT-PCR in Step-one plus system. Relative gene expression was calculated as 2^-ΔΔCT^ (Livak method).

**Results:** Mean age at diagnosis was 32.5 months. Male to female ratio was 2.4:1. Of the 34 patients, 59% had incomplete KD. Twelve genes were up regulated (*IL1B, ITGAM, TLR2, CXCL8, SPI1, S100A12, MMP9, PRF1, TLR8, CD44, FCER1G,* and *FCGR1A*), while 3 genes (*UBB, IL7R*, and *TREM1*) were down regulated. *IL1B* and *MMP9* showed 6-fold increased expression, and S100A12 showed 4-fold increased expression. *ILIB* showed statistically significant upregulation in patients with KD compared to controls (P=0.049). *SPI-1* (p=0.0425) and *TREM1* (p=0.0180) showed a significant downregulation compared to healthy controls. On comparing pre- vs post-IVIG group in KD, genes that were downregulated during pre-IVIg phase has shown increased expression post-IVIg [*PRF1* (p=0.0313), *UBB* (p=<0.0001*), IL7R* (p=0.0022) and *TREM1* (p=0.0187)]. On comparing with the disease controls, expression of *CD44* (p=0.0400) and *TLR2* (p=0.0184) was significantly low in patients with KD. *IL1B* expression is increased in the CAA group compared to controls (p=0.025) The relative gene expression of *CD44* (p=0.023), *ITGAM* (p=0.026), *TLR2* (p=0.008) and *FCGR1A*(p=0.017) were found to be significantly downregulated in CAA group compared to KD without CAA.

**Conclusion:** Among the 15 genes, *IL1B* and *MMP9* had a 6-fold increase in expression, and *IL1B* expressions were reduced after treatment with IVIG. Decreased expression of *UBB* and *PRF1* gene in pre IVIG group suggests an involvement of interferon pathways and NK cells in the pathogenesis of KD. In KD patients with CAA, *IL1B* is found to be upregulated and *FCGR1A, TLR2, ITGAM*, and *CD44* are expressed lower compared to KD patients without CAA.

**Trial registration identifying number:** -

**Date of birth::** 11.05.1993

**Patient Consent**

Yes, I received consent

**Disclosure of Interest**

None Declared

### Vasculitides

#### PT54 Long-term follow-up patients with IGA vasculitis nephritis - the experience of the Croatian national center

##### Mario Sestan, Martina Held, Antonia Vuksic, Nastasia Kifer, Marijan Frkovic, Marija Jelusic

###### Department of Paediatrics, University of Zagreb School of Medicine, University Hospital Centre Zagrebi, Zagreb, Croatia

####### **Correspondence:** Mario Sestan

*Pediatric Rheumatology 2024*, **22(2):**PReS24-ABS-1403

**Introduction:** The discussion surrounding the duration of follow-up for IgA vasculitis (IgAV) patients at risk of developing nephritis (IgAVN) remains unresolved. Moreover, there’s still no consensus on the appropriate follow-up period for patients who develop IgAVN.

**Objectives:** Hence, our research sought to delineate the clinical and laboratory traits of individuals with IgAVN, alongside probing factors linked to both short and long-term prognoses in these patients.

**Methods:** We extracted patients diagnosed with IgAVN from the national cohort of individuals with IgAV diagnosed between 2010 and 2024, utilizing the EULAR/PRES/PRINTO criteria. Specifically, we focused on patients who received follow-up care for a minimum of six months at the largest tertiary center.

**Results:** During the specified timeframe, 739 patients received an IgAV diagnosis, with 155 subsequently developing IgAVN. Among these, 93 were monitored for more than six months, of whom 43 (46%) were male. The median age at IgAVN diagnosis was 7.62 (5.62, 11.635) years, with nephritis typically appearing 4 (0, 20) days after initial symptoms, though onset could occur as late as 559 days afterward. Nephritic syndrome manifested in 14 patients (15%), nephrotic syndrome in 6 (6.5%), and the remainder exhibited abnormal urine findings, primarily erythrocyturia and proteinuria (43%), or isolated erythrocyturia (40.9%). Hypertension was recorded in 22.5% of patients. Median follow-up was 15 (9.5, 43.5) months. Treatment predominantly consisted of systemic glucocorticoids (50.5%), followed by NSAIDs (33.3%) and immunosuppressants (17.2%). At the six-month mark post-diagnosis, abnormalities in urine persisted in 34 patients (36.6%), decreasing to 14 patients (15%) after 12 months, signifying a noteworthy enhancement in laboratory results (p < 0.01). However, after 6 and 12 months, patient outcomes did not significantly differ (p = 1.0), with 78 patients (83.9%) experiencing a good outcome, 13 (14%) achieving a good but suboptimal outcome, and 1% encountering renal function failure.There was no significant outcome difference between patients with isolated erythrocyturia and those with proteinuria after six or twelve months (p = 0.74). Among patients followed-up for over six months (44% of the cohort), there were significantly more males (p = 0.02) and individuals treated with immunosuppressive agents (p = 0.08).

**Conclusion:** Our findings indicate that IgAVN typically arises within the initial 4 days of IgAV onset, with most patients experiencing a positive outcome. We considered outcomes most favorable after more than 12 months post-diagnosis, aligning with simultaneous improvements in our patients’ laboratory findings. However, there’s no notable contrast in outcomes between individuals with isolated erythrocyturia and those with proteinuria. While the outcome doesn’t significantly differ between patients at six and twelve months of follow-up, our study highlights that 4% of patients developed IgAVN after surpassing six months but less than twelve months of monitoring. This underscores the importance of extending follow-up to at least twelve months for individuals with IgAV. SUPPORT: Croatian Science Foundation project IP-2019-04-8822

**Patient Consent**

Yes, I received consent

**Disclosure of Interest**

None Declared

### Vasculitides

#### PT55 Clinical characteristics of pediatric Behçet’s disease in different geographies

##### Ummusen Kaya Akca^1^, Farhad Shahram^2^, Erdem Karabulut^3^, Massoomeh Akhlaghi^2^, Ozlem Akgun^4^, Mustafa Cakan^5^, Esra Esen^6^, Caterina Matucci Cerinic^7,8^, Ruya Torun^9^, Erbil Unsal^9^, Marco Gattorno^8^, Nuray Aktay Ayaz^4^, Aysenur Pac Kısaarslan^6^, Betul Sozeri^5^, Ezgi D. Batu^1^, Isabelle Koné-Paut^10^, Seza Ozen^1^

###### ^1^Department of Pediatric Rheumatology, Hacettepe University Faculty of Medicine, Ankara, Türkiye; ^2^Tehran University of Medical Sciences, Rheumatology Research Center, Shariati Hospital, Tehran, Iran, Islamic Republic Of; ^3^Basic Medical Sciences, Department of Biostatistics, Hacettepe University Faculty of Medicine, Ankara; ^4^Department of Pediatric Rheumatology, Istanbul University, Istanbul Faculty of Medicine; ^5^Department of Pediatric Rheumatology, Umraniye Training and Research Hospital, Istanbul; ^6^Department of Pediatric Rheumatology, Erciyes University Faculty of Medicine, Kayseri, Türkiye; ^7^Università degli Studi di Genova, DINOGMI; ^8^IRCCS Istituto Giannina Gaslini, UOC Rheumatology and autoinflammatory diseases, Genoa, Italy; ^9^Department of Pediatric Rheumatology, Dokuz Eylül University, Faculty of Medicine, İzmir, Türkiye; ^10^Bicêtre University Hospital, Assistance Publique-Hôpitaux de Paris, Paris-Saclay University, Department of Paediatric Rheumatology, CEREMAIA, Le Kremlin-Bicêtre, France

####### **Correspondence:** Ummusen Kaya Akca

*Pediatric Rheumatology 2024*, **22(2):**PReS24-ABS-1708

**Introduction:** Behçet’s disease (BD) has a heterogeneous presentation with a wide range of clinical signs and symptoms. The clinical features vary not only from patient to patient but also according to gender and geographical regions.

**Objectives:** In this study, we aimed to describe the clinical features and patterns of phenotype aggregation in a large cohort in pediatric BD. We also compared the clinical characteristics of BD in three patient groups from three different geographical regions: Turkey, Europe, and Iran.

**Methods:** Retrospective data of pediatric patients with BD in 8 centers from 4 countries were evaluated. Patients with pediatric onset (<18 years) BD who had a follow-up period of at least 6 months were included. Patients from Italy and France were grouped together as a single category, representing the European countries.

**Results:** A total of 600 patients (297 females, 49.5%) were included in the study. Of these, 231 were from Turkey, 306 from Iran, 44 from France, and 19 from Italy. The median age at diagnosis and the median follow-up time were 14.4 (25p-75p; 12.0-16.0) and 3.0 (25p-75p; 0.8-6.3) years, respectively. A family history of BD in first-degree relatives was identified in 14.3 percent of patients (n=86). The most common presentations were mucocutaneous (97.5%) and ocular (48.0%), followed by musculoskeletal (43.2%), neurological (11.8%), vascular (11.5%), gastrointestinal (9.0%), and cardiac (2.0%) involvement. Pathergy test and HLAB51 were positive in 224 (37.3%) and 296 (49.3%) patients, respectively. The frequency of ocular involvement was more prevalent in Iran, gastrointestinal involvement in Europe, and musculoskeletal and vascular involvement in Turkey compared to the other two geographic regions. In pediatric BD, we were not able to define clear clusters as defined in adults.

For treatment, the most commonly prescribed drug was colchicine (73.2%) while systemic steroids were used in 53.6%, azathioprine in 33.9%, methotrexate in 27.3%, and biologic treatments in 12.5% of patients. Remission on drugs was observed in 47.8% of the patients, remission off drugs in 11.4%, and partial remission in 46.0%. Eleven patients (31.4%) experienced relapse.

**Conclusion:** There are notable variations in the prevalence of organ involvement in BD across geographical regions. However, clear clusters were not defined. BD mainly presents with mucocutaneous and ocular manifestations in pediatric population but has wide phenotypic clinical variability. Addressing the full spectrum of clinical manifestations of pediatric BD in different geographic areas may led us to make inferences about the pathogenetic pathways and course of the disease.

**Date of birth::** janvier 01

**Patient Consent**

No, I have not received consent

**Disclosure of Interest**

None Declared

### Vasculitides

#### PT56 Involvement of gd-iga1, hmgb1, rage, and pcdh1 in childhood IGA Vasculitis (IGAV)

##### Martina Held^1^, Ana Kozmar^2^, Mario Sestan^1^, Daniel Turudic^1^, Nastasia Kifer^1^, Sasa Srsen^3^, Alenka Gagro^4^, Marijan Frkovic^1^, Marija Jelusic^1^

###### ^1^University Hospital Centre Zagreb, Department of Paediatrics, Division of Clinical Immunology and Rheumatology; ^2^Department of laboratory diagnostics, University Hospital Centre Zagreb, University of Zagreb School of Medicine, Zagreb; ^3^Department of Paediatrics, University Hospital Centre Split, University of Split School of Medicine, Split; ^4^Children’s Hospital Zagreb, Josip Juraj Strossmayer University of Osijek, Medical Faculty Osijek, Zagreb, Croatia

####### **Correspondence:** Mario Sestan

*Pediatric Rheumatology 2024*, **22(2):**PReS24-ABS-1072

**Introduction:** IgA vasculitis (IgAV) is the most common systemic vasculitis of childhood. The pathogenesis of IgAV is more complex than it appears with many intertwined factors, including various molecules that may play the role of biomarkers. Regarding the latter, biomarkers are some of the current research topics related to IgAV since identification of a useful surrogate biomarker that could indicate an active disease and predict possible damage, particularly IgA vasculitis nephritis (IgAVN), remains a challenge.

**Objectives:** We aimed to investigate potential role of four molecules in the pathogenesis of IgAV: high mobility group box 1 (HMGB1), receptor for advanced glycation end products (RAGE), galactose-deficient immunoglobulin A1 (Gd-IgA1) and protocadherin 1 (PCDH1).

**Methods:** A prospective study enrolled 86 children with IgAV and 70 children from the control group. HMGB1, RAGE, Gd-IgA1 and PCDH1 in serum and urine were determined by the enzyme-linked immunosorbent assay (ELISA) method at the onset of the disease and after six months interval and once in the control group.

**Results:** Concentrations of HMGB1, RAGE and PCDH1 in sera and concentrations of HMGB1, RAGE, Gd-IgA1 and PCDH1 in urine were statistically significantly higher in children with IgAV than in the control group (p<0.001). A statistically significant difference was observed in concentrations of HMGB1 (5573 pg/mL vs. 3477 pg/mL vs. 1088 pg/mL, p<0.001) and RAGE (309 pg/mL vs. 302.4 pg/mL vs. 201.3 pg/mL, p=0.012) in sera of children with IgAV at the onset of the disease compared to six months interval and between the control group. Logistic regression distinguished serum Gd-IgA1 (CI 0.943-0.982, p=0.028), RAGE (CI 0.983-0.998, p=0.026) and PCDH1 (CI 1.021-1.137, p=0.012) and urinary HMGB1 (CI 1.000-1.002, p=0.026) as predictors of arthritis. Cox regression analysis didn’t reveal any of investigated biomarkers as a predictor of IgAVN. However, concentration of HMGB1 in urine after six month follow-up was higher in children with IgAVN compared to IgAV without nephritis (270.9 (146.7-542.7) ng/mmol vs. 133.2 (85.9-318.6) ng/mmol, p=0.049) and significantly positively correlated with urine albumine to creatinine ratio (τ=0.184, p<0.05), the number of erythrocytes in urine samples (τ=0.193, p<0.05) and with outcome of nephritis (τ=0.287, p<0.05).

**Conclusion:** Our results suggest that Gd-IgA1, HMGB1, RAGE and PCDH1 interplay in the complex pathogenesis of IgAV, with HMGB1 and RAGE showing elevated values during the disease follow-up interval and thus may indicate residual low inflammatory activity or tissue damage. Although none of the assessed molecules revealed as a predictor of IgAVN, urinary HMGB1 highlighted as a potential tool in the follow up of children who developed IgAVN.

**SUPPORT:** Croatian Science Foundation Project IP-2019-04-8822.

**Date of birth::** décembre 1

**Patient Consent**

Yes, I received consent

**Disclosure of Interest**

None Declared

### Vasculitides

#### PT57 Factors associated with the need for immunosuppressive therapy in pediatric Behcet’s disease

##### Melike Mehveş Kaplan, Nimet Öner, Cüneyt Karagöl

###### Division of Pediatric Rheumatology, Department of Pediatrics, University of Health Sciences, Ankara Bilkent City Hospital, Ankara, Türkiye

####### **Correspondence:** Melike Mehveş Kaplan

*Pediatric Rheumatology 2024*, **22(2):**PReS24-ABS-1481

**Introduction:** In the treatment of Behcet’s Disease, early management is essential to relieve symptoms, control inflammation, and prevent relapses, damage and complications.

**Objectives:** Our study aimed to determine the predictors for the need for immunosuppressive therapy in pediatric Behcet’s Disease at the time of diagnosis.

**Methods:** This retrospective observational study was conducted in patients diagnosed with Behcet’s Disease in our center between 2010 and 2023. The diagnosis of Behcet’s Disease was confirmed with the Pediatric Behcet’s Disease criteria. Patients were divided into two groups: those treated with only colchicine and those with immunosuppressives.

**Results:** 107 (100%) patients diagnosed with Behcet’s Disease were included in the study. 59 (55.1%) of the patients were female and 48 (44.9%) were male. The median age at symptom onset was 11 (8-13) years, and the median age at diagnosis was 14 (11-15) years. The median duration of symptoms until diagnosis was 1 (1-2) year. There was a family history of Behcet’s Disease in 40 (37.4%) patients. 107 (100%) patients had oral ulcer, 62 (57.9%) had genital ulcer, 46 (43%) had cutaneous involvement, 22 (20.6%) had vascular involvement, 16 (15%) had neurological involvement, 22 (20.6%) had ocular involvement, 37 (34.6%) had musculoskeletal involvement and 8 (5.7%) had epididymitis. 34 (31.8%) patients had a positive pathergy test and 54 (50.5%) patients had a positive human leucocyte antigen (HLA) B5/51 gene test. The median follow-up period was 2 (1-5) years. While 70 (65.4%) patients were treated with only colchicine, 37 (34.6%) patients were treated with immunosuppressive. 27 (25.2%) patients treated with immunosuppressive at the time of diagnosis and 10 (9.3%) patients with during follow-up. 27 (25.2%) patients were treated with azathioprine, 10 (9.3%) with cyclophosphamide, 8 (7.5%) with infliximab, 3 (2.8%) with adalimumab, 23 (21.5%) with steroid and 4 (3.8%) with methotrexate. 13 (12.1%) patients were treated with 1, 13 (12.1%) with 2, 8 (7.5%) with 3, and 3 (2.8%) with 4 different immunosuppressive. The most frequently used biologic immunosuppressive was infliximab. Median leukocyte count, neutrophil count and erythrocyte sedimentation rate values at the time of diagnosis were higher in immunosuppressive-treated patients (p=0.001, p=0.004, p=0.001). Fever was more common in immunosuppressive-treated patients and genital ulcers were more common in patients treated with only colchicine (p=0.0001, p=0.004).

**Conclusion:** It is important to predict patients who may need immunosuppressive therapy at the time of diagnosis in Behcet’s disease. Patients presenting with fever and elevated leukocyte count, neutrophil count and erythrocyte sedimentation rate values should be considered to have higher immunosuppressive needs in treatment management.

**Trial registration identifying number:** The abstract does not report the results of a controlled healthcare intervention

**Date of birth::** octobre 18

**Patient Consent**

Yes, I received consent

**Disclosure of Interest**

None Declared

### COVID-19 (Coronavirus)

#### PT58 A data-driven multi-omic approach identifies shared clinical and biological signatures in multisystem inflammatory syndrome in children and Kawasaki disease

##### Sophie Sun^1,2^, Paul Tsoukas^3,4^, Hua Lu^2,5^, Lysa Langevin^2^, Marla Mendes de Aquino^6^, Trang Duong^2^, Mohammad Massumi^2^, Marvin J. Fritzler^7^, Rae S. Yeung^1,2,3,4^

###### ^1^Immunology, University of Toronto; ^2^Cell Biology Program, The Hospital for Sick Children Research Institude; ^3^The Institute of Medical Science; ^4^Division of Rheumatology, Department of Pediatrics, The Hospital for Sick Children; ^5^Biostatistics, Dalla Lana School of Public Health; ^6^The Center for Applied Genomics, The Hospital for Sick Children, Toronto; ^7^Cumming School of Medicine, University of Calgary, Calgary, Canada

####### **Correspondence:** Rae S. Yeung

*Pediatric Rheumatology 2024*, **22(2):**PReS24-ABS-1786

**Introduction:** Multisystem inflammatory syndrome in children (MIS-C) is a hyperinflammatory condition temporally associated with SARS-CoV-2 infection in children. Initial insights to the treatment of MIS-C derived from Kawasaki Disease (KD), which it clinically resembles. However, currently there is limited and conflicting data describing the pathobiology driving MIS-C and its relation to KD as case definitions for MIS-C differ between countries and organizations. Machine learning can interrogate data in a systematic and objective way to identify homogenous subgroups of patients, providing insights for diagnosis and treatment.

**Objectives:** We aim to systematically characterize children with MIS-C and KD using an unsupervised machine learning approach applied to multi-omic patient data.

**Methods:** A standardized set of pre-treatment clinical data and biospecimens were collected from MIS-C and pre-pandemic KD patients. Serum cytokines, soluble cytokine receptors and interferon response gene (IRG) expression were measured using Luminex and NanoString technologies, respectively. The data collected underwent dimensionality reduction by cross-validated probabilistic principal component analysis (PPCA). Sparsified PPCA scores were subsequently used for patient clustering by Gaussian Mixture Models (GMM). The resulting patient groups were characterized.

**Results:** Sparsified PPCA produced four composite signatures that captured approximately 60% of dataset variation. These composite signatures were meaningful clinically and biologically and can be described as: 1) Elevated IRGs and cytopenias 2) Elevated cytokines and young age, 3) Hyperinflammation and cytokine antagonists, and 4) Platelet and endothelial activation. GMM resulted in four patient clusters with distinct clinical and biological profiles that corresponded to disease severity, treatment response and patient outcome. Patients with MIS-C and KD shared multiple features and were found in all four clusters. The clusters identified unique endotypes, which can be described as: Hyperinflammatory KD, Interferon-mediated KD, Mild KD, and KD Shock. A p-value titration of response variables indicated that the cluster classification outperforms other approaches to classification including traditional clinical diagnoses, bedside clinical phenotypes, and MIS-C case definitions in identifying homogeneous patient groups.

**Conclusion:** Data-driven machine learning approaches identified clinically and biologically meaningful patient subgroups in children with post-infectious hyperinflammatory syndromes. These subgroups correlated with clinical phenotype, biologic signatures and treatment response and included patients with MIS-C and KD, suggesting that KD and MIS-C fit within the same disease spectrum. Data-driven patient subtyping will aid in stratifying children with post-infectious hyperinflammation towards personalized prediction and therapy.

**Patient Consent**

Yes, I received consent

**Disclosure of Interest**

None Declared

### Genetics, genomics and environment

#### PT59 Common seasonal pathogens and epidemiology of Henoch-schönlein purpura among children

##### Arthur Felix^1^, Zein assad^2^, Phillipe Bidet^2^, Cecile Dumaine^2^, Albert Faye^2^, Isabelle Melki^2^, Florentia Kaguelidou^2^, Zaba Valtuile^2^, Naim Ouldali^2^, Ulrich Meinzer^2^

###### ^1^Martinique university hospital, For-de-France; ^2^Robert Debré university hospital, Paris, France

####### **Correspondence:** Ulrich Meinzer

*Pediatric Rheumatology 2024*, **22(2):**PReS24-ABS-1088

**Introduction:** Henoch-Schönlein purpura (HSP) is the most common type of vasculitis in children. The factors that trigger the disease are poorly understood. Although several viruses and seasonal bacterial infections have been associated with HSP, differentiating the specific associations of these pathogens with the onset of HSP remains a challenge due to their overlapping seasonal patterns.

**Objectives:** To analyze the role of seasonal pathogens in the epidemiology of HSP.

**Methods:** This cohort study comprised an interrupted time-series analysis of patient records from a comprehensive national hospital-based surveillance system. Children younger than 18 years hospitalized for HSP in France between January 1, 2015, and March 31, 2023, were included. The main outcomes were the monthly incidence of HSP per 100 000 children, analyzed via a quasi-Poisson regression model, and the estimated percentage of HSP incidence potentially associated with 14 selected common seasonal pathogens over the same period. We studied these incidences before, during and after implementation and relaxation of nonpharmaceutical interventions (NPIs) for the COVID-19 pandemic.

**Results:** The study included 9790 children with HSP (median age, 5 years [IQR, 4-8 years]; 5538 boys [56.4%]) and 757 110 children with the infectious diseases included in the study (median age, 0.7 years [IQR, 0.2-2 years]; 393 697 boys [52.0%]). The incidence of HSP decreased significantly after implementation of NPIs in March 2020 (−53.6%; 95% CI, −66.6% to −40.6%; *P* < .001) and increased significantly after the relaxation of NPIs in April 2021 (37.2%; 95% CI, 28.0%-46.3%; *P* < .001). The percentage of HSP incidence potentially associated with *Streptococcus pneumoniae* was 37.3% (95% CI, 22.3%-52.3%; *P* < .001), the percentage of cases associated with *Streptococcus pyogenes* was 25.6% (95% CI, 16.7%-34.4%; *P* < .001), and the percentage of cases associated with human rhino enterovirus was 17.1% (95% CI, 3.8%-30.4%; *P* = .01). Three sensitivity analyses found similar results.

**Conclusion:** This study found that significant changes in the incidence of HSP simultaneously with major shifts in circulating pathogens after NPIs for the COVID-19 pandemic indicated that approximately 60% of HSP incidence was potentially associated with pneumococcus and group A streptococcus. This finding suggests that preventive measures against these pathogens could reduce the incidence of pediatric HSP.

**Patient Consent**

Yes, I received consent

**Disclosure of Interest**

None Declared

### Miscellaneous rheumatic diseases

#### PT60 A large cohort comparison of paediatric Sjogren’s disease with adult-onset

##### Flavia Riccio^1^, Saviana Gandolfo^2^, Silvana Ancona^3^, Rosaria Irace^1^, Achille Marino^4^, Simona Campus^5^, Gabriele Simonini^6^, Francesco Baldo^4^, Edoardo Marrani^6^, Angela Mauro^7^, Sofia D’Elios^8^, Adele Civino^9^, Carlotta Covizzi^10^, Valeria Manfrè^11^, Roberta Naddei^12^, Roberta Loconte^13^, Valentina Ibba^5^, Francesca Alessi^3^, Marco Gattorno^3^, Francesco Ciccia^1^, Clara Malattia^3, 14^

###### ^1^University of Campania “Luigi Vanvitelli”; ^2^Ospedale San Giovanni Bosco e Ospedale del mare, Naples; ^3^IRCCS Istituto Giannina Gaslini, Genoa; ^4^ASST G. Pini-CTO, Milan; ^5^Ospedale Microcitemico “Antonio Cao”, Cagliari; ^6^Azienda Ospedaliera Universitaria Meyer, Florence; ^7^Ospedale Fatebenefratelli e Oftalmico, Milan; ^8^Ospedale S.Chiara di Pisa, Pisa; ^9^Ospedale “Vito Fazzi”, Lecce; ^10^Azienda Ospedaliera “Città della Salute e della Scienza di Torino”, Turin; ^11^University of Udine, Udine; ^12^University of Naples “Federico II”, Naples; ^13^Azienda Ospedaliera Policlinico Consorziale di Bari, Bari; ^14^University of Genoa, Genoa, Italy

####### **Correspondence:** Flavia Riccio

*Pediatric Rheumatology 2024*, **22(2):**PReS24-ABS-1078

**Introduction:** Paediatric Sjögren’s Disease (SjD) is a rare, poorly defined and probably under-recognized condition. A limited number of studies have highlighted some clinical and immunologic differences with adult patients diagnosed with SjD. However, no comparative studies have been published to date.

**Objectives:** To compare the clinical features, immunologic profile and management in children and adults with SjD.

**Methods:** We recruited 76 patients diagnosed with paediatric SjD from 12 paediatric rheumatology centres and 115 adult SjD patients from a single rheumatology centre. All patients met the ACR/EULAR 2016 classification criteria for SjD. We collected demographic, clinical and laboratory data at the time of diagnosis and at 1-year follow-up.

**Results:** The paediatric cohort consisted of 76 patients (69 female (90.8%)) with a median age at onset of 10.83 years (0.42-16.75) and a mean time from onset to diagnosis of 18 months. The adult cohort consisted of 115 patients (107 female (93%)), with a median age at diagnosis of 55.33 years (21.58-77.58) and a mean time from onset to diagnosis of 23 months. At baseline, we observed significant differences between paediatric and adult SjD patients in xerophthalmia (43.24% vs. 93.9%, p<0.00001), xerostomia (31.08% vs. 86.2%, p<0.00001), myalgia (17.56% vs. 70.69%, p<0.00001), and fatigue (28.37% vs. 66.38%, p<0.00001), which were the predominant symptoms in the adult cohort. Consistent with these findings, Schirmer’s test (29.31% vs. 94.44%, p<0.00001) and unstimulated salivary flow rates (56.25% vs. 86.48%, p=0.005) were more frequently pathologic in adult patients. Recurrent parotid swelling was more common in children (43.2% vs. 16.37%, p=0.00005) who also had a higher incidence of arthritis (p<0.00001), purpura (p=0.01), and other skin manifestations such as erythema multiforme (p=0.005). Serum IgG levels (p<0.0001) and rheumatoid factor values (p=0.001) were significantly higher in children than in adults. A higher frequency of ANA positivity (p=0.001) and anti-SSA (p< 0.00001) and anti-SSB (p=0.0004) antibodies was observed in the adult cohort. No significant differences in treatment were found, except for saliva and tear substitutes, which were used more frequently in adults (p=0.0002 and p=0.035, respectively). ESSDAI values were significantly higher in the paediatric group (p<0.00001), while ESSPRI values were significantly lower (p<0.00001). At 1 year follow-up, although both scores improved in the two groups, ESSDAI scores remained significantly elevated in children compared to adults (p<0.00001).

**Conclusion:** Adults and children with SjD have different clinical and serological phenotypes. Pediatric SjD patients had higher disease activity as confirmed by ESSDAI despite lower ESSPRI. Overall, our results support the need to develop specific recommendations and outcome measures for the management of paediatric SjD.

**Patient Consent**

Yes, I received consent

**Disclosure of Interest**

None Declared

**Reference**

1. Rheumatology (Oxford).2021 Oct 2;60(10):4558-4567

### Miscellaneous rheumatic diseases

#### PT61 Children with type 1 interferonopathy: commonalities and diversities in a large patient cohort

##### Fatih Haslak^1^, Huseyin Kilic^2^, Sezgin Sahin^1^, Busra Hotaman^1^, Nur M. Cebi^1^, Mehmet Yildiz^1^, Amra Adrovic^1^, Aybuke Gunalp^1^, Elif Kilic Konte^1^, Esma Aslan^1^, Umit Gul^1^, Negis Akay^1^, Yilmaz Zindar^2^, Fitnat Ulug^2^, Serhat Guler^2^, Ayca Kiykim^3^, Sezin Aydemir^3^, Kenan Barut^1^, Sema Saltik^2^, Haluk C. Cokugras^3^, Ozgur Kasapcopur^1^

###### ^1^Department of Pediatric Rheumatology; ^2^Department of Pediatric Neurology; ^3^Department of Pediatric Allergy, and Immunology, Istanbul University-Cerrahpasa Cerrahpasa Medical School, Istanbul, Türkiye

####### **Correspondence:** Fatih Haslak

*Pediatric Rheumatology 2024*, **22(2):**PReS24-ABS-1413

**Introduction:** Type I interferonopathies are a class of genetic autoinflammatory disorders that result from an imbalance in the interferon pathway. These diseases are related to the innate immune system’s response to viral antigens, with a variety of genetic mutations leading to different clinical manifestations. Patients with these disorders may experience symptoms related to autoinflammation, autoimmunity, and immunodeficiency. Most of our understanding of these conditions has been derived from case reports.

**Objectives:** Owing to the lack of cohort studies, our main objective was to provide a comprehensive overview of the clinical features, laboratory and screening results, treatment options, and outcomes of patients with various subtypes of type 1 interferonopathy. We also sought to illustrate the relationship between genotype and phenotype based on the involvement of specific systems or organs and the varying degrees of autoinflammation, immunodeficiency, and autoimmunity. Our secondary objective was to determine the factors that predict long-term morbidities or fatalities, which are typically associated with poor outcomes.

**Methods:** We included children with genetically confirmed type 1 interferonopathies, with a follow-up duration of > 1 year. Data were obtained retrospectively from medical records.

**Results:** Of the 40 eligible patients for the study, 52.5% were female, with a median age of disease onset of 1.5 years (0.1-13.2). They were diagnosed at an average age of 6.85 ± 4.56 years. Aicardi-Goutières Syndrome was the most common diagnosis (n=15; 37.5%). The central nervous system was the most frequently affected system (n=27; 67.5%). Janus kinase inhibitors were administered to 17 (42.5%) patients. Twenty-five patients (62.5%) developed at least one permanent morbidity or died during follow-up; thus, they were included in the poor-outcome group. While younger age at disease onset, intracranial calcification (ICC), and lack of chilblains and elevated acute phase reactants (APRs) were significant in univariate logistic regression analysis, only ICC on MRI at admission (aOR, 19.691; 95% C.I.: 1.080-359.054, p=0.044) was found to be a significant predictor of poor outcomes in multivariate logistic regression analysis.

**Conclusion:** For the first time, we evaluated the predictors of poor outcomes in patients with type 1 interferonopathy with a broad spectrum of subtypes. Furthermore, our study’s unique patient characteristics and phenoytpe-genotype correlation data can provide valuable insights into these extremely rare conditions.

**Date of birth:** janvier 02

**Patient Consent**

Yes, I received consent

**Disclosure of Interest**

None Declared

**References**

1. d’Angelo DM, Di Filippo P, Breda L, Chiarelli F. Type I Interferonopathies in Children: An Overview. Front Pediatr. 2021;9:631329.
2. Demirkaya E, Sahin S, Romano M, Zhou Q, Aksentijevich I. New Horizons in the Genetic Etiology of Systemic Lupus Erythematosus and Lupus-Like Disease: Monogenic Lupus and Beyond. J Clin Med. 2020;9(3).
3. Rice GI, Melki I, Frémond ML, et al. Assessment of Type I Interferon Signaling in Pediatric Inflammatory Disease. J Clin Immunol. 2017;37(2):123-32.

### New diseases

#### PT62 Neuroinflammation, Autoinflammation, Splenomegaly and Anaemia (NASA) caused by bi-allelic mutations in IRAK4

##### Samantha Cooray^1^, Fiona Price-Kuehne^1^, Ying Hong^1^, Ebun Omoyinmi^1^, Alice Burleigh^1,2^, Kimberly Gilmour^3^, Bilal Ahmed^4^, Sangdun Choi^4^, Mohammed W. Bahar^5^, Paul Torpiano^3^, Andrey Gagunashvili^6^, Barbara Jensen^1^, Evangelos Bellos^7,8^, Vanessa Sancho Shimizu^7,8^, Jethro Herberg^7,8^, Kshitij Mankad^9^, Atul Kumar^10^, Marios Kaliakatsos^11^, Austen J. J. Worth^3^, Elizabeth Whittaker^7,8^, Despina Eleftheriou^1^, Paul Brogan^1^

###### ^1^Infection, Immunity and Inflammation Department; ^2^Centre for Adolescent Rheumatology Versus Arthritis, University College London Great Ormond Street Institute of Child Health; ^3^Department of Immunology and Gene Therapy, Great Ormond Street Hospital for children NHS Foundation Trust, London, United Kingdom; ^4^Department of Molecular Science and Technology, Ajou University, Suwon, Korea, Republic Of; ^5^Division of Structural Biology, University of Oxford, Oxford, United Kingdom; ^6^Faculty of Life and Environmental Sciences, University of Iceland, Reykjavík, Iceland; ^7^Section of Paediatric Infectious Diseases; ^8^Centre for Paediatrics and Child Health, Faculty of Medicine, Imperial College London; ^9^Department of Radiology; ^10^Department of Histopathology; ^11^Department of Neurology, Great Ormond Street Hospital for children NHS Foundation Trust, London, United Kingdom

####### **Correspondence:** Samantha Cooray

*Pediatric Rheumatology 2024*, **22(2):**PReS24-ABS-1676

**Introduction:** Interleukin-1 (IL-1) receptor-associated kinase-4 (IRAK-4) is a serine/threonine kinase pivotal to Toll-like-receptor (TLR) and IL-1 receptor (IL-1R) signalling. Upon TLR/IL1-R activation, IRAK-4 is recruited to the myddosome complex and assembles with MyD88 via its death domain (DD), leading to activation of IRAK-1, NF-κB, AP-1 and release of proinflammatory cytokines. In humans, bi-allelic mutations in *IRAK4* are associated with IRAK-4 deficiency and increased susceptibility to pyogenic bacterial infections.

**Objectives:** We describe 5 patients from 2 unrelated families with biallelic mutations in *IRAK4* presenting with severe systemic autoinflammation, massive splenomegaly, anaemia and neuroinflammation, in the absence of immunodeficiency.

**Methods:** We performed whole-exome sequencing (WES), targeted gene sequencing, protein expression studies, structural modelling and *in vitro* functional studies of mutated IRAK-4 including L-selectin shedding assays and NF-κB phosphorylation assays. Serum and CSF cytokines were quantified by immunoassay.

**Results:** Affected patients presented with recurrent fever, massive splenomegaly, persistently elevated inflammatory markers and microcytic hypochromic anaemia, requiring blood transfusions in 3/5 patients. Expanded erythropoiesis was observed in bone marrow. Infectious screens were negative and immunology was normal, except elevated IgG. 3/5 patients developed evidence of neuroinflammation on brain MRI and 2/5 experienced seizures. WES revealed compound heterozygous (nonsense+missense) mutations in *IRAK4:* (c.C877T (p.Q293*)/c.G958T (p.D320Y) in 2 patients; and c.A86C (p.Q29P)/c.161+1G>A) in 3 patients, respectively, from different families. IRAK-4 protein expression and TLR-agonist induced L-selectin shedding was reduced, but not absent. Elevated cytokines (TNF, IL-1β, IL-6, IL-8, IFN-α2a, and IFN-β) were detected in patient sera and elevated IL-6 in the CSF of 2/5 patients. *In vitro* NF-κB signalling was unaffected. Modelling of missense mutations p.Q29P (DD) and p.D320Y (KD) predicted increased contact distance and disrupted hydrogen-bond interactions with IRAK-1/2 in the myddosome complex, and stabilisation of the kinase active site, respectively. Blockade of IL-6 with toculizumab resulted in complete amelioration of systemic autoinflammation and anaemia in 3/5 patients treated. Neuroinflammation was recalcitrant to both IL-6 and baricitinib JAK inhibition, likely due to lack of CNS penetration.

**Conclusion:** We describe a novel, severe autoinflammatory phenotype which we have called NASA (neuroinflammation, systemic autoinflammation, splenomegaly, and anemia), caused by bi-allelic mutations in *IRAK4,* suggesting that IRAK-4 dysfunction in humans is not limited to immune deficiency. The mechanism of autoinflammation in NASA remains unclear, though we speculate that loss of negative regulation of IRAK-4/IRAK-1 or dysregulation of myddosome or kinase active site dynamics drives dysregulated IL-6 and TNF production.

**Date of birth:** avril 28,

**Patient Consent**

Yes, I received consent

**Disclosure of Interest**

None Declared

**Reference**

1. Cooray, S. *et al.* (2023) Neuroinflammation, autoinflammation, splenomegaly and anemia (NASA) caused by bi-allelic mutations in *IRAK4*. *Frontiers Immunol*. 14:1-16. 10.3389/fimmu.2023.1231749.

### New diseases

#### PT63 Expanding the phenotype of early-onset familial Behcet’s-like disease related to RelA haploinsufficiency

##### Vidya Sivaraman^1,2^, Shoghik Akoghlanian^1,2^, Sonja Chen^2,3^, Ross Maltz^1,2^, Vinay Prasad^2,3^, Rajitha D. Venkatesh^1,2^, Ruth Chan^4^, Jasmine Thai^4^, Catherine Chung^5^, Esteban Fernandez Faith^2,6^, David Rogers^2,7^, Tyler Rogerson^3^, Joanne Drew^1^, Luis Saca^8^, Ofer Zimmerman^9^, Fabian Hauck^10^, Roshini S. Abraham^3^

###### ^1^Pediatrics, Nationwide Children’s Hospital; ^2^The Ohio State University; ^3^Pathology, Nationwide Children’s Hospital; ^4^Medicine; ^5^Dermatology, The Ohio State University; ^6^Dermatology; ^7^Ophthalmology, Nationwide Children’s Hospital, Columbus; ^8^Internal Medicine, Loma Linda University, Loma Linda; ^9^Medicine, Washington University in St. Louis, St. Louis, United States; ^10^Internal Medicine, University of Munich, Munich, Germany

####### **Correspondence:** Vidya Sivaraman

*Pediatric Rheumatology 2024*, **22(2):**PReS24-ABS-1401

**Introduction:** Early-onset Behcet’s-like disease is associated with autosomal dominant pathogenic variants in *RELA*, encoding the p65 subunit of the canonical NFkB pathway. This causes impaired NFkB activation downstream of TLR and TNF stimulation, impaired upregulation of antiapoptotic genes, and increased stromal cell apoptosis. In contrast to other autoinflammatory diseases, RelA haploinsufficiency is characterized by increased inflammatory cytokine signaling, resulting in mucocutaneous ulceration, defective stromal and epithelial cell survival and impaired RelA function. We previously described a familial cohort with RelA haploinsufficiency presenting with oral and genital ulcers, uveitis, episcleritis and skin manifestations.

**Objectives:** Our objective was to elucidate the clinical phenotype of RelA haploinsufficiency across pediatric and adult patients.

**Methods:** Patients with mucocutaneous inflammatory disease were identified either via genome sequencing or through clinical investigations and targeted gene sequencing. All patients in the cohort were identified to have a monoallelic RELA variant. Clinical data was captured as part of a global RelA registry.

**Results:** Eight adult and pediatric patients were identified (4 males, 4 females; age at diagnosis: 11months - 20 years). Early-onset recurrent oral ulcers before age 5 are a hallmark in the majority of these patients. Gastrointestinal symptoms were prominent, with diarrhea being a presenting symptom in 1/2 of patients. Endoscopic findings included colonic inflammation, focal active ileitis and microgranulomas, consistent with an inflammatory bowel disease (IBD) pattern. Further, variable esophageal mucosal eosinophilia (range: 14-25 intraepithelial eosinophils/hpf) was reported in 37% patients. Failure to thrive requiring growth hormone was also observed in several patients. Cutaneous findings during disease flares include erythema nodosum, Gianotti Crosti-like lesions and nonspecific eruptions. Skin pathology showed interface dermatitis or superficial to deep dermal perivascular chronic inflammatory infiltrate, including prominent eosinophils and rare neutrophils. Ophthalmology findings include episcleritis and uveitis. We have not seen neuromyelitis optica (NMO) in our cohort reported by others. Two adult females were diagnosed with cervical carcinoma. Steroid-sparing treatments included colchicine, TNF inhibitors, IL-1 inhibitors and topical therapies, such as dapsone and calcineurin inhibitors for skin lesions.

**Conclusion:** We broaden the previously reported clinical phenotype of RelA haploinsufficiency to include IBD, esophageal eosinophilia and potentially susceptibility to malignancy. Due to the variations in presentation, accurate diagnosis requires correlation with clinical, histologic and immunologic phenotype, and genetic confirmation. Awareness of the overlap with rheumatological and gastrointestinal diseases, along with early-onset and familial disease should improve diagnostic identification. The penetrance of this disorder is not yet ascertained. The creation of a global registry will contribute to identification of additional patients, improving recognition, diagnosis and clinical management of the disease.

**Trial registration identifying number:** N/A

**Patient Consent**

Yes, I received consent

**Disclosure of Interest**

None Declared

### Scleroderma and related syndromes

#### PT64 Comparative analysis of nailfold capillaroscopic alterations in children with Raynaud’s phenomenon and healthy controls

##### Gülşah Kavrul Kayaalp, Selen Duygu Arık, Özlem Akgün, Bengisu Menentoğlu, Ayşenur Doğru, Figen Çakmak, Nuray Aktay Ayaz

###### Department of Pediatric Rheumatology, Istanbul University, Istanbul Faculty of Medicine , Istanbul, Türkiye

####### **Correspondence:** Gülşah Kavrul Kayaalp

*Pediatric Rheumatology 2024*, **22(2):**PReS24-ABS-1742

**Introduction:** Nailfold capillaroscopy has emerged as a standard procedure in rheumatology, proving particularly valuable for identifying specific patterns associated with systemic sclerosis (SSc). It is recommended to conduct nailfold capillaroscopic assessments for all pediatric patients presenting with Raynaud’s phenomenon (RP), alongside antinuclear antibody (ANA) tests. However, data on capillaroscopy in children remains limited, and it’s recognized that capillaroscopic parameters can vary with age.

**Objectives:** It was aimed to assess nailfold capillaroscopic findings in patients referred to the pediatric rheumatology outpatient clinic with RP in a standardized manner and compare them with those of healthy controls to identify RP related capillaroscopy patterns.

**Methods:** The study included 61 patients aged 0-18 years who had been referred to the pediatric rheumatology clinic with a diagnosis of RP, and 44 age and sex-matched healthy controls. Patients with any conditions of RP related to non-rheumatological disorders were excluded. Capillaroscopic evaluation and definitions were made in accordance with EULAR study group on microcirculation in rheumatic diseases (EULAR SG MC/RD) and the Scleroderma Clinical Trials Consortium’s 2020 recommendations for standardization of nailfold capillaroscopy for patients with RP. Capillaroscopic patterns of 58 patients with primary Raynaud’s phenomenon (RP), who remained free of rheumatological diseases during follow-up, were compared with those of healthy controls.

**Results:** Among the 61 patients referred with RP, one patient was diagnosed with SSc, and two patients with systemic lupus erythematosus. The mean age of the 58 patients with primary RP included in the further analysis was 13.83 ± 2.05 years, with 63.8% being female. The most prevalent symptom accompanying RP was arthralgia, affecting 11 patients (18.96%), extending beyond the fingers and toes. The most common non-musculoskeletal finding was recurrent oral aphthae affecting 4 patients (6.89%). Family history was positive for RP in 3 patients (5.17%). Anti-nuclear antibody (ANA) was positive in 7 patients (12.07%), while anti-ENA antibodies were negative in all cases. Sixteen patients (27.59%) required medication due to persistent symptoms despite protection measures against triggers. Capillaroscopic analysis revealed a significantly larger mean apical loop diameter in RP patients compared to healthy controls (18.83± 4.49 μm vs. 15.75± 2.73 μm, p<0.001). Capillary density did not differ between groups, while RP patients exhibited a higher prevalence of dilated (p<0.001), abnormal capillaries (p<0.001) and microhemorrhages (p<0.001), as well as the normal capillary variations including crossing (p<0.001), and tortuous (p=0.002) capillaries. Giant capillaries were present in 3 RP patients (5.2%), whereas none were found in healthy controls (p=0.257). While the predominant overall capillaroscopy pattern in RP patients was non-specific pattern (58.6%), it appeared as normal pattern (86.4%) in healthy controls (p<0.001). Early scleroderma pattern was observed in 3 RP patients (5.2%) whereas no scleroderma pattern was detected in healthy controls. No correlation was observed between medication use for RP and ANA positivity, capillaroscopy patterns, or initial laboratory values.

**Conclusion:** This study demonstrated that patients with primary RP exhibited a distinct capillaroscopy pattern. Extended follow-up studies are needed to determine the proportion of these patients who may later develop secondary RP, and to explore how capillaroscopic findings evolve over time.

**Date of birth:** octobre 27

**Patient Consent**

Not applicable (there are no patient data)

**Disclosure of Interest**

None Declared

### Scleroderma and related syndromes

#### PT65 Efficacy of tocilizumab on disease activity in patients with juvenile localized scleroderma: a retrospective monocentric study

##### Angela Aquilani, Rebecca Nicolai, Fabrizio De Benedetti, Emiliano Marasco

###### Rheumatology, Ospedale Pediatrico Bambino Gesù, Rome, Italy

####### **Correspondence:** Emiliano Marasco

*Pediatric Rheumatology 2024*, **22(2):**PReS24-ABS-1415

**Introduction:** Biologic agents have been proposed as potential therapeutic options for juvenile localized scleroderma (JLS) refractory to prior therapy. Recently, some case series reported the effectiveness of tocilizumab (TCZ) in these patients.

**Objectives:** To evaluate the efficacy of tocilizumab in JLS patients resistant or intolerant to previous therapy (as second- or third-line therapy) and as first line in patients with severe disease.

**Methods:** We enrolled 10 patients with JLS (circumscribed, linear, pansclerotic and mixed scleroderma) followed at Bambino Gesù Children’s Hospital, who received TCZ for at least 2 years. The modified Localized Scleroderma Activity Index (mLoSSI) and the Localized Scleroderma Skin Damage Index (LoSDI), PGA-disease activity and PGA-damage were retrieved from patients’ charts and used to assess disease activity and damage, respectively.

**Results:** All patients were treated with methotrexate (MTX). Four patients with extensive skin involvement were started on a combination of TCZ + MTX as first line therapy. Six patients were started on TCZ as second-or third-line therapy (4 for refractory disease and 2 for MTX-related side effects), three of these patients had received rituximab (RTX) before TCZ. In the 6 patients who started TCZ as second or third-line therapy, we did not observe statistically significant differences for mLoSSI and PGA activity between baseline (pre-MTX) and pre-TCZ. Two patients showed a reduction in both mLoSSI and PGA activity while receiving MTX treatment but had to discontinue MTX due to side effects. The other 4 patients showed stable or worsening mLoSSI and PGA activity. One year after starting TCZ, all 10 patients showed a reduction in both mLoSSI and PGA activity. The improvement further progressed after 2 years of treatment and overall there was a statistically significant reduction in both mLoSSI (Friedman test p= 0.00013, Kendall W coefficient = 0.814) and PGA activity (Friedman test p= 0.00013, Kendall W coefficient = 0.814). No significant differences in LoSDI and PGA damage between baseline (pre-MTX) and pre-TCZ were observed in the 6 patients who started TCZ as second-or third-line therapy. After starting TCZ, patients did not show a significant reduction in neither LoSDI nor PGA damage after 1 or 2 years of therapy.

**Conclusion:** TCZ effectively reduced disease activity in JLS patients who were resistant or intolerant to prior therapies, but it did not reverse existing damage. Thus, TCZ may represent a possible therapeutic option for patients with severe or refractory JLS. We hypothesize that the irreversible damage associated with extensive tissue sclerosis is secondary to an ongoing skin inflammatory response and an earlier use of TCZ may be useful in promptly abating skin inflammation and preventing chronic outcomes and disability.

**Patient Consent**

Yes, I received consent

**Disclosure of Interest**

None Declared

### Scleroderma and related syndromes

#### PT66 DNA methylation profiles in immune cell subsets from juvenile systemic sclerosis patients identify disease-associated pathways

##### Francesca Sposito^1^, Jenny Hawkes^1^, Amandine Charras^1^, Kathryn S. Torok^2,3,4^, Clare E. Pain^1,5^, Christian M. Hedrich^1,5^

###### ^1^Department of Women’s and Children’s Health, Univeristy of Liverpool, Liverpool, United Kingdom; ^2^Division of Rheumatology, Department of Pediatrics, Children’s Hospital of Pittsburgh, University of Pittsburgh; ^3^Clinical and Translational Science Institute; ^4^Systemic Sclerosis Center of Research Translation, University of Pittsburgh, Pittsburgh, PA, United States; ^5^Department of Paediatric Rheumatology, Alder Hey Children’s NHS Foundation Trust Hospital, Liverpool, United Kingdom

####### **Correspondence:** Francesca Sposito

*Pediatric Rheumatology 2024*, **22(2):**PReS24-ABS-1724

**Introduction:** Juvenile systemic sclerosis (jSSc) is a rare and complex autoimmune/inflammatory disease that affects connective tissues resulting in organ sclerosis and failure. The pathophysiology of jSSc is multifactorial and, due to its rarity, poorly understood. Both innate and adaptive immune cells, particularly monocytes and T lymphocytes have been suggested to play an important role in jSSc.

**Objectives:** To investigate epigenetic and gene expression signatures in immune cell subsets from jSSc patients that may serve as future biomarkers and/or treatment targets.

**Methods:** This study is part of the PASTIES project: PAtient STratification and Individualised trEatment in Systemic sclerosis. Peripheral blood mononuclear cells from jSSc patients (N=14) and matched healthy controls (HC, N=17) were isolated from venous blood. Cells were stained for CD3, CD4, CD8, CD14, CD19, and CD86, and immune cell populations (CD3^-^CD19^+^, CD3^+^CD4^+^, CD3^+^CD8^+^, CD14^+^, CD14^+^CD86^+^) were collected using flow cytometry sorting. CD3^+^CD4^+^ T lymphocytes, CD14^+^ monocytes, and CD3^-^CD19^+^ B lymphocytes were collected and used for DNA methylation analysis via Illumina Infinium 930K MethylationEPIC Arrays (Diagenode). Flow cytometry data were analysed using FlowJo Software (v10.6.1, BD Life Sciences); DNA methylation data were analysed using the R environment (v4.4.0) and ChAMP, minfi, limma, and clusterProfiler packages.

**Results:** No statistically significant differences were seen in the proportions of monocytes, B or T lymphocytes between jSSc patients (N=14) and matched controls (N=17). More than 12,500 differentially methylated positions (DMPs) were detected when comparing CD4^+^ T lymphocytes from the two groups (N=4 each), more than 2,500 when comparing monocytes (N=5 each), and more than 1,300 when looking at B cells (N=4 jSSc and 6 HC). KEGG pathway analysis of genes with at least two differentially methylated positions (DMPs) in their promoter region delivered hypermethylation of genes involved in “cell cycle”, “longevity regulating pathway”, “AMPK signalling pathway”, “ubiquitin mediated proteolysis”, and “FoxO signalling pathway” in T cells, “longevity regulating pathway”, “fatty acid metabolism”, and “insulin resistance and signalling pathways” in monocytes, and “homologous recombination” in B cells.

**Conclusion:** Differentially methylated genes in immune cell subsets inform our understanding of jSSc pathophysiology. In a next step, RNA from collected immune cells will be used towards RNA sequencing to integrate gene expression patterns with DNA methylation signatures. Additional samples will be used for validation of findings (NanoString custom RNA expression panels; DNA bisulfite pyrosequencing). Differentially methylated and expressed genes and pathways may inform the development of biomarkers and future treatments of jSSc.

**Patient Consent**

Yes, I received consent

**Disclosure of Interest**

None Declared

### Genetics, genomics and environment

#### PT67 Validation of Human Phenotype Ontology (HPO) terms using the eurofever registry: the Odino project

##### Caterina Matucci-Cerinic^1^, Giovanni Fiorito^2^, Roberta Caorsi^1^, Marielle E. Van Gijn^3^, Guilaine Boursier^4^, Seth Masters^5,6^, Eugenia Mosci^7^, Paolo Uva^2^, Marco Gattorno^1^

###### ^1^IRCCS Istituto Giannina Gaslini, Rheumatology and Autoinflammatory diseases; ^2^IRCCS Istituto Giannina Gaslini, Clinical Bioinformatics Unit, Genoa, Italy; ^3^Department of Genetics, University of Groningen, University Medical Center Groningen, Groningen, Netherlands; ^4^Institute for Regenerative Medicine and Biotherapy, Inserm, U1183, University of Montpellier; Department of Molecular Genetics, Medical Genetics of Rare and Autoinflammatory Disease Unit, Montpellier University Hospital, Montpellier, France; ^5^Inflammation Division, The Walter and Eliza Hall Institute of Medical Research; Department of Medical Biology, The University of Melbourne, Parkville; ^6^Centre for Innate Immunity and Infectious Diseases, Hudson Institute of Medical Research; Department of Molecular and Translational Science, Monash University, clayton, Australia; ^7^IRCCS Istituto Giannina Gaslini, Gaslini Trial Center, Genoa, Italy

####### **Correspondence:** Caterina Matucci-Cerinic

*Pediatric Rheumatology 2024*, **22(2):**PReS24-ABS-1799

**Introduction:** The HPO project (Human Phenotype Ontology) represents the creation of a terminology database for phenotypical, clinical and laboratory characteristics of genetic diseases. In 2022 the AutoInflammatory diseases section has been revised and updated (1), but the accuracy of the new terms has not yet been validated in real patients.

**Objectives:** to validate the HPO terms in a cohort of real patients, in order to evaluate their diagnostic accuracy and to implement the database with missing terms

**Methods:** the clinical variables have been extracted from the Eurofever registry and codified with their HPO matching terms. Every patient in Eurofever with an autoinflammatory disease has been recoded with the HPO corresponding to his phenotype. To evaluate the phenotypical similarity between the HPO terms assigned to every patient in Eurofever and the HPO already linked to that disease, computerized tools of graph-aware distance were used.

**Results:** 224 variables from EF were considered and codified into one or more HPO codes (as appropriate)**:** 215 clinical, 5 laboratory and 4 demographic terms. The variables without any HPO corresponding term were retained. Specifically, a full correspondence Eurofever-HPO was found for 195 terms, a partial one for 12 and no correspondence for 17. Of these, the time lenght of the fever and the ethnicity of the patients were the most important missing variables.

The clinical variables of 3650 patients present in the Eurofever registry were then reconverted using HPO codes. The phenotypic similarity between the HPO terms assigned to each patient in EF and all the HPO annotated diseases was then calculated using graph-aware distances (R ontology Similarity) to estimate the discriminative power of HPO in assigning a patient phenotype to the correct diagnosis. Using the frequencies of clinical manifestations observed in the Eurofever dataset, we applied four clustering algorithms: Multiclass elastic net penalized regression, K nearest neighbors, Random forest, and Gradient Boosting (XG-Boost). Preliminary findings reveal that Multiclass elastic net penalized regression outperform other clustering algorithms with an average accuracy higher than 0.70. Our preliminary findings suggest that clustering patients using HPO terms differ significantly from cluster analysis based on the actual frequency of clinical manifestations observed in the Eurofever registry. This discrepancy arises from differences in the frequencies of clinical variables reported by HPO and based on the literature, compared to their frequency in a real-life dataset provided by Eurofever.

**Conclusion:** additional analyses are needed to improve classification accuracy and identification of the most relevant symptoms for each disease. Moreover, the HPO terminology needs an implementation with the missing terms, and a revision of the codes associated to each disease.

**Patient Consent**

Not applicable (there are no patient data)

**Disclosure of Interest**

None Declared

**Reference**

1. Haimel M, et al. Curation and expansion of Human Phenotype Ontology for defined groups of inborn errors of immunity. J Allergy Clin Immunol. 2022 Jan;149(1):369-378.

### Spondyloarthritis (SpA) and enthesitis related arthritis (ERA)

#### PT68 Clinical characteristics and disease outcomes of musculoskeletal extraintestinal manifestation in pediatric patients with IBD: a nationwide study on behalf of the Italian society of gastroenterology, hepatology and nutrition

##### Valerio Maniscalco^1,2^, Luca Scarallo^3,4^, Edoardo Marrani^1^, Ilaria Maccora^1,4^, Maria Vincenza Mastrolia^1,4^, Ilaria Pagnini^1^, Paolo Lionetti^3,4^, Gabriele Simonini^1,4^

###### ^1^Rheumatology Unit, Meyer Children Hospital IRCCS, Florence; ^2^Pediatrics Department, Santo Stefano Hospital, Prato; ^3^Gastroenterology and Nutrition Unit, Meyer Children Hospital IRCCS; ^4^NEUROFARBA department, University of Florence, Florence, Italy

####### **Correspondence:** Valerio Maniscalco

*Pediatric Rheumatology 2024*, **22(2):**PReS24-ABS-1390

**Introduction:** Musculoskeletal (MSK) Extraintestinal Manifestations (EIMs) are frequent in Inflammatory Bowel Diseases (IBD) and they can have a significant impact on morbidity of IBD patients [1]. Although knowledge and focus on EIM is increasing, data on prevalence, characterization, and clinical course of pediatric MSK EIM are limited [2].

**Objectives:** Aim of the study was to assess the prevalence and to characterize IBD-related MSK EIMs and to assess articular-related outcomes in a nationwide cohort of pediatric IBD patients.

**Methods:** We collected data of pediatric IBD patients experiencing articular EIMs from the Italian national IBD registry. We gathered baseline and one-year follow-up data from concomitant IBD and arthritis diagnosis.

**Results:** 150 patients [(99 Crohn’s Disease (CD), 51 Ulcerative Colitis (UC) and Unclassified IBD (IBDU)] with MSK EIMs out of 3061 (1301 CD and 1760 UC) patients were identified, with an overall prevalence of 4.9%. Peripheral arthritis was present in 84% of patients, mainly oligoarticular, and affecting large joints. Axial arthritis was present by 42% of the cohort. MSK EIMs were more frequent in CD than in UC (7.6% vs 2.9%, p<0.01). Patients with CD had more frequently concomitant MSK EIM diagnosis than those affected by UC, where EIMs were more frequently observed after IBD diagnosis. Peripheral arthritis was more frequently diagnosed in patients with active IBD than in those with quiescent disease (94.6% vs 67.3%, p < 0.01). At one-year follow-up, articular remission was achieved more frequently in patients with peripheral arthritis than in those with axial involvement (69.9% vs 50.6%, p<0.01). Clinically active IBD was independently associated with lower peripheral arthritis remission and no impact on axial arthritis activity has been detected. The presence of additional EIMs was associated with lower IBD clinical remission rates.

**Conclusion:** We characterized MSK manifestations in the largest pediatric IBD cohort. MSK EIMs were more frequently observed in CD than UC and/or IBDU. Peripheral arthritis, particularly oligoarthritis, was the most frequent articular manifestation. Active intestinal inflammation had a negative impact on peripheral arthritis remission but apparently had no effect on axial arthritis-related outcomes. The coexistence of other EIM are associated with worse articular as well of poorer intestinal outcomes.

**Trial registration identifying number:** Not applicable

**Date of birth:** juin 10, Y

**Patient Consent**

Yes, I received consent

**Disclosure of Interest**

None Declared

**References**

1. Harbord M, Annese V, Vavricka SR, Allez M, Barreiro-de Acosta M, Boberg KM, et al. The First European Evidence-based Consensus on Extra-intestinal Manifestations in Inflammatory Bowel Disease. J Crohns Colitis 2016;10:239–54.
2. Ali A, Schmidt M, Piskin D, Crowley E, Berard R. Epidemiology of Musculoskeletal Manifestations in Pediatric Inflammatory Bowel Disease: A Systematic Review. ACR Open Rheumatol 2022;4:547–54.

### Spondyloarthritis (SpA) and enthesitis related arthritis (ERA)

#### PT69 Russian multicenter study of clinical features of entesitis-related arthritis and/or juvenile ankylosing spondylitis

##### Tatiana Y. Kriulina^1,2,3^, Ekaterina I. Alexeeva^1,2,3^, Irina P. Nikishina^3,4^, Elena S. Zholobova^2,3^, Mikhail M. Kostik^3,5^, Tatiana M. Dvoryakovskaya^1,2,3^, Olga M. Kostareva^4^, Alia N. Arefyeva^4^, Olga G. Sukhov’eva^2^, Lubov S. Sorokina^5^, Regina P. Arkhipova^1^, Maria S. Botova^1^, Ksenia B. Isaeva^1^, Elizaveta A. Krekhova^1,2^, Ivan A. Kriulin^1^, Maria Y. Kokina^1,2^, Natalia M. Kondrat’eva^1^, Olga L. Lomakina^1^, Irina T. Tsulukiya^1^, Evgenia G. Chistyakova^1,2^, Christina V. Chibisova^1^, Aleksandra M. Chomakhidze^1^, Meiri S. Shingarova^1,2^, Anna N. Fetisova^1^

###### ^1^Rheumatology, National Medical Research Center of Children’s Health; ^2^Paediatric, Sechenov First Moscow State Medical University; ^3^Association of Pediatric Rheumatologists; ^4^Paediatric, V.A. Nasonova Scientific and Research Institute of Rheumatology, Moscow; ^5^Paediatric, Saint-Petersburg State Pediatric Medical University, Saint-Petersburg, Russian Federation

####### **Correspondence:** Tatiana Y. Kriulina

*Pediatric Rheumatology 2024*, **22(2):**PReS24-ABS-1428

**Introduction:** Among all categories of juvenile idiopathic arthritis according to the classification ILAR there is a variant of arthritis associated with enthesitis (EAA), which may correspond to the prespondylitic stages of juvenile ankylosing spondylitis (JAS) and be a dominant part of the spectrum of juvenile spondyloarthropathies (JSpA), which unites a group of clinically and pathogenetically similar diseases. The need to meet the criteria of EAA, mutually exclusive with respect to the category of psoriatic arthritis, incomplete compliance with the diagnostic criteria of AS make it difficult to identify JSpA in children. The relevance of the study is due to the ambiguity of existing nomenclatures and definitions defining the group of diseases belonging to JSpA.

**Objectives:** In real clinical practice, analyze the clinical features of the course of JSpA diseases in children in the Russian Federation with assessment of formal compliance with existing classification and diagnostic criteria (modified New York criteria for JAS)

**Methods:** A multicentre, observational, observational cross-section study was conducted. Patients who, in the opinion of the physician, could be classified as JAS or EAA were included in the study. A ‘historical’ (over 18 years of age at the time of the study) and prospective cohort of patients were analyzed. Descriptive statistics of the study results for qualitative and ordinal characteristics are presented in the form of absolute values and percentages (%), quantitative - with the median (Me) (IQR).

**Results:** 402 patients were included, 76% were boys, Me age of onset was 11 (8;13) years, Me duration of diagnosis was 7 (3;15.5) months, Me duration of disease was 64 (39;95) months.The clinical picture of EAA/UAS in the patients in the study is represented by: spinal column symptoms in 238/402(59%) (predominant symptom is spinal stiffness in 114/238 (48%) cases), axial skeletal structures in 75/402 (19%), arthritis in 371/402 (92%), entheses in 110/402 (27%), eyes (uveitis) in 26/402 (6%), gastrointestinal tract (unspecified colitis, Crohn’s Disease, ulcerative colitis) in 49/402 (12%), cardiovascular system in 4/402 (1%) patients respectively, psoriasis was detected in 37/402 (9.2%) patients. The most frequent combinations of clinical symptoms were: spinal column lesion and arthritis - in 90/402 (22%), axial lesion combined with arthritis and enthesitis - 45/402 (11%), arthritis and enthesitis - 21/402 (5%) patients, respectively. There were no symptoms of damage to the respiratory and urinary systems. Radiological diagnostic data were analyzed in 307/402(76%). The prevailing method of radial diagnosis was MRI of sacroiliac joints - the study was performed in 277/307 (90%) patients. Sacroiliitis was verified in 269/307 (88%) patients. There were no differences in clinical manifestation, frequency and spectrum of extraaxial manifestations between the EAA and JAS groups.

**Conclusion:** The peculiarities of clinical manifestations of patients referred to the JSpA group described in our study confirm the possibility of evaluating these diseases within a single nosological group, which indicates the need to develop a unified nomenclature and diagnostic criteria for JAS/EAA with subsequent implementation in real clinical practice.

**Patient Consent**

Yes, I received consent

**Disclosure of Interest**

None Declared

### Treatment

#### PT70 Successful CAR T-cell-therapy in juvenile systemic lupus erythematosus

##### Tobias Krickau^1,2^, Nora Naumann-Bartsch^2,3,4^, Georg Schett^2,5^, Fabian Müller^2,4,6^, Andreas Mackensen^2,4,6^, Markus Metzler^2,3,4^

###### ^1^Paediatrics and Adolescent Medicine, Rheumatology; ^2^Deutsches Zentrum Immuntherapie; ^3^Paediatrics and Adolescent Medicine, Oncology; ^4^Bavarian Cancer Research Center (BZKF); ^5^Department of Medicine 3 - Rheumatology and Immunology; ^6^Department of Medicine 5 - Haematology and Oncology, Friedrich-Alexander-Universität Erlangen-Nürnberg and Uniklinikum Erlangen, Erlangen, Germany

####### **Correspondence:** Tobias Krickau

*Pediatric Rheumatology 2024*, **22(2):**PReS24-ABS-1060

**Introduction:** Lupus nephritis (LN) is one of the most common manifestations of systemic lupus erythematosus (SLE). Children experience higher SLE disease activity compared to adult-onset SLE and permanent hemodialysis is a substantial threat in juvenile-onset SLE. Some patients with juvenile SLE do not respond to conventional treatment and constitute a substantial therapeutic challenge

**Objectives:** To test whether treatment with autologous CD19- chimeric antigen receptor (CAR) T-cells is safe and effective in treatment-resistant juvenile SLE.

**Methods:** We initiated anti-CD19-CAR T-cell therapy in an expanded access program for critically ill patients according to the German *Arzneimittelgesetz, §21/2* and the *Arzneimittel-Härtefall-Verordnung §2*. After informed consent, all immunosuppressive medication was discontinued before leukapheresis. The harvested T-cells were transduced with a previously described lentiviral vector (Miltenyi Biotec) encoding for a second-generation 4-1BB-based CD19-CAR using the CliniMACS Prodigy system.^1^ Lymphodepletion was done before the infusion of 1x10^6 fresh anti-CD19 CAR T-cells per kg body weight. Due to renal insufficieny standard dose of fludarabine (3x25mg/m2) and cyclophosphamide (1x1g/m2) was reduced by 50% in both patients and timed to coincide with ongoing hemodialysis (patient #1).

**Results:** Two patients with treatment-resistant juvenile SLE were treated: One 15-year-old girl with rapidly progressive SLE developing LN WHO IV failing on standard medication including glucocorticosteroids (GCs), hydroxychloroquine (HCQ), azathioprine (AZA), mycophenolat mofetile (MMF), belimumab (BEL) and cyclophosphamide (CYC). Her kidney function deteriorated rapidly resulting in end stage renal disease requiring hemodialysis. One 17-year-old girl with progressive LN (WHO IV+V), persistent hemolytic anemia and hypocomplemtemia failing on GCs, HCQ, AZA, MMF, BEL, abatacept and contraindication for rituximab and high-dose CYC.

Both patients received CAR T-cells. Expansion was measured in the peripheral blood and reached a maximum of 6.6 cells/μl on day +10 in patient #1 and 3.2 cells/μl on day +9 in patient #2. CAR T-cells in patient #1 persisted in lower concentration over the whole monitored period of 10 months after CAR T-cell administration, while disappearing after 14 days in patient #2.

Transient cytopenia due to conditioning treatment, grade I cytokine release syndrome (CRS) and malaise were recorded between days +3 and +7. CRS was treated with antipyretics and in patient #1 additionally with a single infusion of tocilizumab. SLE manifestations like arthritis, rash and hemolytic anaemia completely ceased, complement factor 3 level normalized within 4 weeks (patient #2) and 6 weeks (patient #1). Anti-double-stranded-DNA disappeared within 2 months. Renal function improved dramatically in patient #1 and hemodialysis could be stopped 3 weeks after CAR T-cell therapy.^2^ Patient #2 showed a consistent serum creatinine level of 0.8 mg/dL. In contrast, proteinuria decreased significantly from 4.9 g/g creatinine to 0.2 g/g creatinine.

**Conclusion:** CD19-CAR T-cell therapy can induce drug-free remission in juvenile SLE. Even more importantly, the treatment rescued patient#1 from chronic hemodialysis. Moreover, CD19-CAR T-cell therapy was well tolerated in both adolescents suggesting the feasibility of this treatment in severe SLE.

**Patient Consent**

Yes, I received consent

**Disclosure of Interest**

T. Krickau Consultant with: consulting fees from Novartis and Pfizer, Speaker Bureau with: speaker honoraria from Novartis, Pfizer, and Kiowa Kirin, N. Naumann-Bartsch: None Declared, G. Schett Speaker Bureau with: speaker honoraria from Novartis, Bristol Myers Squibb (BMS), Kyverna, and Cabaletta, F. Müller Grant / Research Support with: grants from Kite/Gilead; research funding from Deutsche Krebshilfe (grant No 0113695), Consultant with: consulting fees from AbbVie, ArgoBio, AstraZeneca, BMS, Crispr Therapeutics, Janssen, Kite, and Novartis, Speaker Bureau with: speaker honoraria from AbbVie, ArgoBio, AstraZeneca, BMS, Crispr Therapeutics, Janssen, Kite, Kyverna, Miltenyi Biomedicine, Novartis, and Sobi, A. Mackensen Grant / Research Support with: grants from Miltenyi Biomedicine and Kyverna, Consultant with: grants from Miltenyi Biomedicine and Kyverna, Speaker Bureau with: speaker honoraria from BMS/ Celgene, Kite/Gilead, Novartis, and Miltenyi Biomedicine, M. Metzler Speaker Bureau with: participating in advisory boards from Novartis

**References**

1. Mackensen A, Muller F, Mougiakakos D, et al. Anti-CD19 CAR T cell therapy for refractory systemic lupus erythematosus. *Nat Med* 2022; **28**(10): 2124-32.
2. Krickau T, Naumann-Bartsch N, Aigner M, et al. CAR T-cell therapy rescues adolescent with rapidly progressive lupus nephritis from haemodialysis. *Lancet*. 2024;**403**(10437):1627-1630.

### Treatment

#### PT71 Flares after withdrawal of anti-tumor necrosis factor therapy in patients with non-systemic juvenile idiopathic arthritis

##### Irina Tsulukiya^1^, Ekaterina Alexeeva^1,2^, Tatyana Dvoryakovskaya^1,2^, Olga Lomakina^1^, Anna Fetisova^1^, Ivan Kriulin^1^, Elizaveta Krekhova^1,2^, Maria Botova^1^, Tatiana Kriulina^1,2^, Natalya Kondratyeva^1^, Meiri Shingarova^1,2^, Maria Kokina^1,2^, Aleksandra Chomakhidze^1^

###### ^1^Rheumatology, National Medical Research Center of Children’s Health; ^2^Pediatric, Sechenov First Moscow State Medical University, Moscow, Russian Federation

####### **Correspondence:** Ekaterina Alexeeva

*Pediatric Rheumatology 2024*, **22(2):**PReS24-ABS-1464

**Introduction:** Juvenile idiopathic arthritis (JIA) is the most common rheumatic disease of childhood which is based on a chronic autoimmune inflammation. With modern treatment and especially biologic treatment, remission is now a realistic goal for patients. Prolonged treatment of patients with inactive disease may result in unnecessary exposure to adverse effects. Given these data our study has been focused on important question is whether anti-tumor necrosis factor (anti-TNF) therapy can be reduced or even stopped in patients with stable JIA.

**Objectives:** To determine the frequency and time to flare upon withdrawal of anti–tumor necrosis factor (anti-TNF) therapy in patients with non-systemic juvenile idiopathic arthritis

**Methods:** We enrolled 76 patients with clinically inactive JIA who were receiving anti-TNF therapy (59% of whom were also receiving methotrexate [MTX]) were prospectively followed up. If the disease remained clinically inactive for the initial 24 months of the study, anti-TNF was stopped and patients were assessed for flare at 3, 6, 9, 12 and 18 months. In 25 patients the withdrawal of drug was performed abruptly, and in 51 in a progressive way, either by reducing the dose (n=26) or by increasing the interval between doses (n=25). Remission time was evaluated according to the Kaplan-Meier survival curve.

**Results:** The mean duration of anti-TNF therapy was 54 (IQR 40; 74) months. The disease persisted at an inactive stage for a mean 46 (IQR 33; 67) months before anti-TNF therapy was interrupted. 58 cases (76%) relapsed at a mean 12,4 (IQR7,7; 12,9) months after drug discontinuation. The survival curve shows that 76% of the patients continued to have inactive disease at 3 months, 58% at 6 months, 47% at 9 months, 42% at 12 months and 24% at 18 months after drug discontinuation. No significant differences were observed in the time to relapse between the group in whom the drug was tapered in progressive way and the group in whom TNFi was discontinued abruptly (12,6 vs 10 vs 12,6 months, respectively; p>0,05). Similarly, no association was found between the duration of inactive disease prior to drug withdrawal and the time to relapse. The first case of flare was after 2 months, the peak of cases of flares of JIA occurred in the 3rd month after stopping anti-TNF therapy (up to half all cases of flares were noted in the period from the 3rd to the 6th month). Patients who relapsed were started again on anti-TNF therapy and 54/56 (96%) responded satisfactorily. In 2/56 (4%) patients due to the ineffectiveness of repeated prescription of etanercept, a “switch” was made to adalimumab.

**Conclusion:** In this real-practice JIA cohort, flares were frequent, the majority of patients (76%) relapsed after discontinuation of anti-TNF therapy, the probability of remaining symptom-free at 6 months was 58%, and the response to reintroduction of treatment was satisfactory. More research is needed to identify the most effective approaches to withdraw medications and predictors of outcomes.

**Patient Consent**

Yes, I received consent

**Disclosure of Interest**

None Declared

### Treat-to-target

#### PT72 Prospective evaluation of low disease activity state as treatment endpoint in a large cohort of childhood onset systemic lupus erythematosus

##### Ruby Gotch^1^, Robert Wilson^1^, Yumna Ahmed^1^, Ellie Hawkins^1^, Misato Niwa^2^, Coziana Ciurtin^2^

###### ^1^Department of Rheumatology, University College London Hospital NHS Trust; ^2^Centre for Adolescent Rheumatology, University College London, London, United Kingdom

####### **Correspondence:** Coziana Ciurtin

*Pediatric Rheumatology 2024*, **22(2):**PReS24-ABS-1151

**Introduction:**

Childhood onset systemic lupus erythematosus (cSLE) is more severe than the adult phenotype. Little is known about the feasibility to implement target treatment strategies for better cSLE control in adulthood. Treat-to-target (T2T) is focused on tight disease management to prevent long-term damage. PReS endorsed consensus definitions for remission and Lupus Low Disease Activity State (LLDAS) in cSLE (1).

**Objectives:**

To assess whether T2T strategies can be implemented in routine clinical practice, we undertook a prospective cohort quality improvement project (QIP) focused on two aims: 1. Assess the feasibility of implementing routine outcome measures collection over 12 months (Jan 2023-Jan 2024) and 2. Explore prospectively the impact of setting remission/LLDAS as therapeutic target in cSLE during the same period.

**Methods:**

Detailed patient/disease characteristics were collected from a large single-centre young adult cSLE cohort (N=135) as part of a local QIP. SLEDAI-2K/pBILAG scores, as well as physician global assessment (PGA) and PedSDI were collected prospectively at least at two time points over 12 months: prior and after patient-clinician agreement to set an achievable treatment target. Results are presented using descriptive statistics.

**Results:**

During Jan 2023-Jan 2024, 135 young adults with cSLE (mean age 26.5±5.1 years/disease duration 13.5 ±4.8 years; 85.1% females) have been routinely assessed at least twice in our service; 9.6% (N=13) had incomplete assessments/no target discussed; while 122/135 (90.4%) had SLEDAI-2K/PGA, and 92/135 (68.1%) had their global pBILAG score recorded at least twice over the project period. The PedSDI scores were recorded annually and therapeutic targets were discussed at least at two different time-points over the 12 months in 122/135 (90.4%) cSLE patients.

At the last assessment, 17 (13.9%) and 32 (26.2%) were in complete remission off and on treatment, respectively. Additionally, 13 (10.7%) and 39 (31.9%) achieved clinical remission off and on treatment, respectively, while 11 (9%) achieved LLDAS; therefore, 112 (91.8%) patients achieved their set therapeutic target. However, 7/122 (5.7%) experienced cSLE flares, while three patients (2.5%) were unable to decrease their steroid dose as per target agreed. Following the implementation of T2T strategies, 14/122 (11.5%) of young adults with cSLE (previously with active disease/on a higher steroid dose) achieved their agreed target, and 7/122 (5.7%) previously in target achieved an even better therapeutic target. Four patients (3.3%) experienced flares despite being previously in target, and three (2.5%) did not achieved their target as still active/unable to decrease steroids. Because of the small sample size, there were no statistically significant predictors for achieving target vs. flaring over the 12-months period investigated.

**Conclusion:**

This single-centre QIP led to an increase proportion of young adults with cSLE achieving a therapeutic target: from 82.7% (N=101) to 91.8% (N=112) over a 12-month period. This study also provides the much needed evidence that T2T strategies are an achievable goal in young adults with cSLE, as well as preliminary data for a potential clinical trial testing T2T strategies in cSLE.

**Trial registration identifying number:** N/A

**Patient Consent**

Yes, I received consent

**Disclosure of Interest**

None Declared

**Reference**

1. Smith EMD et al., Ann Rheum Dis. 2023 Jun;82(6):788-798.

### Treat-to-target

#### PT73 Does clinician opinion align with operational definitions of childhood-onset systemic lupus erythematosus treat-to-target definitions?

##### Maria Hanif^1^, Chandni Sarker^1^, Andrea L. Jorgensen^2^, Michael W. Beresford^1,3^, Eve M. D. Smith^1,3^ and on behalf of the UK JSLE Cohort Study

###### ^1^Institute of Life Course and Medical Sciences; ^2^Institute of Population Health; ^3^Alder Hey Children’s NHS Foundation Trust Hospital, Liverpool, United Kingdom

####### **Correspondence:** Maria Hanif

*Pediatric Rheumatology 2024*, **22(2):**PReS24-ABS-1195

**Introduction:** Childhood-onset Systemic Lupus Erythematosus (cSLE) treat-to-target (T2T) goals: cSLE Lupus Low Disease Activity (cLLDAS),^1^ cSLE Clinical Remission (cCR) and cSLE Clinical Remission Off steroids (cCR-0) have been derived recently.^2^

**Objectives:** Assess the correlation between clinicians’ assessment of patient disease status and attainment of cSLE T2T definitions, exploring implications for treatment.

**Methods:** Analysis included UK JSLE Cohort Study participants, diagnosed at ≤18 years, who met ≥4 ACR 1997 classification criteria and had data regarding clinicians’ opinion on disease status (no clinical flare, no change in activity, minor flare, moderate flare, and major flare), and therapeutic intention (decrease treatment, increase treatment, change DMARD, no change in treatment). cSLE target attainment status was assessed at each visit. Agreement between clinicians’ assessment of disease status and attainment of T2T definitions was explored via cross-tabulation, with Cohen’s kappa providing a quantitative measure of this. For visits showing disagreement, we assessed if awareness of target attainment may have impacted treatment.

**Results:** 125 patients were assessed over 378 visits. At least one of the T2T goals was attained at 116 visits (31%). Cross-tabulation revealed most clinicians believed their patients’ disease was stable at visits where a target definition was attained (106/116, 91%). Of 262 visits where a target was not met, at 178 (68%) of visits, clinicians believed the patient’s disease was stable. At these visits, 30% had a decrease in treatment and 58% had no change in treatment. Of 84 visits where clinicians did not believe patient’s disease was stable and targets were not met, treatment was increased in 57% of patients. When comparing clinicians’ opinion to operational T2T definitions, Cohen’s kappa was estimated to be 0.17 (95% CI 0.11-0.23).

**Conclusion:** Discordance between clinicians’ assessment of patient disease status and attainment of cSLE T2T definitions suggests operational T2T definitions are more stringent than clinical opinion in defining low disease activity and remission, underscoring the importance of assessing T2T attainment at every visit to enhance treatment. Data indicated while clinicians do not overtreat patients meeting targets, there is a risk of undertreating those with moderate disease activity.

**Date of birth:** 11/24/2001

**Patient Consent**

Yes, I received consent

**Disclosure of Interest**

None Declared

**References**

1. Smith et al, 10.1016/j.clim.2023.109296.
2. Smith et al, 10.1016/j.clim.2024.110214

### Treat-to-target

#### PT74 Predictive factors of relapses after withdrawing biotherapies in children with inactive JIA: a retrospective cohort study

##### Vincent Jallot^1^, Perrine Dusser^1^, Véronique Hentgen^2^, Sébastien Cavelot^2^, Isabelle Kone-Paut^1^

###### ^1^Paediatric Rheumatology, CHU Kremlin-Bicêtre, Bicêtre; ^2^Paediatric Rheumatology, CH Versailles, Versailles, France

####### **Correspondence:** Vincent Jallot

*Pediatric Rheumatology 2024*, **22(2):**PReS24-ABS-1739

**Introduction:** The modalities of stopping biological treatments in JIA-patients after remission period under treatment, are still exploratory. To date, there are only a few articles about the best way to stop the b-DMARDS treatment and in most of them s-DMARDS and b-DMARDS are included and analysed together, whereas in practice, s-DMARDS may be stopped before biological drugs. We would like to identify one or more predictors of relapse after b-DMARDS cessation that would guide adaptation of the withdrawal strategy.

**Objectives:** We aimed to identify one or more predictors of relapse after b-DMARDS discontinuation in JIA patients.

**Methods:** We performed a retrospective chart review of JIA patients who fulfilled the ILAR criteria and stopped their b-DMARDS between 2000 and 2023 in French hospitals. We used data from the JIR cohort, a multicentre international registry created in 2013 to collect data on patients with juvenile inflammatory rheumatic diseases. The definition of remission was based on the Wallace criteria. The primary outcome was defined as either the absence or the presence of a relapse within one year following the b-DMARD treatment cessation due to remission status. A relapse was defined as no longer fulfilling remission criteria within one year after b-DMARD withdrawal.

**Results:** In two main centers, CHU Bicêtre and CH Versailles, 690 patients were treated for JIA during the study period. 120 of them (17.3%) met the inclusion criteria in whom (26 % oligo-JIA, 23% poly-JIA, 12% pso-JIA, 23% ERA, and 16% s-JIA). To date, we analyzed more than 110 patients, two-thirds were girls, with a median age of 5.3 years at diagnosis. Patients received Anakinra, Canakinumab, Tocilizumab, Etanercept, Adalimumab, Infliximab or Baricitinib. All discontinued treatments after a median duration of 2.5 years under b-DMARDS. Around 51% of them flared within one year after treatment discontinuation (the highest relapse rate was in the psoriasis group with a rate of 84%, the lowest in the systemic group with a rate of 29.5%). Positive anti-nuclear antibodies at diagnosis appeared associated with a higher flare probability (p < 0.05); on the contrary, a high CRP at diagnosis was associated with fewer flare-ups within one year after b-DMARD withdrawal (p < 0.05).

We will supplement our results during the meeting with subgroup analyses after processing data from all the centers.

**Conclusion:** Data from our large cohort of JIA patients showed as a whole 50% of flares after b-Dmards discontinuation. So-JIA appeared at the lowest risk of relapse after stopping biologic treatment, as previously reported. Other original data will be added after the end of the statistical analyses.

**Date of birth:** octobre 30

**Patient Consent**

Yes, I received consent

**Disclosure of Interest**

None Declared

### Treat-to-target

#### PT75 Assessment and validation of paediatric treat-to-target endpoints in childhood systemic lupus erythematosus

##### Chandni Sarker^1^, Andrea L. Jorgensen^2^, Michael W. Beresford^1, 3^, Eve M. D. Smith^1,3^ and on behalf of the UK JSLE Cohort Study

###### ^1^Department of Women’s and Children’s Health, Institute of Life Course and Medical Sciences, University of Liverpool; ^2^Department of Health Data Science, Institute of Population Health, University of Liverpool; ^3^Department of Paediatric Rheumatology, Alder Hey Children’s NHS Foundation Trust Hospital, Liverpool, United Kingdom

####### **Correspondence:** Eve M. D. Smith

*Pediatric Rheumatology 2024*, **22(2):**PReS24-ABS-1307

**Introduction:** The Paediatric Rheumatology European Society (PReS) have endorsed consensus derived Childhood Lupus (cSLE) treat-to-target (T2T) goals; including childhood Lupus Low Disease Activity State (cLLDAS) (1), clinical remission on (cCR) and off-corticosteroids (cCR-0) (2).

**Objectives:** To employ a data-driven approach to evaluate how effective the agreed-upon consensus criteria are in protecting against severe flares and new damage accrual in cSLE.

**Methods:** Data from UK JSLE Cohort Study patients, <18 years at diagnosis, with ≥4 ACR criteria for SLE were utilised. Individual criteria of the existing cSLE targets were either removed or varied (9, 6 and 5 variations of the cLLDAS, cCR and cCR-0 targets were assessed, respectively). The impact of these variations was explored by using Prentice-Williams-Peterson (PWP) gap-time models, assessing the hazard ratios (HRs) of severe flare and new damage in those attaining original vs varied target definitions. For a given target, where variations of >1 criterion significantly improved outcomes, we integrated these together into a modified target. Student’s t-test for dependent samples compared the HRs between original consensus-derived cSLE targets and corresponding modified targets on the impact of severe flare and new damage.

**Results:** All consensus-derived cSLE targets significantly reduced the hazards of severe flare (cLLDAS: HR 0.18 [CI 0.14, 0.23], cCR: HR 0.18 [CI 0.13, 0.23], cCR-0: HR 0.17 [CI 0.13, 0.23]) and new damage (cLLDAS: HR 0.22 [CI 0.11, 0.44], cCR: HR 0.25 [CI 0.13, 0.49], cCR-0: HR 0.30 [CI 0.15, 0.60]) (all *p*<0.001). Of the 9 variations of cLLDAS, transformation of SLEDAI-2K cut-off to ≤3 (HR 0.13 [CI 0.09, 0.19]) and transformation of Physician Global Assessment (PGA) cut-off to ≤0.5 (HR 0.15 [CI 0.12, 0.21]) led to significant reduction in the hazards of severe flare compared to the original definition (all *p*<0.001). A modified version of cLLDAS was investigated, integrating both variations to cLLDAS criteria. This demonstrated a significant decrease in hazards of severe flare compared to the original cLLDAS target (HR 0.12 [CI 0.08, 0.17], p<0.001), but had no impact on the hazards of new damage. Regarding the cCR and cCR-0 definitions, no variations to the initial consensus-derived target criteria could improve protection from severe flare or new damage (all p>0.05).

**Conclusion:** Refinement of the cLLDAS criteria, specifically transforming SLEDAI-2K score cut-off to ≤3 and the PGA score cut-off to ≤0.5 could potentially enhance protection against severe flare. However, it does not appear to impact on protection from new damage. Further research is needed to evaluate how effective the agreed-upon consensus criteria are in protecting against severe flares and new damage accrual in international patient cohorts.

**Date of birth:** novembre 2

**Patient Consent**

Yes, I received consent

**Disclosure of Interest**

None Declared

**References**

1. Smith E, et al. PReS-endorsed international childhood lupus T2T task force definition of childhood lupus low disease activity state (cLLDAS). Clinical Immunology. 2023;250:109296.
2. Smith E, et al. Defining remission in childhood-onset lupus: PReS-endorsed consensus definitions by an international task force. Clinical Immunology. 2024:110214.

### Uveitis

#### PT76 Juvenile idiopathic arthritis patients with and without uveitis - differences in medication and characteristics

##### Heidi Rahikkala^1,2^, Henri Salo^3^, Erika Uusitupa^2,4^, Tytti Pokka^1,5^, Johanna Kärki^6^, Johanna Huhtakangas^7,8^, Laura Kuusalo^9,10^, Jarno Rutanen^11^, Vappu Rantalaiho^12,13^, Tuulikki Sokka-Isler^14^, Maria Backström^1,15^, Paula Vähäsalo^1,16^

###### ^1^Research Unit of Clinical Medicine, University of Oulu, Oulu; ^2^Pediatrics, Turku University Hospital, Turku; ^3^Knowledge Brokers Department, Finnish Institute for Health and Welfare, Helsinki; ^4^Pediatrics, University of Turku, Turku; ^5^Research Service Unit, Oulu University Hospital, Oulu; ^6^Children and Adolescents, Wellbeing Services County of Kanta-Häme, Hämeenlinna; ^7^University of Eastern Finland; ^8^Division of Rheumatology, Kuopio University Hospital, Kuopio; ^9^Department of Internal Medicine, University of Turku; ^10^Centre for Rheumatology and Clinical Immunology, Turku University Hospital, Turku; ^11^Faculty of Social Sciences; ^12^Faculty of Medicine and Health Technology, University of Tampere; ^13^Centre for Rheumatic Diseases, Tampere University Hospital, Tampere; ^14^Hospital Nova of Central Finland, Wellbeing Services County of Central Finland, Jyväskylä; ^15^Pediatrics, Wellbeing Services County of Ostrobothnia, Vaasa; ^16^Children and Adolescents, Oulu University Hospital, Oulu, Finland

####### **Correspondence:** Heidi Rahikkala

*Pediatric Rheumatology 2024*, **22(2):**PReS24-ABS-1235

**Introduction:** Approximately 12-20% of juvenile idiopathic arthritis (JIA) patients develop uveitis as an extra-articular manifestation in Nordic countries. Methotrexate (MTX) and monoclonal antibody tumor necrosis factor inhibitors are the most important drugs in the systemic treatment of uveitis.

**Objectives:** The aim of this study was to assess characteristics and changes in medication from 2012 to 2021 in JIA patients with (uJIA) and without uveitis (nuJIA).

**Methods:** Data was collected from The Finnish Rheumatology Quality Register (FinRheuma) on non-systemic onset JIA patients whose age at the latest visit was less than 16.0 years. The presence of uveitis required one of the ICD-10 codes H20.0, H20.1, H20.8, H20.9, H22.1, H30.2 at least once in the FinRheuma register or at least twice in the Care Register of National Institute for Health and Welfare. From the Reimbursement register of the Social Insurance Institution of Finland we analysed the proportions of patients who purchased disease-modifying antirheumatic drug (DMARD) or glucocorticoid eye drop medicines within 6-month period before the latest visit between Jan 2020 and Sep 2021 and separately for each 6-month interval between Jan 2012 and Dec 2021. The difference between nuJIA ja uJIA patients was analysed with logistic regression and adjusted for disease duration and age. Joinpoint analysis was used to analyse annual percent change (APC) of the proportions of purchasers.

**Results:** Out of 2057 JIA patients 395 (19.2%) had uveitis. uJIA was more common in girls than in boys (69.9% vs 30.1%; p=0.002). Anti-nuclear antibody positivity was more common in uJIA than nuJIA patients (59.6% vs 35.4%; p<0.001) and HLA-B27 was more sparsely positive in uJIA than nuJIA patients (19.9% vs 31.6%; p=0.002). MTX was the most common conventional DMARD. MTX and biologic DMARDs (bDMARD) were purchased more often in uJIA than nuJIA patients (61.5% vs 43.1%; p<0.001, 52.7% vs 28.8%; p<0.001). The most purchased bDMARD in uJIA patients was adalimumab (uJIA 44.8% vs nJIA 7.2%; p<0.001). Etanercept was purchased more often in nuJIA than uJIA patients (15.8% vs 3.0%; p<0.001), as well as tosilizumab and abatacept. Systemic glucocorticoids were purchased by 3.0% of uJIA and 5.4% of nuJIA patients (NS). Among uJIA patients, between Jun 2013 and Dec 2021, the number of annual adalimumab purchasers increased (APC 6.1%; 95% CI 5.7 to 6.4), and the number of glucocorticoid eye drop purchasers decreased from Jun 2017 to Dec 2019 (APC 3.5%; 95% CI 0.7 to 6.2) and from Jan 2020 to Dec 2021 (APC 13.0%; 95% CI 9.6 to16.2).

**Conclusion:** The prevalence of uveitis in JIA patients is 19.2%, in accordance with earlier reports from Finland. uJIA patients were treated more often with MTX and bDMARDs, especially adalimumab than nuJIA patients. The need for glucocorticoid eye drops seems to decrease with increasing use of adalimumab.

**Patient Consent**

Not applicable (there are no patient data)

**Disclosure of Interest**

None Declared

### JIA (oligo, poly, psoriatic)

#### PT77 Treat-to-target study in polyarticular JIA comparing three PROKIND approaches

##### Gerd Horneff^1^, Kirsten Minden^2^, Dirk Foell^3^, Jens Klotsche^2^, Ralf Trauzeddel^4^, Prasad Oommen^5^, Daniel Windschall^6^, Ivan Foeldvari^7^, Markus Hufnagel^8^, Ariane Klein^1^, Kirsten Moenkemoeller^9^, Johannes-Peter Haas^10^, Normi Brueck^11^, Jasmin Kuemmerle-Deschner^12^, Sonja Musek^13^, Frank Weller^14^, Klaus Tenbrock^15^

###### ^1^Asklepios, Sankt Augustin; ^2^DRFZ, Berlin; ^3^University, Muenster; ^4^Helios, Berlin; ^5^University, Duesseldorf; ^6^St Josef Stift, Sendenhorst; ^7^Private office, Hamburg; ^8^University, Freiburg; ^9^Kinderkrankenhaus, Cologne; ^10^Kinderrheumazentrum, Garmisch-Partenkirchen; ^11^University, Dresden; ^12^University, Tuebingen; ^13^Private office, Baden-Baden; ^14^Kinderkrankenhaus, Bremen; ^15^University, Aachen, Germany

####### **Correspondence:** Gerd Horneff

*Pediatric Rheumatology 2024*, **22(2):**PReS24-ABS-1246

**Introduction:** National and international guidelines call for rapid and effective treatment of polyarticular juvenile idiopathic arthritis (JIA) to achieve an inflammation-free clinical state as quickly as possible. Nevertheless, the prognosis of many patients remains uncertain, so that an optimisation of the care situation appears necessary.

**Objectives:** The aim of the PROKIND protocols is to improve and harmonise diagnosis, monitoring, treatment decisions and prognoses (Ref. 1).

**Methods:** Prospective treat-to-target observational study of patients with polyarticular JIA during the first year of treatment according to the PROKIND recommendations (1). Acceptability and outcomes of different treatment pathways with treat-to-target strategy for polyarticular JIA are investigated in the GBA-funded project. . Patients with initial therapy with methotrexate form cohort 1, patients with additional repeated intravenous corticosteroid therapy form cohort 2, patients with simultaneous intra-articular corticosteroid application in at least at least 5 joints or multiple repeated injections is cohort 3. Biologics were added if targets were not met (1).

**Results:** 160 pJIA patients (RF+ polyarthritis n=24, RF- polyarthritis n=138, unknown RF status n=8) from 23 paediatric rheumatology institutions in Germany and Austria were recruited, 86/53/31 patients were assigned to cohort 1/2/3. Patients of cohort 2 had higher disease activity (JADAS10), higher functional limitations (CHAQ-DI) and higher CRP and ESR.

The mean JADAS10 showed a decrease in disease activity from 19.2+/-7.7 at baseline to 3.5+/-4.6 at month 12, the decrease in CHAQ-DI from 0.9+/-0.8 to 0.3+/-0.5 showed improvement in functional capacity. Similarly, improvements in quality of life, pain and fatique were demonstrable. JADAS-inactive disease was achieved by 20.8% at month 3, 42.1% at month 6 and 57.7% at month 12.

Escalation to biologic-based therapy was received by 40/86 (47%) of patients in cohort 1 (MTX only), 25/53 (47%) in cohort 2 (MTX+steroid pulses), and 14/31 (45%) in cohort 3 (MTX+extensive i.a. steroids).Treatment escalation to second biologic was used in 10%/9%/10%. Finally, in cohort 1/2/3. A total of 60.4%/48.5% and 66.7.2% had a JADAS inactive disease (JADAS10≤ 2.7) and an additional 25.0%/24.2%/20.8% had a minimal disease activity (JADAS 10 2.8-≤6) and 14.6%/24.2%/12.5 had residual moderate disease activity (JADAS10 6.1-≤17) while only 1 had high disease activity.

**Conclusion:** A treat-to-target approach achieves dramatic improvement in disease activity in polyarticular juvenile idiopathic arthritis. Already after 12 months, in about two thirds of patients inactive disease was achieved with marked improvement in functional limitations and quality of life. However, an additional benefit of repeated steroid pulse therapy or extensive intra-articular steroid injections was not recognizable. Due to the study design data must be interpreted with caution.

**Patient Consent**

Not applicable (there are no patient data)

**Disclosure of Interest**

G. Horneff Grant / Research Support with: MSD, Novartis, Pfizer, Roche, K. Minden: None Declared, D. Foell: None Declared, J. Klotsche: None Declared, R. Trauzeddel: None Declared, P. Oommen: None Declared, D. Windschall: None Declared, I. Foeldvari: None Declared, M. Hufnagel: None Declared, A. Klein: None Declared, K. Moenkemoeller: None Declared, J.-P. Haas: None Declared, N. Brueck: None Declared, J. Kuemmerle-Deschner: None Declared, S. Musek: None Declared, F. Weller: None Declared, K. Tenbrock: None Declared

**Reference**

1. Horneff G, Klein A, Ganser G, Sailer-Höck M, Günther A, Foeldvari I, Weller-Heinemann F. Protocols on classification, monitoring and therapy in children's rheumatology (PROKIND): results of the working group Polyarticular juvenile idiopathic arthritis. Pediatr Rheumatol Online J. 2017 Nov 7;15(1):78

### Services and pathways of care

#### PT78 A comparison of the essential medicines available for rheumatic diseases in children and young people in Africa and the World Health Organisation model lists

##### Waheba Slamang^1^, Christiaan Scott^1, 2^, Helen E. Foster^3^

###### ^1^University of Cape Town, Cape Town, South Africa; ^2^University of Ottawa, Ottawa, Canada; ^3^Newcastle University, Newcastle Upon Tyne, United Kingdom

####### **Correspondence:** Waheba Slamang

*Pediatric Rheumatology 2024*, **22(2):**PReS24-ABS-1326

**Introduction:** Lack of access and availability of appropriate medicines and care for rheumatic diseases in children and young people in low- and middle-income countries has been shown to result in greater damage and disability.^1^ National Essential Medicines Lists (NEML) inform the procurement and supply of medicines for priority diseases identified by National Ministries of Health, and guided by the World Health Organisation Essential Medicines List (WHO EML), serve as important advocacy documents for best practice particularly in the public health sector in these regions.

**Objectives:** This study compares the degree to which the basket of medicines recommended for rheumatic diseases in children and young people in NEML of African countries, corresponds to the 2021 WHO EML and WHO EML for children, as a proxy of the medicines available in public healthcare sectors. Health expenditure per capita, sociodemographic index and the availability of rheumatology services (paediatric and/or adult) which may affect this, are further considered.

**Methods:** Online searches of the WHO medicines and health technology portal, the Health Ministry websites of the 54 countries constituting the WHO Africa region, PUBMED and Google scholar were conducted for NEML. The number of medicines and dosage forms listed were compared to a predefined template based on the 2021 WHO model lists of essential medicines for rheumatic diseases; and the percentage similarity calculated. Descriptive statistics and regression models for the predictors of the number of medicines listed on NEML were derived using STATA.

**Results:** 47 countries in the WHO Africa region had developed an NEML. Eleven countries did not have any medicines listed for rheumatic diseases. Forty-three countries had last updated their NEML before 2021; and 40 countries had less than or equal to 50% similarity with the medicines listed on the 2021 WHO EML, median 3 medicines (IQR 1—4). The most common medicines on the NEML from Africa were methotrexate, sulfasalazine and azathioprine. Biologic disease-modifying anti-rheumatic drugs were available in 8 countries. Seven countries had only one medicine, acetylsalicylic acid listed in the section ‘Juvenile Joint diseases’. A multiple linear regression model for the predictors of the number of medicines on the NEML established that 20% of the variability was predicted by country factors (p=0.006); sociodemographic index (p=0.035, 95% CI 0.64-16.16) and the availability of rheumatology services (p=0.033, 95% CI 0.13–2.90) were significant, with health expenditure per capita not significant p=0.45.

**Conclusion:** Only 4 countries in the WHO Africa region have sufficiently updated their NEML to reflect standard care for children and young people with rheumatic diseases. Efforts should focus on aligning medicines with the WHO EML for standard care; and strengthening paediatric rheumatology education and service delivery in the WHO Africa region for safe, affordable access to care and medicines for children and young people with rheumatic diseases.

This study is limited to NEML that were available online and cannot be extrapolated to the overall availability of medicines in a country.

**Patient Consent**

Not applicable (there are no patient data)

**Disclosure of Interest**

None Declared

**Reference**

1. Consolaro A, et al. Phenotypic variability and disparities in treatment and outcomes of childhood arthritis throughout the world: an observational cohort study. Lancet Child Adolesc Health 2019 Apr;3(4):255–63.

### Spondyloarthritis (SpA) and enthesitis related arthritis (ERA)

#### PT79 HLA-B27 is associated with worse outcome in Juvenile Idiopathic Arthritis (JIA) after 18 years in a long-term Nordic cohort study

##### Maria Ekelund^1, 2^, Agnes Szentpetery^3^, Ellen D. Arnstad^4, 5^, Kristiina Aalto^6^, Anders Fasth^7^, Mia Glerup^8^, Troels Herlin^8^, Charlotte Myrup^9^, Suvi Peltoniemi^10^, Marite Rygg^4, 11^, Veronica G. Rypdal^12, 13^, Ellen Berit Nordal^12, 13^, Lillemor Berntson^1^ and Nordic Study Group of Pediatric Rheumatology (NoSPeR)

###### ^1^Department of Women’s and Children’s Health, Uppsala University, Uppsala; ^2^Department of Pediatrics, Ryhov County hospital, Jönköping; ^3^Department of Medical Sciences, Rheumatology, Uppsala University, Uppsala, Sweden; ^4^Department of Clinical and Molecular Medicine, Norwegian University of Science and Technology, Trondheim; ^5^Department of Pediatrics, Levanger Hospital, Levanger, Norway; ^6^Department of Pediatrics, New Children’s Hospital, Pediatric Research Center, Helsinki University Hospital, Helsinki, Finland; ^7^Department of Pediatrics, Institute of Clinical Sciences, Sahlgrenska Academy, University of Gothenburg, Göteborg, Sweden; ^8^Department of Pediatrics, Aarhus University Hospital, Aarhus; ^9^Department of Paediatrics, Rigshospitalet, Copenhagen University Hospital, Copenhagen, Denmark; ^10^Clinic of Rheumatology, Helsinki University Hospital, Helsinki, Finland; ^11^Department of Pediatrics, St. Olavs Hospital, Trondheim; ^12^Institute of Clinical Medicine, UiT The Arctic University of Norway; ^13^Department of Pediatrics, University Hospital of North NorwayDepartment of Medical Sciences, Rheumatology, Uppsala University, Tromsø, Norway

####### **Correspondence:** Maria Ekelund

*Pediatric Rheumatology 2024*, **22(2):**PReS24-ABS-1215

**Introduction:** We have previously shown that HLA-B27 was negatively associated with remission status eight years after disease onset in JIA, possibly because of its association with clinical disease characteristics, such as sacroiliitis, rather than being a general marker of persistent disease(1).

**Objectives:** We aimed to study if this was still the case 18 years after disease onset

**Methods:** As part of the population-based Nordic cohort study, consecutive cases of JIA with disease onset between 1997 and 2000 from Denmark, Finland, Norway, and Sweden were prospectively included. All patients were invited to participate in a follow-up at 18-years, either with a clinical visit, or with a standardized telephone interview. Demographic and clinical data such as a joint examination and remission status (preliminary Wallace criteria) was collected consecutively at baseline, 8, and 18 years after disease onset and presented in relation to HLA-B27 status.

**Results:** HLA-B27 was assessed in 416 (96%) of 434 included participants, 93 (22.4%) were positive, more commonly in boys (p= 0.01). The HLA-B27 positive individuals were spread across the various JIA categories but as expected, most frequently found in the ERA category (79.5%), and in the undifferentiated category (27.3%). Symptomatic uveitis, inflammatory back pain, palpatory pain of the sacroiliac joints, sacroiliitis on imaging (MRI, CT or plain x-ray) as well as enthesitis during the course of the disease were associated with lower remission rate off medication after 18 years of disease. This was found irrespective of HLA-B27 positivity or gender, but were significantly more common among the HLA-B27 positive individuals. HLA-B27 positivity was associated with higher risk of not being in remission off medication after 18 years, OR= 2.6 (95% confidence interval (CI) 1.5-4.3), significantly higher in males, OR = 5.6 (95% confidence interval (CI) 2.3-13.7) but without significance in females, OR= 1.7. The sacroiliac, hips, tarsal, and subtalar joints were more frequently involved in HLA-B27 positive individuals than in HLA-B27 negative individuals.

**Conclusion:** Clinical features known to be more common in HLA-B27 positive patients were associated with worse outcome 18 years after disease onset irrespective of HLA-B27 positivity and gender but did occur more often in boys. HLA-B27 was associated with an increased risk of not being in remission off medication after 18 years of disease in males but not in females. Our results underline the adverse impact of being HLA-B27 positive on long term outcomes in JIA.

**Patient Consent**

Not applicable (there are no patient data)

**Disclosure of Interest**

None Declared

**Reference**

1. Berntson L, Nordal E, Aalto K, Peltoniemi S, Herlin T, Zak M, et al. HLA-B27 predicts a more chronic disease course in an 8-year followup cohort of patients with juvenile idiopathic arthritis. J Rheumatol. 2013;40(5):725-31.

### e-health and digital health applications

#### PT80 The effect of an immersive virtual reality exercise program (JIAFIT-XR) on physical fitness and functional capacity in adolescents with juvenile idiopathic arthritis: preliminary results of a randomized control trial

##### Nilay Arman^1^, Asya Albayrak^2,3^, Irem Donmez^4^, Yusuf Acikgoz^4^, Asena Yekdaneh^2^, Figen Cakmak^5^, Betul Sozeri^6^, Nuray Aktay Ayaz^7^

###### ^1^Faculty of Health Sciences, Department of Physiotherapy and Rehabilitation; ^2^Institute of Graduate Studies, Physiotherapy and Rehabilitation Doctorate Program, Istanbul University-Cerrahpasa; ^3^Faculty of Health Sciences, Department of Physiotherapy and Rehabilitation, Istanbul Kent University; ^4^Institute of Graduate Studies, Physiotherapy and Rehabilitation Master Program, Istanbul University-Cerrahpasa; ^5^Department of Pediatric Rheumatology, Basaksehir Cam ve Sakura Hospital; ^6^Ümraniye Training and Research Hospital; ^7^Istanbul Faculty of Medicine, Department of Pediatric Rheumatology, Istanbul University, Istanbul, Türkiye

####### **Correspondence:** Asya Albayrak

*Pediatric Rheumatology 2024*, **22(2):**PReS24-ABS-1680

**Introduction:** Juvenile Idiopathic Arthritis (JIA), the most common pediatric inflammatory disease characterized by chronic joint pain, swelling, and limited range of motion, highlights the importance of prescribing physical activity and exercise in this population (1). The combination of exercise training and video games, termed “Exergaming,” merges the competitive aspects and enjoyment of video games with physical activity, potentially reducing perceived exertion while maintaining high energy expenditure and increasing motivation (2). In recent years, exergaming has begun to incorporate immersive virtual reality (IVR) elements to further enhance the user experience (3).

**Objectives:** The aim of the present study was to investigate the effect of a personalized home-based exercise program (HBEP) versus an IVR exergame (JiaFIT-XR) program on physical fitness (PF) and functional capacity in adolescents with JIA.

**Methods:** Seventeen patients with JIA were randomly assigned to an IVR exergame program (n=10) called JiaFIT-XR and the HBEP (n=7). For the JiaFIT-XR program, utilizing the Oculus Quest 3 headset, a virtual reality experience for PF was provided through Fit-XR games including “Boxing”, “HIIT”, “Combat”, “Sculpt” and “Dance” tailored to the individual’s level of PF, thus creating an individualized multicomponent (balance, strength, agility, endurance) IVR exergame program. The exercise program was limited to 25-30 min per day and implemented under physiotherapist supervision in the clinic twice a week for 8 weeks. In the HBEP, a personalized multicomponent exercises were implemented based on the participants’ level of PF. The participants’ PF was assessed using the FitnessGram test battery, which included the Curl-up Test, Push-up Test, Trunk Lift Test (TLT) and Back Saver Sit and Reach Test (BSSRT). Functional capacity was evaluated through the 6-minute walk test (6MWT), the 10-step stair climbing (10-SSC) test and 1-minute sit-to-stand test (1-STS), along with isometric muscle strength of upper and lower extremities using a dynamometer. Additionally, in the JiaFit-XR group, participants’ scores after each game, as well as the estimated calorie expenditure (ECX) recorded and perceived rate of exertion (PRE) were assessed using the Borg Scale.

**Results:** Intragroup analysis showed significant improvements in the JiaFIT-XR group for the scores of the TLT, 6MWT, 10- SSC, and 1-STS, while the HBEP group demonstrated significant improvements in the scores of TLT, BSSRT, and 1-STS (p<0.05). In intergroup analysis, however, test results were similar in both groups (p>0.05).

Additionally, a significant change was observed in hamstring strength at 30 degrees of flexion and isometric muscle strength in the clubbing position of the upper extremity in JiaFIT-XR group. Furthermore, while there was a statistically significant increase in ECX for the JiaFIT-XR group over 16 sessions (p<0.05), no significant change was found in the PRE (p>0.05).

**Conclusion:** The preliminary results of this study indicate that the JiaFIT-XR program, applied for the first time for exercise purposes, is suitable for improving PF and functional capacity in JIA. It was found to be similarly effective as a HBEP in terms of trunk endurance and flexibility. Furthermore, as a significant outcome, while the ECX increased significantly as the difficulty level of the games increased, there was no significant change in the PRE. This suggests that despite the increasing challenge of the games and the consequent increase in physical activity, the perceived exertion level remaining unchanged indicates that children are benefiting optimally from the exercises, possibly due to the motivation and enjoyment provided by the games.

**Trial registration identifying number:** ClinicalTrials ID: NCT06176846

**Patient Consent**

Yes, I received consent

**Disclosure of Interest**

None Declared

**References**

1. Kuntze, G. et al. (2018). Exercise therapy in juvenile idiopathic arthritis: a systematic review and meta-analysis. *Arch Phys Med&Rehab* 99.1:178-193.
2. B., Jansen, B. et al. (2016). The use of commercial video games in rehabilitation: a systematic review. *Int J Rehab Res* 39(4),277-290.
3. Faric, et al. (2019). What players of virtual reality exercise games want: thematic analysis of web-based reviews. *J Med Internet Res*,21(9),e13833.

### e-health and digital health applications

#### PT81 Social media platforms as new classrooms for pediatric rheumatology: a large comparative study with real-data from over 14500 contents

##### Saverio La Bella^1,2^, Armando Di Ludovico^1^, Francesca Mainieri^3^, Federico Lauriola^1^, Luisa Silvestrini^3^, Francesca Ciarelli^3^, Jacopo Osmelli^3^, Virginia Girlando^1^, Marta Rinaldi^4^, Francesco Chiarelli^3^, Marco Gattorno^2^, Marina Attanasi^3^, Luciana Breda^1^

###### ^1^Department of Pediatrics - Pediatric Clinic and Rheumatology Unit, University “G. D’Annunzio” of Chieti, Chieti; ^2^Division of Reumatology and Autoinflammatory Diseases, IRCCS Istituto G. Gaslini, Genova; ^3^Department of Pediatrics - Pediatric Clinic, University “G. D’Annunzio” of Chieti, Chieti, Italy; ^4^Department of Pediatrics, Stoke Mandeville Hospital, Thames Valley Deanery, Oxford, United Kingdom

####### **Correspondence:** Saverio La Bella

*Pediatric Rheumatology 2024*, **22(2):**PReS24-ABS-1221

**Introduction:** Social media platforms are free and familiar tools used by patients, caregivers, influencers, and health professionals (HPs) for medical purposes, including Pediatric Rheumatology (1,2).

**Objectives:** The aim of this study was to investigate the characteristics, accuracy, and quality of social media content related to Pediatric Rheumatology.

**Methods:** The 150 most popular posts from 18 relevant hashtags/topics related to Pediatric Rheumatology were assessed on the social media platforms Facebook, Instagram, and TikTok.

Metrics, content creator types, sentiment, and misinformation were evaluated; for videos, the Journal of American Medical Association Benchmark Scale (JAMAS) was used for determining quality, while understandability and actionability were evaluated with the Patient Education Materials Assessment Tool (PEMAT).

**Results:** Of the 14551 posts evaluated, 6723 were included (pertinent content in English or Italian).

Videos obtained 520,8 million views and had a duration of more than 76,3 hours; videos and photos accounted for 34,6 million likes and 37,6 million interactions. 3165 posts regarded autoimmune diseases (47,1%), 1441 vasculitis (21,4%), 992 autoinflammatory diseases (14,8%), and 1125 other diseases/topics (16,7%).

Non-health professionals (NHPs) represented the majority of creators (5160, 76,8%), with 2700 patients (40,2%) and 1119 caregivers (17,8%). HPs were 1563 (23,2%), with 142 pediatric rheumatologists, 124 rheumatologists, and 68 pediatricians. Content was most often shared for reporting a patient or caregiver experience (3462, 51,5%), usually with neutral (1303, 34,8%) or positive sentiment (1296, 34,7%).

Educational content (2074/6723, 30,8%) consisted of 907/3593 videos and 1167/3130 photos. HPs provided longer (59 sec, IQR 85 sec vs 50 sec, IQR 77 sec; *p<0,001*) more understandable (PEMAT understandability 85,7, IQR 18,9 vs 75, IQR 25; *p<0,001*), more actionable (PEMAT actionability 66,7, IQR 33,3 vs 50, IQR 41,7; *p<0,001*), and higher-quality (JAMAS 3, IQR 0 vs 3, IQR 1; *p<0,001*) educational videos than NHPs.

NHPs shared educational photos (3, IQR 11 vs 1, IQR 8; *p<0,001*) and videos (8, IQR 50 vs 4, IQR 27; *p<0,001*) with more comments, videos with more views (6181, IQR 23417 vs 2967,5, IQR 20943; *p=0,034*), likes (116, IQR 691 vs 61, IQR 488; *p=0,014*), and interactions (172, IQR 904 vs 93,5, IQR 656; *p=0,011*) than HPs, probably due to the major personal involvement and interest of other users with similar health issues.

Educational videos without misinformation (827/907) were mostly provided by HPs (514 vs 313; *p<0,001*); in addition, quality was correlated with duration (rho 0,172, *p<0,*001), understandability (rho 0,150; *p<0,001*), actionability (rho 0,106; *p<0,001*), and being HPs (rho 0,206; *p<0,001*).

**Conclusion:** This is the largest study on Pediatric Rheumatology content on social media. While NHPs are actively participating in these discussions, there is a need for more high-quality, accurate information from HPs. The medical community should take a decisive step in this new field of education.

**Trial registration identifying number:** N/A

**Patient Consent**

Not applicable (there are no patient data)

**Disclosure of Interest**

None Declared

**References**

1. Nikiphorou et al, Ann Rheum Dis 2017; 2. La Bella et al, J Rheumatol 2024

### Pain, fatigue, disease experience and quality of life

#### PT82 Identifying the unmet needs of children, adolescents and families with Uveitis associated to Juvenile Idiopatic Arthritis (U-JIA): qualitative phase results of the work package 4 of pave (producing an arthritis value-framework with economic evidence)

##### Laura Martínez Mifsut^1^, Jordi Antón^2^, Joan Vinyets Rejon^1^, Elisabet Puiggrós Ruiz^1^, Joan Calzada Hernández^2^, Andrea Zacarias Crovato^2^, Sonia Carriqui Arenas^2^, Nolvia Castillo Giron^2^, Estibaliz Iglesias Jiménez^2^, Juan Manuel Mosquera Angarita^2^, Carolina Estepa Guillen^2^, Jesús Díaz Cascajosa^3^, Marta Morales Ballus^3^, Laura Sebastián Chapman^3^, Marta Llorens Capdevila ^4^, Marta González Martínez^5^, Maria Crespo Bosch^6^, Ariadna Riera Castelló^7^ and Group PAVE Sant Joan de Deu on behalf of the PAVE consortium

###### ^1^Quality and Patient Experience Department; ^2^Pediatric Rheumatology Department; ^3^Ophthalmology Department; ^4^Mental Health Area; ^5^Child Life Unit; ^6^Social Work Service; ^7^Rehabilitation and Physical Medicine Department, Sant Joan de Deu Barcelona Children’s Hospital, Barcelona, Spain

####### **Correspondence:** Jordi Antón

*Pediatric Rheumatology 2024*, **22(2):**PReS24-ABS-1071

**Introduction:** Beyond the clinical impact of JIA, this rheumatic disease generates a hidden socioeconomic burden that directly affects the quality of life (QoL) of patients and families. For this reason, a standardized approach to capture and disclose this burden is urgently needed. PAVE is a multinational study of the European Joint Program in Rare Diseases which main aim is to generate, synthesize and integrate evidence on the outcomes and socio-economic burden of the Juvenile Idiopathic Arthritis (JIA) to patients, families and society, through a collaborative partnership between six complementary working packages (WP).

**Objectives:** WP4 main goal is to identify unmet needs of children, adolescents and their families in U-JIA. The secondary goals are: 1:Identify the impact of U-JIA on their QoL and their activities of daily living and their emotional, social and economic well-being; 2: Define key strategies to solve their needs and; 3: Propose actions to improve their experience throughout the course of their disease (patients’ journey).

**Methods:** WP4 is developed in three distinct phases: 1: Qualitative research; 2: Quantitative research and; 3: Design and co-creation.

First phase used design thinking research techniques, considering two main parallel groups: U-JIA patients and JIA patients. A desk research was completed (107 articles reviewed) and different research techniques were applied: 15 non-participatory observations; 12 semi-structured in-depth interviews; 30 daily activities logbooks and; 4 focus groups. The last two research techniques were innovatively adapted to 4 subgroups (under 10; 10 to 13; 14 to 18 years old; and families). At the end of the first phase, a qualitative data analysis was done to identify the diversity of the different unmet needs and QoL of patients and families.

**Results:** A total of 15 professionals (pediatric rheumatologists, ophthalmologists, social workers, physiatrists and mental health), 2 patient associations and 62 families (45 patients, 67 parents) have participated on the first phase. Following the analysis of the research, the key insight and four opportunity areas (OA) have been identified to improve their experience and QoL:

Key insight: «The experience of patients and their families is determined by the uncertainty caused by the remission of flares of the pathologies (JIA and uveitis), both frequency and severity impact on their perception and experience».

Opportunity Areas:

- OA#1: Manage the remission of flares - to accompany patients and families in managing the constant change of the remission and flares.
- OA#2: Learn to live with uncertainty - to provide tools to learn how to manage the high level of uncertainty, offering as much calm as possible.
- OA#3: Resolve the misunderstanding - to raise awareness of the pathologies (healthcare system and society).
- OA#4: Reduce self-demand and guilt - to facilitate that patients and families feel understood so that their feelings of guilt disappear.

**Conclusion:** On this first qualitative phase, the key patients’ and families’ unmet needs have been identified. On the second phase - quantitative research, it will be possible to obtain the relevance of the needs among different profiles of patients and families. Through the definition of the patient journey and the identification of OA, on the last phase of the project it will be possible to define an action plan with innovative value-proposition initiatives to improve the QoL of JIA patients and families, with or without associated uveitis.

**Patient Consent**

Not applicable (there are no patient data)

**Disclosure of Interest**

None Declared

### Pain, fatigue, disease experience and quality of life

#### PT83 Exploring the hidden waters: evaluating lower urinary tract involvement in patients with juvenile idiopathic arthritis and familial mediterranean fever

##### Sedef Karamahmut^1^, Nilay Arman^2^, Asya Albayrak^3,4^, Asena Yekdaneh^3,5^, Selen D. Arik^6^, Nuray Aktay Ayaz^6^, İbrahim O. Ayaz^7^

###### ^1^Institute of Graduate Studies Physiotherapy and Rehabilitation Master of Science Program, Istanbul University-Cerrahpasa; ^2^Faculty of Health Sciences Department of Physiotherapy and Rehabilitation, Istanbul University-Cerrahpaşa; ^3^Institute of Graduate Studies Physiotherapy and Rehabilitation Doctorate Program, Istanbul University-Cerrahpasa; ^4^Faculty of Health Sciences, Department of Physiotherapy and Rehabilitation, Istanbul Kent University; ^5^Vocational School of Health Services Physiotherapy English Program, Fenerbahce University; ^6^Istanbul Faculty of Medicine, Department of Pediatric Rheumatology, Istanbul University, Istanbul, Türkiye; ^7^Urology Depertment, Başakşehir Çam and Sakura City Hospital, Istanbul, Türkiye

####### **Correspondence:** Sedef Karamahmut

*Pediatric Rheumatology 2024*, **22(2):**PReS24-ABS-1573

**Introduction:** Juvenile Idiopathic Arthritis (JIA) and Familial Mediterranean Fever (FMF) are the most common autoimmune and autoinflammatory rheumatic diseases in childhood (1,2). The effects of these diseases, especially on the lower urinary tract (LUT), have not been sufficiently addressed in the literature. Uroflowmetry is a simple and practical method and has been accepted as the first-line screening tool for evaluating LUT symptoms in children (3). However, there are limited studies evaluating uroflowmetry in JIA and FMF.

**Objectives:** The aim of this study was to investigate the urological involvement in patients with JIA and FMF.

**Methods:** Thirty-eight children and adolescents (15 JIA, 13 FMF, 10 healthy peers) aged 6-17 years, participated in the study. The Childhood Bladder and Bowel Dysfunction Questionnaire (CBBDQ) and the Dysfunction Voiding Symptom Score (DVSS) were used to assess the LUT symptoms of the participants. 15 patients with JIA and FMF with the highest scores were assessed with simple uroflowmetry. Uroflowmetry parameters included maximum flow rate (Qmax), voided volume and time, average flow rate, time to Qmax, hesitancy, and post-void residual (PVR) values.

**Results:** The mean ages of JIA, FMF and healthy controls were 13.06±3.12, 11.53±2.14, and 9.22±2.77 years, respectively. The scores of CBBDQ and DVSS were similar in JIA and FMF (p>0.05). However, statistically higher scores were found in CBBDQ and DVSS in JIA and FMF compared to the healthy controls (p<0.001). Of the JIA-FMF patients, 35.7% reported urinary incontinence, 57.7% reported postponement, 49.4% reported urgency. According to the uroflowmetry results, differences were found in 12 out of 15 children with JIA and FMF. In these 12 children voided volume, average flow rate, time to Qmax, hesitancy and post-void residual were 321.00±315.11 ml, 11.67±8.26 ml/s, 10.25±7.23 s, 13.00±7.60 s and 54.42±50.24 ml, respectively. Voided volume and the average flow rate were found to be lower than the determined normative values, whereas other values were found to be higher.

**Conclusion:** It is seen that the lower urinary system is negatively affected in patients with JIA and FMF compared the their healty peers. We consider that the multisystemic nature of the diseases, chronic inflammation, changes in the musculaskeletal system and medications can alter urine biochemistry and can lead to structural changes in the LUT in patients with JIA and FMF. This study emphasizes the importance of evaluating urinary system involvement in patients with JIA and FMF, and we believe that pelvic floor rehabilitation should be considered as a potential treatment method in managing lower urinary system dysfunctions in children with JIA and FMF.

**Date of birth::** 02.09.1998

**Patient Consent**

Yes, I received consent

**Disclosure of Interest**

None Declared

**References**

1. Ravelli, A.,&Martini, A. 2007.Juvenile idiopathic arthritis.The Lancet,369(9563), 767-778.
2. Ozdogan,H.,&Ugurlu,S.2019.Familial mediterranean fever.La Presse Médicale, 48(1),e61-e76.
3. Van de Beek C, Stoevelaar HJ, McDonnell J, Nijs HG, Casparie AF, Janknegt RA.Interpretation of uroflowmetry curves by urologists. J Urol. 1997;157(1):164‐168.

### Psycho-social aspects and rehabilitation

#### PT84 Food and nutrient intake in children with juvenile idiopathic arthritis: exploring nutritional risks of the specific carbohydrate diet

##### Naima Hagström^1^, Afsaneh Koochek^2^, Eva Warensjö Lemming^2,3^, Anders Öman^1^, Henrik Arnell^4,5^, Lillemor Berntson^1^

###### ^1^Women’s and Children’s Health; ^2^Food Studies, Nutrition and Dietetics; ^3^Medical Epidemiology, Department of Surgical Sciences, Uppsala University, Uppsala; ^4^Paediatric Gastroenterology, Hepatology and Nutrition, Karolinska University Hospital; ^5^Women’s and Children’s Health, Karolinska Institutet, Stockholm, Sweden

####### **Correspondence:** Naima Hagström

*Pediatric Rheumatology 2024*, **22(2):**PReS24-ABS-1121

**Introduction:** The James Lind Alliance has recognized diet as a key research priority for juvenile idiopathic arthritis (JIA)^(1)^. Despite this, and considerable interest from families affected by JIA, studies focusing on dietary interventions are scarce. The Specific Carbohydrate Diet (SCD), known for its potential in managing inflammatory bowel disease, has also shown promise for JIA^(2)^. Nonetheless, there is a lack of knowledge regarding its nutritional impact and associated risks.

**Objectives:** To comprehensively describe and evaluate food and nutrient intakes in children with JIA following the SCD and contextualize the results relative to general recommendations and intakes in the general population.

**Methods:** Using three-day dietary records at the end of a 4-week SCD intervention, food and nutrient intakes were evaluated in relation to the Nordic Nutrition Recommendations 2023 and data from a large Swedish dietary survey, Riksmaten Adolescents 2016-17 (RMA) (n=1282).

**Results:** Out of the 21 children who completed the one-month SCD intervention, ten submitted complete food records. All ten reported a high fruit and vegetable intake, meeting the recommended minimum intake of 500g/day, compared to only 6% in RMA. Median dietary fiber intake was 26g/d, (IQR 21-33) in SCD compared to 16g/d (IQR 12-22) in RMA. Both groups showed elevated saturated fat intake; however, the SCD group also had a high red meat intake compared to recommendations. While the SCD provided high intakes of most micronutrients, four out of ten children had a low Vitamin D intake. Calcium was the only nutrient for which a standard diet offered a higher intake than the SCD, with nine out of ten in the SCD group having inadequate intake.

**Conclusion:** The high consumption of fruits and vegetables likely contributed to a lower likelihood of nutrient inadequacy among children on the SCD compared to the general population. It also provided beneficial fiber and anti-inflammatory phytonutrients. However, inadequate calcium intake, and low vitamin D levels are concerns relevant for children with JIA who may be prone to low bone mineral density. For further dietary optimization, particular attention should be given to reducing saturated fat and red meat intake, considering the increased risk of cardiovascular disease that children with JIA may face in adulthood. These findings underline the necessity of tailored dietary guidance, specific to the disease, for optimal patient and parent support, regardless of adherence to a specialized diet. Further research is imperative to establish specific nutrient recommendations for children with chronic inflammatory conditions, paving the way for improved disease management.

**Patient Consent**

Yes, I received consent

**Disclosure of Interest**

None Declared

**References**

1. James Lind Alliance. Juvenile Idiopathic Arthritis (Netherlands) Top 10 [internet]. Southampton: National Institute for Health and Care Research; 2020 [2024 April 30]. Available from: https://www.jla.nihr.ac.uk/priority-setting-partnerships/juvenile-idiopathic-arthritis/top-10.htm.
2. Berntson L. A pilot study of possible anti-inflammatory effects of the specific carbohydrate diet in children with juvenile idiopathic arthritis. Pediatr Rheumatol Online J. 2021;19(1):88.

### Psycho-social aspects and rehabilitation

#### PT85 Significant levels of psychological stress, depression and anxiety symptoms in children with pediatric rheumatologic diseases

##### Erin M. Treemarcki^1^, Natalie Rosenwasser^2^, Tamar Rubinstein^3^, Natoshia Cunningham^4^, Aimee O. Hersh^1^, Vincent Del Gaizo^5^, Andrea Knight^6^ and CARRA Mental Health Workgroup and the CARRA Registry Investigators

###### ^1^Division of Pediatric Rheumatology, University of Utah, Salt Lake City; ^2^Seattle Children’s Hospital, Seattle; ^3^Children’s Hospital at Montefiore, Albert Einstein College of Medicine , Bronx; ^4^College of Human Medicine, Michigan State University, Grand Rapids; ^5^Childhood Arthritis and Rheumatology Research Alliance, Washington, United States; ^6^Hospital for Sick Children, University of Toronto, Toronto, Canada

####### **Correspondence:** Erin M. Treemarcki

*Pediatric Rheumatology 2024*, **22(2):**PReS24-ABS-1342

**Introduction:** Mental health problems are common in children with pediatric rheumatologic diseases (PRDs) and are associated with worsened quality of life and poorer disease-related outcomes. Psychological distress results from exposure to stress, may be exacerbated in response to traumatic events (e.g., COVID-19 pandemic), and can lead to significant mental health problems.

**Objectives:** We aimed to determine the burden of psychological distress in children with PRDs as defined by psychological stress experiences and investigate associations between physical stress, perceived psychological distress, anxiety, depressive symptoms, and COVID-related distress.

**Methods:** Patients at participating centers with a diagnosis of JIA (juvenile idiopathic arthritis), jSLE (juvenile systemic lupus erythematosus), or JDM (juvenile dermatomyositis) registered in The Childhood Arthritis and Research Alliance (CARRA) Registry were approached for enrollment. Consented participants completed a one-time survey during a scheduled rheumatology visit, including Patient-Reported Outcomes Measurement Information System® (PROMIS) measures for psychological stress experiences, physical stress, and depressive symptoms, in addition to the NIH-Toolbox Perceived Stress survey, clinically validated measures of depression (PROMIS, PHQ-9) and anxiety (SCARED), and a visual analog scale for COVID-related distress. Scores on the PROMIS and NIH-Toolbox measures of one standard deviation above the mean of the reference population (T-score 50) indicated high levels of that measure; for these measures, high levels signify a positive screen. Elevated scores on the PHQ-9 and SCARED were determined by clinical cutoffs (5 and 30 respectively). Descriptive statistics were used for patient characteristics and patient-reported outcomes. The relationship between psychological stress and other measures was determined by Pearson Correlation Coefficient.

**Results:** The 150 patients who completed the survey had a mean age of 13.5 years (SD=2.7) and a diagnosis of JIA in 136 (91%) (Table 1). Psychological stress experiences were elevated in 34% and physical stress experiences in 41% (Table 2). High levels of perceived stress were reported in 20% of patients aged 13-17 years and 16% of those aged 8-12 years. While increased depressive symptoms were seen in only 24% on the PROMIS measure, 51% of patients had a positive PHQ-9 depression screen; of those with a positive PHQ-9, only 6 (5%) had severe depression. Over a third of the cohort (38%) had SCARED scores concerning for anxiety disorder. Most patients endorsed mild distress from the COVID-19 pandemic (median 2, IQR 0,5); only 5 (3.5%) endorsed severe distress. Psychological stress was highly correlated with perceived stress, depressive symptoms (PROMIS and PHQ-9), and anxiety and COVID stress (Table 3); however, correlation with physical stress was not statistically significant.

**Conclusion:** Psychological distress is common in children with PRDs and associated with perceived stress, depressive symptoms, and anxiety, but not physical stress. Next steps include expanding this cohort to include a greater number of patients with JDM and jSLE, to ensure findings are representative of a broader general rheumatology clinical sample. Further study is needed to understand the relationship between psychological distress and disease factors, as well as the potential role of targeting psychological distress to mitigate risk of poor mental health and clinical outcomes.

**Patient Consent**

Not applicable (there are no patient data)

**Disclosure of Interest**

None Declared

### Psycho-social aspects and rehabilitation

#### PT86 Effects of neuromuscular excercises combined with exergaming program in a case of juvenile dermatomyositis

##### Asena Yekdaneh^1,2^, Nilay Arman^3^, Asya Albayrak^2^, Figen Çakmak^4^, Nuray Aktay Ayaz^5^

###### ^1^Vocational School of Health Services Physiotherapy English Program, Fenerbahce University; ^2^Institute of Graduate Studies Physiotherapy and Rehabilitation Doctorate Program; ^3^Faculty of Health Sciences Department of Physiotherapy and Rehabilitation, Istanbul University-Cerrahpaşa; ^4^Pediatric Rheumatology, Başakşehir Çam and Sakura City Hospital; ^5^Istanbul Faculty of Medicine, Department of Pediatric Rheumatology, Istanbul University, Istanbul, Türkiye

####### **Correspondence:** Asena Yekdaneh

*Pediatric Rheumatology 2024*, **22(2):**PReS24-ABS-1346

**Introduction:** Juvenile dermatomyositis (JDM) is the most common inflammatory myositis in children, characterized by chronic muscle inflammation, proximal muscle weakness and/or immobility (1). Neuromuscular control represents muscle strength-endurance, joint stability, proprioception and postural control (2). Meanwhile, exergaming, includes task-oriented exercise and enhances neuromuscular control in a virtual environment (3). Although there are studies highlights the importance of neuromuscular control and investigating the effects of exergaming in pediatric rheumatic diseases, no study has been found in JDM (3-4).

**Objectives:** The aim of the case study was to investigate the effects of a neuromuscular exercise and exergaming program in a patient with JDM.

**Methods:** A 16-year-old male patient had a right knee joint stiffness (secondary to trauma related inflammation in the Hamstrings (H) by MRI), pain, weakness in the legs, and walking difficulty, was included in a neuromuscular exercise and virtual reality (VR) based exergaming program twice a week, for 16 weeks, with a physiotherapist. Pre and post exercise program, muscle strength of the 30° knee flexion H and 60° knee extension Quadriceps (Q) and balance were evaluated using K-Push and K-Force Plates devices, respectively. The program formed 12 weeks of neuromuscular exercise and 4 weeks of VR based exergaming aimed at increasing muscle strength-endurance, balance, coordination, and speed.

**Results:** The pre-post peak, average strength (kg) and time to max (s) of the left H were 6.8-12.9, 5.6-10.5, 1.36-3.35, respectively. And the right H were 10.3-16.6, 7.9-12.9, and 2.52-4.09, respectively. For the left Q, the pre-post peak, average force (kg) and time to max (s) were 13.3-29.2, 10.7-24.4, 3.78-1.91, respectively. And the right Q were 7.3-28.9, 6.2-21.9 and 5.02-2.69, respectively. The pre-post asymmetry (%) values for the H and Q were 34.4-22.1 and 45.0-1.1, respectively. During the half squat, the pre-post surface area of the left lower extremity was 2587.6 mm^2^-1591.5 mm^2^, and the right lower extremity was 3595.3 mm^2^-2730.4 mm^2^.

**Conclusion:** The findings from this JDM case study demonstrate the initial efficacy of combining neuromuscular exercises with VR-based exergaming programs. Differences in H and Q parameters indicate improved muscle strength-endurance and symmetry. Reduced oscillations during squats, reflecting enhanced neuromuscular control, suggest restored proprioception, postural control, and stabilization. Therefore, for patients with JDM, a task-oriented multicomponent exercise program focusing on neuromuscular control may be effective, addressing complexities beyond muscle weakness.

**Date of birth::** 29.12.1994

**Patient Consent**

Yes, I received consent

**Disclosure of Interest**

None Declared

**References**

1. Robinson AB,Reed AM. Juvenile dermatomyositis.In:Kliegmann RM,Berhrman RE, Jenson HB,Stanton BF,editors. Nelson Textbook of Paediatrics. 20th ed. Philadelphia, USA:Saunders;2014.pp.1630–5.
2. Myer,G.D.,Brunner,H.I.,Melson,P.G.,Paterno,M.V.,Ford,K.R.,&Hewett,T.E.(2005).Specialized neuromuscular training to improve neuromuscular function and biomechanics in a patient with quiescent juvenile rheumatoid arthritis. *Physical therapy*, *85*(8),791–802.
3. Arman,N.,Tarakci,E.Tarakci,D.,&Kasapcopur,O.(2019).Effects of Video Games-Based Task-Oriented Activity Training (Xbox 360 Kinect) on Activity Performance and Participation in Patients With Juvenile Idiopathic Arthritis: A Randomized Clinical Trial. American journal of physical medicine & rehabilitation,98(3),174–181.
4. Klepper S.E.(2008).Exercise in pediatric rheumatic diseases. Current opinion in rheumatology,20(5),619–624.

### Services and pathways of care

#### PT87 Implementation and outcomes of a physiotherapy-led paediatric rheumatology triage service in the national centre for paediatric rheumatology, Ireland

##### Edel Carberry, Susan Ward, Derek Deeley, Karen Peate, Kathy Gallagher, Emma-Jane Mac Dermott, Orla Killeen

###### Rheumatology, National Centre of Paediatric Rheumatology, Childrens Health Ireland (CHI) at Crumlin, Dublin, Ireland

####### **Correspondence:** Susan Ward

*Pediatric Rheumatology 2024*, **22(2):**PReS24-ABS-1081

**Introduction:** A novel physiotherapist-led paediatric rheumatology triage service was established at the Irish National Centre for Paediatric Rheumatology (NCPR), in Children’s Health Ireland (CHI) at Crumlin in 2019. This was an initiative in response to waiting times which were in significant breach of access to care as per Standards of Care for children and young people with JIA (Davies et. al, 2010). This model of care has successfully and safely reduced waiting lists and provided intermediate care pathways for patients who do not necessarily require rheumatologist review in adult and paediatric services (Stanhope *et al.*, 2012).

**Objectives:**

- Establish how many referrals are suitable for a physiotherapy-led paediatric rheumatology triage service
- Outline how many referrals are managed independently
- Outline the number and type of outcomes following assessment
- Identify the frequency of imaging and other investigations as a result of this model of care

**Methods:** Referrals were actively triaged by a Consultant Paediatric Rheumatologist and deemed appropriate for physiotherapy-led triage service if: i) pattern of signs and symptoms appear to be non-inflammatory in nature, and ii) cases were not indicative of connective tissue disorder, specific rheumatologic disorder, nor an unexplained and/or significant co-morbid medical condition or complex neuro-disability history. Appropriate patients were then assessed by the Clinical Specialist Physiotherapist in the physiotherapy-led triage clinic.

**Results:** Between September 2019 and March 2024, n=434 new patients were managed in the physiotherapy-led triage clinic. 70% (n=303) were managed independently, of those 74% (n=250) were discharged following the first visit. 30% (n=131) of total patients seen required review with a Rheumatologist. Investigations were required to augment the clinical exam for 52% (n=224) of patients, including x-ray (38%, n=163), MRI (5%, n=20) and blood tests (41%, n=178)). Of the total patients 43% (n=185) were identified with non-inflammatory musculoskeletal pain, and 15% (n=69) were suspected to have JIA.

**Conclusion:** Physiotherapy-led triage clinics effectively manage paediatric rheumatology patients. Under the governance of Consultant Paediatric Rheumatologists, this service can independently manage patients who do not require Consultant Rheumatologist review and can appropriately identify those patients who do require further assessment with a Consultant Paediatric Rheumatologist. Almost 40% of patients who attended this service required imaging following clinical assessment.

**Patient Consent**

Not applicable (there are no patient data)

**Disclosure of Interest**

None Declared

**References**

1. Stanhope J, Beaton K, Grimmer-Somers K, Morris J. (2012) The role of extended scope physiotherapists in managing patients with inflammatory arthropathies: a systematic review. *Open Access Rheumatology: research and reviews.* May 30;4:49-55. eCollection 2012.
2. Davies K, Cleary G, Foster H, Hutchinson E, Baildam E; British Society of Paediatric and Adolescent Rheumatology. BSPAR Standards of Care for children and young people with juvenile idiopathic arthritis. Rheumatology (Oxford). 2010 Jul;49(7):1406-8. 10.1093/rheumatology/kep460. Epub 2010 Feb 19. PMID: 20173199.

### Services and pathways of care

#### PT88 Designing a care pathway for improving physical activity, exercise and sports in children and young people with juvenile idiopathic arthritis

##### Jeannette Cappon

###### Childrens Rehabillitation, Reade, Centre for Rehabillitation and Rheumatology, Amsterdam, Netherlands

*Pediatric Rheumatology 2024*, **22(2):**PReS24-ABS-1816

**Introduction:** Children and Young people (CYP) with JIA are at risk for developing low physical activity levels and decreased physical fitness compared to their peers with negative consequences on their general health and wellbeing and outcome of the disease.[1] Main barriers for optimal PA levels are symptoms of JIA, like pain and self imposed barriers. Facilitators are enjoyment and support from Health Professionals(HP) by providing information and encouraging children to stay active[2]. HP of the multidisciplinary CYP team of Reade wished to improve the access to the specialized HP team of Reade by designing a care path leading to address physical activity and sports as early as possible in the disease course in CYP with JIA.

**Objectives**

1. Improve access to the specialised HP team to address PA from an early stage of JIA.
2. To offer social support and care by HP with experience in paediatric rheumatology encouraging children to stay active.
3. To create a care path for a personalised plan for managing symptoms of JIA facilitating CYP to participate as much as possible in activities with peers and in sports.
4. Embedding the personalized plan of CYP in their daily life and close to their home, collaborating with community HP.

**Methods**

1. Meetings with paediatric rheumatologists and a specialized nurse were organised where the need for this care path was discussed.
2. Meetings with financial controller of Reade to confirm the boundaries of the pathway by a maximum number of consults.
3. Meetings with team members were organised to determine the content of the care pathway including measurement instruments
4. Develop flyers for patients on the pathway.
5. Inform the planning department how to schedule appointments
6. Inform the paediatric rheumatologists about the designed pathway
7. Supply the paediatric rheumatologists with the flyers to hand over to the patients

**Results:** A 4-week care pathway was designed and is opened in May 2024, during which a personal plan for physical activity, exercise and sports plan will be made CYP with JIA containing: 1 combination consult Rehabilitation doctor and Physiotherapist (60 min) followed by 3 weekly consults with the KFT (60 min). Components are education on JIA, focusing on possibilities and the needs of the individual child, collaborating with community PT and meaningful goal setting.

**Conclusion:** Thanks to this care pathway CYP with JIA and their parents have access to care addressing physical activity and sports as early as possible in the disease course.

**Patient Consent**

Not applicable (there are no patient data)

**Disclosure of Interest**

None Declared

**References**

1. ‘It might hurt, but you have to push through the pain’: Perspectives on physical activity from children with juvenile idiopathic arthritis and their parents Douglas L Race 2016
2. Physical activity as a promising alternative for young people with juvenile idiopathic arthritis: Towards an evidencebased prescription Rochette 2023

### e-health and digital health applications

#### PT89 The iPeer2Peer program for youth with juvenile idiopathic arthritis: a randomized controlled trial

##### Fareha Nishat^1^, Sara Ahola Kohut^1^, Paula Forgeron^2^, Adam Huber^3^, Lori Tucker^4^, Lynn Spiegel^1^, Ciaran Duffy^5^, Karen Watanabe Duffy^5^, Brian M. Feldman^1^, Sofia Olaizola^1^, Brenna McDermott^1^, Farideh Tavangar^6^, Jennifer N. Stinson^1,7^

###### ^1^Hospital for Sick Children, Toronto; ^2^University of Ottawa, Ottawa; ^3^Division of Pediatric Rheumatology, IWK Health Centre, Halifax; ^4^BC Children’s Hospital, Vancouver; ^5^Children’s Hospital of Eastern Ontario, Ottawa; ^6^University of Toronto , Toronto, Canada; ^7^Lawrence S. Bloomberg, Faculty of Nursing, University of Toronto , Toronto, Canada

####### **Correspondence:** Jennifer N. Stinson

*Pediatric Rheumatology 2024*, **22(2):**PReS24-ABS-1337

**Introduction:** Juvenile Idiopathic Arthritis (JIA) is chronic pediatric illness, whereby youth experience physical, and psychosocial challenges that result in reduced health related quality of life. Peer mentoring has been shown to improve disease self-management, promote positive health behaviours and provide a platform for social support in youth with chronic disease.

**Objectives:** To determine the effectiveness of the iPeer2Peer program on disease self-management (primary) and other clinical (secondary) outcomes in youth with JIA. Acceptability and satisfaction with the program were also assessed.

**Methods:** A waitlist randomized controlled trial was conducted to examine the iPeer2Peer program. English speaking youth aged 12-18 years with JIA were recruited from six tertiary care pediatric rheumatology centers in Canada. Youth randomized to the intervention group were matched with trained peer mentors (18-25 years; successfully managing their JIA), who provided peer support and JIA education. Mentoring sessions consisted of up to10 sessions of approximately 30-minute video calls conducted over a 15-week period. Those randomized to the control group received standard care for 15-weeks, upon completion of all endpoint measures, they were offered access to the iPeer2Peer program. Outcome assessments occurred at enrollment, 15 weeks post-intervention and 6-months post randomization. Self-management was measured using the TRANSITION-Q, higher scores suggesting better self-management. Effectiveness of the iPeer2Peer program was assessed using linear mixed effects models, with time, group and an interaction by time and group as predictors. Upon study completion, satisfaction with the program was assessed using content analysis of semi-structured interviews and focus groups with mentors and mentees randomized to receive the iPeer2Peer program.

**Results:** In total, 164 youth (mean age 14.4 ± 1.9 years, 78% female) were randomized to the study (iP2P Program n = 81, Control n = 83). The proposed sample size of 262 youth (131 per group) was not reached due to challenges in recruitment, likely impacted by the COVID-19 pandemic. The iPeer2Peer program did not show significant improvement in self-management (Baseline: iP2P Program: 58.8, Control = 58.1; 15 weeks scores: iP2P Program = 55.7, Control = 54.3; 6 monghts scores: iP2P Program = 63.1, Control = 60.2; p=0.71) or in any of the secondary clinical outcomes. However, youth in the intervention group were engaged, 80% (65/81) completed at least one call, the average number calls completed was 6. Both mentors and mentees were satisfied with the program. Mentees valued the ability to converse with mentors who empathized and related to their disease experience. Mentors found it fulfilling to be a resource to mentees, and noted that they themselves might have benefited from this type of support.

**Conclusion:** The iPeer2Peer did not produce a significant improvement in self management, or other secondary clinical outcomes. However, mentors and mentees were satisfied with the program and felt that mentorship provided real-world benefits for disease management and overall wellbeing.

**Trial registration identifying number:** ClinicalTrials.gov: NCT03116763

**Patient Consent**

Not applicable (there are no patient data)

**Disclosure of Interest**

None Declared

### JIA (oligo, poly, psoriatic)

#### PT90 Exploring occupational participation in children with juvenile idiopathic arthritis: a qualitative study on parents & carers perspectives

##### Amy Wescott

###### School of Allied Health and CommunityC, University of Worcester, Worcester, United Kingdom

*Pediatric Rheumatology 2024*, **22(2):**PReS24-ABS-1022

**Introduction:** Juvenile Idiopathic Arthritis (JIA) is a condition diagnosed before 16 years of age & is the most common rheumatic condition in children. It is reported children with JIA have reduced health related quality of life and participate less in physical & social activities. Participation within leisure activities is critical to childhood development (Bult et al. 2011). Occupational participation is engaging in work, play & activities of daily living which are desired and/or necessary to well-being (Forsyth, 2021). Participation is complex but vital for development & families with a child with a disability experience changes in their occupational routines and family participation (Law, 2002). Little research has been completed into how JIA impacts the child and the family.

**Objectives:** To explore parents/caregiver’s perspectives of their child & family’s occupational participation and how JIA has an impact on this. Barriers & facilitators to participation were also investigated.

**Methods:** The project was approved by the University of Worcester’s ethics committee following submission of relevant documents. A phenomenological approach was chosen as this encompasses an interpretive constructivist paradigm. The study aims to understand the lived experiences of parents/caregivers of a child with JIA & phenomenology recognises the depth of information that a person’s lived experience can offer to research. Using a qualitative methodology, eight semi-structured interviews were conducted to gain the perspectives of parents/caregivers. Thematic Analysis was used to identify themes.

**Results:** Three themes were found with six subthemes. As occupational therapy aims to encompass holistic values (RCOT, 2021), it is important to acknowledge the relationship the themes have with each other & how families manage this when experiencing occupational participation. The themes found were “Just getting on with it” including adapting. Outside support including relationships and support groups. Finally, the impact on mental health including the child’s mental health & parental stress. JIA impacts the whole family which influences occupational participation. The mental health aspect was significant when exploring the family’s occupational participation.

**Conclusion:** Occupational therapists & allied health professionals can use knowledge from the study to ensure collaborated approaches with families are taken to address family occupational participation. Holistic approaches are necessary to understand the complexities of participation and the barriers and facilitators experienced by families. The research has highlighted barriers and facilitators of family occupational participation. The themes of “Just getting on with it”, outside support and the impact on mental health were found to be barriers & facilitators to participation. With participation being an important area of childhood development, it is vital for participation to be addressed by health professionals to support the continuing care of children with JIA & their families.

**Patient Consent**

Not applicable (there are no patient data)

**Disclosure of Interest**

None Declared

**References**

1. Bult et al (2011) What influences participation in leisure activities of children & youth with physical disabilities? A systematic review *Research in Developmental Disabilities 32*(5) 1521–1529
2. Forsyth K (2021) “The Model of Human Occupation” in Duncan E. *Foundations for Practice in Occupational Therapy* 6^th^ ed London Elsevier 46-74
3. Law M (2002) Participation in the Occupations of Everyday Life *American Journal of Occupational Therapy 56*(6) 640–649

### JIA (oligo, poly, psoriatic)

#### PT91 Synovial tissue biopsies for the purpose of research in the UK tissue research in childhood arthritis consortium

##### Elizabeth Clay^1^, Christine Bolton^1,2,3,4^, Charlotte Smith^1^, Alyssia McNeece^3^, Sugrah Sultan^5^, James Kelly^6^, Neelam Khan^5^, Vicky Alexiou^3,4^, Klaudia Kupiec^7^, Inga Turtsevich^7^, Charlene Foley^7^, Chadwick Pils^3,4^, Christopher Mahony^1^, Annie Hackland^1^, Holly Adams^1^, Emily Powell^1^, Huong Nguyen^3,4^, Manigandan Thyagarajan^5^, Zishan Sheikh^5^, Catherine Cotter^5^, Patricia Nisa^1^, Samantha Chippington^7^, Sandrine Compeyrot-Lacassagne^7^, Elizabeth C. Rosser^8,9^, Andrew Filer^1,10^, Eslam Al-Abadi^5^, Lucy Wedderburn^3,4,7,8^, Adam Croft^1,10^

###### ^1^Rheumatology Research Group, Institute of Inflammation and Ageing, University of Birmingham, Birmingham; ^2^Kennedy Institute, University of Oxford, Oxford; ^3^Infection, Immunity and Inflammation Research and Teaching Department, University College London; ^4^NIHR Biomedical Research Centre at Great Ormond Street Hospital, University College London/ GOSH, London; ^5^Birmingham Women’s and Children’s NHS Foundation Trust; ^6^University of Birmingham, Birmingham; ^7^Great Ormond Street Hospital NHS Foundation Trust; ^8^Centre for Adolescent Rheumatology Versus Arthritis at UCL UCLH and GOSH, University College London/ GOSH; ^9^Centre for Rheumatology, University College London, London; ^10^NIHR Birmingham Biomedical Research Centre, Birmingham, United Kingdom

####### **Correspondence:** Elizabeth Clay

*Pediatric Rheumatology 2024*, **22(2):**PReS24-ABS-1678

**Introduction:** Analysis of synovial tissue in adult-onset inflammatory arthritis has resulted in a shift-change in our understanding of the disease pathophysiology; biopsy-driven, pathology-led treatment stratification trials are ongoing. Despite this success in adults, progress of tissue research in children has been hindered by lack of access to tissue.

**Objectives:** To develop a national, multi-centre synovial tissue biopsy research program to build capacity to perform tissue-based research studies in children and young people with Juvenile Idiopathic Arthritis (JIA).

**Methods:** Minimally invasive ultrasound-guided synovial tissue biopsies were obtained from children during joint injection procedures. Records of adverse events were made along with patient demographic and disease-related data. Pre and post-procedure questionnaires were performed to explore tolerability of the procedure. Joint tissue was fixed and paraffin embedded for histological analysis.

**Results:** We have established a UK wide observational cohort study (MAP-JAG) which has recruited seventy-six children diagnosed with JIA. In this tolerability analysis, data from fifty-nine participants were analysed. The demographics of recruited patients aligned with previous observations of JIA, with a female preponderance (67.1%) and a majority white ethnic background (59.2%). Within those that underwent biopsy procedures, biopsies were well tolerated with no serious adverse events, and no additional hospital stays or re-presentations. Eight children went on to have repeat biopsy procedures later in their disease course. Seventy-one percent of families who completed post-procedure quesionnaires within thirty days stated that they would be “very likely” to allow their children to have a repeat procedure, whereas only three percent stated they would be “very unlikely” to do so. Histology showed synovial lining layer confirming the presence of synovium and analysis revealved key inflammatory features of the sub-lining synovium.

**Conclusion:** Synovial tissue sampling can be integrated into routine care during joint injection procedures. Minimally invasive, ultrasound-guided synovial biopsies in children are safe, well tolerated and acceptable to families.

**Patient Consent**

Yes, I received consent

**Disclosure of Interest**

None Declared

### JIA (oligo, poly, psoriatic)

#### PT92 Feasibility and validity of a decision support tool for withdrawal of biologic therapy in non-systemic juvenile idiopathic arthritis

##### Janine V. Til^1^, Michelle Kip^1,2^, Robert Marinescu-Muster^1^, Karin Groothuis-Oudshoorn^1^, Gillian Currie^3^, Susa Benseler^3^, Joost Swart^2,4,5^, Bas Vastert^2,4,5^, Nico Wulffraat^2,4,5^, Rae Yeung^6^, Deborah Marshall^7^, Maarten IJzerman^8^ and UCAN CAN-DU and UCAN CURE consortia

###### ^1^University of Twente, Enschede; ^2^University Medical Center Utrecht, Wilhelmina Children’s Hospital, Utrecht, Netherlands; ^3^Alberta Children’s Hospital Research Institute, University of Calgary, Calgary, Alberta, Canada; ^4^Faculty of Medicine, Utrecht University; ^5^RITA, ERN, Utrecht, Netherlands; ^6^Toronto, University of Toronto, Ontario; ^7^Calgary, Alberta Children’s Hospital Research Institute, University of Calgary, Alberta, Canada; ^8^Erasmus School of Health Policy & Management, Rotterdam, Netherlands

####### **Correspondence:** Michelle Kip

*Pediatric Rheumatology 2024*, **22(2):**PReS24-ABS-1104

**Introduction:** Biologic disease-modifying anti-rheumatic drugs are highly effective in controlling disease activity in children with juvenile idiopathic arthritis (JIA). However, evidence on if and when to withdraw biologic therapy after a child has reached clinically inactive disease (CID) is urgently needed.

**Objectives:** To evaluate a judgement-based Decision Support Tool (DST) to support pediatric rheumatologists in making biologic therapy withdrawal decisions in children with non-systemic JIA.

**Methods:** The web-based DST model was based on multi-criteria decision analysis (MCDA). The DST content, including criteria and relative weightings, were based on interviews, focus groups and a clinical vignette study that were published previously (1-3). A combination of focus groups and surveys were used to elicit feedback about the design, face and content validity and feasibility in clinical practice of the prototype version of the DST among a sample of potential users from the Dutch-Canadian UCAN consortium.

**Results:** Eleven pediatric rheumatologists were included; eight (73%) were female and five (45%) were from Canada. Key themes that arose during the focus groups were: 1) The need to precisely define terminology, such as “clinically inactive disease”, to ensure uniform interpretation among all users; 2) The need for concise instructions on how and when to adjust relative importance of criteria in the model; 3) The need to practice with the DST to increase trust in the DST; 4) Suggestions about different purposes for use of the DST in clinical practice, such as its potential to explain a decision to patients or to involve patients in the decision-making process; 5) Future improvements of the DST, such as the inclusion of predictive clinical data regarding successful withdrawal decisions. The survey results showed that user willingness to use the DST in practice was high. Two respondents were willing to spend 1-2 minutes per patient consult on using the DST, 6 respondents 3-5 minutes, and 2 respondents were willing to spend 5-10 minutes.

**Conclusion:** Overall, the DST was well received in terms of its value in supporting pediatric rheumatologists in their decision process. Minor modifications were needed to improve the design and instructions in the DST. The next development step will be to add predictive clinical data regarding successful withdrawal decisions. The DST offers a structured approach to decision making regarding the withdrawal of biologic therapy, which could increase patient and parent involvement in decision making, and result in more consistent decision making among pediatric rheumatologists.

**Patient Consent**

Not applicable (there are no patient data)

**Disclosure of Interest**

J. Til: None Declared, M. Kip: None Declared, R. Marinescu-Muster: None Declared, K. Groothuis-Oudshoorn: None Declared, G. Currie: None Declared, S. Benseler: None Declared, J. Swart Consultant with: Consulting fee from Amgen, outside the submitted work, B. Vastert Grant / Research Support with: Grants and personal fees from SOBI and Novartis during the conduct of the study, N. Wulffraat: None Declared, R. Yeung Consultant with: Consulting fees from Novartis and Lily outside the submitted work, D. Marshall Grant / Research Support with: Non-financial support from ISPOR, and personal fees from Analytica, outside the submitted work, Consultant with: Non-financial support from consultancy (Illumina), outside the submitted work, M. IJzerman Grant / Research Support with: Institutional support from Illumina

**References**

1. Currie, G.R., Pham, T., Twilt, M. *et al.* Perspectives of Pediatric Rheumatologists on Initiating and Tapering Biologics in Patients with Juvenile Idiopathic Arthritis: A Formative Qualitative Study. *Patient* **15**, 599–609 (2022).
2. Currie GR, Groothuis-Oudshoorn CGM, Twilt M, Kip MMA, MJ IJ, Benseler SM, et al. What matters most to pediatric rheumatologists in deciding whether to discontinue biologics in a child with juvenile idiopathic arthritis? A best-worst scaling survey. Clin Rheumatol. 2023;42(8):2173-80.
3. van Til JA, Kip MMA, Schatorjé EJH, Currie G, Twilt M, Benseler SM, et al. Withdrawing biologics in non-systemic JIA: what matters to pediatric rheumatologists? Pediatr Rheumatol Online J. 2023;21(1):69.

### JIA (oligo, poly, psoriatic)

#### PT93 New early indicators of polyarticular course in new-onset oligoarthritis patients

##### Chiara Rossi^1^, Federica Raggi^1^, Simone Pelassa^1^, Francesca Antonini^2^, Chiara Artale^1^, Federica Briasco^1^, Martina Bartolucci^2^, Silvia Maria Orsi^1^, Marco Gattorno^1^, Angelo Ravelli^3^, Alessandro Consolaro^1^, Maria Carla Bosco^1^

###### ^1^Unit of Rheumatology and Autoinflammatory Diseases, Pediatric Science Department; ^2^Core Facilities, Clinical Proteomics and Metabolomics; ^3^Scientific Direction, IRCCS Istituto Giannina Gaslini, Genoa, Italy

####### **Correspondence:** Chiara Rossi

*Pediatric Rheumatology 2024*, **22(2):**PReS24-ABS-1524

**Introduction:** Oligoarthritis represents the most common category of Juvenile Idiopathic Arthritis in Western countries. Disease course and prognosis within the first two years after diagnosis exhibit considerable variability. The majority of patients shows an oligoarticular course, more benign and with a higher likelihood of achieving remission. However, a substantial proportion of patients (30-40%) develops a polyarticular course (pcJIA), more severe and requiring more aggressive therapeutic interventions. New biomarkers are demanded for early discrimination of patients at risk of developing pcJIA.

**Objectives:** The aim of this study was to identify new early biomarkers of pcJIA by characterizing inflammatory cells both in plasma and synovial fluid of patients with Oligoarthritis at disease onset combined with the analysis of extracellular vesicles (EVs) released by these cells.

**Methods:** We employed a strategy that combines classical methodologies for investigating inflammatory cells in liquid biopsies with system biology-driven omics techniques (miRNomics/proteomics) for analyzing extracellular vesicles (EVs) released by these cells. Ninety-seven treatment-naïve Oligoarthritis patients were recruited at disease onset and followed up for 24 months after diagnosis. Expression profiling of EV-miRNA (EV-miR) and EV-protein (EV-Prot) was carried out in plasma and synovial fluid samples collected at disease onset. Plasma samples from 25 age-matched healthy children were utilized as controls. Phenotypic characterization of monocytes/macrophages and T cell subsets was performed in peripheral blood samples from 26 patients and compared across different clinical courses.

**Results:** By omics approaches, we identified a signature of 7 EV-miRNAs and 112 proteins expressed both systemically and locally, able to discriminate new-onset patients from controls with a high potential diagnostic value. Employing supervised machine learning techniques and WGCNA analysis, we demonstrated the ability of EV-miR 29a, EV-miR 223, and 16 protein clusters in stratifying patients undergoing different disease course. Cytofluorimetric analysis revealed different proportions of activated CD4/CD8, effector memory CD8, T regulatory cells, and M1/M2 monocyte/macrophages expressing the inflammatory hypoxic receptor TREM1 between outcome groups both in synovial fluid and peripheral blood samples.

**Conclusion:** Our findings provide novel early potential indicators of pcJIA in new-onset Oligoarthritis patients, encompassing EV-miRNAs, EV-proteins, and distinct inflammatory cell subsets.

**Date of birth:** 3/26/1993 1

**Patient Consent**

Not applicable (there are no patient data)

**Disclosure of Interest**

None Declared

### JIA (oligo, poly, psoriatic)

#### PT94 Increased incidence of adverse events and events of special interest with step-up therapy in non-systemic JIA

##### Paivi Miettunen^1,2^, Ana I. Rebello-Gimenez^2^, Luca Carlini^2^, Violeta Panaviene^3^, Jordi Anton Lopez^4^, Sylvia Kamphuis^5^, Troels Troels Herlin6^6^, Pavla Dolezalova^7^, Marco Cattalini^8^, Helga Sanner^9^, Gordana Susic^10^, Maria C. Maggio^11^, Soad Hashad^12^, Reem Abdwani^13^, Donato Rigante^14^, Ana L. Rodriquez Lozano^15^, Chiara Pallotti^2^, Joost Swart^16^, Nicolino Ruperto^16^ on behalf of PRINTO and On behalf of the PRINTO investigators

###### ^1^Pediatrics, University of Calgary, Calgary, Canada; ^2^Pediatrics, Istituto Giannina Gaslini, Genoa, Italy; ^3^Children’s Hospital, Affiliate of Vilnius University Hospital Santaros Klinikos, Vilnius, Lithuania; ^4^Institut de Recerca Sant Joan de Déu - SJD Barcelona Children’s Hospital, Barcelona, Spain; ^5^Polikliniek Kinderimmunologie en -reumatologie, Sophia Kinderziekenhuis, Erasmus MC - Erasmus Medisch Centrum, Rotterdam, Netherlands; ^6^Aarhus University Hospital, Department of Paediatrics and Adolescent Medicine, Aarhus, Denmark; ^7^7 Charles University in Prague, Prague, Czech Republic; ^8^Università degli Studi di Brescia, Brescia (UNIBS), Italy; ^9^Rheumatology, Oslo University Hospital, Oslo, Norway; ^10^Institute of Rheumatology Belgrade, Belgrade, Serbia; ^11^University Department PROMISE “G. D’Alessandro, University of Palermo, Palermo, Italy; ^12^Tripoli Children’s Hospital, Tripoli, Libya; ^13^Sultan Qaboos University, Muscat, United Arab Emirates; ^14^Department of Life Sciences and Public Health, Fondazione Policlinico Universitario A. Gemelli IRCCS, Rome, Italy; ^15^Immunology, Instituto Nacional de Pediatría, Mexico City, Mexico; ^16^UOSID Centro Trial, PRINTO, IRCCS Istituto Giannina Gaslini, Genoa, Italy

####### **Correspondence:** Paivi Miettunen

*Pediatric Rheumatology 2024*, **22(2):**PReS24-ABS-1265

**Introduction:** It is not known if patients’ adverse event (AE) profile worsens, as their treatment is intensified.

**Objectives:** To report AEs of at least moderate intensity, serious AE and events of special interest (ESI) in non-systemic-JIA patients as they progressed from less intensive treatment with non-steroidal anti-inflammatory drugs (NSAIDs) to treatment with conventional synthetic and biologic DMARDs (csDMARDs/bDMARDs) with data from Pharmachild registry.

**Methods:** Inclusion criteria were children with non-systemic-JIA as per ILAR criteria with whole drug exposure from onset to last observation. Data was available from 1987 until December 2021. Non-systemic-JIA patients were classified according to their treatment (chosen by the treating physician) into either a “Control group” (CG) (NSAIDs +/- intra-articular (IA) glucocorticoids only) or a “STEP-up group” (starting with NSAIDS +/- IA glucocorticoids but subsequently requiring DMARDS as required for disease control): STEP-1 (NSAIDs +/- intra-articular glucocorticoids); STEP-2 (csDMARDs +/- oral glucocorticoids), and STEP-3 (bDMARDs +/- other medications). AEs were classified by the latest version of MedDRA dictionary (Version 23.1). Statistical analysis included descriptive statistics and Cox multivariate regression model.

**Results:** A total of 8052 non-systemic JIA patients (69.9% female) were included: 719 (8.95%) in CG; 7333 (91.1%) in STEP-1; 6856 (85.1%) in STEP-2 and 5052 (62.7%) in STEP-3. The most frequent JIA category was oligoarthritis (76 % in CG versus 39.1% in STEP-1). The median (IQR) duration of each treatment period was 5.54 (2.88-9.18) years: CG 2.65 (1.04-6.38), STEP-1 0.62 (0.25-1.79), STEP-2 1.59 (0.57-3.85) and STEP-3 3.13 (1.48-5.58) years. Methotrexate (94%) and Etanercept (70%) were the most frequent conventional synthetic and biologic DMARDs, respectively. AEs were seen in all groups, least frequently in STEP-1 (1.8%) and most frequently in STEP-3 (24.4%). SAEs were most common in STEP-3 (492 (8%) patients), and rare in STEP-1 (62 (0.8%) patients) and CG (6 (0.8%) patients). Infections were the most frequent AEs in all groups (Incidence rates 0.33-4.14/100 patient years), with bDMARD group having the highest rate. Gastrointestinal disorders were most frequent in STEP-2 (N, IR, 95% CI: 330, 1.81 (1.62- 2.01)), all other AEs in STEP-3. Patients treated with bDMARDs had the highest risk for development of first episodes of AE, SAE, infection and serious infection, with highest hazard ratio for serious infection (HR, 95% CI) at 19.17 (9.74- 37.74).

**Conclusion:** This is the first attempt to present a dynamic AE profile in a very large sample of non-systemic-JIA patients from an international pharmacovigilance registry applying a novel method of using patients as their “own controls”. Risk of AEs increased, and AE severity worsened with treatment intensification, with bDMARDs associated with a higher prevalence of AE, SAE and ESI when compared to treatment with csDMARDS or NSAIDs. The most frequent ESIs were infections.

**Patient Consent**

Not applicable (there are no patient data)

**Disclosure of Interest**

P. Miettunen: None Declared, A. Rebello-Gimenez: None Declared, L. Carlini: None Declared, V. Panaviene: None Declared, J. Anton Lopez: None Declared, S. Kamphuis: None Declared, T. Troels Herlin6: None Declared, P. Dolezalova: None Declared, M. Cattalini: None Declared, H. Sanner: None Declared, G. Susic: None Declared, M. Maggio: None Declared, S. Hashad: None Declared, R. Abdwani: None Declared, D. Rigante: None Declared, A. Rodriquez Lozano: None Declared, C. Pallotti: None Declared, J. Swart: None Declared, N. Ruperto Consultant with: 0, Speaker Bureau with: 0

### JIA (oligo, poly, psoriatic)

#### PT95 Microrna profiling in the synovial fluid and plasma of monoarthritis-onset JIA patients may uncover novel biomarkers for early prediction of joint involvement progression

##### Francesca Ridella^1^, Simone Pelassa^2^, Federica Raggi^2^, Chiara Rossi^2^, Silvia M. Orsi^1^, Marco Burrone^1,3^, Marco Gattorno^3^, Angelo Ravelli^4^, Alessandro Consolaro^1,3^, Maria C. Bosco^2^

###### ^1^Dipartimento di Neuroscienze, Riabilitazione, Oftalmologia, Genetica e Scienze Materno Infantili (DINOGMI), University of Genova; ^2^UOC Rheumatology and Autoinflammatory Diseases, Department of Pediatric Sciences; ^3^UOC Rheumatology and Autoinflammatory diseases; ^4^Direzione Scientifica, IRCCS Giannina Gaslini Institute, Genova, Italy

####### **Correspondence:** Francesca Ridella

*Pediatric Rheumatology 2024*, **22(2):**PReS24-ABS-1752

**Introduction:** Juvenile Idiopathic Arthritis (JIA) is the most common chronic pediatric rheumatic disease globally, posing a significant burden of disability. Monoarthritis is a frequent manifestation at disease onset, especially in Western countries; some patients persistently experience flares in the same joint throughout their illness. Unraveling the molecular mechanisms underlying monoarthritis pathophysiology, holds promises for identifying novel biomarkers crucial for early diagnosis, patient stratification, and targeted therapeutic approaches. As inflammatory and tissue-destructive cells release extracellular vesicles (EVs) into both plasma (PL) and synovial fluid (SF), characterizing EVs content at disease onset could yield valuable predictive biomarkers.

**Objectives:** The objective of this study was to identify potential early biomarkers in patients with recurrent monoarthritis, differentiating them from those who will experience different disease courses.

**Methods:** Thirty treatment-naive patients with monoarticular knee arthritis at disease onset were enrolled and followed for at least 24 months. They were categorized into three subgroups based on disease course at follow-up: Group 1 experienced recurrent flares in the same joint, Group 2 never flared, and Group 3 had other joints affected during follow-up. EV miRNA (EV-miR) expression profiling was conducted on PL and SF samples using TaqMan Array RT-PCR and results were compared among patients experiencing different disease courses.

**Results:** Differential expression analysis revealed significant decreased expression of EV-miR-99b in SF of Group 1 patients compared to Group 2. Conversely, EV-miR-193b, 320, and 886-5p were overexpressed in SF of Group 3 patients compared to Group 2, while EV-miR-155 was upregulated in SF of Group 3 patients compared to Group 1. In PL samples from Group 1, EV-miR 150 and 100 were upregulated compared to Group 3, while several EV-miRs, including 363, 125b, 210, 100, and 10a were upregulated compared to Group 2. Group 2 plasma samples exhibited downregulation of EV-miR 210 and upregulation of EV-miR-204 compared to Group 3.

**Conclusion:** Understanding the mechanisms of disease flares in JIA patients with recurrent monoarthritis through the characterization of the PL and SF EV-miRNomere, presents a promising avenue to identify targetable mediators of disease chronicity in autoimmune arthritis.

**Patient Consent**

Yes, I received consent

**Disclosure of Interest**

None Declared

### JIA (oligo, poly, psoriatic)

#### PT96 Age at onset of diagnosis as a prognostic factor in juvenile idiopathic arthritis

##### Anna-Kaisa Tuomi^1^, Katariina Rebane^1^, Hannu Kautiainen^2^, Mia Glerup^3^, Kristiina Aalto^1^ and Nordic Study Group of Pediatric Rheumatology (NoSPeR)

###### ^1^New Children’s Hospital, University of Helsinki and Pediatric Research Center; ^2^Folkhälsan Research Centre, Helsinki, Finland; ^3^Aarhus University Hospital, Department of Pediatrics, Aarhus, Denmark

####### **Correspondence:** Anna-Kaisa Tuomi

*Pediatric Rheumatology 2024*, **22(2):**PReS24-ABS-1544

**Introduction:** Age at onset of a chronic disease may have a negative impact on wellbeing. There are only few studies concerning age at onset as a prognostic factor for juvenile idiopathic arthritis (JIA).

**Objectives:** The aim of this study was to clarify how age at onset of JIA affects the outcome of the disease. We focused on the primary outcome parameters such as remission and Health-Related Quality of Life (HRQoL). In addition, functional ability after 18 years of the diagnosis was evaluated.

**Methods:** This study is part of the population-based Nordic JIA cohort study (1). Newly diagnosed patients with JIA were recruited consecutively between 1997-2000 in specific regions in Finland, Sweden, Norway, and Denmark. Initially, 510 patients were recruited and 358 (70%) of them attended this study. Excluded patients had insufficient information at six months and 18-year follow-up visit. Patients attending the study were divided into three different age groups: <3 years, 3-5 years and ≥6 years. They were also categorized into three groups according to their International League of Associations for Rheumatology (ILAR) JIA subtypes (2) at six months after the onset of the disease: oligoarthritis, seronegative polyarthritis and others (enthesitis-related arthritis, psoriatic arthritis, and undifferentiated arthritis. Seropositive polyarthritis (n=6) and systemic onset (n=14) JIA were excluded due to limited number of patients. Clinical data were collected six months and 18 years after the disease onset.

**Results:** Mean age was 5.8 years (N=201, 72% female), 5.9 years (N=85, 74% female) and 8.7 years (N=72, 51% female) for oligoarthritis, seronegative polyarthritis and in the group of others, respectively. Remission rates off medication were 62% (95% CI: 54-68), 49% (95% CI: 38-60) and 51% (95% CI: 39-66), respectively, p=0.012.

Related factors predicting remission at the time point of 18 years in the oligoarthritis group included age onset, OR = 0.89 (95% confidence interval (CI) 0.82-0.97), male gender, OR (95% CI) = 2.32 (1.09-4.98), Juvenile Arthritis Disease Activity Score -71 (JADAS71), OR (95% CI) = 0.75 (0.56-1.00), and uveitis, OR (95% CI) = 0.29 (0.11-0.78). In the seronegative polyarthritis group, onset age OR (95% CI) = 1.15 (1.01-1.32) and JADAS71 score, OR (95% CI) = 0.82 (0.73 to 0.94) predicted remission.

By using logistic regression models, estimated probability of achieving remission 18 years after the diagnosis shows that both genders achieve remission earlier when diagnosed at an earlier age in the oligoarthritis group. In seronegative polyarthritis group, achieving remission is the opposite compared to oligoarthritis group in females. In the group of others, patients in remission were similar (U-shaped) in both genders. Onset age had no significant effect on HRQoL and functional ability. ROC-analyses show optimal different age cut-off points between JIA subgroups and gender.

**Conclusion:** The current study shows a relationship between continuous age at onset of oligoarthritis and seronegative polyarthritis and remission outcome.

**Patient Consent**

Yes, I received consent

**Disclosure of Interest**

None Declared

**References**

1. Glerup M, Rypdal V, Arnstad ED, Ekelund M, Peltoniemi S, Aalto K, Rygg M, Toftedal P, Nielsen S, Fasth A, Berntson L, Nordal E, Herlin T; Nordic Study Group of Pediatric Rheumatology. Long-Term Outcomes in Juvenile Idiopathic Arthritis: Eighteen Years of Follow-Up in the Population-Based Nordic Juvenile Idiopathic Arthritis Cohort. Arthritis Care Res (Hoboken). 2020;72(4)

### Disease outcome and transition

#### PT97 Socioeconomic cost and health-related quality of life in patients with juvenile idiopathic arthritis

##### Juliane Dirks^1,2^, Jens Klotsche^2^, Martina Niewerth^2^, Ina Liedmann^2^, Florian Milatz^2^, Brittany Gerber^3^, Gillian Currie^3^, Jordi Antón^4^, Lien de Somer^5^, Traudel Saurenmann^6^, Joost Swart^7^, Yosef Uziel^8^, Carine Wouters^5^, Deborah A. Marshall^3^, Kirsten Minden^1,2^ and on behalf of the PAVE consortium

###### ^1^Charité Universitätsmedizin Berlin; ^2^Deutsches Rheuma-Forschungszentrum , Berlin, Germany; ^3^University of Calgary, Calgary, Canada; ^4^Hospital Sant Joan de Déu, Esplugues de Llobregat, Spain; ^5^Pediatric Rheumatology Leuven University Hospital, Leuven, Belgium; ^6^Cantonal hospital Winterthur, Winterthur, Switzerland; ^7^University Medical Centre Utrecht, Utrecht, Netherlands; ^8^Tel Aviv school of medicine, Tel Aviv, Israel

####### **Correspondence:** Jens Klotsche

*Pediatric Rheumatology 2024*, **22(2):**PReS24-ABS-1546

**Introduction:** Juvenile idiopathic arthritis (JIA) not only has a negative impact on body structures and functions but can affect all aspects of the life of a child and his or her family. The European Joint Programme on Rare Diseases Producing an Arthritis Value-Framework with Economic Evidence: Paving the Way for Rare Childhood Diseases (PAVE) project aims at building a framework to capture the full impact of JIA. As part of PAVE, the socio-economic impact of JIA is to be evaluated, which requires an assessment of the financial costs and health-related quality of life (HRQoL) of patients.

**Objectives:** The aim of this study was to quantify the socioeconomic impact of (JIA) from a societal perspective.

**Methods:** Prospectively collected clinical and cost data of the German multicenter inception cohort study of newly diagnosed JIA patients (ICON) were used to assess the annual costs and the three-year outcome of JIA. A bottom-up, cross-sectional, cost-of-illness analysis was performed. Parents of affected children completed questionnaires on the utilization of health resources, loss of productivity, and the patients’ HRQoL by the Pediatric Quality of Life Inventory (PedsQL 4.0). Patients for whom complete data were available for the first three years of observation were considered for the assessment of HRQoL over time.

**Results:** A total of 779 patients (76% female) and their families were assessed after a mean disease duration of 34±18 months. More than one-third of patients (38%) had polyarthritis, including extended oligoarthritis, another third (37%) had oligoarthritis, and 11% had enthesitis-related arthritis. Sixty-eight percent of patients had ever received DMARDs up to the time of assessment, 22% biological DMARDs. The mean clinical JADAS-10 score at assessment was 3.9±4.9, corresponding to average minimally active disease, and the mean CHAQ score was 0.3±0.5.

The mean total costs of JIA per year amounted to 9,321 (median € 4,611) per patient. Medication costs, in particular biologic drug costs (38% of total costs), and inpatient costs (23%) accounted for the largest share of total costs. Productivity cost due to parents’ absence from work accounted for 14% of total costs. Out-of-pocket costs borne by families were relatively low, averaging € 491 per year. Patients with systemic JIA incurred the highest costs (mean € 30,025, median € 9,222), followed by those with rheumatoid-factor positive polyarthritis (€ 14,572, median € 10,061).

At three-year follow-up, the 400 patients with complete data for each of the three observation years had improved in their overall HRQoL compared to the start of observation (87.9±12.8 vs 72.7±17.9, p<0.001) and achieved a favorable HRQoL on average (PedsQL ≥79.3 according to [1]). Both the physical (88.9±14.3 vs. 67.2±24.7, p<0.001) and psychosocial health (83.2±19.3 vs. 75.9±16.6, p<0.001) of the patients had improved significantly.

**Conclusion:** JIA poses a significant cost burden on the society. However, the cost incurred result in a clinically relevant improvement in the quality of life of those affected.

This study as part of the PAVE consortium is funded by the Federal Ministry of Research and Education (01GM2108)

**Patient Consent**

Not applicable (there are no patient data)

**Disclosure of Interest**

None Declared

**Reference**

1. Listing M, Mönkemöller K, Liedmann I, et al. The majority of patients with newly diagnosed juvenile idiopathic arthritis achieve a health-related quality of life that is similar to that of healthy peers: results of the German multicenter inception cohort (ICON). Arthritis Res Ther. 2018;20:106.

### Imaging

#### PT98 Preliminary evaluation of the Musculoskeletal Ultrasound Sum Score (MUSS) in the pro-kind project

##### Faekah Gohar^1^, Daniel Windschall^1,2^

###### ^1^Pediatric Rheumatology, St.Josef-Stift Sendenhorst, Sendenhorst; ^2^Medical Faculty, University of Halle-Wittenberg, Halle (Saale), Germany

####### **Correspondence:** Daniel Windschall

*Pediatric Rheumatology 2024*, **22(2):**PReS24-ABS-1238

**Introduction:** Musculoskeletal ultrasound (MSUS) evaluation is increasingly used in the monitoring of disease activity (DA) in patients with juvenile idiopathic arthritis (JIA). However, whilst power doppler (PD) indicates intra-synovial blood flow and B-Mode (BM) the extent of effusion or synovitis, MSUS disease activity (SDA) remains an unvalidated marker for DA in JIA, being tested in few longitudinal studies.

**Objectives:** We analysed the longitudinal change in clinical DA (CDA, measured by JADAS-10) compared to SDA (measured by BM and PD) and the novel MUSS score (sum of the highest individual BM and PD values per patient) in children with newly diagnosed JIA.

**Methods:** Patients in our centre who participated in the PROKIND^1^ prospective treat-to-target longitudinal multi-center study recieved MSUS of standard (knee, ankle, midfoot (TN, CN)) and additional affected joints at each visit. MSUS BM and PD scores were collated for each individual scanned joint using sonography reports or retrospective image review. The semi-quantitative paediatric OMERACT score (0 to 3) was used for scoring, which depends on the extent of effusion and/or synovial hypertrophy (BM) or intrasynovial doppler signals (PD) with 0 representing a normal finding and 3 the highest level of pathology. Finally, the highest BM and PD score of any affected joint/patient was combined to form the MUSS score (maximal possible score 6).

**Results:** Patients with Oligoarthritis (OA), n=12, age (median, range) 4 (1-16) years, OA-Extended (OA-Ex)/RF- Polyarthritis, n=27, 6 (1-15) and RF+ Polyarthritis, n=4, 13 (9-16) at baseline (T0) were included. AT T0, 100% of patients with a polyarticular course had standard joint plus hip ultrasounds, and 81% of RF-/Oligo-Ex patients also had MSUS of both elbows and hands, in comparison to 100% of those with RF+ polyarthritis. At follow up the majority of patients received knee, ankle and midfoot scans as standard, and other joints were scanned according to clinical need. In each JIA-subgroup a significant reduction in JADAS-10 from T0 to T1 (3-4 mths) could be demonstrated, which correlated with >50% reduction in the MUSS. Mean JADAS-10 and MUSS at each follow-up were: OA: T0: 13, 4, T1: 3, 0, T2: 2, 0, T3: 1, 0, T4 (12-16mth): 2, 2. OA-Ex/RF-Poly: T0: 22, 4, T1: 8, 2, T2: 5, 1, T3: 2, 0, T4: 2, 1. RF+Poly: T0: 20, 5, T1: 7, 2, T2: 3, 0, T3: 2, 0, T4: 6, 1. In 19/27 patients with a seronegative polyarthritis, therapy change over time was also analysed. Treatment intensification occured in n=6/19, 2/16, 0/8 and 1/8 at T1, T2, T3 and T4 respectively. At T1, 4/6 (66%) patients with treatment intensification had SDA (MUSS>2) and CDA (JADAS-10>3.8), the remaining 2/6 (33%) had no SDA but CDA. No patients with SDA alone at T1 had treatment intensification.

**Conclusion:** The novel MUSS correlated well with CDA over time, with both MUSS and JADAS-10 improving after the initiation of therapy. Therapy escalation was most likely in patients with CDA and SDA at first follow-up after treatment initiation. However, the combined use of SDA and CDA for the prediction of step-up therapy need requires further investigation. MUSS as a proxy marker for SDA in the treat-to-target management of children with JIA should be tested and validated in further studies.

**Trial registration identifying number:** ProKind is funded by the Innovation Fund “Gemeinsamer Bundesausschuss”, FKZ: 01VSF18031

**Patient Consent**

Yes, I received consent

**Disclosure of Interest**

None Declared

**Reference**

1. Eulert S, Vollbach K, Tenbrock K*, et al.* Pos0171 A standardized assessment of treatment and outcome of newly diagnosed patients with JIA within the PROKIND project – pathways for polyarticular jia. *Annals of the Rheumatic Diseases* 2022;81:315.

### Immunoregulation and basic science

#### PT99 Regulatory t cell blood signatures can measure and predict disease activity in juvenile idiopathic arthritis

##### Meryl H. Attrill^1,2^, Diana Shinko^1^, Telma Martins Viveiros^1^, Martina Milighetti^1,3^, Nina M. de Gruijter^4,5^, Bethany Jebson^2,4^, Melissa Kartawinata^2,4^, Elizabeth C. Rosser^4,5^, Lucy R. Wedderburn^2,4,6^, CHARMS study^2,4,6^, JIAP study^2,4,6^, Anne M. Pesenacker^1^

###### ^1^Institute of Immunity and Transplantation, UCL; ^2^Infection, Immunity & Inflammation Research and Teaching Department, UCL Great Ormond Street Institute of Child Health; ^3^Cancer Institute; ^4^Centre for Adolescent Rheumatology Versus Arthritis at UCL UCLH and GOSH; ^5^Centre for Rheumatology Research, Division of Medicine, UCL; ^6^NIHR Biomedical Research Centre, GOSH, London, United Kingdom

####### **Correspondence:** Meryl H. Attrill

*Pediatric Rheumatology 2024*, **22(2):**PReS24-ABS-1282

**Introduction:** No reliable biomarker currently exists to predict the erratic disease course of Juvenile Idiopathic Arthritis (JIA), characterised by persistent inflammatory flares of the joint. Normally, regulatory T cells (Tregs) maintain immune tolerance, with altered Tregs associated with autoimmunity. Treg signatures have shown promise in monitoring other autoimmune conditions, therefore a Treg gene and/or protein signature could offer novel biomarker potential in JIA.

**Objectives:** Here, we aim to assess peripheral blood (PB) Treg mRNA signatures, and Treg subsets by protein expression, to distinguish active from inactive JIA. If PB Treg measurements correlate with disease activity and can predict the risk of imminent flares, a Treg-derived biomarker could inform clinical decisions and treatment outcomes.

**Methods:** A 48 gene nanoString Treg signature was analysed on sorted CD4+CD25highCD127low PB Tregs from healthy controls, and individuals with active (active joint count, AJC ≥1) and inactive (AJC=0) oligoarticular or polyarticular RF negative JIA. Additionally, JIA Tregs were assessed by spectral flow cytometry, with unbiased clustering on gated CD4+Foxp3+ cells across 20 markers for activation, proliferation, and co-receptor expression.

**Results:** Machine learning on our Treg gene signature on PB Tregs generated a model to distinguish active JIA Tregs from healthy controls (AUC=0.9875). Biomarker scores from this model successfully differentiated inactive from active JIA PB Tregs. Scores correlated with clinical disease activity (cJADAS), and identified subclinical disease (AJC=0, cJADAS≥0.5) from remission (AUC=0.8980, Sens=0.8571, Spec= 0.8571). Furthermore, spectral flow cytometry revealed three Treg clusters by protein expression increased in active JIA PB, while one Treg cluster predominated in inactive JIA PB (AJC=0). The ratio of these Treg clusters correlated to cJADAS, and higher ratios (>4.0) could predict inactive individuals that flared by 6-month follow-up. Methotrexate (MTX) had no effect on biomarker potential of these Treg measures, yet active individuals on MTX had lower Treg cluster ratios closer to those in remission, possibly suggesting Treg cluster ratios could be adapted as a treatment response biomarker to indicate when remission has been achieved.

**Conclusion:** We therefore demonstrate altered Treg signatures and subsets as an important factor, and useful biomarker, for maintained remission in JIA. Ultimately, PB Treg signatures could serve as routine biomarkers to guide disease and treatment management.

**Patient Consent**

Yes, I received consent

**Disclosure of Interest**

None Declared

### Scleroderma and related syndromes

#### P001 Juvenile Localised Scleroderma (jls) patients experience delay in diagnosis, disease damage and poor patient-related outcomes: initial data from an international, prospective cohort study

##### Hanna Lythgoe^1^, Kaveh Ardalan^2^, Ivan Foeldvari^3^, Ozgur Kasapcopur^4^, Valentina Leone^5^, Suzanne C. Li^6^, Georgia Martini^7^, Linda Schraven^8^, Kathryn S. Torok^9^, Christina K. Zigler^2^, Nicolino Ruperto^10^, Clare E. Pain^11^ and for the Paediatric Rheumatology European Society (PRES) scleroderma working party and the Paediatric Rheumatology International Trial Organization (PRINTO)

###### ^1^Royal Manchester Children’s Hospital, Manchester, United Kingdom; ^2^Duke University School of Medicine, Durham, United States; ^3^Hamburg Centre for Pediatric and Adolescence Rheumatology, Hamburg, Germany; ^4^Istanbul University-Cerrahpasa, Istanbul, Türkiye; ^5^Istituto Mediterraneo Per I Trapianti e Terapie Ad Alta Specializzazione ISMETT, Palermo, Italy; ^6^Joseph M Sanzari Children’s Hospital, Hackensack Meridian School of Medicine, Hackensack, United States; ^7^University of Udine, Udine, Italy; ^8^Federation of European Scleroderma Associations, Tournai, Belgium; ^9^Children’s Hospital of UPMC, Pittsburgh, United States; ^10^IRCCS Istituto Giannina Gaslini UOC Gaslini Trial Centre/Servizio di Sperimentazioni Cliniche Pediatriche, Genova, Italy; ^11^Alder Hey Children’s NHS Foundation Trust, Liverpool, United Kingdom

####### **Correspondence:** Hanna Lythgoe

*Pediatric Rheumatology 2024*, **22(2):**PReS24-ABS-1097

**Introduction:** JLS is characterised by chronic inflammation of the skin leading to fibrosis and may be associated with local complications and extra-cutaneous manifestations. Patient-related outcome measures are not well-established for JLS research or widely used in clinical settings. The Localised Scleroderma Quality of Life Index (LoSQI) was developed following work showing that the Children’s Dermatology Quality of Life Index (CDLQI) may not capture the full impact of JLS(1,2). The LoSQI study was developed to facilitate cross-cultural adaptation and validation of the LOSQI.

**Objectives:** To evaluate established outcome measures and delay in diagnosis in patients enrolled within the LoSQI study.

**Methods:** Patients aged 8-18 years with JLS were recruited via PRINTO centres participating in the LoSQI study. Demographic, disease data and clinician and patient-reported outcome measures were collected prospectively (where translation available) using the PRINTO web platform. Data from this interim analysis is described descriptively from patients with locked baseline data. Ethical approval was obtained in accordance with local/national requirements.

**Results:** To date 142 patients have been recruited from 25 sites in 17 countries. There were 83 patients with locked data at baseline visit included in this analysis. Patients were mostly Caucasian (64/83, 77%), female (59/83, 71%) with a median age at baseline visit of 9.5 years (interquartile range 7.4-11.8 years). The median age at diagnosis was 8.8 years (6.1-10.5) with a median delay in diagnosis of 1.0 years (0.4-2.0). Over a third (38%) of patients experienced a delay in diagnosis of >1 year. Most patients had inactive disease at baseline (mLoSSI 1 (0-5), physician activity VAS 0 (0-2.3)) but many patients had damage (LoSDI 6 (4-12.5), physician damage VAS 2 (1-4)). The median CHAQ score was 0 (0.0-0.1).

Patients and parents/carers completed VASs on a 0-10 scale and scored the impact of JLS on them/their child in the last month similarly with a median of 1 (IQR 0-3.5 and 0-3 respectively). Parents were also asked to score worry about long-term impact of JLS and worry about medications with median scores of 6.5 (3.5-8.0) and 5 (2.0-8.0) respectively.

Patient-Reported Outcomes Measurement Information System (PROMIS) outcomes were categorised as per Carle et al(3). Patients were categorised as moderate or severe for fatigue in 33%, pain in 31%, anxiety in 29%, and depression in 19%. Patients were categorised as fair or poor in mobility in 35%, global health in 23%, and upper extremity in 21%. Median CDLQI score was 2 (0-5) suggesting a small effect, but as above may not capture the full impact of JLS.

**Conclusion:** This large, international prospective study of patients with JLS demonstrates that patients experience significant delay in diagnosis and disease damage. Despite most patients achieving clinically inactive disease they continue to experience fatigue and anxiety with parents having high levels of worry regarding long-term outcomes and medication impact.

**Patient Consent**

Not applicable (there are no patient data)

**Disclosure of Interest**

None Declared

**References**

1. Lewis-Jones and Finlay. Br J Dermatol 1995; 132:942-9
2. Ardalan et al. J Scleroderma Relat Disord 2018;3(2),175-81
3. Carle et al. Qual Life Res 2021;30(4)997-1004

### Psycho-social aspects and rehabilitation

#### P002 Postural balance may be more influential than muscle strength in stair ascent-descent performance in patients with juvenile idiopathic arthritis

##### Irem Donmez^1^, Nilay Arman^2^, Asena Yekdaneh^3,4^, Asya Albayrak^3^, Yusuf Acıkgoz^1^, Kubra Ucar^2^, Ozlem Akgun^5^, Nuray Aktay Ayaz^5^

###### ^1^Institute of Graduate Studies, Physiotherapy and Rehabilitation Master of Science Program; ^2^Faculty of Health Sciences, Department of Physiotherapy and Rehabilitation; ^3^Institute of Graduate Studies, Physiotherapy and Rehabilitation Doctorate Program, Istanbul University-Cerrahpasa; ^4^Vocational School of Health Services, Physiotherapy English Program, Fenerbahçe University; ^5^Istanbul Faculty of Medicine, Department of Pediatrics, Department of Pediatric Rheumatology, Istanbul University, Istanbul, Türkiye

####### **Correspondence:** Irem Donmez

*Pediatric Rheumatology 2024*, **22(2):**PReS24-ABS-1458

**Introduction:** Juvenile idiopathic arthritis (JIA) stands as the most prevalent chronic, autoimmune, and inflammatory disease within the pediatric population. It’s characterized by symptoms such as muscle weakness, joint involvement, pain and fatigue(1). The accompanying symptoms of the disease often impact the functional abilities of children(2). Studies have shown that individuals with JIA have lower muscle strength and walking speed, poorer postural balance and slower stair descent-ascent skills compared to their healthy peers(3). In children with JIA exhibiting lower physical proficiency compared to their healthy peers, it remains uncertain which condition should be prioritized in rehabilitation.

**Objectives:** The primary aim of this study was to investigate the relationship between lower extremity endurance, muscle strength and postural balance in patients with JIA and to determine which parameter is prioritized for enhancing endurance in the rehabilitation program.

**Methods:** A total of 45 individuals (21 girls-24 boys) diagnosed with JIA were included in the study. A 10-step stair ascent-descent test (10SCT) was applied to evaluate the participants’ functional performance.Objective measurements were made using the newly developed K-Plates and K-Push (Kinvent Physio, France) devices for isometric muscle strength and postural balance assessment.For postural balance measurement, they were asked to stand on one leg for 15 seconds with their eyes closed and open, and the asymmetry between the two extremities was recorded using the application of the device.Isometric muscle strength was measured with the knee flexed at 60° for Quadriceps muscle and at 30° for Hamstring muscle. Participants were instructed to maintain isometric contraction for 5 seconds in each position with a 5-second rest interval between limb measurements.Extremity muscle strengths were recorded with the application of the device.SPSS Version 24.0 program was used for statistical analysis.

**Results:** The mean age, height and weight of children with JIA were 13.76±2.14 years, 158.38±11.79 cm and 52.73±16.19 kg respectively. The mean of the 10SCT performance was 8.31±2.618 seconds. A significant correlation was observed between the stair ascent-descent test and the isometric muscle strength results of the eyes-closed single-leg stance test, 60° quadriceps, and 30° hamstring muscles(p<0.005). However, no correlation was found with eyes-open balance(p>0.005). The regression analysis of the relevant parameters indicated that the asymmetry in the single-leg stance with eyes closed was the most influential factor on the 10SCT performance(p<0.001).

**Conclusion:** The findings of our study indicate that postural alignment takes precedence in the stair ascent-descent performance in JIA. While the test conducted with eyes open did not yield statistical significance, a significant difference was observed in the measurement with eyes closed. This difference underlines the importance of sensory input and proprioception in physical competence. Consequently, when planning exercise prescription for individuals with JIA, it’s imperative to not only address muscle strength but also emphasize exercises aimed at improving balance and proprioception and to provide appropriate posture instructions during exercise.

This study was supported within the scope of TUBITAK 1001-Scientific and Technological Research Projects Support Program 121E690.

**Date of birth:** novembre 0

**Patient Consent**

Yes, I received consent

**Disclosure of Interest**

None Declared

**References**

1. Thatayatikom A. Modica R. De Leucio A.(2023).Juvenile Idiopathic Arthritis. In StatPearls. StatPearls Publishing.
2. Kraft, M., Hansmann, S. Analysis of movement disorders in pediatric and adolescent rheumatology. Orthopedics 52,618–625 (2023).
3. Vincent, K., Sharififar, S., Abdelmalik, B., Lentini, L., Chen, C., Woolnough, L. U. (2022).Gait parameters, functional performance and physical activity in active and inactive Juvenile Idiopathic Arthritis. Gait & posture, 98.

### JIA (oligo, poly, psoriatic)

#### P003 Unmet needs of adolescents and young adults with juvenile idiopathic arthritis: a systematic review

##### Sophie Hecquet^1^, Maurine Jouret^2^, Marion Thomas^1^, Alice Combier^1^, Julien Wipff^1^, Gertrude Touanga^1^, Anne Lohse^3^, Alexandre Belot^2^, Jean-Paul Larbre^4^, Véronique Hentgen^5^, Michael Hofer^6^, Pierre Quartier^7^, Alexandra Loisel^8^, Hervé Lefevre^8^, Yannick Allanore^1^

###### ^1^Rheumatology, Cochin Hospital, Paris; ^2^Pediatric, CHU Lyon, Lyon, ^3^Rheumatology, HNFC, Trevenans; ^4^Rheumatology, CHU Lyon, Lyon; ^5^Pediatric, Centre Hospitalier de Versailles, Le Chesnay, France; ^6^Pediatric, CHUV, Lausanne, Switzerland, ^7^Pediatric, Necker Hospital; ^8^Maison des adolescents, Cochin Hospital, Paris, France

####### **Correspondence:** Sophie Hecquet

*Pediatric Rheumatology 2024*, **22(2):**PReS24-ABS-1796

**Introduction:** Adolescents and young adults (YA) with chronic diseases require special care to meet their developmental and disease-related needs. Juvenile idiopathic arthritis (JIA) affects adolescents and can persist into adulthood. Physicians require training in the challenges faced by these specific age groups to facilitate transition and enhance therapeutic alliance.

**Objectives:** To describe the unmet needs of adolescents and YA with JIA

**Methods:** A systematic review was conducted. Medline, Embase, and Cochrane Library databases were searched. Studies published in the last 40 years assessing the needs of adolescents and YA with JIA were selected. This systematic review was guided by a protocol developed using the Preferred Reporting Items for Systematic Reviews and Meta-Analysis (PRISMA) statement.

**Results:** A total of 63 articles that met the inclusion criteria were reviewed and analysed for content. Data from 29 studies of 1913 patients were analyzed. The age range of the patients was 11-30 years and there was a predominance of patients with FR- polyarthritis and oligoarthritis. Of these studies, nine studies assessed the general needs of YAs with a view to improving their quality of life. In these nine studies, the main general needs mentioned by adolescents and YAs were mobility, (11%), future (22%), education (22%), disease management (33%), treatment (44%), social life (including family and sexual life) (55%), daily life activities (66%), patient involvement in therapeutic decisions and mental health (66%). Six studies focused on mental health needs and compassed various issues, such as the need for independence, emotional management, comparison with peers, constraint of the disease, normalizing illness, and lack of mental support. Anxiety was significantly higher in patients with JIA than in healthy controls. Studies underscored the insufficient support available, notably the inadequate training of psychologists in JIA. In this review, 4 studies focused on the physician-patient relationship expected by the patients. Patients highlighted the importance of “personalized care", “a change of roles" and “partnership”. Patients’ reported needs did not vary according to JIA subtype.

**Conclusion:** This review of the literature has highlighted the different issues that need to be addressed in the follow-up of adolescents and young adults with JIA and is consistent with studies conducted in other chronic diseases. Socio-professional life and mental health are critical issues in this group of patients who are more prone to anxiety. The systematic use of questionnaires such as the HEEADSSS or the GOOD2GO could be a practical tool to address these issues in our patients. The originality of this study lies in the description of the physician-patient relationship that adolescents expect to improve adherence. A cohort dedicated to this specific population, established collaboratively by adult rheumatologists and pediatricians, would facilitate the accumulation of long-term data. This would enable a better understanding of the population’s needs, improve the physician-patient relationship and allow for tracking the evolution of clinical practice during transition.

**Patient Consent**

Not applicable (there are no patient data)

**Disclosure of Interest**

None Declared

### JIA (oligo, poly, psoriatic)

#### P004 Development and content validity of a physical performance test battery for children diagnosed with juvenile idiopathic arthritis

##### Su-ané Zwiegelaar^1,2^, Kasha Dickie^3^, Deepthi R. Abraham^4^, Karen Welman^1,2^

###### ^1^Division of Movement Science and Exercise Therapy, Department of Sport, Exercise and Lifestyle Medicine, Faculty of Medicine and Health Sciences, Stellenbosch University; ^2^Division of Movement Science and Exercise Therapy, Department of Sport, Exercise and Lifestyle Medicine, Faculty of Medicine and Health Sciences, Stellenbosch University, The Movement Labratory, Stellenbosch, South Africa; ^3^Institute of Applied Sciences, University of Chichester, Chichester, United Kingdom; ^4^Department of Pediatrics and Child Health, Faculty of Medicine and Health Sciences, Stellenbosch University, Stellenbosch, South Africa

####### **Correspondence:** Su-ané Zwiegelaar

*Pediatric Rheumatology 2024*, **22(2):**PReS24-ABS-1032

**Introduction:** Movement specialists have a vital role in the treatment of children diagnosed with juvenile idiopathic arthritis (JIA) due to the long- and short-term musculoskeletal disabilities resulting from the disease, pharmacological intervention, and a resulting deconditioning cycle. Consequently, physical health assessment is critical to ensure the appropriate exercise prescription. Yet, within JIA exercise intervention research a high volume of outcome measures are being used, making it challenging to select appropriate physical performance outcome measures (PPOMs) in clinical practice and compare different interventions.

**Objectives:** The current research aimed to describe the development of a physical performance test battery, the Juvenile Arthritis Kinetic Health Index (JAKHI), and determine its content validity through expert opinion based on the COnsensus-based Standards for the selection of health status Measurement INstruments (COSMIN) guidelines.

**Methods:** The JAKHI was developed by selecting an appropriate conceptual framework, completing a systematic review, interviewing children with JIA, and surveying clinicians’ opinions regarding PPOMs. Content validity was assessed by 15 experts, namely four pediatric rheumatologists and 11 pediatric movement specialists based on the COSMIN guidelines through REDCap® (Version 12.0.3, EDC Software by Vanderbilt University).

**Results:** The systematic review, semi-structured interviews, and clinician questionnaire support the six JAKHI domains’ assessments. The JAKHI achieved satisfactory validity based on an 85% agreement. However, some experts did raise concerns, such as the lack of upper extremity activity inclusion, which the principal investigator addressed.

**Conclusion:** The JAKHI has satisfactory content validity and seems applicable as a low-cost, easy-to-use, individualized, instructor-administered assessment of holistic physical health in children aged six to 16, diagnosed with JIA.

**Patient Consent**

Not applicable (there are no patient data)

**Disclosure of Interest**

None Declared

**References**

1. Al-Mayouf SM, Al Mutairi M, Bouayed K, et al. Epidemiology and demographics of juvenile idiopathic arthritis in Africa and Middle East. *Pediatr Rheumatol*. 2021;19(1):166. 10.1186/s12969-021-00650-x.
2. Rochette E, Saidi O, Merlin É, Duché P. Physical activity as a promising alternative for young people with juvenile idiopathic arthritis: Towards an evidence-based prescription. *Front Immunol*. 2023;14:1119930. 10.3389/fimmu.2023.1119930.
3. Al-Muqiren TN, Al-Eisa ES, Alghadir AH, Anwer S. Implementation and use of standardized outcome measures by physical therapists in Saudi Arabia: barriers, facilitators and perceptions. *BMC Health Serv Res*. 2017;17(1):748. 10.1186/s12913-017-2693-2.
4. Saladin L, Voight M. Introduction to the Movement System as the foundation for physical therapist practice education and research. *Intl J Sports Phys Ther*. 2017;12(6):858-861. 10.26603/ijspt20170858.
5. McClure P, Tevald M, Zarzycki R, et al. The 4-Element Movement System Model to guide physical therapist education, practice, and movement-related research. *Phys Ther*. 2021;101(3):1-10. 10.1093/ptj/pzab024.
6. Davis LL. Instrument review: Getting the most from a panel of experts. *Applied Nursing Research*. 1992;5(4):194-197. 10.1016/S0897-1897(05)80008-4.

### Psycho-social aspects and rehabilitation

#### P005 Development of points to consider for promoting physical activity, exercise and sports in children and young people with juvenile idiopathic arthritis by PReS health professionals community of practice and science

##### Jeannette Cappon^1^ on behalf of PReS-HP Community of Practice and Science , Nilay Arman ^2^, Deniz Bayraktar^3^, Kristine Risum^4^, Florian Milatz^5^ and PReS-HP Community of Practice and Science

###### ^1^Childrens Rehabillitation, Reade, Centre for Rehabilitation and Rheumatology, Amsterdam, Netherlands; ^2^Department of Physiotherapy and Rehabilitation, Istanbul University-Cerrahpasa, Faculty of Health Science, Istanbul; ^3^Department of Physiotherapy and Rehabilitation, Izmir Katip Celebi University, Izmir, Türkiye; ^4^Oslo University Hospital/ Oslo Metropolitan University, Oslo, Norway; ^5^Deutsches Rheuma-Forschungszentrum Berlin, Berlin, Germany

####### **Correspondence:** Jeannette Cappon

*Pediatric Rheumatology 2024*, **22(2):**PReS24-ABS-1585

**Introduction:** PReS Health Professionals Community of Practice and Science (HP ComPaS) aims to develop the best possible evidence-based methods for preventing and managing physical and psychosocial consequences of rheumatic diseases in children and young people (CYP). Lifestyle factors such as physical activity (PA) have been associated with better outcomes in juvenile idiopathic arthritis (JIA) [1]. CYP with JIA tend to have lower PA levels than their peers without JIA and tend not to reach the minimum level of PA recommended by the WHO, despite favourable disease control [2]*.* Clear guidelines for promoting PA in JIA disease management are lacking.

**Objectives:** To develop points to consider in promoting PA based on the expert opinions of PReS-HP ComPaS and to explore the feasibility of developing recommendations for promoting PA, exercise, and sports following standardized international guidelines.

**Methods:** As part of restructuring PReS HP in 2020, an interest group for PA and fitness was established during a virtual ComPaS meeting. PReS HP from various disciplines were invited to three virtual meetings to discuss their opinions on promoting PA in CYP with JIA. Participants completed a survey confirming their expertise over five years in paediatric rheumatology, mainly working with CYP with JIA. Recommendations for Promoting PA in inflammatory arthritis and osteoarthritis in adults [3] served as starting point for discussing and drafting overarching principles for CYP with JIA.

**Results:** 19 HP from 4 disciplines and 11 countries contributed in 3 virtual meetings to develop 6 overarching principles and 10 points to consider compassing: standard care integration, the role of healthcare providers, skilled delivery, PA evaluation, consideration of contraindications, personalized goals, barrier management, individual adaptations, behavioural techniques, and flexible delivery modes to promote PA for CYP with JIA. Participants concluded an additional systematic literature review is needed to include evidence from the literature and expert opinions in an official guideline, which accomplishment was not feasible during the timeframe of the meetings.

**Conclusion:** PReS-HP succeeded in developing expert opinion based points to consider for promoting PA, exercise and sports in CYP with JIA. These efforts may lay the foundation for the development of standardized international guidelines by PReS-HP in the future.

**Date of birth::** avril 09,

**Patient Consent**

Not applicable (there are no patient data)

**Disclosure of Interest**

None Declared

**References**

1. Gualano B, et al. Physical activity for paediatric rheumatic diseases: standing up against old paradigms. Nat Rev Rheumatol 2017;13(6):368-79.
2. Bourdier P, et al. Physical activity and sedentary levels in children with juvenile idiopathic arthritis and inflammatory bowel disease. A systematic review and meta-analysis. Pediatr Res 2019;86:149-156.
3. Rausch Osthoff AK, et al. 2018 EULAR recommendations for physical activity in people with inflammatory arthritis and osteoarthritis. Ann Rheum Dis 2018;77:1251-60.

### Treatment

#### P006 The long-term effects of a short-term specific carbohydrate diet intervention in children with juvenile idiopathic arthritis

##### Naima Hagström^1^, Afsaneh Koochek^2^, Anders Öman^1^, Henrik Arnell^3,4^, Lillemor Berntson^1^

###### ^1^Women’s and Children’s Health; ^2^Food Studies, Nutrition and Dietetics, Uppsala University, Uppsala; ^3^Paediatric Gastroenterology, Hepatology and Nutrition, Karolinska University Hospital; ^4^Women’s and Children’s Health, Karolinska Institutet, Stockholm, Sweden

####### **Correspondence:** Naima Hagström

*Pediatric Rheumatology 2024*, **22(2):**PReS24-ABS-1297

**Introduction:** Diet is discussed for its potential to serve as an adjunctive treatment option for juvenile idiopathic arthritis (JIA). To date, research has focused on the specific carbohydrate diet (SCD), showing promising results albeit in a limited patient cohort ^(1)^. However, little is known regarding long-term sustainability and efficacy over time, factors critical to validate its role as an adjunctive, therapeutic approach for JIA.

**Objectives:** To examine long-term effects of the SCD in children with JIA as well as the change in use of disease-modifying antirheumatic drugs (DMARDs) before and after the intervention.

**Methods:** Influence of the one-month diet intervention and results from follow-up assessments at 3, 6, and 12 months were included. Disease activity was assessed using Juvenile Arthritis Disease Activity Score 27 (JADAS27), other measurements included Pain Visual Analog Scale (VAS) (0-10 cm), global assessment (GlobAss) patient/parent VAS (0-10 cm), duration of morning stiffness in minutes and the frequency of DMARDs usage both one year before and one year after the intervention.

**Results:** A total of 28 children were enrolled, with 21 completing the one-month intervention; 15 were followed for one year. The majority of patients adhered strictly to the diet for one to two months after the initial one-month intervention and then gradually returned to a standard diet. The levels of JADAS27, pain VAS (0-10 cm), Glob Ass patient/parent VAS (0-10 cm) and minutes of morning stiffness decreased significantly after one month of dietary intervention. The improvement observed in JADAS27 scores was sustained for the 15 patients who completed the one-year follow up (p = 0.037, Kruskal Wallis). Additionally, there was a noticeable decrease in the use of DMARDs the year following the dietary intervention compared to the previous year.

**Conclusion:** The significant clinical impact observed following a short trial of the SCD, and sustained improvement in JADAS27 scores throughout the year, highlight the potential for long-term benefits. However, the high dropout rate and brief adherence to the diet suggest it may be challenging to maintain long-term. Although, preliminary findings also point to a potential decrease in medical burden, further verification is necessary.

**Trial registration identifying number:** https://register.clinicaltrials.gov, Clinical trials identifier: NCT04205500, 2019/12/17)

**Patient Consent**

Yes, I received consent

**Disclosure of Interest**

None Declared

**Reference**

1. Berntson L. A pilot study of possible anti-inflammatory effects of the specific carbohydrate diet in children with juvenile idiopathic arthritis. Pediatr Rheumatol Online J. 2021;19(1):88.

### Macrophage activation syndrome

#### P007 Serum levels of interleukin-18, CXCL9, and interferon gamma in still’s disease complicated by macrophage activation syndrome

##### Inga Z. Turtsevich^1,2^, Kimberly C. Gilmour^3^, Ying Hong^1^, Despina Eleftheriou^1,2^, Paul A. Brogan^1,2^

###### ^1^Infection, Immunity and Inflammation, University College London Great Ormond Street Institute of Child Health; ^2^Paediatric Rheumatology; ^3^Immunology, Great Ormond Street Hospital NHS Trust, London, United Kingdom

####### **Correspondence:** Inga Z. Turtsevich

*Pediatric Rheumatology 2024*, **22(2):**PReS24-ABS-1617

**Introduction: Macrophage activation syndrome (MAS)** is classified as a form of secondary haemophagocytic lymphohistiocytosis (HLH) is a life-threatening complication of various rheumatic diseases such as Still’s disease affecting up to 46% of all paediatric Still’s patients (1,2). The immunological mechanisms involve the self-perpetuating activation of T cells and macrophages with sustained production of key pro-inflammatory mediators including IL-18, Interferon-gamma (IFNg) and CXCL9, a marker of IFNg activity (2,3). Limited data exist on the serum cytokine levels potentially guiding the implementation of tailored therapeutic strategies for MAS.

**Objectives:** To document the serum levels of IL-18, CXCL9, and IFNg in patients with active Still’s-MAS; inactive Still’s-MAS, healthy controls, and disease controls (pHLH or other connective tissue diseases [CTDs] without MAS) and explore their diagnostic utility to differentiate Still’s-MAS from pHLH.

**Methods:** The analysis included 72 serum samples collected before the COVID pandemic and stored at the Immunology laboratory at Great Ormond Street Hospital. The samples included: 13 with active Still’s-MAS; 3 with inactive Still’s-MAS; 18 with pHLH; 15 with CTDs without MAS and 23 non-matched healthy controls. Serum samples were analysed using Multiplex immunoassay (Meso Scale Diagnostics, (MSD^â^) LLC, Rockville, Maryland, USA) for IFNg, CXCL9 and IL-18. Statistical analyses employed GraphPad-Prism10.

**Results:** IL-18 levels were highest in active Still’s-MAS, followed by pHLH and inactive Still’s-MAS patients (p<0.0001). Although not significant (p=0.06), IL-18 was higher in active Still’s-MAS compared to pHLH, while IL-18 was higher in active Still’s-MAS versus inactive Still’s-MAS group (p=0.014). CXCL9 level was overall higher in active and inactive Still’s-MAS or pHLH versus CTDs without MAS and healthy controls (p<0.0001). There was no statistical difference in CXCL9 between patients with MAS and pHLH (p=0.77). IFNg concentrations did not significantly differ among all groups (p=0.122). ROC-analyses were performed to explore the diagnostic utility of IL-18 to differentiate active Still’s-MAS from pHLH: at an optimal cut-off level of 30,764 pg/ml IL-18 demonstrated only modest diagnostic utility (sensitivity 0.615, specificity 0.778, AUC 0.705, J index 0.393, p=0.055).

**Conclusion:** IL-18 could emerge as a crucial therapeutic target for Still’s-MAS, warranting trials in children and adults. However, IL-18 levels may not reliably distinguish Still’s-MAS from pHLH.

**Patient Consent**

Not applicable (there are no patient data)

**Disclosure of Interest**

None Declared

**References**

1. Foley CM, McKenna D, Gallagher K, McLellan K, Alkhdher H, Lacassagne S, et al. Systemic juvenile idiopathic arthritis: The Great Ormond Street Hospital experience (2005-2021). Front Pediatr [Internet]. 2023 [cited 2024 Mar 5];11. Available from: https://pubmed.ncbi.nlm.nih.gov/37780048/.
2. Crayne C, Cron RQ. Pediatric macrophage activation syndrome, recognizing the tip of the Iceberg. Eur J Rheumatol [Internet]. 2020 Feb 3 [cited 2023 Nov 19];7(Suppl1):13–20. Available from: https://pubmed.ncbi.nlm.nih.gov/31804174/.
3. Schulert GS. The IL-18/IFNγaxis in systemic JIA and MAS - New answers, more questions. Rheumatology (United Kingdom). 2021 Jul 1;60(7):3045–7.

### JIA (oligo, poly, psoriatic)

#### P008 Identifying the mechanisms of monocyte activation by synovial fibroblasts in juvenile idiopathic arthritis

##### Tobias Schmidt^1,2,3^, Anki Mossberg^1,3^, Elisabet Berthold^2^, Petra Król^1,3^, Anders A. Bengtsson^2^, Fredrik Kahn^4^, Bengt Månsson^1^, Robin Kahn^1,3^

###### ^1^Divison of Pediatrics, Deparment of Clinical Sciences Lund; ^2^Division of Rheumatology, Department of Clinical Sciences Lund; ^3^Wallenberg Center for Molecular Medicine; ^4^Divison of Infection Medicine, Department of Clinical Sciences Lund, Lund University, Lund, Sweden

####### **Correspondence:** Tobias Schmidt

*Pediatric Rheumatology 2024*, **22(2):**PReS24-ABS-1169

**Introduction:** Synovial monocytes from individuals with oligoarticular Juvenile Idiopathic Arthritis (oJIA) display an activated phenotype with functional alterations. Synovial fibroblasts (S-Fib) are recognized as key drivers of inflammation in adult arthritis, but little is known of their role in driving activation of key immune cells, such as monocytes, in oJIA.

**Objectives:** To explore the mechanisms of how S-Fib from oJIA patients induce activation in monocytes.

**Methods:** S-Fib were isolated from the synovial fluid (SF) of patients with oJIA through passaging. Isolated S-Fib were subsequently primed or not with a pool of cell-free SF for 48hrs, to re-introduce them into the inflammatory environment. The interaction with monocytes was studied in co-cultures using monocytes isolated from healthy donors. The monocytes were analyzed following co-culture with S-Fib using various assays believed to reflect key functions, such as surface expression, cytokine production and T-cell activation. To identify the mechanisms of monocyte activation induced by S-Fib, co-cultures were also performed in 0.4μm transwells. Additionally, the surface proteome of S-Fib was characterized by surface biotinylation and subsequent liquid chromatography mass-spectrometry. Finally, potential candidates were blocked in co-cultures using neutralizing antibodies.

**Results:** S-Fib induced an inflammatory phenotype in healthy monocytes, evidenced by upregulation of surface markers such as CD86 (p<0.0175). Additionally, co-culture induced the production of inflammatory cytokines (p<0.0209) and an increased ability of the monocytes to induce T-cell proliferation (p=0.0351). Interestingly, these effects were further enhanced by priming of the S-Fib with inflammatory SF prior to co-culture. To investigate the mode of activation, co-cultures were performed in 0.4um transwells, where the monocytes did not acquire the phenotype observed in direct co-cultures, suggesting a role of cell-cell contact. Preliminary data suggests that 32 surface proteins are upregulated on S-Fib at least 7-fold following priming with SF. These include previously described surface proteins important in immune cell crosstalk by S-Fib, such as ICAM-1. Indeed, blocking experiments suggest that ICAM-1 is partly involved in monocyte activation, as evidenced by inhibition of CD86 upregulation (p=0.0620). Additional key proteins involved in monocyte activation are currently being explored.

**Conclusion:** Our data show a role for S-Fib in driving inflammation in oJIA by inducing inflammatory monocytes. This process is dependent on cell contact and ICAM-1 represents one promising candidate that needs further exploration. These data further support that targeting cell-cell interactions could be a viable option to explore for novel treatment strategies in arthritis.

**Patient Consent**

Not applicable (there are no patient data)

**Disclosure of Interest**

None Declared

### Autoinflammatory diseases

#### P009 Clinical phenotype and laboratory markers in patients affected by A20 Haploinsufficiency (HA20): a case series from 2 Italian centers

##### Laura De Nardi^1^, Silvia Federici^1^, Marco Francesco Natale^1^, Camilla Celani^1^, Valentina Matteo^1^, Ivan Caiello^1^, Giusi Prencipe^1^, Eleonora De Martino^2^, Alessia Pin^2^, Fabrizio De Benedetti^1^, Alberto Tommasini^2,3^, Antonella Insalaco^1^

###### ^1^IRCCS Ospedale Pediatrico “Bambino Gesù”, Roma; ^2^IRCCS Materno Infantile “Burlo Garofolo”; ^3^University of Trieste, Trieste, Italy

####### **Correspondence:** Laura De Nardi

*Pediatric Rheumatology 2024*, **22(2):**PReS24-ABS-1449

**Introduction:** HA20 is a monogenic disease caused by heterozygous mutations in *TNFAIP3* which encodes A20, a negative regulator of inflammation. A20 reduced expression is associated with a wide range of clinical phenotypes including autoinflammatory and autoimmune manifestations. Despite the increasing number of patients described, no clear genotype-phenotype correlation has been found thus far^1^.Central nervous system(CNS) involvement has been reported in murine models carrying *TNFAIP3* variants but its prevalence in vivo is unknown^2^. We previously described high circulating levels of IFNγ-inducible chemokines(CXCL9/10) in a family with HA20^3^.

**Objectives:** To describe a cohort of patients with HA20 from 2 centers evaluating the different clinical features and their variability with patients' age; to assess the prevalence of CNS manifestations; to examine possible associations between the clinical phenotype, ongoing therapy and the inflammatory profile including CXCL9/10 and/or Interferon Score(IS) values.

**Methods:** Clinical and laboratory data of 25 subjects from 11 families with *TNFAIP3* variants were collected. We measured the circulating levels of CXCL9/10 and/or IS. Categorical variables were expressed as medians and IQR, median ages of symptoms onset were compared through Kruskal-Wallis test and groups were compared using R function Wilcoxon test.

**Results:** 6 out of 11 families carry mutations resulting in stop codon(AGMC class4-5) while 5 had missense variants(AGMC class3).Patients' clinical features were as follows: 76% oral aphtosis; 60% gastrointestinal inflammation; 44% recurrent fever;44% autoimmunity (celiac disease or type 1 diabetes or thyroiditis); 44% CNS involvement; 40% genital ulcers; 24% skin inflammation; 24% arthritis/tenosynovitis.15 patients had clinical onset <6years and presented more frequently oral aphtosis(p=0.016) and gastrointestinal inflammation(p=0.015) during lifetime. The median ages of the single symptom onset were significantly different among groups (p<0.001) and were: oral aphtosis(5 yrs, 4.5-6),recurrent fever(6 yrs, 2.5-6), gastrointestinal disease(9yrs, 8.5-17.5), skin inflammation(11.5yrs, 7.8-13.8), arthritis/tenosynovitis(15.5yrs,10.5-23.5).11 patients presented neuropsychiatric symptoms:1 autoimmune encephalitis, 4 mood disorders, 4 anxiety, 3 fibromyalgia, 2 ADHD,1 pica. No correlations were found between CXCL9/10 values and the type of variant, the clinical phenotype, disease activity or the ongoing therapy. A mild correlation between IS(>2) and CNS symptoms was found (r=0.53).

**Conclusion:** A marked clinical heterogeneity was observed among patients, even within the same variants. Children with early onset(<6 yrs) presented more often oral aphtosis and gastrointestinal disease. Different symptoms seem to be more prevalent in specific age ranges. Neuropsychiatric involvement has been found in several patients and might further expand the clinical phenotype. No correlation between CXCL9/10 values and clinical features, disease activity or response to treatment have been found, suggesting they cannnot be used as reliable markers of disease activity or response to treatment.

**Patient Consent**

Yes, I received consent

**Disclosure of Interest**

None Declared

**References**

1. Front. Immunol.11:574992
2. Journal of Neuroinflammation 2014,11:122
3. Front Immunol 2022;13:804401.

### Autoinflammatory diseases

#### P010 Familial Mediterranean fever: without fever?

##### Hatice Dilara Karakas, Zeynep B. Ozcakar, Fatma Aydin

###### Pediatric Rheumatology, Ankara University, Ankara, Türkiye

####### **Correspondence:** Hatice Dilara Karakas

*Pediatric Rheumatology 2024*, **22(2):**PReS24-ABS-1528

**Introduction:** Familial Mediterranean fever (FMF), one of the most common periodic fever syndromes of childhood, is an autosomal recessive autoinflammatory disease characterized by manifestations of polyserositis, arthritis, or erysipelas-like erythema accompanying recurrent fever. Its pathophysiology is based on the uncontrolled release of proinflammatory and pyrogen cytokines, primarily IL-1β, by MEFV gene mutations encoding the pyrin protein.

**Objectives:** The aim of this study was to investigate the demographic, clinical, and laboratory findings of patients diagnosed with FMF without fever. In addition, their differences with other patients without fever in the study group were evaluated.

**Methods:** Patients who were followed up for at least 6 months with the diagnosis of FMF in the last 10 years (2011-2021) in the Department of Pediatric Rheumatology of Ankara University Faculty of Medicine were included in the study. The diagnosis of familial Mediterranean fever was made according to the “Ankara” pediatric diagnostic criteria. The data were taken from electronic patient files and analyzed retrospectively.

**Results:** In the last 10 years, 576 patients (52.3% female) were followed up with FMF in our clinic. In 7.1% (n=41) of these patients, fever was not a sign of FMF attacks. There was no difference in gender distribution between the groups with and without fever (p>0.05). Patients without fever were compared to patients with fever; it was found that the age of onset and diagnosis was statistically significantly later (p<0.001), and the delay time to diagnosis was shorter in those without fever (p=0.039). In these patients, abdominal pain was found to be less common (p<0.001), but recurrent acute arthritis was more common (p=0.020). FMF-related diseases were found to be more common in these patients (p<0.001), and FMF was less common in the family (p=0.040). It was determined that this group did not require any anti-IL-1 treatment.

**Conclusion:** Fever may not accompany attacks as a clinical complaint in a very small proportion of FMF patients. In patients with recurrent short-term abdominal pain, chest pain, inflammatory complaints such as arthritis, and family history, FMF should also be considered in the differential diagnosis in the absence of fever.

**Trial registration identifying number:** the ethics committee of Ankara University 2022/486

**Date of birth:** février 17

**Patient Consent**

Yes, I received consent

**Disclosure of Interest**

None Declared

**References**

1. Ben-Chetrit E, Levy M. Familial Mediterranean Fever. Lancet 1998; 351: 659-664.
2. Yalcinkaya F, Ozen S, Ozcakar ZB, et al. A new set of criteria for the diagnosis of familial Mediterranean fever in childhood. Rheumatology 2009; 48: 395-398.
3. Gezgin Yildirim D, Bakkaloglu SA, Buyan N. Protracted febrile myalgia as a challenging manifestation of familial Mediterranean fever: case-based review. Rheumatol Int 2019; 39: 147-152.
4. Aydin F, Ozcakar ZB, Yalcinkaya F. Diagnosis and Treatment of Familial Mediterranean Fever in Children. Turkiye Klinikleri Rheumatol-Special Topics. 2017; 10(1):46-54.

### Autoinflammatory diseases

#### P011 Interim analysis of canakinumab dose extension in children with colchicine resistant familial Mediterranean fever: PERA-RG experience

##### Gülşah Kavrul Kayaalp^1^, Şengül Çağlayan^2^, Kadir Ulu^2^, Fatma G. Demirkan^1^, Vafa Guliyeva^1^, Gülçin Otar Yener^3^, Kübra Öztürk^4^, Ferhat Demir^5^, Semanur Özdel^6^, Mustafa Çakan^7^, Hafize E. Sönmez^8^, Betül Sözeri^2^, Nuray Aktay Ayaz^1^ and PeRA Research Group

###### ^1^Department of Pediatric Rheumatology, Istanbul University, Istanbul Faculty of Medicine; ^2^Department of Pediatric Rheumatology, Ümraniye Research and Training Hospital, Istanbul; ^3^Department of Pediatric Rheumatology, Sanliurfa Research and Training Hospital, Sanliurfa; ^4^Department of Pediatric Rheumatology, Goztepe Prof. Dr. Süleyman Yalçın Research and Training Hospital, Istanbul Medeniyet University; ^5^Department of Pediatric Rheumatology, Acıbadem Health Groups Hospital, Istanbul; ^6^Department of Pediatric Rheumatology, Ankara Dr. Sami Ulus Research and Training Hospital, Ankara; ^7^Department of Pediatric Rheumatology, Zeynep Kamil Research and Training Hospital, Istanbul; ^8^Department of Pediatric Rheumatology, Kocaeli University Faculty of Medicine, Kocaeli, Türkiye

####### **Correspondence:** Gülşah Kavrul Kayaalp

*Pediatric Rheumatology 2024*, **22(2):**PReS24-ABS-1582

**Introduction:** In familial Mediterranean fever (FMF) patients unresponsive to colchicine, successful outcomes have been achieved with the use of anti-interleukin-1 therapies. Nevertheless, there is a lack of definitive data concerning the duration and dosage regimens of these treatments in children. Positive results have emerged from a multicenter study from Turkey regarding a standard dose extension protocol implemented in FMF patients treated with canakinumab; however, the follow-up periods of patients in this study were short and longer-term results should be evaluated.

**Objectives:** The aim of this study is to evaluate the long-term outcomes of the canakinumab dose extension protocol in patients with FMF who are resistant to colchicine, and to establish the feasibility of implementing the related protocol.

**Methods:** The dose extension protocol was based on a consensus protocol previously established in Turkey through a multicenter approach and the Delphi technique. According to this protocol, in patients who remained attack-free during the first 6 months after starting canakinumab treatment, the dose interval can be doubled. Furthermore, it is recommended that in patients who remain attack-free for one year after the dose interval has been doubled, the dose interval can be tripled. This study included the patients diagnosed and followed-up with FMF in 7 centers in Turkey, and initiated canakinumab treatment due to inadequate response to colchicine, and whose canakinumab treatment regimen was in accordance with this protocol. The data was obtained from medical charts retrospectively.

**Results:** The study included 45 patients who initially started on monthly canakinumab treatment, and after 6 months, the dosing interval was extended to every 2 months. Due to FMF attacks observed in 7 patients (15.56%) while receiving treatment every 2 months, the medication dosages were reverted to monthly, and the dosing interval was not further extended. Out of the remaining 38 patients (representing 84.44%), after one year of the initial dose extension, the dosing interval was extended further to every 3 months. In 9 of these patients, the dosing interval was reverted back to bi-monthly due to the occurrence of attacks. For the 7 patients who were receiving treatment every 3 months, canakinumab was discontinued due to long-term remission, with a median remission duration of 10 months (range 6-21 months). Among them, 3 patients continued to remain attack-free even after discontinuation, while 4 experienced attacks upon stopping the medication, necessitating the resumption of treatment. Currently, 22 patients (57.89%) are still receiving canakinumab every 3 months. The median follow-up duration for these patients is 19.5 (6-55) months. During this period, 7 patients experienced one attack while on the every 3-month regimen, yet they continue to receive treatment every 3 months, while no FMF attacks or subclinical inflammation were observed in the remaining 15 patients.

**Conclusion:** The protocol mentioned for the dose extension of canakinumab treatment appears promising. Further extensive studies with larger cohorts and prospective randomized controlled trials are needed to explore anti-interleukin-1 treatment dosage regimens more comprehensively.

**Date of birth:** octobre 27

**Patient Consent**

Not applicable (there are no patient data)

**Disclosure of Interest**

None Declared

### Autoinflammatory diseases

#### P012 The scope of anti cytokine treatment in familial Mediterranean fever; a translational study

##### Erdal Sag^1,2^, Nur K. Tasdemir^2^, Busra Aydin^2^, Ozge Basaran^1^, Veysel Cam^1^, Yelda Bilginer^1^, Seza Ozen^1,2^

###### ^1^Department of Pediatric Rheumatology; ^2^Translational Medicine Laboratories, Pediatric Rheumatology Unit, Hacettepe University, Ankara, Türkiye

####### **Correspondence:** Erdal Sag

*Pediatric Rheumatology 2024*, **22(2):**PReS24-ABS-1090

**Introduction:** Familial Mediterranean Fever (FMF) is the most common monogenic autoinflammatory disease, characterized by recurrent attacks of inflammation. About 2-5% of patients are resistant to colchicine and will require further therapy to control ongoing inflammation. Over the years it became evident that FMF patients and carriers could have some inflammatory comorbidities, such as spondyloarthropathies, affecting the course and management of the disease and requiring additional therapy to control this musculoskeletal morbidity.

**Objectives:** We investigated the efficacy of methotrexate and various biologic drugs in the cell cultures of Familial Mediterranean fever (FMF) patients, particularly those resistant to colchicine and those with SpA.

**Methods:** Peripheral blood mononuclear cells (PBMCs) from nine FMF patients meeting Eurofever criteria and undergoing colchicine treatment were included. Patients were divided into three groups: 3 patients had colchicine-resistant FMF, 3 patients had FMF and SpA and 3 patients had homozygous exon 10 mutations in the *MEFV* gene who were responsive to colchicine. PBMCs were stimulated with TcdA to induce pyrin inflammasome activation and treated with the relevant drugs with a concentration validated according to a previous paper were added to the cells.^1^ The cells were further incubated for 6 hours at 37°C with 5% CO2. After 6 hours, supernatants were collected and frozen at −80°C for further analysis. Since we used different biological DMARDs targeting different cytokines, we decided to use S100A8/9 levels in the supernatant of the cell cultures as the read-out of our experiment setup. Quantifications of S100A8/9 were performed by using sandwich ELISA. We calculated the change of inflammation (% decrease in the levels of S100A8/A9) compared to the positive control (TcdA only).

We first compared the effect of tocilizumab, canakinumab and tofacitinib in homozygous colchicine responsive FMF patients and colchicine resistant FMF patients. In a second setting we compared the effect of methotrexate, etanercept, secukinumab, tocilizumab, canakinumab and tofacitinib in homozygous colchicine responsive FMF patients and FMF patients with SpA.

**Results:** We first tested whether different DMARDs (tocilizumab, canakinumab and tofacitinib) affected production of S100A8/A9 by PBMCs of colchicine responsive and resistant homozygous FMF patients. All DMARDs resulted in a decrease in S100A8/A9 production in all cultures. Canakinumab caused the most prominent result in the colchicine-resistant group (25.7%); the effect of tofacitinib was more pronounced (12.6%) than tocilizumab (4.2%). The effect of tocilizumab was more pronounced in the homozygous FMF group responsive to colchicine (26%). Surprisingly, tofacitinib decreased the S100A8/A9 levels in both colchicine responsive (15.5%) and resistant group (12.6%) . Statistical significance was not reached due to the low sample size.

We next evaluated whether different DMARDs (Mtx, etanercept, secukinumab, tocilizumab, canakinumab and tofacitinib) affected production of S100A8/A9 by PBMCs of homozygous FMF patients (colchicine responsive) and FMF patients with SpA. All DMARDs resulted in a decrease in S100A8/A9 production in all cultures except tocilizumab. Among all DMARDs, secukinumab reduced the inflammation most significantly (by 33.3%) in the FMF+SpA patients, more than the effect on the homozygous FMF group (11.3%). Furthermore, canakinumab (24.1% vs 12%), tofacitinib (19.1% vs 15.5%) and methotrexate (7.4% vs 5.9%) also decreased the inflammation more efficiently in FMF+SpA group when compared the homozygous FMF group. In contrast, etanercept and tocilizumab worked better in homozygous FMF group.

**Conclusion:** In *in-vitro* setting, JAK kinase inhibitors showed potential in suppressing inflammation in colchicine-resistant FMF patients, though not as effectively as anti-IL1 treatment. The findings suggest that anti-IL17 and JAK kinase inhibitors might serve as potential alternative treatment options for FMF patients with SpA, providing valuable insights into managing FMF patients resistant to conventional colchicine therapy or experiencing comorbid SpA.

**Patient Consent**

Yes, I received consent

**Disclosure of Interest**

None Declared

**Reference**

1. Nielsen, M.A., et al., *Responses to Cytokine Inhibitors Associated with Cellular Composition in Models of Immune-Mediated Inflammatory Arthritis.* ACR Open Rheumatol, 2020. **2**(1): p. 3-10.

### Autoinflammatory diseases

#### P013 The Eurofever FMF longitudinal cohort: update on the longitudinal data

##### Roberta Caorsi^1^, Marta Bustaffa^1^, Gayane Amaryan^2^, Romina Gallizzi^3^, Efimia Papadopoulou-Alataki^4^, Maria Carrabba^5^, Jordi Anton^6^, Luciana Breda^7^, Gabriele Simonini^8^, Jasmin Kuemmerle-Deschner^9^, Maria Alessio^10^, Donato Rigante^11^, Laura Obici^12^, G. Elizabeth Legger^13^, Antonella Insalaco^14^, Jurgen Brunner^15^, Judith Sánchez-Manubens^16^, Gerd Horneff^17^, Luca Cantarini^18^, Nicolino Ruperto^1^, Marco Gattorno^1^, Seza Ozen^19^

###### ^1^IRCCS Istituto Giannina Gaslini, Genova, Italy; ^2^Arabkir Joint Medical Centre- Institute of Child and Adolescent Health, Yerevan, Armenia; ^3^Azienda Ospedaliera Universitaria Renato Dulbecco di Catanzaro, Catanzaro, Italy; ^4^Papageorgiou General Hospital, Thessaloniki, Greece; ^5^Fondazione IRCCS Ca’ Granda-Ospedale Maggiore Policlinico- Università degli Studi di Milano, Milano, Italy; ^6^Pediatric Rheumatology Department, Hospital Sant Joan de Déu, University of Barcelona, Esplugues de Llobregat, Barcelona, Spain; ^7^Ospedale Policlinico – Universita’ degli studi di Chieti, Chieti; ^8^Azienda Ospedaliero Universitaria Meyer, Firenze; ^9^University Children’s Hospital Tuebingen, Tuebingen; ^10^Universita’ di Napoli Federico II, Napoli; ^11^Cattolica Sacro Cuore University, Roma; ^12^Fondazione IRCCS Policlinico San Matteo, Pavia; ^13^Beatrix Kinderkliniek, University Medical Center, Groningen; ^14^Ospedale Pediatrico Bambino Gesu’, Roma, Italy; ^15^Medical University Innsbruck, Innsbruck, Austria; ^16^Hospital de Sabadell, Barcelona, Spain; ^17^Asklepios Children’s Hospital Sankt Augustin, Sankt Augustin, Germany; ^18^Policlinico le Scotte, Universita’ di Siena, Siena, Italy; ^19^Hacettepe University Children’s Hospital, Ankara, Türkiye

####### **Correspondence:** Roberta Caorsi

*Pediatric Rheumatology 2024*, **22(2):**PReS24-ABS-1688

**Introduction:** FMF is the most frequent monogenic autoinfiammatory disease, secondary to mutation of MEFV gene. By now, few long-term follow-up data are available on the efficacy and safety of different treatments available.

**Objectives:** In 2021 we described the baseline clinical information about 887 FMF patients enrolled in the Eurofever registry since 2009. We describe now the first longitudinal data about these patients.

**Methods:** Patients with FMF enrolled in Eurofever registry with at least one follow-up visit were included in a longitudinal study. Demographic and clinical data were analyzed. Disease activity and maximum dose of colchicine were defined according to the 2016 EULAR recommendation.

**Results:** 1200 patients with FMF are enrolled in Eurofever registry in April 2024, 574 males and 530 females. The median age at disease onset was 3.8 (0.4 - 27.9); the median diagnostic delay was 2.4 (0.1 – 29.8); the median age at enrollment was 7.8 (1.7 – 51.1). Follow-up information was available in 671 patients, with a mean number of follow-up visits of 2.1 (IQR 1-3) and a mean duration of follow-up of 4.4 years (IQR 1.9 – 8.5). At the last follow-up 48.7% of patients in the longitudinal cohort were in complete remission, 35.3% experienced some disease activity (<1 episode/month) while only 7.4% experienced ≥ 1 episode/month and are therefore considered resistant to treatment according to the 2016 EULAR Guidelines.

At last follow-up visit 85.4% of patients of the longitudinal cohort were receiving colchicine, with a median duration of treatment of 5.9 years (IQR 2.0 – 8.4). Among resistant patients only 1 reached maximum recommended dose per age of colchicine according EULAR Guidelines (3patients in partial remission, only 4 patients in complete remission). Moreover, at last follow-up 53% of resistant patients were still receiving the recommended starting dose of colchicine according EULAR Guidelines (74.2% of patients in complete remission, 45.5% of patients in partial remission). 10.4% of patients were on canakinumab while 1.3% were receiving anakinra. Among patients receiving biological treatments, 73 (80.2%) were receiving a combination of colchicine and biologics. Rate of withdrawal for colchicine were 9.7%, for canakinumab 6.7% while for anakinra was higher (21.5%).

**Conclusion:** This study analyzes the initial data concerning the natural history of the Eurofever FMF cohort. At the last follow-up less than 50% of the patients have achieved complete disease control. Colchicine is the most commonly used treatment. Among biologic drugs, IL-1 inhibitors are the most frequently used, with a good treatment retention rate at the last follow-up, especially for anti-IL-1 monoclonal antibody.

**Patient Consent**

Yes, I received consent

**Disclosure of Interest**

R. Caorsi: None Declared, M. Bustaffa: None Declared, G. Amaryan: None Declared, R. Gallizzi: None Declared, E. Papadopoulou-Alataki: None Declared, M. Carrabba: None Declared, J. Anton Grant / Research Support with: N/A, Consultant with: N/A, Speaker Bureau with: N/A, L. Breda: None Declared, G. Simonini: None Declared, J. Kuemmerle-Deschner: None Declared, M. Alessio: None Declared, D. Rigante: None Declared, L. Obici: None Declared, G. E. Legger: None Declared, A. Insalaco: None Declared, J. Brunner: None Declared, J. Sánchez-Manubens: None Declared, G. Horneff: None Declared, L. Cantarini: None Declared, N. Ruperto: None Declared, M. Gattorno: None Declared, S. Ozen: None Declared

### Autoinflammatory diseases

#### P014 Observational French cohort study on patients with H syndrome (SLC29A3-related disorders): exploring inflammatory and autoimmune manifestations

##### Maurine Jouret^1^, Bénédicte Neven^2^, Brigitte Bader-Meunier^2^, Pascal Pillet ^3^, Arthur Felix^4^, Mathieu Gerfaud-Valentin^5^, Julien Haroche^6^, Stephane Barete^7^, Guilaine Boursier^8^, Isabelle Melki^9^, Jeremie Rosain^10^, Heloise Reumaux^11^, Laurence Jonard^12^, Pauline Le Tanno^13^, Sebastien De Almeida^14^, Guillaume Le Guenno^15^, Estibaliz Lazaro^16^, Jean François Emile^17^, Alexandre Belot^1^, Jean Donadieu^18^ on behalf of Histiocytosis Study Group

###### ^1^Department of Pediatric Rheumatology, Dermatology, and Nephrology, Hospices Civils de Lyon, Lyon; ^2^Department of Pediatric Immunohematology and Rheumatology, Necker-Enfants Malades Hospital, Paris; ^3^General Pediatrics, Pediatric Rheumatology, and Pediatric Internal Medicine, Children’s Hospital, Bordeaux; ^4^Department of General Pediatrics, University Hospital of Martinique, Fort-de-France; ^5^Internal Medicine, Saint-Joseph Saint-Luc Hospital, Lyon; ^6^Internal Medicine; ^7^Dermatology, Pitié-Salpêtrière Hospital, Paris; ^8^Genetic, A. De Villeneuve Hospital, Montpellier; ^9^General Paediatrics, Infectious Disease and Internal Medicine Department, Hôpital Robert Debré; ^10^Inserm U1163, Laboratory of Human Genetics of Infectious Diseases, Paris; ^11^Pediatric Rheumatology Service, Jeanne de Flandres Hospital, University of Lille, Lille; ^12^Molecular Genetics Laboratory, Reference Center for Genetic Hearing Impairments, Hôpital Necker-Enfants Malades, Paris; ^13^Department of Medical Genetics, CHU Grenoble, Grenoble; ^14^Internal medecine, University Hospital of Toulouse, Toulouse; ^15^Internal Medecine, Estaing University Hospital Center, Clermont Ferrand; ^16^Department of Internal Medicine and Infectious Diseases, University Hospital Centre, Bordeaux; ^17^Department of Pathology, Ambroise-Paré Hospital, Boulogne; ^18^Pediatric Hematology, Trousseau Hospital , Paris, France

####### **Correspondence:** Maurine Jouret

*Pediatric Rheumatology 2024*, **22(2):**PReS24-ABS-1256

**Introduction:** The H syndrome is an autoinflammatory disorder associated with mutations in the *SLC29A3* gene, classified within the R group of histiocytosis. Recent studies have suggested that Toll-like receptor (TLR7/8) activation is due to impaired nucleoside trafficking, resulting in persistent activation of ERK and histiocytosis proliferation.

**Objectives:** This cohort study aims to investigate the long-term evolution over several years and evaluate the efficacy of novel therapeutic interventions for individuals with H Syndrome.

**Methods:** In November 2023, an observational call was distributed through various nationwide rare disease networks and French adult and pediatric scientific societies. Inclusion criteria involved individuals, whether children or adults, diagnosed with H syndrome. Demographic, clinical, and biological data were collected using an electronic database called “Histiobase.”

**Results:** In May 2024, thirty patients from France were enrolled. The gender distribution was balanced, with a median age at inclusion of 23 years (ranging from 0.5 to 58 years). Patients exhibited diverse inflammatory phenotypes including skin hyperpigmentation or induration (n=19), adenopathy enlargement (n=15), articular deformities (n=16), arthritis (n=8), kidney involvement (tubular (n=3) and glomerular lesions (n=4)), pericarditis (n=5), aortic inflammation (n=2), pachymeningitis (n=2), and macrophagic activation syndrome (n=1). A few patients also showed signs of autoimmunity, such as autoimmune cytopenia (n=1), ANCA-vasculitis with MPO antibodies (n=1), type 1 diabetes with specific autoantibodies (n=2), and antinuclear antibodies (n=2). Two patients experienced infectious complications associated with lymphocyte B deficiency. Twenty patients were found to have deafness. Some severe complications could be attributed to histiocyte infiltrations, such as ureteral compression (n=3), cardiac mass (n=1), and peridural infiltration (n=2). Thirteen patients received treatment with Tocilizumab, 5 with Cobimetinib (MEK inhibitor), and 2 with anti-TNF biotherapies, resulting in complete or partial responses to inflammatory and proliferative manifestations. H syndrome can mimick various rheumatologic diseases. Prior to genetic analysis, diagnoses retained included idiopathic juvenile arthritis, IgG4-related syndrome, systemic scleroderma, Erdheim-Chester disease, periarteritis nodosa, unclassified auto-inflammatory disease and immune deficiency.

**Conclusion:** H syndrome presents as a rare condition with a wide spectrum of systemic clinical features, encompassing proliferative, autoinflammatory, autoimmune manifestations, and immune deficiency. Although promising therapeutics show efficacy, their long-term effectiveness needs assessment. An international cohort study could offer more insights to enhance patient management.

**Patient Consent**

Yes, I received consent

**Disclosure of Interest**

None Declared

**Reference**

1. Shiloh R, Lubin R, David O, Geron I, Okon E, Hazan I, et al. Loss-of-function of ENT3 drives histiocytosis and inflammation through TLR-MAPK signaling. Blood Journal. 22 sept 2023;blood.2023020714.

### Autoinflammatory diseases

#### P015 Use of type I interferon score as a biomarker of disease activity in ADA2 deficiency

##### Silvia Federici^1^, Marjon Wouters^2^, Valentina Matteo^3^, Anneleen Hombrouck^2^, Giusi Prencipe^3^, Leen Moens^2^, Isabelle Meyts^4,5^, Antonella Insalaco^1^

###### ^1^Rheumatology, Bambino Gesù children’s Hospital, Rome, Italy; ^2^Laboratory of Inborn Errors of Immunity, Department of Microbiology, Immunology and Transplantation, KU Leuven, Leuven, Belgium; ^3^Laboratory of Immuno-Rheumatology, Bambino Gesù children’s Hospital, Rome, Italy; ^4^Department of Microbiology, Immunology and Transplantation, Inborn Errors of Immunity, KU Leuven; ^5^Childhood Immunology, Department of Pediatrics, Leuven University Hospitals, Leuven, Belgium

####### **Correspondence:** Silvia Federici

*Pediatric Rheumatology 2024*, **22(2):**PReS24-ABS-1368

**Introduction:** Deficiency of adenosine deaminase 2 (DADA2) is a rare systemic autoinflammatory disease with autosomal recessive inheritance caused by biallelic loss of function mutations in the ADA2 gene. The phenotypic spectrum of the disease is broad, including fever, early-onset vasculitis, stroke, immunologic and hematologic dysfunction. If not recognized and treated the disease can provoke mortality and serious long-term sequelae. Although the exact mechanism involved in the pathogenesis of the disease is still unclear, in patients with DADA2 an increased type I IFN score (IS), correlated with disease activity and response to treatment, has been demonstrated. Recently an activity and damage score (DADA2AI and DADA2DI) have been developed^1^ to aid in the longitudinal assessment of these patients, inter- and intra-patients comparisons and in the evaluation of long term outcome with different types of therapy.

**Objectives:** To apply the recently published DADA2AI and DADA2DI in a bicentric cohort of DADA2 patients and to evaluate whether longitudinal follow-up of type I IS reflects disease activity and could potentially be used as a biomarker of the disease.

**Methods:** ADA2 enzymatic activity was measured in a colorimetric assay adapted from Giusti et al (1974). An HEK293T overexpression system was setup to evaluate the impact of suspected pathogenic ADA2 variants on protein expression/secretion and enzymatic activity. Real time PCR assays were performed on whole blood cells, the expression levels of 6 interferon-induced genes was evaluated and the IS was calculated^2^. DADA2AI was calculated based on criteria described by Bucciol et al.

**Results:** 14 patients from 11 families were included. All patients carried 2 pathogenic ADA2 variants and showed reduced serum ADA2 activity. Twelve patients presented with an inflammatory vasculitis phenotype while 2 had an hematological one. One patient displayed hypogammaglobilinemia. Enzymatic activity of patient ADA2 variants in an HEK2937 overexpression system correlated with residual enzymatic activity measured in patient serum samples. Longitudinal follow-up of type I IS was performed in all patients in combination with assessment of DADA2 activity index. In our cohort, IS score was only slightly elevated in the majority of patients (median value of 4.055 versus normal IS <2.05 ) and did not correlate with DADA2AI or with routine laboratory parameters. The DADA2DI remain stable or decrease in all patients with the inflammatory vasculitis phenotype treated with TNF-inhibitor thus confirming the efficacy of this treatment in preventing new damage/sequelae

**Conclusion:** The DADA2AI and DADA2DI can be easily calculated from the patients charts and may represent a valid and reproducible instrument to monitor patient’s disease activity and response to treatment. The pathogenetic mechanism in DADA2 is not entirely understood and type I IFN pathway activation is probably part of a much more complex mechanism. In our cohort, 6-gene type I IFN score did not prove to be a good biomarker for longitudinal follow-up of DADA2 disease activity and response to treatment.

**Funding**

IM, MW, are supported by grant G0B5120N from Research Foundation Flanders, and grant 948959 from the European Research Council under the European Union’s Horizon 2020 research and innovation program.

**Patient Consent**

Not applicable (there are no patient data)

**Disclosure of Interest**

None Declared

**References**

1. Bucciol et al, J. Clin Immunol. 2023
2. Rice et al J. Clin Immunol. 2017

### Autoinflammatory diseases

#### P016 The diagnostic and therapeutic challenges of chronic arthritis in familial Mediterranean fever

##### Eray Tunce, Betül Sözeri

###### Pediatric Rheumatology, Umraniye Training and Research Hospital, İstanbul, Türkiye

####### **Correspondence:** Eray Tunce

*Pediatric Rheumatology 2024*, **22(2):**PReS24-ABS-1737

**Introduction:** Familial Mediterranean Fever (FMF) and Juvenile Idiopathic Arthritis (JIA) are two significant rheumatological diseases commonly seen in childhood. FMF is an autosomal recessive disorder typically observed in populations of Mediterranean origin, characterized by recurrent episodes of fever, peritonitis, pleuritis, and arthralgia/arthritis. FMF is an autoinflammatory disease, while JIA is an autoimmune disease. It is not yet fully understood whether chronic arthritis in FMF patients is related to FMF itself or occurs independently of FMF.

**Objectives:** We aimed the diagnostic and therapeutic challenges of chronic arthritis in patients with FMF.

**Methods:** This study was a retrospective analysis of pediatric FMF patients followed at our pediatric rheumatology clinic between 2016 and 2023. A total of 472 pediatric patients diagnosed with FMF and having a homozygous genotype in exon 10 were included in the study, and out of these patients, 32 who developed chronic arthritis during follow-up were evaluated. We tried to include the 32 patients with FMF diagnosis who developed chronic arthritis during follow-up in the JIA subgroup. The 2019 Eurofever/PRINTO criteria were used to diagnose AAA and the 2001 ILAR criteria were used to diagnose JIA. Demographic and clinical data were recorded from the patients’ medical charts.

**Results:** A total of 472 children (251 (53,2%) female and 221 male) were included in the study. The median (IQR; 25 - 75) of age disease onset and age at diagnose was 3.5 (1.8-6) years and 5 (3-8) years, respectively. Parental consanguinity was present in 36.2% of patients and 58% had a family history of FMF. The median of following time was 32 (18-43) months. Genotypes of patients: 402 (85,2%) M694V/M694V, 38 (8,1%) M680I/M680I, 23 (4,9%) V726A/V726A, 8 (1,7%) R761H/R761H, 1 (0,2%) I641F/I641F.

Chronic arthritis was observed in 32 out of 472 patients (6.8%) during follow-ups. 17 (53,1%) were female. The median (IQR; 25 - 75) of age disease onset and age at diagnose was 5 (3-8) years and 7 (5-15) years, respectively. Parental consanguinity was present in 25% of patients and 50% had a family history of FMF. Genotypes of patients: 30 (94%) M694V/M694V, 1 (3%) M680I/M680I, 1 (3%) V726A/V726A. JIA subgroups were as follows: 16 (50%) oligoarticular JIA, 10 (31,3%) juvenile spondyloarthritis (SpA), 2 (6,3%) polyarticular JIA, 1 (3,1%) psoriatic arthritis (PsA), 2 (6,3%) systemic JIA and 1 (3,1%) unclassified arthritis. In patients, ANA positivity rate was 25% and HLA B27 positivity rate was 18,6% (The HLA-B27 test was able to be performed in 27 patients.). RF and CCP values were negative in 2 patients with polyarticular JIA. Only 1 patient was found to have uveitis, and that patient had SpA. Complete response to disease-modifying antirheumatic drugs (methotrexate, salazoprine, leflunomide) was achieved in 34,4% of patients, while 65,6% needed biological agents (mainly etanercept, adalimumab, anakinra, kanakinumab, infliximab). Biological treatment was required in 62,5% of oligoarticular JIA, 60% of SpA, 100% of polyarticular JIA, 100% of PsA, 50% of systemic JIA and 100% of unclassified arthritis.

**Conclusion:** Chronic arthritis was observed in 32 out of 472 patients (6.8%) during follow-ups in FMF patients. In the group with chronic arthritis, the age at onset of attacks and diagnosis was higher. The need for biological therapy in this group was 65.6%, significantly higher than in JIA studies in the literature.

**Date of birth:** septembre

**Patient Consent**

No, I have not received consent

**Disclosure of Interest**

None Declared

### Autoinflammatory diseases

#### P017 Urine mevalonate excretion – a specific and sensitive screening test for mevalonate kinase deficiency

##### Šárka Fingerhutová^1^, Petr Chrastina^1^, Lenka Dvořáková^1^, Kateřina Šingelová^1^, Eva Jančová^2^, Pavla Doležalová^1^

###### ^1^Department of Paediatrics and Inherited Metabolic Disorders, General University Hospital in Prague and 1st Faculty of Medicine, Charles University; ^2^Clinic of Nephrology, General University Hospital in Prague and 1st Faculty of Medicine, Charles University , Prague, Czech Republic

####### **Correspondence:** Šárka Fingerhutová

*Pediatric Rheumatology 2024*, **22(2):**PReS24-ABS-1552

**Introduction:** Detection of mevalonate in urine of patients with periodic fever (PF) may aid the diagnosis of mevalonate kinase deficiency (MKD) as a fast and cheap screening test complementing genetic analysis. Little is known about dynamics of mevalonate excretion during febrile attacks and about sensitivity of single urine sample against collected urine.

**Objectives:** To evaluate the sensitivity and specificity of urine mevalonate in patients with PF. To assess dynamics of mevalonate excretion during febrile attacks in MKD patients.

**Methods:** Consecutive patients with PF (including MKD) were instructed to take a sample of urine collected from 3 subsequently voided portions on the 1^st^ or 2^nd^ day od febrile episode. Additionally, MKD patients were asked to provide single-voided samples one from each of 5 consecutive days of fever and a sample from afebrile interval. Urine samples were assayed by gas chromatography-mass spectrometry and quantified after an ethyl acetate extraction and ethoxime-trimethylsilyl derivatization. Presence of any amount of mevalonolactone was considered abnormal.

**Results:** 527 urine samples from febrile episodes of 435 patients with PF other than MKD (mostly periodic fever-aphtae-pharyngitis-adenitis (PFAPA) syndrome) were tested between 2021 and 2023. All samples were negative. From 12 genetically confirmed MKD patients, 63 samples from collected urine were available and all were positive for mevalonolactone. 4 MKD patients provided 20 consecutive single-voided samples from febrile episodes. All but one sample (from day 1 of fever) were also positive as were single urine samples from afebrile interval of the same patients. There was no trend detected in the dynamics of amount of mevalonate excreted during febrile episodes.

**Conclusion:** Absence of mevalonate in all febrile urine samples of substantial number of children with PF and its presence in all patients with MKD proves very high sensitivity and specificity of the test. During febrile episodes single-voided samples seem to have comparable sensitivity as collected urine, regardless the timing of sampling. Nevertheless, higher number of MKD single-voided samples need to be tested before we fully change our current practice of testing collected urine samples as a screening for MKD in PF patients.

**Patient Consent**

Yes, I received consent

**Disclosure of Interest**

Š. Fingerhutová: None Declared, P. Chrastina: None Declared, L. Dvořáková: None Declared, K. Šingelová: None Declared, E. Jančová: None Declared, P. Doležalová Consultant with: Novartis, Sobi, Speaker Bureau with: Novartis, Sobi

**Reference**

1. Supported by the Czech Health Research Council (AZV CR) grant NU21-05-00522

### Autoinflammatory diseases

#### P018 Exploring the factors for predicting colchicine responsiveness in children with PFAPA

##### Zeynep Özaslan^1^, Abdulvahap Şen^2^, Sıla Atamyıldız Uçar ^3^, Mustafa Çakan^4^, Bengisu Sanisoğlu^5^, Feray Kaya^6^, Gülçin Otar Yener^7^, Ferhat Demir^8^, Ayşe Tanatar^9^, Semanur Özdel^2^, Kübra Öztürk^6^, Nihal Şahin^1^, Hafize E. Sönmez^1^, Nuray Aktay Ayaz^5^, Betül Sözeri^3^

###### ^1^Department of Pediatric Rheumatology, Kocaeli University Faculty of Medicine, kocaeli; ^2^Department of Pediatric Rheumatology, Etlik City Hospital, Ankara; ^3^Department of Pediatric Rheumatology, Health Sciences University, Umraniye Training and Research Hospital; ^4^Department of Pediatric Rheumatology, Zeynep Kamil Training and Research Hospital; ^5^Department of Pediatric Rheumatology, İstanbul University; ^6^Department of Pediatric Rheumatology, Göztepe Training and Research Hospital, İstanbul; ^7^Department of Pediatric Rheumatology, Eskişehir City Hospital, Eskişehir; ^8^Department of Pediatric Rheumatology, Acıbadem Ataşehir Hospital, İstanbul; ^9^Department of Pediatric Rheumatology, Gaziantep City Hospital, Gaziantep, Türkiye

####### **Correspondence:** Zeynep Özaslan

*Pediatric Rheumatology 2024*, **22(2):**PReS24-ABS-1066

**Introduction:** Periodic fever, aphthous stomatitis, pharyngitis, and cervical adenitis syndrome (PFAPA) is the most common autoinflammatory syndrome in children. Since there are no typical laboratory findings specific to the disease, diagnosis is often delayed, and treatment approaches vary. In this study, we aimed to examine the response to colchicine in PFAPA disease and evaluate whether parameters predicting the response to colchicine based on first-line clinical and laboratory parameters.

**Objectives:** Nine referral centers from our country enrolled in the study. Parameters were documented from the patient’s medical records, retrospectively. The diagnosis of PFAPA syndrome was made according to modified Marshall’s criteria. Patients whose disease episodes ceased or became less frequent after colchicine treatment were classified as “colchicine responsive.”

**Results:** A total of 806 patients enrolled in the study. Fever was the most common clinical finding, reported in all patients, followed by exudative tonsillitis in 697 (86.5%), pharyngitis in 652 (80.9%), aphthous stomatitis in 407 (50.5%), cervical lymphadenopathy in 340 (42.2%), abdominal pain in 253 (31.4%), arthralgia in 191 (23.7%), headache in 34 (4.12%) and arthritis in 8 (1%) patients. The median age of diagnosis and symptom onset were 44 (9-151) and 24 (3-120) months, respectively. The mean attack frequency was 13.5 ± 6.8 attacks per year lasting for a mean of 3.9 ± 1.1 days. Colchicine treatment was attempted in 519 (64.4%) patients, with 419 (80.7%) showing a favorable response and 12 (2.3%) showing a partial response. MEFV gene sequencing was performed on 571 (70.8%) patients, revealing no genetic mutation in 335 (58.1%) patients, while the most common variant was M694V heterozygote in 96 (16.8%) patients. Clinical and laboratory parameters of patients were compared based on their responses to colchicine (Table 1). A multivariate regression analysis identified several factors associated with colchicine unresponsiveness, including the presence of pharyngitis (p=0.03, CI95% 0.885 to 0.994), the presence of arthralgia (p=0.04, CI95% 0.169 to 0.958), and more frequent attacks (p= 0.001, CI95% 0.028 to 0.748). Carrying the M694V variant (p= 0.001, CI95% 0.065 to 0.242) was the sole factor predicting colchicine responsiveness.

**Conclusion:** Our study identified significant predictors of colchicine unresponsiveness in PFAPA disease, including the presence of pharyngitis, arthralgia, and increased attack frequency. Conversely, the presence of the M694V variant carriage emerged as a significant predictor of colchicine responsiveness. These findings underscore the potential utility of predicting colchicine response at disease onset, thereby facilitating more effective management of PFAPA disease.

**Date of birth::** 16.11.1990

**Patient Consent**

Yes, I received consent

**Disclosure of Interest**

None Declared

### Autoinflammatory diseases

#### P019 The hints to differentiate cryopyrin-associated periodic syndrome from acute/recurrent and acute urticaria

##### Seher Sener^1^, Deniz I. Guler^2^, Aybuke Gunalp^3^, Emine N. Sunar Yayla^4^, Sengul Caglayan^5^, Ceyda Arslanoglu^6^, Kubra Zora^7^, Ozge Baba^8^, Elif Kilic Konte^3^, Ezgi D. Batu^1^, Ozge Basaran^1^, Aysenur Pac Kisaarslan^6^, Sara S. Kilic^7^, Betul Sozeri^5^, Mukaddes Kalyoncu^8^, Umit M. Sahiner^2^, Yelda Bilginer^1^, Ozgur Kasapcopur^3^, Ozge Soyer^2^, Seza Ozen^1^

###### ^1^Pediatric Rheumatology; ^2^Pediatric Allergy and Immunology, Hacettepe University, Ankara; ^3^Pediatric Rheumatology, Istanbul Cerrahpasa University, Istanbul, ^4^Pediatric Rheumatology, Ankara Etlik City Hospital, Ankara; ^5^Pediatric Rheumatology; Umraniye Training and Research Hospital, Istanbul; ^6^Pediatric Rheumatology, Erciyes University Faculty of Medicine, Kayseri; ^7^Immunology and Rheumatology, Uludag University, Bursa; ^8^Pediatric Rheumatology, Karadeniz Technical University, Ankara, Türkiye

####### **Correspondence:** Seher Sener

*Pediatric Rheumatology 2024*, **22(2):**PReS24-ABS-1134

**Introduction:** Cryopyrin-associated periodic syndrome (CAPS) is a very rare disease occurring in an estimated one to three per million children worldwide. It is one of the autoinflammatory diseases that may occur with urticaria-like rashes. Hence, it is crucial and difficult to differentiate between urticaria-like rash in CAPS and recurrent acute urticaria.

**Objectives:** This study aimed to evaluate the similarities and differences between acute urticaria, CAPS rash, and acute recurrent urticaria.

**Methods:** This multicenter study involved patients with CAPS (Muckle wells syndrome [MWS] or familial cold autoinflammatory syndrome [FCAS]) (0-18 years of age) from eight different centres. All CAPS patients fulfilled clinical and/or genetic EUROFEVER/PRINTO classification criteria. The study comprised pediatric patients with acute and recurring urticaria who underwent follow-up at Hacettepe University Pediatric Allergy Clinic from June 2016 to June 2023 for the purpose of performing comparisons.

**Results:** A total of 153 patients were included in the study. Of these patients, 60 were CAPS (median age 1.0 [IQR 0.3-1.2], M/F=35/25), 57 were patients with acute urticaria (median age 1.8 [IQR 1.2- 3.0], M/F=35/22), and 36 were patients with recurrent acute urticaria (median age 2.0 [IQR 1.0-4.8], M/F=19/17). Of the CAPS patients, 31 were diagnosed as MWS and 29 were as FCAS. Significantly, those with CAPS had a younger age at which the disease presented compared to the other two groups, but were diagnosed at a later age (p<0.001). The logistic regression analysis revealed family history of autoinflammatory disease (OR 6.42, CI 1.08-38.06, p=0.040), exposure to cold as a triggering factor (OR 7.24, CI 1.19-43.82, p=0.030), duration of urticaria (≤2 days) (OR 7.55, CI 2.04-27.86, p=0.002), absence of itching (OR 4.03, CI 1.13-14.37, p=0.030), and the requirement for use of corticosteroids for improvement of rashes (OR 6.90, CI 1.88-25.30, p=0.004) as independent risk factors for diagnosing CAPS.

**Conclusion:** CAPS, characterized by clinical signs such as a rash resembling urticaria, is a challenging autoinflammatory disease to identify. The results of our study emphasize the specific clinical characteristics that distinguish CAPS-related urticaria-like rash from acute and acute recurrent urticaria. Our findings highlight distinct clinical features that differentiate CAPS-related urticaria-like rash from acute and acute recurrent urticaria, providing valuable insights for diagnostic and therapeutic considerations.

**Date of birth:** 09.10.1989

**Patient Consent**

Yes, I received consent

**Disclosure of Interest**

None Declared

### Autoinflammatory diseases

#### P020 Chronic Nonbacterial Osteomyelitis / Chronic Recurrent Multifocal Osteomyelitis (CNO / CRMO): looking beyond the bone

##### Aarat Patel^1,2^, Kelley Lee^1^, Ashley Kim^2^

###### ^1^Rheumatology, Bon Secours Mercy Health, Richmond; ^2^Pediatrics, University of Virginia, Charlottesville, United States

####### **Correspondence:** Aarat Patel

*Pediatric Rheumatology 2024*, **22(2):**PReS24-ABS-1516

**Introduction:** Chronic nonbacterial osteomyelitis/chronic recurrent multifocal osteomyelitis (CNO/CRMO) is an aseptic autoinflammatory bone disease of unknown aetiology. The characteristic feature of CNO/CRMO is bone pain of insidious onset with possible swelling, tenderness and/or warmth over the affected area. Silent lesions are common and CNO/CRMO can be episodic with unifocal or multifocal involvement. The disease itself is rare and likely underdiagnosed. Some patients will go months to years without a diagnosis. Extra-osseous (EO) manifestations are possible and can help lead to the diagnosis. The frequency of these EO manifestations is not completely known. Previous cohort studies have shown EO manifestations lead to a more severe disease. Standard treatments include nonsteroidals, conventional DMARDs, anti-TNF inhibitors, JAK inhibitors, and bisphosphonates. The treatment goal is the resolution of bone pain, swelling and control of EO manifestations if present.

**Objectives:** Our objective is to describe the frequency of EO manifestations in a cohort of CNO/CRMO patients. We will also compare the use of biologics/JAK inhibitors in those with and without EO manifestations.

**Methods:** This retrospective study included CNO/CRMO patients followed from 2012 to 2024 in Virginia, USA (n=42) at the University of Virginia in Charlottesville and at the Bon Secours Rheumatology Center in Richmond. We looked for six EO manifestations: inflammatory arthritis, sacroiliitis, palmoplantar pustulosis (PPP), psoriasis, inflammatory bowel disease and uveitis. Charts were reviewed for treatments used in each patient. Fisher’s exact test was used to show statistical significance.

**Results:** The newly proposed classification criteria for paediatric CNO were met by 95% of patients (n=40). Two did not meet these criteria because of their age at diagnosis and elevated inflammatory markers. The average age of the group at diagnosis was 10 years-old (range 2-17) with 51% being female. The average time to diagnosis was 24 months (range 1-120). Any of the six EO manifestations were seen in 48% of patients (n=20). The most common being inflammatory arthritis (40%; n=17) and sacroiliitis (33%; n=14). Some of these patients did overlap with both (21%; n=9). Skin EO manifestations were seen much less with PPP in two (5%) and psoriasis in three (7%) patients, respectively. Even less common were IBD (2%; n=1) and uveitis (2%; n=1). Biologics and JAK inhibitors were necessary in more patient with EO manifestations than without. These medicines were used in 76% of those with inflammatory arthritis versus in 28% of those without inflammatory arthritis (p<0.005). These medicines were used in 86% of those with sacroiliitis versus 25% in those without sacroiliitis (p<0.005). For those that had both inflammatory arthritis and sacroiliitis, 88% required biologics and/or JAK inhibitors.

**Conclusion:** This retrospective analysis in CNO/CRMO shows that the most common EO manifestations in this cohort are inflammatory arthritis and sacroiliitis. As noted in other similar studies, those with EO manifestations have severe disease that more often require biologics and JAK inhibitors. However, in this cohort, skin manifestations, inflammatory bowel disease, and uveitis were not as commonly seen as shown in other cohorts.

**Patient Consent**

Yes, I received consent

**Disclosure of Interest**

None Declared

**References**

1. PMID: 37698983
2. PMID: 36456962

### Autoinflammatory diseases

#### P021 Disease presentation, response to treatment and outcome of pediatric and adult patients with DADA2 (Deficiency of Adenosine Deaminase 2): results from the Eurofever registry

##### Roberta Caorsi^1^, Seza Ozen^2^, Antonella Insalaco^3^, Chiara Conti^4^, Joost Frenkel^5^, Nicola Ruperto^6^, Marco Gattorno^1^ and The Paediatric Rheumatology International Trial Organization (PRINTO) and the Eurofever Project

###### ^1^Rheumatology and autoinflammatory diseases, IRCCS Istituto Giannina Gaslini, Genova, Italy; ^2^Department of Pediatric Rheumatology, Hacettepe University, Ankara, Turks and Caicos Islands; ^3^Rheumatology, IRCCS Ospedale Pediatrico Bambino Gesù, Roma; ^4^University of Genova, Genova, Italy; ^5^Pediatric rheumatology, University hospital of Utrecht, Utrecht, Netherlands; ^6^Trial Center, IRCCS Istituto Giannina Gaslini, Genova, Italy

####### **Correspondence:** Roberta Caorsi

*Pediatric Rheumatology 2024*, **22(2):**PReS24-ABS-1596

**Introduction:** DADA2 is a monogenic autoinflammatory condition characterised by a broad spectrum of clinical manifestation, ranging from cutaneous to severe systemic vasculitis with multiorgan involvement, immunodeficiency and bone marrow failure. Few data are nowadays available about the clinical characteristic, the response to treatment and the outcome of this disease.

**Objectives:** to analyse the data of the DADA2 patients enrolled in the Eurofever registry.

**Methods:** the data analysed were extracted from the Eurofever registry, which is hosted in the PRINTO website. The patients were included in the study in the presence of clinical manifestations consistent with DADA2, a confirmatory genotype or a pathologic ADA2 enzymatic activity. Demographic data, clinical manifestations, treatment, safety and outcome were analysed.

**Results:** In May 2024 baseline and clinical information were available of 92 DADA2 patients (43M:49F), from 23 centers, in the Eurofever registry; of these, follow-up data were available for 63 patients (mean follow-up duration 2.8 years). 71 patients (77%) had a confirmatory genotype, while 21 a non-confirmatory genotype. 17 patients had a positive family history (12 couples of siblings were included in the registry). The mean age at enrolment was 12.5 years (74 paediatric and 18 adult patients), at disease onset 7,4 years (SD 8,6) and at diagnosis 15.2 years (SD 12), with a mean delay of 8 years.

The disease course was continuous in 46% of patients, recurrent in 27%, continuous with recrudescence in 27%. 81% of patients presented skin involvement during their disease course, 58% neurological, 53% musculoskeletal, 41% gastrointestinal, 38% of lymphoid organs, 24% haematological, 17% cardiovascular, 14% ocular and 5% genitourinary. In 4 patients a neoplasm occurred.

Synthetic DMARDS were used in 34 patients. Azathioprine and cyclophosphamide, used in 10 and 3 patients respectively, were withdrawn for inefficacy; thalidomide, used in 3 patients, was withdrawn for side effects. Mofetil mycophenolate was used in 6 patients, still ongoing at last follow-up in 2, while methotrexate in 14 patients, still ongoing at last follow-up in 8, of those 5 associated to anti-TNF.

76 patients (82%) received treatment with anti-TNF: 66 patients were treated with etanercept (in 63 treatment was ongoing al last follow-up), 17 patients with adalimumab (in 10 treatment was ongoing at last follow-up). IL-1 and 6 inhibitors were used in 7 and 1 patients respectively, withdrawn for inefficacy.

24 adverse events were reported, while on treatment; of these, 11 were serious: 2 were disease-related, 3 resolved with surgery, 4 were infections and one (hepatic nodular hyperplasia) required drug-change. One patient attempted suicide while on biological treatment.

79% of patientsa chieved a complete control of the disease during follow-up.. 2 patients died: one for sepsis at the age of 52, one for lung cancer at the age of 28.

**Conclusion:** the study analyses a large series of DADA2 patients with prolonged follow-up, confirming the clinical heterogeneity of this condition and the difficulty in the molecular diagnosis. Anti-TNF drugs confirms their efficacy and good safety profile in this condition.

**Patient Consent**

Yes, I received consent

**Disclosure of Interest**

None Declared

### Autoinflammatory diseases

#### P022 Clinical characteristics and biomarkers of patients with non-hereditary periodic fever syndromes in a Czech single centre cohort

##### Anna Doležalová^1^, Šárka Fingerhutová^1^, Blanka Stibůrková^2^, Jana Mašínová^2^, Nikol Vinšová^1^, Prokop Šeferna^1^, Markéta Pavlíková^3^, Hana Hulejová^2^, Pavla Doležalová^1^

###### ^1^Department of Paediatrics and Inherited Metabolic Disorders, General University Hospital and 1st Faculty of Medicine, Charles University; ^2^Institute of Rheumatology and Department of Rheumatology, 1st Faculty of Medicine, Charles University; ^3^Department of Probability and Mathematical Statistics, Faculty of Mathematics and Physics, Prague, Czech Republic

####### **Correspondence:** Anna Doležalová

*Pediatric Rheumatology 2024*, **22(2):**PReS24-ABS-1606

**Introduction:** Non-hereditary periodic fevers encompass syndromes lacking a known causal gene. Although periodic fever-aphtae-pharyngitis-adenitis syndrome (PFAPA) is the most prevalent among Caucasian children, some individuals are categorized under the syndrome of undifferentiated recurrent fever (SURF). Nevertheless, proposed classification criteria for SURF are yet to be validated.

**Objectives:** To define clinical, biochemical, and immune profiles of children with PFAPA and SURF during febrile and afebrile phases, comparing them to control subjects.

**Methods:** Patients diagnosed with PFAPA and SURF were prospectively enrolled and their symptoms systematically recorded. Blood samples were collected at specific time points: during the febrile episode (within 24 hours of fever >38.0°C) and afebrile interval (at least 2 weeks after the last fever). Febrile controls (FC) were otherwise healthy children with acute infections, healthy controls (HC) had non-inflammatory conditions. Sera were analysed for standard parameters (CRP, SAA, procalcitonin (PCT), ferritin), S100A8/9 and S100A12 proteins and a 48-cytokine Bio-Plex panel.

**Results:** Total of 93 periodic fever patients and 35 controls were recruited: PFAPA (n=71), SURF (n=22), HC (n=28), FC (n=7). In 34 patients (15 PFAPA, 19 SURF) paired (febrile/afebrile) samples were available. While the most prevalent symptoms in PFAPA were pharyngitis (96%), adenitis (69%), fatigue (39%), and aphthae (26%), in SURF patients fatigue (55%), abdominalgia (46%), arthralgia (42%) and myalgia (36,%) prevailed. Following symptoms were significantly more frequent in SURF: skin rash and diarrhea (p<0,001), arthralgia (p=0,002), myalgia (p=0,019), abdominalgia (p=0,031). The mean duration of fever episodes was significantly longer in SURF than PFAPA (5.7 (SD 2.8) vs. 3.9 (SD 0.9) days, p= 0,0001). In all paired samples proinflammatory parameters differed significantly. There were no significant differences between febrile PFAPA and SURF patients, or between febrile periodic fever patients and FC. Despite normal CRP, SAA, PCT and S100 protein levels in afebrile samples in both periodic fever groups, serum cytokines were significantly higher in SURF at p<0.01 for IL-1ra, IL-4, IL-17, G-CSF, MIP-1a, IL12p40, IL-18, HGF, M-CSF, and SCF, and at p<0.05 for IL-8, IL-2ra, IL-16, IFN-a2, LIF, and MIG. Afebrile PFAPA and SURF patients had higher levels than HC at p<0.001 in IL-1b, IL-1ra, FGF basic, IL-1ra, IL-18, GROa, LIF, SCF, TNF-b and TRAIL, at p<0,005 in TNF-a, IL-1a, IL-12p40, IFNa2 and SDF-1a, at p<0,05 in G-CSF, IP-10, MIP-1b, IL-16, IFNa2, M-CSF and SCGFb.

**Conclusion:** We have shown significant clinical differences between SURF and PFAPA patients, but their proinflammatory mediators did not differ as they did not differ from FC. Higher FC number is needed to confirm this finding. Multiple serum cytokines differed significantly in afebrile samples between SURF and PFAPA as well as between periodic fevers and HC reflecting potential ongoing immune dysbalance. Changes in cytokines that have not been reported so far will have to be confirmed by single ELISA.

**Acknowledgements**

Supported by the Czech Health Research Council (AZV CR) grant NU21-05-00522

**Patient Consent**

Yes, I received consent

**Disclosure of Interest**

None Declared

**References**

1. Supported by the Czech Health Research Council (AZV CR) grant NU21-05-00522

### Autoinflammatory diseases

#### P023 Malnutrition in children with familial Mediterranean fever

##### Onur Bahceci^1^, Fatma Aydin^1^, Zarife Kuloglu^2^, Salih Isik^3^, Ozen Tas^1^, Betül Oksuz Aydin^1^, Hatice D. Karakas^4^, Zeynep B. Ozcakar^1^

###### ^1^ Pediatric Rheumatology; ^2^Pediatric Gastroenterology; ^3^Department of Child Health and Diseases, Ankara University Faculty of Medicine, Ankara; ^4^Pediatric Rheumatology, Diyarbakir Children Diseases State Hospital, Diyarbakir, Türkiye

####### **Correspondence:** Onur Bahceci

*Pediatric Rheumatology 2024*, **22(2):**PReS24-ABS-1657

**Introduction:** Familial Mediterranean fever (FMF) is a monogenically autosomal recessively inherited autoinflammatory disease characterized by recurrent, self-limiting episodes of fever and sterile serositis. Malnutrition is a set of pathological conditions in which structural and functional disorders occur in tissues and organs due to unbalanced, inadequate or excessive intake of macro and micronutrients necessary for growth and development. Malnutrition may be observed in patients with FMF, but this may be overlooked during routine follow-up. It is essential to identify malnutrition that may accompany these patients to maintain their normal growth and development.

**Objectives:** Our study aims to determine the frequency and types of malnutrition in FMF patients and evaluate malnutrition status after colchicine treatment.

**Methods:** The electronic medical records of the patients who were followed up between 2011 and 2023 with the diagnosis of FMF were retrospectively analyzed. The types of malnutrition of all patients before and after treatment were determined as mild, moderate, severe, overweight, and obesity.

**Results:** A total of 532 patients were included in the study. The median (IQR) age at diagnosis was 6.5 (16.5) years. The median (IQR) follow-up period was 6 (15.5) years. Among 532 patients with FMF, 173 patients (32.5%) had malnutrition at diagnosis. 123 of these patients were underweight; 92 patients had mild (53.2%), 26 patients had moderate (15%) and 5 patients had severe (2.9%) malnutrition. 34 patients were overweight (19.7%) and 16 patients were obese (9.2%). The frequency of malnutrition decreased to 110 patients (20.7%) after colchicine treatment. Especially the frequency of mild, moderate malnutrition and obese patients decreased.

**Conclusion:** Malnutrition is an important issue in children with FMF. According to the results of this study, while approximately one-third of the patients had malnutrition before colchicine treatment, both the frequency and severity of malnutrition decreased after treatment. The presence and type of malnutrition should be considered in the clinical follow-up of FMF patients.

**Date of birth:** 01.06.1989

**Patient Consent**

Not applicable (there are no patient data)

**Disclosure of Interest**

None Declared

**References**

1. Özçakar ZB, Özdel S, Yılmaz S, Kurt-Şükür ED, Ekim M, Yalçınkaya F. Anti-IL-1 treatment in familial Mediterranean fever and related amyloidosis. Clin Rheumatol. 2016, 35:441-446
2. Ozen S, Demirkaya E, Erer B, Livneh A, Ben-Cherit E, et al. EULAR recommendations for the management of familial Mediterranean fever. Ann. Rheum. Dis. 2016, 75:644-651
3. Durmaz, Y., Ilhanli, I., Cengiz, A. K., Kaptanoğlu, E., Hasbek, Z. Ö., & Hizmetli, S. (2020). Association Between Familial Mediterranean Fever and Cachexia in Females. Archives of Rheumatology, 35(4), 477.
4. World Health Organisation: Global Database on Child Growth and Malnutrition, 2017.
5. Grover Z, Ee LC. Protein energy malnutrition. Pediatr Clin North Am 2009; 56: 1055-1068.

### Autoinflammatory diseases

#### P024 How do we diagnose, treat and monitor patients with autoinflammatory diseases mediated by interleukin-1 in central and Eastern Europe?

##### Mario Sestan^1^, Natasa Toplak^2^, Constantin Tamas^3^, Jelena Vojinovic^4^, Zbigniew Zuber^5^, Beata Wolska-Kusnierz^6^, Mihaela Sparchez^7^, Milos Jesenak^8^, Skirmante Rusoniene^9^, Valda Stanevica^10^, Pavla Dolezalova^11^, Liora Harel^12^, Yosef Uziel^13^, Marco Gattorno^14^, Marija Jelusic^15^ on behalf of European Reference Network for Rare Immunodeficiency, Autoinflammatory and Autoimmune Diseases (ERN-RITA)

###### ^1^Department of Paediatrics, University of Zagreb School of Medicine, University Hospital Centre Zagreb, Zagreb, Croatia; ^2^Department of Allergology, Rheumatology and Clinical Immunology, Faculty of Medicine, Ljubljana and University Children’s Hospital, University Medical Center Ljubljana, Ljubljana, Slovenia; ^3^Division of Pediatric Rheumatology and Immunology, Tuzolto street Department, Pediatric Center, Semmelweis University, Budapest, Hungary; ^4^Clinic of Pediatrics, University Clinical Center, Faculty of Medicine, University of Nis, Nis, Serbia; ^5^Department of Pediatrics and Rheumatology, Andrzej Frycz Modrzewski Krakow University, St. Louis Children Hospital, Krakow; ^6^Department of Immunology, Children’s Memorial Health Institute, Warsaw, Poland; ^7^Second Department of Paediatrics, Iuliu Hatieganu University of Medicine and Pharmacy, Cluj-Napoca, Romania; ^8^National Centre for Periodic Fever Syndromes, Department of Pediatrics, Jessenius Faculty of Medicine, Comenius University in Bratislava, University Teaching Hospital in Martin, Martin, Slovakia; ^9^Clinic of Children’s Diseases, Institute of Clinical Medicine, Faculty of Medicine, Vilnius University, Vilnius, Lithuania; ^10^Department of Pediatrics, Riga Stradins University, Riga, Latvia; ^11^Centre for Paediatric Rheumatology and Autoinflammatory Diseases ERN RITA, 1st Faculty of Medicine, Charles University and General University Hospital in Prague, Prague, Czech Republic; ^12^Pediatric Rheumatology Unit, Schneider Children’s Medical Center of Israel, Petach Tikva, Israel; Sackler Faculty of Medicine, Tel Aviv University; ^13^Department of Pediatrics, Pediatric Rheumatology Unit, Meir Medical Center, Kfar Saba, and Tel Aviv University, School of Medicine, Tel Aviv, Israel; ^14^Unit of Rheumatology and Autoinflammatory Diseases, IRCCS Istituto G. Gaslini, Genoa, Italy; ^15^University of Zagreb School of Medicine, University Hospital Centre Zagreb, Zagreb, Croatia

####### **Correspondence:** Mario Sestan

*Pediatric Rheumatology 2024*, **22(2):**PReS24-ABS-1659

**Introduction:** Autoinflammatory diseases mediated by Interleukin-1 (IL-1 AID) constitute the largest category within this group. Notably, significant disparities exist between the recommendations for diagnosis, treatment, and patient follow-up and the actual clinical practices.

**Objectives:** Our aim was to examine the day-to-day clinical practices regarding the diagnosis, treatment, and follow-up of IL-1 AID patients in Central and Eastern Europe and compare them with the 2021 recommendations of the European Alliance of Associations for Rheumatology (EULAR)/American College of Rheumatology (ACR).

**Methods:** In 2023, a collaborative meeting convened representatives from 10 Central and Eastern European countries to deliberate on the current clinical practices related to IL-1 AID: Croatia, Czech Republic, Hungary, Latvia, Lithuania, Poland, Romania, Serbia, Slovakia, and Slovenia.

**Results:** Except for Latvia and Lithuania, specialized centers for diagnosing and treating IL-1 AID with multidisciplinary teams exist in all surveyed countries. Various countries offer massive parallel sequencing panels for autoinflammatory diseases, with turnaround times for results typically ranging from 3 to 6 months. In Slovenia, Hungary, Romania, and Latvia, the waiting period is relatively brief, typically ranging from 1 to 3 months for results from the massive parallel sequencing panel. Public health insurance covers the costs of genetic analyses in most countries. However, Romania stands out as an exception, where patients and foundations largely bear the costs of genetic analysis. Access to disease-specific laboratory assessments, such as S100 proteins, is limited. None of the countries surveyed offer the ability to determine mevalonate kinase enzyme activity or measure IL-1 in serum. Both anakinra and canakinumab are accessible in all countries except Latvia, where canakinumab is unavailable. Notably, the Czech Republic, Croatia, and Slovenia utilize fewer patient-reported outcomes and disease assessment tools in their routine practices compared to other countries. Structured transition programs for IL-1 AID patients are lacking in most countries, although Czech Republic, Slovenia, and Hungary offer pediatricians the option to continue monitoring patients as they transition into adulthood. The starting age of the transition process varies, but in most countries, it generally commences later, usually around 18 years of age or later.

**Conclusion:** Central and Eastern European countries demonstrate potential for adhering to the 2021 EULAR/ACR recommendations for IL-1 AID. However, determining the prevalence and incidence of these diseases in this region remains a persistent challenge for future research.

**Patient Consent**

Not applicable (there are no patient data)

**Disclosure of Interest**

None Declared

### Autoinflammatory diseases

#### P025 Does having additional mutations other than MEFV variants affect FMF phenotype?

##### Ayşenur Doğru^1^, Büşra Başer Taşkın^1^, Bengisu Menentoğlu^1^, Gülşah Kavrul Kayaalp^1^, Özlem Akgün^1^, Ayşe Tanatar^2^, Mustafa Çakan^3^, Nuray Aktay Ayaz^1^

###### ^1^Pediatric Rheumatology, Istanbul University Faculty of Medicine, Istanbul; ^2^Pediatric Rheumatology, Gaziantep City Hospital, Gaziantep; ^3^Pediatric Rheumatology, Zeynep Kamil Women And Children Diseases Traning And Research Hospital, Istanbul, Türkiye

####### **Correspondence:** Ayşenur Doğru

*Pediatric Rheumatology 2024*, **22(2):**PReS24-ABS-1802

**Introduction:** Familial Mediterranean Fever (FMF) is the most common monogenic autoinflammatory disease caused by mutations in the MEFV gene. Fever, serositis, and arthritis are the major clinical findings.

**Objectives:** We aimed to evaluate the impact of concomitant autoinflammatory disease mutations on the phenotype of patients diagnosed with FMF.

**Methods:** The files of 2035 patients diagnosed with FMF according to the Eurofever/PRINTO autoinflammatory recurrent fever classification criteria in three Pediatric Rheumatology centers were retrospectively examined. An autoinflammatory panel was submitted from patients diagnosed with FMF who were unresponsive to colchicine or had additional clinical features such as rash, diarrhea, conjunctivitis, or a family history of other autoinflammatory diseases.

Fifty-eight patients who were sent an autoinflammatory genetic panel were included in the study. Patients were divided into two as only MEFV mutation carriers (group 1) and MEFV and concomitant autoinflammatory disease mutation carriers (group 2). Demographic findings, genetic mutations, clinical features, AIDAI and PRAS scores, and treatments used were recorded and the two groups were compared.

**Results:** Twenty-nine patients (50%) were carrying only MEFV mutation (group 1) and 29 patients (50%) were MEFV and concomitant autoinflammatory disease mutation carriers (group 2).

In group 1, 26 patients (89.6%) had heterozygous MEFV mutations, and 3 patients (10.4%) had compound heterozygous MEFV mutations. In group 2, 22 patients (75.9%) had heterozygous MEFV mutations, 5 patients (17.2%) had compound heterozygous, 2 patients (6.9%) had homozygous MEFV mutations. Patients in group 2 exhibit mutations in genes associated with autoinflammatory diseases apart from MEFV, including NLRP3, NLRP12, MVK, TNFRSF1A, NOD2, IL10-RB, CARD14, LPIN2, ADA2, PSMB8, PSTPIP1 in addition to MEFV mutations.

Demographic characteristics, time between the onset of symptoms and the number of attacks, clinical characteristics, disease activity and severity scores, and the number of patients using biologic drugs were similar between groups.

**Conclusion:** The coexistence of gene variants of other autoinflammatory diseases along with MEFV gene variants implied no significant difference in demographics, clinical findings, outcome measures, and treatments of patients carrying only MEFV gene variants. However, the results need to be validated in larger groups of patients with FMF to clarify the role of concomitant variants in pathology and phenotype of the disease.

**Date of birth::** octobre 20

**Patient Consent**

Not applicable (there are no patient data)

**Disclosure of Interest**

None Declared

**Reference**

1. Gattorno, M., Hofer, M., Federici, S., Vanoni, F., Bovis, F., Aksentijevich, I., ... & Ruperto, N. (2019). Classification criteria for autoinflammatory recurrent fevers. *Annals of the rheumatic diseases*, *78*(8), 1025-1032.

### Autoinflammatory diseases

#### P026 Auto-inflammatory disease associated with constitutional trisomy 8: a French cases series

##### Pauline Bresson^1^, Pierre Quartier dit Maire^2^, Florence Aeschlimann^3^, Isabelle Koné-Paut^4^, Anne-Laure Jurquet^5^, Henri Margot^1^, Fanny Morice-Picard^1^, Cyril Gitiaux^2^, Brigitte Bader-Meunier^2^, Pascal Pillet^1^

###### ^1^CHU Bordeaux, BORDEAUX; ^2^Hopital Necker, Paris, France; ^3^Hôpital pédiatrique Universitaire, Bâle, Switzerland; ^4^Hôpital Bicêtre, Le Kremlin-Bicêtre; ^5^Hôpitaux Nord de Marseille, Marseille, France

####### **Correspondence:** Pauline Bresson

*Pediatric Rheumatology 2024*, **22(2):**PReS24-ABS-1394

**Introduction:** The association between trisomy 8 and Behçet-like autoinflammatory disease has mainly been reported in adult patients with myelodysplastic syndrome associated with trisomy 8. More recently, cases with constitutional trisomy 8 without MDS have been reported to present with systemic autoinflammatory disease with mucocutaneous ulcerations, febrile episodes and abdominal pain.

**Objectives:** We retrospectively report 7 French pediatric cases with recurrent fever, oral aphthosis or bipolar associated with constitutional trisomy 8. We examine their clinical and biological characteristics and present the advances in terms of physiopathology and therapeutic proposal.

**Methods:** A multicenter retrospective chart review of French children with trisomy 8 and autoinflammatory syndrome.

**Results:** We identified 7 children with febrile disease before the age of 5. Five patients of them were female. Four patients had morphological features of trisomy 8 and 3/7 had developmental delay. All patients had oral ulcers; 5/7 had genital or anal ulcers. Cervical adenopathy, arthralgia, abdominal pain and asthenia were also frequently present. Two of 7 patients had with distal axonal motor neuropathy. Six patients had biological inflammation and 3/7 had macrocytosis without associated cytopenia. Treatments showing partial efficacy were: colchicine (6/7), corticosteroids on attack period (6/7), and anti-TNFα as monotherapy (1/7). Total efficacy was noted with prolonged corticosteroid therapy (3/7) but corticosteroid dependence was almost systematic, anakinra (1/7) and the combination of adalimumab and apremilast (1/7).

**Conclusion:** Early-onset recurrent fever associated with recurrent oral or bipolar ulcers calls either for a monogenic cause or for a constitutional trisomy 8. Presence of musculoskeletal abnormalities, facial dysmorphia or acquisition delay requires array CGH analysis.

**Patient Consent**

Yes, I received consent

**Disclosure of Interest**

None Declared

### Autoinflammatory diseases

#### P027 Update on undifferentiated recurrent fevers (SURF) Gaslini’s cohort: consolidating clinical characteristics, evaluating persistence of colchicine efficacy, and exploring the role of Anti-IL-1 treatment

##### Serena Palmeri^1,2^, Chiara Conti^1,2^, Marta Bustaffa^1^, Caterina Matucci-cerinic^1,2^, Diana Sutera^1^, Valentina Natoli^1,2^, Enrico Drago^1,2^, Federica Penco^1^, Ignazia Prigione^1^, Stefano Volpi^1,2^, Riccardo Papa^1^, Roberta Caorsi^1,2^, Marco Gattorno^1^

###### ^1^IRCCS Istituto Giannina Gaslini, Paediatric Rheumatology and Autoinflammatory Diseases Unit; ^2^Università degli Studi di Genova, Dipartimento di Neuroscienze, Riabilitazione, Oftalmologia, Genetica e Scienze Materno-Infantili (DiNOGMI), Genova, Italy

####### **Correspondence:** Serena Palmeri

*Pediatric Rheumatology 2024*, **22(2):**PReS24-ABS-1340

**Introduction:** SURF syndrome, a recent described clinical entity, differs from other monogenic recurrent fevers and PFAPA syndrome. Although clinically similar to familial Mediterranean fever, they lack compatible genetics. Our recent study further delineates biological differences between SURF, FMF and PFAPA.(1,2)

**Objectives:** To describe, updating and supplementing the existing literature data, the follow-up of a homogeneous cohort of 90 patients with SURF, managed at a tertiary referral center.

**Methods:** Patients followed for SURF from 2008 to 2023 were retrospectively included. Exclusions criteria were: confirmed mutations for monogenic recurrent fever (extensive NGS panel) or meeting Eurofever criteria for PFAPA. Treatment response was categorized as:i) complete response (no symptoms and normal acute phase reactants), ii) partial response (symptoms persist with ≥50% reduction in fever episodes), iii) resistance (minimal or no improvement, or worsening).

**Results:** Initially, 92 patients were enrolled, with 2 patients later excluded for meeting PFAPA criteria within 6 months. Of the selected 90 patients (59 males, 31 females), median age at onset was 2.6 years (CI 2.0-3.2), median age at diagnosis 6.5 years (CI 5.2-7.5), and median diagnostic delay 2.6 years (CI 1.8-3.6). Median duration of inflammatory episodes was 4.0 days (CI 3.5-4.0), with a median of 12.0 episodes/year (CI 12.0-15.0). Arthralgia (67%) and abdominal pain (64%) were common symptoms, while only 6% patients presented with oligoarthritis or monoarthritis. Median follow-up was 2.75 years (CI 1.8-3.2, range 0-15.4). Tonsillectomy was ineffective in all 14 patients who underwent the procedure. Colchicine was prescribed in 80/90 patients. Among those with 1-year follow-up data (55/80), it showed 95% efficacy: 39/55 (71%) had complete response, 13/55 (24%) partial, and 3/55 (5%) were ineffective. Two-year follow-up data for 39 patients indicated 97% efficacy: 30/39 (77%) complete response, 8/39 (20%) partial, and 1/39 (3%) resistant. Four patients with complete 1-year response showed partial response by year 2, necessitating anti-IL1 anakinra in 3 cases. Anakinra was initiated in 7 patients: 3 due to colchicine-response decline, 3 for persistent partial response, and 1 for colchicine inefficacy. One patient switched to canakinumab post-allergic reaction to anakinra, reporting efficacy. At last follow-up, 2 patients had never been treated, 49 on colchicine (5 with anakinra, 1 with canakinumab), 13 discontinued colchicine, and 1 received only anakinra. Colchicine withdrawal reasons were: remission (7/13, 54%) and inefficacy (5/13, 38%).

**Conclusion:** This study provides data on an expanded SURF cohort at Gaslini Institute with extended follow-up. Abdominal pain, arthralgia, and minimal pharyngo-tonsillar involvement are confirmed as main clinical features. Colchicine efficacy is affirmed, but gradual loss of efficacy occurs in a small percentage of patients over time. Anti-IL-1 treatment, alone or with colchicine, benefits most non-responsive patients. Further research is needed to fully characterize this patient population, identify possible genetic determinants, and optimize treatment strategies for SURF.

**Patient Consent**

Not applicable (there are no patient data)

**Disclosure of Interest**

S. Palmeri: None Declared, C. Conti: None Declared, M. Bustaffa: None Declared, C. Matucci-cerinic: None Declared, D. Sutera: None Declared, V. Natoli: None Declared, E. Drago: None Declared, F. Penco: None Declared, I. Prigione: None Declared, S. Volpi Speaker Bureau with: Sobi, R. Papa: None Declared, R. Caorsi Speaker Bureau with: Novartis, Sobi, M. Gattorno Speaker Bureau with: Novartis, Sobi, Fresenius Kabi, Kiniksa

**References**

1. Sutera, D. et al., Semin Arthritis Rheum, 2022
2. Palmeri, S. et al. Journal Clin Immun, 2024

### Autoinflammatory diseases

#### P028 Chronic nonbacterial osteomyelitis of mandible in children: descriptive study from a tertiary centre in Bristol

##### Chaitra Govardhan^1^, Ashwini Prithvi^1^, Marion R. Roderick^2^, Ewan Simpson^3^, Athimalaipet V. Ramanan^1^

###### ^1^Paediatric Rheumatology; ^2^Paediatric Immunology; ^3^Paediatric Radiology, Bristol Royal Hospital for Children, Bristol, United Kingdom

####### **Correspondence:** Chaitra Govardhan

*Pediatric Rheumatology 2024*, **22(2):**PReS24-ABS-1430

**Introduction:** Chronic nonbacterial osteomyelitis (CNO) is a focal non-infectious autoinflammatory bone disorder of unknown aetiology in children with mandibular involvement at presentation being a rarity(1).

**Objectives:** To describe the clinical presentation, radiographic appearances and response to treatment in children with mandibular CNO.

**Methods:** The study was conducted at Bristol Royal Hospital for Children. Records of all children less than 18 years diagnosed with CNO of mandible between 2010 to 2023 were retrospectively reviewed. Demographic, clinical, laboratory, radio imaging and response to treatment were recorded.

**Results:** A total of 142 children were diagnosed with CNO. Among them 7 children (4.9%) had mandibular involvement at presentation. All were girls with median age of presentation at 11 years. The presenting symptoms included swelling (n=6; 85.71%), pain (n=2; 28.57%), trismus (n=1; 14.28%) and difficulty in chewing (n=1; 14.28%). 2 children had a family history of psoriasis. Children most often presented to maxillofacial surgeons (n=4) followed by otorhinolaryngologist, orthopaedician and dentist (n=1 each) before being referred to a paediatric rheumatologist. Mean lag time between onset of symptoms and diagnosis was 2.8 years. C-reactive protein and erythrocyte sedimentation rate were within normal range in all children. HLA B27 was positive in 1 (14.28%) child. 6(85.71%) children had diagnostic biopsy. All children underwent whole body magnetic resonance imaging (MRI) at presentation. Monofocal mandibular lesions were present in 5 (71.42%) children. All children received non-steroidal anti-inflammatory drugs (NSAIDs). 1(14.28%) child responded to NSAIDs with complete resolution and required no further treatment. 6(85.71%) children received pamidronate and showed good clinical response with resolution of symptoms on follow up. Following pamidronate, 4 had follow up MRI at the end of 1 year from start of treatment. On repeat MRI, 3 showed radiological response with reduction in signal intensity and 1 showed complete radiological resolution.

**Conclusion:** Although CNO of mandible is rare, awareness of this entity among other specialties is prudent for early referral and initiation of treatment thus resulting in better outcomes. Radiological response may not necessarily correlate with clinical response.

**Trial registration identifying number:** N/A

**Date of birth::** janvier 23

**Patient Consent**

Not applicable (there are no patient data)

**Disclosure of Interest**

None Declared

**Reference**

1. Murray GM, Schnabel A, Alessi M, Chopra M, et al. Is chronic non-infectious osteomyelitis with mandibular involvement a distinct disease? Lancet Rheumatol 2021 Feb 1;3(2):e90–2.

### Autoinflammatory diseases

#### P029 Chronic recurrent multifocal osteomyelitis associated to inflammatory bowel diseases: evidences for a possible association. a multicentric study

##### Caterina Matucci-Cerinic^1,2^, Yagmur Bayindir^3^, Chiara Longo^4^, Francesca Alessi^2^, Francesco Mori^2^, Alessandro Consolaro^1, 2^, Silvia Rosina^2^, Riccardo Papa^2^, Stefano Volpi^1, 2^, Roberta Caorsi^1, 2^, Nicolino Ruperto^5^, Serena Arrigo^4^, Seza Ozen^3^, Marco Gattorno^2^, Clara Malattia^1, 2^

###### ^1^Department of Neurosciences, Rehabilitation, Ophthalmology, Genetics, Maternal andChild Health, University of Genoa; ^2^Rheumatology and Autoinflammatory diseases, IRCCS Istituto Giannina Gaslini, Genoa, Italy; ^3^Department of Pediatric Rheumatology; Hacettepe University, Ankara, Türkiye; ^4^Pediatric Gastroenterology and Endoscopy; ^5^Gaslini Trial Center, IRCCS Istituto Giannina Gaslini, Genoa, Italy

####### **Correspondence:** Caterina Matucci-Cerinic

*Pediatric Rheumatology 2024*, **22(2):**PReS24-ABS-1469

**Introduction:** Chronic non-bacterial osteitis (CNO) is a rare inflammatory disease characterized by multiple sterile bone lesions involving the long bones and the axial skeleton. Association of CNO with Inflammatory Bowel Diseases (IBD), has been reported in some cases. However, still little is known about the topic

**Objectives:** to evaluate the characteristics of CNO patients with IBD (C-IBD) and to compare their clinical, radiologic characteristics and response to treatments in comparison to CNO patients without gastrointestinal (GI) disease

**Methods:** Patients with CNO and C-IBD followed in the Rheumatology Units of the Istituto Gaslini, Genova, and Hacettepe Hospital, Ankara, were retrospectively enrolled in the Eurofever Registry. The clinical, serological and radiological characteristics of the C- IBD (Crohn’s disease(CD),ulcerative colitis(UC),undifferentiated IBD(U-IBD), and of CNO patients were reviewed

**Results:** 17 C-IBD were compared to 131 CNO.In the C-IBD cohort,70% of patients were male, the disease onset was characterized in 88% of cases by CNO, in 11.7% by GI symptoms, and in 11.7% was simultaneous.The median age at CNO onset was 9 years, at IBD onset 11.9 years. In the CNO cohort, 55% were female, median age at disease onset 9.9 years. Long bones were affected in all C-IBD and in 84% of CNO. Spinal involvement was significantly more frequent in CNO (CNO 55% *vs* C-IBD 25%, p=0.01), while sacroiliac involvement was more frequent in C-IBD (CNO 19% *vs* C-IBD 52%, p=0.002). In C-IBD, 59% presented CD,18% UC and 23% IBD-U. 23% were asymptomatic, 64% presented diarrhea,47% abdominal pain,35% weight loss and 23% haematochezia. Also, 41% of patients presented a low-grade fever (present only in 20% of CNO patients).All C-IBD patients had an elevated fecal calprotectine,hemoccult was positive in 78%. Microcytic anemia was reported in 64.7% of C-IBD and in 22% of CNO (p<0.001). At disease onset, increased values of CRP were found in 100% of C-IBD and in only 51% of CNO (p<0.0001). Moreover, in 64.7% of C-IBD,CRP decreased only after IBD treatment was started.In C-IBD, before IBD was diagnosed,70% received NSAIDs,23% steroids,23% methotrexate(MTX) and 35% biologics. After IBD diagnosis, 88% of patients needed an oral steroid course and 82.4% a biological therapy (23.5 % Adalimumab (ADA), 47% ADA+MTX, 6% infliximab).In the CNO group,90% of patients received NSAIDS,39% SAL,36% MTX and 29% bisphosphonates. Biologics were added in 21%(MTX + ADA being the most frequent combination, 13.7%).

**Conclusion:** This represents the largest cohort of IBD associated to CNO reported in the literature. Microcytic anemia, persistently elevated CRP and sacroiliac involvement are key hallmarks of IBD coexistence. We suggest a screening with fecal calprotectin in all CNO patients with a persistent CRP elevation despite therapies. Elucidation of the influence of IBD on CNO severity could provide insights for the development of more effective and targeted therapeutic approaches.

**Patient Consent**

Yes, I received consent

**Disclosure of Interest**

None Declared

### Autoinflammatory diseases

#### P030 Neonatal presentations of A20 haploinsufficiency, a novel familial variant and review of literature

##### Naiera Assalia, Nurit A. Batzir, Gil Amarilyo, Yoel Levinsky, Nufar Marcus

###### Schneider Children’s Medical Center of Israel, Petah Tekva, Israel

####### **Correspondence:** Naiera Assalia

*Pediatric Rheumatology 2024*, **22(2):**PReS24-ABS-1069

**Introduction:** *TNFAIP3* encodes a ubiquitin-editing enzyme wich has a critical role in controlling immune responses. Loss of function variants in *TNFAIP3* causes autosomal dominant Haploinsufficiency of A20. Clinical manifestations are extremely variable and data on neonatal presentations is scarce.

**Objectives:** To further characterize the neonatal spectrum manifestations of A20-haploinsufficincy.

**Methods:** A neonatal case who was admitted at our center is described; data was collected from medical records. Literature was reviewed to identify additional cases of patients with *TNFAIP3*-related symptoms who were presented in the first three months of life.

**Results:** Case presentation

A two weeks old boy with family history of multiple first-degree relatives with recurrent oral ulcers, inflammatory bowel disease (IBD) like symptoms and autoimmunity. Was presented with hypovolemic shock, fever, diarrhea, vomiting, diffuse macular rash, remarkably elevated inflammatory markers, pancytopenia, hyperbilirubinemia, increased INR and hypoalbuminemia. His symptoms persisted despite conservative treatment. When shifted to fully hydrolysed milk protein formula, there was a significant improvement in all of his symptoms. Endoscopic assessment demonstrated multiple colonic ulcerations. A multigene panel identified a novel heterozygous deletion encompassing exons 3-9/9 in *TNFAIP3*.

Review of additional cases in the literature identified 15 cases with neonatal presentations. genetic variants included 4 nonsense, 6 frameshift, one missense and 4 deletions encompassing *TNFAIP3*. The most common and prominent symptom was fever 13 cases (81%) mainly with intermittent pattern. Recurrent ulcers 6 cases (38%), 4 out of them were oral. Enteropathy 6 cases (38%), manifested as diarrhoea, bloody/mucus stool, and colonic ulcers. Hepatosplenomegaly, 4 cases (25%). Skin involvement, 3 cases (19%). 2 cases presented with sepsis like features: Fever, hemodynamic instability and elevated inflammatory markers. one case presented with Hemophagocytic lymphohistiocytosis like features: Pancytopenia, hyperferritinemia, hypertriglyceridemia, hypofibrinogenemia, Elevated liver enzymes, LDH and inflammatory markers.

**Conclusion:** A20 Haploinsufficiency can present early in life, in the neonatal period.

Based on our review, this disease should be considered in the differential diagnosis of neonatal autoinflammatory syndromes, very early onset IBD and neonatal liver involvement, mainly hepatosplenomegaly when it represent with other systemic inflammatory features. Noteworthy to mention, our results identified 3 emergent severe presentations during the neonatal period. No clear genotype-phenotype correlations were noted.

Further studies are required to better characterize the disease in this age group, prognosis and explore treatment strategies.

**We wish to thank the family for participation in the study**

**Patient Consent**

Yes, I received consent

**Disclosure of Interest**

None Declared

**References**

1. Aeschliman et al.PMID 29317407
2. Kim et al.PMID 32743991
3. Kadowaki et al.PMID 29241730
4. Ye et al.PMID 29140941
5. Zheng et al.PMID 29788367
6. Taniguchi et al.PMID 33446651
7. Aslani et al.PMID 36064566
8. Sun et al.PMID 35897075
9. Zhang et al.PMID 38183052
10. Burleigh et al.PMID 38006337
11. Debeljak et al.PMID 37388287
12. Tsuchida et al.PMID 31164164

### Autoinflammatory diseases

#### P031 Long-term follow-up of Blau syndrome in a tertiary hospital

##### Mireia L. Corbeto, Estefanía M. Ruzafa, Lucía R. Diez

###### Pediatric Rheumatology, Vall d’Hebron Barcelona Hospital Campus, Barcelona, Spain

####### **Correspondence:** Mireia L. Corbeto

*Pediatric Rheumatology 2024*, **22(2):**PReS24-ABS-1080

**Introduction:** Blau syndrome (BS) is a rare genetic pediatric arthritis, caused by mutations in the NOD2 receptor. It presents with rash, arthritis, and uveitis, with systemic involvement and risk of blindness and joint deformity. The most common mutations, R334W and R334Q, are present in more than 50% of patients (1). Although some patients have NOD2 mutations without the classic triad, BS is severe and without specific treatment. Recent studies indicate that Tofacitinib may suppress inflammation in BS by inhibiting NOD2 expression, offering a promising option in refractory cases (2). Long-term evolution is unknown, with international and multicenter case series including fewer than 30 patients (3).

**Objectives:** To analyze the long-term evolution and treatment response of patients diagnosed with BS in a tertiary center.

**Methods:** Observational, descriptive, cross-sectional, and single-center study. Patients consecutively starting follow-up at Vall d’Hebron Hospital with a genetic diagnosis compatible with BS are included. The main clinical manifestations, treatments received, and clinical evolution more than 10 years after diagnosis are described.

**Results:** A total of 11 patients were included, 63% female, with a mean age of 29.8 (± 19.38) years, onset age at diagnosis of 13.51 (± 6.87) years, and follow-up time of 13 years. The most frequent mutations detected were R334Q (3 patients, 27.27%) and R587C (3 patients, 27.27%), with the majority being autosomal dominant inheritance (81.81%). Joint involvement was predominant, affecting 6 patients (54.55%) in the form of tenosynovitis. More than half of the patients had cutaneous symptoms, with erythema nodosum being the most frequent (36.36%). 3 patients presented with anterior uveitis at onset, and 2 of them required surgery during follow-up. Other observed symptoms included hearing loss, extensive myelitis, and nonspecific colitis. Methotrexate was the initial treatment of choice, and most patients required escalation to anti-TNF treatment (6 adalimumab, 1 infliximab), with an average of 2 (1-5) biologic treatments. Adalimumab treatment was the most commonly used, with a survival of 9.05 (± 5.34) years. Regarding JAK inhibitors, tofacitinib was used as second-line treatment in 1 patient, and baricitinib in 1 patient after refractoriness to previous biologic therapies for refractory tenosynovitis in both cases.

**Conclusion:** BS presents a variety of clinical manifestations, with tenosynovitis being the most predominant. Although methotrexate is the initial treatment, most patients require biologic drugs, mainly adalimumab, with a mean survival of 9 years. JAK inhibitor treatment shows promise in refractory cases of tenosynovitis. The lack of long-term studies highlights the need for ongoing research to better understand the evolution and treatment of BS.

**Patient Consent**

Yes, I received consent

**Disclosure of Interest**

None Declared

**References**

1. Lassoued Ferjani. Eur J Pediatr. 2023 Sep 2022.
2. Zhang S. Pediatr Rheumatol Online J. Nov 2021.
3. Rosé CD. Rheumatology (Oxford). June 2015.

### Autoinflammatory diseases

#### P032 Gastrointestinal patterns in pediatric Behçet’s disease: insights and associations

##### Joana B. Lima^1^, Francisco R. Mourão^2^, Sérgio Alves^3^, Helena Silva^2^, Rosa Lima^2^, Carla Zilhão^3^

###### ^1^Paediatric Department; ^2^Paediatric Gastroenterology Unit; ^3^Paediatric Rheumatology Unit, Centro Materno Infantil do Norte, Unidade Local de Saúde de Santo António, Porto, Portugal

####### **Correspondence:** Joana B. Lima

*Pediatric Rheumatology 2024*, **22(2):**PReS24-ABS-1601

**Introduction:** Behçet’s Disease (BD) is an autoinflammatory (AI) syndrome characterized by variable vessel vasculitis. Its clinical presentation includes recurrent oral and genital ulcers, involvement of the skin, eyes, joints, gastrointestinal (GI) tract and central nervous system. GI manifestations of childhood BD can be mild and/or heterogeneous, and the diagnosis may be challenging due to the lack of a specific diagnostic test.

**Objectives:** The aim of this study is to characterize children with BD, focusing on GI involvement.

**Methods:** Single center retrospective study of patients with BD diagnosed under 18 years-old and followed between January 2016 and December 2023 in a tertiary center.

**Results:** Thirty-two patients were enrolled, with a mean age at onset for the first manifestation of 8.1±3.8 years and a mean age at diagnosis of 13.6±2.6 years. There was a female predominance (68.8%). Recurrent oral ulcers were the most common manifestation (100%), followed by genital ulcers (81.3%), skin (46.9%), GI (34.4%), articular (28.1%), and ocular (12.5%) involvement. BD family history in 21.9%. Within those with GI symptoms (n=11; 34.4%), 4 (36.3%) were symptomatic at the first evaluation. Adolescents (>=10 years) were more likely to be affected (p=0.038). Mean age of GI symptoms presentation was 13.1±3.4 years. Abdominal pain was the most frequent symptom (28.1%), followed by diarrhea (12.5%), vomiting (6.3%), nausea (6.3%) and rectal bleeding (6,3%). GI patients were also more likely to have previous history of PFAPA-like syndrome (p=0.003). From the patients undergoing endoscopic work-up (n=8, 25.0%), 6 (18.8%) revealed pathological findings. Calprotectin values were higher in patients with GI symptoms (476.5±655.7 vs 30.20±28.3, p=0.045). However, when comparing levels between patients with and without pathological findings in endoscopic work-up, no statistical differences were found (584.2±738.2 vs 698.0±973.0, p=0.864). HLA typing revealed B51 in 3 (30.0%), B44 in 3 (30.0%), B57 in 2 (18.2%), and B7/B15 in 1 (10.0%). No statistical difference was found in HLA typing between patients with and without GI involvement. Patients were treated with azathioprine (54.5%), colchicine (54.5%), adalimumab (27,3%) and corticosteroids during flares (37.3%).

**Conclusion:** This study highlights the heterogeneity of pediatric BD focusing on GI tract. Adolescents exhibited higher likelihood of presenting GI symptoms upon admission and the overall higher prevalence of GI complaints is in accordance with other published data. Considering these issues and the low sensitivity of pediatric classification criteria, we suggest the inclusion of GI manifestations to increase their applicability on a clinical basis.

**Date of birth::** 9/13/1995 1

**Patient Consent**

Not applicable (there are no patient data)

**Disclosure of Interest**

None Declared

### Autoinflammatory diseases

#### P033 Different phenotypes and cytokine profiles in three family members with papa syndrome

##### Nico Wittmann^1^, Dominik Nell^2^, Neha Mishra^2^, Magdalena Mohs^2^, Marie C. Grude^2^, Lukas Bossaller^2^, Almut Meyer-Bahlburg^1^

###### ^1^Ped. Rheumatology and Immunology, Deparment of Pediatrics; ^2^Section of Rheumatology, Department of Medicine A , University Medicine Greifswald, Greifswald, Germany

####### **Correspondence:** Almut Meyer-Bahlburg

*Pediatric Rheumatology 2024*, **22(2):**PReS24-ABS-1628

**Introduction:** PAPA syndrome, an acronym for pyogenic sterile arthritis, pyoderma gangraenosum and acne, is a rare autoinflammatory disease caused by an autosomal dominant mutation in the PSTPIP1 gene. Here, we present a family with PAPA syndrome with three affected members (mother and her two sons) with different clinical phenotypes and cytokine profiles.

**Objectives:** We sought to identify correlations between clinical manifestation, therapy and cytokine profile.

**Methods:** After signed informed consent, blood samples were taken from the patients at different times for cytokine determination using a multiplex bead array. In addition, ASC speck formation was analysed by flow cytometry. Inflammatory parameters were determined as part of routine diagnostics. Blood samples from healthy donors were used as controls. The results were analysed for correlations with clinical data and inflammatory parameters.

**Results:** The clinical spectrum of manifestations of PAPA syndrome in this family included pyogenic arthritis, acne, pyoderma gangraenosum, ulcerative colitis, Crohn’s disease and autoimmune hepatitis. ASC specks as a sign of inflammasome activation were not detected in any of the samples. Most impressively, IL-18 levels were extremely upregulated, independent of disease activity and therapy, and correlated with IFN-α levels, which were also elevated. IL-1β was also upregulated, but did not correlate with IL-18 levels or inflammatory parameters. Other cytokines that were upregulated compared to healthy donors included IL-6, IL-8, IL-10 and IL-33. However, no correlation was found between cytokines, therapy and disease activity.

**Conclusion:** Several cytokines have been found to be upregulated in patients with PAPA syndrome, most importantly IL-18; however, there is no significant correlation with disease activity, therapy or clinical manifestations.

**Patient Consent**

Yes, I received consent

**Disclosure of Interest**

None Declared

### Autoinflammatory diseases

#### P034 Feasibility and acceptance of kidscreen-52: a comprehensive screening tool for unmet needs in children with inflammatory diseases

##### Özlem Satirer^1^, Gabi Erbis^1^, Verena Heck^1^, Tatjana Welzel^2^, Christiane Reiser^1^, Anne-Kathrin Gellner^3^, Susanne M. Benseler^4,5^, Jasmin Kümmerle-Deschner^1^

###### ^1^Division of Paediatric Rheumatology, Department of Paediatrics and Autoinflammation reference Center Tuebingen (arcT), Tübingen University, Tübingen, Germany; ^2^Pediatric Rheumatology, University Children’s Hospital Basel (UKBB), Basel, Switzerland; ^3^Department of Psychiatry and Psychotherapy , Bonn University Hospital, Bonn, Germany; ^4^Rheumatology, Department of Paediatrics, Alberta Children’s Hospital (ACH), ACH Research Institute, University of Calgary, Calgary, Canada; ^5^Children’s Health Ireland, Dublin, Ireland

####### **Correspondence:** Özlem Satirer

*Pediatric Rheumatology 2024*, **22(2):**PReS24-ABS-1208

**Introduction:** Children with rare and chronic diseases often experience multiple psychosocial challenges. Identifying the unmet needs of these children is crucial to deploy targeted support and allocate resources. Comprehensive screening tools are urgently needed

**Objectives:** To evaluate the feasibility and acceptance of the comprehensive KIDSCREEN-52 tool for identifying the unmet needs in children diagnosed with inflammatory diseases and their families

**Methods:** A prospective, single-center study of consecutive pediatric patients ages 8-18 years with inflammatory diseases and their caregivers was performed. The comprehensive KIDSCREEN-52 tool, available in 13 languages, was utilized; 52 items assessed the dimensions of health-related quality of life (HRQoL). Analysis: 1) HRQoL – data obtained from KIDSCREEN self-report and caregiver proxy were equated with appropriate normative data sets and compared withing families 2) feasibility and acceptance – completion rate, time to completion and, self-developed acceptance questionnaire 3) comparison of self-reported psychosocial burden of autoinflammatory versus rheumatic diseases.

**Results:** A total of 106 participants were included, 53 pediatric patients and their 53 caregivers. The patients were 35 females and 18 males with a median age of 16 years (9-18). Inflammatory diagnoses: 25 (49%) with autoinflammatory and 26 (51%) with rheumatic diseases. 1) Mean values obtained from proxy and self-reports were consistent with the German normative dataset across all domains. Comparison self-report/proxy: Differences were observed in the domains “bullying” (mean self-reports: 52.5 vs proxy: 52.7) and “financial resources” (mean self-reports: 57.8 vs proxy: 59.4). Similarities were seen for the other eight domains within families. 2) Feasibility and acceptance: Overall completion rate was 75%; the mean completion time was 17min (10-25). Both parents and children expressed substantial acceptance regarding the clarity, relevance, and adequacy of the questionnaire. 3) Compared to autoinflammation the psychosocial burden was higher in patients with rheumatic diseases most significantly in the domains of Parent Relations and Home Life, School Environment and Self-Perception.

**Conclusion:** The KIDSCREEN-52 tool shows promise as a feasible and accepted screening tool for identifying the unmet needs in children with inflammatory diseases. The short and comprehensive tool available in multiple languages provides a unique opportunity to deploy strategies for improving the HRQoL in routine care.

**Patient Consent**

Yes, I received consent

**Disclosure of Interest**

None Declared

### Autoinflammatory diseases

#### P035 Autoinflammatory diseases in Oman: insights and perspectives from Oman

##### Hamna Al Musalhi^1^, Ruqaiya Al Jashmi^2^, Safiya Al Abrawi^2^

###### ^1^Pediatric Residency program, Oman Medical Specialty Board; ^2^Child Health, Royal hospital, Muscat, Oman

####### **Correspondence:** Hamna Al Musalhi

*Pediatric Rheumatology 2024*, **22(2):**PReS24-ABS-1250

**Introduction:** Autoinflammatory diseases (AIDs) are a group of disorders characterized by recurrent episodes of inflammation without the involvement of autoantibodies or autoreactive T cells, which are typically seen in autoimmune conditions(1,2). These diseases often result from intrinsic dysregulation of the innate immune system, leading to periodic or continuous inflammation that can manifest in a variety of symptoms(3). Published data from Oman regarding autoinflammatory diseases are limited thus studying their prevalence, clinical presentations, and treatment responses in Oman provides crucial insights into regional and global healthcare strategies.

**Objectives:** The main aim of this study is to offer a comprehensive overview of autoinflammatory diseases (AIDs) in Oman, encompassing their clinical, genetic, and therapeutic dimensions. Additionally, it seeks to investigate the disease’s presentation, onset age, diagnostic methods, and treatment options. Furthermore, the research endeavors to delineate the clinical profile of systemic amyloidosis patients. Secondary objectives include assessing treatment efficacy, response, and impact on growth parameters, aiming to provide a holistic understanding of this disease.

**Methods:** This is a retrospective cross-sectional study that included all patients with genetically proven AIDS who visited Pediatric Rheumatology or Genetic clinic in The Royal Hospital in Oman from January 2013 to July 2023. A hospital’s electronic records were used to collect data on patients with ICD 10 CODES: periodic fever syndrome and recurrent fever.

**Results:** A total of 47 patients, comprising 53% females, were included in the study. The mean age at onset was 3.3 years, while the mean age at diagnosis was 8.6 years; 63% of patients had consanguinity. The diagnoses were distributed as follow: FMF (27.7%), TRAPS (25.5%), CNO (14.9%), AGS (12.8%), PFAPA (8.5%), MKD (6.4%), DIRA, and PAMI syndrome (2.1% each). Genetic analysis was performed in 70% (33) of patients, revealing that 8 out of 12 TRAPS patients had the genetic mutation C375T>G/pCYs125TRP. Common presenting symptoms included fever (66.0%), abdominal pain (57.4%), and arthralgia (53.2%). Corticosteroids were used in 54% of patients, while 15% received biologic agents. Growth failure was reported in 14 (30%) of the patients. This study highlights a diverse range of autoinflammatory diseases, with a significant proportion being genetically confirmed monogenic disorders.

**Conclusion:** This study provides valuable insights into the epidemiology, diagnosis, and management of autoinflammatory diseases, reaffirming the need for a multidisciplinary approach involving genetic testing, early recognition of symptoms, and tailored treatment strategies to improve patient outcomes.

**Patient Consent**

Yes, I received consent

**Disclosure of Interest**

None Declared

**References**

1. Ashari KA, Hausmann JS, Dedeoglu F. Update on autoinflammatory diseases. Curr Opin Rheumatol. 2023 Jul 6;35(5):285-92
2. Pathak S, McDermott MF, Savic S. Autoinflammatory diseases: Update on classification diagnosis and management. J Clin Pathol. 2016 Sep 19;70(1):1-8.
3. Gutierrez MJ, Lapidus SK. Systemic Autoinflammatory Diseases: A Growing Family of Disorders of Overlapping Immune Dysfunction. Rheum Dis Clin North Am. 2022 Feb;48(1):371-395.

### Autoinflammatory diseases

#### P036 Exploring the impact of bisphosphonates in pediatric CRMO: insights from a French study with 30 patients over 3 years

##### Sarah Remtoula^1^, Justin Ulm^2^, Marie Robert^3^, Perrine Dusser^1,4^

###### ^1^94275, Department of Pediatric Rheumatology, National Reference Centre for Auto-inflammatory Diseases and Amyloidosis of Inflammatory origin (CEREMAIA), Bicêtre hospital, Assistance Publique-Hôpitaux de Paris (AP-HP), Le Kremlin Bicêtre; ^2^92140, Percy military teaching hospital, Clamart; ^3^94275, Department of Rheumatology, Hôpital Bicêtre, Assistance Publique – Hôpitaux de Paris, Université Paris-Saclay, Le Kremlin-Bicêtre, France, Le Kremlin Bicêtre; ^4^94807, Université Paris-Saclay, UVSQ, Inserm, CESP, Villejuif, France

####### **Correspondence:** Perrine Dusser

*Pediatric Rheumatology 2024*, **22(2):**PReS24-ABS-1574

**Introduction:** Chronic recurrent multifocal osteomyelitis (CRMO) presents challenges in treatment, with NSAIDs being the primary choice. However, in cases of NSAID resistance, bisphosphonates (BPs) and anti-TNF drugs are often utilized, despite lacking marketing authorization.

**Objectives:** The primary objective was to identify factors influencing the response to BPs in pediatric CRMO patients. We assessed clinical, biological, and radiological parameters at 12 months and the final visit. Secondary objectives included examining the tolerability of BPs and the impact of treatment regimen on outcomes.

**Methods:** Monocentric retrospective study of <16-year-old CRMO patients at CHU Kremlin Bicêtre (2010-2022) treated with BPs either due to resistance or intolerance to NSAIDs or because of spinal involvement. Epidemiological and radiological data were collected. Linear and logistic regression models analyzed factors associated with clinical, biological, and radiological outcomes.

**Results:** In this study, 30 patients (SR H/F=0.6) treated with BPs were included followed for an average of 49.9±26.5 months (range 18-110). Among them, 7 patients received BPs intermittently, while 23 patients underwent systematic treatment with 4 courses administered over 1 year. 22.3% received anti-TNFs alongside BPs.

On average, patients received 2.9 BP courses at 12 months and 4.8 at follow-up. Notably, 70.4% required additional treatment beyond 12 months due to vertebral collapse or relapse. Regarding remission rates, 33.3% achieved clinical, radiological, and biological remission at follow-up compared to 14.8% at 12 months (p = 0.111). Significant improvements were observed at 12 months, including decreased pain intensity (p <0.001), reduction in symptomatic lesion count from 2.17 to 1.47 (p = 0.164), and decreased inflammatory syndrome (p = 0.286). Further improvements were noted at the last follow-up (p <0.001). Limb involvement was correlated with clinical improvement (p = 0.001 for upper limbs, p = 0.050 for lower limbs). Hypocalcemia occurred in 20 patients (66.7%), managed with oral calcium and vitamin D. Additionally, 18 patients (60.0%) experienced flu-like symptoms after the first BP course.

**Conclusion:** BPs effectively reduced pain intensity, symptomatic lesions, and inflammatory syndrome. Systematic 1-year treatment showed better outcomes than intermittent therapy. However, impact on radiological progression remains uncertain. Prospective placebo-controlled studies are needed to address this and assess long-term risk-benefit ratio beyond 1 year.

**Patient Consent**

Not applicable (there are no patient data)

**Disclosure of Interest**

None Declared

### Autoinflammatory diseases

#### P037 Comparing the efficacy of various treatment regimes on termination of the attacks in familial Mediterranean fever

##### Elif Kilic Konte, Nergis Akay, Umit Gul, Kardelen Karaahmetli, Gokce N. Cengiz, Sevval Kaplan Kilic, Esra Ozturk, Aybuke Gunalp, Esma Aslan, Fatih Haslak, Mehmet Yildiz, Amra Adrovic, Sezgin Sahin, Kenan Barut, Ozgur Kasapcopur

###### Department of Child Health and Disease, Division of Pediatric Rheumatology, Istanbul University-Cerrahpasa, Cerrahpasa Faculty of Medicine, Istanbul, Türkiye, Türkiye

####### **Correspondence:** Elif Kilic Konte

*Pediatric Rheumatology 2024*, **22(2):**PReS24-ABS-1641

**Introduction:** Familial Mediterranean fever (FMF) is the most common inherited autoinflammatory disease. Although attacks of FMF are usually self-limiting, there is a strong need for therapies in order to shorten the duration of the 1-3 days lasting attacks, thereby decreasing the need for hospitalization.

**Objectives:** This study aimed to compare the effectiveness of 3 treatment modalities, all of which are widely used for FMF attacks.

**Methods:** The study included FMF patients with only compound heterozygous or homozygous mutations in exon 10. In the routine clinical setting, for patients admitted to the ER with an FMF episode, one of the 3 different treatment modalities is preferred as a first-line therapy according to the decision of the consultant pediatric rheumatologist: Anakinra, NSAID, and intravenous 0.9% saline. Demographic data, laboratory parameters, attack symptoms and pain VAS scores were prospectively recorded after administration of the respective treatment. Pain VAS scores were monitored by phone call every two hours, and CRP levels were measured at the time of treatment and at 24^th^ hour of attack therapy. The data was analyzed using the Mann-Whitney U-test for non-normally distributed VAS scores and the paired t-test for normally distributed CRP values.

**Results:** Of 56 patients, 23 (50%) were female. The median ages for symptom onset, diagnosis, and last visit were 14.8(9.8-18.5), 3(1.5-6), and 4(2.5-7) years, respectively. The yearly median number of attack was 3.5 (2-10). More than half (53.5%) had a history of irregular colchicine use. Common symptoms during the attack included abdominal pain (85.7%), chest pain (57.1%), fever (53.5%), vomiting (32.1%), and diarrhea (21.4%). Lymphopenia was present in 26 (46%) patients and leucocytosis in 12 (21.4%) patients. The median duration of the attack was 24 (6-48) hours after initiation of treatment. The mean (SD) CRP values at 0 and 24 hours were 118 (78) and 100 (52) mg/L, respectively (p=0.21). Patients, whose FMF episode have been treated with anakinra, had statistically lower VAS values at 6,12 and 24 hours (p<0.05).

**Conclusion:** Anakinra treatment provided significant improvement in pain VAS scores comparing to other 2 nationally used FMF attack therapies. As a conclusion, single-dose anakinra treatment could decrease the requirement of hospitalization in FMF attack patients.

**Date of birth::** octobre 12

**Patient Consent**

Yes, I received consent

**Disclosure of Interest**

None Declared

**References**

1. Babaoglu H, Varan O, Kucuk H, Atas N, Satis H, Salman R, Ozturk MA, Haznedaroglu S, Goker B, Tufan A (2019) On demand use of anakinra for attacks of familial Mediterranean fever (FMF). Clin Rheumatol 38(2):577–581.
2. Cebeci SO, Yildiz M, Gunalp A, Cebi MN, Kilinc B, Pinar E, Konte EK, Aslan E, Haslak F, Adrovic A, Sahin S, Barut K, Kasapcopur O. The efficacy of a single-dose anakinra injection during disease attack in pediatric familial Mediterranean fever. Rheumatol Int. 2023 Jun 6. 10.1007/s00296-023-05351-0.
3. Yıldız M, Haşlak F, Adrovic A, Barut K, Kasapçopur Ö (2020) Autoinflammatory diseases in childhood. Balk Med J 37(5):236–246.
4. Gül A, Ozdogan H, Erer B, Ugurlu S, Kasapcopur O, Davis N, Sevgi S (2015) Efficacy and safety of canakinumab in adolescents and adults with colchicine-resistant familial Mediterranean fever. Arthr Res Ther 17(1):243.

### Autoinflammatory diseases

#### P038 Successful treatment of TREX-1 monogenic lupus with anifrolumab

##### Patricia Morán Álvarez^1^, Francesca Soscia^2^, Virginia Messia^1^, Valentina Matteo^1^, Marco Francesco Natale^1^, Fabrizio De Benedetti^1^, Antonella Insalaco^1^

###### ^1^Ospedale Pediatrico Bambino Gesù, Rome; ^2^Ospedale Santa Maria della Stella, Orvieto, Italy

####### **Correspondence:** Patricia Morán Álvarez

*Pediatric Rheumatology 2024*, **22(2):**PReS24-ABS-1372

**Introduction:** systemic lupus erythematosus (SLE) is a chronic, life-threatening, multi-systemic autoimmune disease characterized by the presence of antinuclear antibodies. Classically, SLE was defined as a polygenic disease. However, during the last decades several novel variants have been identified, which are thought to act strongly in SLE pathogenesis. Thus, a challenging insight into monogenic lupus is emerging. TREX1 is a widely expressed homo-dimeric protein which plays an important role in single-stranded DNA degradation. Loss of function genetic defects in TREX1 lead to an inappropriate activation of type I interferon. TREX1 mutations have been shown to cause Aicardi-Goutières syndrome but also monogenic SLE.

**Objectives:** to report the case of a child with TREX1- related monogenic lupus carrying a de novo mutation (c.598G>C p.Asp200His), successfully treated with anifrolumab.

**Methods:** a 16-year-old female presented since the age of 7 with chilblain on the fingers. At the age of 13 she presented also with chilblain on the toes, arthralgias, proteinuria (500 mg/24h) and lymphopenia. A kidney’s biopsy showed a picture compatible with class I lupus nephritis. The diagnosis of SLE was made. In the following years, despite multiple treatments (high-dose glucocorticoids, mycophenolate and hydroxychloroquine), the clinical picture persisted with the exception of proteinuria and she started to present hypocomplementemia (C3 0.84, nv 0,9-1,8 g/L). Therefore, based on genetic and biomarker findings (Interferon Score of 83,5), we decided to start a treatment with anifrolumab, a human immunoglobulin G1 kappa monoclonal antibody that binds to subunit 1 of the type I interferon receptor.

**Results:** infusions of intravenous anifrolumab, at a dose of 300 mg every 4 weeks were started. After 3 months of treatment, a significant clinical and biochemical improvement was observed. After one single infusion, interferon score has become negative (1, nv < 2). At 3 month-follow up, she presented with a complete resolution of the skin involvement, of the hypocomplementemia (C3 levels of 1 g/L from C3 0.84 g/L at baseline) and of the lymphopenia (1.390/mm3 from 900/mm3 at T0).

**Conclusion:** we report the first case of monogenic SLE treated successfully with anifrolumab. Our findings suggest that anifrolumab should be considered as a treatment option of type-I interferonopathies. More studies to assess long-term treatment efficacy of anifrolumab are needed in order to determine the impact of IFN-I blockade in autoinflammatory interferonopathies.

**Date of birth:** octobre 15

**Patient Consent**

Yes, I received consent

**Disclosure of Interest**

None Declared

**References**

1. Alperin JM, Ortiz-Fernández L, Sawalha AH. Monogenic Lupus: A Developing Paradigm of Disease. Front Immunol. 2018
2. Rice GI, Rodero MP, Crow YJ. Human disease phenotypes associated with mutations in TREX1. J Clin Immunol. 2015

### Autoinflammatory diseases

#### P039 Clinical presentation and course of pulmonary involvement in chronic nonbacterial osteomyelitis

##### Toni Hospach^1^, Friederike Blankenburg^1^, Christiane Reiser^2^, Jens Klotsche^3^, Christian Hedrich^4^, Thekla von Kalle^5^

###### ^1^Pediatric Rheumatology, Klinikum Stuttgart, Stuttgart, Germany; ^2^Pediatric Hospital, Bregenz, Austria; ^3^German research Center Rheumatology, Berlin, Germany; ^4^University, Liverpool, United Kingdom; ^5^Pediatric Radiology, Klinikum Stuttgart, Stuttgart, Germany

####### **Correspondence:** Toni Hospach

*Pediatric Rheumatology 2024*, **22(2):**PReS24-ABS-1048

**Introduction:** Pulmonary involvement in chronic nonbacterial osteomyelitis (CNO) is rare and limited awareness and experience, even among CNO experts, may result in diagnostic challenges, especially as malignancy or infection need to be considered.

**Objectives:** This study systematically investigated clinical and imaging presentations, demographic features, treatment response and outcomes of pulmonary involvement in CNO (pCNO).

**Methods:** A survey was shared among centers participating in the *Kerndokumentation Deutsches Rheumaforschungszentrum* (Germany) cohort to identify patients with pCNO. Clinicodemographic data were collected from hospital records, and features were compared between pCNO patients and the remaining cohort. MRI and CT images were read and interpreted centrally by an experienced pediatric radiologist

**Results:** A total of 22 patients with pCNO were included in this study. Pulmonary involvement was more common in girls (91% versus 62.8%, p=0.006) and patients with multifocal bone lesions (95% versus 65%, p<0.001), but did not associate with altered systemic inflammatory parameters (CRP) or other organ involvement. Altogether, 42 lesions were counted with a median of 2 per patient (2-6). Pulmonary lesions had a median size of 1.8 cm (0.3-4.0 cm), followed mono- (40%) and oligo-focal (60%) patterns, representing consolidations or nodules of moderate size, abutting the pleura in half the patients. Most lesions affected the lower lobes. While prominent hilar lymph nodes were present (in 19%), no pathological enlargement (>1cm) was seen. Where available (3/22), histology revealed granulomatous inflammation with lymphocyte infiltration. Development and courses of pCNO did not associate with absence or presence of treatment. Complete remission of pCNO was reported in 60% patients, partial remission in 20%, and remitting or unchanged disease was seen in 20%. Pulmonary lesions reoccurred after initial resolution in three patients (15%), in all cases affecting previously not affected regions.

**Conclusion:** In conclusion, pCNO is usually asymptomatic. While more common in girls and patients with multifocal CNO, pCNO is not associated with systemic inflammatory parameters or specific organ involvement. Prognosis of pCNO is favorable, and most lesions resolve over time. Thus, a careful watch-and-wait strategy may be appropriate

**Patient Consent**

Yes, I received consent

**Disclosure of Interest**

None Declared

### Autoinflammatory diseases

#### P040 Interleukin 1 receptor antagonist as biomarker for disease flares in fibrodysplasia ossificans progressiva

##### Riccardo Papa, Arinna Bertoni, Caterina Matucci-Cerinic, Enrico Drago, Francesca Liberatore, Anna Corcione, Marco Gattorno

###### UOC Reumatologia e Malattie Autoinfiammatorie, IRCCS Istituto Giannina Gaslini, Genova, Italy

####### **Correspondence:** Riccardo Papa

*Pediatric Rheumatology 2024*, **22(2):**PReS24-ABS-1073

**Introduction:** Fibrodyplasia ossificans progressiva (FOP) is an ultra-rare, autosomal dominant disorder caused by heterozygous gain-of-function *ACVR1* mutations. Beginning in childhood, FOP patients suffer of massive heterotopic ossifications with a proximal to distal pattern, some of which takes form as inflammatory flare-ups, which partially respond to anti-inflammatory agents. Currently, no laboratory markers are available to support clinical suspicion of flare-ups.

**Objectives:** To discern a potential cytokine hierarchy during a typical flare, we examined four FOP patients in the midst of an active disease flare.

**Methods:** We utilized a multi-cytokine enzyme-linked immunosorbent assay kit to measure the levels of interleukin (IL)-1 receptor antagonist (IL-1RA), IL-6, IL-18, and p55 tumor necrosis factor receptor 1. The obtained data were then compared with results from five inactive FOP patients and a control group of healthy individuals.

**Results:** IL-1RA exhibited a significant increase during disease flares in classic and non-classic FOP patients, whereas the levels of the other cytokines showed no substantial differences when compared to both inactive FOP patients and healthy controls. As expected, routine laboratory tests, including C reactive protein and the erythrocyte sedimentation rate, were always normal, even during disease flare. In all cases, heterotopic ossification secondary to disease flare was then confirmed by CT scan.

**Conclusion:** These preliminary findings suggest that IL-1RA could serve as a potential biomarker for disease flares in FOP, providing additional support for the potential involvement of IL-1 in the pathogenesis of heterotopic ossifications in this condition.

**Patient Consent**

Not applicable (there are no patient data)

**Disclosure of Interest**

None Declared

### Autoinflammatory diseases

#### P041 Chronic non-bacterial osteomyelitis and associated diseases in childhood period

##### Betül Öksüz Aydın, Fatma Aydın, Özen Taş, Onur Bahçeci, Zeynep Birsin Özçakar

###### Pediatric Rheumatology, Ankara University Faculty of Medicine, Ankara, Türkiye

####### **Correspondence:** Betül Öksüz Aydın

*Pediatric Rheumatology 2024*, **22(2):**PReS24-ABS-1247

**Introduction:** Chronic non-bacterial osteomyelitis (CNO) is a rare autoinflammatory bone disease and may occur alone or in association with other inflammatory diseases such as psoriasis, palmoplantar pustulosis and familial Mediterranean fever (FMF). Whole-body magnetic resonance imaging (MRI) is considered the gold standard for diagnosis.

**Objectives:** The aim of this study was to present clinical and follow up results of patients with CNO and to compare these features in patients with and without associates diseases.

**Methods:** Patients diagnosed with CNO with a minimum follow-up period of 6 months between 2018 and 2023 were included in the study. Demographic characteristics, clinical course, laboratory and imaging findings, treatments and comorbidities were recorded. The patients were divided into two groups according to the presence of additional diseases and their characteristics were compared.

**Results:** The study included 37 patients (n=25, 67.6% male) diagnosed with CNO. The median (IQR) age at symptom onset was 10.4 (7.2) years and at diagnosis was 11.7 (5.25) years. The median (IQR) follow-up period was 39 (44.7) months. The most common presenting complaints were joint pain (n=31, 83.8%) and bone pain (n=28, 75.7%). The most common physical examination finding was pain on bone palpation (n=10, 27%). Multifocal involvement was present in 36 (97.3%) patients. Four patients (10.8%) had arthritis.

All patients received NSAIDs, 16 (43.2%) methotrexate, 3 (8.1%) salazoprine, 10 (27%) prednisolone, 20 (54%) biologic agents (anti TNF, anti IL-1) and 4 (10.8%) bisphosphonate. Thirty three (89.2%) of the patients were clinically in remission. While 29 patients (78.4%) were in remission with treatment, 4 patients (10.8%) were in remission without treatment. Ten (27%) patients developed relapse after a median (IQR) of 3.8 (2) months after treatment discontinuation. Thirteen (35.1%) of thirty seven patients had radiologically new lesions detected on control MRI. Twenty three patients (62.2%) had regressed existing lesions on control MRI and were considered to be in radiologic remission. During follow up, vertebral height loss developed in 3 patients (8.1%), osteopenia in 3 patients (8.1%), and sclerosis in 2 (5.4%).

Thirteen (35%) patients had comorbidities. The comorbidities were FMF (n=8), inflammatory bowel disease (IBD) (n=3), uveitis (n=3), psoriasis (n=2) and acne conglabatae (n=2).

It was found that 92.3% of patients with comorbidities were male (p=0.027). Bone pain was more common in the group without concomitant disease than in the group with concomitant disease (p=0.042). The mean±SD number of bone involvement was 3±1.39. The group with comorbidities had the higher number of involved bones (3 or more bones) (n=11, 84.6%) (p=0,023). 11 (84.6%) of 17 patients who received biologic treatment had a comorbid disease and this result was statistically significant (p=0.001).

**Conclusion:** Chronic non-bacterial osteomyelitis is an autoinflammatory disease, which may coexist with other inflammatory conditions like FMF, IBD, psoriasis and uveitis in approximately one-third of the patients. Patients with associated diseases had greater number of bones affected and required more biological agents. Suggesting that CNO may be more severe in the presence of comorbidities.

**Date of birth::** 04.12.1992

**Patient Consent**

Not applicable (there are no patient data)

**Disclosure of Interest**

None Declared

### Autoinflammatory diseases

#### P042 A family with novel variation in TNFAIP3 gene causes A20 haploinsuffiency, a description and highlighting a distinct features

##### Naiera Assalia, Nurit A. Batzir, Gil Amarilyo, Yoel Levinsky, Nufar Marcus

###### Schneider Children’s Medical Center of Israel, Petah Tekva, Israel

####### **Correspondence:** Naiera Assalia

*Pediatric Rheumatology 2024*, **22(2):**PReS24-ABS-1331

**Introduction:** A20 Haploinsufficiency was first described in 2016. It is caused by Loss-of-function mutations in *TNFAIP3* that result in activation of the nuclear factor (NF)-kB pathway. Symptoms driven by primary dysfunction of innate immune system leading to autoimmunity, autoinflammation and immune dysregulation.

**Objectives:** To describe the clinical phenotype and disease course of a novel *TNFAIP3* germline variant in multiple first degree family members.

**Methods:** The medical records of a family (parents and their 7 offsprings), who have attended our institution were reviewed. The mutation was detected in the proband patient, due to suspected inborn error of immunity during the neonatal period. Genetic analysis for the proband patient, his brother and father were done by next generation sequencing and for the rest of the family members tests were done by Multiplex Ligation dependent Probe Amplification.

**Results:** A deletion encompassing exons 3-9 in *TNFAIP3* gene was identified in the proband patient, a neonate who presented with prolonged fever, enteropathy, and rash. Subsequently, genetic analysis for all the family members identified 6 additional patients (father, 2 sisters and 3 brothers). 2/7 had disease onset during the first 3 months of life and the others at early childhood (4-7 years). 6/7 had recurrent oral ulcers. 4/7 had significant recurrent flares of abdominal pain and diarrhea, two of them had normal endoscopy findings and another 2 had pathologic colonoscopy with eosinophilia on histology. One (the father) had psoriasis. 3/6 had recurrent episodes of rhinorrhoea, allergic conjunctivitis like symptoms, oral ulcers, and fevers. ANA was positive for 6/7. All of them had increased inflammatory markers (ESR and CRP) during attacks, while 2/7 had persistent elevated inflammatory markers. 1/7 had multiple autoimmune disorders including: lupus-like phenotype; lupus nephritis class 5 and low complement levels C3 and C4, autoimmune hepatitis type 2, autoimmune gastritis, and thyroid autoantibodies. Interestingly, all family members carrying the variant had persistent moderate eosinophilia and 2 of them had milk protein allergy. All of them their gastrointestinal symptoms, oral ulcers and inflammatory markers exacerbate after exposure to milk protein products.

**Conclusion:** We describe a novel variant, deletions of exons 3-9 in *TNFAIP3* gene, in a family with broad spectrum clinical phenotypes. Phenotypes vary in presentation, severity, and age of onset, despite the same genetic variation. Diseases described include autoimmunity with various organ involvement and autoinflammation. Although, in the literature allergy is described as part of the syndrome, we highlight a distinctive clinical and immunological phenotype; milk protein allergy, peripheral eosinophilia and exacerbation of the disease symptoms including elevation of inflammatory markers after exposure to milk protein products. Moreover, milk protein allergy, could be the only manifestation of the disease. Therefore, A20 haploinsufficiency may manifest clinical phenotype that masquerade as allergic disorders.

**Acknowledgement**

We wish to thank the family for the participation in the study

**Patient Consent**

Yes, I received consent

**Disclosure of Interest**

None Declared

### Autoinflammatory diseases

#### P043 Attack triggers in childhood familial Mediterranean fever

##### Elif Kucuk, Fatih Haslak, Kubra Ozturk

###### Pediatric Rheumatology, Istanbul Medeniyet University, İstanbul, Türkiye

####### **Correspondence:** Elif Kucuk

*Pediatric Rheumatology 2024*, **22(2):**PReS24-ABS-1766

**Introduction:** Familial Mediterranean Fever (FMF) is the most common monogenic autoinflammatory disorder. Data regarding the triggers of this rare disease are scarce.

**Objectives:** Patients were diagnosed with FMF according to Eurofever/PRINTO clinical classification criteria. Patients with heterozygous,homozygous, and compound heterozygous mutations in the 10^th^ exon of *the Mediterranean fever gene (MEFV)* were included in the study. Patients with less than six months of follow-up duration were excluded. Patients were interviewed about attack triggers face-to-face between February and May 2024, and other patient information was retrospectively obtained from their medical records.

**Results:** This study included 266 patients (Female: n=141, 53 %). The median age at diagnosis was 60 (6-198) months, and the median age at symptom onset was 48 (1-186) months. Heterozygous mutations were found in 46.6% (n=124) of the patients, homozygous in 30.8 % (n = 82), and compound heterozygous in 22.6 % (n = 60). More than two-thirds of our patients (n=184, 69.2%) had a family history of FMF and 80 patients (30.1%) had consanguineous marriages. The majority of patients (n=246, 92.5%) reported having fewer than 12 attacks per year. The most common presenting symptom was abdominal pain during the attacks (n=169; 63.5%). Attack triggers were identified in 189 patients (71.1%), and the most common was fatigue (n=141; 53%). The others were as follow: prolonged standing (38%), emotional stress (35.7%), exposure to cold (32.3%), insomnia (27.8%), consumption of high-fat food (12%), exercise (11.7%), prolonged travel (10.2%), fasting (9%), menstruation (7.5%), exposure to sunlight (4.1%), physical trauma (1.9%) respectively. In total, 223 patients (83.3%) had attack relievers, and the most common one was the use of nonsteroidal anti-inflammatory drugs (n=183, 68.8%). Biologic agents were required in 13 (4.9 %) patients. Patients with compound heterozygous and homozygous mutations were more likely to benefit from attack-relieving factors (p=0.013). Attack triggers (p=0.023) were more common in patients receiving biological agents. Arthralgia/arthritis was significantly more common in those who reported that standing for longer period as an attack trigger (p<0.001).

**Conclusion:** There is not previously published such a study focused on attack triggers in childhood FMF. Although our study present unique findings,our data based on patient/parent statements. Therefore, our results need to be supported by clinical and laboratory evidence.

**Date of birth:** juin 06, Y

**Patient Consent**

Yes, I received consent

**Disclosure of Interest**

None Declared

**References**

1. Gattorno M, *et al*. Classification criteria for autoinflammatory recurrent fevers. *Ann Rheum Dis* 2019;78:1025-1032.
2. Yenokyan Y, Armenian H.K. Triggers for attacks in familial Mediterranean fever:application of the case-crossover design. Am J Epidemiol.2012 May 15;175(10):1054-61.
3. Karadağ O, *et al*. The factors considered as trigger for the attacks in patients with familial Mediterranean fever. *Rheumatol Int* 2013;33:893-7.

### Autoinflammatory diseases

#### P044 Pediatric-onset familial Mediterranean fever in central-southern Italy: a multicentric retrospective cohort study

##### Saverio La Bella^1^, Roberta Loconte^2^, Mario Muselli^3^, Giulia Di Donato^1^, Armando Di Ludovico^1^, Marco Natale^1^, Violetta Mastrorilli^2^, Andrea Giugno^4^, Santi Papa^4^, Marina Attanasi^5^, Francesco Chiarelli^5^, Stefano Necozione^3^, Patrizia Barone^4^, Francesco La Torre^2^, Luciana Breda^1^

###### ^1^Department of Pediatrics - Pediatric Clinic and Rheumatology Unit, University “G. D’Annunzio” of Chieti, Chieti; ^2^Department of Pediatrics, Giovanni XXIII Pediatric Hospital, University of Bari, Bari; ^3^Department of Life, Health and Environmental Sciences, Public Health Section, University of L’Aquila, L’Aquila; ^4^Department of integrated Maternal-Child and Reproduction Activity, “Policlinico-San Marco” University of Catania, Catania; ^5^Department of Pediatrics - Pediatric Clinic, University “G. D’Annunzio” of Chieti, Chieti, Italy

####### **Correspondence:** Saverio La Bella

*Pediatric Rheumatology 2024*, **22(2):**PReS24-ABS-1495

**Introduction:** Familial Mediterranean Fever (FMF) is the most common autoinflammatory disease, with an onset under the age of 20 observed in 80% of cases.

Countries with the highest prevalence are Turkey (1:400—1:1000), Armenia (1:500), and Israel (1:1000)^1^. The prevalence of FMF in Italy is unknown, but recently estimated at around 1:60000^2^.

**Objectives:** The aim of this study was to describe the children who had a diagnosis of FMF in three pediatric rheumatology centres in central and southern Italy.

**Methods:** Pediatric patients followed by the pediatric rheumatology centres of Chieti (Abruzzo), Bari (Apulia), and Catania (Sicily) who were diagnosed with FMF according to the EuroFever/PRINTO classification criteria^3^ were retrospectively evaluated; patients without a genetic test or carrying only a variant of unknown significance (VUS) were excluded.

**Results:** Overall, 52 patients were enrolled (31 females, 21 males), with a mean age at onset of 4.1 ± 3.3 years, a mean age at diagnosis of 10.4 ± 10.4 years, and a mean follow-up period of 7.9 ± 8.4 years.

30 patients were born in Abruzzo (57.7%), 11 in Apulia (21.2%), 9 in Sicily (17.3%), 1 in Marche (1.9), and 1 in Molise (1.9%).

The *MEFV* genotype was in heterozygosity in 27 patients (52.0%), in compound heterozygosity in 15 patients (28.8%), and in homozygosity in 10 patients (19.2%). M694V was the most common pathogenic *MEFV* variant, observed in 24 patients (46.2%). The most common *MEFV* genotype was M694V/- (11 patients, 21.2%), followed by M694V/M694V (6 patients, 11.5%), and M680IGA/- (5 patients, 9.6%).

At onset, recurrent fever was observed in 51 patients (98.1%), with a mean duration of 2.2 ± 1.2 days, a mean maximum body temperature (°C) of 39.5 ± 0.9, and a mean time between attacks of 20.8 ± 15.4 days. In addition, the most common clinical features at onset were abdominal pain (75%), lymphadenopathy (48.1%), arthralgias (38.5%), and chest pain (23.1%). Overall, the most prevalent clinical manifestations were recurrent fever (100%), abdominal pain (92.3%), arthralgias (71.2%), lymphadenopathy (51.9%), oral aphthosis (42.3%), and chest pain (34.6%). Headache occurred in 7 patients at onset (13.5%) and in 22 patients overall (42.3%). Arthritis, proteinuria, and erysipelas-like erythema were observed in 7 (13.5%), 4 (7.7%), and 2 (3.8%) patients, respectively.

Serum amyloid A was found elevated between attacks in 9 patients (17.3%). At the last examination, 28 patients were on colchicine (53.8%), 2 were on biologic treatment (3.8%), 5 were on colchicine plus a biologic drug (9.6%), and 17 without any therapy (32.7%). Canakinumab was the most used biologic drug (7 patients, 13.5%).

According to the International Severity Score for FMF (ISSF) 40 patients had a mild disease (81.6%), 9 had an intermediate disease (18.4%), and nobody had a severe disease (3 missing).

**Conclusion:** FMF is a relevant health issue in children living in central-southern Italy, and probably its prevalence in this country is higher than expected.

A larger, multicentric national study should be performed to better understand the epidemiology and other relevant aspects of FMF in Italian children.

**Patient Consent**

Not applicable (there are no patient data)

**Disclosure of Interest**

None Declared

**References**

1. Ben-Chetrit et al *Arthritis Rheum* 2009
2. Aiello A et al *J Mark Access Health Policy* 2023
3. Gattorno et al *Ann Rheum Dis* 2019

### Autoinflammatory diseases

#### P045 Evolving phenotypic and genotypic spectrum of human ISG15 and USP18 deficiencies

##### Alhanouf Alsaleem^1^, Wafaa Abdulghaffar^1^, Lujain Akbar^2^, Meshal Alhassan^2^, Fatimah Alkhars^3^, Sulaiman Al-Mayouf^1^

###### ^1^KFSHRC, RIYADH; ^2^Imam Abdulrahman Bin Faisal University, Dammam; ^3^Maternity and Children Hospital, Alhassa, Saudi Arabia

####### **Correspondence:** Wafaa Abdulghaffar

*Pediatric Rheumatology 2024*, **22(2):**PReS24-ABS-1521

**Introduction:** IFN-mediated diseases are mendelian innate immunodysregulatory disorders comprise an expanding group of monogenic diseases that present early in life with fever, sterile organ inflammation, and a high type-I IFN-response gene signature. Loss of negative regulator in ISG15 and USP18 results in recently described condition with diversity of clinical charecterstics related to enhanced IFN-a/b immunity.

**Objectives:** To describe the phenotype, genotype and outcome of Saudi children with proved pathogenic mutation of ISG15/USP18 genes.

**Methods:** This is a multicenter descriptive study of pediatric patients with final genetically confirmed type I interferonopathies and ISG15/USP18 gene mutation. Medical records were reviewed for demographic, family history, clinical and genetic data.

**Results:** Total of fourteen patients from unrelated six Saudi families; only nine had proven gene mutation. Five patients (55%) presented within the first two years. Median age of disease onset was twenty-four months. Constitutional features were found in all patients. The most frequent organ involvements were neurology (77%), interstitial lung diseases (55%), cutaneous abscess (55%), and musculoskeletal (66%). All patients had elevated inflammatory markers. Two patients had severe macrophage activation syndrome. Autoantibodies were evident in five patients; two of them developed lupus like disease. Four patients had abnormal immunology work up and three had recurrent infections. Genetic sequencing identified ISG15 gene mutation in eight patients, of which all had novel genetic variants. Only one patient had USP18 gene mutation with previously reported variant. Majority of the patients were treated with corticosteroids (n=8), intravenous immunoglobulin (n=3), Janus kinase inhibitors (n=2), in addition to various other immunosuppressive agents. Only one patient died with multiorgan failure.

**Conclusion:** Loss of negative regulator in ISG15 and USP18 genes highlight a recently described interferon mediated disease. This the first and largest cohort from the Arab region to date. In this report we expand the clinical and genetic spectrum with novel identified variants of this seldom reported disease entity along with diversity of disease severity and outcome.

**Patient Consent**

Yes, I received consent

**Disclosure of Interest**

None Declared

### Autoinflammatory diseases

#### P046 Comparative analysis of anakinra and canakinumab in colchicine-resistant familial Mediterranean fever: an assessment of early period efficacy and safety

##### Sengul Caglayan, Taner Coşkuner, Kadir Ulu, Betül Sözeri

###### Department of Pediatric Rheumatology, University of Health Sciences, Umraniye Training and Research Hospital, İstanbul, Türkiye

####### **Correspondence:** Sengul Caglayan

*Pediatric Rheumatology 2024*, **22(2):**PReS24-ABS-1560

**Introduction:** Familial Mediterranean fever (FMF) is the first-described and most common autoinflammatory disease.The primary pharmacological intervention involves the use of colchicine. However, approximately 5-10% of patients are resistant to colchicine. Anti-IL-1 agents such as anakinra or canakinumab are used in these patients. Although many studies have been conducted on these two treatment agents, there has not been a study comparing the data of the two treatment regimens head-to-head.

**Objectives:** The aim of our study was to compare the treatment responses and safety data of patients with colchicine-resistant FMF (crFMF) who were treated with anakinra and canakinumab.

**Methods:** Patients who were followed up with a diagnosis of crFMF at the Pediatric Rheumatology Department, Clinic of Umraniye Training and Research Hospital between June 2016 and February 2024 were included in the study. The study included 91 patients who initiated biological treatment with anakinra or canakinumab for the first time. Patients who were switched to each other were excluded from the study. The efficacy of the treatments was assessed after a three-month period.

**Results:** A total of 91 patients (F/M: 52/39) were included in the study. The median age at study, age at diagnosis, and age at initiation of biological treatment were 15 (4-22), 5 (1-17) and 13 (2-19) years, respectively. Thirty-four patients (37.3%) had consanguinity, and 51 patients (56%) had a family history of FMF. The most common genetic mutation was M694V homozygous variant. The most common clinical findings were fever (98.9%), abdominal pain (97.8%), and arthralgia (67%). Myalgia (49.4%), chest pain (41.7%), arthritis (40.6%), exertional leg pain (24.1%), diarrhea (22%), and erysipelas-like erythema (19.8%) were other findings accompanying the attacks. Amyloidosis was also present in two patients. There was no statistically significant difference in the frequency of attacks, AIDAI scores, or serum amyloid A levels after biological treatment in anakinra and canakinumab groups (p=0.251, p=0.198, p=0.107, respectively). In the total patient cohort, 12 side effects were observed. In the anakinra group allergic reaction (n=5) were the most common side effect. In the canakinumab group, acne rosacea (n=1), hydroadenitis suppurativa (n=1), psoriasis (n=1), frequent acute upper respiratory tract infection (n=1), and acute myeloid leukemia (n=1) were the most common side effects.

**Conclusion:** In this study, the efficacy and safety data of two treatment agents used in patients with crFMF were compared. It was concluded that both drugs had similar efficacy and safety in reducing attacks, suppressing inflammatory markers, and reducing disease activity. The main limitation of the study is that it was performed in a single center based on retrospective data, and it is thought that the data obtained should be confirmed by randomized controlled multicenter studies.

**Date of birth:** septembre

**Patient Consent**

Yes, I received consent

Disclosure of Interest

None Declared

### Autoinflammatory diseases

#### P047 Beyond undifferentiated recurrent fever syndrome (SURF): a compelling case with hypertriglyceridemia induced recurrent pancreatitis

##### Esma Aslan^1^, Nergis Akay^1^, Umit Gul^1^, Elif Kilic Konte^1^, Aybuke Gunalp^1^, Fatih Haslak^1^, Mehmet Yildiz^1^, Amra Adrovic^1^, Kenan Barut^1^, Ayse C. Aktuglu Zeybek^2^, Sezgin Sahin^1^, Ozgur Kasapcopur^1^

###### ^1^Pediatric Rheumatology; ^2^Division of Nutrition and Metabolism Department of Pediatrics., Istanbul University-Cerrahpasa, Cerrahpasa Medical Faculty , Istanbul, Türkiye

####### **Correspondence:** Esma Aslan

*Pediatric Rheumatology 2024*, **22(2):**PReS24-ABS-1700

**Introduction:** Syndrome of undifferentiated recurrent fever (SURF) is a heterogeneous group of autoinflammatory diseases (AID) characterized by self-limiting episodes without a confirmed molecular diagnosis and a complete or partial response to colchicine [1-3].

**Objectives:** A 9-year-old girl was admitted with periodic fever and abdominal pain, which occurred 3-4 times in the last three months and lasted for 3-10 days. The acute phase reactants were also found to be elevated during these episodes. Following these attacks, clinical and laboratory features were completely recovered. Colchicine treatment was started with a preliminary diagnosis of familial Mediterranean fever (FMF), albeit normal MEFV gene analysis. With partial colchicine-responsive fever-abdominal pain attacks and normal MEFV gene analysis, the patient was considered as SURF. Approximately one attack per 2 months occurred within the 12 months following the SURF diagnosis. Markedly elevated SAA (311 mg/L; Normal range:0-6.4) and C-reactive protein (CRP) (257 mg/L; Normal range:0-5) levels and heterogeneous fluid collection with dense septations in abdominal ultrasonography were observed during the attacks. An auto-inflammatory panel was requested to check for other periodic fever syndromes. Autoinflammatory gene panel yielded a c.2113C>A p.(Gln705Lys) missense heterozygous variant (VUS in AGCM criteria) in the NLRP3 gene. Interestingly, some of these attacks involved moderate elevations in serum creatinine levels (3.41 mg/dL). The creatinine level, which an auto-inflammatory disease attack could not explain, was found to be within the normal range for his age (0.55 mg/dl) when rechecked a few days later. In addition, the lipemic index of the serum sample was as high as 1948 (Figure-1). Lipid panel resulted with a triglyceride level of 1082 mg/dL (normal range: 114-200). So, it was understood that the patient’s abdominal pain and fever episodes were due to recurrent pancreatitis due to hypertriglyceridemia, and the occasional high urea and creatinine levels were possibly a result of acute renal failure due to pancreatitis. The patient is being followed up in the Division of Nutrition and Metabolism Department of Pediatrics.

**Conclusion:** This case showed that recurrent pancreatitis due to hypertriglyceridemia should be included in the differential diagnosis of SURF patients.

**Date of birth::** avril 23,

**Patient Consent**

Yes, I received consent

**Disclosure of Interest**

None Declared

**References**

1. Papa R, Penco F, Volpi S, et al. Syndrome of Undifferentiated Recurrent Fever (SURF): An Emerging Group of Autoinflammatory Recurrent Fevers. J Clin Med. 2021;10(9).
2. Orange DE, Yao V, Sawicka K, et al. RNA Identification of PRIME Cells Predicting Rheumatoid Arthritis Flares. N Engl J Med. 2020;383(3):218-28.
3. Sutera D, Bustaffa M, Papa R, et al. Clinical characterization, long-term follow-up, and response to treatment of patients with syndrome of undifferentiated recurrent fever (SURF). Semin Arthritis Rheum. 2022;55:152024.

### Autoinflammatory diseases

#### P048 Pediatric Behçet’s disease: clinical profiles and cluster analysis

##### Joana B. Lima^1^, Sérgio Alves^2^, Carla Zilhão^2^

###### ^1^Paediatric Department; ^2^Paediatric Rheumatology Unit, Centro Materno Infantil do Norte, Unidade Local de Saúde de Santo António, Porto, Portugal

####### **Correspondence:** Joana B. Lima

*Pediatric Rheumatology 2024*, **22(2):**PReS24-ABS-1775

**Introduction:** Behçet’s Disease (BD) is an autoinflammatory (AI) condition characterized by variable vessel vasculitis, that involves oral and genital mucosa, skin, eyes, joints, gastrointestinal (GI) and central nervous systems. There has been an effort to find phenotypic cluster to tailor individualized management for patients with BD.

**Objectives:** The aim of this study was to characterize both clinical features and phenotypic traits of pediatric BD, while analyzing clustering patterns.

**Methods:** Single center retrospective study of patients, with BD diagnosis under 18 years, followed between Jan2016-Dec2023. Patients were divided into 4 clusters according to disease involvement (C1–purely mucocutaneous; C2–ocular; C3–articular; C4–GI).

**Results:** Thirty-two patients were included. **C1** (n=6, 18.8%) showed a female predominance (83.3%), with a mean age at diagnosis of 12.5±3.4 years. All patients (n=6, 100%) presented with oral aphthous lesions, and 5(83.3%) with genital ulcers. A lower mean age of genital ulcer presentation was observed (10.4±1.3vs13.1±2.7, p=0.045) when compared with non-C1 patients. Two (33.3%) had pseudofolliculitis, and 1 (25%) erythema nodosum. There was also a predominance of HLA-B15 (50%, p=0.032, OR=10.5, 95%CI=0.9-120.3). **C2** (n=4, 12.5%) had an equal gender distribution, with a mean age at diagnosis of 13.0±2.9 years. Anterior uveitis was present in 3 (75%), and retinal vasculitis in 2 (50%). Three (75%) had genital ulcers and cutaneous involvement. None had GI or articular involvement. **C3** (n=10, 31.3%) also exhibited a female predominance (70%) with a mean age at diagnosis of 13.4±2.3years. Nearly all developed articular complaints during disease course, but only 2 (22.2%) had arthritis. Eight (80%) had cutaneous involvement, and 4 (40%) GI. The presence of a BD family history was higher (50%vs9.1%, p=0.009, OR=10, 95%CI=1.5-67.6). **C4** (n=11, 34.4%) also demonstrated a female predominance (63.6%), with a mean age at diagnosis of 13.6±2.5 years. Four (36.4%) patients presented symptoms at diagnosis, and 7(25%) afterwards. Six (54.5%) had cutaneous involvement, and 3(27.3%) articular involvement. None with ocular lesions. There was a higher probability of a previous history of PFAPAs in this cluster (36.4%vs0%, p=0.09). Azathioprine was significantly more commonly used (63.6%vs23.8%,p=0.027,OR=5.6, 95%CI=1.1-27.4).

**Conclusion:** This study demonstrates distinct clinical phenotypes among pediatric BD patients. Female predominance was observed in patients in C1, C3 and C4. C1 had a higher probability of presence of HLA-B15, suggesting a different potential genetic susceptibility between groups. C3 was associated with a family history of BD, suggesting familial aggregation in joint-involved patients, which is in accordance with recent literature. A history of PFAPAs was more common in C4. These findings not only emphasize the heterogeneous nature of pediatric BD but also hint at targeted therapeutic interventions tailored to specific phenotypic clusters.

**Date of birth:** 9/13/1995 1

**Patient Consent**

Not applicable (there are no patient data)

**Disclosure of Interest**

None Declared

### Autoinflammatory diseases

#### P049 A retrospective study of patients with early-onset familial Mediterranean fever and patients with periodic fever, pharyngitis, aphthous stomatitis and adenitis syndrome

##### Pınar Garipçin, Ayşenur Paç Kısaarslan, Aydan Yekedüz Bülbül, Eda Kayhan, Sümeyra Özdemir Çiçek, Muammer Hakan Poyrazoğlu

###### Pediatric Rheumatology, Erciyes Univercity, Kayseri, Türkiye

####### **Correspondence:** Ayşenur Paç Kısaarslan

*Pediatric Rheumatology 2024*, **22(2):**PReS24-ABS-1804

**Introduction:** Familial Mediterranean fever (FMF) is the prototype of single-gene autoinflammatory diseases. Attacks may be atypical, especially in early life, and serositis may be less severe. Periodic fever, aphthous stomatitis, pharyngitis and adenitis (PFAPA) syndrome is the most common periodic fever syndrome in childhood. It’s most common in children under 5 years of age and it’s difficult to differentiate the attacks from FMF.

**Objectives:** We aimed to compare the characteristics of FMF patients with clinical onset before the age of 5 years with PFAPA patients, and to identify the clinical and laboratory findings that may help differentiate the attacks.

**Methods:** The study included 99 FMF and 58 PFAPA patients whose clinical symptoms started before the age of 5 years. Demographic data, clinical and laboratory findings, treatments, and any complications of the disease were obtained from the hospital information system. The characteristics of the two groups were compared.

**Results:** The frequency of aphthous stomatitis (53.9% vs 4%, p<0.001) pharyngitis (65.5%vs 4%, p<0.001), and lymphadenitis (75.9%vs 5.1%, p<0. 001) were more frequent in the PFAPA group, while abdominal pain (94.9%vs43.1%, p<0,001), chest pain (38.4%vs 1.7%, p<0.001), arthralgia (73.7%vs 38.3%, p<0.001) and diarrhea (27.3%vs 5.2%, p<0.001) were more frequent in the FMF group. The presence of prodromal symptoms before the attack was seen in 14 patients (14%) in the FMF group, while it was seen in 27 patients (46.6%) in the PFAPA group (p<0.001) In laboratory examinations during disease flares, median WBC count was 14275 (4200-26260), neutrophil count was 9625 (4000-21000) and CRP value was 106 (26-274) mg/L, and were significantly higher in the PFAPA group (p<0.001, p<0.001, p=0.026, respectively). Cut off values for differentiation PFAPA flares from FMF flares were WBC count 12.835/mm³ (AUC:0.775 95%CI:0.691-0.859, p<0.001) with 71% sensitivity and 71% specificity, neutrophil count 7.855/mm³ (AUC:0. 786 %95CI:0.705-0.867, p<0.001) with 75% sensitivity and 73% specificity and CRP value 99.5 mg/L (AUC:0.633 %95CI:0.521-0.745, p=0.026) with 62% sensitivity and 60% specificity.

**Conclusion:** In our study, the clinical findings of FMF patients with early-onset FMF and PFAPA patients during attacks were different, as expected. We concluded that a good medical history, and assessment of clinical features of flares can help to differentiate these two diseases. The presence of prodromal symptoms before an attack, higher white blood cell, neutrophil counts, and CRP levels in the laboratory findings may be helpful in the differential diagnosis of attacks of the two diseases.

**Date of birth:** janvier 03

**Patient Consent**

Not applicable (there are no patient data)

**Disclosure of Interest**

None Declared

**References**

1. Gezgin Yildirim D, Gönen S, Fidan K, Söylemezoğlu O. Does Age at Onset Affect the Clinical Presentation of Familial Mediterranean Fever in Children? J Clin Rheumatol. 2022 Jan 1;28(1):e125-e128. doi: 10.1097/RHU.0000000000001637. PMID: 33252389.
2. Wang A, Manthiram K, Dedeoglu F, Licameli GR. Periodic fever, aphthous stomatitis, pharyngitis, and adenitis (PFAPA) syndrome: A review. World J Otorhinolaryngol Head Neck Surg. 2021 Jun 27;7(3):166-173. doi: 10.1016/j.wjorl.2021.05.004. PMID: 34430824; PMCID: PMC8356195.

### Autoinflammatory diseases

#### P050 Idiopathic or genetic? The story of a patient diagnosed with JIA and intestinal lung disease

##### Małgorzata Gdak^1^, Anna Bodajko-Grochowska^2^, Katarzyna Koman^1^, Arkadiusz Jędrzejewski^2^, Monika Lejman^3^, Violetta Opoka-Winiarska^2^

###### ^1^Department of Pediatric Pulmonology and Rheumatology, University Children’s Hospital; ^2^Department of Pediatric Pulmonology and Rheumatology; ^3^Laboratory of Genetic Diagnostics, Medical University of Lublin, Lublin, Poland

####### **Correspondence:** Violetta Opoka-Winiarska

*Pediatric Rheumatology 2024*, **22(2):**PReS24-ABS-1806

**Introduction:** Autoinflammatory type I interferonopathies are a group of disorders caused by molecular defects leading to the activation of the production of type I interferon.

Stimulator of interferon genes [STING] associated vasculitis of infancy (SAVI) is the result of a dominant missense mutation in the STING1 gene.

**Objectives:** To present the case of a 12-year-old female patient with juvenile idiopathic arthritis (JIA) diagnosed at the age of 6, complicated by interstitial lung disease at the age of 10.

**Methods:** Patient’s medical records were reviewed. Analysis of Whole Exome Sequencing (WES) was performed.

**Results:** The first symptoms of arthritis occurred in the patient at the age of 6 - she was diagnosed with oligoarticular onset JIA. Initially, intra-articular glucocorticosteroids were used in the treatment, followed by methotrexate, which was continued for 2 years, achieving remission.

The first respiratory symptoms occurred at the age of 10 as pneumonia that did not respond to standard treatment. Other symptoms at this time: weight loss, liver enlargement, auscultatory changes, without signs of joints’ inflammation. Bodyplethysmography showed a decrease in TLC. CT scan of the lungs revealed a ground glass image. The slow progression of respiratory failure symptoms, deterioration of the lung’s diffusion capacity, and signs of significant restriction at the expense of VC were observed.

Analysis of WES revealed the presence of a missense mutation, in a heterozygous system, in the *STING1* gene (OMIM 612374). The detected variant (rs587777610) was described in the HGMD2 and ClinVar3 databases as pathogenic. These mutations have been described in patients with infantile-onset *STING*-related vasculopathy (OMIM 615934)4, with autosomal dominant inheritance. The diagnosis was SAVI syndrome. According to recommendations [1], a Janus Kinase inhibitor (tofacitinib) with a bridging dose of prednisone was started. Currently, 12 months of treatment are ongoing with clinical improvement. No side effects of the drug have been observed so far. The girl is constantly monitored for infections.

**Conclusion:** The interstitial lung disease is considered a rare complication of JIA. In this case, genetic testing showed that it was not an idiopathic disease. Additionally, we showed a different clinical manifestation of SAVI, with a later onset.

**Patient Consent**

Yes, I received consent

**Disclosure of Interest**

None Declared

**Reference**

1. Cetin Gedik K, Lamot L, Romano M, et al. The 2021 European Alliance of Associations for Rheumatology/American College of Rheumatology points to consider for diagnosis and management of autoinflammatory type I interferonopathies: CANDLE/PRAAS, SAVI and AGS. Ann Rheum Dis. 2022;81(5):601-613.

### Autoinflammatory diseases

#### P051 Monitoring liver function after colchicine treatment in familial Mediterranean fever patients

##### Büşra Başer Taşkın, Ayşenur Doğru, Selen D. Arık, Bengisu Menentoğlu, Nuray Aktay Ayaz

###### Pediatric Rheumatology, Istanbul university, Istanbul, Türkiye

####### **Correspondence:** Büşra Başer Taşkın

*Pediatric Rheumatology 2024*, **22(2):**PReS24-ABS-1812

**Introduction:** Familial Mediterranean Fever (FMF) is an autosomal recessive disorder characterized by recurrent febrile episodes and inflammatory attacks. Colchicine is the mainstay of treatment for FMF, aimed at reducing the frequency and severity of attacks. However, liver function abnormalities have been reported as potential adverse effects of colchicine therapy.

**Objectives:** This study aims to assess the monitoring of liver function tests in FMF patients undergoing colchicine treatment.

**Methods:** The medical records of FMF patients followed-up in pediatric rheumatology clinic at least two years were analyzed retrospectively. Demographic data, laboratory results, and the progression of liver enzyme elevations were documented.

**Results:** During the follow-up period, liver enzyme elevation was detected at least once in 21 out of 915 patients (2.3%). The median follow-up duration at the time of liver enzyme elevation was 18 (IQR: 13.25-65.0) months. Among these patients, 14 (66.7%) were male. The mean age of the cohort was 12.45 ± 4.56 years, with a mean age of diagnosis at 5.31 ± 3.07 years. The most common MEFV gene mutations observed in patients were as follows: M694V homozygous in 5 cases (23.8%), M694V heterozygous in 4 cases (19%), and M694V/V726A compound heterozygous in 4 cases (19%).

All patients received colchicine treatment within the recommended dosage limits based on their age and weight; none exceeded the maximum prescribed dose. One patient, due to an inadequate response to colchicine, was concurrently treated with canakinumab, another received azathioprine and salazoprine for Crohn’s disease, and out of the three patients with JIA, two were on methotrexate, while one was on adalimumab. The median AST value was 56 (IQR: 44-68) U/L, and the median ALT value was 58 (IQR: 50-86) U/L. Upon detecting liver enzyme elevation, colchicine dosage was reduced in two patients, and treatment was temporarily discontinued in one patient. Infection was found to be associated with liver enzyme elevation in five patients (23.8%), while hepatosteatosis was identified in three patients (14.3%). Two patients were referred to the gastroenterology outpatient clinic, while the liver enzyme status of the remaining patients was monitored in the pediatric rheumatology outpatient clinic. Median elevation duration of liver enzymes was one month (IQR: 1-2.5). None of the patients progressed to fulminant hepatitis, and liver enzymes spontaneously normalized in all cases.

**Conclusion:** Regular monitoring of liver function tests is imperative for patients with Familial Mediterranean Fever (FMF) undergoing colchicine therapy. Colchicine treatment appears to be safe in FMF patients.

**Date of birth::** juillet 12

**Patient Consent**

Not applicable (there are no patient data)

**Disclosure of Interest**

None Declared

### Autoinflammatory diseases

#### P052 Chronic Non-bacterial Osteomyelitis (CNO) in a patient with myeloperoxidase (MPO) deficiency

##### Zoya Nesterenko, Vasiliy Burlakov, Anna Kozlova, Oyuna Lodoeva, Ekaterina Adamanskaya, Sofiya Galkina, Amina Kieva, Anastasia Sveshnikova, Yulia Rodina, Elena Raikina, Dmitriy Pershin, Anna Shcherbina

###### Dmitry Rogachev National Medical Research Center of Pediatric Hematology, Oncology and Immunology, Moscow, Russian Federation

####### **Correspondence:** Zoya Nesterenko

*Pediatric Rheumatology 2024*, **22(2):**PReS24-ABS-1578

**Introduction:** CNO is an autoinflammatory disease typically affecting children and adolescents and predominantly (but not exclusively) characterized by osteolytic as well hyperostotic/osteosclerotic lesions.

MPO deficiency is the most common inborn defect of phagocytes. MPO is a hemoprotein expressed in azurophilic granules of neutrophils and in the lysosomes of monocytes, with strong antibacterial properties mediated by its ability to generate hypochlorous acid MPO deficiency is clinically characterized by increased susceptibility to infection and chronic inflammation.

CNO in a patient with MPO deficiency was previously described by Sundqvist et al(2023).

**Objectives:** To describe the second case of CNO in a patient with MPO deficiency.

**Methods:** Genetic testing was performed using NGS targeted panel, with subsequent Sanger confirmation of the variant in the patient and his carrier parents. MPO expression and active oxygen production (Rhodamine test) were assessed via flow cytometry. Granulocyte activity was analyzed via Nikon Ti2 confocal microscope in two modes. Granulocyte chemotaxis velocity was assessed in parallel-plate flow chambers with collagen, NET-osis was assessed in fixed blood smears stained with anti-elastase antibodies.

**Results:** Male patient was diagnosed at the age of 7 with CNO affecting Th12, S4-S5 vertebrae, left femur and fibula, coccyx, mandible based on typical MRI and histological findings. Consecutive treatment with nonsteroidal anti-inflammatory drugs, corticosteroids, bisphosphonates did not stop the disease progression.

The patient was started on TNFa inhibitor adalimumab, with immediate response. After 6 years of adalimumab treatment and stable remission the treatment was discontinued, resulting in rapid symptom recurrence. When adalimumab was re-introduced, the patient did not respond. The patient was started Janus-kinase inhibitor (tofacitinib), effectiveness has yet to be assessed.

At the same time MPO deficiency was confirmed by detection of compound heterozygous c.493del, p.Val165Ter and c.1642C>T, p.Arg548Trp *MPO* variants. MPO expression was absent and active oxygen production drastically reduced.

We showed that granulocytes NET-osis was compared to normal control, with domination of suicidal NET-s visualized by co-localization of extracellular DNA strands with neutrophil elastase. Yet, neutrophil adhesion to collagen and their chemotaxis velocities were significantly increased.

**Conclusion:** The ethiopathological connection of the MPO deficiency and CNO, as well the implications for the treatment options requires further investigation. CNO is considered a multifactorial autoinflammatory disorder, yet we feel it is important to study patients for genetic defects including MPO deficiency.

**Patient Consent**

Yes, I received consent

**Disclosure of Interest**

None Declared

### Autoinflammatory diseases

#### P053 Interferonopathy or NF-kappab-pathy: a case report

##### Mario Sestan^1^, Mirna Bradamante^2^, Matej Sapina^3^, Marijan Frkovic^1^, Danica Grguric^1^, Lana Omerza^1^, Zlatko Marusic^4^, Stefano Volpi^5^, Adriana de Jesus^6^, Raphaela Goldbach-Mansky^6^, Marija Jelusic^1^

###### ^1^Department of Paediatrics, University Hospital Centre Zagreb, University of Zagreb School of Medicine; ^2^Depratment of Dermatology, University of Zagreb School of Medicine, University Hospital Centre Zagreb, Zagreb; ^3^Department of Paediatrics, University of Osijek School of Medicine, University Hospital Centre Osijek, Osijek; ^4^Depratment of Pathology, University of Zagreb School of Medicine, University Hospital Centre Zagreb, Zagreb, Croatia; ^5^Istituto Giannina Gaslini, Università degli Studi di Genova, Genoa, Italy; ^6^National Institute of Allergy and Infectious Diseases (NIH) Bethesda, Bethesda, United States

####### **Correspondence:** Mario Sestan

*Pediatric Rheumatology 2024*, **22(2):**PReS24-ABS-1405

**Introduction:** Interferonopathies and NF-kappaB-pathies represent a newly identified subset of autoinflammatory conditions. They are characterized by dysregulation in either the type I interferon or NF-kappaB signaling pathways, resulting in their continual overactivation or inadequate regulation of negative feedback mechanisms.

**Objectives:** We present a case of monogenic autoinflammatory disease exhibiting features of both interferonopathy and NF-kappaB-pathy.

**Methods:** Information about the patient was collected from the medical records.

**Results:** The disease onset occurred immediately after birth, characterized by painful erythematous subcutaneous infiltrates, initially on the face and later spreading to the trunk and extremities. The patient experienced intermittent fevers and exhibited elevated inflammatory markers including hyperferritinemia, hypercomplementemia, hyperfibrinogenemia, increased sedimentation rate, elevated C-reactive protein, leukocytosis with neutrophilia, and thrombocytosis. Transaminase levels and dyslipidemia were also elevated. Notably, cerebrospinal fluid analysis revealed pleocytosis with monocyte predominance and significant hypoglycorachia, although extensive microbiological investigations failed to identify an underlying infection explaining the prolonged inflammatory state. Skin biopsy demonstrated histological features consistent with lobular panniculitis. Considering the clinical presentation, exclusion of infectious and malignant etiologies, and a highly positive interferon signature, an autoinflammatory disorder was suspected. CANDLE syndrome emerged as the leading candidate in the differential diagnosis.Subsequently, the patient developed cyclical episodes of abdominal pain accompanied by hematochezia and diarrhea. Esophagogastroduodenoscopy and colonoscopy revealed acute colitis with increased eosinophils on histological examination. Following the diagnosed condition, treatment commenced with systemic glucocorticoids, initiated at gradually decreasing doses alongside the Janus kinase inhibitor, baricitinib. This regimen led to partial improvement, though full disease control was not achieved. Subsequently, whole exome sequencing identified a pathogenic variant in exon 5 (c.671+1G>C) of the IKBKG gene, confirming the diagnosis of NEMO-NDAS (NEMO deleted exon 5-autoinflammatory syndrome). Therapy with a tumor necrosis factor alpha inhibitor (adalimumab) was initiated. Despite treatment, the patient continues to experience febrile episodes with elevated inflammatory markers and recurrent skin manifestations. Additionally, there has been significant growth delay, with the patient weighing only 6000 grams at 18 months of age.

**Conclusion:** Interferonopathies and NF-kappaB-pathies denote a recently identified category of autoinflammatory conditions. It’s crucial for clinicians to recognize the clinical indicators of disruptions in the type I interferon and NF-kappaB pathways to suspect these conditions accurately and guide appropriate diagnostic investigations.

**Patient Consent**

Yes, I received consent

**Disclosure of Interest**

None Declared

### Autoinflammatory diseases

#### P054 Sirolimus as a steroid-sparing agent in a patient affected by Cleavage-resistant RIPK1-induced Autoinflammatory (CRIA) syndrome

##### Manuela Cortesi^1^, Francesca Ricci^1^, Raffaele Badolato^2^, Marco Cattalini^2^

###### ^1^Pediatrics, ASST Spedali Civili di Brescia; ^2^Pediatrics, University of Brescia, ASST Spedali Civili of Brescia, Brescia, Italy

####### **Correspondence:** Manuela Cortesi

*Pediatric Rheumatology 2024*, **22(2):**PReS24-ABS-1257

**Introduction:** Cleavage-resistant RIPK1-induced autoinflammatory (CRIA) syndrome is an autosomal-dominant autoinflammatory disease (AID) characterized by early-onset periodic fever, lymphadenopathies, oral ulcers, abdominal pain, splenomegaly, and arthralgia.

The loss of regulatory mechanisms of RIPK1 activity provokes increased levels of inflammasome-related cytokines (IL-1, IL-18) and inflammatory cell death, which cause an overproduction of IL-6 and TNF (1).

Some patients with CRIA displayed partial response to anti IL-6, while others failed to respond (2).

RIPK1 cleavage limits both necroptosis and caspase-8-mediated apoptosis (2). As result, in cleavage-resistant RIPK1, there is unchecked activation of the extrinsic apoptotic pathway triggered by caspase-8 activation, leading to heightened stimulation of mTOR pathway. Increased mTOR activation can be inhibited by mTOR inhibitors, such as sirolimus (3).

**Objectives:** The aim of this study was to assess the clinical and laboratory response to sirolimus therapy in a patient with CRIA syndrome, evaluated in terms of number of days of fever and of cumulative steroid dose per kg/month and CRP levels after starting sirolimus.

**Methods:** We describe an 18-year-old girl presenting at one month of life with recurrent febrile episodes, treated with steroids.

A research exome sequencing, which was performed at the age of 14 years, revealed a *de novo* heterozygous pathogenic variant c.970G>C (p.Asp324His) in *RIPK1*, suggesting the diagnosis of CRIA syndrome. The patients received multiple treatments, encompassing anti IL-1, colchicine, azathioprine, mycophenolate mofetil without a satisfactory response. A slight improvement was observed after therapy with tocilizumab, stopped after occurrence of a severe infusion reaction. The only effective drug in controlling fever episodes was prednisone, with meaningful collateral effects (growth and pubertal delay).

**Results:** Sirolimus was administered for off-label use after receiving a favorable opinion from the Regional Center for Rare Diseases and obtaining informed consent.

We collected data from 16 months before up to 24 months after the starting of sirolimus (blood concentration 4-9,4 ng/ml).

Before sirolimus treatment, the average number of days with fever,lymphadenopathies, and abdominal pain per month was approximately 13.7 days; after treatment, the average dropped to about 3.2 days per month (p<0.0001).

The average steroid dose was 1.225 mg/kg/month before and 0.119 mg/kg/month after treatment (p<0.0001).

Regarding CRP values, the average CRP was 81 mg/l before and 14 mg/l after treatment (p<0.0001).

No side effects related to sirolimus occurred, other than the occurrence of recurrent oral ulcers.

**Conclusion:** Sirolimus effectively improved the patient’s clinical manifestation, allowing a statistically significant reduction in the dosage of steroids.

Sirolimus may represent an alternative and effective therapeutic approach for patients affected by CRIA syndrome. However, since sirolimus has not been tested in other patients, additional studies and specific clinical trials aimed at assessing the efficacy of the response to sirolimus are needed.

**Patient Consent**

Yes, I received consent

**Disclosure of Interest**

None Declared

**References**

1. Tao P, Sun J, et al. A dominant autoinflammatory disease caused by non-cleavable variants of RIPK1. Nature. 2020 Jan 2;577(7788):109–14
2. Lalaoui N, Boyden SE, et al. Mutations that prevent caspase cleavage of RIPK1 cause autoinflammatory disease. Nature. 2020 Jan 2;577(7788):103–8
3. Toskov V, Ehl S. Autoimmune lymphoproliferative immunodeficiencies (ALPID) in childhood: breakdown of immune homeostasis and immune dysregulation. Mol Cell Pediatr. 2023 Sep 13;10(1):11

### Autoinflammatory diseases

#### P055 A prospective cohort of children with Kawasaki disease from a South African children’s hospital

##### Claire Butters, Zainad Karaan, Kate Webb

###### Paediatrics, University of Cape Town, Cape Town, South Africa

####### **Correspondence:** Claire Butters

*Pediatric Rheumatology 2024*, **22(2):**PReS24-ABS-1288

**Introduction:** Kawasaki Disease (KD) is rarely described in Africa with scattered case reports giving an impression of rarity on the continent.

**Objectives:** Describe the clinical phenotype and frequency of KD cases in a tertiary level, public, children’s hospital in Cape Town, South Africa.

**Methods:** A prospective cohort of children diagnosed with KD as per AHA 2017 criteria was recruited with consent from June 2020 until February 2024 from the Red Cross War Memorial Children’s Hospital in Cape Town, South Africa. Children recruited prior to March 2022 were required to be negative for SARS-CoV-2 antibodies in order to distinguish from MIS-C; after March 2022, this requirement was discarded. Demographic and summary clinical data were collected from all children recruited.

**Results:** Thirty-three children were recruited during the 46-month period with a median age of 1,82 years old (minimum 3 months, maximum 11 years). One was excluded due to insufficient clinical data. Sixteen (50%) were black, 15 (47%) were mixed race and one (13%) was white reflecting the demographics of the city. Seventeen (47%) were male. Eight children had comorbidities - 2 were perinatally exposed to HIV (uninfected), 2 exposed to TB, 1 had clubfoot, 1 failed to thrive and 1 had malnutrition. During admission, 30/32 had a rash, 29/32 had conjunctivitis, 18/32 had diarrhoea, 13/32 had arthritis, 8/32 had lung involvement and 2/32 had CNS symptoms. One child developed macrophage activation syndrome and two children developed coronary artery aneurysms. Maximum CRP, white cell count, neutrophil count, LDH, AST, ferritin, D-dimer and fibrinogen during admission were all higher than the normal range determined by the local laboratory. Minimum haemoglobin and sodium during admission were lower than the normal range. The average ejection fraction was 66%. One child required ICU admission for ionotropic blood pressure support and one child required ventilation. Twenty (63%) received IV antibiotics. All but 3 children received IVIG, and 4 children required a second IVIG infusion. Eleven (34%) children additionally received IV methylprednisolone (median total dose 30mg/kg). No children died and the median duration of admission was 6 days.

**Conclusion:** KD is not uncommon at this institute. In the author’s opinion, a lack of reports of KD in Africa likely reflects lack of diagnosis and access to care rather than true disease scarcity in the region. The clinical phenotype and response to treatment in this small cohort is similar to others although larger, collaborative registries and cohorts in the region are required to confirm this.

**Date of birth::** mai 07, YY

**Patient Consent**

Not applicable (there are no patient data)

**Disclosure of Interest**

None Declared

### Autoinflammatory diseases

#### P056 The use of steroids in PFAPA syndrome and surf: preliminary results from the JIR-CliPS survey

##### Ezgi D. Batu^1^, Seher Sener^1^, Mariana Rodrigues^2^, Caroline Vinit^3, 4^, Francois Hofer^5^, Katerina Laskari^6^, Ricardo Craveiro Costa^7^, Margarida Faria^8^, Natasa Toplak^9^, Marco Gattorno ^10^, Michaël Hofer^11^

###### ^1^Pediatric Rheumatology, Hacettepe University, Ankara, Türkiye; ^2^Pediatric Rheumatology and Young Adult Unit, Centro Hospitalar Universitário São João; Porto Medical School, Porto, Portugal; ^3^General Pediatrics, Jean Verdier Hospital, Bondy; ^4^Paediatric Rheumatology, Robert Debre Hospital, Paris, France; ^5^France, Fondation RES, Lausanne, Switzerland; ^6^Propaedeutic Internal Medicine, National and Kapodistrian University of Athens, Athens, Greece; ^7^Hospital Pediátrico, Unidade Local de Saúde de Coimbra, Coimbra; ^8^Rheumatology, Hospital Central do Funchal, Funchal, Portugal; ^9^Dept. of Allergology, Rheumatology and Clin. Immunology, UCH, UMC, Faculty of medicine , Ljubljana, Slovenia; ^10^Rheumatolgy and Autoinflammatory Diseases, IRCCS Istituto Giannina Gaslini, Genoa, Italy; ^11^Pediatric Immunology, Allergology, and Rheumatology, Lausanne University Hospital, Lausanne, Switzerland

####### **Correspondence:** Seher Sener

*Pediatric Rheumatology 2024*, **22(2):**PReS24-ABS-1349

**Introduction:** PFAPA syndrome is the most common periodic fever syndrome during childhood. The number of patients with SURF (syndrome of undifferentiated recurrent fever) is also increasing despite the improvements in genetic diagnosis of autoinflammatory diseases. Single dose steroids are used in the treatment of these patients to abort the disease flares.

**Objectives:** We aimed to obtain a global overview of real-life steroid usage strategies among physicians treating PFAPA and SURF patients.

**Methods:** The JIR-CliPS PFAPA and SURF online questionnaires, consisting of 15 questions focused on steroid use, were distributed via e-email to potential respondents. Data analysis was performed using descriptive statistics.

**Results:** From 34 countries, 81 physicians (56 female/24 male) responded to the survey. Of those, 50 (62%) had experience with PFAPA and/or SURF patients for >10 years, and 49 (60%) were pediatric rheumatologists. 76 (93.8%) respondents mentioned that they prescribe steroid treatment at flare onset in PFAPA patients, while 54 (66.7%) prescribe steroids to SURF patients. Prednisolone at a dose of 1 mg/kg was the most frequently preferred steroid (n=37; 45.7%), followed by 1 mg/kg prednisone (n=31; 38.3%). While 25 (30.9%) respondents reported “routinely” prescribing steroids to PFAPA patients, only 12 (14.8%) indicated doing the same for SURF patients. Regarding the number of doses per flare, most respondents (54 [66.7%] for PFAPA and 59 [72.8%] for SURF) preferred 1 or 2 doses of steroids depending on the response. The most frequent maximum uses recommended per year were 5 to 10 doses (n=36; 44.4%). The definition of response to a steroid dose was indicated as “response within 12 hours” by the highest number of respondents for both PFAPA and SURF (n=37, 45.7% and n=34, 42%, respectively). For both PFAPA and SURF, most physicians consider that the patient does not respond to steroids if the patient needs >2 doses of steroids per flare and no improvement of fever within 24 hours after steroid dose. When steroids cause an increase in attack frequency, most of the respondents consider another treatment if this causes a decrease in quality of life (n=52 [64.2%] for PFAPA and n=41 [50.6%] for SURF).

**Conclusion:** The results of this survey show that physicians use steroids quite frequently for PFAPA and SURF patients in their routine clinical practice. Regarding treatment modification, the quality of life was a prominent consideration for physicians.

**Date of birth::** octobre 09

**Funding**

This project is funded as a COST (European Cooperation for Science and Technology) action CA21168.

**Patient Consent**

Yes, I received consent

**Disclosure of Interest**

None Declared

### Autoinflammatory diseases

#### P057 Markers of acute phase (CRP, SAA) in patients with autoinflammatory diseases with inactive clinical disease status

##### Alina Sukhanina, Svetlana Salugina, Eugeni Fedorov, Irina Nikishina and Torgashina A.V., Avdeeva A.S., Diatroptov M.E.

###### V. A. Nasonova Research Institute of Rheumatology, Moscow, Russian Federation

####### **Correspondence:** Irina Nikishina

*Pediatric Rheumatology 2024*, **22(2):**PReS24-ABS-1370

**Introduction:** SAA is the most sensitive marker of subclinical inflammation in the interictal period. Subclinical inflammation may persist during long-term clinical remission. Constant high concentration of SAA is one of the factors in the development of AA amyloidosis.

**Objectives:** to determine the role of inflammatory markers (C-reactive protein (CRP), serum amyloid A (SAA) in patients with major monogenic autoinflammatory diseases (mAID) and adult-onset Still’s disease (ASD) with inactive clinical disease status.

**Methods:** the study included 39 patients with mAID (22 men and 17 women) observed at the V.A. Nasonova Research Institute of Rheumatology, Moscow, Russia. Among them is family Mediterranean fever (FMF) - 10, cryopyrin-associated periodic syndromes (CAPS) - 11, TRAPS - 8, ASD - 10. The age of the pts ranged from 2 to 64 years (me 17 [12;40] years). The median duration of the disease was 7 [3;14] years (from 1 month up to 59 years). Levels SAA and CRP were determined for all pts by using the immunonephelometry. Reference values for CRP and SAA were 0-5 mg/l and 0-6,4 mg/l respectively.

**Results:** all pts had no clinical signs of the disease (fever, rash, etc.). 14 of them received therapy with colchicine (FMF-9, CAPS-3, TRAPS-2), 3 of them received anakinra (all patients with CAPS), 19 - canakinumab, among them were 9 CAPS, 6 TRAPS, 3 FMF and 1 ASD. An increase level of CRP (Me 2.1 [0.88;8.5 mg/l]) was detected in 15 (38%) pts, in pts with CAPS - 5 (45%), (Me 4.8 [1.1;11.4]), ASD - 5 (50%), (Me 4 [1.2;8.5]), FMF - 3 (30%), (Me 1.05 [0.3;15.3]), TRAPS - 2 (25%), (Me 2.45 [0 ,3;5,1]). An increase level of SAA (Me 4.64 [2;27] mg/l) was detected in 19 (49%), in pts with ASD – 9 (90%), (Me 22.35 [11.9;62.3]), FMF - 4 (45%), (Me 3.42 [1.9;7]), CAPS - 5 (45%), (Me 3.63[1.75;27.2]) and TRAPS – 1 (12.5%), (Me 2.8 [1.6;4.2]). There were no statistically significant differences between the groups. Among 24 patients with normal CRP levels, 8 (33.3%) had elevated SAA levels, 4 of these pts had ASD, 2 had FMF, 2 had CAPS.

**Conclusion:** An increase levels of CRP and SAA in patients with inactive clinical status of the disease was detected in 38% and 49% of patients, furthermore significantly more often SAA was found, which reflects the persistence of subclinical activity of the disease. A third of patients with normal CRP levels showed an increase SAA level, which indicates the necessity of using both markers (CRP and SAA) during dynamic monitoring.

**Patient Consent**

Yes, I received consent

**Disclosure of Interest**

None Declared

### Autoinflammatory diseases

#### P058 Symptom duration in familial Mediterranean fever – never longer than 72 hours? A review of protracted febrile myalgia syndrome in children

##### Toni Hospach^1^, Kristina Rücklová^1^, Friederike Blankenburg^1^, Anita Heinkele^1^, Tilo Kallinich^2^

###### ^1^Klinikum Stuttgart, Stuttgart; ^2^Charité Berlin, Berlin, Germany

####### **Correspondence:** Toni Hospach

*Pediatric Rheumatology 2024*, **22(2):**PReS24-ABS-1045

**Introduction:** Protracted febrile myalgia syndrome (PFMS) is a rare manifestation of familial mediterranean fever (FMF). It is characterized by severe muscle pain, fever and elevated inflammatory markers lasting for up to several weeks. It may be accompanied by abdominal pain, diarrhea or a rash mimicking an IgA vasculitis. The PFMS may last up to several weeks. As the hallmark of FMF are short (maximum 3 day long) episodes of disease symptoms, the long duration of PFMS may lead to a delayed diagnosis especially in patients where PFMS was the first manifestation of FMF. A timely diagnosis is important to relieve the patients’ pain and to avoid unnecessary diagnostic procedures.

**Objectives:** 1.To raise awareness of PFMS using our own case. 2. To review the literature on clinical features with focus on primary manifestation of FMF and review treatment options. 3. To scrutinize standard textbooks of rheumatology for information on PFMS.

**Methods:** Presentation of a case report of a 6 year old girl who presented with PFMS and was diagnosed only after undergoing multiple diagnostic procedures to exclude alternative diagnoses. Systematic literature review in PubMed using the keywords “protracted febrile myalgia”. Search for information on PFMS in seven standard textbooks of rheumatology.

**Results:** The presented 6 year old patient manifested with fever, disabling myalgias, abdominal pain and petechial rash on the dorsal side of the feet lasting over 6 weeks. Her inflammatory markers were high. FMF was only suspected after the parents mentioned a positive family history for FMF and the genetic testing revealed a homozygosity for M694V pathogenic variant in the MEFV gene.

Based on the PubMed search 18 articles with a total of 77 pediatric patients (excluding our own report) were retrieved. The publication date ranged from 2000-2024; the number of the patients in the case series was low, ranging from 1-14. More than half (42/77, 55%) of the patients presented with PFMS as the first manifestation of FMF and 8 (10%) were afebrile. Corticosteroids (CS) were reported to be effective in 59 out of 77 patients (77%), in CS-refractory cases anakinra was successfully given in 5 patients.

The scrutiny of seven textbooks of rheumatology showed that PFMS presenting with severe myalgia was mentioned in six, abdominal pain, diarrhea and rash was mentioned only once. The possible extremely long duration of symptoms exceeding several weeks was clearly described only in 2 books. The efficacy of treatment with corticosteroids was addressed in three books, with anakinra only in one.

**Conclusion:** PFMS represents a rare but severe manifestation of FMF with a plethora of symptoms. The long duration of symptoms may be misleading to clinicians especially in cases when PFMS occurs as the first manifestation of FMF. Hence, PFMS should be discussed in more detail in standard textbooks of pediatric rheumatology, especially mentioning that episodes with fever may last for several weeks. Genetic testing should be considered early in the course of suspected autoinflammatory diseases even when the symptoms do not correspond to the typical well known clinical picture.

**Patient Consent**

Yes, I received consent

**Disclosure of Interest**

None Declared

### Autoinflammatory diseases

#### P059 Late-onset mevalonate kinase deficiency with non-erosive, osteoproliferative arthritis: a case report

##### Takako Miyamae^1,2^, Tomohiro Kawabe^1,2^, Atsuo Taniguchi^3^

###### ^1^Department of Pediatric Rheumatology, Institute of Rheumatology, Tokyo Women’s Medical University Hospital; ^2^Division of Rheumatology, Department of Internal Medicine, Tokyo Women’s Medical University School of Medicine, Tokyo; ^3^Division of Rheumatology, Fukujuji Hospital, Kiyose, Japan

####### **Correspondence:** Takako Miyamae

*Pediatric Rheumatology 2024*, **22(2):**PReS24-ABS-1142

**Introduction:** Mevalonate kinase deficiency (MKD) arises from loss-of-function mutations in MVK, precipitating mevalonate accumulation. The inflammatory episodes typically emerge in early childhood, often by the age of 6 months, notwithstanding several years of diagnostic delay. This clinically presents as pyrexia and may be accompanied by gastrointestinal manifestations, lymphadenopathy, aphthous ulcers, and arthralgia/arthritis. The residual level of mevalonate kinase activity is postulated to be the underlying determinant of phenotypic heterogeneity in MKD. A spectrum of clinical manifestations has been documented. Nonetheless, the comprehensive scope of the malady remains incompletely elucidated.

**Objectives:** To describe a case entailing a patient exhibiting non-erosive arthritis with prominent osseous hyperplasia since the age of 13 years, later progressing into febrile paroxysms after the age of 20 years, ultimately leading to the diagnosis of MKD at 57 years of age.

**Methods:** A case report

**Results:** A 13-year-old boy exhibited robust health aside from a history of laparotomy for acute abdominal distress at ages 6 and 8, respectively. He presented with painful, swollen ankle and knee joints that required periodic synovial punctures but gradually diminished after the age of 16. However, at 23, he became cognizant of wrist arthralgia accompanied by periodic fevers ranging from 39 to 40°C. Recurrent fever paroxysms recurred every three months, lasting 7 to 14 days. Subsequently, the frequency of fever attacks escalated to monthly. C-reactive protein (CRP) levels persisted elevated irrespective of fever presence or absence. In the patient’s 20s, deformities of the phalanges and flexion contractures in elbows and knees had manifested. Joint radiographs revealed no evidence of bone erosion but exhibited pronounced bone proliferation. No periarticular osseous sclerosis or heterogeneous joint space narrowing characteristic of osteoarthritis was noted. Methotrexate demonstrated ineffectiveness in addressing both joint manifestations and fever, while adalimumab alleviated fever with a decrease in the level of CRP from 100-120 to 10-50 mg/L. Genetic analysis unveiled a homozygous mutation (c.976G>A; p.Gly326Arg) in the MVK gene, deemed pathogenic. The diagnosis of MKD was confirmed by elevated urinary mevalonate levels (6.7 μg/mgCr, normal control 0.24±0.24) by LC-MS/MS. His parents were consanguineous, being second cousins. The patient is slated to undergo canakinumab therapy against still enduring chronic inflammation.

**Conclusion:** An instance of MVK characterized by joint manifestations in adolescence and notable febrile episodes in adulthood, which we perceive as components of the as-yet incompletely understood clinical phenotype of MVK, was presented. Despite limited reports of joint involvements associated with MVK, this case clearly illustrated the non-destructive nature of the condition and marked bone proliferation.

**Patient Consent**

Yes, I received consent

**Disclosure of Interest**

T. Miyamae Speaker Bureau with: Novartis Pharma K.K., T. Kawabe: None Declared, A. Taniguchi: None Declared

### Autoinflammatory diseases

#### P060 IL-1 inhibitors in the treatment of familial Mediterranean fever – the experience of the federal center

##### Svetlana Salugina, Evgeny Fedorov, Anna Torgashina, Irina Nikishina, Maria Kaleda

###### Pediatrics, V. A. Nasonova Research Institute of Rheumatology, Moscow, Russian Federation

####### **Correspondence:** Irina Nikishina

*Pediatric Rheumatology 2024*, **22(2):**PReS24-ABS-1150

**Introduction:** Familial Mediterranean Fever (FMF) is one of the most common monogenic autoinflammatory disease (mAID). Main manifestations are recurrent attacks of fever, polyserositis, erysipeloid rash, arthritis, increased levels of acute phase markers (ESR, CRP, SAA). The risk of developing amyloidosis is up to 60% in untreated patients. Colchicine is still the mainstay of FMF treatment. If patients do not respond to the maximal tolerated dose (colchicine-resistance) or intolerant to the drug due to its side effects IL-1 inhibitors (iIL-1) (anakinra, canakinumab) are recommended as second-line therapy. Other biologics (B) can also be used for individual indications.

**Objectives:** to present the experience of using iIL-1 in patients with colchicine-resistant FMF according to the Federal Rheumatology Center.

**Methods:** from 2016 to 2023, 18 patients (11 males) receiving iIL-1 (anakinra, canakinumab) were included in the study. The median age of patients at the start of therapy was 10 [5;17.5] years, range from 4.5 to 47 years. Anakinra was prescribed at a dose of 1-4mg/kg or 100mg daily, subcutaneously, canakinumab – 2mg / kg or 150 mg every 4 weeks subcutaneously.

**Results:** Among 112 patients with FMF, colchicine -108 (96.4%) were treated, unsatisfactory drug tolerance of the full dose was noted in 6 (5.6%), colchicine-resistance in 18 (16.7%). B was prescribed in 24 patients (21.4%). iIL-1 received 18 patients (16.1%): canakinumab - 15 (13.4%), anakinra - 3 (2.8%). TNF inhibitors received 10 patients: etanercept - 6 (5.4%), adalimumab - 4(3.6%). 1 patient received tocilizumab (0.9%), 1 - secukinumab (0.9%). Before the start of IL-1 therapy all patients had fever, peritoneal and pleural manifestations were in 17 (94.4%) and in 12 (66.7%) accordingly, skin involvement - 13 (72.2%), joints involvement - 14 (77.8%), all patients had elevated acute phase reactants. Amyloidosis was diagnosed in 2 (1.8%). The duration of taking iIL-1 is from 2 months to 6 years. A complete response to il-1 therapy was obtained in 12 (66.7%), partial response was in 1 (5.6%).

**Conclusion:** iIL-1 are a good therapeutic option in colchicine-resistant patients or in case of intolerance to colchicine. Canakinumab was more often used in rheumatologist’s practice. Most patients have a complete response to therapy. The tolerability of therapy is satisfactory.

**Patient Consent**

Yes, I received consent

**Disclosure of Interest**

None Declared

### Autoinflammatory diseases

#### P061 A novel homozygous variant in CD3E gene causing Omenn syndrome

##### Jumanah Nassar^1^, Mohammed Nashawi^2,3^, Saddiq Habiballah^2,3^

###### ^1^Medicine, Batterjee Medical College; ^2^Department of Pediatrics, King Abdulaziz University, Faculty of Medicine; ^3^Immunology Unit, King Abdulaziz University, King Fahad Medical Research Center, Jeddah, Saudi Arabia

####### **Correspondence:** Jumanah Nassar

*Pediatric Rheumatology 2024*, **22(2):**PReS24-ABS-1211

**Introduction:** Omenn syndrome (OS) is a known clinical phenotype for several form of severe combined immunodeficiency (SCID), characterized by certain features such as scaly erythematous rash, chronic diarrhea, recurrent infections, lymphadenopathy, hepatosplenomegaly, and failure to thrive, with RAG-1/RAG-2 being the most common genes associated with this syndrome. (1)

**Objectives:** We present an infant with clinical and laboratory features of Omenn syndrome who genetically confirmed to have a novel homozygous variant in CD3E gene.

**Methods:** Case report

**Results:** A girl infant presented to our hospital at the age of 3 months, showing symptoms such as erythroderma, fever, alopecia universalis, lymphadenopathy, and splenomegaly. Laboratory workup showed significant lymphocytosis, eosinophilia, elevated IgE level, and high CRP level. Flow cytometry showed normal CD3+, CD4+, CD8+, and CD19+ counts, with elevated CD56+ counts. Omenn syndrome was suspected, and she was started on immunoglobulin replacement therapy as well as antibiotics and antifungal prophylaxis. Ultrasound confirmed the absence of the thymus, and genetic testing revealed a novel homozygous missense variant of the CD3E gene at p.GLy94Ala. The patient is currently undergoing evaluation for bone marrow transplant.

**Conclusion:** Although CD3E mutations are known to cause T-B+NK+ SCID (2), they have not been reported to cause Omenn syndrome. To our knowledge, this is the first reported case of CD3E monogenic disease-causing variant presenting as Ommen syndrome.

**Patient Consent**

Yes, I received consent

**Disclosure of Interest**

None Declared

**References**

1. Dvorak CC, Haddad E, Heimall J, Dunn E, Buckley RH, Kohn DB, Cowan MJ, Pai SY, Griffith LM, Cuvelier GDE, Eissa H, Shah AJ, O’Reilly RJ, Pulsipher MA, Wright NAM, Abraham RS, Satter LF, Notarangelo LD, Puck JM. The diagnosis of severe combined immunodeficiency (SCID): The Primary Immune Deficiency Treatment Consortium (PIDTC) 2022 Definitions. J Allergy Clin Immunol. 2023 Feb;151(2):539-546. 10.1016/j.jaci.2022.10.022. Epub 2022 Nov 28. PMID: 36456361; PMCID: PMC9905311.
2. Erman B, Fırtına S, Fışgın T, Bozkurt C, Çipe FE. Biallelic Form of a Known CD3E Mutation in a Patient with Severe Combined Immunodeficiency. J Clin Immunol. 2020 Apr;40(3):539-542. 10.1007/s10875-020-00752-3. Epub 2020 Feb 4. PMID: 32016651.

### Autoinflammatory diseases

#### P062 E148Q mutation: a disease-causing mutation or a sequence variant? Disease severity in specific ethnic groups of FMF patients

##### Mohamad Hamad Saied^1,2,3^, Joeri van Straalen^3^, Elham Orouk Awaad^1^, Tal Gazitt^4,5^, Adi Miller-Barmak^6^

###### ^1^Department of Pediatrics, Carmel Medical Center; ^2^Faculty of Medicine, Technion - Israel Institute of Technology, Haifa, Israel; ^3^Department of Pediatric Immunology and Rheumatology, Wilhelmina Children’s Hospital, University Medical Center Utrecht, Utrecht, Utrecht, Netherlands; ^4^Department of Medicine, University of Washington Medical Center, Seattle, Washington, United States; ^5^Rheumatology Unit, Carmel Medical Center; ^6^Technion - Israel Institute of Technology, Haifa, Israel

####### **Correspondence:** Mohamad Hamad Saied

*Pediatric Rheumatology 2024*, **22(2):**PReS24-ABS-1223

**Introduction:** Currently, in the field of FMF research, controversy exists as to whether the E148Q mutation is a benign or disease-causing polymorphism.

**Objectives:** To evaluate clinical characteristics and disease severity in specific ethnic groups of FMF patients homozygous for the E148Q mutation, and to examine the contribution of a single allele V726A mutation to disease severity.

**Methods:** A retrospective cohort study included patients homozygous for the E148Q mutation. Demographic, clinical, and laboratory data were extracted from electronic medical records of the Clalit Health Services (CHS)-affiliated Carmel Medical Center in Haifa, Israel. The Characteristics of FMF patients with E148Q/E148Q and E148Q/E148Q + V726A/- MEFV mutations were compared. The primary outcome measure was FMF disease severity as measured by Tel Hashomer Key to Severity Score among patients homozygous for the E148Q mutation alone or homozygous for the E148Q mutation along with one mutation for the V726A allele.

**Results:** A total of 61 FMF patients were included of which 24 (39%) had the E148Q/E148Q MEFV mutation and 37 (61%) had the E148Q/E148Q + V726A/- MEFV mutation. Patients with the E148Q/E148Q + V726A/- MEVF mutation were significantly more often of Druze descent compared to patients with the E148Q/E148Q MEFV mutation alone. Other characteristics did not differ significantly between the two groups. For both groups, the majority of patients had mild or moderate disease severity. CRP levels decreased significantly after colchicine treatment.

**Conclusion:** Patients with the E148Q mutation alone present with mild to moderate disease severity in all ethnic groups of Israeli FMF patients. Disease severity did not differ between patients with E148Q/E148Q+ V726A/- MEFV mutation and E148Q/E148Q mutation alone. Colchicine treatment should be given.

**Date of birth:** mars 19, Y

**Patient Consent**

No, I have not received consent

**Disclosure of Interest**

None Declared

### Autoinflammatory diseases

#### P063 A family case of Blau syndrome in three siblings: a different course of the disease

##### Yury M. Spivakovskiy^1^, Anna Y. Spivakovskaya^1^, Olesja V. Volkova^1^, Irina P. Nikishina^2^

###### ^1^Department of Faculty Pediatrics, State Medical University n.a. Razumovskiy, Saratov; ^2^Pediatrics, V.A. Nasonova Research Institute of Rheumatology, Moscow, Russian Federation

####### **Correspondence:** Irina P. Nikishina

*Pediatric Rheumatology 2024*, **22(2):**PReS24-ABS-1441

**Introduction:** Autoinflammatory diseases continue to attract the close attention of the medical community. One of these diseases is Blau syndrome (SB), a rare autosomal dominant disease manifested by arthritis, uveitis, dermatitis and early onset.

**Objectives:** To present various clinical variants of the course of Blau syndrome (genetically verified variant) children from the same family.

**Methods:** The analysis of medical documentation and objective observation of three patients from the same family for 6 to 14.5 years was carried out with an assessment of the dynamics of the course of the disease and the effectiveness of therapy.

**Results:** There are 3 children in the family of clinically healthy parents, all boys. The middle child was the first to come to the rheumatologist’s attention when he was about 5 years old. He has been ill since the age of 2, was observed by a traumatologist and a pediatrician about “hygrom”, under the brand of which bogge arthritis was occurring. Initially, the diagnosis of polyarticular JIA was established. The absence of a significant effect on methotrexate (MTX) required the appointment of cyclosporine. Subsequently, etanercept therapy was initiated with a moderate positive effect, but intercurrent infections determined recurrent exacerbations, while stabilization was possible only with the use of systemic corticosteroids. Genetic verification of the diagnosis of SB at the age of 10 years. The instability of the condition required a further change of GIBP to golimumab, and subsequently to rituximab, against which a relative stabilization of the patient’s condition was achieved. During all the years of observation, the articular syndrome was the leading one in the clinic, eye damage was registered after 17 years, and the phenomena of dermatitis were minimal.

The eldest child was examined by a rheumatologist, solely in connection with the observation of the average at the age of 10 years. According to the mother, some swelling of the wrist joints was noted at the age of 2 years, but they did not go to the doctor, the symptoms were stopped on their own. Under the supervision of a rheumatologist, deformity of the small joints of the hand was revealed. Functional disorders and biochemical activity were not observed during the entire observation period. Since the age of 12, he has been receiving MTX as basic therapy. There is no progression of the disease.

The youngest child, taking into account the clinic of the elders, was observed from birth. The onset of symptoms from the age of 2. There is a steady progression of articular syndrome and uveitis, and the appearance of dermatitis from the age of 5. As therapy, he received adalimumab with insufficient effect and replacement with tofacitinib.

**Conclusion:** The existing joint symptoms, with their resistance to ongoing therapy and especially in patients with family ties, require mandatory inclusion in the examination plan of genetic studies. The present clinical presentation demonstrates that even with the same genetics, the effect of treatment is largely determined by the phenotypic characteristics of each individual patient.

**Patient Consent**

Yes, I received consent

**Disclosure of Interest**

None Declared

### Autoinflammatory diseases

#### P064 Emergence of colchicine resistance in pediatric familial Mediterranean fever: a harbinger of severe enterocolitis

##### Paivi Miettunen^1,2^, Jacky Chow^3^, Aidan Stephenson^1^, Sanjay Sethi^3^

###### ^1^Pediatric Rheumatology, Alberta Children’s Hospital; ^2^Pediatrics; ^3^Radiology, University of Calgary, Calgary, Canada

####### **Correspondence**: Paivi Miettunen

*Pediatric Rheumatology 2024*, **22(2):**PReS24-ABS-1788

**Introduction:** Inflammatory bowel disease unclassified (IBDU) is characterized by features of IBD that are difficult to classify either as ulcerative colitis (UC) or Crohn’s disease (CD). IBDU has been reported in patients with MEFV gene mutation, with suggested name “*MEFV*-gene-related enterocolitis”, within the classification of IBDU^1^. Additionally, FMF causes abdominal attacks due to serositis, but also gastrointestinal involvement with similar clinical presentation to IBD. The clinical characteristics of *MEFV -*gene-related enterocolitis remain unclear.

**Objectives:** We report a pediatric FMF patient who developed enterocolitis while on colchicine.

**Methods: Case:** A 17-year-old male, with 2 known variants in the *MEFV* gene (C.818C and C.549G), in remission on colchicine for 11 years, presented to rheumatology clinic with a 6-month history of “break through” FMF episodes. Despite optimizing colchicine to 2.4 mg/day, he was having monthly episodes of sore throat, fever, headache, abdominal pain and nausea. Episodes became gradually longer (7-8 days), associated with night sweats and weight loss, but complete symptom resolution between the episodes. He developed a prolonged FMF episode (10 days), with severe abdominal pain, nausea and weight loss due to inadequate oral intake. He was started on oral prednisone 20 mg/day. He was admitted to hospital 3 days later because of ongoing abdominal symptomatology and intolerance of oral medications. Physical examination was unremarkable apart from a 5-kg weight loss in 7 days, and dry mucous membranes. His abdominal exam was benign. His temperature was 36.6 Celcius. Three days prior to his admission CRP was 81.2 mg/L (Normal (N) < 8.0 mg/L), with WBC 11.8 (N 4.5-13.0 (10^9^/L)), Hgb 156 (N 125-170 (g/L)) and Platelets 203 (N 140-400 (10^9^/L)).

He had blood tests, infectious (blood/enteric) workup, H pylori antigen and abdominal ultrasound (US).

**Results:** On admission, his CRP was 12.3 mg/L, with normal WBC, Hgb and Platelets. ESR was 12 (N < 10 mm/hr). Albumin, liver enzymes, and creatinine were all normal. H pylori antigen was negative as was infectious workup, including tuberculosis, hepatitis, blood and enteric pathogens. Bowel ultrasound revealed diffuse wall thickening of the entire colon associated with hyperemia, suggestive of colitis “likely infective or inflammatory”. He received oral ondansetron, IV methylprednisolone 60 mg x 1, followed by oral prednisone 20 mg/day and ongoing colchicine. He was started on Anakinra 100 mg SC once daily, with rapid resolution of his symptoms. His prednisone was discontinued within 9 days, and he remains on colchicine 1.2 mg/day with anakinra 100 mg SC. He remains asymptomatic and has regained his weight.

**Conclusion:** In this pediatric FMF patient, loss of symptom control with colchicine, gradual prolongation of FMF episodes and worsening abdominal symptomatology heralded the diagnosis of FMF enterocolitis. Colchicine malabsorption was ruled out (negative H. pylori). Because his abdominal symptoms resolved on anakinra, IBD was felt to be unlikely. Our case adds to the emerging literature of FMF with enterocolitis, and illustrates that enterocolitis can emerge despite previously well controlled FMF and that anakinra can be helpful in colchicine resistant colitis management.

**Patient Consent**

Yes, I received consent

**Disclosure of Interest**

None Declared

**Reference**

1. Y. Yokoyama, T. Yamakawa, T. Ichimiya, et al. *“Gastrointestinal involvement in a patient with familial Mediterranean fever mimicking Crohn’s disease: a case report.”* Clinical Journal of Gastroenterology (2021) 14: 1103-1107.

### Autoinflammatory diseases

#### P065 Behçet’s disease in pediatrics, a diagnostic challenge: qualitative systematic review of the literature

##### Nathalie Yepes^1^, María D. P. Gómez^2^, José F. Gomez^1^

###### ^1^Pediatrics; ^2^Pediatric Rheumatology, Universidad Libre, Seccional Cali, Cali, Colombia

####### **Correspondence:** Nathalie Yepes

*Pediatric Rheumatology 2024*, **22(2):**PReS24-ABS-1021

**Introduction:** Behçet’s disease (BD) is a systemic inflammatory disease that mainly affects the oral and genital mucosa, skin and eyes.

**Objectives:** A qualitative systematic review of the literature is carried out in search of answering the following questions related to Behçet’s disease in the pediatric population:

1. What is the global and local epidemiology of this pathology?
2. What is known about its etiopathogenesis?
3. What is the spectrum of clinical manifestations?
4. What is the diagnostic approach to this pathology?
5. What is the treatment approach and what are the current Clinical Trials for this pathology?

**Methods:** A qualitative systematic review of the literature is carried out in search of finding the epidemiology, etiopathogenesis, spectrum of clinical manifestations, diagnosis, treatment approach and current Clinical Trials of Behçet’s disease in the pediatric population. A bibliographic search was performed in PubMed without language or publication date restrictions. References of included articles were examined for additional relevant literature

**Results:** The initial search yielded a total of 570 studies from PubMed, 1 from a website, 4 from the Colombian Ministry of Health and 4 from a review of bibliographic citations, of which 32 articles were included used for the present review, finding that the prevalence At a global level it is estimated around 10.3 per 100,000 inhabitants. BD is a vasculitis that affects vessels of all sizes including veins. It was recently reclassified as variable type vasculitis. Other frequent clinical manifestations are joint, skin and digestive manifestations. Although its etiopathogenesis is not clear, in recent years it has been considered a multicausal autoinflammatory entity. Its diagnosis is clinical, without diagnostic tests. Management should be individualized based on the manifestations of the disease given the clinical variability.

**Conclusion:** Multicenter, placebo-controlled, standardized studies that involve large series of patients, use clinical scores, and have long-term follow-up are needed to better understand the nature of this disease.

**Date of birth::** novembre 0

**Patient Consent**

Not applicable (there are no patient data)

**Disclosure of Interest**

None Declared

**References**

1. Tiago Santos Trindade, Catarina Granjo Morais, Ana Maia, Mariana Rodrigues, Iva Brito. Demographic and clinical features of pediatric vasculitis: a single-center study. The official Journal of the Portuguese Society of Rheumatology [Internet]. 2023;2(1):30–40. Available from: www.arprheumatology.com
2. Scherrer MAR, Rocha VB, Garcia LC. Behçet’s disease: Review with emphasis on dermatological aspects. Vol. 92, Anais Brasileiros de Dermatologia. Sociedade Brasileira de Dermatologia; 2017. p. 452–64.
3. Yildiz M, Haslak F, Adrovic A, Sahin S, Koker O, Barut K, et al. Pediatric Behçet’s Disease. Vol. 8, Frontiers in Medicine. Frontiers Media S.A.; 2021.
4. Behçet’sUK. Behçet’s UK. 2023 [cited 2023 Aug 9]. p. 1–3 Behçet’s Factsheet 13: In Children. Available from: https://behcetsuk.org/wp-content/uploads/2019/04/13.-Behcets-In-Children-v2.0-Jan18.pdf.

### Autoinflammatory diseases

#### P066 A new variant in the ADA2 gene in a 9-year-old female patient with simultaneous 22Q11 duplication

##### Leosirlay Nathaly Rojas Gomez^1,2^, Edward Camilo Vera-Parra^2,3^, Adriana Soraya Diaz-Maldonado^1,4,5,6^, Maria Fernanda Reina-Ávila^2,5^, Oscar Mauricio Espitia Segura^7,8^, Silvia Juliana Maradei Anaya^9^, Natalia Vélez-Tirado^10^

###### ^1^Pediatric Rheumatology Program, Universidad El Bosque, School of Medicine; ^2^Member of the Colombian Association of Rheumatology; ^3^Rheumatology Program, Universidad Militar Nueva Granada; ^4^Pediatric Rheumatology Program, Member of the Colombian Association of Rheumatology; ^5^Pediatric Rheumatology, HOMI, Fundación Hospital Pediatrico La Misericordia; ^6^CARE FOR KIDS; ^7^Neuropediatrics Unit, HOMI, Fundación Hospital Pediatrico La Misericordia; ^8^RICCNeP Network of Qualitative and Quantitative Research in Neuropediatrics; ^9^Genetics Unit; ^10^Pediatric Immunology and Allergy Department, HOMI, Fundación Hospital Pediatrico La Misericordia, Bogotá, Colombia

####### **Correspondence:** Leosirlay Nathaly Rojas Gomez

*Pediatric Rheumatology 2024*, **22(2):**PReS24-ABS-1399

**Introduction:** Adenosine deaminase 2 (ADA2) deficiency is an autoinflammatory disease with autosomal recessive inheritance. Studies and evaluation of ADA2 variants in different populations have led to the estimation of carrier status in 1/236 individuals with a prevalence of the disease in 1/222,000. ADA2 deficiency manifests as an autoinflammatory syndrome with systemic manifestations. The onset of symptoms usually occurs in the first decade of life. Regarding the clinical manifestations of the disease, three well-established phenotype presentation have been identified: the inflammatory/vasculitic phenotype, the hematological phenotype and finally the immune dysregulation phenotype. One third of patients present with neurological manifestations, lacunar infarctions being a typical finding of this disease.

**Objectives:** We present the case of a 9-year-old girl with a history of consanguinity in the first degree, with cognitive deficit associated with microduplication of 22q11, and a history of cerebral lacunar ischemia events that began at 7 years of age. During the study, the main vascular, cardiac, blood, neoplastic, autoimmune and genetic etiologies were discarded, so it was decided to perform whole exome sequencing and mitochondrial DNA, finding a variant in the ADA2 gene C.647G>T, p.Gly216Val, in homozygosity, of uncertain significance, seeking to functionally confirm the variant reported in ADA2, a measurement of enzymatic activity was carried out, which gave a result of 0.0 mU/g. compared to the internal control that had an activity of 94 mU/g, thus confirming the ADA2 deficiency phenotype associated with this new variant.

**Conclusion:** ADA2 deficiency is a monogenic vasculitis with three presenting phenotypes, usually debuts in children and there may be a high underreporting in the adult population. Ischemic stroke as an initial manifestation is not highly frequent, however, those patients who present it usually have a high impact on morbidity and functionality. Timely diagnosis of the disease is often challenging, therefore, performing genetic sequencing and measuring enzyme activity in a timely manner helps to achieve a timely diagnosis that allows us to initiate immunomodulatory therapy or therapies directed to the phenotype of the disease, thus reducing the risk of relapses and complications.

**Patient Consent**

Yes, I received consent

**Disclosure of Interest**

None Declared

**References**

1. Meyts I, Aksentijevich I. Deficiency of Adenosine Deaminase 2 (DADA2): Updates on the Phenotype, Genetics, Pathogenesis, and Treatment. J Clin Immunol. 2018 Jul; 38(5):569-578. 10.1007/S10875-018-0525-8.
2. Jee H, Huang Z, Baxter S, Huang Y, Taylor ML, Henderson LA, Rosenzweig S, Sharma A, Chambers EP, Hershfield MS, Zhou Q, Dedeoglu F, Aksentijevich I, Nigrovic PA, O’Donnell-Luria A, Lee PY. Comprehensive analysis of ADA2 genetic variants and estimation of carrier frequency driven by a function-based approach. J Allergy Clin Immunol. 2022 Jan; 149(1):379-387. 10.1016/j.jaci.2021.04.034.*.*

### Autoinflammatory diseases

#### P067 Effectiveness of etanercept in a patient with primary ciliary dyskinesia and TRNT1 related autoinflammatory syndrome

##### Simona Di Gennaro^1^, Francesca Della Casa^2^, Angelica Petraroli^2^, Melissa Borrelli^3^, Francesca Santamaria^3^, Maria Alessio^1^, Roberta Naddei^1^

###### ^1^Department of Translational Medical Science, Paediatric Rheumatology; ^2^Department of Translational Medical Sciences, Division of Autoimmune & Allergic Diseases; ^3^Department of Translational Medical Sciences, Paediatric Pulmonology, University of Naples Federico II, Naples, Italy

####### **Correspondence:** Simona Di Gennaro

*Pediatric Rheumatology 2024*, **22(2):**PReS24-ABS-1407

**Introduction:** TRNT1 mutations are associated to an autoinflammatory syndrome (SAID) presenting with a wide clinical heterogeneity.

**Objectives:** To describe the clinical course of a young woman with primary ciliary dyskinesia (PCD) leading to the diagnosis of TRNT1 related SAID, and to report her clinical response to Etanercept.

**Methods:** Case report.

**Results:** We report the case of a 19-year-old girl affected by PCD referred at our Centre with a history of recurrent episodes of fever and arthralgias. Her past medical history started at the age of 10 when she was diagnosed with PCD due to chronic sinusitis, recurrent pneumonia and bronchiectasis associated to compound heterozygous mutations in DNAH5 gene, so she started inhalation treatment, respiratory physiotherapy, and antibiotic prophylaxis with occasional history of increased expectorations treated with antibiotic therapy. At the age of 16, she presented recurrent episodes of fever with arthralgias and polyarthritis. She started an oral course of prednisone with clinical resolution. In the following years, she presented recurrent episodes of fever which were treated with oral courses of antibiotics, in the suspicion of exacerbations of her lung disease. However, at the age of 19, the patient newly presented fever episodes with arthralgia or arthritis. So, she was referred at our Centre. Her clinical examination was unremarkable, except for wheezing and mild facial dysmorphisms. No signs of arthritis were detected. Laboratory investigations revealed a remarkable increase of serum amyloid A and an isolated splenomegaly was found at the abdomen ultrasound. A next generation sequencing panel for SAIDs was performed, showing the homozygous pathogenetic mutation c.1246A>G (p.Lys416Glu) in the TRNT1 gene, associated with the clinical phenotype of “Congenital sideroblastic anemia with immunodeficiency, fevers, and developmental delay (SIFD)”. The immunological, ophthalmological, audiometric and cardiologic assessments resulted normal. After starting Etanercept at the weekly dose of 50 mg, the patient presented the resolution of fever and arthralgias episodes and reported an improvement in lung symptoms.

**Conclusion:** The presence of recurrent fever and joint symptoms associated with elevation of inflammatory indexes led to consider the diagnosis of an autoinflammatory syndrome, despite the delayed age at onset and the concomitant history of lung disease. Etanercept was effective, as we have already reported in other two girls affected with the same disease, sharing the pathogenetic mutation c.1246A>G (p.Lys416Glu) with the patient herein described (1). Of note, the patient also reported a significant improvement in her respiratory symptoms after initiating Etanercept.

**Patient Consent**

Yes, I received consent

**Disclosure of Interest**

None Declared

**Reference**

1. Orlando et al*, Etanercept as a successful therapy in autoinflammatory syndrome related to TRNT1 mutations: a case-based review,* Clin Rheumatol. 2021.

### Autoinflammatory diseases

#### P068 Not so “SAVI” after all! - a diagnostic and therapeutic conundrum

##### Anitha Sokay^1^, Kathy Gallagher^1^, Emma Mac Dermott^1^, Ronan Leahy^2^, Orla Killeen^1^

###### ^1^Rheumatology; ^2^Immunology, CHI at Crumlin, Dublin, Ireland

####### **Correspondence:** Anitha Sokay

*Pediatric Rheumatology 2024*, **22(2):**PReS24-ABS-1139

**Introduction:** Interferonopathies (IFN’s) are a constellation of diseases, arising primarily through antigen-independent hyperactivation of immune pathways.It is primarily due to abnormal production or signalling of type I IFNs.This condition is relatively new with the first case only recognised in 2011.To date 20 interferonopathies are recognized,with new interferonopathies being identified.

**Objectives:** We present a case of a 11-month-old with a SAVI like phenotype but unsupportive genetics.

**Methods:**

Clinical data was obtained from patient files and collaboration with subspecialities.

**Results:** Our patient presented with a severe panniculitis rash,Raynaud’s,swelling of her limbs and hepatosplenomegaly at 5 weeks of age*.*She developed significant respiratory distress.A CT Thorax revealed diffuse groundglass opacification suggestive of interstitial lung disease.She had elevated inflammatory markers(CRP 24 mg/L,ferritin 1448 ng/ml,ESR 88 mm/hr), a transaminitis(ALT 193 U/ml, AST 356 U/L).She had multiple positive auto antibodies(Table 1).Clinically SAVI was suspected as the most likely diagnosis.

Her interferon signature gene assay demonstrated exaggerated IFI 27 and IFI44L levels.A Trio exome sequencing and Inhibitor of nuclear factor kappa B gene(IKBKG)sequencing did not indicate any currently recognised interferonopathy.Further genetic analysis has been requested to explore possible novel pathogenic mutations.A reasonable clinical response was seen with corticosteroid therapy in combination with Jak Inhibition.With worsening of her cutaneous disease and an increasing need for corticosteroid use a trial of anti IL-6 therapy was initiated.This was subsequently discontinued when she presented with severe acute digital ischemia.It was managed with escalating doses of ruxolitinib, pulse high dose corticosteroids and epoprostenol infusion.

**Conclusion:** This case enhances our current understanding of interferonopathies.Although the phenotype and course of the disease is compatible with SAVI,genetic testing is negative to date.Presently there are eleven heterozygous gain-of-function (GoF) variants for SAVI.With JAK inhibition as the most successful therapeutic option to date.Our case possibly represents an as yet unreported genetic variant, further studies are awaited.

**Patient Consent**

Yes, I received consent

**Disclosure of Interest**

None Declared

**References**

1. Nigrovic et al.Monogenic autoinflammatory disorders:Overview, phenotype, clinical approach.Allergy Clin Immunol.2020 Nov;146(5):925-937.
2. Crow et al.Type I interferonopathies:10 years on. Nat Rev Immunol.2022 Aug;22(8):471-483.
3. Omoyinmi et al.STING-associated vasculitis of infancy.Arthritis Rheumatol. 2015 Mar;67(3):808.
4. Liu et al.Activated STING in vascular and pulmonary syndrome.N Engl. 2014 Aug 7;371(6):507-518.
5. Cetin et al.2021 EULAR/ACR points for diagnosis and management of type I interferonopathies:CANDLE/PRAAS, SAVI, AGS.Ann Rheum Dis. 2022 May;81(5):601-613

### Autoinflammatory diseases

#### P069 Blau syndrome with lung involvement and pulmonary hypertension: a case report

##### Maria Kaleda, Irina Nikishina, Ludmila Semenova, Anna Shapovalenko, Svetlana Salugina

###### Pediatrics, V. A. Nasonova Research Institute of Rheumatology, Moscow, Russian Federation

####### **Correspondence:** Irina Nikishina

*Pediatric Rheumatology 2024*, **22(2):**PReS24-ABS-1149

**Introduction:** Atypical symptoms are detected in 1/3-1/2 of patients with Blau syndrome (BS), including some cases of lung involvement. Animal experiments have shown that deficiency of the NOD2 gene product exacerbates hypoxia-induced pulmonary hypertension (PH).

**Objectives:** to present a rare case of BS with pulmonary granulomatosis and PH.

**Methods:** Case report.

**Results:** We reported the case of a 11y.o. boy who was referred to our clinic by a pulmonologist. He had poor exercise tolerance after 4 y.o. Diagnosis of idiopathic PH was verificated in 8 y.o, was confirmed based on Doppler echocardiography (D-EchoCG) and right-heart catheterization. The max systolic pulmonary artery pressure (sPAP) was 141 mm Hg. He began to receive sildenafil with insufficient effect. At the age 9 y.o. on chest computed tomography (СT) were presented multiple foci of ground-glass opacity in both lungs. The patient received triple PAH-specific therapy (sildenafil, macitentan, selexipag) without full disease control. He was examined for systemic diseases with lung involvement. Infections, systemic autoimmune diseases, proliferative disorders were excluded. A complete sequencing of the exome was performed at 10 y.o.: a heterozygous mutation of the NOD2 gene c.2074T>C (p.Phe692Leu) was identified. His father has this mutation too. On 1^st^ admission in our clinic (Febriary,2024) he had general fatigue, tachypnea with minimal physical activity. Physical examination: xerosis of the skin, intertriginous dermatitis; swelling of wrists, ankles, right knee. Laboratory abnormalities: Hb 102 g/l, ferritin 2.5 mkg/l, pro-BNP 155.8 pg/ml (range 0-125). US signs of synovitis of wrists, right knee. MR signs of tenosynovitis of the flexors of the III, IV fingers of the right hand. D-EchoCG: enlargement of the trunk and branches of the PA, dilation of the right-heart; sPAP 98 mmHg. Chest CT: multiple foci 9-22 mm of ground-glass opacity in both lungs and 2 isolated areas of consolidation in the right lung (S4, S5). Slit-lamp microscopy revealed no uveitis. The lung biopsy: microscopically, epithelioid-histiocytic granulomas without clear boundaries were detected in the interalveolar septa, peribronchially, perivascularly, which was regarded as granulomatous inflammation of a sarcoid-like type. He received sildenafil 60 mg/day, selexipag 2400 mg/day, macitentan 10 mg/day; torasemide 2.5 mg/day, spironolactone 37.5 mg/day. After confirmation of BS, he began taking prednisolone 15 mg/day, tofacitinib 10 mg/day with an initial effect, including decreased sPAP from 98 to 87 mm Hg, and well tolerance.

**Conclusion:** The morphologically confirmed pulmonary granulomatosis with synovitis, skin involvement and a mutation of the NOD2 gene made it possible to verify the atypical BS. The severity of interstitial lung disease doesn’t fully correlate with PH, which may indicate a mixed genesis of PH. The initial response to therapy allows us to hope for an improvement in the prognosis of our patient.

**Patient Consent**

Yes, I received consent

**Disclosure of Interest**

None Declared

### Autoinflammatory diseases

#### P070 De novo stop codon TNFAIP3 variant leading to A20 haploinsufficiency: a case of rapidly progressive ILD and fibrosis

##### Afroditi Barmpakou^1^, Senne Cuyx^1^, Helena Codes-Méndez^2^, Muthana Al Obaidi^1^, Paul Brogan^1^, Samatha Sonnappa^3^, Charalampia Papadopoulou^1^, Sandrine Compeyrot-Lacassagne^1^

###### ^1^Paediatric Rheumatology, Great Ormond Street Hospital for Children NHS Foundation Trust , London, United Kingdom; ^2^Rheumatology, Hospital de la Santa Creu i Sant Pau, Barcelona, Spain; ^3^Paediatric Respiratory Medicine, Royal Brompton Hospital, ROYAL BROMPTON & HAREFIELD NHS FOUNDATION TRUST, London, United Kingdom

####### **Correspondence:** Afroditi Barmpakou

*Pediatric Rheumatology 2024*, **22(2):**PReS24-ABS-1183

**Introduction:** A20 haploinsufficiency (HA20) is an autosomal dominant autoinflammatory disease stemming from mutations in the TNFAIP3 gene, characterized by a diverse clinical spectrum reminiscent of Behçet syndrome and other autoimmune disorders^1^. Here, we present a case report delineating severe progressive lung disease as the primary manifestation of HA20.

**Objectives:** The objective of this report is to document a unique case of HA20 presenting with rapidly progressive interstitial lung disease (ILD) and fibrosis.

**Methods:** We detail the clinical presentation and management of a 4-year-old girl of Syrian descent who developed severe acute respiratory distress syndrome (ARDS) and organized pneumonia necessitating extracorporeal membrane oxygenation (ECMO) and immunosuppressive therapy.

**Results:** The patient, previously healthy, had a recent diagnosis of tuberculosis (TB) following exposure to an adult TB case. Despite completing TB treatment, she subsequently presented with febrile lower respiratory tract infection, accompanied by a transient skin rash and hepatic abnormalities suggestive of autoimmune hepatitis. Following further investigation, including genetic testing, she was diagnosed with HA20 due to a de novo stop codon TNFAIP3 variant (p.Arg183*) .

Despite aggressive treatment with corticosteroids, mycophenolate mofetil (MMF), rituximab, and anakinra, along with supportive measures such as ECMO, the patient experienced progressive deterioration, culminating in withdrawal of life support.

**Conclusion:** This case highlights the expanding spectrum of HA20 and underscores the importance of early genetic testing in young children presenting with early onset ILD to facilitate timely diagnosis and intervention. This represents the first reported case of HA20 associated with severe progressive ILD and fibrosis in a paediatric patient, emphasizing the need for heightened clinical awareness and comprehensive evaluation in similar clinical scenarios.

**Patient Consent**

Yes, I received consent

**Disclosure of Interest**

None Declared

**Reference**

1. Zhang D, et al.Clinical characteristics and genetic analysis of A20 haploinsufficiency Pediatric Rheumatology (2021)

### Autoinflammatory diseases

#### P071 In-patient hospital stays for children with PFAPA

##### Karin Rydenman^1,2^, Stefan Berg^1,3^, Anders Fasth^1,3^, Per Wekell^1,2,3^

###### ^1^Dept of Pediatrics, Institute of Clinical Sciences, University of Gothenburg, Gothenburg; ^2^Department of Paediatrics, NU Hospital Group, Uddevalla; ^3^Department of Paediatric Rheumatology and Immunology, Queen Silvia Children’s Hospital, Gothenburg, Sweden

####### **Correspondence:** Karin Rydenman

*Pediatric Rheumatology 2024*, **22(2):**PReS24-ABS-1242

**Introduction:** Periodic fever, aphthous stomatitis, pharyngitis and cervical adenitis (PFAPA) is an autoinflammatory syndrome that causes regularly recurring fever episodes associated with symptoms depicted in the acronym. In addition, flares are often associated with abdominal pain, nausea/vomiting and myalgia/arthralgia. The onset of symptoms typically occurs before five years of age. A long diagnostic delay is common in PFAPA and the presentation of high fever in a small child may bring about an increased number of in-patient hospital stays before the diagnosis is made.

**Objectives:** To investigate the number of in-patient hospital stays for children with PFAPA before and after they are diagnosed with the condition. Further, to compare the frequency of in-patient stays between children with PFAPA and the general population in the youngest age group of 0-4 years.

**Methods:** Individual data on in-patient hospital stays were acquired from the Swedish National Patient Register for 317 patients with PFAPA born in 1997-2016 from a previously published cohort (1). The register contains all in-patient stays, linked to individuals by the Swedish personal identity numbers. Data were obtained from 1997 to July 2022. The Related Samples Wilcoxon Signed Rank test was used to compare the number of in-patient stays before and after patients received a PFAPA diagnosis. Group level data on in-patient stays for the general population in the age group 0-4 years in Region Västra Götaland was also obtained from the same register (2).

**Results:** The oldest person in the cohort was born in 1997 and the youngest 2016, resulting in a follow-up time of 6 to 25 years. The median age of onset of PFAPA was 2.0 (range 0.1-16.0) years and median age at the time of diagnosis was 4.2 (range 0.75-17.9) years. Out of the 317 patients with PFAPA, 227 (72%) were admitted to hospital during the follow-up time. The total number of in-patient stays in the cohort was 593 and the median age at the time of the stay was 3 years (range 0-23). Tonsillectomy and complications to such surgery accounted for 68 (11%) of the stays. In the further analyses, in-patient stays due to tonsillectomy were excluded. In the year before the PFAPA diagnosis was made, 79/317 (25%) patients had ≥1 in-patient stay. This decreased to 26/317 (8%) patients in the year following the PFAPA diagnosis. The total number of stays were reduced from 119 the year before PFAPA diagnosis to 32 the year after (*P*<0.001).

In the subgroup of children with PFAPA diagnosed before 4 years of age, which consisted of 150 patients, 59 (39%) were admitted for an in-patient stay ≥1 in the year before PFAPA diagnosis. This decreased to 19 (13%) the year after diagnosis. The total number of stays decreased from 88 to 24 (*P*<0.001). In the general population of children 0-4 years of age in Region Västra Götaland, the mean yearly proportion of inhabitants admitted for in-patient stays was 9% in the years 1998-2022 (2).

**Conclusion:** Children with PFAPA are frequently admitted for in-patient hospital stays the year before they are diagnosed with the condition, and admissions decrease the year after the diagnosis. Increased awareness of PFAPA syndrome and shortened diagnostic delay may reduce the need for in-patient stays.

**Patient Consent**

Not applicable (there are no patient data)

**Disclosure of Interest**

None Declared

**References**

1. Rydenman K et al. Epidemiology and clinical features of PFAPA: a retrospective cohort study of 336 patients in western Sweden. Pediatr Rheumatol Online J. 2022;20(1):82.
2. Swedish National Patient Register. Available from: https://www.socialstyrelsen.se/statistik-och-data/statistik/statistikdatabasen/ [Accessed 30-04-2024].

### Autoinflammatory diseases

#### P072 A case report of a patient with adenosine deaminase 2 deficiency from the Czech Republic

##### Erik Nedorost^1,2^, Marcel Schüller^1,2^, Helena Schneiderová^1,2^, Jana Fráňová^1,2^, Marie Macků^1,2^

###### ^1^Department of Paediatrics, University Hospital Brno; ^2^Faculty of Medicine, Masaryk University, Brno, Czech Republic

####### **Correspondence:** Erik Nedorost

*Pediatric Rheumatology 2024*, **22(2):**PReS24-ABS-1249

**Introduction:** Adenosine deaminase 2 deficiency (DADA2) is a monogenic autoinflammatory disease with a wide spectrum of phenotypic manifestations, mainly vasculitis similar to polyarteritis nodosa, often with ischemic cerebrovascular disease, immunity dysregulations, and haematological abnormalities, including bone marrow failure^1^. Due to the availability of genetic testing, diagnosis can be established even in more obscure cases.

**Objectives:** To describe a patient with clinical presentation of recurrent genital and oral ulcers and immunity abnormalities in the first patient with DADA2 reported in the Czech Republic.

**Methods:** A case report

**Results:** We present the case of a 12-year-old girl having a history of recurrent oral ulcers since infancy, sometimes accompanied by fever. Additionally, from the age of 11, the patient has suffered from recurrent genital ulcers with an increase in CRP (maximum 50.3 mg/l) and IL-6 levels (maximum 26.8 ng/l, UNL 7). Microbiological tests were repeatedly negative, and the ulcers showed no response to antimicrobial treatment. However, there was evidence of the efficacy of therapy with short courses of systemic and local steroids. Between ulceration episodes, there was normal CRP (2 mg/l), with slightly elevated IL-6 (12.6 ng/l). Further investigations revealed lower IgG, IgM and IgA levels, B-cell lymphocytopenia, and mild sideropenic anaemia responsive to iron supplementation. Laboratory tests for liver and kidney functions showed normal results. Abdominal ultrasound revealed splenomegaly, diffusely heterogenous structure of liver with mild fibrosis on transient liver elastography, and structural abnormality of vena cava inferior, confirmed by abdominal MRI with angiography. Genetic testing for immunodeficiencies identified two biallelic pathogenic missense mutations in the *ADA2* gene (c.872C>T, p.Ser291Leu and c.505C>T, p.Arg169Trp), with each parent carrying a different mutation in a heterozygous state. Subsequently, the patient’s plasma ADA2 catalytic activity was proved very low. Cardiological, ophthalmological and neurological examinations along with brain MRI including angiography found no abnormalities. Treatment with anti-TNF etanercept was initiated with a prompt effect.

**Conclusion:** Genital and oral ulcers are rather rare clinical presentations of DADA2^1^. In this case, the primary immunodeficiency genetic panel supported by immunoglobulin and B-cell abnormalities led to the diagnosis of DADA2. Fortunately, neither central nervous system vasculitis nor severe haematology abnormalities, both more indicative of the disease, have not been detected yet. To our knowledge, this is the first patient diagnosed with DADA2 in the Czech Republic.

**Patient Consent**

Yes, I received consent

**Disclosure of Interest**

None Declared

**Reference**

1. Maccora I, Maniscalco V, Campani S, et al. A wide spectrum of phenotype of deficiency of deaminase 2 (DADA2): a systematic literature review. *Orphanet J Rare Dis.* 2023;18. 10.1186/s13023-023-02721-6.

### Autoinflammatory diseases

#### P073 Chronic non-bacterial osteomyelitis in a 13-year-old male patient with spontaneous regression of soft tissue involvement

##### Lutfiye Koru, Fatih Haslak, Kubra Ozturk

###### Pediatric Rheumatology, Istanbul Medeniyet University, Istanbul, Türkiye

####### **Correspondence:** Lutfiye Koru

*Pediatric Rheumatology 2024*, **22(2):**PReS24-ABS-1740

**Introduction:** Chronic non-bacterial osteomyelitis (CNO) is a persistent bone disorder that affects children and is characterized by osteolytic lesions, leading to bone pain and soft tissue swelling. Non-steroidal anti-inflammatory drugs (NSAIDs) are the first-line treatment, while bisphosphonates or tumor necrosis factor-α (TNF-α) blockers are used in refractory cases.

**Objectives:** A 13-year-old male patient was admitted with hip pain localized especially to the left side. He described pain in the left half of the jaw that became evident with mouth movements. He had no constitutional symptoms such as fever and malaise.During the hip joint examination, the FABER test was positive in both hips, and the patient experienced tenderness in the left temporomandibular joint (TMJ). The laboratory results showed hemoglobin at 10 g/dl, white blood cell (WBC) at 17.000 Ul, neutrophil at 11.000 uL, lymphocyte count at 4600 uL, platelet count at 343.000 uL, C-reactive protein (CRP) level at 185 mg/L, and erythrocyte sedimentation rate at 70 mm/h. The MRI demonstrated diffuse bone marrow edema in both sacral bones and intense contrast enhancement in the ischial muscles and surrounding soft tissue. TMJ imaging was normal.The patient was evaluated for malignancy due to the intense contrast enhancing lesion which gave the appearance of a mass. Bone marrow aspiration performed for this purpose was normal. In addition, Positron Emission Tomography ( PET-CT) showed no findings except for a lytic lesion on the left lateral aspect of the sacrum without significant fluorodeoxyglucose (FDG) uptake. Computed tomography (CT)-guided bone biopsy was performed on the identified lesion. Chronic inflammatory osteomyelitis was detected. Tuberculosis polymerase chain reaction (PCR) was performed on the biopsy material to exclude tuberculosis in the etiology and was negative. The patient’s complaints spontaneously resolved during the investigation period within one month. The control acute phase reactants (APRs) were also turned into be normal without treatment. MRI imaging was repeated after one month, and showed that both bone and soft tissue involvement signs in sacral region were significantly regressed. The patient was considered to have CNO. Weekly subcutaneous methotrexate and alendronate treatment orally were started.

**Conclusion:** We wanted to emphasize that prominent soft tissue involvement which may be challenging to distinguish with mass may accompany in the patients with CNO, and lesions may regress spontaneously.

**Date of birth:** octobre 15

**Patient Consent**

Yes, I received consent

**Disclosure of Interest**

None Declared

**References**

1. Koryllou, A., Mejbri, M., et all. (2021). Chronic Nonbacterial Osteomyelitis in Children. *Children (Basel, Switzerland)*, *8*(7).
2. Marangoni, R. G., & Halpern, A. S. R. (2010). Chronic recurrent multifocal osteomyelitis primarily affecting the spine treated with anti-TNF therapy. *Spine*, *35*(7).

### Autoinflammatory diseases

#### P074 Mevalonate kinase deficiency treatment efficacy with Anakinra and Canacinumab

##### Ieva Šleinotienė^1,2^, Skirmante Rusoniene^1,2^

###### ^1^Pediatric, Children’s Hospital, Affiliate of Vilnius University Hospital Santaros Clinics, Vilnius, Lithuania; ^2^Vilnius University, Department of Pediatrics, Vilnius, Lithuania, Vilnius, Lithuania

####### **Correspondence:** Ieva Šleinotienė

*Pediatric Rheumatology 2024*, **22(2):**PReS24-ABS-1758

**Introduction:** Mevalonate kinase deficiency is a rare metabolic autoinflammatory syndrome caused by mutations in the MVK gene. Because of changes in the *MKV* gene, people with mevalonate kinase deficiency have reduced levels and activity of the mevalonate kinase enzyme.

Two phenotypes of mevalonate kinase deficiency are known based on the level of enzymatic deficiency, mevalonic aciduria and hyperimmunoglobulinemia D syndrome, but a wide spectrum of intermediate phenotypes has been reported. The immune system makes higher levels of interleukin 1b, which contributes to episodes of hyperinflammation. People with HIDS have between 1.8% and 28% of residual enzyme activity. Currently one of the most effective treatments is biological therapy (with interleukin-1 antagonist anakinra or tumour necrosis factor-α inhibitor etanercept).

**Objectives:** The patient in this case has a phenotype contributing to a severe disease that caused the symptoms to manifest very early, in the prenatal period. Mevalonate kinase deficiency was suspected on the basis of clinical (hydrops fetalis, hepatosplenomegaly, hypotonia) and laboratory signs (anaemia, intense acute phase reaction, increased urinary excretion of mevalonic acid). Mutation analysis of the MVK gene confirmed the biochemical diagnosis. Patient main symptoms were diarrea, abdominal pains, sometimes – fever. Treatment with the interleukin-1 antagonist anakinra was started in 2014 (minimal dose of 1 mg/kg/day) and revealed its efficacy after three days, but later it didin’t gave full efectivness and the pacient started treatment with Canacinumab (anti-IL-1β monoclonal antibody) in 2020, efficacy was seen very fast, and it lasts til now.

**Methods:** we analised patient medical records.

**Results:** Our case higlights the need for very detailed early diagnostic and shows different response to treatment with IL-1 monoclonal antibody. Shows HIDS different presentation in clinic, because our patient only has symptoms as abdominal pains and diarrea, sometimes vomitting. He very reraly had fever.

**Conclusion:** Treatment with canacinumab shows good effectivity.

**Patient Consent**

Yes, I received consent

**Disclosure of Interest**

None Declared

### Autoinflammatory diseases

#### P075 STAT1 GOF immundysregulation: a case report with chronic mucocutaneous candidiasis, polyendocrinopathy, and arthritis. Highlighting on the efficacy of adalimumab

##### Reima Bakry^1^, Yasser A. Binafif^2^

###### ^1^Pediatric, Maternity and Children Specialized Hospital; ^2^Pediatric, East Jeddah Hospital, Jeddah, Saudi Arabia

####### **Correspondence:** Reima Bakry

*Pediatric Rheumatology 2024*, **22(2):**PReS24-ABS-1476

**Introduction:** STAT1 gain-of-function is a primary immunodeficiency typically characterized by chronic mucocutaneous candidiasis, recurrent respiratory infections, and autoimmunity.

CMC is considered a component of combined immunodeficiency with increasing susceptibility to infectious agents and non-infectious agents such as autoimmune disease.CMC can be presented as a single disease or coexisting with other conditions due to immune dysregulation.

Herby, we present a case with STAT 1 GOF mutation manifesting with CMC, Hypothyroidism, partial adrenal insufficiency, short stature, and arthritis.

**Objectives:** Reporting the rare association of Arthritis with STAT1 GOF mutation anf highlighting on the efficacy of Adalimumab on controlling joint inflammation.

**Methods:** Case Presentation

13-year-old girl, who is a known case of short stature on Growth hormone and nail candidiasis and nail dystrophy following up in Endocrinology and Dermatology clinic respectively. Presented to the Pediatric Rheumatology clinic with complaints of multiple joint swelling and pain for 1 year. Joints involved include the left knee, bilateral wrists, and small joints of both hands. Her mother is known to have hypothyroidism with no other autoimmune disease in the family. Upon examination, her weight and height were below the 10^th^ centile, Exophthalmus, huge goiter, and extensive bilateral nail dystrophy of both hands. Her joint examinations showed arthritis of the left knees, bilateral wrists, and PIPs and DIPs of both hands. Her labs (Table 1) showed high inflammatory markers, positive ANA, with negative RF. Further endocrinology assessment and investigations revealed primary hypothyroidism and partial adrenal insufficiency, for which she was started on L-thyroxine and hydrocortisone. She was also started on an anti-fungal medication (Azol) . Genetic test (WES) showed a Heterozygous mutation for STAT1-GOF, C.1160C>Gp(Thr387Arg)

AD Immunodeficiency type 32C. The patient was started on Adalimumab to control her arthritis. Currently, she has JADAS10 of minimal disease activity, Thyroid function is normalized, CMC improved (figure), and repeated Quntiferon tests revealed negative results.

**Results:**

**Conclusion:** CMC, fungal and bacterial infections, and autoimmune diseases including hematological and endocrinological are common features of STAT1 GOF. However, our patient experienced arthritis that showed a prompt response to adalimumab.

**Patient Consent**

Yes, I received consent

**Disclosure of Interest**

None Declared

### Autoinflammatory diseases

#### P076 Does blood monocyte count differentiate PFAPA from FMF: a preliminary study

##### Emmanuelle Queste^1^, Perrine Dusser^1,2^, Isabelle Kone-Paut^1,2^

###### ^1^Department of Pediatric Rheumatology, National Reference Centre for Auto-inflammatory Diseases and Amyloidosis of Inflammatory origin (CEREMAIA), Bicêtre hospital, Assistance Publique-Hôpitaux de Paris (AP-HP); ^2^Paris Saclay University., Kremlin bicetre, France

####### **Correspondence:** Emmanuelle Queste

*Pediatric Rheumatology 2024*, **22(2):**PReS24-ABS-1537

**Introduction:** PFAPA is the most common auto-inflammatory disease in France. It is not always easy to diagnose with certainty, and shares common features with certain other auto inflammatory disease. Monocytes have been found to be increased in some autoimmune diseases, but also in cases of infection, inflammation and allergy.

**Objectives:** Our aim was to compare blood monocyte levels in PFAPA and FMF in crisis.

**Methods:** Monocentric retrospective study of PFAPA and FMF patients followed at Kremlin Bicêtre’s hospital and included in the BAMARA rare disease database. Demographic characteristics and white blood cell count in crisis were collected. The Mann-Whitney test was used to compare the two groups. A p <0.05 was considered statistically significant.

**Results:** Data from 20 PFAPAs and 19 FMFs were collected. There were no significant differences between PFAPA and FMF in neutrophil counts (respectively 7130/mm3 [min2722- max 16190/mm3] versus 6330/mm3 [min 2530- max 15750]) and CRP (respectively 76,5mg/l [min 43- max 254] versus 65mg/l [min 24- max 296]). In contrast, monocytosis was found in PFAPA with a median rate of 1470/mm3 [min 452- max 2460] compared with FMF (770/mm3 [min 380- max 1340]) (p<0,0001).

**Conclusion:** Monocytosis could be a discriminating factor in differentiating PFAPA from FMF or other monogenic auto inflammatory disease. A larger study is currently underway with other auto inflamatory disease to confirm these preliminary results.

**Patient Consent**

Yes, I received consent

**Disclosure of Interest**

None Declared

### Autoinflammatory diseases

#### P077 Novel genetic variants of H syndrome with elevated interferon signature: janus kinase inhibition as a potential new treatment option

##### Pascale Kurt^1^, Isabel Filges^2^, Sabrina Stefanelli^2^, Mike Recher^3,4^, Andreas Woerner^1^

###### ^1^Pediatric Rheumatology, University Children’s Hospital of Basel UKBB; ^2^Medical Genetics, Institute of Medical Genetics and Pathology, University Hospital of Basel, University of Basel; ^3^Department of Biomedicine, Immunodeficiency Laboratory, University of Basel , ^4^University Center for Immunology, University Hospital of Basel, Basel, Switzerland

####### **Correspondence:** Pascale Kurt

*Pediatric Rheumatology 2024*, **22(2):**PReS24-ABS-1541

**Introduction:** H syndrome is a rare autosomal recessive genetic disorder caused by pathogenic variants in SLC29A3 gene, leading to abnormal histiocytic proliferation and excessive inflammatory response. Clinical symptoms include recurrent fever, hyperpigmentation, hypertrichosis, hepatosplenomegaly, heart anomalies, hearing loss, hypogonadism, low height, arthritis, fixed flexion contractures of the toe or proximal interphalangeal joints and diabetes. Treatment options are limited.

**Objectives:** To report a case of H syndrome caused by novel genetic variants of SLC29A3 with high interferon (IFN) signature, predominantly autoinflammatory manifestations and prompt response to janus kinase inhibition (JAKI).

**Methods:** A 16-year-old girl presented with recurrent fever, myalgia, urticarial exanthema, livedo, plantar panniculitis and increasing fatigue. Similar episodes were described in early childhood. At the age of 4 she developed an antibody-negative diabetes. Over the course, slowly progressive camptodactyly of fingers and toes, hallux valgus and mild hypertrichosis on the lower back were noted. A sister had a history of diabetes, camptodactyly, livedo and bilateral orbital tumor with histolymphocytic pattern.Laboratory tests showed increased erythrocyte sedimentation rate (ESR) up to 50 mm/h with slightly increased CRP and serum amyloid A, hypergammaglobinemia (IgG, IgA) and increased IFN signature (median score 5, elevation of IFI27, IFI44L, ISG15, RSAD2, SIGLEC).An organ screening revealed mild hepatomegaly and mild septal hypertrophy without signs of congenital heart disease.Genetic testing for common monogenic fever syndromes was negative. Due to continued intermittent systemic inflammation the girl was treated with prednisone and methotrexate with discontinuation due to intolerance and anakinra with discontinuation due to ineffectiveness. Awaiting the extended genetic results, a therapy trial with the JAKI ruxolitinib was started.

**Results:** A trio-exome analysis was performed. The patient was found to be compound heterozygous for SLC29A3 variants c.914C>G (PM2, PM3, PP4) and c.1077_1084del (PM2, PS4, PVS1, PP4), compatible with the diagnosis of H syndrome. These SLC29A3 variants have not yet been described in literature or databases.JAKI treatment with ruxolitinib in a dose of 0.25 mg/kg twice per day resulted in rapid clinical improvement. Fever and fatigue resolved, skin lesions improved. Laboratory showed normalization of IFN signature and ESR.

**Conclusion:** H syndrome is a rare disease with heterogeneous clinical presentation due to pathogenic variants in SLC29A3. In this patient with predominantly autoinflammatory manifestations without response to IL-1 inhibition, the JAKI ruxolitinib was effective for both clinical and laboratory outcomes. JAKI may be effective in H syndrome and should be considered as a new treatment option.

**Patient Consent**

Yes, I received consent

**Disclosure of Interest**

None Declared

**References**

1. Jacquot R, Jouret M, Valentin MG, Richard M, Jamilloux Y, Rousset F, Emile JF, Haroche J, Steinmüller L, Zekre F, Phan A, Belot A, Seve P. H syndrome treated with Tocilizumab: two case reports and literature review. Front Immunol. 2023 Aug 11;14:1061182. 10.3389/fimmu.2023.1061182. PMID: 37638031; PMCID: PMC10451072.
2. Molho-Pessach V, Lerer I, Abeliovich D, Agha Z, Abu Libdeh A, Broshtilova V, Elpeleg O, Zlotogorski A. The H syndrome is caused by mutations in the nucleoside transporter hENT3. Am J Hum Genet. 2008 Oct;83(4):529-34. doi: 10.1016/j.ajhg.2008.09.013. PMID: 18940313; PMCID: PMC2561939.

### Autoinflammatory diseases

#### P078 Chronic recurrent multifocal osteomyelitis and lung involvement: report of two cases

##### Chiara Conti^1,2^, Riccardo Papa^1^, Barbara Cafferata^3^, Valerio Gaetano Vellone^3,4^, Stefano Volpi^1,2^, Marco Gattorno^1^, Roberta Caorsi^1^

###### ^1^Rheumatology and Autoinflammatory Diseases, IRCCS, Istituto Giannina Gaslini; ^2^DINOGMI, University of Genoa; ^3^Pathology Unit, IRCCS, Istituto Giannina Gaslini; ^4^DISC, University of Genoa, Genoa, Italy

####### **Correspondence:** Chiara Conti

*Pediatric Rheumatology 2024*, **22(2):**PReS24-ABS-1581

**Introduction:** Chronic recurrent multifocal osteomyelitis (CRMO) is a chronic inflammatory condition of the bone. Except for cutaneous involvement, extraskeletal manifestations are rare, in particular lung lesions.

**Objectives:** To report two CRMO pediatric cases with extensive lung consolidations.

**Methods:** We describe the clinical picture, imaging and outcome of two children with CRMO and pulmonary disease, followed by our rheumatology and autoinflammatory diaseases unit.

**Results: Case 1.** A 10-year-old Caucasian girl was followed by our tertiary referral center for an aggressive steroid-dependent SAPHO syndrome, characterized by PPP and severe bone involvement; the patient underwent treatment with different DMARDs, without complete control of the clinical picture. Three years after disease onset, while on treatment with salazopyrin and adalimumab, a radiological follow up showed stability of the bone lesions and the appearance of a large parenchymal consolidation of the right superior lobe on whole body-MRI. Histologic analysis detected an inflammatory infiltrate consistent with subacute organizing pneumonia. Salazopyrin was withdrawn. Due to the absence of symptoms, no other therapeutic changes were introduced. The lung consolidation resolved within three months and no more pulmonary anomalies were found in the following years.

**Case 2.** An 11-year-old Caucasian boy with CRMO was initially treated with ibuprofen, then withdrawn for disease remission. Due to the onset of chest pain, he underwent a chest X-ray showing a retrocardiac oval shaped image of the lung. Acute phase reactants were within normal range. A WB-MRI revealed a posteroinferior left lobe consolidation with pleural effusion and hilar lymphadenopathy, in absence of bone lesions. Bioptic findings showed an inflammatory infiltrate consistent with chronic organizing pneumonia. Non-necrotizing granulomas were also detected, despite of Quantiferon, Ziehl-Neelsen coloration and ANCA antibodies negativity. The boy was treated with NSAIDS and symptoms resolved within a few days of onset. No further treatment for CRMO was started. Radiological findings disappeared in a few months.

**Conclusion:** CRMO is an idiopathic inflammatory disorder of the bone, which is predominant among pediatric population. In contrast to skin manifestations, lung disease associated with CRMO was rarely described. The diagnostic workup of pulmonary disease may be challenging, as lung manifestations may be silent and detected occasionally. Moreover, radiological features could mimic a large variety of diseases, hence histopathology is pivotal. The evolution of the disease was benign with spontaneous disappearance in all cases. These findings suggest that lung lesions may be part of CRMO clinical picture. However, the spontaneous remission of the parenchymal consolidation, makes a direct link between the pulmonal manifestations and the disease activity still unclear.

**Patient Consent**

Yes, I received consent

**Disclosure of Interest**

None Declared

### Autoinflammatory diseases

#### P079 Ewing sarcoma in Armenian child with familial Mediterranean fever: a case report

##### Hasmik Sargsyan

###### Pediatrics N2, Yerevan State Medical University, Arabkir JMC-Institute of Child and Adolescent Health, Yerevan, Armenia

*Pediatric Rheumatology 2024*, **22(2):**PReS24-ABS-1793

**Introduction:** Familial Mediterranean Fever (FMF) is the most common hereditary monogenic autoinflammatory syndrome that is characterized by recurrent attacks of fever and polyserositis. It is found among individuals of Mediterranean descent:Armenians. Jews, Arabs, Greeks, Italians, Turks(1). The gene responsible for FMF (MEFV- *Mediterranean Fever*) is located on the short arm of chromosome 16. M694V is the most frequent mutation in Armenia. Colchicine is the basic treatment for FMF. Ewing sarcoma (ES) is a type of cancer that begins as a growth of cells in the bones and the soft tissue around the bones. ES is a highly aggressive malignancy, most common bone sarcoma affecting primarily children and adolescents(2).The incidence is much higher among Caucasians than among African Americans and Asians. A high percentage of ES cells have a chromosomal translocation, which is usually between chromosomes 22 (EWSR1) and 11(FLI1). There are few studies cancer incidence in FMF.

**Objectives:** We present FMF patient coexistence of ES.

**Methods:** Clinical and study results are presented

**Results:** With a 1.5years old boy (two years ago) was established FMF with M694V/M694V mutation in “Arabkir” JMC. Colchicine was started 0.25mg/day and gradually increased up to 0.75mg/day. The pain and swelling of the right thigh was manifested after 6 months of diagnosis of FMF.The investigations with oncohematologist confirm the diagnosis of the tumor of the Ewing family (MRI, histological typical changes, EWSR1:FLI1 genes).The radiation treatment and chemotherapy (VDC/IE) was prescribed.

**Conclusion:** Coexistence of two above genetic disorders requires holistic approach, taking into consideration the pathogenesis of these diseases, combination and side effects of drugs. Not excluded, that patients with the M694V/M694V mutation could be the risk factors not only for the development of complications (amyloidosis, intestinal obstruction, liver involvement) of FMF, but also play a favorable role for the development oncology diseases involvement in FMF. However, for the understanding relationship between ES and FMF should be further studies with more patients for a providing precise interpretation.

**Date of birth:** avril 05,

**Patient Consent**

Yes, I received consent

**Disclosure of Interest**

None Declared

**References**

1. Sohar E, Gafni J, Pras M, Heller H (1967) Familial Mediterranean fever. A survey of 470 cases and review of literature. Amer. J Med.43:227-53
2. 10.1371/journal.pone.0053223

### Autoinflammatory diseases

#### P080 Preliminary results of a molecular genetic study on the “Autoinflammatory syndromes” panel of patients with systemic juvenile idiopathic arthritis in Moscow

##### Sania Valieva, Seda Kurbanova, Ivan Gaziev, Yuliya Shubina

###### Morozov City Children’s Clinical Hospital , Moscow, Russian Federation

####### **Correspondence:** Seda Kurbanova

*Pediatric Rheumatology 2024*, **22(2):**PReS24-ABS-1587

**Introduction:** Autoinflammatory diseases (AIDs) are being intensively studied. Molecular genetic testing of patients is of great importance for the diagnosis of AIDs, since the basis for the development of AIDs are pathological mutations that cause disturbances in the innate (antigen-nonspecific) immune system and the development of inflammation. This also applies to patients with systemic juvenile arthritis (sJIA), which in recent years has been classified in the AIDs group due to the great similarity of symptoms. In this regard, it became quite reasonable to assume that in a number of patients, monogenic AIDs are hidden under the mask of sJIA.

**Objectives:** identify patients with monogenic AIDs among patients with sJIA using molecular genetic testing on the “Autoinflammatory diseases” panel

**Methods:** Currently, results have been obtained from 38 patients with sJIA. A molecular genetic analysis of 117 genes in the “Autoinflammatory syndromes” panel was performed using mass sequencing (NGS). Molecular genetic analysis was carried out in the laboratory at the Morozov Children’s City Clinical Hospital, Moscow. The medical records of 38 patients with a final diagnosis of sJIA were retrospectively analyzed.

**Results:** The study included 38 children (boys - 21, girls - 17) aged from 11 months to 17 years, median - 10 years (6.7; 13.5), who were hospitalized in the rheumatology department with a referral diagnosis of sJIA. Clinical manifestations characteristic of sIJA were observed with the following frequency: febrile fever - 97.3%, arthritis - 62%, arthralgia - 23%, rash - 68.4%, lymphadenopathy -71.1%, hepatolienal syndrome - 71.1%, serositis – 31.5%.Macrophage activation syndrome was observed in 34.2% patients.Before molecular genetic examination of autoinflammatory syndrome, all patients were observed with a diagnosis of sJIA and received the following therapy: tocilizumab - 89.5%, canakinumab - 10.5%, methotrexate - 42%. All patients with macrophage activation syndrome received glucocorticosteroids.In 6 (15.7%) patients, variants of pathogenic mutations in the studied genes were identified: 1 patient - p.R334Q in the NOD2 gene (Blau Syndrome), 2 patients - p.V726A in the MEFV gene, 3 patients - p. K695R. MEFV. Other genetic variants have also been identified, the clinical significance of which remains to be studied: 2 patients - c.841_849+19del mutation in the C2 gene, 2 patients - p.F28L in the RIPK1 gene.

**Conclusion:** In 6 (15.7%) patients with suspected AIDs , a molecular genetic study on the “Autoinflammatory syndromes” panel allowed us to identify gene mutations. It is necessary to continue screening patients with sJIA for autoinflammatory diseases. Patients with a negative result of molecular genetic testing require further examination with whole exome sequencing.

**Patient Consent**

Yes, I received consent

**Disclosure of Interest**

None Declared

### Autoinflammatory diseases

#### P081 An analysis of demographics, clinical presentation, and investigations in children and young people under 18 years of age with Chronic Recurrent Multifocal Osteomyelitis (CRMO) managed at a tertiary paediatric rheumatology centre: a retrospective audit

##### Nabilah Ali^1^, Nandita Pai^2^, Kapil Gargh^2^, Samundeeswari Deepak^2^, Kishore Warrier^2^

###### ^1^University of Nottingham; ^2^Nottingham University Hospitals NHS Trust, Nottingham, United Kingdom

####### **Correspondence:** Nabilah Ali

*Pediatric Rheumatology 2024*, **22(2):**PReS24-ABS-1613

**Introduction:** Chronic Recurrent Multifocal Osteomyelitis (CRMO) or Chronic non-bacterial osteomyelitis (CNO) is a rare inflammatory bone disorder characterised by sterile osteolytic lesions in bones, mainly in children and young adults. Patients with CRMO often present with nonspecific symptoms to a variety of specialties. The diagnosis can be delayed, as it involves exclusion of infection and neoplasia. However, delayed or missed diagnosis can lead to vertebral fractures and chronic pain.

**Objectives:** To investigate the demographic characteristics, clinical presentation and investigations in paediatric patients with CRMO, aiming to provide insights that facilitate education of clinicians and refine diagnostic pathways, ultimately improving the accuracy and timeliness of the diagnosis in children. As a secondary aim, we looked at additional information like referring specialists and comorbidities.

**Methods:** This was a retrospective study, collecting data from electronic medical records of 10 consecutive patients with CRMO managed by Paediatric Rheumatology team at Nottingham Children’s Hospital since 2019.

**Results:** The mean age of diagnosis was 13 years with 60% being females. The main source of the referral was the orthopaedics with 60%, followed by the maxillofacial surgery (20%). The most common presenting symptoms were pain (80%), swelling (40%) and limitation of movements (30%). 80% of the patients had symptoms pertaining to only one site, although 90% of the patients had changes in multiple sites on WBMRI. The most common areas affected were pelvis (70%), tibia (50%), spine (30%) and femur (30%). Pelvis, spine and tibia were the most common sites with asymptomatic involvement, noticed on the WBMRI. Only 40% of patients required biopsy; the rest were diagnosed based on typical features on WBMRI. The comorbidities amongst these patients included arthritis (20%), inflammatory bowel disease (20%), psoriasis (10%) and SAPHO syndrome (10%).

**Conclusion:** The data show that CRMO can present with a wide variety of symptoms, coming through a range of specialists. It further shows a significant asymptomatic involvement of sites, highlighting the importance of WBMRI in the diagnosis, which in some cases may obviate the need of invasive procedures like biopsy. Increasing awareness of this condition among paediatricians as well as other specialists is key to early diagnosis and management.

**Date of birth::** août 21, Y

**Patient Consent**

Not applicable (there are no patient data)

**Disclosure of Interest**

None Declared

### Autoinflammatory diseases

#### P082 Coexistence of Blau syndrome with the hemoglobin C trait misdiagnosed as systemic juvenile idiopathic arthritis treated successfully with tocilizumab

##### Awatif A. Abushhaiwia

###### Paediatric Rheumatology, Tripoli Children’s Hospital, Faculty Of Medicine, University Of Tripoli, Tripoli , Libya

*Pediatric Rheumatology 2024*, **22(2):**PReS24-ABS-1035

**Introduction:** BS is a rare monogenic autoinflammatory granulomatous disease that occurs as a result of mutations in the NOD2 gene pattern recognition receptor. which mostly presenting before age 5.Is characterized by a clinical triad of symmetrical polyarthritis, granulomatous skin dermatitis, and recurrent uveitis.

**Objectives:** Highlighting that BS remains underreported, and often misdiagnosed as s juvenile idiopathic arthritis, however,Tocilizumab may prove to be the treatment of choice in this situation

**Methods:** We report a rare case of coexistence of BS with the hemoglobin C trait characterised by significant, persistent symmerical boggy polyarthritis, and systemic inflammation without eyes or skin manifestations successfully treated with tocilizumab.Which was administered IV at a dose of 12 mg/kg once per month after failing to respond to systemic steroids and methotrexate therapy sequentially.

**Results: A 34-month-old** Libyan black girl presented with persisting polyarthritis involving bilateral metacarpophalangeal (MCP) proximal and distal interphalangeal (PIP, DIP) joints of the hands, wrists, elbows, knees, and ankles since 11 months associated with progressive flexion contractures of her third, fourth, and fifth fingers of both hands (camptodactyly). She has also had an intermittent fever since then. She was initially diagnosed with systemic juvenile idiopathic arthritis that was treated with oral corticosteroids and methotrexate SC; she also received IVIG, which had no effect. She had no significant family history.Her physical examination revealed all her major joints and small joints were affected. Cushioned face, hirsutism, pale , No lymphadenopathy, and no organomegally.Other systemic examinations were unremarakable.Her blood tests showed WBC18X103, HGB 6.6g\dl, MCV 48.5, MCHC 25.3, PLAT 680 x103, lymph 33%, and NEUT 61% blood film showed leuckocytosis with absolute neutrophili anisocytosis, poikilocytosis hyopchromic miicrocytic .ESR 80 ml/hr, CRP > 300 mg/dl, S ferritin 163 was normal.ANA, and RF were all negative.Serum amyloid A 305 mg/l; very high, ACE was negative.Haemoglobin electrophoresis also ordered to rule sickle cell anaemia, which revealed the haemoglobin C trait , Haemoglobin C 36%, echocardiography was normal.Her eyes exam was normal.Based on the clinical findings,She was diagnosed with BS, which has persistent boggy polyarthritis without ocular or skin manifestations.Treated with a combination of oral predinsolone 1mg\kg\day, which was tapered gradually on each following visit until 0.2mg\kg. methotrexate SC, colchicine tab 0.5mg pluse Tocilizumab IV. WBC, ESR CRP and SAA rapidly decreased to normal levels Haemoglobin improved and returned to normal, after therapy.She responded good to Tocilzumab and is currently doing well. Only she needs to do genetic analysis of the CARD15/Nod2 gene to confirm diagnosis.

**Conclusion:** This case provides further evidence of the efficacy of tocilizumab IV in BS.Unfortunately, genetic analysis couldn’t be performed due to financial limitations, a common problem in countries like Libya.

**Patient Consent**

Yes, I received consent

**Disclosure of Interest**

None Declared

**References**

1. Katherine P. Kaufman & Mara L. Becker. Distinguishing Blau Syndrome from Systemic Sarcoidosis.Current Allergy and Asthma Reports (2021) 21: 10. 10.1007/s11882-021-00991-3.
2. Katherine P. Kaufman Distinguishing Blau Syndrome from Systemic Sarcoidosis.Current Allergy and Asthma Reports (2021) 21: 10. 10.1007/s11882-021-00991-3.

### Autoinflammatory diseases

#### P083 Hidradenitis suppurativa: when it’s first misdiagnosed in adolescent with poor treatment adherence

##### Dragana S. Lazarevic^1,2^, Maja Zecevic^2,3^, Jelena Vojinovic^1,2^

###### ^1^Clinic of Pediatrics, University Clinical Center Nis, Serbia; ^2^Faculty of Medicine, University of Nis; ^3^Clinic For Pediatric Surgery and Orthopedics, University Clinical Center Nis, Nis, Serbia

####### **Correspondence:** Dragana S. Lazarevic

*Pediatric Rheumatology 2024*, **22(2):**PReS24-ABS-1052

**Introduction:** Hidradenitis suppurativa (HS) is a chronic recurrent inflammatory disorder of the skin primary affecting apocrine gland areas of the body. It is presented with painful nodules, abscesses and scarring of the intertriginous areas. If misdiagnosed or poorly managed, HS significant decrease patient’s quality of life.

**Objectives:** Delayed diagnosis of hydradentits suppurativa in female adolescent with poor treatment adherence.

**Results:** Female adolescent presented with right axillary abscesses. Biopsy and patohistological diagnose of cat scratch disease was established. Despite the treatment, abscess perforated and antibiotics started according to the isolated Staphyloccocus epidermis. Four months later an axillary perforative abscess occured on the opposite side, with Klebsiella species in culture. That period we saw her for the first time. He denied other complaints except the hair loss. Family history was positive for inguinal and perianal abscesses. Diferential diagnostic sum up work was done. Laboratory finding revealed elevation of inflammatory biomarkers, neutrophilia, hypochromic anemia and hypergammaglobulinemia. Antinuclear, antidsDNA, ANCA, ASCA antibodies were negative. Additional diagnostics methods ruled out the possibility of hematological disease, chronic granulomatous disease (NBT test normal), sarcoidosis (ACE enzyme normal), inflammatory bowel disease (fecal calprotectin negative) and tuberculosis (quantiferon test negative). Thyroid gland function, ultrasound of the abdomen and X ray of the lungs and heart were normal. As complaints persisted, rebiopsy was done and diagnose of HS was confirmed. According to the protocol, dual antibiotic therapy (Clindamycin and Rimfapicin) was administrated, but patient was unable to complete it for 10 weeks due to nausea. Therefore, Dapsone treatment was introduced, but with poor treatment adherence and therefore slight clinical improvement. We have decided to start biologics and Adalimumab was commenced with significant clinical and laboratory improvement only 3 months later.

**Conclusion:** Proper, timely diagnosis of HS, adequately conducted treatment and patient cooperation are crucial to achieve maximum results and enable normal functioning of the patients.

**Date of birth::** octobre 28

**Patient Consent**

Yes, I received consent

**Disclosure of Interest**

None Declared

### Autoinflammatory diseases

#### P084 Novel MVK mutation (C.298G>A); a new case of hyper-IGD syndrome

##### Reza Shiari^1^, Pooneh Tabibi^1^, Shabnam H. Ghotbabadi^2^

###### ^1^Pediatrics, Shahid Beheshti University of Medical Sciences , Tehran; ^2^Pediatrics, Shiraz Medical University, Shiraz, Iran, Islamic Republic Of

####### **Correspondence:** Reza Shiari

*Pediatric Rheumatology 2024*, **22(2):**PReS24-ABS-1480

**Introduction:** Hyper IgD syndrome (HIDS) is a rare autosomal recessive autoinflammatory condition, determined by recurrent fe- brileattacksassociatedwithlymphadenopathy, arthralgia, diarrhea, abdominal pain, and skin rash.This disease has an early onset, generally in infancy, and febrile crises recur at varying intervals. Fever flares have a sudden onset and last approximately 4–6days. An attack begins with chills, and patients often complain of weakness, headache, nausea, and diarrhea.The hallmark character- istic of the syndrome is the presence of recurrent episodes of fever and chills, often accompanied by skin rashes. HIDS is caused by a mutation in the mevalonate kinase (MVK) gene, which results in a lack of mevalonate kinase enzyme activity.

**Objectives:** Early diagnosis and treatment of HIDS patients is momentous, because it prevents irreversible organ damage and improves the quality of life of patients. Management of HIDS focuses primarily on symptomatic relief of febrile episodes and prevention of complications. Nonsteroidal anti-inflammatory drugs (NSAIDs) are considered as first- line treatment for pain and fever associated with HIDS episodes. In some patients, immunomodulatory therapy may be recommended to help control the frequency and severity of episodes.

**Methods:** A 5-year-old girl, born of a non-consanguineous marriage, presented with clinical indications of low to moderate-grade fever persisting for the past 2years, along with recurrent skin lesions and infections. The patient, a first-born child of non-consanguineous parents (G1P1L1), had a birth weight of 3800 g and current weight of 17 kg. Her date of birth was January 2017, and she had a history of normal neurodevel- opment. She was a native of Tajikistan with no known his- tory of specific diseases. There were no positive points in the family history regarding similar conditions. Approximately 2years ago, the patient started expe- riencing episodes of low to moderate-grade fever and recurrent generalized skin lesions, affecting the trunk, abdomen, and face. The skin lesions exhibit a recurring pattern and resemble petechial rashes observed in certain areas of the body. She also complained of arthralgia. Initially, multiple physicians in Tajikistan suspected a der- matological condition.

**Results:** Whole-exome sequencing was performed on a blood sample from the patient. Variant interpretation of specific variants of interest was conducted. A heterozygous missense varia- tion was identified in exon 4 of the MVK gene, resulting in the amino acid substitution of asparagine for aspartic acid at codon 100 (▫▫.298▫▫>▫▫; ▫▫. ▫▫▫▫▫▫100▫▫▫▫▫▫). This variant is classified as having uncertain significance according to the ACMG guidelines and is associated with the OMIM phenotype of Hyper IgD Syndrome, which is typically caused by homozygous or compound heterozygous mu- tations in the MVK gene. The clinical symptoms of the patient are consistent with this phenotype.

**Conclusion:** The variant in MVK (C.298G>A) is a mutation that can lead primary immunodeficiency in patients. Autoinflammatory syndromes always pose diagnostic and therapeutic challenges for therapists. Our report highlights the broad clinical phenotype of MVK, and emphasizes the need to consider early genetic screening for young children presenting with attacks of fever associated with skin lesions. Effective management of HIDS involves a multidisciplinary approach in which rheuma- tologists, immunologists, and geneticists collaborate to provide comprehensive care.

**Date of birth::** décembre 3

**Patient Consent**

Yes, I received consent

**Disclosure of Interest**

None Declared

**References**

1. D’Osualdo A, Picco P, Caroli F, et al. MVK mutations and as- sociated clinical features in Italian patients affected with auto- inflammatory disorders and recurrent fever. Eur J Hum Genet. 2005;13(3):314-320.
2. Santos JA, Aróstegui JI, Brito MJ, Neves C, Conde M. Hyper-IgD and periodic fever syndrome: a new MVK mutation (p.R277G) associated with a severe phenotype. Gene. 2014;542(2):217-220.

### Autoinflammatory diseases

#### P085 Chronic nonbacterial osteomyelitis in pediatric age: a case series

##### Inês Santos^1^, Inês Magalhães^2^, Francisca Aguiar^3,4^, Sara Ganhão^3,4^, Mariana Rodrigues^3,4^, Iva Brito^3,4^

###### ^1^Rheumatology Unit, Unidade Local de Saúde de Viseu Dão-Lafões, Viseu; ^2^Pediatric Department, Unidade Local de Saúde do Alto Minho, Viana do Castelo; ^3^Pediatric and Young Adult Rheumatology Unit, Unidade Local de Saúde São João; ^4^Department of Medicine, Faculty of Medicine of University of Porto, Porto, Portugal

####### **Correspondence:** Mariana Rodrigues

*Pediatric Rheumatology 2024*, **22(2):**PReS24-ABS-1184

**Introduction:** Chronic nonbacterial osteomyelitis (CNO) is an autoinflammatory bone disease most prevalent in children that covers a wide clinical spectrum, with mild and limited single-site involvement at one end and severe, chronically active or recurrent multifocal disease at the other end.^1^

**Objectives:** Our study aimed to characterize the demographic features, clinical presentation and short-term response to treatment of CNO patients.

**Methods:** This is a retrospective single-center study including patients under 18 years of age diagnosed with CNO between 2007 and 2023. Demographic and clinical data were collected. The diagnosis of CNO was based on the history of bone pain and/or local swelling, presence of radiologic evidence of compatible lesions and bone biopsies showing sterile chronic inflammation.

**Results:** A total of 4 patients were included, all of whom were females. Main demographic, clinical and therapeutic characteristics are described in Table 1. The mean age at diagnosis was 7.3±4.3 years and the mean delay in diagnosis was 5.3±4.8 months.

Most patients had multifocal disease (n=3; 75%) and the pelvic girdle was the most commonly involved location (n=3; 75%). Bone pain was universally present with local swelling in 50% (n=2), mild fever in 25% (n=1) and acne in 25% (n=1). Patient 3 presented early-onset peripheral arthritis and pustulosis suggestive of a monogenic form of CNO, but no genetic cause was identified. No patients in this series had axial arthritis, enthesitis, psoriasis, uveitis or inflammatory bowel disease.

A non-steroidal anti-inflammatory drug (NSAID) was used as the first-line therapy in all the patients, with clinical improvement at the first month in 75% (n=3). Exceptionally, in case 3, NSAID was combined *ad initium* with a corticosteroid and pamidronate; however, no response was observed after 1 month.

The mean follow-up time was 63.3±63.5 months and no complications were reported.

**Conclusion:** Our case series corroborates the clinical heterogeneity of CNO. In addition to the lack of awareness for this condition, this can justify a significant diagnostic delay, although we report a lower value than other studies.^1, 2^

**Date of birth:** septembre

**Patient Consent**

Yes, I received consent

**Disclosure of Interest**

None Declared

**References**

1. McCann L, Beresford M, Hahn G, Hedrich C. Chronic Nonbacterial Osteomyelitis. In: Efthimiou P, ed. *Auto-Inflammatory Syndromes: Pathophysiology, Diagnosis, and Management*. New York: Springer; 2019:227-247.
2. Girschick H, Finetti M, Orlando F, et al. The multifaceted presentation of chronic recurrent multifocal osteomyelitis: a series of 486 cases from the Eurofever international registry. Rheumatology (Oxford). 2018;57(7):1203-1211.

### Autoinflammatory diseases

#### P086 Clinical case of cryopyrin-associated periodic syndrome

##### Iryna Chyzheuskaya^1^, Tatyana Matsushko^2^, Anastasiya Chyzhevskaya^3^, Lyudmila Belyaeva^1^

###### ^1^4th City Children’s Clinical Hospital; ^2^Minsk Scientific and Practical Center for Surgery, Transplantology and Hematology; ^3^Institute of Genetics and Cytology of the National Academy of Sciences of Belarus, Minsk, Belarus

####### **Correspondence:** Iryna Chyzheuskaya

*Pediatric Rheumatology 2024*, **22(2):**PReS24-ABS-1395

**Introduction:** Cryopyrin-associated periodic syndrome (CAPS) is a group of monogenic autoinflammatory diseases, based the development of which is a mutation in the NLRP3 gene. When discussing the manifestations of CAPS, special attention should be paid to the diagnostic search due to the rarity of this disease and the lack of clinical knowledge about it in pediatric practice. This leads to an incorrect diagnosis, the prescription of ineffective treatment, to the progression of the disease and disability of the patient.

**Objectives:** conduct a clinical analysis of the CAPS

**Methods:** The materials of the work were anamnestic, clinical, laboratory and instrumental data from a clinical case of CAPS in a 15-year-old teenager who was treated at the 4th City Children’s Clinical Hospital in Minsk.

**Results:** The patient was born from a multiple pregnancy (triplets, in vitro fertilization). He has been ill since 7 months, when, against the background of a high fever, a large-spotted rash appeared on the body, disappearing after the body temperature normalized and reappearing again at the peak of the fever. Laboratory examination revealed an increase in ESR and CRP, and neutrophilic leukocytosis. At the age of 1 year, articular syndrome of the type of oligoarthritis appeared. The diagnosis was made: Juvenile arthritis with systemic onset. Medrol was prescribed, which resulted in clinical improvement, but when an attempt was made to discontinue medrol, the disease recurred again. In 2013, methorexate was added to the therapy, but it was not possible to achieve remission of the disease. In 2014, it was decided to initiate biological therapy with the tocilizumab. An allergic reaction such as urticaria was observed after the first administration of tocilizumab, and therefore the drug was discontinued. In January 2017, adalimumab therapy was initiated, but no effect was achieved. In December 2017, hearing loss was noted. Over the course of a year, bilateral acute sensorineural hearing loss progressed from grade 1 to grade 3. In the summer of 2020, a complete analysis of the NLRP3 gene was carried out using direct sequencing; single nucleotide substitutions were found, described in the HGMD database as pathogenic: c.2269G>A, p.Gly757Arg NM_001079821.2 in a heterozygous state. Based on the data obtained, the diagnosis is highly probable: Cryopyrin-associated periodic syndrome. In February 2021, biological therapy with the interleukin-1 inhibitor canakinumab was initiated at a dose of 150 mg subcutaneously once every 8 weeks, against the background of which clinical and laboratory improvements were noted: relief of rash and fever, normalization of general clinical and biochemical blood tests, improvement of hearing in as the degree of hearing loss decreased from grade 4 to grade 1.

**Conclusion:** For a long time, the patient was diagnosed with juvenile arthritis with systemic onset, and appropriate treatment was carried out without significant success. The correct diagnosis was made only in 2020. The only treatment method that can stop the manifestations of the disease and prevent life-threatening kidney damage is the use of IL-1b inhibitors.

**Patient Consent**

Yes, I received consent

**Disclosure of Interest**

None Declared

### Autoinflammatory diseases

#### P087 Some diseases and familial Mediterranean fever: why they are connected?

##### Hasmik Sargsyan

###### Pediatrics N2, Yerevan State Medical University, Arabkir JMC-Institute of Child and Adolescent Health, Yerevan, Armenia

*Pediatric Rheumatology 2024*, **22(2):**PReS24-ABS-1813

**Introduction:** Familial Mediterranean Fever (FMF) is an autosomal recessive disease particularly frequent around the Mediterranean basin. FMF characterized by recurrent fever, peritonitis, arthritis, pleuritis, pericarditis attacks. The frequent association of FMF with other disorders is remarkable and of great interest. Gilbert syndrome is an autosomal recessive disorder of bilirubin metabolism within the liver. Psoriasis is an immune-mediated disease that causes inflammation such as raised plaques and scales on the skin. Menstrual disorders are problems that affect a woman’s normal menstrual cycle.

**Objectives:** To describe coexistence of FMF with psoriases, Gilbert syndrome, menstrual cycle disorders and ovarian dysfunction. The diagnosis of FMF established according criteria Tel-Hashomer and genetic investigation. Coexistence other diseases with FMF was diagnosed according clinical and laboratory results. There are some cases of FMF with Gilbert syndrome(1), FMF with psoriasis(2), FMF with menstrual cycle disorders, ovarian dysfunction(3). Despite the high level of FMF in Armenia, this is the first case of these disorders in the same patient.

**Methods:** Clinical and laboratory findings are presented.

**Results:** 15year-old girl admitted to the “Arabkir” JMC with complains of: abdominal pain, chest pain , fever, arthralgia, myalgia.. Examination results: ECR 40mm/hour, CRP 6.12mg/dl, splenomegaly, plural effusion from the left side, MEFV - M694V/V726A mutations. FMF was established and treatment with colchicine was started by 1.0mg/day. She is under the follow up gynecologist due to above mentioned diseases (from 14years old), was diagnosed with psoriasis at age of five. The family history was: father has psoriasis.

**Conclusion:** This is a case presenting coexistence of FMF with Gilbert syndrome, psoriasis, menstrual cycle disorders, ovarian dysfunction. However, we suppose, that these diseases may be related not only with the relationship of geneses, but also with major pathological development in mesenchymal tissue in morphogenesis. We do not exclude that the manifestation of the above mentioned diseases can be explained by the concept of Signal Transduction.

**Date of birth::** avril 05,

**Patient Consent**

Yes, I received consent

**Disclosure of Interest**

None Declared

**References**

1. 10.1111/ped.14526. Epub 2021 Jun 8.
2. 10.1080/03009734.2017.1423425
3. 10.1155/2015/380354

### Autoinflammatory diseases

#### P088 Clinical case of chronic non-bacterial osteomyelitis initially presented by symptoms of systemic juvenile arthritis: long way to diagnosis

##### Svetlana Salugina^1^, Irina Nikishina^1^, Aliya Arefieva^1^, Olga Borodacheva^1^, Emil Gasymov^2^

###### ^1^Pediatric; ^2^Radiology, V.A.Nasonova Research Institute of Rheumatology, Moscow, Russian Federation

####### **Correspondence:** Irina Nikishina

*Pediatric Rheumatology 2024*, **22(2):**PReS24-ABS-1258

**Introduction:** Systemic juvenile idiopathic arthritis (sJIA) is the most severe variant of JIA, occurring with signs of a systemic inflammation. Chronic non-bacterial osteomyelitis (CNO) is a rare autoinflammatory condition with aseptic bone lesion. Both of them belong to multifactorial autoinflammatory desease (AIDS) and may have similar manifestations

**Objectives:** To describe a clinical case of 10 years way to diagnosis of CNO in boy who was initially recognized as having sJIA.

**Methods:** Description of clinical case of 3 y.o. boy with CNO who were observed in our department whithin 10 years.

**Results:** 3 y.o. boy in 2012 had skin-abdominal form of hemorrhagic vasculitis due to vaccination. Then arthritis of ankle and left knee manifested with polymorphic maculo-papular rash, fever, ESR 58mm/h, CRP 229mg/l. sJIA was verified and patient received GС, methotrexate (MTX) with good response. Subsequently recurrent episodes of fever, rash, arthritis observed with no functional insufficiency. Infections, oncology, IBD, monogenic AIDS were excluded. By 2022, periodic episodes of fever up to 4 times a year, severe pain in the left elbow joint appeared including night pain. Osteitis detected on MRI of the left elbow joint and «whole body» (WB) MRI verified multiple areas of bone marrow edema and synovitis of the knees, ankles, left elbow joint. The diagnosis was changed to CNO. Previous treatment with NSAIDs and MTX was continued and etanercept was administrated with good response. In February, 2023, there were no pain, fever, arthritis, skin rashes, laboratory activity and Improving of joint function and positive dynamics according to MRI data were detected. NSAIDs and GC have been completely abolished. No adverse events observed.

**Conclusion:** This case shows that CNO may be diagnosed in 10 years after disease onset. Pts with manifestations of systemic inflammation signs, arthritis, night pain in bone and joints, especially in atypical course of the disease, insufficient response to therapy, need to exclude CNO.

**Patient Consent**

Yes, I received consent

**Disclosure of Interest**

None Declared

### Autoinflammatory diseases

#### P089 Haploinsufficiency of A20 (HA20)- unravelling of a suspect autoinflammatory disorder

##### Karolina Warciak^1^, Emma J. MacDermott^1^, Emer Fitzpatrick^2^, Orla Killeen^3^

###### ^1^Rheumatology, National Centre for Rheumatology, CHI - Crumlin; ^2^Gastroenterology, CHI – Crumlin; ^3^Rheumatology, National Centre for Rheumatology, CHI-Crumlin, Dublin, Ireland

####### **Correspondence:** Karolina Warciak

*Pediatric Rheumatology 2024*, **22(2):**PReS24-ABS-1594

**Introduction:** We present the first Irish case of HA20 in a 16-year-old female.

**Objectives:** HA20 is a novel autoinflammatory disease that results in the disturbance in the ubiquitination process ^1^. HA20 results from mutation in Tumour Necrosis Factor a Induced Protein 3 gene(TNF/AIP3) located on chromosome 6^1-6^, that activates the nuclear factor NFkB pathway resulting in increased expression of proinflammatory cytokines and systemic inflammation^1-6^.

**Methods:** Our patient had recurrent bloody diarrhoea from 13 months of age. At 3-years-of age, she had onset of painful mouth, perianal and genital ulcers, abdominal pain and iron deficiency anaemia. At 5-years-9 months, gastroscopy and colonoscopy confirmed Crohn’s disease of mouth, colon, terminal ileum and stomach. Sulfasalazine and Benzoate free diet was commenced with her gastrointestinal symptoms resolving within a few months, but painful mouth perianal and genital ulcers were constant over the following 10 years.

At 10-years-old, patient was diagnosed with severe atopic conjunctivitis and she reported recurrent arthralgias. At 12 years a colonoscopy showed deep ulceration throughout the colon but biopsy was deferred due to bleeding concerns. A trial of Adalimumab subcutaneously was commenced in addition. Over the course of her illness, she had persistently raised erythrocyte sedimentation rate (ESR), highest faecal calprotectin of 670ug/g, one raised anti-dsDna, and a moderately positive antinuclear antibody. A recent admission with fever, severe headache, ocular pain, painful genital and mouth ulcers and intermittent arthralgia resulted in consultation with our Rheumatology service and autoinflammatory panel (CEGAT)was requested.

**Results:** Colchicine and a weaning course of Prednisolone were started, and Adalimumab was increased to 80mg. Whole exome sequencing confirmed a TNFAIP3 mutation. On follow up, genital ulceration and fever had resolved, with infrequent mouth ulcers or joint pains.

**Conclusion:** HA20 should be considered in all cases of early onset chronic mucosal (oral and genital) ulcerations, in particular conditions similar or suspected to be Behcet’s disease like in nature.

**Patient Consent**

Yes, I received consent

**Disclosure of Interest**

None Declared

**References**

1. Debeljak, Blazina S, J. Brecelj, T. Avčin, N. Toplak. The spectrum of clinical presentation in haploinsufficiency of A20; a case report of a novel mutation in TNFAIP3 gene. Frontiers in pediatrics [Internet]. 2023 Jun 14 [cited 2024 May 2];11. Available from: https://doi.org/10.3389%2Ffped.2023.1132596
2. Aeschlimann FA, Laxer RM. Haploinsufficiency of A20 and other paediatric inflammatory disorders with mucosal involvement. Current Opinion in Rheumatology [Internet]. 2018 Sep 1 [cited 2022 May 16];30(5):506–13. Available from: https://journals.lww.com/corheumatology/Abstract/2018/09000/Haploinsufficiency_of_A20_and_other_paediatric.9.aspx
3. Zhang D, Su G, Zhou Z, Lai J. Clinical characteristics and genetic analysis of A20 haploinsufficiency. Pediatric Rheumatology. 2021 May 24;19(1).
4. Zanatta L, Biscaro F, Bresolin S, Maurizio Marzaro, Sarcognato S, Cataldo I, et al. Case Report: An early-onset inflammatory colitis due to a variant in TNFAIP3 causing A20 haploinsufficiency. Frontiers in pediatrics. 2022 Nov 18;10
5. Berteau F, B. Rouviere, Aurélien Delluc, Nau A, Rozenn Le Berre, Guillaume Sarrabay, et al. Autosomic dominant familial Behçet disease and haploinsufficiency A20: A review of the literature. 2018 Aug 1;17(8):809–15
6. Aeschlimann FA, Batu ED, Canna SW, Go E, Gül A, Hoffmann P, et al. A20 haploinsufficiency (HA20): clinical phenotypes and disease course of patients with a newly recognised NF-kB-mediated autoinflammatory disease. Annals of the Rheumatic Diseases. 2018 Jan 9;77(5):728–35

### Bone in rheumatic diseases

#### P091 One-year post-diagnosis outcome in a national Chronic Recurrent Multifocal Osteomyelitis / Chronic Non-bacterial Osteomyelitis (CRMO/CNO) cohort

##### Sarah Sayers^1^, Daphne T. Chia^1^, Andoni Toms^2^, Anish Sanghrajka^3^, Athimalaipet Ramanan^4,5^, Orla Killeen^6^, Christina Ilea^7^, Clare Pain^8^, Neil Martin^9^, Kate Armon^1,10^, Chenqu Suo^1^

###### ^1^Department of Paediatrics, University of Cambridge, Cambridge; ^2^Department of Radiology; ^3^Department of Trauma and Orthopaedics, Norfolk and Norwich University Hospitals, Norwich; ^4^Department of Paediatric Rheumatology, Bristol Royal Hospital for Children; ^5^Translational Health Sciences, University of Bristol, Bristol, United Kingdom; ^6^National Centre for Paediatric Rheumatology, Children’s Health Ireland, Dublin, Ireland; ^7^Department of Paediatrics, Royal National Orthopaedic Hospital, London; ^8^Department of Rheumatology, Alder Hey Children’s Hospital, Liverpool; ^9^Department of Paediatric Rheumatology, Royal Hospital for Children, Glasgow; ^10^Department of Paediatric Rheumatology, Cambridge University Hospitals, Cambridge, United Kingdom

####### **Correspondence:** Sarah Sayers

*Pediatric Rheumatology 2024*, **22(2):**PReS24-ABS-1102

**Introduction:**

CRMO, also known as CNO, is an autoinflammatory condition affecting the bones of children. There is a wide spectrum of clinical presentations, ranging from singular lesions to relapsing, multifocal, or continuous disease. There is currently a lack of prospective study on the short to medium term disease outcome.

**Objectives:**

The primary aim of this study was to understand the one-year post-diagnosis outcome in a prospectively identified national cohort of patients with CRMO/CNO in the United Kingdom and Republic of Ireland.

**Methods:**

We conducted monthly surveys among all paediatric consultants and paediatric orthopaedic surgeons through British Paediatric Surveillance Unit and British Society for Children’s Orthopaedic Surgery to identify children and young people (<16 years) newly diagnosed with CRMO/CNO between October 2020 and November 2022. Standardised questionnaires were sent to reporting clinicians to collect detailed clinical information about each case within a few months of diagnosis (baseline) and one-year post-diagnosis.

**Results:**

Over the initial 25-month surveillance period, 185 children and young people with CRMO/CNO were identified. One-year follow-up questionnaires were received from 120/185 (65%) cases. Disease courses within the first year were categorised by the primary clinicians caring for the patients as continuous (23/113; 20%), recurrent (52/113; 46%) or a stand-alone episode (38/117; 34%). Disease control compared to baseline was reported to have improved in 104/115 (90%) of cases, with 47/115 (41%) of patients in remission. Overall, at one-year, 38/107 (36%) were not on any medication (including analgesics), and 34/107 (32%) were on NSAID alone (with or without other analgesics). There was a trend towards improved mobility, though this was not statistically significant. Reported complications at one-year follow-up included pathological fractures (1/113; 0.88%) and bone deformity (4/113; 3.5%).

There was no difference in outcome between patients treated with pamidronate (n = 30) or zoledronate (n = 21). Multi-logistic regression analysis did not identify any presenting features that were significantly associated with better or worse one-year outcome.

**Conclusion:**

The majority of children diagnosed with CRMO/CNO have disease relatively well controlled one-year post-diagnosis, as evidenced by clinician’s perspective on disease control and the majority of patients treated only with NSAIDs, or no medication. A trend towards functional improvement was observed.

**Patient Consent**

Not applicable (there are no patient data)

**Disclosure of Interest**

None Declare

### Bone in rheumatic diseases

#### P092 Heterotopic ossification - new competencies for pediatric rheumatologists

##### Irina Nikishina^1^, Svetlana Arsenyeva^1^, Valeria Matkava^1^, Aliya Arefieva^1^, Artem Borovikov^2^

###### ^1^Paediatric, V.A. Nasonova Research Institute of Rheumatology; ^2^Research Centre for Medical Genetics, Moscow, Russian Federation

####### **Correspondence:** Irina Nikishina

*Pediatric Rheumatology 2024*, **22(2):**PReS24-ABS-1503

**Introduction:** Нeterotopic ossification (HO) is the process of pathological bone formation in soft tissues and divided into two categories: non-genetic and inherited genetic forms (fibrodysplasia ossificans progressive (FOP) and Progressive osseous heteroplasia (POH)).

**Objectives:** To describe variety of patients (pts) with HO and analyzed the main clinical features of FOP and POH.

**Methods:** From 1998 to 2024, we identified 54 pts with FOP, 8 - POH and 18 - acquired forms of HO. All pts met typical clinical features of FOP/POH, mutations in the ACVR1/GNAS genes respectively.

**Results:** In all 54 FOP pts (28 male/26 female) the diagnosis was verified by “classic” phenotype: malformed great toes - 54/100%pts; malformed thumbs-22/40%; peripheral osteochondromas, abnormalities of the cervical spine - 54/100% and HO – 53/98%. Genetic test (ACVR1) was done in 45/%83 pts and confirmed typical or ultra-rare mutation. We found a lot of similarities with rheumatic diseases such as synovitis, sacroiliitis, patterns of bone ankylosis, which allowed us to implement some antirheumatic drugs (glucocorticoids, NSAIDs, bisphosphonates and tofacitinib). Other group with genetic-related HO included 8 pts with POH (5 male/3 female) and characterized by superficial and deep HO that begins in dermis and extended into skeletal muscles. The average manifestation age was 17.8 months (from 0 weeks to 4 years). All pts had diagnostic delay from 1,5 to 20 years. Most pts were misdiagnosed with calcinosis and even dermatomyositis instead of НO. Skin biopsy or surgical manipulations led to progression of POH in 5 pts. Unilateral disorder of extremities with loss of mobility detected in 5/63%pts, 3 of them had severe skin damage and limb shortening because of HO. Bilateral asymmetrical involvement with superficial HO was detected in 3/47% pts with no functional impairment. All pts had different GNAS mutations, 6 pts–de novo, in 2 family genetic test has not been performed yet. Non-hereditary HO included 18 pts with various causes of ossification: posttraumatic, inflammatory mostly and some cases of paralytic HO. One patient with HO was suffered from malignant tumor from the peripheral nerves of the left foot and left tibia.

**Conclusion:** It is important to establish the type of HO in order to choose the correct management strategy and avoid invasive diagnostic methods which can lead to progression of HO and severe disability. Early diagnosis and reposition of certain antirheumatic drugs may improve the prognosis for HO.

**Patient Consent**

Not applicable (there are no patient data)

**Disclosure of Interest**

None Declared

### Bone in rheumatic diseases

#### P093 Bone health in juvenile idiopathic arthritis compared to controls based on a norwegian observational study

##### Anette Lundestad^1,2^, Lena Cetrelli^1,3^, Oskar W. Angenete^4,5^, Thomas A. Augdal^6,7^, Karin Tylleskär^8^, Ellen Nordal^9,10^, Karen Rosendahl^9,11^, Gry B. Hoftun^1,2^, Mari Hoff^12,13^, Pål R. Romunstad^14^, Marite Rygg^1,2^

###### ^1^Department of Clinical and Molecular Medicine (IKOM), NTNU - Norwegian University of Science and Technology; ^2^Department of Pediatrics, St.Olavs Hospital; ^3^Center for Oral Health Services and Research, Mid-Norway (TkMidt); ^4^The Department of Circulation and Medical Imaging (ISB), NTNU - Norwegian University of Science and Technology; ^5^Department of Radiology and Nuclear Medicine, St.Olavs Hospital, Trondheim; ^6^Section of Paediatric Radiology, UiT The Artic University of Norway; ^7^Department of Clinical Medicine, University Hospital of North Norway(UNN), Tromsø; ^8^Child and Youth Clinic, Haukeland University Hospital , Bergen; ^9^Department of Clinical Medicine, UiT The Artic University of Norway; ^10^Department of Pediatrics; ^11^Section of Paediatric Radiology, University Hospital of North Norway(UNN), Tromsø; ^12^Department of Neuromedicine and Movement Science (INB), NTNU - Norwegian University of Science and Technology; ^13^Department of Rheumatology, St.Olavs Hospital; ^14^Department of Public Health and Nursing (ISM), NTNU - Norwegian University of Science and Technology, Trondheim , Norway

####### **Correspondence:** Anette Lundestad

*Pediatric Rheumatology 2024*, **22(2):**PReS24-ABS-1165

**Introduction:** Little is known about bone health in children with juvenile idiopathic arthritis (JIA) in the era of biologic treatment. Studies on factors affecting bone health and long-term outcomes among children with JIA are needed.

**Objectives:** To increase our understanding of bone health in children with JIA, and to identify risk factors for reduced bone health in order to facilitate intervention strategies.

**Methods:** In the NorJIA study https://norjia.com, 205 Norwegian children with JIA according to The International League of Associations for Rheumatology (ILAR) criteria (1), and age- and sex-matched controls from the general population participated in a multi-center, longitudinal cohort study. Clinical examination, validated patient/parent-reported questionnaires, imaging, and blood tests were performed. Physical activity was reported according to WHO definition. Bone mineral density (BMD) was measured with dual-energy X-ray absorptiometry (DXA). The lumbar spine (L1-L4), femoral neck, total hip, total body less head (TBLH) were measured in all study participants in accordance with the International Society for Clinical Densitometry (ISCD) pediatric official position (2). BMD Z-scores were adjusted for bone age. We used standard descriptive statistics and t-tests.

**Results:** Median age of the 205 children with JIA (60% girls) was 15 (IQR 12-17) years, with similar figures in the control group. Median disease duration was 7 years, 51% had used or were currently using biologic disease-modifying anti-rheumatic drugs (bDMARDs), and 26% had ever used systemic steroids. The JIA group tended to have slightly lower BMD Z-scores compared to the controls, but the difference was minor, and no substantial difference was found. Mean BMD Z-score, L1-L4, in JIA were 0.0 (95% CI -0.1 to 0.1) and in controls 0.1 (95% CI -0.1 to 0.3). Low education, being underweight or having low serum 25-OH-vitamin D levels were associated with slightly reduced BMD Z-scores in the JIA group. Among the controls, the reduced score was observed only in those who were underweight. We found a clear pattern of decreasing BMD with decreasing levels of physical activity. In children with JIA, mean BMD Z-score, L1-L4, was -0.3 (95% CI -0.6 to 0.0) in the low physical activity group and 0.2 (95% CI 0.0 to 0.4) in those with high activity. A similar trend was observed in controls. We also found that children with JIA were as physically active as the control group.

**Conclusion:** In this study on bone health, we found no substantial differences in BMD Z-scores in JIA versus controls, but a clear pattern of decreasing BMD Z-scores with decreasing physical activity levels. Our results underline the importance of physical activity to optimize bone health in children with JIA.

**Trial registration identifying number:** https://clinicaltrials.gov/ Identifier: NCT03904459

**Patient Consent**

Not applicable (there are no patient data)

**Disclosure of Interest**

None Declared

**References**

1. Petty RE, Southwood TR, Manners P, Baum J, Glass DN, Goldenberg J, et al. International League of Associations for Rheumatology classification of juvenile idiopathic arthritis: second revision, Edmonton, 2001. J Rheumatol. 2004;31(2):390-2.
2. Crabtree NJ, Arabi A, Bachrach LK, Fewtrell M, El-Hajj Fuleihan G, Kecskemethy HH, et al. Dual-energy X-ray absorptiometry interpretation and reporting in children and adolescents: the revised 2013 ISCD Pediatric Official Positions. J Clin Densitom. 2014;17(2):225-42.

### Bone in rheumatic diseases

#### P094 Evaluation of health literacy levels and illness perceptions of chronic recurrent multifocal osteomyelitis patients and their parents

##### Emine Özçelik, Vildan Güngörer, Didem Öztürk, Banu Çelikel Acar

###### Pediatric Rheumatology, Bilkent City Hospital , Ankara, Türkiye

####### **Correspondence:** Emine Özçelik

*Pediatric Rheumatology 2024*, **22(2):**PReS24-ABS-1254

**Introduction:** Chronic recurrent multifocal osteomyelitis (CRMO) is a chronic, non-bacterial, autoinflammatory bone disease. During the diagnostic phase of the disease, patients may experience an exhausting process, including multiple imaging, radiation exposure, prolonged antibiotic treatment and bone biopsies.

**Objectives:** The aim of this study was to evaluate the illness perceptions of CRMO patients and their parents according to their sociodemographic characteristics and health literacy (HL) levels.

**Methods:** Twenty-nine patients aged <18 years who were followed up for at least 6 months with a diagnosis of CRMO between January 2018 and January 2024 and their parents (mother/father) were included in the study. The brief illness perception questionnaire (IPQ) developed by Broadbent et al. was used to assess illness perception. Health literacy of the patients was assessed with the 10-question health literacy scale for school-age children. Turkey Health Literacy Scale was used to assess the health literacy level of parents.

**Results:** Twenty (69%) of 29 CRMO patients were male. HL level was moderate in CRMO patients (n=22, 75.9%). There was a moderately significant negative correlation between total IPQ score and HL score (r=-0.426 p=0.021). It was shown that as patients’ HL levels increased, their personal control over the disease was higher (r=0.404, p=0.030). A similar relationship was found between HL level and disease control with treatment (r=0.444, p=0.016). The concern score of patients who used the internet as a source of health-related information was higher than those who received information from their physicians (p=0.026). The HL level of the parents was found to be “problematic-limited” most frequently (n=13, 44.8%). The total IPQ score of parents with excellent HL level was found to be lower than those with problematic limited and inadequate HL level (p=0.037, p=0.017, respectively). Parents with inadequate HL thought that their children experienced the symptoms of the disease more severely than with excellent HL (p=0.040). The concern score was significantly higher in the problematic-limited and inadequate group compared to those with excellent HL (p=0.047 and p=0.042, respectively). There was a high positive correlation between the total illness perception score of patients and their relatives (r=0.748, p<0.01). Patients’ illness perception was compared with their parents’ illness perception, concern and understandability of the disease were significantly higher in parents (p=0.016 and p=0.028).

**Conclusion:** Improving health literacy can help patients with chronic diseases such as CRMO to see their disease as less threatening. It can help reduce parents’ concerns by improving their disease management.

**Date of birth::** janvier 10

**Patient Consent**

Not applicable (there are no patient data)

**Disclosure of Interest**

None Declared

**References**

1. Broadbent E, Petrie KJ, Main J, Weinman J. The brief illness perception questionnaire. J Psychosom Res. 2006 Jun;60(6):631-7.
2. Paakkari O, Torppa M, Kannas L, Paakkari L. Subjective health literacy: Development of a brief instrument for school-aged children. Scand J Public Health. 2016 Dec;44(8):751-757.
3. Haney MO. Psychometric testing of the Turkish version of the Health Literacy for School-Aged Children Scale. J Child Health Care. 2018 Mar;22(1):97-107.

### Bone in rheumatic diseases

#### P095 Bone microbiom in children with different forms of osteomyelitis

##### Veronika Petukhova^1^, Mikhail Kostik^2^, Alexei Maletin^1^, Lavrentii Danilov^3^, Vasilisa Dudurich^4^, Oleg Glotov^5^, Lubov Sorokina^2^, Eugenia Isupova^2^, Alexander Mushkin^1,6^

###### ^1^Saint-Petersburg Research Institute of Phthisiopulmonology; ^2^Saint-Petersburg State Pediatric Medical University; ^3^Department Saint-Petersburg State University; ^4^Cerbalab Laboratory; ^5^Pediatric Research and Clinical Center for Infectious Diseases; ^6^First Saint-Petersburg State Medical University n.a. academician Pavlov, Saint-Petersburg, Russian Federation

####### **Correspondence:** Mikhail Kostik

*Pediatric Rheumatology 2024*, **22(2):**PReS24-ABS-1563

**Introduction:** Bone inflammatory diseases are the heterogenous group with infection and non-infection etiology. The infection osteomyelitis are hematogenous osteomyelitis (HO) and tuberculosis osteomyelitis (TBO) and chronic nonbacterial osteomyelitis (CNO) is an immune-mediated disease with unknown etiology with possible combination of environmental, including low-virulent infection and genetic factors interaction, despite the name “nonbacterial”. Some previous studies suspected some bacteria, e.g. Propionobacteria as a possible ethiological factor for the CNO.

**Objectives:** The study aimed to evaluate the differences of bone mircobiom in children with bone inflammatory diseases.

**Methods:** In the prospective study we included children with CNO (n=13), TBO (n=21) and HO (n=5). The diagnosis of CNO was made with 2007 and 2016 criteria with negative culture from the bone; TBO was confirmed by culture and/or tissue PCR and HO according acute clinical course, high CRP, positive culture (St.aureus in all cases) and positive effect of antibiotics. In all cases the total DNA was isolated during diagnostic bone biopsy or bone surgery and was subjected to homogenization in a lysing solution. Homogenization was carried out together with the beads, followed by DNA extraction by the sorbent column method (Qiagen, Germantown, MD, USA) in accordance with the manufacturer’s recommendations.

Libraries for 16S DNA sequencing were prepared in accordance with the Illumina protocol for the preparation of 16S metagenomic libraries for sequencing (Part #15044223 Rev. B). Identification of bacterial species was performed using the exact matching algorithm in DADA2 using SILVA v138 sequences pre-processed in an appropriate way using the custom scripts. To identify special taxa for each group, sparse partial least squares discriminant analysis (sPLS-DA) was carried out with the “multiomix” software (mixOmics software package) in the R programming language.

**Results:** The CNO group is characterized by an increase in such genera of bacteria as Halomonas, Anaerobacillus, Bacillus, especially Acinetobacter ursingii, Pseudomonas aeruginosa, Acinetobacter haemolyticus. The TBO group is characterized by a decrease in such bacterial species as *Acinetobacter baumannii, Sphingomonas insulae, Stenotrophomonas maltophilia.* The HO group is characterized by a decrease in such genera of bacteria Serratia, Bosea, Nissabacter and decrease of *Dermacoccus nishinomiyaensis, Sphingobacterium cladoniae, Pseudomonas mendocina*.

**Conclusion:** Children with bone inflammatory disease have unique bone bacterial microbiom profile. Further investigations are needed.

**Patient Consent**

Not applicable (there are no patient data)

**Disclosure of Interest**

None Declared

### Bone in rheumatic diseases

#### P096 Rare case of progressive myositis ossificans circumscripta

##### Rodrigo Suárez

###### Pediatric Rheumatology, CHPR, Montevideo, Uruguay

*Pediatric Rheumatology 2024*, **22(2):**PReS24-ABS-1114

**Introduction:** Heterotopic ossification is a very rare pathology, most frequently self-limited and following trauma. When ossification progresses over time we should think about Progressive osseous heteroplasia (POH) and Fibrodysplasia ossificans progressiva (FOP). FOP is a genetic condition (mutation in activin receptor IA) characterized by progressive heterotopic ossification in specific anatomic patterns.

Most cases of POH are caused by mutations in the GNAS gene, starts in the dermis, and progresses into the deep tissue.

**Objectives:** Report the case of a patient with an ultra-rare entity that causes progressive soft tissue ossification, its diagnostic process and clinical evolution.

**Methods:** Report of a case with follow-up for 6 years.

**Results:** The parents of a 2 years and 4 months old boy, previously healthy, noticed limited mobility with a fixed flexion at 100°, and a bulk in the right elbow.

Without clear trauma, he did not have fever or any other systemic or organ-specific symptoms.

Radiographs show a calcified mass at humerus-ulnar joint.

Bone scintigraphy shows an abnormal increased uptake in the lower third of the right humeral diaphysis, and magnetic resonance confirm a exophytic osteochondral bulk that originates in the proximal ulna and compromises the humeroulnar joint, with edema of bone, and adjacent soft tissues.

Biopsy: reactive bone process.

The tumor increased twice its original size in the subsequent months and the elbow remained rigid at 90 degrees.

After trauma we tried treatment with ibuprofen and pamidronate, 0.1 mg/kg weight for three consecutive days, without results.

Secuencing of the GNAS gene to rule out POH, and ACVR1 gene to rule out FOP did not show pathogenic variants.

In 2022 consultation with Frederick S. Kaplan, M.D. concludes that due to the very strange ossification pattern, the evolution, and the genetic results, rule out FOP and POH, and corresponding a circumscribed myositis, POH like of unknown cause.

A Positron Emission Tomography was carried out in 2022 that showns a extensive calcified process in the soft tissues without hypermetabolism that suggests inflammatory activity.

**Conclusion:** We present a case of an ultra-rare pathology, which involved a complex diagnostic process. Initially the challenge was to rule out malignant causes, and those amenable to medical intervention, and in the evolution, the decision of whether or not to perform an excision procedure, which could cause thrust and increase in heterotopic ossification.

**Patient Consent**

Yes, I received consent

**Disclosure of Interest**

None Declared

**Reference**

1. Kaplan FS, Al Mukaddam M, Baujat G, Brown M, Cali A, Cho T-J, Crowe C, De Cunto C, Delai P, International Clinical Council on FOP (ICC) & Consultants: THE MEDICAL MANAGEMENT OF FIBRODYSPLASIA OSSIFICANS PROGRESSIVA: CURRENT TREATMENT CONSIDERATIONS.

### Bone in rheumatic diseases

#### P097 The similarities between transient synovitis of the hip and Perthes’ disease

##### Rinat Raupov^1,2^, Segei Vissarionov^1^, Pavel Bortulev^1^, Inna Morozova^2^, Alina Trishkina^2^, Angelina Tsyban^2^, Mikhail Kostik^2^

###### ^1^H. Turner National Medical Research Center for Children’s Orthopedics and Trauma Surgery; ^2^Saint-Petersburg State Pediatric Medical University, Saint-Petersburg, Russian Federation

####### **Correspondence:** Mikhail Kostik

*Pediatric Rheumatology 2024*, **22(2):**PReS24-ABS-1233

**Introduction:** transient synovitis of the hip (TSH) is one of the most common condition affecting joints in children. Perthes’ disease (PD) usually starts with hip inflammation and mimicking TSH.

**Objectives:** to compare clinical features TSH and PD

**Methods:** 84 children (31 boys and 53 girls) with TSH and 73 patients (51 boys and 22 girls) with PD are included in the retrospective study. Clinical features, triggers of TSH, PD stage were assessed.

**Results:** Girls are affected frequently 63% vs 30% (p=0.001) with younger onset age 5.0 (3.0; 7.0) vs 6.0 (5.0; 8.) years (p=0.002) in TSH compared PD. The clinical signs at onset were similar in both groups, TSH and PD: groin pain (51.2% vs 43.8%, p=0.357), knee pain (15.5% 11.0%, p=0.407), limping (54% vs 740, p=0.191). TSH was triggered by infection in 53.6 % of cases, by excessive physical activity in 14,3%. There’s no trigger factor in the remaining cases. The duration of the symptoms was 5 (2-10) days, 83/84 (99%) patients had unilateral hip involvement. 89% patients had self-limited course with improvement, 7 % had relapse and 3 % transformed to other diagnosis (2 patients to Perthes’ disease and 1 patient to juvenile idiopathic arthritis). Mean duration to PD diagnosis was 2 (0-7) month. Four (5.5%) patients had 1^st^ stage, 36 (49,3%) had 2^nd^ stage, 21 (28.8%) had 3^rd^ stage and 12 (16.4) had 4^th^ stage of PD. 22 (31%) of patients with PD were needed surgical treatment.

**Conclusion:** patients with TSH were younger and more often presented with girls than PD, while there’re no differences in clinical manifestations at onset.

**Patient Consent**

Not applicable (there are no patient data)

**Disclosure of Interest**

None Declared

### Bone in rheumatic diseases

#### P098 Chronic Nonbacterial Osteomyelitis (CNO) in a tertiary center children cohort of south Italy: treatment and outcome

##### Roberta Loconte^1^, Rossella Donghia^2^, Violetta Mastrorilli^1^, Fabio Cardinale^1^, Francesco La Torre^1^

###### ^1^Department of Pediatrics, Pediatric Rheumatology Center, Giovanni XXIII Pediatric Hospital, University of Bari, Bari; ^2^Gastroenterology, National Istituite Gastroenterology -IRCCS “Saverio de Bellis”, Castellana Grotte, Italy

####### **Correspondence:** Roberta Loconte

*Pediatric Rheumatology 2024*, **22(2):**PReS24-ABS-1576

**Introduction:** Outcome measures in Chronic Nonbacterial Osteomyelitis (CNO) are not yet standardized.

**Objectives:** The aim of this study was to correlate the outcome with the presence of symptoms, bone lesions detected on magnetic resonance imaging (MRI) and type of therapy carried out in a cohort of paediatric patients with CNO in a tertiary centre in South of Italy.

**Methods:** Retrospective data of CNO patients enrolled between January 2013 and October 2023 were collected. Imaging data were compared with clinical outcomes. The outcome measure was stratified into four groups with Delphy technique among 4 experts in the management of CNO, depending on the symptoms and the MRI bone lesions in comparison with class of the therapy: 1) Poor control on medication (in therapy, presence of symptoms and MRI bone lesions); 2) Control on medication (in therapy, absence of symptoms but presence of MRI bone lesions); 3) Control off medication (without therapy, absence of symptoms but presence of MRI bone lesions); 4) Remission off medication (without therapy, absence of symptoms and MRI bone lesions). Patients were treated according to a step-up treatment, based on “treat-to-target” strategy and response to therapy: nonsteroidal anti-inflammatory drugs (NSAIDs) (ibuprofen or naproxen) as first line treatment, bisphosphonates (pamidronate) as second line started after 1 month of first-line therapy in case of poor response or together with NSAIDs at diagnosis if spinal involvement was present, disease modifying antirheumatic drugs (DMARDs) methotrexate (MTX) as third line if symptoms persisted 3 months after starting pamidronate or in case of relapse of the disease after this period, finally anti-TNF biologic (etanercept) as fourth-line if symptoms persisted after 3 months from the start of MTX or in case of disease recurrence during MTX.

**Results:** We enrolled 20 patients, 12 males and 8 females. All patients were treated with NSAIDs, 16 patients (80%) with pamidronate of which 2 (10%) already from the diagnosis due to vertebral involvement, 9 (45%) with MTX, 3 (15%) with etanercept, 1 (5 %) with prednisone for severe presentation at disease onset. At the outcome evaluation after one year of treatment (T12) with our step-up protocol, 21% (4 patients) achieved “remission off medication”, 26.3% (5 pts) achieved “control off medication”, 42.2% (8 pts) “control on medication” and only 10.5% (2 pts) “poor control”. 1 patient did not reach T12.

**Conclusion:** Our standardized step-up treatment approach was shown to be effective in disease management of CNO with disease control or remission in nearly 90% of patients 12 months after diagnosis.

**Patient Consent**

Yes, I received consent

**Disclosure of Interest**

None Declared

### Bone in rheumatic diseases

#### P099 Zoledronic acid use in a quaternary paediatric rheumatology centre: gosh experience

##### Ovgu Kul Cinar^1,2^, Qiong Wu^1^, Kalindi Rajani^1^, Mark Heathfield^3^, Marianna Shiafkou^3^, Yvonne Glackin^1^, Moira Cheung^3^, Alexander Chesover^3^, Sandrine Compeyrot-Lacassagne^1,2^

###### ^1^Paediatric Rheumatology; ^2^NIHR Biomedical Research Centre; ^3^Paediatric Endocrinology and Bone metabolism, Great Ormond Street Hospital for Children NHS Trust, London, United Kingdom

####### **Correspondence:** Ovgu Kul Cinar

*Pediatric Rheumatology 2024*, **22(2):**PReS24-ABS-1707

**Introduction:** Bisphosphonates are inhibitors of the osteoclastic function. Success with their earliest reported indication in children for osteogenesis imperfecta has led to their use in secondary osteoporosis in rheumatological diseases and inflammatory bone diseases besides other indications. Side effect profiles can vary, and acute phase response might be more pronounced in inflammatory conditions. Zoledronic acid (ZA) has been increasingly used in our department in replacement of pamidronate.

**Objectives:** In this study, we aimed to understand the side effect profile of ZA and necessity of post-infusion blood monitoring in paediatric rheumatology patients.

**Methods:** Paediatric rheumatology patients who received ZA infusion in a 6-month period were included. The study comprised two arms: 1. data collection on biochemical markers including pre- and post-infusion vitamin D levels, bone and kidney profile. 2. post-infusion patient and parent survey on the side effect profile. Mann-Whitney U test is used for the statistical analysis.

**Results:** Nine patients received a first dose of ZA, and one patient a second infusion. 8/9 (%89) were female, with a mean age of 11.11 years and a median age of 11.00 years (range: 8.0-15.0 years). Chronic recurrent multifocal osteomyelitis (CRMO) was the treatment indication for 6/9 (67%) and secondary osteoporosis for 3/9 (33%). 7/9 (78%) had a flu-like illness with fever, nausea, and muscle aches in the first few days after infusion. 6/9 (67%) experienced bone or joint pain within the first 2 weeks post-infusion. Although 3/9 (33%) patients described symptomatic hypocalcaemia, only 1/9 (11%) without any known risk factors and with normal pre-infusion vitamin D level [100 nmol/L (range: 50-150 nmol/L)] had confirmed hypocalcaemia [Ca: 1.75 mmol/L (range: 2.19-2.66)] on the post-infusion blood test and required hospital admission. 7/9 (78%) took the prescribed calcium supplementation for 7 days post-infusion. 2/9 (22%) had low vitamin D levels before infusion, and 6/9 (67%) were on vitamin D supplementation before infusion. There was no statistically significant difference between vitamin D, Ca^+2^, phosphate, ALP, urea, creatinine, and albumin levels pre and post-infusion (p>0.05).

**Conclusion:** ZA has been successfully used in paediatric rheumatology patients. The main indications in our cohort were CRMO and secondary osteoporosis. Checking renal functions, bone profile and vitamin D levels before infusion and prescribing calcium and vitamin D supplementation for at least 7 days post-infusion is important to avoid side effects. Acute phase response and flu-like symptoms were seen in the majority of our patients, therefore regular paracetamol use for 24-48 hours after first infusion might be required in paediatric rheumatology practice.

**Patient Consent**

Not applicable (there are no patient data)

**Disclosure of Interest**

None Declared

**Reference**

1. Cheung M, Raimann A, Semler O, Hogler W. Short term side effects of first bisphosphonate infusion in children with different underlying bone pathologies. Abstracts of the ESPE 61 Annual Society Meeting. Horm Res Paediatr25 September 2023; 96 (Suppl. 4): 1–643.

### Bone in rheumatic diseases

#### P100 Pre results of efficacy and safety of bisphosphonate therapy for avascular necrosis of the femoral head course in form of osteoarthritis in children

##### Aleksei Kozhevnikov^1,2^, Dmitrii Barsukov^1^, Pavel Bortulev^1^, Nina Pozdeeva^3^

###### ^1^H. Turner National Medical Research Center for Children’s Orthopedics and Trauma Surgery; ^2^St. Petersburg State Pediatric Medical University; ^3^State Budgetary Healthcare Institution Children’s Out-Patient Department №49, Saint-Petersburg, Russian Federation

####### **Correspondence:** Aleksei Kozhevnikov

*Pediatric Rheumatology 2024*, **22(2):**PReS24-ABS-1260

**Introduction:** Avascular necrosis of the femoral head (AVN) in children is a multifactorial joint disease with non-inflammatory nature. The disease occur in young and middle-aged children and proceeds through several stages characterized by typically clinical and instrumental picture. Some children occurs more aggressive course of disease in form of osteoarthritis (OA). OA characterized by JIA-like sings not fulfilled of the ILAR criteria. OA is associated with hyperactivity osteoclasts and use NSAIDs not efficacy. Bisphosphonate therapy (BT) has been shown to be effective in the treatment of adults with AVN.

**Objectives:** The aim of this study was evaluated the efficacy and safety of BT in children with avascular necrosis of the femoral head course in form of osteoarthritis (OA/AVN)*.*

**Methods:** The study included 18 children (83,3% girls) with active OA/AVN treated intravenous bisphosphonates. Aged median onset OA/AVN in children were 9,2 ± 2,4 years. The average follow-up of course OA/AVN before BT was 8 [6; 12] months. Control group was 20 children with active OA/AVN treated by standard care. The outcome measure was radiographic deformity index (DI) and hip inflammation MRI scoring system (HIMRISS) assessed at the first step 6 months, then 12 and 18 months following of start BT. We used ibandronic acid (IA) at 3-monthly doses of 1,0 mg at baseline children before 7 yr and 1,5 mg – older. The study included 5 consecutive infusions. VAS score was used to measure pain. Also were involved some laboratory blood inflammatory tests and markers of bone metabolisms.

**Results:** First results of efficacy BT were analyzed in 14 children with active OA/AVN which received three and more intravenous of IA. Post infusion-related reactions were revealed at 85,7% (12) children after first intravenous of IA and characterized by short-term fever and arthralgia or back pain. Relieve hip pain was noted in all children already after first infusion of IA. Reduce of synovitis by MR imaging and flam area of osteonecrosis of the femoral head were showed after third intravenous of IA in 78,5% (11) children and in 21,5% (3) - after fourth infusion. Course of active OA/AVN in children didn’t characterized elevated concentrations of serum IL6, TNF-alpha and CRP. The average ESR at onset OA/AVN were 9 [5;17] mm/h. ANA were positive in 64,3% (9) and deficiency of vitamin D were in *85,7%* (12) children.

**Conclusion:** Use of BT in children with OA/AVN had shown promise to treatment. Analysis of long-term results of efficacy IA treatment will allow indicated guidelines for BT in children with OA/AVN.

**Patient Consent**

Not applicable (there are no patient data)

**Disclosure of Interest**

None Declared

**References**

1. Liu N, Zheng C, Wang Q, Huang Z. Treatment of non-traumatic avascular necrosis of the femoral head (Review). Exp Ther Med. 2022 May;23(5):321.
2. Kumar V, Ali S, Verma V, Singh A. Do bisphosphonates alter the clinico-radiological profile of children with Perthes disease? A systematic review and meta-analysis. Eur Rev Med Pharmacol Sci. 2021;25(15):4875-4894.
3. Ren Y, Deng Z, Gokani V, et al. Anti-Interleukin-6 Therapy Decreases Hip Synovitis and Bone Resorption and Increases Bone Formation Following Ischemic Osteonecrosis of the Femoral Head. J Bone Miner Res. 2021;36(2):357-368.

### Bone in rheumatic diseases

#### P101 The use of pamidronate represents a new promising approach to bone restoration in the surgical treatment of tuberculous osteomyelitis

##### Veronika Petukhova^1^, Alexander Mushkin^1, 2^, Tatiana Vinogradova^1^, Mikhail Kostik^3^

###### ^1^Saint-Petersburg Research Institute of Phthisiopulmonology; ^2^First Saint-Petersburg State Medical University n.a. academician Pavlov; ^3^Saint-Petersburg State Pediatric Medical University, Saint-Petersburg, Russian Federation

####### **Correspondence:** Mikhail Kostik

*Pediatric Rheumatology 2024*, **22(2):**PReS24-ABS-1376

**Introduction:** Bisphosphonates showed good efficacy in the treatment of patients with chronic non-bacterial osteomyelitis, but their place in the management of infectious osteomyelitis is not clear and approved.

**Objectives:** To validate the efficacy of pamidronic acid in bone restoration during the surgical treatment of experimental tuberculous osteomyelitis **(**TB-OM).

**Methods:** Clinical, radiological, morphological, and morphometric characteristics were studied using an experimental TB-OM model of the femur condyle (M. tub. H37Rv) in 21 rabbits. The rabbits were divided into four groups: control group (no treatment), group 2-4 underwent radical surgical removal of the focus and implant plasty with 2DBM, supplemented as follows: group 2-anti-TB drugs, group 3-anti-TB drugs and a single injection of PAM 1 mg/kg, group 4-only PAM. Autopsy was conducted at 3 and 6 months post-surgery to assess implantation zone features, bone tissue area, bone beam thickness, osteoblasts, osteocytes, osteoclasts, and their active forms.

**Results:** The use of PAM: a) aided implant adaptation by preventing premature lysis (CT detection of the implant in 85.7 - 100% of cases in groups 3 and 4 after 3 and 6 months, 50% in group 2 after 3 months; 0% after 6 months); and maintaining its osteoconductive and osteoinductive properties (implant ingrowth by bone beams in 100% of cases in groups 3 and 4 according to CT and morphology (fig. 1), formation of stromal cords and numerous growing bone beams around the implant); b) demonstrated safety in the postoperative period, did not increase TB-OM progression risk, and was compatible with anti-TB drugs (no adverse reactions, deaths, reduced specific inflammation in 50-75% of cases in groups 2-4 during the experiment, p<0.05); c) prolonged induction of osteogenesis (increase in active osteoblast proportion in perifocal zones by 6 months in groups 3 and 4, p<0.05); e) led to increased bone tissue area and bone beam thickness (group 4 showed maximum values of % of bone tissue area in the epiphysis and beam thickness after 3 and 6 months, groups 3 and 4 showed higher beam thickness values compared to group 2 after 3 months, p<0.05).

**Conclusion:** Experimental evidence supports the effectiveness of pamidronic acid in the surgical management of tuberculous osteomyelitis.

**Patient Consent**

Not applicable (there are no patient data)

**Disclosure of Interest**

None Declared

### Bone in rheumatic diseases

#### P102 Factors affecting the diagnostic process of chronic recurrent multifocal osteomyelitis patients

##### Mehveş Işıklar Ekici, Yasemin Uğur Es, Elif Çelikel, Banu Çelikel Acar

###### Pediatric Rheumatology, Ankara Bilkent City Hospital, Ankara, Türkiye

####### **Correspondence:** Mehveş Işıklar Ekici

*Pediatric Rheumatology 2024*, **22(2):**PReS24-ABS-1444

**Introduction:** Chronic recurrent multifocal osteomyelitis (CRMO) is a chronic autoinflammatory disease that is more common in children. It presents with swelling, local tenderness, bone pain in the affected areas. Non-specific symptoms can be attributed to growing pains, infection or malignancy, which can delay diagnosis for days or even years.

**Objectives:** The aim of this study was to evaluate the time from symptom onset to diagnosis in patients with CRMO and to determine the factors affecting the diagnostic process.

**Methods:** This study was conducted with the patients who were followed up in our hospital with the diagnoses of CRMO between 2016 and 2023. Patients who were not diagnosed in our center and whose follow-up period was <6 months were excluded. The patients’ data were retrospectively analyzed. In order to evaluate the factors affecting the diagnostic process, patients were grouped as those diagnosed in less than 6 months and those diagnosed in a longer period.

**Results:** The study included 32 patients, 23 (72%) of whom were male. The mean age at admission to the rheumatology outpatient clinic was 11,8±3,3, the mean age at diagnosis of CRMO was 12,1±1,2 years. Thirteen patients (40%) had multiple physician visits before being referred to rheumatology. The mean duration of symptoms at presentation was 12,3±15,1 months, the mean time from symptom to diagnosis was 13,3±15,2 months. At admission, all patients had arthralgia and 9 (28%) had a history of arthritis. The mean number of symptomatic sites was 2,2±0,1 and the findings were multifocal in 25 (78%) and symmetrical in 13 (40%) patients. MRI revealed bone marrow edema in the affected areas in all patients. MRI findings were multifocal in all and symmetrical in 23 (72%) patients. Twenty-six (81%) patients were consulted to the Department of Oncology for differential diagnosis, 20 (62%) patients required bone marrow aspiration, and 9 (28%) patients were followed up with suspected infection. Two patients were referred to orthopedics and bone biopsy was performed. One patient was consulted to the Department of Neurology with the suspicion of neuropathic pain. When the clinical and laboratory parameters of the patients were compared between patients with a duration of <6 months and >6 months until the diagnosis of CRMO, no significant difference was observed. In 4 of 17 (53%) patients with a duration of >6 months until the diagnosis of CRMO, it was observed that the family did not consult a doctor by ignoring the complaints, 1 patient had non-compliance with follow-up, 2 patients required time for further investigations to rule out a differential diagnosis after rheumatology referral, and 10 patients were followed up with different diagnoses and were not referred to the rheumatology department. After the referral to the rheumatology department, the examinations performed for differential diagnosis affected the process by an average of 1,1±0,3 months.

**Conclusion:** The most important factor affecting the diagnostic process of CRMO is the lack of recognition and misdiagnosis of the disease. Considering that the time from the patient’s admission to the rheumatology physician to the diagnosis is short, the importance of raising awareness about the disease emerges.

**Date of birth:** 04.01.1992

**Patient Consent**

No, I have not received consent

**Disclosure of Interest**

None Declared

### Bone in rheumatic diseases

#### P103 Chronic recurrent multifocal osteomyelitis: an atypical presentation with acute panniculitis and recurrent fever

##### Catherine Zhu^1^, Linda Rossi-Semerano^2^, Annonciade Frassati-Biaggi^3^, Marie-Chantal Morcelet^3^, Maryam Piram^4^

###### ^1^Faculty of Medicine, McGill University, Montreal, Canada; ^2^Service de Rhumatologie pédiatrique, Centre de référence des maladies auto-inflammatoires et de l’amylose (CEREMAIA), CHU de Bicêtre, Le Kremlin Bicêtre; ^3^Division of Dermatopathology, CHU de Bicêtre, Paris, France; ^4^Division of Pediatric Dermatology, CHU Sainte-Justine, Montreal, Canada

####### **Correspondence:** Linda Rossi-Semerano

*Pediatric Rheumatology 2024*, **22(2):**PReS24-ABS-1427

**Introduction:** Chronic recurrent multifocal osteomyelitis (CRMO) is a rare autoinflammatory bone disease primarily characterized by musculoskeletal symptoms, affecting 4 per 1 million children. Children with CRMO usually first present with bone pain and arthralgias. Diagnosis remains challenging due to the absence of widely accepted criteria, often necessitating exclusion of other conditions.

**Objectives:** We present a unique case of CRMO initially presenting with recurrent fevers and panniculitis, deviating from the typical pattern of bone pain.

**Results:** A 5-year-old girl, known for atopic dermatitis and asthma, presented with recurrent fevers and erythema nodosum-like red and painful nodules on her shins. She experienced recurrent febrile episodes over two years, accompanied by oral aphthous ulcers and lower limb arthralgias. Laboratory investigations revealed an inflammatory portrait with elevated c-reactive protein (53mg/L) and erythrocyte sedimentation rate (45mm/hr). Extensive workups, including infectious, autoimmune, and autoinflammatory investigations, were inconclusive. A skin biopsy revealed acute panniculitis. Treatment with colchicine alleviated cutaneous and febrile episodes but did not fully resolve bone pain. Subsequent whole-body MRI revealed inflammatory lesions affecting the metaphyseal regions of the upper limbs and lower limbs, mainly of the tibia, fibula and calcaneus finally confirming the diagnosis of CRMO. Non-steroidal anti-inflammatory drug (NSAID) therapy (naproxen 15mg/kg/day) was initiated, with attempts to taper colchicine resulting in panniculitis flare-ups. Thus, it was decided to keep the patient on dual therapy of colchicine and NSAIDs. After three years, the patient’s symptoms remain controlled with daily colchicine and naproxen during flare-ups.

**Conclusion:** This case highlights an atypical presentation of CRMO with panniculitis and recurrent fevers, effectively managed with colchicine. It underscores the importance of considering CRMO in children with unusual initial symptoms, emphasizing the need for heightened clinical suspicion to facilitate early diagnosis and appropriate management.

**Date of birth::** octobre 25

**Patient Consent**

Yes, I received consent

**Disclosure of Interest**

None Declared

### Bone in rheumatic diseases

#### P104 Trichorhinophalangeal syndrome type I; case report with a novel mutation

##### Reima Bakry^1^, Feras K. Olayan^2^

###### ^1^Pediatric; ^2^Radiology, Maternity and Children Specialized Hospital, Jeddah, Saudi Arabia

####### **Correspondence:** Reima Bakry

*Pediatric Rheumatology 2024*, **22(2):**PReS24-ABS-1488

**Introduction:** Trichorhinophalangeal syndrome (TRPS) is a rare autosomal dominant skeletal dysplasia caused by defects involving the TRPS1 gene. Three types (TRPSs I, II, and III) have been described. The key clinical features of the syndrome include fine, slowly growing hair, a high frontal hairline, and rarefaction of the lateral eyebrows; craniofacial peculiarities with a typical pear-shaped nose, long and flat philtrum, thin upper lip, receding chin, and protruding ears; phalangeal cone-shaped epiphyses—resulting in brachydactyly or clinodactyly—and other orthopedic abnormalities, such as hip malformation and short stature. We hereby report a novel mutation of TRPS that has not been reported in the literature.

**Objectives:** To report a novel mutation in TRPS1 syndrome

**Results:** Case presentation:

This is a 10 years old girl, who presented to our pediatric rheumatology clinic complaining of pain in bilateral small joints of her hands. On systemic review, the Mother claimed the slow growth of hair and poor school performance compared to her siblings. Her parents are healthy and non-consanguineous, and she has an autistic brother and 2 healthy sisters. On clinical examination, she showed the weight of the 50^th^ centile and height of the 5^th^ centile. She had fair scanty hair, a pear-shaped nose, a flat philtrum, and a thin upper lip. Her joint examinations revealed tenderness over PIP and MCPs of both hands and both wrists. She also had minimal tenderness on hip rotation. The back examination was unremarkable. Lab values including CBC, LFT, RFT, Inflammatory markers, immunoglobulins, ANA, and ENA were unremarkable. Skeletal survey showed cone-shaped epiphysis of the proximal phalanges of both hands and feet, bone shortening of PIPs with minimal PIP joint subluxation, and bilateral symmetrical sacroiliac joints narrowing with increased sclerosis. WES revealed TRPS1 (NM_014112.5):c.929C>G (p.Ser310X), Chr8(GRCh37):g.116631396G>C, a heterozygous mutation, which is along with the phenotype presentation, considered as pathogenic mutation.

**Conclusion:** Clinical examinations remain the most important tool to identify those patients. There are none curative treatments for this syndrome. However, early diagnosis allows early orthopedic referral and intervention to prevent functional disabilities or chronic pain. Our molecular finding added one new mutation to the list of mutations in TRPS patients.

**Patient Consent**

Yes, I received consent

**Disclosure of Interest**

None Declared

### Bone in rheumatic diseases

#### P105 Goldbloom’s syndrome: not just a long bone disease

##### Roberta Loconte^1,2^, Chiara Cannata^2,3^, Maria Laura Cagnato^2^, Camilla Sembenini^2^, Andrea Leo^2^, Francesca Tirelli^2^, Alessandra Meneghel^2^, Francesco Zulian^2^

###### ^1^Department of Pediatrics, Pediatric Rheumatology Center, Giovanni XXIII Pediatric Hospital, University of Bari, Bari; ^2^Pediatric Rheumatology Unit, Department for Women and Child’s Health, University Hospital of Padua, Padua i; ^3^ProMISE Department “G. D’Alessandro”, AOUP “Paolo Giaccone”, University of Palermo, Palermo, Italy

####### **Correspondence:** Roberta Loconte

*Pediatric Rheumatology 2024*, **22(2):**PReS24-ABS-1780

**Introduction:** Goldbloom’s syndrome (GS) is a rare condition characterized by inflammatory periostitis presenting with fever, bone pain and constitutional symptoms ^[1]^ that may resemble an oncological disease ^[2]^. Etiopathogenesis is still unknown but a post-infectious process has been suggested ^[3]^. Laboratory tests show an increase of inflammatory markers and often dysproteinemia ^[3]^. Long bones are the most affected sites, with symmetrical involvement ^[4]^.

**Objectives:** Case report of GS involving the scalp.

**Results:** A previously healthy 11-year-old girl, presented with swelling on the 9^th^ right rib after accidental trauma during a dance lesson. Chest x-ray documented remodeling and lysis of the rib. PET-MRI demonstrated hypermetabolism of the rib so that a bone biopsy was done showing unspecific inflammatory alterations. Five months later she presented with recurrent fever episode since 3 months, associated with fatigue, loss of appetite, weight loss and dry cough. Blood tests showed: ESR 120 mm/h, CRP 153.6 mg/L, ASLO 2388 U/L, Hb 9.4 g/dL, MCV 67 fl, albumin 26 g/L, gamma-globulin 20%. Chest x-ray indicated diffuse periosteal sleeve thickening of the 9th rib. PET-MRI was repeated and showed a widespread hypermetabolism involving the periosteum of both the femurs, tibias, fibulas, radius, ulna, all the ribs, the scapulae and the temporal bone, without significant bone marrow edema. Blood test confirmed an increase of the inflammatory markers. GS was suspected and corticosteroid therapy was started, with rapid clinical and laboratory improvement. One month later, prednisone was tapered and then gradually stopped. A PET-MRI performed after 2 months demonstrated complete resolution. Follow-up is ongoing, and after 12 months the patient is still asymptomatic.

**Conclusion:** To the best of our knowledge this is the first case of GS with scalp involvement. The exclusive and very symmetrical involvement of the periosteum in multiple sites, along with the other clinical and laboratory features, allowed a prompt diagnosis. Based on our experience we suggest to consider GS in the differential diagnosis of multifocal inflammatory bone disease, above all when it showed a symmetrical distribution, in order to correctly address treatment.

**Patient Consent:**

Yes, I received consent

**Disclosure of Interest**:

None Declared

**References:**

1. Goldbloom RB, Stein PB, Eisen A, McSheffrey JB, Brown BS, Wiglesworth FW (1966) Idiopathic periosteal hyperostosis with dysproteinemia. A new clinical entity. N Engl J Med 274(16): 873–878.
2. Santos S, Estanqueiro P, Salgado M (2013) Goldbloom’s syndrome—a case report. Acta Reumatol Port 38(1):51–55
3. Cameron BJ, Laxer RM, Wilmot DM, Greenberg ML, Stein LD (1987) Idiopathic periosteal hyperostosis with dysproteinemia (Goldbloom’s syndrome): case report and review of the literature. Arthritis Rheum 30(11):1307–1312.
4. Gerscovich EO, Greenspan A, Lehman WB (1990) Idiopathic periosteal hyperostosis with dysproteinemia-Goldbloom’s syndrome. Pediatr Radiol 20(3):208–211.

### Bone in rheumatic diseases

#### P106 The successful Zoledronate treatment for patients with hematogenous osteomyelitis: the case series

##### Lubov Sorokina^1^, Eugenia Isupova^1^, Veronika Petukhova^2^, Alexei Maletin^2^, Alexander Mushkin^2,3^, Mikhail Kostik^1^

###### ^1^Saint-Petersburg State Pediatric Medical University; ^2^Saint-Petersburg Research Institute of Phthisiopulmonology; ^3^First Saint-Petersburg State Medical University n.a. academician Pavlov, Saint-Petersburg, Russian Federation

####### **Correspondence:** Mikhail Kostik

*Pediatric Rheumatology 2024*, **22(2):**PReS24-ABS-1373

**Introduction:** Hematogenous osteomyelitis (HO) is usually an acute disease, caused by different bacteria. The disease usually responds well to antibiotics, but in some cases, it may have a chronic course. In some localization (e.g. femoral head) of destructive foci the disease may have severe consequences that require further orthopedic surgeries and arthroplasty.

Several previous studies showed the bone-conserving effects in bisphosphonates which may stop/decrease the following bone destruction in different bone diseases (chronic non-bacterial osteomyelitis, bone malignancy, or bone metastases), and have anti-inflammatory effects. In the experimental studies, the positive effect of bisphosphonates in rabbit experimental tuberculosis model. The role of bisphosphonates in HO has not yet been studied before.

**Objectives:** Our study aimed to evaluate the safety and efficacy of bisphosphonates in the HO

**Results:** Five patients (3 boys and 2 girls) aged 3-13 years with chronic osteomyelitis as an outcome of acute HO became acutely ill, in the summer and autumn period, against the background of complete well-being. All patients had a septic course with bilateral pneumonia with respiratory failure, and all were treated in the ICU and had experience in massive broad-spectrum antibacterial therapy. Two patients developed femoral venous thrombosis. Four patients underwent surgical intervention for osteomyelitis. Three had proximal hip involvement with concomitant hip arthritis with flattering of the epiphysis and further femoral head subluxation development. One patient had the involvement of the distal femur metaphysis and pelvic bones, and one had humerus osteomyelitis.

All patients received 2-4 infusions of zoledronic acid (0.05-1 mg/kg/infusion every 6 months) with significant pain and radiological and functional improvement with discontinuation of the analgetics. One patient underwent hip arthroplasty and one is pending.

**Conclusion:** Bisphosphonates could be used in patients with HO. Studies about early bisphosphonate interventions in the acute stage are required.

**Patient Consent**

Not applicable (there are no patient data)

**Disclosure of Interest**:

None Declared

### Bone in rheumatic diseases

#### P107 Chronic recurrent multifocal osteomyelitis: case series from Western India

##### Jitendra S. Oswal, Deepti Agarwal

###### Bharati Vidyapeeth Medical College, Pune, India

####### **Correspondence:** Jitendra S. Oswal

*Pediatric Rheumatology 2024*, **22(2):**PReS24-ABS-1463

**Introduction:** Chronic recurrent multifocal osteomyelitis (CRMO) is a rare autoinflammatory disease of the bone. It can be sporadic or occur as part of monogenic conditions.

**Objectives:** To study the demographic, clinical, laboratory profile and treatment of CRMO in children.

**Methods:** This was a retrospective case record based study. Seven patients were diagnosed and treated as CRMO between years 2020 to 2023 at our tertiary care center. Records were reviewed for clinical features, investigations and treatment. Diagnosis of CRMO was made based on the history of insidious onset of bone pain/swelling and presence of radiologic evidence of multiple osteolytic lesions in the long bones. Jansson or Bristol criteria were used for classification of cases.

**Results:** Over three year period, seven patients were diagnosed as CRMO, out of which six were girls and one boy. (Table 1) Median age at the onset of symptoms was 9 years (range: 3 months to 12 years) and the median age at diagnosis was 9 years. Median time from onset of symptoms to diagnosis was 12 months (range: 1 month to 4 years). Pain with or without swelling was the most common symptom. One out of the seven children presented with low grade fever. The femur was the most commonly involved bone and was seen in 4/7 children. MRI was done in 6/7 children, all of which had features suggestive of osteomyelitis. The inflammatory markers were elevated in 5/5 children. The 6/7 had multifocal disease while one had recurrent disease. All children received NSAIDs. One patient each required methotrexate and bisphosphonate.

**Conclusion:** CRMO was seen predominantly seen in long bones and in girls. There is a significant delay in the diagnosis owing to the lack of awareness about the disease. Most of the children had multifocal disease. Early recognition and timely referral can help in better outcomes.

**Patient Consent:**

Yes, I received consent

**Disclosure of Interest**:

None Declared

### Bone in rheumatic diseases

#### P108 Algorithmic approach to arthropthy in children

##### Niloofar Shashaani^1^, Vadood Javadi Parvaneh^1^, Khosro Rahmani^1^, Reza Shiari^1^, Vahideh Zeinali^2^

###### ^1^Pediatrics; ^2^Shahid Beheshti University of Medical Sciences, Tehran , Iran, Islamic Republic Of

####### **Correspondence:** Vadood Javadi Parvaneh

*Pediatric Rheumatology 2024*, **22(2):**PReS24-ABS-1492

**Introduction:** In the routine daily pediatric rheumatology clinics, the clincian may encounter with situations which decision about chronic inflammatory arhtritis may be challenging. On the other hand, in some patients, it is not clear that the patient’s complaints is truely arthritis or it mimic arthritis. Furthermore, this issue is prominent when the physician treat the patient with the diagnosis of juvenile idiopathic arthritis , but the response is poor.

**Objectives:** This paper proposes an innovative algorithmic approach to streamline the diagnostic process and improve patient outcomes.

**Methods:** Our approach begins with a comprehensive clinical evaluation, followed by the application of a decision-making algorithm that incorporates key indicators such as patiient history, physical examination, and/or imaging techniques. The algorithm is designed to guide clinicians through a series of differntial diagnosesn leading to a more precise identification of the underlying cause of arthropathy.

**Results:** Our experience in Iran indicate this algorithmic approach can lead to a more precise diagnosis and avoid from mis diagnosis of some disorders which mimic arthritis including skeletal dysplasia instead of juvenile idiopathic arthrits. Ultimately, we can use more targeted treatment strategies which contribute to improved quality of life for children suffering from arthropathy.

**Conclusion:** This approach aims to stimulate discussion anf further focus and research into the use of algorithmic approaches in pediatric rheumatology, with the ultimate goal of inproving patient care and outcomes.

**Patient Consent:**

Not applicable (there are no patient data)

**Disclosure of Interest**:

None Declared

### COVID-19 (Coronavirus)

#### P109 Long-term outcome of patients with MIS-C in Slovenia

##### Nina Emeršič^1^, Mojca Zajc Avramovič^1^, David Gosar^2^, Anja Koren Jeverica^1^, Gašper Markelj^1^, Gorazd Mlakar^3^, Nataša Toplak^1, 4^, Tina Vesel Tajnšek^1^, Maša Bizjak^1^, Tadej Avčin^1, 4^

###### ^1^ Department of Allergology, Rheumatology and Clinical Immunology; ^2^Department of Child, Adolescent and Developmental Neurology; ^3^3Department of Cardiology, Children’s Hospital, University Medical Centre Ljubljana; ^4^Department of Pediatrics, Faculty of Medicine, University of Ljubljana, Ljubljana, Slovenia

####### **Correspondence:** Nina Emeršič

*Pediatric Rheumatology 2024*, **22(2):**PReS24-ABS-1367

**Introduction:** Multisystem inflammatory syndrome in children (MIS-C) is a serious complication of SARS-CoV-2 infection. Short-term outcome is mostly favourable, but less is known about the long-term sequelae.

**Objectives:** The objective of this study was to evaluate a long-term outcome of patients with MIS-C.

**Methods:** A cohort study included children up to 18 years of age diagnosed with MIS-C who were examined at Children’s hospital University Medical centre Ljubljana, Slovenia, between September 2020 and May 2022. Their possible organ involvement as well as neuropsychological and psychosocial status were regularly assessed and finally checked at the last follow-up visit 1,5-3 years after initial presentation. We used clinical, laboratory, imaging evaluations and neuropsychological tests battery measuring attention, executive function, memory and fine motor skills. Psychopathological symptoms were assessed using the Achenbach Child Behavior Checklist.

**Results:** Out of 78 enrolled patients, 4/78 (5%) reported non-specific symptoms at the final follow-up, such as headache and fatigue. We observed individual cases of weight gain, arterial hypertension, dyspnea, nightmares, nodules on the vocal cords and allergic rhinitis. Cardiac involvement was observed in 67/78 children (86%) at initial presentation, with residual changes in 5/78 (6%). No MIS-C recurrence was noted after SARS-CoV-2 reinfection or COVID-19 vaccination. 60/78 (77%) patients were included in neuropsychological and psychosocial assessment, in which we found cognitive deficits beyond the expected baseline levels specfic domains.

**Conclusion:** The long-term outcome of MIS-C in Slovenian patients was generally favourable, with no recorded deaths. Non-specific symptoms, residual cardiac changes and cognitive deficits and symptoms of depression were identified, but no recurrence of MIS-C was noted post-reinfection or COVID-19 vaccination.

**Patient Consent**

Yes, I received consent

**Disclosure of Interest**:

None Declared

### COVID-19 (Coronavirus)

#### P110 COVID19-related systemic lupus erythematosus in children: a single center study

##### Chiara Cannata^1^, Francesca Tirelli^2^, Camilla Sembenini^2^, Alessandra Meneghel^2^, Francesco Zulian^2^

###### ^1^Promise, University of Palermo, Palermo; ^2^Departement for Women and Child’s Health, Pediatric Rheumathology Unit, Padua, Italy

####### **Correspondence:** Chiara Cannata

*Pediatric Rheumatology 2024*, **22(2):**PReS24-ABS-1767

**Introduction:** It is well known that SARS-CoV-2 virus is a powerful inducer of autoimmune phenomena both during the disease and later on. Observations coming from our own clinical practice seem to highlight that Systemic Lupus Erythematosus that began during the SARS-CoV-2 pandemic (CR-SLE) is somehow different from those with onset in the pre-Covid period (pC-SLE).

**Objectives:** To compare the clinical characteristics of a cohort of patients with CR-SLE with a cohort of pC-SLE.

**Methods:** We performed a retrospective analysis of a prospective collection of data to compared demographic, clinical and laboratory features of pediatric patients with CR-SLE and pC-SLE at disease onset. Covid-19 correlation was defined as recent infection (< 8 weeks interval time), serology or significant recent family history for Covid.

**Results:** A total of 47 patients (10 CR-SLE, 37 pC-SLE) were included in the study. The mean age at presentation was 12.7 years in the CR-SLE group and 10.9 years in the cSLE group. At disease onset, the majority of patients in both groups presented with systemic and/or mucocutaneous symptoms. In the CR-SLE patients, musculoskeletal (30% vs 65%, *p=0.07*) and renal involvement (10% vs 59.5%, *p=0.010*) were significantly less common than pC-SLE. This different clinical picture correlates with the lower SLEDAI score in CR-SLE (mean 7.0 vs 17.5, *p<0.001*). Thrombocytopenia was significantly more frequent in the CR-SLE group (70% vs 21%, *p<0.05*), while low C3-C4 and high erythrocyte sedimentation rate (ESR) were rarer than in pC-SLE (20% vs 67-70%, *p<0.05*). Similarly, hematuria and proteinuria were present in only one CR-SLE patient (10%) as compared with 54-57% in the pC-SLE group (*p<0.05*). ANA antibodies (abs) were equally present in the vast majority of patients in both groups. Conversely, while anti-dsDNA abs were less frequent in CR-SLE (40% vs 67.6%), ACL abs and Lupus anticoagulant (LAC) were found with higher frequency in this group (70% vs 58.3 and 50% vs 30.4, respectively). In the CR-SLE cohort, hydroxychloroquine (HCQ) was more commonly used than in pC-SLE (70% vs 13.5%, *p<0.001*), whereas immunosuppressive (IS) drugs were more rarely used (20% vs 49%, *p<0.05*). At 12 month follow up, 6/10 CR-SLE patients were either on HCQ or off-therapy and only 2 on IS while 56.7% of the CR-SLE patients were still on IS treatment and only 2/30 off therapy.

**Conclusion:** CR-SLE represents a milder disease in childhood. The systemic symptoms with thrombocytopenia with autoantibodies typical of antiphospholipid syndrome and the rarity of renal involvement represent the peculiar clinical aspects. These, together with the need for less aggressive treatment and a better prognosis, represent distinguishing characteristics as compared to the classic pC-SLE. Very few pediatric SLE cases following COVID-19 have been reported so far. The possible role of SARS-COV-2 in triggering pediatric SLE in genetically susceptible individuals is conceivable as interferons are thought to play an important role in the development of autoimmune diseases after COVID-19 infection and an increased expression of IFN-α has been observed in many patients with SLE. Further studies are needed to confirm these preliminary results.

**Patient Consent:**

Yes, I received consent

**Disclosure of Interest**:

None Declared

### COVID-19 (Coronavirus)

#### P111 Post-COVID vascular syndrome: infectiously provoked microthromboangiopathy

##### Ulyana Lushchyk^1^, Viktor V. Novytskyy^2^, Viktor V. Novytskyy^1^, Diaa N. Moamar^3^, Serhiy O. Sazchenko^3^, Ivanna I. Legka^1^

###### ^1^Veritas Research Center; ^2^Institute of Mathematics of NAS of Ukraine; ^3^Clinic of Vascular Innovations, Kyiv, Ukraine

####### **Correspondence:** Ulyana Lushchyk

*Pediatric Rheumatology 2024*, **22(2):**PReS24-ABS-1202

**Introduction:** The Сovid pandemic of 2019-2022 became a challenge not only for world civilization, but also for the medical community, as it manifested itself in various forms of the mutated virus with different degrees of severity of the course of pathology and invisible subclinical consequences - complications in the cardiovascular and nervous systems.

**Objectives:** Therefore, the Covid problem, both the disease itself of a viral nature, and the disease state after suffering Covid require special attention of both researchers and clinicians.

**Methods:** We have carried out an analysis of vascular screening of microcirculatory and macrocirculatory changes in the vascular bed in patients of various ages - from 1-year-old children to elderly people during the years 2015-2023, who applied to the Clinic of Vascular Innovations for various reasons [1, 2].

We have examined 4,832 patients, 17% (822 patients) are children of preschool and school age, 25% (1,208) are young (25-45 years old), 51.5% (2,488) are middle-aged (46-65 years old), 6.3% (304) - senior citizens (66-90 years old), 0.2% (10) - long-lived (over 90 years old). Patients who were examined from 2015 to 2019 were the control group (2015 patients it is 41.7% of all patients), and patients who have been examined from 2020 to 2023, inclusive, are the C group. At the same time, the C group has been divided into 2 subgroups by year pandemic activity - C1 - 2020-2021 (1750 patients, which is 36.2% of all patients), C2 - 2022-2023 (4832-2015-1750 = 1067, which is 22% of all patients).

**Results:** As a result of the study, we established have a specific post-Covid vascular pattern of microthromboangiopathy, which appeared in the 2-3 weeks of the primary Covid disease at the level of the microcirculatory bed and gradually progressed to the level of visual microthrombi in the dynamic viscosity test and the appearance of stenosis patterns at the macrovascular level during a year and thereafter their progress in the absence of adequate treatment.

Structural pathological transformations of a rheumatoid nature and pathological uncontrolled neoangiogenesis, which we began to detect more often in groups C1 and C2, indicate a high risk of provocation of oncological and rheumatological diseases after suffering Covid.

Because of the conducted research, a new term “post-Covid vascular syndrome” has been introduced, which reflects microthromboangiopathy of the deep layers of the microcirculatory bed and subdecompensated perivascular edema of a draining, total, highly intense nature. These conditions pose a threat to a person’s life in critical situations, as they imperceptibly block microcirculation at a time when the patient experiences general asthenia in the background and complains of slowing of thoughts, deterioration of memory, sleep, dulling of emotions.

**Conclusion:** Our research shows that existing Covid-virus-induced occlusive microthromboangiopathies carry a high risk of cardiovascular catastrophes and require intensive angiocorrection to restore perfusion of organs and systems.

**Patient Consent:**

Yes, I received consent

**Disclosure of Interest**:

None Declared

**References:**

1. Lushchyk U: Device for the vascular screening: State Patent of Ukraine. No. 85052. 11.11.2013.
2. Lushchyk UB, Novytskyy VV et al. (2021). Vascular Screening of PathoNeoAngioOncogenesis. (Analytical Approach to an Early Diagnosis of Pathological ArterioVenous Angiotransformations at the Micro- and Macrovascular Levels). Journal of Blood Disorders, Symptoms & Treatments- 2021- Vol. 3.

### COVID-19 (Coronavirus)

#### P112 The protective effect of biological therapies on developing Multisystem Inflammatory Syndrome in Children (MIS-C): using big data in a large health care organization

##### Lana Khoury^1,2^, Adi Miller-Barmak^1,2^, Mohamad Hamad Saied^1,2,3^

###### ^1^Department of Pediatrics, Carmel Medical Center; ^2^Faculty of Medicine, Technion Israel Institute of Technology, Haifa, Israel; ^3^Department of Pediatric Immunology and Rheumatology, Wilhelmina Children’s Hospital, Utrecht, Netherlands

####### **Correspondence:** Mohamad Hamad Saied

*Pediatric Rheumatology 2024*, **22(2):**PReS24-ABS-1355

**Introduction:** Multisystem Inflammatory Syndrome in Children (MIS-C) is a severe, life threatening, complication arises weeks after acute COVID-19 infection, often presenting with fever and diverse systemic symptoms. Limited data exist on the effectiveness of biological therapies in preventing MIS-C development.

**Objectives:** To investigate whether biological therapy can prevent the occurrence of multisystem inflammatory syndrome in children (MIS-C) and adolescents.

**Methods:** We assessed the Clalit Health Services database, the largest health care organization (HCO) in Israel, data from 793,909 children aged 0 to 18 years who tested positive for COVID-19 were analyzed. The diagnosis of MIS-C was adjudicated using the case definition used by the Centers for Disease Control and Prevention. Patients receiving biological therapies were compared to a control group. We performed a Chis square analysis of the incidence of MIS-C in both groups following COVID-19 infection.

**Results:** Among 793,909 cases, 573 children received biological therapies, and 143 cases of MIS-C were identified. Notably, none of the individuals treated with biological therapies developed MIS-C. Logistic regression analysis adjusted for demographics showed no significant association between biological therapy and MIS-C development.

**Conclusion:** Our study highlights our hypothesis on the efficacy of biological treatments in preventing MIS-C. Although statistical significance was not achieved due to the absence of MIS-C cases in patients receiving biological therapies, our study shows a potential protective role for such therapies against MIS-C following COVID-19 infection in children. Further research, including prospective studies with larger cohorts, is warranted to confirm these findings and elucidate underlying mechanisms.

**Patient Consent**

Not applicable (there are no patient data)

**Disclosure of Interest**

None Declared

### COVID-19 (Coronavirus)

#### P113 Evaluation of long-term comorbidities and prognoses of children with COVID-19-associated multisystem inflammatory syndrome

##### Pelin Özçilingir Hakverdi^1^, Kübra Aykaç^2^, Yağmur Bayındır^3^, Seher Şener^3^, İlker Ertuğrul^4^, Özge H. Başaran^3^, Yelda Bilginer^3^, Tevfik Karagöz^4^, Yasemin Özsürekçi^2^, Seza Özen^3^

###### ^1^Department of Pediatrics; ^2^Department of Pediatric Infectious Diseases; ^3^Department of Pediatric Rheumatology; ^4^Department of Pediatric Cardiology, Hacettepe University, Ankara, Türkiye

####### **Correspondence:** Pelin Özçilingir Hakverdi

*Pediatric Rheumatology 2024*, **22(2):**PReS24-ABS-1468

**Introduction:** Multisystem inflammatory syndrome in children (MIS-C) is a life-threatening condition characterized by persistent fever, multi-organ involvement, and significant elevation of inflammatory markers following a SARS-CoV-2 infection (1).

**Objectives:** The objective of this study is to evaluate the follow-up and outcome of patients with MIS-C.

**Methods:** This is a single-center retrospective cohort study evaluating MIS-C patients (0-18yr) diagnosed between 2020 and 2023. At the time of diagnosis, at 6 weeks, and at 1 year follow-up, the following data of the patients were recorded: demographic characteristics, clinical and laboratory findings (including autoantibodies), echocardiography and other imaging results, treatments received, development of autoimmune disease and neurological sequelae and prognoses. In addition the follow-up was compared to that of 13 patients who experienced severe COVID pneumonia between 2020 and 2023.

**Results:** Of the 132 patients, 65,9% were male and 34,1% were female. The median age at diagnosis was 8.2 years (0,2-17,6). Prior to December 2021, 98 children were diagnosed with MIS-C (mostly associated with the delta variant), whereas following the emergence of the omicron variant (after December 2021), MIS-C was observed in a total of 34 patients. The most commonly observed symptoms at the time of diagnosis were fever (100%).At the time of diagnosis, almost all had lymphopenia (0.2-1.58), platelets were 180 x 10^3/μL (27-649), and almost all had high C-reactive protein (CRP) levels with a mean of 18.5 mg/dL (0.5-144.7). Abnormal liver function tests were detected in 57 patients (43.2%). Abnormal echocardiography findings were observed in 88 patients (67.2%) with an average ejection fraction was 65% (22-85).

During treatment, all patients received IV antibiotics, intravenous immunoglobulin (IVIG) and steroids, with 123 patients (93.2%) also receiving enoxaparin sodium, and 100 patients (75.8%) receiving aspirin. Anakinra therapy was administered to 82 patients (62.1%). The average duration of steroid therapy was 5 days (2-60), and the duration of anakinra therapy was also 5 days (2-21). Inotropic support was required by 44 patients (33.3%). Additionally, Therapeutic Plasma Exchange was performed in 30 patients (22.7%), and 4 patients (3%) underwent extracorporeal membrane oxygenation (ECMO). Clinical remission was achieved in 127 patients (96.2%), and 3 patients (2.3%) died (all before December 2021).

After discharge, sequelae were observed in 78 patients (82.1%). Decreased school performance (32.5%) and neurological symptoms were among the significant sequelae. Neurological symptoms ranging from headache to ocular myasthenia were detected in 15.8% of the patients. 60 patients had autoantibodies (ANA, anti-ds DNA, ENA, APS) (63.2%), and thyroid autoantibodies were positive in 29 patients (30.5%).

**Conclusion:** We were able to reach a high remission rate in our MIS-C patients. However at follow-up neurological symptoms were present in 15% and 2/3 had autoantibodies. This data suggests that MIS-C is a distinct immunopathogenic disease.

**Date of birth:** septembre

**Patient Consent**

Yes, I received consent

**Disclosure of Interest**

None Declared

**Reference**

1. Hoste L, Van Paemel R, Haerynck F. Multisystem inflammatory syndrome in children related to COVID-19: a systematic review. European Journal of Pediatrics. 2021;180(7):2019-34.

### COVID-19 (Coronavirus)

#### P114 Pediatric rheumatic diseases in the light of COVID-19 pandemic, a retrospective observational big data study

##### Rim Kasem Ali Sliman, Mohamad Hamad Saied

###### Pediatric Department, Carmel Medical Center, Haifa, Haifa, Israel

####### **Correspondence:** Rim Kasem Ali Sliman

*Pediatric Rheumatology 2024*, **22(2):**PReS24-ABS-1779

**Introduction:** Viral infections like SARS-CoV-2 have been implicated in triggering autoimmune diseases potentially through mechanisms such as molecular mimicry and bystander activation. COVID-19 patients exhibited autoantibodies and viral peptide similarities to human proteins, which could provoke autoimmune responses.

**Objectives:** To compare the incidence rate (IR) of different pediatric autoimmune rheumatic diseases over the last five years before, through, and after the COVID-19 pandemic.

**Methods:** An anonymous big data cohort analysis between 2019 and 2023 spanned nearly 1.5 million pediatric patients aged 0 to 18 years. The study focused on newly diagnosed cases of Juvenile Idiopathic Arthritis (JIA), Systemic Lupus Erythematosus (SLE), or Henoch-Schönlein Purpura (HSP).

**Results:** No statistical significance in IR change was noted over the years for JIA or SLE when comparing each year to the following year or the pre-pandemic year 2019. However, a statistically significant increase was observed for HSP when comparing 2021 to 2022 (P=0.01), while a trend was noted when comparing 2021 to 2019 (P=0.078).

**Conclusion:** The IR of these pediatric autoimmune diseases showed some fluctuations over the years, with no statistically significant changes except for HSP, which showed a significant increase in IR when comparing 2022 to 2021. Overall, the IR increased in the latter years of the study period. These findings suggest a potential association between the occurrence of these autoimmune diseases and factors or events that may have influenced their incidence rates, particularly in recent years, but further research is needed to establish a causal relationship

**Trial registration identifying number:** Helsinki Comitte Protocil Number : **0017-23-CMC**

**Date of birth:** mai 27, YY

**Patient Consent**

Not applicable (there are no patient data)

**Disclosure of Interest**

None Declared

**References**

1. Diagnosis C. New Onset of Autoimmune Diseases Following COVID-19 Diagnosis. 2021;1–19.
2. Hussein HM, Rahal EA. The role of viral infections in the development of autoimmune diseases. Vol. 45, Critical Reviews in Microbiology. Taylor and Francis Ltd; 2019. p. 394–412.
3. El-Hor N, Adams M. Pediatric Rheumatologic Effects of COVID-19. Pediatr Clin North Am. 2021;68(5):1011–27.
4. Freites Nuñez DD, Leon L, Mucientes A, Rodriguez-Rodriguez L, Font Urgelles J, Madrid García A, et al. Risk factors for hospital admissions related to COVID-19 in patients with autoimmune inflammatory rheumatic diseases. Ann Rheum Dis. 2020;79(11):1393–9.
5. City NY, Appendix S. C or r e sp ondence Covid-19 in Immune-Mediated Inflammatory Diseases — Case Series from New York. 2020;8–10.
6. Batu ED, Sener S, Ozen S. COVID-19 associated pediatric vasculitis: A systematic review and detailed analysis of the pathogenesis. Semin Arthritis Rheum. 2022;55(June):152047.
7. Caso F, Costa L, Ruscitti P, Navarini L, Del Puente A, Giacomelli R, et al. Could Sars-coronavirus-2 trigger autoimmune and/or autoinflammatory mechanisms in genetically predisposed subjects? Autoimmun Rev. 2020;19(5):2019–20.
8. Mohkhedkar M, Venigalla SSK, Janakiraman V. Untangling COVID-19 and autoimmunity: Identification of plausible targets suggests multi-organ involvement. Mol Immunol. 2021 Sep 1;137:105–13.
9. Tutal E, Ozaras R, Leblebicioglu H. Systematic review of COVID-19 and autoimmune thyroiditis. Travel Med Infect Dis. 2022;47(March):102314.

### COVID-19 (Coronavirus)

#### P115 Immune response towards SARS-COV-2 vaccination in children with previous multi-system inflammatory syndrome in children

##### Claire Butters^1^, Jonathan Day^1^, Hamza Van Der Ross^1^, Timothy Spracklen^1^, Ursula Rohlwink^2^, Muki Shey^2^, Kate Webb^1^

###### ^1^Paediatrics; ^2^Surgery, University of Cape Town, Cape Town, South Africa

####### **Correspondence:** Claire Butters

*Pediatric Rheumatology 2024*, **22(2):**PReS24-ABS-1523

**Introduction:** Multi-system inflammatory syndrome in children (MIS-C) is a rare, severe hyperinflammatory condition triggered by SARS-CoV-2 infection. SARS-CoV-2 vaccination in children with previous MIS-C appears safe, and we have previously shown that there is no difference in SARS-CoV-2 antibody production or neutralisation ability; however, the immune response elicited towards SARS-CoV-2 vaccination in these children is not known.

**Objectives:** To investigate the cytokine and chemokine production, and whole blood RNA expression after SARS-CoV-2 vaccination in children with previous MIS-C.

**Methods:** Children older than 12 years, who were diagnosed with MIS-C between 2020 and 2021 and healthy children were offered 2 doses of Pfizer COMIRNATY vaccine, 3 weeks apart, as per international guidelines. Serum and whole blood RNA were collected prior to vaccination, 1 week after each vaccine and 6 weeks after the first vaccine dose. At every time point, Luminex for cytokine and chemokine production, and qPCR for immune gene expression were performed.

**Results:** Three out of the eleven eligible children with previous MIS-C agreed to receive vaccination (1 girl, 2 boys, all aged 12). Four healthy children volunteered for vaccination (3 boys, 1 girl, all aged 12). Throughout the vaccination schedule, there was no difference in serum IL-1β, IL-1RA, IL-6, IL-27, IP-10, MCP-1 or TNF-ɑ between children with prior MIS-C or healthy children. However, at all sampling points children with prior MIS-C had a consistently raised serum IL-10 at baseline (p=0.011), 1 week after the first dose (p=0.002) and at 6 weeks follow-up (p=0.014). Immune gene expression analysis revealed no significant differences between the two groups at any timepoint, although several transcripts were nominally differentially expressed (p = 0.057). *IL27* was upregulated in children with previous MIS-C after the first dose, while CXCR3 was downregulated at this timepoint and *TIMP2*, *TRMT2A*, *RAB33A* and *GZMB* were downregulated after the second dose. At 6 weeks after the first dose, *FCGR2A*, *IL1B*, *IFNAR1* and *GZMB* were all downregulated in children with prior MIS-C.

**Conclusion:** In this small group, the immune response to SARS-CoV-2 mRNA vaccination in children with previous MIS-C appears to be slightly altered compared to healthy controls. However, these differences do not appear to be clinically relevant, and vaccination of these children should continue.

**Date of birth:** mai 07, YY

**Patient Consent**

Not applicable (there are no patient data)

**Disclosure of Interest**

None Declared

### COVID-19 (Coronavirus)

#### P116 Coronavirus infection and arthritis in children

##### Akhenbekova Aida^1^, Gulmira Erzhanova^2^, Indira Dzhaksybaeva^1^, Gulsara Rakhym^3^

###### ^1^Department of Children’s Diseases, Kazakh National Medical University named after S.D.Asfendiyarov; ^2^Department of Pediatrics, Scientific Center of Pediatrics and Pediatric Surgery; ^3^Department of Rheumatology, Kazakh National Medical University named after S.D.Asfendiyarov, Almaty, Kazakhstan

####### **Correspondence:** Akhenbekova Aida

*Pediatric Rheumatology 2024*, **22(2):**PReS24-ABS-1693

**Introduction:** It is known that coronavirus infection has influenced the course of many diseases, including rheumatic ones, which was probably due to the production of autoantibodies in patients with COVID-19, with the most common associations described with antiphospholipid antibodies, ANA, RF and ACCP. In children, coronavirus infection manifested itself in the form of multisystem inflammatory syndrome and macrophage activation syndrome, and the previous coronavirus infection influenced the onset and course of rheumatological pathology.

**Objectives:** To study clinical and laboratory features of the course of arthritis in children who have suffered coronavirus infection.

**Methods:** A retrospective analysis of medical records and prospective observation of children with reactive arthritis (ReA) and juvenile idiopathic arthritis (JIA) of patients at the Scientific Center of Pediatrics and Pediatric Surgery and the Children’s Emergency Medical Care Сenter were carried out in 2022-2023. Clinical examination of arthritis patients included assessment of disease activity based on laboratory and instrumental data and organ damage. Statistical analysis was carried out using the StatTech v. 4.1.2 (developer - “Stattekh” LLC, Russia). Differences were considered statistically significant at p < 0.05.

**Results:** There were 70 children under observation, who were divided into groups depending on the presence of antibodies to Covid 19. The first study group with ReA consisted of 28 children with AT for COVID-19 and 42 seronegative children. In this group with ReA, a comparative analysis of laboratory parameters of children with positive antibodies to COVID-19 with a control group without antibodies showed significantly higher levels of leukocytes (p<0.04), platelets (p<0.01), ESR (p<0.05), inflammatory markers CRP (p<0.001), ferritin (p<0.001), and LDH (p<0.02).

Patients with JIA comprised the second study group. A positive SARS Covid-19 antibody titer was more often detected in children with the systemic variant: 86% versus 65% in the articular form of JIA. With positive antibodies for COVID-19, significantly higher levels of peripheral blood leukocytes, ESR, CRP, D-dimer, decreased hemoglobin, as well as hyperferritinemia on average 294 ng/ml (which is significantly higher in the absence of antibodies for COVID-19). The JIA clinic with AT for COVID-19 was characterized by fever, lymphadenopathy, carditis, articular syndrome with frequent damage to the hip joint in the form of synovitis, early rapidly progressive uveitis, and in one case - development of macrophage activation syndrome, which we know as a severe complication of coronavirus infection.

Using indirect immunofluorescence for HEp-2, various ANAs were identified. DFS-70, often found in systemic connective tissue diseases, was a common finding. Children with JIA who had a latent or mild form of coronavirus infection had higher rates of inflammation, immunological activity and required early initiation of biological therapy.

**Conclusion:** Coronavirus infection, transmitted in a mild or latent form, has a risk of developing autoimmune diseases in children with the production of autoantibodies to connective tissue and vascular endothelium and is characterized by higher rates of inflammatory process activity and increased ferritin levels in contrast to the control group. In the treatment of reactive arthritis associated with COVID-19, it is recommended to carry out extended diagnostics, longer observation, and in the case of JIA, early initiation of biological therapy.

**Patient Consent**

Yes, I received consent

**Disclosure of Interest**

None Declared

### COVID-19 (Coronavirus)

#### P117 Cellular immune response to the anti-SARS-CoV-2 BNT162B2 MRNA vaccine in pediatric autoimmune inflammatory rheumatic disease patients and controls

##### Tali Eviatar^1,2^, Adi Pappo^3^, Tal Freund^2,4^, Yishai Friedlander^5^, Ori Elkayam^1,2^, David Hagin^2,4^, Merav Heshin-Bekenstein^*2,6^, m m^4^

###### ^1^Rheumatology, Tel Aviv Medical Center; ^2^School of Medicine, Tel Aviv University, Tel Aviv; ^3^Pediatric Rheumatology, Schneider Children’s Hospital, Petach Tikva; ^4^Allergy and clinical Immunology, Tel Aviv Medical Center, Tel Aviv; ^5^StatistiX, Beer Tuvya; ^6^Pediatric Rheumatology, Dana Children’s Hospital of Tel Aviv Medical Center, Tel Aviv, Israel

####### **Correspondence:** Merav Heshin-Bekenstein

*Pediatric Rheumatology 2024*, **22(2):**PReS24-ABS-1025

**Introduction:** Children with autoimmune inflammatory rheumatic diseases (AIIRD) are recommended to receive anti-SARS-CoV-2 vaccines. The data regarding the cellular immune response to BNT162b2 mRNA vaccine in pediatric AIIRD patients is scarce.

**Objectives:** To compare the cellular response to BNT162b2 vaccine of pediatric AIIRD patients and healthy control.

**Methods:** A prospective longitudinal study was conducted between April 2021 to December 2022 at the Tel Aviv Medical Center, Israel. Children under the age of 18, with pediatric-onset AIIRD and healthy controls, who have received at least two doses of the BNT162b2 mRNA vaccine, were included. Blood samples were collected 2-8 weeks after the second vaccine dose. Humoral response was evaluated by serum levels of anti-SARS-CoV-2 spike receptor-binding domain (RBD) antibodies, (SARS-CoV-2 IgG II Quant assay, ARCHITECT, Abbott). Cellular response was evaluated by flow cytometry, measuring IFNg and TNFa production by CD4+ T-cells following stimulation with SARS-CoV-2 Spike peptide mix.

**Results:** The study included 20 pediatric AIIRD patients and 11 controls with similar age and sex distribution. The mean age of all participants was 12.6±2.94 years, and 58.1% (n=18) were females. The AIIRD patients’ diagnoses included 8 (40%) with juvenile idiopathic arthritis, 3 (15%) each of lupus, scleroderma, and uveitis, and 1 (5% each) with either vasculitis, myositis or inflammatory bowel disease with arthritis.

The cellular response to the BNT162b2 vaccine was statistically similar in both groups, but numerically higher in the AIIRD children compared with controls, as measured by calculated absolute number of CD4s staining positive for TNFα (median (range) 660 (0-2140) vs. 600 (0-1890), p=0.36), IFNg production (380 (0-1700) vs. 280 (0-840), p=0.27), or both TNFα and IFNgg (465 (0-2900) vs. 390 (0-920) p=0.22), respectively. However, the humoral response, as measured by anti-RBD antibody levels, was statistically higher in the control group compared with AIIRD children (median (range) 34,837.9 (3716.7-40,000) vs. 6789.6 (243-23,274.7), respectively, p<0.001).

During the study period, 7 out of 16 (43.75%) AIIRD children and 8/11 (72.7%) controls had a breakthrough COVID-19 infection (p=0.48).

**Conclusion:** Compared with healthy controls, pediatric patients with AIIRD demonstrated similar cellular responses. Patients showed reduced humoral response compared with healthy adolescents, but similar breakthrough infection rates. These findings may support the importance of the cellular response in protecting against COVID-19 infections.

**Trial registration identifying number:** Not Applicable.

**Date of birth:** janvier 30

**Patient Consent**

Not applicable (there are no patient data)

**Disclosure of Interest**

None Declared

**References**

1. Kearsley-Fleet L, Chang ML, Lawson-Tovey S, *et al.* Outcomes of SARS-CoV-2 infection among children and young people with pre-existing rheumatic and musculoskeletal diseases. *Ann Rheum Dis*. 2022;81:998–1005.
2. Walters HM, Mian Z, Thomas L, *et al.* Seroprevalence and clinical outcomes of SARS-CoV-2 in paediatric patients with rheumatic disease. *Rheumatology (United Kingdom)*. 2022;61:SI112–9.

### COVID-19 (Coronavirus)

#### P118 A Singapore experience with Multisystem Inflammatory Syndrome in Children (MIS-C) post-COVID-19 infection

##### Pauline P. L. Chan Ng^1,2^, Jacqueline X. L. Yap^2^, Alicia Y. H. Kang^2^, Lee Kean Lim^1^, Elizabeth Y. Ang^1,2^

###### ^1^National University Health System, Khoo Teck Puat-National University Children’s Medical Institute; ^2^Department of Paediatrics, Yong Loo Lin School of Medicine, National University of Singapore, Singapore, Singapore

####### **Correspondence:** Pauline P. L. Chan Ng

*Pediatric Rheumatology 2024*, **22(2):**PReS24-ABS-1451

**Introduction:** Singapore saw its first case of MIS-C in Oct 21.

**Objectives:** We aim to describe a series of patients diagnosed with MIS-C in a local tertiary referral hospital and identify best markers of treatment response. We also aim to describe the long term outcome of these patients.

**Methods:** Clinical charts of paediatric patients diagnosed with MIS-C (by CDC case definition) from Oct 21-Oct 22 were retrospectively reviewed. They were followed up for at least 6 months post-diagnosis.

**Results:** A total of 14 patients were diagnosed from Oct 21-22. No further cases of MIS-C have been diagnosed in our institution after Oct 22. Diagnosis was made at a median of 5 days (range (R):4-8 days) from onset of symptoms. Mean age at diagnosis was 4.5 years (R:21 months-8 years) with an equal gender distribution. All but one had symptomatic COVID-19 infection at mean of 32 days (R:17-52 days) prior to MIS-C onset.

All patients had fever and fulfilled at least 1 criteria of Kawasaki Disease; majority (79%) were incomplete. Non-suppurative conjunctivitis (86%), oro-mucosal changes (64%) and rash (57%) were most common. Cardiovascular (93%), gastrointestinal (79%), haematological (79%) and neurological (64%) systems were most commonly involved.

At presentation, median C-reactive protein (CRP), procalcitonin (PCT), ferritin and erythrocyte sedimentation rate were 121.6 mg/L, 3.5 ng/mL, 409 μg/L and 33 mm/h respectively. Ten (71%) had thrombocytopenia and 7 (50%) had lymphopenia. Raised troponin I, CKMB and NT-proBNP at presentation were seen in 50%, 14% and 93% respectively.

Three (21%) needed inotropes. None had invasive ventilation. All received intravenous immunoglobulin (IVIg) 2g/kg and systemic steroids (only one received oral steroids only while the rest received intravenous followed by tapering dose of oral steroids). Eight (57%) received high dose steroids (defined as IV methylprednisolone 10 mg/kg/day or more). None required biologics.

Following treatment, fever lysed after a mean of 2 days (R:1-5 days). CRP and PCT were the fastest blood markers to improve: halving 3 days after treatment (R_CRP_:2-5 days; R_PCT_:1-5 days). Ferritin halved over 8 days(R:4-47 days) and NT-proBNP over 3 days (R:1-4 days). Lymphopaenia resolved after 3 days (R:1-6 days) and thrombocytopaenia after 4 days (R:1-9 days).

Seven (50%) patients had abnormal echocardiogram at diagnosis. This reduced to 3 (21%), 1 (7%) and none by week 2, week 4-6 and 6 month respectively. All 3 patients who required inotropes had normal echocardiograms by 4-6 weeks. None had recurrence of MIS-C till present (May 24). Five (36%) went on to receive at least one dose of COVID-19 vaccine with no adverse reaction.

**Conclusion:** Though younger than previously described cohorts, our patients showed excellent outcomes with IVIg and early systemic steroids. All had normal echocardiogram by 6 months post diagnosis. CRP, PCT and NT-proBNP were the fastest blood markers of improvement. Rates of MIS-C have decreased with time and none had recurred when followed up till date. Vaccination against COVID-19 appears to be safe in patients with MIS-C.

**Patient Consent**

Yes, I received consent

**Disclosure of Interest**

None Declared

### COVID-19 (Coronavirus)

#### P119 Review on admission for child maltreatment in a regional hospital before and during COVID-19 pandemic

##### Cheuk Yin Leung, Pui King Chiang, Kwing Wan Tsui

###### Paediatrics and Adolescent Medicine, Alice Ho Miu Ling Nethersole Hospital, Hong Kong, China

####### **Correspondence:** Cheuk Yin Leung

*Pediatric Rheumatology 2024*, **22(2):**PReS24-ABS-1120

**Introduction:** As an unprecedented global health crisis, the COVID-19 pandemic has adversely impacted the physical and mental well-being of children and young people. Limited studies were conducted on the impact of COVID-19 pandemic on child maltreatment.

**Objectives:** This study is designed to review on the epidemiology, clinical characteristics, risk factors and precipitating factors of the child maltreatment hospitalization before and during COVID-19 pandemic.

**Methods:** We conducted a retrospective chart review of children aged below 18 years who were admitted for suspected child abuse in the Paediatric unit of Alice Ho Miu Ling Nethersole Hospital (AHNH), Hong Kong. Data were collected from January 2017 to December 2020 using Clinical Data Analysis and Reporting System (CDARS) and Child Abuse Surveillance Form.

**Results:** 408 hospitalizations of suspected child maltreatment were identified. A total of 376 cases were included after reviewing the medical records. 177 out of 376 (47.1%) cases were concluded as “established” child abuse while 293 (77.9%) were “established or high-risk” child abuse in the multi-disciplinary case conference (MDCC) or welfare meeting. Among the “established or high-risk” cases, 72% were physical abuse, followed by neglect (17.7%), sexual abuse (15.4%), and multi-type abuse (6.1%). “Established or high-risk” sexual abuse cases had increased (OR 2.00, p=0.041) during the pandemic.

Children aged 12 years old or older had a higher risk of being abused (p=0.024). Victims with learning disability (p=0.017) or mood-related disorders (p=0.001) were associated with higher risk of child abuse. Significantly more child abuse cases were self-reported (p=0.005). The mean of length-of-stay in hospital was 11.0 ±SD 11.4 days which was of no difference before and during the pandemic. The proportion of victims with complications increased during the pandemic (p=0.006) and they were likely to suffer from physical or psychological disabilities (p<0.001).

**Conclusion:** During the pandemic, there was a shift of age group of child abuse victims to older children with more cases being self-reported. The true magnitude of the problem was possibly underestimated owing to the school suspension and disruption of child protection safeguard services.

**Patient Consent**

No, I have not received consent

**Disclosure of Interest**

None Declared

### COVID-19 (Coronavirus)

#### P120 Subcutaneous anakira in the management of severe and refractory MIS-C in a single center in greece

##### Maria Tsinti^1^, Vassiliki Dermentzoglou^1^, Elena Tsitsami^2^

###### ^1^Rheumatology Unit, Children’s Hospital, Agia Sofia; ^2^Medical School, University of Athens, Athens, Greece

####### **Correspondence:** Maria Tsinti

*Pediatric Rheumatology 2024*, **22(2):**PReS24-ABS-1489

**Introduction:** Multisystem inflammatory syndrime in children (MIS-C) is a cytokine storm syndrome that may lead to significant cardiovascular and respiratory dysfunction. Prompt initiation of treatment can stop the hyperinflammatory response. Anakinra can be effective in children unresponsive to first line therapies, i.e. IVIG and steroids.

**Objectives:** To describe the characteristics of MIS-C patients treated in our center, necessitating subcutaneous anakinra for inflammation resolution.

**Methods:** Demographic and clinical characteristics of patients diagnosed with MIS-C between Jan 2022 to Apr 2024 are analyzed.

**Results:** Twelve children (boys=7) received anakinra, dose 2-6 mg/kg, max 300mg/ day. All had received IVIG and pulse methylprednisolone (IVMP) 30 mg/kg/24h, 3-6 doses. Eight were admitted to ICU. In 5/8 anakinra was initiated concomitantly with IVIG and IVMP due to rapid deterioration of cardiovascular function, necessitating support with inotropes; in 3/8 due to non response to IVIG and IVMP. Three infants (all boys, aged 5-7 months) received anakinra for 6 months due to coronary involvement, 2 with Coronary Artery Aneurysms with improvement in a 1-month period, one with medium CA dilation with complete recovery. All other children were aged 5 to 14 years; 1 received anakinra for MAS resistant to 6 IVMP pulses, 8 for cardiovascular collapse. Median time to response in maximum anakinra dose was 2 days. All children had gastrointestinal involvement and four gallbladder edema; 2 developed pancreatitis that resolved after one month of treatment. In 7/9 older children duration of treatment with anakinra was 2 months and of steroids 1 month. A 13-year-old girl and an 11-year-old girl received anakinra for 6 months due to recalcitrant pericarditis and fever with hyperinflammation recurrence after URI, respectively. Except for the 2 infants with CAA all children recovered completely without sequelae. Cardiac MRI was performed 6 months after initial hospitalization and revealed normal cardiac function.

**Conclusion:** Subcutaneous anakinra was effective for hyperinflammation resolution in MIS-C patients. It was initiated in ICU- admitted patients concomitantly with IVIG and IVMP due to rapid cardiovascular deterioration or in the ward in cases where inflammation did not resolve under IVIG and IVMP treatment. Prompt initiation of anakinra arrested the hyperinflammatory response and the majority of patients recovered without sequelae.

**Patient Consent**

Yes, I received consent

**Disclosure of Interest**

None Declared

**References**

1. Front. Pediatr. 11: 1137051
2. Front. Pediatr. 12: 1270878
3. Pediatr Rheumatol (2023) 21: 141

### COVID-19 (Coronavirus)

#### P121 Subcutaneous fat tissue necrosis of newborn - case report

##### Eka Nakhutsrishvili, Tinatin Kutubidze

###### Tbilisi State Medical University, Tbilisi, Georgia

####### **Correspondence:** Eka Nakhutsrishvili

*Pediatric Rheumatology 2024*, **22(2):**PReS24-ABS-1034

**Introduction:** Covid - 19 has become a challenge for doctors around the world, including pediatricians since December 2019. Most children with COVID-19 have mild symptoms or have no symptoms at all. However, some children can get severely ill from COVID-19. Subcutaneous fat necrosis of newborn is a rare cutaneous disorder. It usually presents as subcutaneous nodules or plaques, within the first few weeks of life, following an eventful delivery.

**Objectives:** To determine the connection between the transmitted Covid-19 and the manifestation of autoimmune, rheumatic diseases.

**Methods:** Here we present a case of a newborn who presented with severe panniculitis and pancytopenia after acquiring a SARS-CoV-2 infection.

**Results:** Patient 2 months old, male, G1P1, in the 3rd trimester of pregnancy, the mother had a viral infection, and antibacterial therapy was also used, delivery by caesarean section, gestation 37 weeks, mass -3400 g, length - 51 cm, newborn was treated for hyperbilirubinemia. At the age of 17 days, the newborn was diagnosed with acute bronchiolitis, at the age of 1 month, he was confirmed with Covid-19, from which in 5 days multiple hemorrhagic and erythematous nodules appeared on the face, chest, back, and limbs. During hospitalization, there was general malaise, diffuse nodular hemorrhagic rash, liver 2 cm below the level of the rib, lungs - vesicular sound, cardiovascular system - soft systolic murmur, leukopenia in peripheral blood, with severe neutropenia. He was consulted by an infectious disease specialist, a hemato-oncologist, a neurologist, a small amount of fluid in the pericardial cavity, a moderate dilatation of the left coronary artery was revealed by echocardiography. The existing changes were assessed as a post-viral (post-covid) condition. IV antibacterial and glucocorticosteroid therapy was started. In dynamics, wbc, neutrophils increased, the nodular erythematous rash disappeared, the patient was active, actively sucking the breast, he was discharged. 4 days after discharge from the clinic, fever up to 39 degrees Celsius, malaise, adynamia were observed, hospitalization was repeated. CBC revealed anemia, thrombocytopenia, leukopenia. A blood transfusion was performed. Broad-spectrum antibiotic therapy was started, neuroinfection was ruled out. In dynamics, the body temperature was normalized, although anemia, thrombocytopenia and severe leukopenia remained in the CBC. A bone marrow puncture was performed, the morphological picture of the puncture was consistent with bone marrow aplasia.

**Conclusion:** It can be concluded that even with the asymptomatic course of SARS-CoV-2 Infection in children, complications can be observed, and the syndrome of the so-called late Covid, which dictates the need for a thorough examination of these patients.There is a need for more high-quality pediatric SARS-CoV-2 research and observation in dynamics.

**Date of birth:** août 15, Y

**Patient Consent**

Yes, I received consent

**Disclosure of Interest**

None Declared

### COVID-19 (Coronavirus)

#### P122 Neutropenia and JIA - autoimmunity after COVID-19?

##### Eka Nakhutsrishvili, Tinatin Kutubidze

###### Tbilisi State Medical University, Tbilisi, Georgia

####### **Correspondence:** Eka Nakhutsrishvili

*Pediatric Rheumatology 2024*, **22(2):**PReS24-ABS-1335

**Introduction:** Neutrophils are the most numerous innate immune effectors in the circulation and play a key role in both host defense and auto-inflammatory processes. In the classic presentation of systemic juvenile idiopathic arthritis (sJIA), white blood cell counts are almost always elevated and range from 20,000 to 30,000/mm3, positive antinuclear antibodies and rheumatoid factor are rare in sJIA, although recent research has shown , that patients with systemic JIA have increased ANA and rheumatoid factor titers over time.

**Objectives:** To discuss whether maternal SARS-CoV-2 infection may lead to innate immune dysregulation and the resulting unusual presentation of JIA and neutropenia.

**Methods:** We present a clinical case of a 13-month-old patient with severe neutropenia, anemia, and arthritis.

**Results:** The girl was born P2G2, full-term, with a body weight of 3300 g. Maternal SARS-CoV-2 infection was confirmed at 12 weeks of gestation, requiring short-term anticoagulation therapy. At the age of 6 months after vaccination, the patient developed a high fever, which lasted for 1 month, later, moderate anemia and agranulocytosis were observed. A number of studies were performed, including a bone marrow aspirate, which did not show any abnormalities. Considering neutropenia, treatment included antibiotic therapy for the next 4 months. In 3 months, the patient again had an episode of fever accompanied with weakness, swelling, pain, limitation of movement in the PIP joints and both knees, both ankles, right hip, elbow, wrist.. A mass deficit <- 2SD was observed. The conducted laboratory studies ruled out immunodeficiency. Peripheral blood analysis again showed severe anemia and agranulocytosis, antinuclear factor 1:2560 with homogeneous type fluorescence in high titer. The condition was assessed as sJIA and high-dose glucocorticosteroid therapy was prescribed i.v. The intensity of arthritis decreased in dynamics, markers of inflammation - improved, fever was no longer observed. The patient is currently receiving 7.5 mg prednisolone per day, ANF 1:640, no exacerbation of the disease is noted.

**Conclusion:** New-onset rheumatic diseases are a new challenge for rheumatologists and data are scarce. Although clearly implicated in JIA, the specific contribution of neutrophils to pathogenesis and the use of neutrophil activity as biomarkers require further study. Elucidation of these important issues will impact the diagnosis and treatment of pediatric rheumatologic conditions.

**Date of birth:** août 15, Y

**Patient Consent**

Yes, I received consent

**Disclosure of Interest**

None Declared

**References**

1. Nauseef WM, Borregaard N. Neutrophils at work. Nat Immunol. (2014) 15:602–11. 10.1038/ni.292.
2. Hügle B, Hinze C, Lainka E, Fischer N, Haas J-P. Development of positive antinuclear antibodies and rheumatoid factor in systemic juvenile idiopathic arthritis points toward an autoimmune phenotype later in the disease course. Pediatr Rheumatol Online J. (2014) 12:28. 10.1186/1546-0096-12-28.
3. Abraham Edgar Gracia-Ramos Cells 2021, 10, 3592- New Onset of Autoimmune Diseases Following COVID-19 Diagnosis

### COVID-19 (Coronavirus)

#### P123 Leukocytoclastic vasculitis after COVID-19 infection in a 5-year-old girl - case report

##### Eka Nakhutsrishvili, Tinatin Kutubidze

###### Tbilisi State Medical University, Tbilisi, Georgia

####### **Correspondence:** Eka Nakhutsrishvili

*Pediatric Rheumatology 2024*, **22(2):**PReS24-ABS-1333

**Introduction:** Viral infections are known triggers of leukocytoclastic vasculitis. There has been an increase in the number of vasculitis detected after the transfer of the COVID-19 infection. There have been many reports in the literature about the different manifestations of COVID-19 in children. Various authors note that there is a spectrum of dermatological and vascular manifestations that can be expressed from a mild skin rash to a severe stroke. Our clinical case presents a presentation of leukocytoclastic vasculitis after asymptomatic COVID-19 infection.

**Objectives:** To explore the relationship between vasculitis and COVID-19.

**Methods:** We are presenting the case of purpuric rush in 5-year-old girl, who had asymptomatic Covid-19 infection prior to clinical manifestation of vasculitis.

**Results:** A previously healthy 5-year-old girl presented to the emergency department with a 1-week history of a rash. The rash started on the shins and feet, which gradually spread to the back. Elements were petechiae and purpuric type, palpable, without itching. There was no abdominal pain, fever, chills, joint pain or swelling. Medical history was significant for asymptomatic COVID-19 infection approximately 4 weeks before the onset of rash, which was detected as part of contact screening. During physical examination: parameters of growth and development are normal, vital signs - within normal range. Rashes on the skin were observed on the soles, feet and shins - round dark palpable purpuric elements and petechiae. Complete blood count, liver function tests, and kidney function were within normal limits. Assessment of inflammatory markers showed normal C-reactive protein, procalcitonin and ferritin, but slightly elevated erythrocyte sedimentation rate, C3-normal, ANA negative, D-dimer elevated. ECG - normal. Urinalysis showed moderate erythrocyturia without proteinuria. The patient was prescribed a course of oral prednisolone, she spent 2 days in the hospital. No skin biopsy was performed.

Our patient was completely asymptomatic and did not have any clinical manifestations of COVID-19. Therefore, we hypothesize that the patient developed a hypersensitivity reaction and so-called leukocytoclastic vasculitis after contracting COVID-19.

**Conclusion:** More research should be conducted on the immunological aspects of COVID-19 infection and blood vessel involvement in order to formulate a better understanding of the correlation between COVID-19 infection, post-COVID syndrome, and secondary vasculitis due to viral inflammation.

**Date of birth:** août 15, Y

**Patient Consent**

Yes, I received consent

**Disclosure of Interest**

None Declared

**References**

1. Genovese G, Moltrasio C, Berti E, et al. Skin manifestations associated with COVID-19: current knowledge and future perspectives. Dermatology 2021;237:1–12
2. Becker RC. COVID-19-associated vasculitis and vasculopathy. J Thromb Thrombolysis 2020;50:499–511
3. Gurinder Kumar ,1 Shanta Pillai,1 Paige Norwick,2 Hulya Bukulmez1 - Leucocytoclastic vasculitis secondary to COVID-19 infection in a young child, BMJ Case Rep 2021;14:e242192. 10.1136/bcr2021-242192.

### Disease outcome and transition

#### P124 From paediatric to adult care: validating JADAS for adults with juvenile idiopathic arthritis

##### Filipa Oliveira-Ramos^1^, Lianne Kearsley-Fleet^2^, Ana Rebollo^3^, Duarte Vinha^1^, Jenny Humphreys^2^, Veronika Rypdal^4^, Juan José Bethencourt-Baute^5^, Jordi Antón^6^, Maria José Santos^7^, Elsa Mateus^8^, Claudia Gomes^8^, Alessandro Consolaro^9^, Stephanie Shoop-Worrall^2^

###### ^1^Hospital Universitário Santa Maria, Lisbon University, Lisbon, Portugal; ^2^University of Manchester, Manchester, United Kingdom; ^3^Beata María Ana Hospital, Ruber University Hospital, Madrid, Spain; ^4^University Hospital of North Norway, Arctic University of Norway, Tromsø, Norway; ^5^Hospital Universitario de Canarias, Tenerife; ^6^Hospital Sant Joan de Déu, Universitat de Barcelona, Barcelona, Spain; ^7^Hospital Garcia de Orta, Almada; ^8^LPCDR, National Association of Patients with Juvenile Arthritis, Lisbon, Portugal; ^9^IRCCS Istituto Giannina Gaslini, Università di Genova, Genoa, Italy

####### **Correspondence:** Filipa Oliveira-Ramos

*Pediatric Rheumatology 2024*, **22(2):**PReS24-ABS-1474

**Introduction:** Many children with Juvenile Idiopathic Arthritis (JIA) continue to experience active disease into adulthood, yet no validated disease activity measure exists for adult use. While the Juvenile Arthritis Disease Activity Score (JADAS) is established for children, its relevance and validation for adults remain unexplored.

**Objectives:** Validate JADAS for JIA patients older than 18 years, focusing on criterion and construct validity.

**Methods:** This analysis used data from EPOCA (cross-sectional cohort) and two prospective cohorts: Rheumatic Diseases Portuguese Registry (Reuma.pt), and Spanish national registry for adults with JIA (JUVENSER). Included JIA patients were >18 years old, met ILAR criteria, and had data available for JADAS and other disease activity scores (DAS28, SDAI, CDAI). Demographic details, disease characteristics, and clinical assessments were collected. Patient’s most recent visits, ranging from January-2001 to February-2024 (99% from 2010 onwards) were used. Construct validity was evaluated through Spearman’s rank correlation between JADAS (10/27/71/cJADAS10), joint counts, physician, and patient global assessment with the adult constructs (DAS28/SDAI/CDAI). Criterion validity was assessed using Cohen’s weighted kappa to assess accuracy of JADAS versus adult constructs, and sensitivity and specificity of detecting clinically inactive disease (CID).

**Results:** Analysis included 1315 adult JIA patients, from Reuma.pt (N=545), JUVENSER (N=392), and EPOCA (N=378) across 39 nationalities. Median age at disease onset was 9 years for Reuma.pt (IQR 4-13) and JUVENSER (IQR 3.7-13) patients, and 11 years (IQR 6.7-14) for EPOCA patients. The median age at the last study visit and disease duration varied: Reuma.pt patients were older (25 versus 19-21 years), with longer disease duration (18 versus 8-13 years). EPOCA patients had higher JADAS in the registered visits versus the other cohorts; median JADAS27 3.5 versus 2 (Reuma.pt) and 0 (JUVENSER).

For construct validity, the Spearman’s correlations between JADAS10 and cJADAS10 with SDAI (JADAS10:0.95; cJADAS10: 0.96) and CDAI (JADAS10: 0.96; cJADAS10:0.99) were high. Whilst correlation between JADAS10 and cJADAS10 with DAS28 was lower: 0.69 and 0.64 respectively. Similar correlations were seen for JADAS27 and JADAS71, and also when limited to only those patients with RF-negative polyarthritis, or persistent or extended oligoarthritis. All JADAS versions showed higher correlation with active joint count and patient and physician global assessment than DAS28.

For criterion validity, the Cohen’s weighted kappa for JADAS10 with the adult indices were 0.51 (95% CI 0.48-0.54) for DAS28, 0.72 (0.70-0.74) for SDAI, and 0.72 (0.70-0.74) for CDAI. Results were similar when limited to patients with RF-negative polyarthritis, or persistent or extended oligoarthritis. However, performance was poorer across the other JADAS versions. For those with CID, sensitivity ranged from 0.64-0.87, and specificity ranged from 0.96-0.97.

**Conclusion:** Preliminary findings suggest that JADAS is a valid tool for measuring disease activity in adults with JIA. Its strong performance in criterion and construct validity supports its potential for broader use in clinical practice, helping to bridge the gap in transition care from paediatric to adult rheumatology. Further studies are needed to explore other aspects of validity.

**Patient Consent**

Not applicable (there are no patient data)

**Disclosure of Interest**

None Declare

### Disease outcome and transition

#### P125 The transition from pediatric to adult healthcare. Caregiver parents point of view in Italy

##### Matteo Santopietro^1,2^, Antonella Celano^1^

###### ^1^APMARR National Association of People with Rheumatic and Rare Disease - APS-ETS, LECCE; ^2^WeResearch Marketing Research, Milano, Italy

####### **Correspondence:** Matteo Santopietro

*Pediatric Rheumatology 2024*, **22(2):**PReS24-ABS-1491

**Introduction:** Rheumatological diseases affect over 5 and a half million people in Italy and are diversified into more than 180 pathologies. On average 10,000 adolescents in Italy are affected by rheumatological disease, the most common being juvenile idiopathic arthritis. The transition from pediatric to adult healthcare is a crucial moment in an adolescent’s developmental direction. The research was carried out in Italy to investigate the barriers in the transition process, from the point of view parent caregivers.

**Objectives:** Gather data from Italian parents caregiver of patients (14-20 y.o.) affected from a rheumatological disease to understand the transition process in terms of: 1) touchpoints of information; 2) level of information.

**Methods:** A quantitative survey was carried out through a questionnaire administered throughout the Italian national territory to a sample of N=197 caregiver parents.

The total sample was divided into two targets.

- Target 1: caregiver parents of rheumatological patients (14-20 y.o.) who made the transition (42,6%,N=84).
- Target 2: caregiver parents of rheumatological patients (14-20 y.o.) who didn’t make the transition (57,4%,N=113).

The questionnaire was made up of 26 questions, of which 23 were closed and 3 were open. For the administration of the questionnaires, the CAWI (Computer Aided Web Interview) methodology of on-line survey was used. The 197 interviews were carried out from September 1st to 30th 2023.

**Results**

1. Touchpoints of information (multiple answers possible).
  Total sample (N=197): pediatric rheumatologist 55,8%, N=110; general practicioner 41,6%, N=82; adult rheumatologist 31,5%, N=62.
  Target 1: caregiver parents of rheumatological patients (14-20 y.o.) who made the transition (N=84): pediatric rheumatologist 48,8%, N=41; general practitioner 48,8%, N=41; adult rheumatologist 45,2%, N=38.
  Target 2: caregiver parents of rheumatological patients (14-20 y.o.) who didn’t make the transition (N=113): pediatric rheumatologist 61,1%, N=69; general practitioner 36,3%, N=41; adult rheumatologist 21,2%, N=24).
2. Level of information (5 points Likert scale. 1: not complete at all, to 5: complete at all).
  Total sample: (N=197): 3 out of 10 parents caregiver (29,4%, N=58) declared that it has no complete information on the transition process.
  Target 1: (N=84) caregiver parents of rheumatological patients (14-20 y.o.) who made the transition: 20,2%,N=17, declared that it has no complete information on the transition process.
  Target 2: (N=113) caregiver parents of rheumatological patients (14-20 y.o.) who didn’t make the transition: 36,3%, N=41, declared that it has no complete information on the transition process.

**Conclusion:** The main barrier that parents caregivers and patients face in the transition from pediatric to adult healthcare is the lack of clear information about this process. Also, the transition involves psychological and social challenges, such as adapting to new medical environments and increasing responsibility for your own health. The research highlights insufficient information as the main critical area that makes the transition a path that is not always easy in Italy. Future research will have to highlight the correlation between the level of information and a correct transition from pediatric to adult healthcare.

**Patient Consent**

Not applicable (there are no patient data)

**Disclosure of Interest**

None Declared

### Disease outcome and transition

#### P126 Transitional care for rheumatic conditions across the globe in 2024: results from a survey investigating current clinical practice, available resources, barriers and opportunities

##### Elena Moraitis^1,2^, Selcan Demir^3^, Micol Romano^4^, Coziana Ciurtin^5^, Joost Swart^6,7^, Ivan Foeldvari^8^, Polly Livermore^1,2^, Dalila Tremarias^9^, Erkan Demirkaya^4^, Sylvia Kamphuis^10,11^ and on behalf of the PReS Working Parties

###### ^1^Paediatric Rheumatology, Great Ormond Street Hospital for Children; ^2^UCL Institute of Child Health, London, United Kingdom; ^3^Paediatric Rheumatology, Eskisehir Osmangazi University, Eskisehir, Türkiye; ^4^Paediatric Rheumatology, Schulich School of Medicine & Dentistry, University of Western Ontario, London, Ontario, Canada; ^5^Centre for Adolescent Rheumatology, University College London, London, United Kingdom; ^6^Paediatric Rheumatology, Immunology and Infectious Diseases, Wilhelmina Children’s Hospital; ^7^University Medical Center, Utrecht University, Utrecht , Netherlands; ^8^Paediatric Rheumatology, Hamburger Zentrum für Kinder- und Jugendrheumatologie, Hamburg , Germany; ^9^Lupus Europe, Brussels, Belgium; ^10^Paediatric Rheumatology, Sophia Children’s Hospital; ^11^Paediatric Rheumatology, Erasmus University Medical Center, Rotterdam, Netherlands

####### **Correspondence:** Elena Moraitis

*Pediatric Rheumatology 2024*, **22(2):**PReS24-ABS-1717

**Introduction:** EULAR/PReS published guideline and recommendations for transitional care in 2017. Despite the significant efforts to improve transitional care in recent years, we hypothesized that paediatric and adult rheumatology care providers still face significant barriers to successful transitional care and that the 2017 guidelines need to be updated and refined, with special focus on addressing transitional care at a global level and adding disease-specific modules.

**Objectives:** To re-assess the current pediatric rheumatology providers’ transition practices, resources, policy awareness, barriers, perspectives and needs post publication of PReS/EULAR guidelines.

**Methods:** Paediatric rheumatology providers worldwide were invited to complete a 21-item anonymised online survey which was distributed via PReS, Bulletin Board and Asia email lists.

**Results:** The survey was completed by 153 participants from 42 countries on 6 continents, with Europe representing 54% of respondents: 114/153 (75%) qualified paediatric rheumatologists, 6/153 (3%) adult rheumatologists, 9/153 (6%) dually trained rheumatologists, 12/153 (8%) trainees, 12/153 (8%) rheumatology nurses and AHP. Forty six percent (70/153) of respondents do not have transition clinics in their centre, and only 56% have a defined transition pathway for their patients. Half of participants responded that there is a cross over period when the young person is seen by both the paediatric rheumatologist and adult rheumatologist together, and 39% of respondents stated that only the medical aspects and not psychosocial, educational and vocational are covered at transition appointments. The majority (62%) reported that no one in their centre has received any training in transitional care, although half of respondents use transition tools during the transitional care process, including online resources as self-training support. In only a minority of cases the transitional care process is supported by a multidisciplinary team. The most common barriers to transitional care were insufficient funding/support from the government or healthcare institutions, limited access to specialist multidisciplinary team, and lack of standardised protocols for transitional care. The need for incorporation of disease-specific modules to an update of the 2017 PReS/EULAR standards/recommendation for transitional care was highlighted by 62% of respondents.

**Conclusion:** A wide variation in current provision of transition services still exists worldwide. The 2017 recommendations highlighted the need for a written transition policy, yet less than half of respondents in our survey indicated that their department had a formal transition program. The EULAR/PReS recommendations include the need to secure funding for provision of transitional care, but unfortunately this remains a significant barrier to successful transitional care in 2024. Respondents support further refinement of the rheumatology transition standards/recommendations with inclusion of disease-specific modules.

**Patient Consent**

Not applicable (there are no patient data)

**Disclosure of Interest**

None Declared

### Disease outcome and transition

#### P127 Ready to leave? – Adolescents’ and parents’ perceptions of the transition from pediatric to adult rheumatology care

##### Anna Vermé^1,2^, Marika Wenemark^1,3,4^, Johanna Granhagen Jungner^1^, Eva Broström^1^, Cecilia Bartholdson^1,2^

###### ^1^Department of Women’s and Children’s Health, Karolinska institutet; ^2^Astrid Lindgren Children’s Hospital, Stockholm; ^3^Department of Health, Medicine and Caring Sciences, Faculty of Medicine and Health Sciences, Linköping University; ^4^East Region, Unit for Public Health and Statistics, Linköping, Sweden

####### **Correspondence:** Anna Vermé

*Pediatric Rheumatology 2024*, **22(2):**PReS24-ABS-1273

**Introduction:** In Sweden, about 2000 children suffer from Juvenile Idiopathic Arthritis (JIA) (1). Approximately half of them continue to have an active disease as they enter adulthood, and need to transfer to adult care (2, 3). This transfer is often perceived as difficult and creates a great deal of concern for adolescents and their parents (4).

**Objectives:** In this study, the aim was to investigate Swedish adolescents with JIA and their parents’ perceptions of readiness for transition from pediatric to adult rheumatology care.

**Methods:** The study was a cross-sectional quantitative study based on the questionnaire: Readiness for Transition Questionnaire (RTQ). There were 106 adolescents with JIA aged 14-18 and 96 parents answering the RTQ. Adolescent responsibility and parental involvement were, assessed separately and used in a combined measure of independent responsibility. Data were analyzed with descriptive statistics, non-parametric tests factor analyses, and logistic regression.

**Results:** Both adolescents and parents reported that the adolescents had difficulty taking over full responsibility for various healthcare behaviours such as booking specialty care appointments, calling to renew prescriptions, and communicating with medical staff on phone and were poorly prepared to do the transfer to adult care. In general, 16-18-year-old adolescents reported taking more responsibility and having lower parental involvement than 13-15-year-olds.The same results were shown in the combined measure of responsibility with different levels of parental involvement. The analysis showed that direct contact over phone with the healthcare system was the most challenging. In many of the other healthcare behaviours, the adolescents perceived themselves as taking major responsibility, while perceiving parents as often involved. The substantial progress in responsibility, compared between the younger and older adolescent groups, was for getting regular labs and attending medical appointments. For the more challenging activities, there was progress between the younger and older age groups, but still, less than 25 % of the older adolescents perceived major responsibility, with parents often/sometimes involved. There was a significant difference (p=0.005) between the age groups when reporting perceptions of overall readiness to transfer to adult care. In the younger group (13-15 years), 2% perceived that they were fully ready to transfer to adult care compared to 7% of the adolescents in the older group (16-18 years). Moreover, in the younger age group (13-15 years), 48% perceived that they were not ready to transfer to adult care compared to 24% in the older age group (16-18 years). Parents’ perceptions are congruent with the younger age group of adolescents. Three percent reported that they perceived their adolescents as being fully ready to transfer and half (49%) of the parental group reported that they did not perceive their adolescents as ready to transfer.

**Conclusion:** Both adolescents and parents report that the adolescents are moderately prepared to do the transfer to adult care and need more support. With the results from this study, it is possible to evidence base, customize, and optimize transitional care to create opportunities for adolescents to have a good transfer to adult care.

**Patient Consent**

Not applicable (there are no patient data)

**Disclosure of Interest**

None Declared

### Disease outcome and transition

#### P128 Early predictors of lethal outcome in patients with immunoinflammatory diseases admitted in the intensive care unit: the data of first day ICU admission

##### Natalia Abramova, Ilia Avrusin, Anastasia Kuleshova, Vyacheslav Chasnyk, Yuriy Aleksandrovich, Mikhail Kostik

###### Saint Petersburg State Pediatric Medical University, Saint-Petersburg, Russian Federation

####### **Correspondence:** Mikhail Kostik

*Pediatric Rheumatology 2024*, **22(2):**PReS24-ABS-1797

**Introduction:** Systemic immune-mediated diseases can affect multiple systems and organs and have extremely serious course and severe complications, that can cause what can cause multiple organ failure and death. Quite often these diseases can require admission to intensive care unit (ICU). For example, approximately 50% of patients with multisystem inflammatory syndrome associated with COVID-19 in children (MIS-C) need admission to ICU.

**Objectives:** The aim of the study was to find early predictors of death in patients with immune-mediated diseases admitted in the ICU.

**Methods:** The study included 51 patients (23 boys, 28 girls) with systemic immune-mediated diseases such as MIS-C, systemic juvenile idiopathic arthritis, systemic lupus erythematosus, and systemic vasculitis, aged from 7 months up to 17 years old, admitted to the ICU of the clinic of St. Petersburg State Pediatric Medical University in the period from 2007 to 2023. All patients were divided into 2 groups: 1 - with a fatal outcome, 2 - recovered. We assessed routine clinical, laboratorial, instrumental parameters and ongoing treatment on the first day after admission to the ICU.

**Results:** Patients with an unfavorable outcome were significantly older and were admitted to the ICU later than recovered patients (28 vs. 7 days, p=0.006), and also had a longer stay in the ICU (39 vs. 7 days, p=0.0006).

Among patients with fatal outcome, the main distinguishing clinical and instrumental criteria were: reduced diuresis (1.1 vs. 1.9 ml/kg/hour, p=0.015), SpO2 (87 vs. 95, p=0.018), thrombocytopenia (87 vs. 213x10^9^/l, p=0.001), hypoalbuminemia (24 vs. 30.7 g/l, p=0.009), increased creatinine level (131 vs. 50.8 mmol/l, p=0.021), tendency to acidosis: pH (7.35 vs. 7.42, p=0.026), BE (-4.4 vs. -1.2 mmol/l p=0.017), HCO3 (20.2 vs. 23.4 mmol/l, p=0.031).

According to the SOFA scale, the scores in died patients were higher, indicating the development of multiple organ failure (6.5 vs. 2 points, p=0.0006). Such patients more often required oxygen support (81.8% vs. 42.1%, p=0.020), mechanical ventilation (41.7% vs. 13.2%, p=0.032), and inotropic support (33.3% vs. 18.9%, p=0.298). Died patients more often needed therapy with biological drugs (15.4% vs. 0%, p=0.015) and transfusions of fresh frozen plasma (50% vs. 16.7%, p=0.021). According to these differences we found the parameters that can be used as the early predictors of lethal outcome.

**Conclusion:** Identified clinical and laboratory differences, such as reduced rates of diuresis, hypoxia, thrombocytopenia, hypoalbuminemia, increased creatinine levels, and a tendency to acidosis, can be used as early markers of an unfavorable outcome among patients with immune-mediated diseases. This may allow timely identification of patients at high risk of an unfavorable outcome of the disease and, accordingly, ensure the most careful monitoring of vital functions and promptly prescribe appropriate treatment.

**Patient Consent**

Not applicable (there are no patient data)

**Disclosure of Interest**

None Declared

### Disease outcome and transition

#### P129 Juvenile idiopathic arthritis in adults: course of the disease and transitional care – results from a retrospective polish cohort study

##### Tomasz Kosakowski^1^, Violetta Opoka-Winiarska^2^

###### ^1^Świętokrzyskie Rheumatology Center,, St. Lukas Hospital, Końskie; ^2^ Department of Pediatric Pulmonology and Rheumatology, Medical University of Lublin, Lublin, Poland

####### **Correspondence:** Tomasz Kosakowski

*Pediatric Rheumatology 2024*, **22(2):**PReS24-ABS-1163

**Introduction:** The course of juvenile idiopathic arthritis (JIA) often continues into adulthood. The adult population with JIA is highly heterogeneous. Less than half of the patients meet the classification criteria for adult inflammatory diseases. There is a strong desire for rheumatology-specific guidelines for transition and for these to be implemented across pediatric, adolescent and adult rheumatology healthcare settings (1).

**Objectives:** To determine how patients fulfil classification criteria for adult rheumatic diseases, evaluate their outcomes in the biologic era. To assess the course of JIA in adulthood. Analysis of transitional care for patients diagnosed with JIA.

**Methods:** Population-based, multicenter, reprospective JIA Polish cohort. 54 eligible patients diagnosed with JIA treated between the years 2008-2018. Data were collected from available pediatric and adult medical records, outpatient clinic, and hospitalizations.

Inclusions criteria: Patients older than 18 years and with more than 5 years of disease duration were included.

The coexistence of the relationship between the course of the disease was analyzed, taking into account the data on the differences among individual forms of JIA, fulfilment of adult rheumatic diseases classification criteria. The rediagnosis was made if the adult patient with JIA met one of the following criteria: 2010 ACR/EULAR rheumatoid arthritis (RA) criteria, modified New York criteria for ankylosing spondylitis (AS), 2009 Assessment of Spondyloarthritis International Society (ASAS) for axial spondyloarthropathy (axSpA), CASPAR criteria for psoriasis arthritis (PsA).

**Results:** Of the adult JIA patients, 54% were rediagnosed and met the classification criteria for adult inflammatory diseases. The remaining 46% of the patients were left with a JIA diagnosis. RA and AS were most commonly diagnosed after reaching adulthood.

Patients with systemic JIA mostly remained JIA diagnosis and to a lesser extent into RA – only 25% of them fulfilled criteria for RF (-) polyarthritis. 75% of the patients with RF (+) polyarthritis and 33% of the patients with RF (-) polyarthritis matched criteria for RA. 50% of the patients with extended oligoarthritis and 18,5% of the patients with persistent oligoarthritis were classified as RA. Patients with enthesitis-related arthritis (ERA) fulfilled criteria for axSpA in 25% and AS in 50%. The most diverse course was observed in persistent oligoarthritis, which can evolve into any type of arthritis. 46% of all the patients with JIA maintained this classification in adults.

**Conclusion:** We confirmed the adult population with JIA is very heterogeneous. The existence of a relationship between some forms of JIA patients and the diagnosis of the disease in adulthood was found. After revision of the diagnosis from JIA to adult-onset inflammatory disease the patients require ongoing, long-term treatment and developmentally appropriate care during and beyond adolescence to ensure optimal functioning in adulthood.

**Patient Consent**

Not applicable (there are no patient data)

**Disclosure of Interest**

None Declared

**Reference**

1. *Foster HE, Minden K, Clemente D i wsp. EULAR/PReS standards and recommendations for the transitional care of young people with juvenile-onset rheumatic diseases. Ann Rheum Dis 2017; 76: 639-646.*

**Table 1 (Abstract PReS24-ABS-1163):** The change of JIA patients diagnosis in adulthood

Diagnosis	Men ***n***=11 (%)	Women ***n***=43 (%)	Together ***n***=54 (%)
**JIA**	3 (27,27)	22 (51,16)	25 (46,30)
**RA**	1 (9,09)	13 (30,23)	14 (25,93)
**AS-axial**	4 (36,36)	3 (6,98)	7 (12,96)
**axSpA**	1 (9,09)	4 (9,30)	5 (9,26)
**PsA**	1 (9,09)	1 (2,33)	2 (3,70)
***Undifferentiated***	1 (9,09)	0 (0,00)	1 (1,85)

*χ*^*2*^*=11,70487, df=5, p=0,039 p<α; α=0,05*

### Disease outcome and transition

#### P130 Transition care programme for chronic rheumatic diseases is still a challenge: evidence from the 2023 ERN-ReCONNET transition of care task force survey.

##### Edoardo Marrani^1^, Mojca Zajc Avramovic^2^, Diana Marinello^3^, Rosaria Talarico^3^, Chiara Baldini^3^, Eva Collado^4^, João Eurico Fonseca^5^, Linda Schraven^6^, Filipa Oliveira Ramos^7^, Paola Triaggese^8^, Arjan Vissink^9^, Marta Mosca^3^, Tadej Avcin^2^, Gabriele Simonini^1^ on behalf of ERN ReCONNET

###### ^1^Rheumatology Unit, ERN-ReCONNET Center, IRCCS Meyer Children’s Hospital, Firenze, Italy; ^2^Department for Allergology, Rheumatology and Clinical Immunology, Children’s Hospital, University Medical Centre Ljubljana, Ljubljana, Slovenia; ^3^Rheumatology Unit, ERN-ReCONNET Center, Azienda Ospedaliero Universitaria Pisana, AOUP, Pisa, Italy; ^4^Asociación Nacional del Síndrome de Ehlers-Danlos e Hiperlaxitud (ANSEDH), ERN- ReCONNET European Patient Advocacy Group (ePAG), Murcia, Spain; ^5^Instituto de Medicina Molecular, Faculdade de Medicina, Universidade de Lisboa, ULS Santa Maria,, Lisbon, Portugal; ^6^Federation of European Scleroderma Associations (FESCA), Amsterdam, Netherlands; ^7^Hospital Universitário Santa Maria, Faculdade de Medicina, Universidade de Lisboa, Lisbon, Portugal; ^8^Department of Systems Medicine, University of Rome Tor Vergata, Roma, Italy; ^9^Department of Oral and Maxillofacial Surgery, University of Groningen and University Medical Center Groningen, Groningen, Netherlands

####### **Correspondence:** Edoardo Marrani

*Pediatric Rheumatology 2024*, **22(2):**PReS24-ABS-1520

**Introduction:** Rare diseases, defined in the European Union as those affecting fewer than 1 in 2000 individuals, collectively impact millions across the EU. European Reference Networks (ERNs) have emerged to address the complexities of rare diseases by connecting healthcare providers (HCPs) across Europe. ERNs aim to facilitate discussions, review diagnoses, conduct research, and establish guidelines for treatments. ERN ReCONNET focuses on rare connective tissue and musculoskeletal diseases, addressing ten conditions with dedicated efforts. Despite these initiatives, challenges persist in transitioning care, especially for paediatric-onset rare diseases.

**Objectives:** to obtain a snapshot of the transitional care programme for rheumatic diseases across Europe

**Methods:** To assess transitional care standards within ERN ReCONNET, in September 2023, a Transition of Care Task Force comprising of expert clinicians, patient advocates, and coordination team members was formed in 2023. The task force developed a 45-item survey on the EUSURVEY platform to assess transitional care practices and opinions. Ethical approval was not required. In November 2023, an offcial collaboration between ERN ReCONNET and ERN RITA facilitated distribution of the survey to relevant centers. The survey was available to be filled online from 27 December 2023 till 15 March 2024.

**Results:** Sixty-seven responses were collected from 59 centers across 20 European countries. Respondents included adult and paediatric rheumatologists, internal medicine specialists, and geneticists. Transition policies varied among centers, but 29 (43%) respondents claimed to have a formal process. Designated staff members mainly comprised doctors overseeing transition coordination. Thirty (44%) respondents claim to follow clinical guidelines, 18 (27%) reported lacking awareness or formal procedures. Paediatric rheumatologists were often identified as key figures in initiating transition processes. Joint clinics involving both adult and paediatric HCPs were available in 21 centers. The age of patients for starting the transition of care was identified to be 15-18 years old for most of the respondents (n=37, 63%). Patient opinion was a key factor for transition for only two respondents.

**Conclusion:** The survey revealed heterogeneity in transition practices and resources across ERN ReCONNET centers. Challenges in patient identification, readiness evaluation, and coordination were made evident. Lack of guidelines and engagement from adult centers posed significant obstacles. Despite these challenges, respondents generally perceived the efficacy of ongoing transition processes positively. The point of view of patients to confirm the efficacy of the program remains an unmet need.

**Patient Consent**

Not applicable (there are no patient data)

**Disclosure of Interest**

None Declared

### Disease outcome and transition

#### P131 Rheumatological diseases and inflammatory bowel disease in childhood

##### Özen Taş^1^, Fatma Aydın^1^, Zarife Kuloğlu^2^, Ceyda Tuna Kırsaçlıoğlu^2^, Nur A. Çelik^3^, Onur Bahçeci^1^, Betül Öksüz Aydın^1^, Zeynep B. Özçakar^4^

###### ^1^Paediatric Rheumatology; ^2^Paediatric Gastroenterology; ^3^Paediatric health and diseases; ^4^Paediatric Nephrology and Rheumatology, Ankara University of Medicine, Ankara, Türkiye

####### **Correspondence:** Özen Taş

*Pediatric Rheumatology 2024*, **22(2):**PReS24-ABS-1199

**Introduction:** Rheumatological diseases in childhood are systemic diseases that occur on the basis of auto-immunity or inflammation and among rheumatological diseases, especially familial Mediterranean fever (FMF), juvenile idiopathic arthritis (JIA) and chronic nonbacterial osteomyelitis (CNO) may be accompanied by inflammatory bowel disease (IBD). When there is no knowledge of this association, the treatments applied may not be sufficient and/or treatments given for rheumatological disease may even lead to aggrevation of IBD findings. Thus, early identification of an association is crucial for the correct management of the diseases.

**Objectives:** The aim of this study is to show the frequency of coexistence of rheumatological diseases and IBD. We also aimed to investigate in which cases inflammatory bowel disease should be suspected in children with rheumatological diseases.

**Methods:** Electronic medical records of the patients who were followed up between 2012 and 2024 with a diagnosis of rheumatological disease in the Department of Pediatric Rheumatology of Ankara University Faculty of Medicine and who were diagnosed with IBD were reviewed retrospectively.

**Results:** Between 2012 and 2024, 20 (3%) of 650 FMF patients, 3 (7.5%) of 40 CNO patients and 2 (1.2%) of 170 JIA patients were diagnosed with IBD. While 12 (48%) of the patients received a rheumatologic diagnosis as the initial diagnosis, 8 (32%) of them were initially diagnosed with IBD and then referred to rheumatology for their symptoms. In 4 patients (16%), IBD and rheumatologic disease were diagnosed simultaneously.

The median age at the diagnosis of rheumatologic diseases was 6.3 years (IQR:8.8). Seventeen patients (68%) were diagnosed with FMF, 1 (4%) with FMF and CRMO, 2 (8%) with FMF and sacroiliitis, 2 (8%) with CRMO and 2 (8%) with JIA. When the types of IBD are evaluated, it was seen that 10 patients (40%) had Crohn’s disease, 11 patients (44%) ulcerative colitis (UC) and 3 patients (12%) indeterminate colitis. The median age at the diagnosis of IBD was 11.0 years (IQR:11.3). The most common symptoms for the diagnosis of IBD were diarrhea (76%) and abdominal pain (40%). In our rheumatologically followed patients, the most common symptoms that led to suspicion of IBD diagnosis were diarrhea (40%), abdominal pain (36%) and fever (24%). In addition to these obvious symptoms, we had 4 patients who presented with a more vague symptoms and were diagnosed with IBD. These symptoms were treatment-resistant iron deficiency anemia in 1 patient, anal abscess in 1 patient, weight loss in 1 patient and short stature 1 patient.

**Conclusion:** Rheumatologic diseases and IBD share similar pathologic pathways and clinical findings and IBD can accompany to various rheumatological diseases. The diagnosis of IBD should be considered in the presence of rare and atypical symptoms.

**Trial registration identifying number:** This study doesn’t report a controlled health care intervention.

**Date of birth:** janvier 01

**Patient Consent**

Not applicable (there are no patient data)

**Disclosure of Interest**

None Declared

**References**

1. Bindu S, Dandapat S, Manikandan R, et al. Prophylactic and therapeutic insights into trained immunity: A renewed concept of innate immune memory. Hum Vaccines Immunother 2022;18(1):204238.
2. Zhang Y, Yang W, Li W, et al. NLRP3 Inflammasome: Checkpoint Connecting Innate and Adaptive Immunity in Autoimmune Diseases. Front Immunol 2021; 12:732933.

### Disease outcome and transition

#### P132 Transitional care experiences in young people living with rheumatic disease: a narrative based medicine study

##### Andrea Tomasini^1^, Antonella Celano^2^, Antonnella Insalaco^3^, Maria Alessio^4^

###### ^1^Italian national Association People with Rheumatic and Rare Diseases - APMARR APS ETS, Rome; ^2^Italian national Association People with Rheumatic and Rare Diseases - APMARR APS ETS, Lecce; ^3^Rheumathology, IRCCS Ospedale Pediatrico Bambino Gesù, Rome; ^4^Department of Pediatrics, Rheumatology Unit, University of Naples Federico II, Naples, Italy

####### **Correspondence:** Andrea Tomasini

*Pediatric Rheumatology 2024*, **22(2):**PReS24-ABS-1494

**Introduction:** Transitional care, vital for individuals with chronic childhood illnesses, facilitates the shift from pediatric to adult healthcare. This phase requires meticulous preparation and coordination among healthcare providers to ensure therapy continuity. Despite established guidelines, the patient’s experiential perspective has often been overlooked.

**Objectives:** To investigate the experience of illness and suggest constraints, opportunities, successes and difficulties in the path examined, in terms of daily life, assistance and perceived care as well as health performance, APMARR has launched a first narrative medicine study aimed at investigating the experience of people who have embarked on the transition path.

**Methods:** Eight narrative interviews were conducted with individuals with rheumatological diseases, seeking diverse perspectives to saturate the sample. Participants were recruited through collaboration with pediatric rheumatology departments of the IRCCS Bambino Gesù in Rome and the University of Naples. The textual analysis of the approximately 400 minutes produces some evidences that can be described and a frame hypothesis suggested.

**Results:** The study revealed narratives predominantly characterized by imperfect transitions, where individuals maintained significant reliance on pediatric care even after transitioning to adult services. Limited preparation and communication between pediatricians and adult physicians hindered trust and rapport with the new healthcare provider. Diagnostic reviews by adult doctors were often perceived as an expropriation of identity, as individuals had negotiated and shaped their lives based on the diagnosis from their pediatrician.

Themes of imperfect transitions emerged, highlighting the profound understanding of their condition and treatment among participants. However, they struggled with the contrasting care styles between pediatric and adult settings, often leading to a return to pediatric care due to the lack of continuity and personalized care in adult services.

**Conclusion:** The study underscores also the importance of considering the patient’s perspective in transitional care, highlighting the need for improved preparation and communication strategies to facilitate smoother transitions and better outcomes for individuals with chronic illnesses transitioning from pediatric to adult healthcare.

Drawing on insights from anthropology and social sciences, the study advocates for a multidisciplinary approach to transitional care, emphasizing psychological and social support alongside logistical aspects. It prompts a critical examination of adolescent health discourse and the concept of adulthood, urging healthcare professionals to reevaluate existing transitional care frameworks to better meet the needs of adolescents with chronic illnesses.

**Trial registration identifying number:** I haven’t submitted a case report

**Patient Consent**

Not applicable (there are no patient data)

**Disclosure of Interest**

None Declared

### Disease outcome and transition

#### P133 Transition readiness assessment in Portuguese adolescents and young adults with pediatric-onset rheumatic diseases: a single-center study

##### Joana B. Monteiro^1^, Francisca Aguiar^1,2^, Ana Sofia Figueiredo^3^, Sara G. Paulino^4^, Ana Sofia Teixeira^5^, Sara Ganhão^2^, Mariana Rodrigues^1,2^, Iva Brito^1,2^

###### ^1^Faculty of Medicine of Porto University; ^2^Pediatric and Young Adult Rheumatology Unit, ULS São João; ^3^Pediatrics Department, ULS Trás-os-Montes e Alto Douro; ^4^Pediatrics Department, ULS São João; ^5^Faculty of Engineering, University of Porto, Porto, Portugal

####### **Correspondence:** Francisca Aguiar

*Pediatric Rheumatology 2024*, **22(2):**PReS24-ABS-1057

**Introduction:** The achievement of a successful transition process is crucial for adolescent and young adult (AYAs) patients with chronic rheumatic immune-mediated disorders, since their likelihood of maintaining disease activity in adulthood is high and those who achieve remission can experience damage or drug-related complications. Nonetheless, several studies suggest that the transition process has not been reaching its desired objectives. A high proportion of these patients do not make a successful transfer to adult rheumatology services and are therefore particularly at risk of nonadherence to health care, loss of follow-up and, consequently, unfavorable outcomes. To achieve successful rates of transition to adult care, the acquisition of disease knowledge and disease self-management skills is of extreme importance [12, 13]. To address this issue, the use of standardized transition readiness assessment tools has been suggested.

**Objectives:** In this study, we aimed to assess the transition readiness levels amongst patients with childhood-onset rheumatic diseases. Additionally, we sought to identify and analyze predictive factors associated with better transition readiness skills in AYAs.

**Methods:** This is a monocentric cross-sectional study that includes patients between 14 and 26 years of age who attended outpatient pediatric and young adult rheumatology appointments in a Portuguese tertiary centre, between October and December of 2023 and that were diagnosed with an immune-mediated rheumatic disease before reaching 18 years of age, with at least 1 year of disease duration. Patients were presented with a questionnaire that contained demographic and clinical questions, TRACS (Questionário de Preparação da Transição para a Autonomia nos Cuidados de Saúde) questionnaire – a validated Portuguese version of the Transition Readiness Assessment Questionnaire (TRAQ), and Hospital Anxiety and Depression Scale (HADS) questionnaire. Data was analyzed to assess the significant associations between the different variables and transition readiness outcome measured by the TRACS. Descriptive statistics, statistical comparisons and logistic regression analysis were performed with R version 4.3.2. A significance level of p < 0.05 was considered for statistical significance.

**Results:** A total of 69 patients with a median age of 20 [17.5-22.5] were included in this study. The median TRACS score was 4.41 [4.09-4.74]. Significantly higher TRACS scores were observed in patients who were female, 18 years of age or older, had a higher level of education, were employed, had active disease or that belonged to middle-class (when compared to patients belonging to upper-middle class). The logistic regression analysis demonstrated that being a female or having an educational status equal to 12th grade or superior emerged as predictors of higher transition readiness levels.

**Conclusion:** Our study identified female sex and higher level of education as predictors of increased transition readiness levels. Therefore, healthcare providers should consider these variables when assessing patients for transition readiness and focus on improving transition process, especially in male and less educated AYAs.

**Patient Consent**

Yes, I received consent

**Disclosure of Interest**

None Declared

### Disease outcome and transition

#### P134 Evaluating the transition process in patients with familial Mediterranean fever

##### Betül Sözeri^1^, Sıla Atamyıldız Uçar^1^, Yunus Emre Bayrak^2^, Şeyma Türkmen^1^, Nihal Şahin^2^, Hafize Emine Sönmez^2^

###### ^1^Department of Pediatric Rheumatology, Umraniye Training and Research Hospital, Istanbul; ^2^Department of Pediatric Rheumatology, Kocaeli University Faculty of Medicine, Kocaeli, Türkiye

####### **Correspondence:** Sıla Atamyıldız Uçar

*Pediatric Rheumatology 2024*, **22(2):**PReS24-ABS-1062

**Introduction:** Transition is the planned process of pediatric patients moving from child-centered to adult-oriented treatment.

**Objectives:** Transitional care is crucial for patients with chronic diseases. Because most patients referred to the pediatric rheumatology clinic are diagnosed with familial Mediterranean fever (FMF) in our region, it is essential to transition these patients to adult care for the continuity of their treatment. The objective of this study was to assess the readiness of patients with FMF for the transition process.

**Methods:** This was a cross-sectional study. All patients were surveyed regarding their awareness of and willingness to undergo transitional care. The Transition Readiness Assessment Questionnaire (TRAQ) was administered to all participants. Colchicine-responsive and colchicine-resistant cases were compared. Furthermore, patients who had been transitioned to adult care were contacted via phone calls and questioned about whether they attended their follow-up appointments regularly.

**Results:** A total of 106 patients were enrolled. Of them, 63 (59.4%) were girls and 43 (40.6%) were boys. Seventy-two (67.9%) patients were colchicine-responsive and 34 (32.1%) were colchicine-resistant. The median age of the patients was 17 (15-22) years. The mean total TRAQ score was 3.82 ± 0.59 (2-5). When we compared TRAQ scores between colchicine-responsive and colchicine-resistant cases; the median TRAQ score was 3.94 ± 0.55 in colchicine-responsive and 3.56 ± 0.61 in colchicine-resistant patients (p=0.001). Furthermore, 15 patients who had been transitioned to adult care were questioned about whether they attended their follow-up appointments regularly. Of these, 7 patients reported that they attended their follow-up appointments regularly, whereas 8 did not adhere to their scheduled visits.

**Conclusion:** Assessing the readiness of patients with FMF for transition care will enhance their awareness and help determine the optimal time for transition. A more severe illness can influence the preparatory processes of the disease.

**Date of birth:** 25.04.1989

**Patient Consent**

Yes, I received consent

**Disclosure of Interest**

None Declared

### Disease outcome and transition

#### P135 A complicated case of a 7.5-years old girl with mixed connective tissue diseases: polymyositis, polyarthritis and scleroderma, diabetes mellitus, and celiac disease from Armenia

##### Ani Margaryan, Aida Papikyan, Hrachuhi Ghazaryan

###### Wigmore Hospital, Yerevan, Armenia

####### **Correspondence:** Ani Margaryan

*Pediatric Rheumatology 2024*, **22(2):**PReS24-ABS-1416

**Introduction:** Mixed connective tissue disease (MCTD) is a rare condition overlapping at least two connective tissue diseases (CTD). There are no randomized controlled trials to guide the treatment of MCTD. It is well known that steroids can increase blood glucose levels and result in diabetes mellitus (DM).

**Objectives:** A 7.5-year-old girl with a history of 2.5 years DM was admitted to the “Wigmore” Hospital for episodes of uncontrolled hyperglycemia and frequent hypoglycemia. On admission, she complains of fatigue, weakness, difficulty in movement during the last 9 months, constipation. Objective examination revealed poor growth, dry, pale skin without rash, poorly developed subcutaneous fat, hepatosplenomegaly, difficulties in movements, inability to stand up from a lying/sited position, inability to raise and hold the head in a lying position, inability to keep the legs elevated, painful and limited movements in the shoulders, arms, wrists, knee joints, also swelling in the PIP joints. From labs: CBC, CRP, Cr-normal, low Vitamin D and ferritin, CK-4468 U/I, ALT-133, AST-196, anti-tTg-IgA: 89, EMA: 18.5ME/ml, RF and ANA positive. MRI of buttock and thigh: muscle swelling and infiltration. Capillaroscopy: no any capillaries. The diagnosis of MCTD was made and the treatment with IVIG and methotrexate was started. On 3rd day the child was transferred to PICU with fever, pleuritis, pericarditis, respiratory failure, hypercapnia. The CT-angiography was performed: multiple small renal abscesses. The MTX was stopped, Ceftriaxone was initiated. UC’ E coli, BC: no growth. The antibiotics were switched to Ciprofloxacin according to sensitivity results. The child’s condition worsened, she was intubated. The steroid pulse therapy and Cyclosporine started, MTX restarted, IVIG continued. After 4 months of hospitalization the child had no stiffness, no pain, no limitation of movements, was discharged from hospital. On a 6-month follow-up: clinical and laboratory remission, continues Cyclosporine, MTX, Mpred 4mg/day.

**Conclusion:** The presentation aims to underline the importance of early identification of MCTD for the possible best outcome, to discuss the difficulties of the treatment of patients with autoimmune diseases and comorbidities such as DM, and to emphasize the importance of managing complex cases in a multidisciplinary team.

**Date of birth:** décembre 1

**Patient Consent**

Yes, I received consent

**Disclosure of Interest**

None Declared

### Disease outcome and transition

#### P136 Dress syndrome - case report of a 15-year-old patient with JIA

##### Dusana Gensor^1^, Vladimir Zolak^1^, Milos Jesenak^1^, Branislav Slenker^1^, Pavel Bician^2^, Peter Szepe^3^, Peter Banovcin^1^

###### ^1^Department of Pediatrics, Jessenius Faculty of Medicine of Comenius University Bratislava, University Hospital in Martin, Martin; ^2^Department of Pediatric Hematology and Oncology, Children University Hospital, Banska Bystrica, Banska Bystrica; ^3^Constitution of pathological anatomy, Jessenius Faculty of Medicine of Comenius University Bratislava, University Hospital in Martin, Martin, Slovakia

####### **Correspondence:** Dusana Gensor

*Pediatric Rheumatology 2024*, **22(2):**PReS24-ABS-1159

**Introduction:** DRESS syndrome (Drug Reaction with Eosinophilia and Systemic Symptoms syndrome) is a rare adverse drug reaction that can result in a life-threatening condition and can be fatal if inadequately treated. It is a severe form of idiosyncratic drug reaction that manifests clinically with a longer latency from the medication administered (most commonly 2-6 weeks). The disease has a variable clinical course, with prolonged febrile state, cutaneous exanthema, lymphadenopathy and eosinophilia being the most common manifestations. The disease can have a mild, but also severe course. The severe course is most often manifested by systemic manifestations with subsequent organ damage.

**Objectives:** The authors provide case report of a 15-year-old patient with a severe form of DRESS syndrome after Sulfasalazine, manifesting as lymphoproliferation.

**Conclusion:** DRESS syndrome is one of the rare yet severe field reactions. The reaction manifests in predisposed individuals after exposure to risky drugs. Despite the typical clinical symptoms, it is important to exclude an infectious cause and a paraneoplastic process in patients in particular. Early differential diagnosis, including subsequent adequate therapy of DRESS syndrome, will prevent the development of possible organ damage.

**Patient Consent**

Yes, I received consent

**Disclosure of Interest**

None Declared

### e-health and digital health applications

#### P137 DanBioPæd: a nationwide registry of children and adolescents with juvenile idiopathic arthritis

##### Anne Estmann Christensen^1^, Peter Toftedal^1^, Mia Glerup^2^

###### ^1^Hans Christian Andersen Children Hospital, Odense University Hospital, Odense; ^2^Department of Pediatrics, Aarhus University Hospital, Aarhus, Denmark

####### **Correspondence:** Anne Estmann Christensen

*Pediatric Rheumatology 2024*, **22(2):**PReS24-ABS-1508

**Introduction:** DanBioPæd is a new nationwide registry including all children and adolescents diagnosed with juvenile idiopathic arthritis (JIA) in Denmark according to the ILAR criteria.

**Objectives:** The main purpose is to capture clinical data provided from both health personnel and patients/parents as part of the daily clinical care. In addition, the registry will be a powerful source for monitoring clinical treatment quality and for research.

**Methods:**

**Study population**: All children diagnosed with JIA in Denmark are treated in a hospital setting and all patients are enrolled in the DanBioPæd registry regardless of disease course and medical treatment. Clinical data is registered electronically by the treating pediatric rheumatologist at every clinical visit. Patient/parent-reported outcomes are collected before each appointment via a web-based platform. The prospective evaluations of the temporomandibular joints are recorded in the same registry by the orthodontists.

**Main variables**: Core variables including age and JIA subtype at diagnosis, serological markers and family history are registered. Objective measures of disease activity by the physician, C-reactive protein (CRP) or erythrocyte sedimentation rate (ESR), presence of uveitis since the last visit and treatment details are recorded at every visit. Information from imaging (X-ray and MRI) is recorded. Patient/parents report pain and global assessment on a visual analog scale at every visit and the Juvenile Arthritis Multidimensional Assessment Report (JAMAR) once a year. Juvenile Arthritis Disease Activity Score (JADAS) is calculated at every visit. Additionally, the platform provides information on the cumulative joints visualized on a homunculus and injections performed in each specific joint with corresponding dates.

**Results:** Patient recruitment started in two centers in September 2022 as part of a pilot study. National approvement for the nationwide registry was achieved in 2023 and all 6 centers are now including patients. Currently, there are 1130 patients included in the inception cohort.

**Conclusion:** The nationwide DanBioPæd registry for all pediatric patients diagnosed with JIA is an excellent tool for daily clinical care. The registry will provide valuable opportunities for conducting research on both a national and international scale, as well as for monitoring clinical quality in the future.

**Patient Consent**

Not applicable (there are no patient data)

**Disclosure of Interest**

None Declared

### e-health and digital health applications

#### P138 ChatGPT vs. pediatric rheumatologists: a quest for excellence in pediatric rheumatology

##### Figen Çakmak, Nuray Aktay Ayaz

###### Pediatric Rheumatology, Istanbul Faculty of Medicine, Istanbul, Türkiye

####### **Correspondence:** Figen Çakmak

*Pediatric Rheumatology 2024*, **22(2):**PReS24-ABS-1748

**Introduction:** Importance Chat Generative Pre-Trained Transformer (ChatGPT) has shown promising performance in various fields, including medicine, but its accuracy on subject-specific medical questions, especially in pediatric rheumatology, is still uncertain.

**Objectives:** With this study, we aimed to investigate the ability of ChatGPT to correctly answer pediatric rheumatology questions asked in specialty exams in our country. This study, which focuses on how accurately and effectively the model can answer the exam questions set by experts, can help understand the role of artificial intelligence in pediatric rheumatology.

**Methods:** The examinations were scanned and questions on pediatric rheumatology that had been asked in specialist examinations over the last 10 years were collected. From the collected questions, 35 questions were randomly selected and forwarded to the ChatGPT 3.5 version. In the original exam version, we asked ChatGPT the questions with five options and explained that there was only one option. The answers were recorded and the performance of ChatGPT was evaluated. The incorrectly answered questions were classified according to their main themes and question formats, and frequently recurring themes were identified. The main topics were: inflammatory joint diseases, inflammatory connective tissue diseases, autoinflammatory diseases, vasculitides and rheumatic manifestations of non-rheumatic diseases.

**Results:** ChatGPT achieved 60% success by answering 21 out of 35 questions correctly. Success performance was 100% in inflammatory connective tissue diseases, 57% in autoinflammatory diseases, 55% in vasculitis, 50% in inflammatory joint diseases (JIA) and 50% in rheumatic manifestations of non-rheumatic diseases, respectively. While the success rate for questions based on clinical solutions was 29%, the success rate for questions based on general information was 89%.

**Conclusion:** With the knowledge base currently available, ChatGPT’s performance in answering questions may unfortunately fall short of the success of pediatric rheumatologists who achieve very high exam scores. In particular, the low performance in solving patient questions in a clinical setting suggests that artificial intelligence models cannot replace clinicians over an extended period of time. Artificial intelligence models may perform better in collaboration with clinicians.

**Date of birth:** avril 18,

**Patient Consent**

Not applicable (there are no patient data)

**Disclosure of Interest**

None Declared

### e-health and digital health applications

#### P139 The effect of therapeutic exercise program, via telerehabilitation, on the functional ability and quality of life in adult patients with Juvenile Idiopathic Arthritis (JIA) preliminary announcement

##### Maria Stavrakidou^1^, Alejandros Garyfallos^1^, Theodoros Dimitroulas^1^, Maria Trachana^2^, Artemis Koutsonikoli^2^, Kyriaki Spanidou^3^, Despina Dimopoulou^1^

###### ^1^Clinic of Connective Tissue Diseases, Clinic for Adult Patients with Pediatric Rheumatic Diseases; ^2^First Dept of Pediatrics, Pediatric Immunology and Rheumatology Referral Center, Hippokration Hospital; ^3^Asklepeio Physiotherapy Clinic, Aristotle University of Thessaloniki , Thessaloniki, Greece

####### **Correspondence:** Maria Stavrakidou

*Pediatric Rheumatology 2024*, **22(2):**PReS24-ABS-1200

**Introduction:** A significant proportion of children as well as adults with inflammatory arthritis having balance disorders. Exercise therapy significantly improves musculoskeletal symptoms. However, proprioceptive balance exercises might be more effective than strength exercises for improving function. Home exercise interventions via telerehabilitation have gained more popularity in recent years and even more so due to the COVID-19 pandemic.

**Objectives:** Primary: Investigation of the effect of a therapeutic exercise program, via telerehabilitation, on the functional capacity and quality of life of adult patients with JIA. Secondary: 1) The evaluation of balance as a factor of functionality and overall quality of life of adult patients with JIA 2) The comparison with healthy individuals of the same age group, before and immediately after the intervention.

**Methods:** At six adult patients with JIA aged ≥18 years with MDVAS (0-2) (patients’ group-PG) personalized program of therapeutic exercises was applied, through telerehabilitation (OpenTera platform), for 12 weeks, three times per week (two via telerehabilitation and one time by themselves, after being reminded). All patients were evaluated for functional capacity through balance tests Y Balance Test (YBT), Functional Reach Test (FRT) and Stabilometry and with the HAQ questionnaire. Quality of life was assessed by SF36. For 5 healthy subjects (CG), the same test procedure was followed and the results were compared with those of the PG.

**Results:** In measurements and scores of the questionnaires after the telerehabilitation program, the PG showed improvement and approached the values of the CG, despite the initial difference. In detail the PG improved in YBT with symmetry index in Right lower extremity 23.47% (mean from 2.125 before, to 2.628 after) and in Left lower extremity 15.67% (mean from 2.165 before, to 2.506 after) approaching the CG (Right mean 2.628 and Left mean 2.62). The same was observed in the FRT with an improvement of 28.17% (mean from 35.5cm before, to 45.5cm after) approaching the CG (mean 45.9cm). In SWAY Stabilometry with eyes open there was an improvement of 37,07% (mean 3024,73 mm^2^ before, to 1903.36 mm^2^ after) and with eyes closed a 54.26% improvement (mean 5480.55 mm^2^ before, to 2606.78 mm^2^ after), values higher than those of the CG (2361.1 mm^2^ and 3484.08 mm^2^ respectively). There was also an improvement in the HAQ score with a total score from 0.375 (mildly impaired) before to 0.25 after (normal) and a global rating (ten-point scale) of 0.11 before to 0.09 after the program. Finally, in SF36 physical health improved by 1.62% (before 77.185% and after 78.436%) approaching the CG value of 88.5% and mental health by 3.44% (before 75.858 and after 78.464%), a value better than that of the CG (67.0415%).

**Conclusion:** Our first findings were indicative for effectiveness of the therapeutic program via telerehabilitation on the functional capacity and quality of life of adults patients with JIA. This encouraged us to continue the study to a larger number of patients.

**Trial registration identifying number:** Bioethics Committee: Protocol Number 201/2023.

Scientific Council Hippokration Hospital, Thessaloniki, Greece Protocol Number: 33849 and Research Paper code: 23-ΕΔ-11

**Patient Consent**

Yes, I received consent

**Disclosure of Interest**

None Declared

### e-health and digital health applications

#### P140 Empowering health professionals participation in cannabis & hemp data collection through the Budsinfo™ Cerium™ education platforms

##### Jahan P. Marcu^1,2^, Philip Molloy^1^, Kayla Pray^1^, John H. Simon^1^, Shannon Heaning^1^, Matthew Wabwire^1^, Teresa A. Simon^1^

###### ^1^Physicians Research Center, Toms River; ^2^Marcu Enterprises, Jersey City, United States

####### **Correspondence:** Jahan P. Marcu

*Pediatric Rheumatology 2024*, **22(2):**PReS24-ABS-1568

**Introduction:** Cannabis and cannabis-derived products (CCDPs) are generally not recommended for children or adolescents. The European drug report (2023) showed that 15.5% of European Union inhabitants aged 15–34 years used cannabis in the past year. US SAMHSA (2021) data estimated 1.5 million aged 12 -17 years reported use of marijuana. CCDP use among children and adolescents is associated with adverse events (AE). Among WHO Vigibase and FAERs national reporting databases, 8% of those aged 2 to 17 years reported an AE after exposure to a synthetic CBD or cannabis product. As the prevalence and diversity of cannabinoid-containing products rises globally, the task of gathering useful Real-World Data (RWD) becomes increasingly complex. Health Care Professionals (HCPs) are often hesitant to record CCDP use data in patient files in countries and states where there are perceived legal challenges associated with documenting its use. The combination of a dearth of systematic education and documentation of use hinders a comprehensive understanding of the benefits, risks, and potential drug-drug interactions (DDIs) associated with these products. Addressing this gap is crucial for informed healthcare decisions. There remains a significant lack of evidence and data collection for CCDPs overall and within the pediatric rheumatic disease community.

**Objectives:** To improve HCPs knowledge, attitudes (K/A), and data collection practices related to CCDP (includes hemp and hemp-derived products).

**Methods:** We conducted a preliminary survey with HCPs, evaluating K/A of CCDPs. K/A survey is 40 questions in length. Building on the insights gained, we developed a pilot study that includes an updated K/A survey with an educational platform, known as Cerium. The study utilized qualitative methods to gather feedback on the content and delivery of the educational material. We designed four distinct modules for the pilot educational program. A systematic data collection tool, Budsinfo.com, was integrated into the program to report and analyze real-time outcomes related to CCDP use from the participating HCPs and their patients.

**Results:** At the time of submission our survey and educational intervention is ongoing. To date, 63 % support the use of an anonymous experience (i.e., AE) reporting tool. Only 16 % of respondents report being confident in their current knowledge of cannabinoid molecules. Data collection continues and is expected to be complete by August 2024. Preliminary data and analysis will be shared regarding the pre- and post-completed survey results, along with novel AE data. Currently, within the AE tool (Budsinfo.com), 5% are < 18 years.

**Conclusion:** A holistic approach of providing education and data collection tools contributes to enhanced product safety understanding in rapidly evolving markets, fostering a more informed and responsive public health ecosystem. We believe this is the first attempt at providing an intervention that addresses both CCDP data collection and education for the HCP.

**Patient Consent**

Not applicable (there are no patient data)

**Disclosure of Interest**

J. Marcu Consultant with: < 5,000 USD, P. Molloy: None Declared, K. Pray Consultant with: < 1,000 USD, J. Simon Consultant with: < 5,000.00 USD, S. Heaning Consultant with: < 1,000 USD, M. Wabwire Consultant with: < 1,000 USD, T. Simon Shareholder with: Owns Physicians Research Center

### e-health and digital health applications

#### P141 School day and weekend patterns of physical activity in juvenile idiopathic arthritis comprising with healthy peers

##### Yusuf Acikgoz^1^, Nilay Arman^2^, Asena Yekdaneh^3^, Asya Albayrak^4^, Irem Donmez^1^, Nuray Aktay Ayaz^5^

###### ^1^Institute of Graduate Studies, Physiotherapy and Rehabilitation Master Program; ^2^Faculty of Health Sciences, Department of Physiotherapy and Rehabilitation, Istanbul University-Cerrahpasa; ^3^Vocational School of Health Services, Physiotherapy English Program, Istanbul, Turkey, Fenerbahçe University; ^4^Institute of Graduate Studies, Physiotherapy and Rehabilitation Doctorate Program, Istanbul University-Cerrahpasa; ^5^Istanbul Faculty of Medicine, Department of Pediatrics, Department of Pediatric Rheumatology, Istanbul University, Istanbul, Türkiye

####### **Correspondence:** Yusuf Acikgoz

*Pediatric Rheumatology 2024*, **22(2):**PReS24-ABS-1600

**Introduction:** Insufficient physical activity in children and adolescents is a global public health issue (1). A study conducted with healthy adolescents in Turkey reported that 75% of adolescents were physically inactive (2). The daily step count goal for individuals aged 6-19 years is recommended to be 12,000 steps (3). School-aged children are more active on school days compared to weekends (4).

**Objectives:** The aim of the study was to assess the physical activity patterns of Juvenile Idiopathic Arthritis (JIA) patients on school days and weekends, comparing them to healthy peers. We specifically look to identify differences in physical activity levels between weekdays and weekends and to determine whether both groups meet the recommended step count.

**Methods:** Nineteen JIA patients aged 12-18 years and 17 healthy adolescents using Android-based mobile phones participated in the study. Participants received smartwatches from four brands (Samsung, Huawei, Mi, Tetfit) and were trained in their use and data management. The Pedi@ctivity mobile app, developed by our team, recorded daily step counts from these watches, which were synced with the Google Fit app on the Pedi@ctivity web platform for remote monitoring. Step counts during the winter school term were logged and analyzed remotely. Statistical analysis was conducted using SPSS Version 24.0.

**Results:** The study found no significant differences in school day physical activity levels between JIA patients and healthy peers (p > 0.05). However, JIA patients displayed significantly higher step counts on weekends compared to their healthy peers (p < 0.05). Nonetheless, both groups fell significantly short of the recommended step count.

**Conclusion:** The results suggest that JIA patients are more active on weekends and engage in more physical activity compared to healthy peers, likely due to their involvement in specific exercise programs. Despite this, both groups did not meet the recommended step counts, possibly due to the study being conducted in winter. This highlights the need to support and encourage physical activity in both groups.

This study was supported within the scope of TUBITAK 1001-Scientific and Technological Research Projects Support Program 121E690.

**Date of birth:** octobre 07

**Patient Consent**

Yes, I received consent

**Disclosure of Interest**

None Declare

**References**

1. Asma MB, Andan H, Kurt ME, Ceylan A. (2022). Adölesan Öğrencilerde Fiziksel Aktivite ve Obezitenin Adölesan Yaşam Biçimi, Pedometre ve BIA ile Değerlendirilmesi. Gaziantep Üniversitesi Spor Bilimleri Dergisi, 7(3), 188-202.
2. Aksoydan E, ÇAKIR N, Adölesanların beslenme alışkanlıkları, fiziksel aktivite düzeyleri ve vücut kitle indekslerinin değerlendirilmesi, 264 – 270, (2011).
3. Colley, R. C., Janssen, I. A. N., & Tremblay, M. S. (2012). Daily step target to measure adherence to physical activity guidelines in children. Med Sci Sports Exerc, 44(5), 977-982.
4. Brooke HL, Corder K, Atkin AJ, van Sluijs EM. A systematic literature review with meta-analyses of within- and between-day differences in objectively measured physical activity in school-aged children. Sports Med. 2014 Oct;44(10):1427-38.

### e-health and digital health applications

#### P142 Knowledge, experience and attitudes of pediatric rheumatologists regarding virtual reality applications: a questionnaire-based study

##### Ayşenur Doğru^1^, Selen Duygu Arık^1^, Özlem Akgün^1^, Fatmagül Demirkan^2^, Bengisu Menentoğlu^1^, Büşra Başer Taşkın^1^, Nuray Aktay Ayaz^1^

###### ^1^Pediatric Rheumatology, Istanbul University Faculty of Medicine; ^2^Pediatric Rheumatology, Kanuni Sultan Süleyman Research and Training Hospital, Istanbul, Türkiye

####### **Correspondence:** Ayşenur Doğru

*Pediatric Rheumatology 2024*, **22(2):**PReS24-ABS-1808

**Introduction:** In Virtual Reality (VR) technology, users experience real-world sensations within a virtual environment, generated by specialized equipment. The primary component is a head-mounted display, which interfaces with a mobile phone or computer. In addition hand controllers, sensory detectors, gloves, and clothing are utilized to enhance the reality. This creates a three-dimensional, multi-sensory and immersive environment. VR technology seems to have a major role in education and multidimensional management of healthcare. It could emerge as a game changer in medical education and practical training, clinical management, patient education, exercise, and scientific research.

**Objectives:** We aimed to evaluate the knowledge, attitudes, usage tendencies and experiences of paediatric rheumatologists regarding VR technology in clinical practice.

**Methods:** This study was conducted among pediatric rheumatologists (PR) in Turkiye. Eigthy-one physicians completed an anonymous questionnaire sent via e-mail or telephone. The electronic questionnaire consisted of 27 questions divided into three sections: the first section included demographic characteristics of the participants (age, gender, academic title, affiliated center, pediatric rheumatology experience), the second section included participants’ knowledge about social media and telemedicine, and the third section included participants’ knowledge, attitudes and behaviors about virtual reality. Participants were divided into two groups based on their familiarity with VR technology: Group 1 consisted of those who claimed to have knowledge about VR technology, and Group 2 included individuals who reported that they had no knowledge of VR technology.

**Results:** The survey was distributed to 81 clinicians working in the field of PR in Turkey. Within the cohort, 55 individuals (67.9%) possessed knowledge of VR (group 1), while 26 individuals (32.1%) lacked familiarity with VR (group 2). In group 1, 47 participants (85.5%) were women, while in group 2, 20 participants (76.9%) were women. Statistical analysis revealed no significant difference in gender distribution (p=0.343). The median (min-max) ages of group 1 and group 2 were similar (38 (31-64), 36.5 (31-56), p:0.206, respectively). In group 1; 23 (41.8%) were fellows, 15 (27.3%) were speciality doctors, 17 (30.9%) were faculty members while 17 (%65.4) were fellows, 4 (%15.4) were speciality doctors, 5 (19.2) were faculty members in group 2 (p:0.164). Five (%9.1) participants worked in a governmental health facility, 50 (%90.9) participants worked in a tertiary hospital in Group 1. Two (%7.7) participants worked in a governmental health facility, 23 (%88.5) participants worked in a tertiary hospital, and 1 (%3.8) participants worked in a private hospital in Group 2 (p:0.461). In Group 1, 34 (61.8%) participants reported having 1-5 years of experience, 13 (23.6%) had 6-10 years, 4 (7.3%) had 16-20 years, and 4 (7.3%) had >20 years of experience. In Group 2, 19 participants (73.1%) had 1-5 years of experience, 4 (15.4%) had 6-10 years, 1 (3.8%) had 11-15 years, and 2 (7.7%) had 16-20 years of work experience (p=0.333). Group 1 had significantly more telemedicine knowledge and health technology knowledge (p:0.003 and p<0.001, respectively).Group 1 is more experienced in virtual reality and more familiar with virtual reality equipment (p<0.001 both).

**Conclusion:** Since technology has advanced over the past two decades, VR applications have become widespread in the field of medicine and rheumatology. The fact that those who have knowledge about VR in our study also have knowledge about VR usage areas and equipment is promising that it will be used more in the field of rheumatology in our country in the coming years.

**Date of birth::** octobre 20

**Patient Consent**

Not applicable (there are no patient data)

**Disclosure of Interest**

None Declared

### e-health and digital health applications

#### P143 Implementing JAMAR questionnaire to patients with JIA

##### Anette Pieler, Heidi K. Ipsen

###### Hans Christian Andersen Children’s Hospital, Odense, Denmark

####### **Correspondence:** Anette Pieler

*Pediatric Rheumatology 2024*, **22(2):**PReS24-ABS-1675

**Introduction:** In January 2023, a Danish National JIA database was ready for use. One part of the database is a JAMAR (Juvenile Arthritis Multidimensional Assessment Report) questionnaire that the patients (and their families) have to answer before each consultation.

**Objectives:** To introduce the questionnaire to the patients in a user-friendly way to ensure high participation for all consultations and to make it possible to answer without involvement of the health professionals.

**Methods**

1. We started by randomly selecting patients to try out the questionnaire to ensure that it worked and made sense for the patients.
2. When confident that the questionnaire was usable we began informing patients at their consultations of the questionnaire and how the data would be used. They received oral and written information and asked for most relevant phone number for login to questionnaire.
3. The patients received information about a regional health app, and was asked to join. In the app they could access the questionnaire, contact the nurses and see their appointments. The app also sends a reminder of both appointment and questionnaire a week prior.
4. To further the process we created a special appointment card with a QR code to the app and directions of use.
5. With the use of the app, it was no longer necessary to have an open phone line to the nurses. Instead, we asked patients to write to the nurses via app, thereby creating a very important reason to join the app.

**Results:** The implementation process started in January 2023 and in May 2024, it is still ongoing. It has been necessary to adjust the process several times, as it was clear that implementing a new procedure was very difficult and that many patients found it difficult to adapt to something new. New patients found no problem with either app or questionnaire.

There was also frequently technical issues with either app or questionnaire. The database created a support contact that made it easy for the health professionals to report all difficulties. Patients were asked to contact the team if they had technical difficulties but this is not something that they do.

**Conclusion:** Implementing a new routine is difficult. It quickly became obvious that patients and, especially parents found it difficult to adjust to something new. For most patients it will take about 3 or 4 consultations where they are reminded to get the app and answer the questionnaire, before they start doing it unprompted.

Planning the process and troubleshooting as much a possible could make it easier. It is much recommended to have a support setup for when the technology is not working, as it should. It became an extra inconvenience for the patients when the app and questionnaire did not work correctly, and was not motivating for the patients.

The biggest motivation for getting the app, and thereby getting reminded of the questionnaire was the closing of the nurses phone. The phone was a point of frustration for many as it could be hard to get in contact with nurses. Patients, in general, liked that they could write whenever they wanted.

**Patient Consent**

Not applicable (there are no patient data)

**Disclosure of Interest**

None Declare

### e-health and digital health applications

#### P144 B-ACTIVE: a platform for children with rheumatic diseases

##### Laura Gago^1,2^, Mariana L. Emília^1,3^, Maria Helena Lourenço^1,3^, Paula Maciel^4^, Ana Pinto^4^, Mariana Matos^5^, Carlos Luz^6^, Francisco António^7^, Pedro Sousa^7^, Manuela Costa^1,2^, Ana Rodrigues^8,9,10^, Helena Canhão^9,10,11^, Ana Filipa Mourão^1,2^

###### ^1^Rheumatology, Unidade Local de Saúde Lisboa Ocidental; ^2^Comprehensive Health Research Centre, Nova Medical School, Lisbon, Portugal; ^3^Rheumatology, Comprehensive Health Research Centre, Nova Medical School; ^4^Pediatrics; ^5^Clinical Nutricional, Unidade Local de Saúde Lisboa Ocidental; ^6^Escola Superior de Educação de Lisboa/Centro Interdisciplinar de Estudo da Performance Humana; ^7^Department of Electrical Engineering of the Faculty of Science and Technology; ^8^Rheumatology, Hospital da Luz; ^9^Comprehensive Health Research Centre, Nova Medical School; ^10^EpiDOC Unit, NOVA Medical School; ^11^Rheumatology, Unidade Local de Saúde de São José, Lisbon, Portugal

####### **Correspondence:** Laura Gago

*Pediatric Rheumatology 2024*, **22(2):**PReS24-ABS-1252

**Introduction:** Young people often struggle to self-manage chronic diseases during the transition from childhood to adulthood.Doctors do their best to treat diseases, but also have a central role in promoting healthy lifestyles since childhood.We believe that we must put in practice interventions to make children “sit less and move more, sleep well and eat better”,not only to improve disease symptoms but also to prevent future damage.

**Objectives:** We developed a virtual platform,where patients with juvenile rheumatic diseases completed questionnaires about disease activity,nutritional,physical activity habits and mental health.Before using the platform, they had a first evaluation performed by a multidisciplinary team(composed by rheumatologist,psychologist,personal trainer and nutritionist).After this evaluation,they had to fill out the platform on a bi-weekly basis and could access an exercise plan according to their disease involvement, a nutritional plan adjusted to individual preferences and needs.Mindfulness programs and stress management strategies are given, according to the answers.

**Results:** We started recruiting patients in June 2022.Thirty-two patients were included(21 females and 11 males).Patients were diagnosed with oligoarticular juvenile idiopathic arthritis (JIA)(n=8),polyarticular JIA(n=5),psoriatic JIA(n=4),enthesitis-related arthritis(n=3),systemic lupus erythematosus(SLE)(n=4),primary Raynaud’s phenomenon(n=2), juvenile fibromyalgia(n=2),Marfan syndrome(n=1),Takayasu arteritis(n=1), juvenile osteoporosis(n=1) and one case of periodic fever, aphthous stomatitis, pharyngitis, adenitis(PFAPA, n=1).On the first use of the application, most patients(n=20) had a VAS of 0, 5 patients had a VAS between 20 and 40, 6 patients had a VAS between 40 and 60, and 3 patients had a VAS above 60.The 3 patients that had a VAS superior to 60 were diagnosed with juvenile fibromyalgia and Marfan syndrome. Median Body Mass Index(BMI) was 22 (normal BMI).Considering eating habits,25% of the included patients did not eat fruit or vegetables daily, and half of the patients did not eat fish regularly. We highlight that five patients reported to eat candys daily.In the psychological evaluation, we found that the majority (94%) of our sample felt happy and fulfilled, but a small percentage of patients (6%) reported self-hate and frequent sadness. Regarding physical activity, half of the patients didn’t exercise at all, including during physical education classes.Among those, VAS ranged from 0 to 30.

**Conclusion:** Despite the small amount of data, since our results depend on the patients engagement in the project, it is important to reinforce the need of a multidisciplinary approach, aiming the creation of tools to improve the lifestyle of the children suffering from rheumatic diseases. In the future, we hope to recruit more children by engaging more hospitals in participating in this innovative project.

**Date of birth:** mars 30, Y

**Patient Consent**

Not applicable (there are no patient data)

**Disclosure of Interest**

None Declared

### e-health and digital health applications

#### P145 Investigation of body composition by bioelectrical impedance analysis in adolescents with juvenile idiopathic arthritis and familial mediterranean fever: healthy control comparison

##### Nilay Arman^2^, Özlem Akgün^1^, Asya Albayrak^3^, Asena Yekdaneh^3^, Kübra Uçar^4^, Nuray Aktay Ayaz^1^

###### ^1^Division of Pediatric Rheumatology, Istanbul University Faculty of Medicine; ^2^Department of Physiotherapy and Rehabilitation, Istanbul University-Cerrahpaşa Faculty of Health Sciences; ^3^Cerrahpaşa Institute of Graduate Studies Physiotherapy and Rehabilitation Doctorate Program, Istanbul University; ^4^Department of Physiotherapy and Rehabilitation, Istanbul University-Cerrahpaşa Faculty of Health Sciences, Istanbul, Türkiye

####### **Correspondence:** Özlem Akgün

*Pediatric Rheumatology 2024*, **22(2):**PReS24-ABS-1810

**Introduction:** Body composition status in adolescence provides predictivity about the status in adulthood. Therefore, it is important to monitor the changes in total body water, fat mass and lean body mass (1). Bioelectrical Impedance Analysis (BIA) is frequently used for the evaluation of body composition because of its ease of use (2).

**Objectives:** The aim of this study was to evaluate body composition status using BIA in adolescents diagnosed with Juvenile Idiopathic Arthritis (JIA) and Familial Mediterranean Fever (FMF), and compare them with their healthy peers.

**Methods:** The study included 46 JIA patients, 17 FMF patients, and 122 healthy controls of similar age and gender. Body composition was assessed using a BIA device (Tanita SC240MA). Various measurements were taken, and a comparison was made between the patients and control groups.

**Results:** In JIA patients, girls constituted 53.2% of cohort, with a mean age of 13,86 ± 1,47 years, in FMF patients girls constituted 35.2% of cohort, with a mean age of 13,94 ± 1,88 years. Patients diagnosed with JIA were found to have lower body weight (p=0,041), shorter height (p=0,036), higher total body mineral % (p=0,017), lower fluid (p=0,006) and lean mass (p=0,008) compared to their healthy peers. Patients with FMF had higher BMI (p=0,027), fat mass (p=0,003) and fat % (p=0,031) compared to their healthy peers. When patients with JIA and FMF were compared, it was found that patients with JIA had lower body weight (p=0,001), height (p=0,036), fluid (p=0,005) and fat-free mass (p=0,005), while patients with FMF had higher fat mass (p=0,005).

**Conclusion:** Our findings showed that JIA patients had shorter height, lower body weight, and fluid and fat-free mass compared to both healthy controls and FMF patients. Understanding how the metabolic and inflammatory effects of the diseases affect body composition in adolescents with JIA and FMF using the BIA method may play an important role in disease management, development of treatment strategies, and establishment of exercise programs.

This study was supported by TÜBİTAK 1001-Scientific and Technological Research Projects Support Program, project number 121E690.

**Patient Consent**

Not applicable (there are no patient data)

**Disclosure of Interest**

None Declared

**References**

1. Van Eyck, A., Eerens, S., Trouet, D., Lauwers, E., Wouters, K., De Winter, B. Y.,.. Ledeganck, K. J. (2021). Body composition monitoring in children and adolescents: reproducibility and reference values. European Journal of Pediatrics, 180, 1721-1732.
2. Brantlov, S., Ward, L. C., Jødal, L., Rittig, S., & Lange, A. (2017). Critical factors and their impact on bioelectrical impedance analysis in children: a review. Journal of Medical Engineering & Technology, 41(1), 22-35.

### Genetics, genomics and environment

#### P146 Preliminary results of the molecular genetic testing on the autoinflammatory disorders gene panel in patients with suspected monogenic autoinflammatory diseases

##### Mariia Beketova^1^, Svetlana Salugina^2^, Evgeny Fedorov^2^, Ekaterina Zakharova^3^

###### ^1^Lomonosov Moscow State University; ^2^V.A. Nasonova Research Institute of Rheumatology; ^3^Research Center of Medical Genetics, Moscow, Russian Federation

####### **Correspondence:** Mariia Beketova

*Pediatric Rheumatology 2024*, **22(2):**PReS24-ABS-1179

**Introduction:** The development of molecular genetic testing and using of the “genotype-first” approach made it possible to discover new monogenic autoinflammatory diseases (mAIDs) that require further research.

**Objectives:** To study the frequency and spectrum of genetic variants in molecular genetic testing on the autoinflammatory disorders gene panel and clinical features in patients (pts) with suspected mAIDs.

**Methods:** From Feb 2021 to Jan 2024 the study included 127 pts with suspected mAIDs, who were examined in V.A. Nasonova Research Institute of Rheumatology and underwent genetic analysis using massively parallel sequencing in Research Centre for Medical Genetics. In most pts sequencing was performed through a 65-gene panel, but in some cases with 16-, 83-, 458- and 514-gene panel. Median age 9.5 [0.7–54] yrs; M:F=1:1,1. Inclusion criteria were: presence of systemic inflammation signs; absence of response to previous therapy. Further, pts with negative result on 16-gene panel was excluded from analysis clinical and laboratory features.

**Results:** Depending on the results of the genetic testing, pts were divided into 2 groups: 45 pts (35%) with genetic mutations and 82 negative cases. There were identified 37 monogenic mutations (12 in gene MEFV, 4 - NLRP3 и NOD2, 3 - TNFRSF1A, 2 - MVK, RAG1, NLRP1, ADA2, and 1 - IFIH1, NLRP12, UNC13D, RIPK1, IL36RN) and 8 digenic/oligogenic mutations (2 - MEFV/TNFRSF1A, 1 - MEFV/MVK/TNFAIP3, MEFV/NOD2/PSTPIP1, NOD2/MEFV, MEFV/NLRP12, IL36RN/STX11, SH2D1A/ TNFAIP3). Pts with genetic mutations (group 1) and 72 pts with negative result on 65- 83-, 458- and 514-gene panel (group 2) were compared. The age at the onset in the groups was not statistically significant difference (Me 5(0-34) and 5(0-44) yrs). Fever was observed both in 89%, intermittent fever in 73% and 71% in group 1 and 2, respectively. The most frequent symptoms were skin lesions (52% and 49%), arthralgia (61% and 46%), arthritis (34% and 25%), gastrointestinal symptoms (34% and 36%) and lymphadenopathy (27% and 33%). Chest pain was more common in group 1 (16% and 1%, p=0.009). The other clinical differences between the groups were not significant. Laboratory inflammatory activity was present in 81% and 80%. Median CRP was 40 (0.1–262.4) and 38.5 (0.1–273.1) mg/L, p=0.887. Anemia was detected in 39% and 38%, hyperproduction of autoantibodies (ANA, aDNA, ACL, RF) in 19% and 33% (p=0.240).

**Conclusion:** Thus, molecular genetic testing was positive in a third of pts with suspected monogenic AIDs. Among them digenic/oligogenic combinations of genetic variants were noted in 18%, which requires further interpretation. The differences between clinical picture in groups with and without gene mutations were not significant, with the exception of a higher frequency of chest pain in monogenic AIDs. Cases with negative genetic testing result require whole exome/genome sequencing. It is necessary further clarification of the criteria for prescribing molecular genetic testing to confirm mAIDs.

**Date of birth:** juin 12, Y

**Patient Consent**

Yes, I received consent

**Disclosure of Interest**

None Declared

**Reference**

1. Bousfiha A, Moundir A, Tangye SG, Picard C, Jeddane L, Al-Herz W, et al. The 2022 Update of IUIS Phenotypical Classification for Human Inborn Errors of Immunity. J Clin Immunol. 2022;42(7):1508-1520. 10.1007/s10875-022-01352-z.

### Genetics, genomics and environment

#### P147 Association of alleles of the HLA-A, HLA-B, HLA-C, HLA-DRB1, HLA-DQB1 genes with susceptibility to juvenile-onset sle in Russian population

##### Irina Guseva^1^, Mikhail Chanyshev^2^, Maria Kaleda^1^, Natalia Vlasenko^2^, Irina Nikishina^1^, Elena Samarkina^1^, Maria Shabatina^1^, Kamil Khafizov^2^

###### ^1^V.A. Nasonova Research Institute of Rheumatology; ^2^Central Research Institute of Epidemiology, Moscow, Russian Federation

####### **Correspondence:** Irina Nikishina

*Pediatric Rheumatology 2024*, **22(2):**PReS24-ABS-1219

**Introduction:** Systemic lupus erythematosus (SLE) is a multifactorial chronic autoimmune disease with strong genetic components. Susceptibility to developing SLE has been strongly associated with HLA alleles in different ethnic and populations groups. These studies are also importance in juvenile-onset SLE (jSLE).

**Objectives:** To investigate the association of alleles of the *HLA-A, HLA-B, HLA-C, HLA-DRB1, HLA-DQB1* genes with susceptibility to jSLE in Russian population.

**Methods:** A total of 82 patients (pts) with jSLE (66 females) with an average age of 11.9±3.2 years at onset and 160 sex- and age-matched healthy individuals (controls) without autoimmune diseases or family history of them were included in this case-control study. Diagnosis of SLE was reviewed based on 2012 SLICC criteria. 76.6% of pts had acute cutaneous lupus at the onset, 42.2% - nonscarring alopecia, 76.0% - arthritis, 22.0% - oral and nasal ulcers, 20.3% - serositis, 10.9% - osteonecrosis, 40.6% - renal involvement, 25.0%–neuropsychiatric disorders. Leucopenia was found in 68.0% of pts, thrombocytopenia – in 28.0%. ANA were detected in 100% pts, anti-dsDNA – in 90.0%, anti-Sm – in 26.5%, antiphospholipid antibodies - in 20.0%, hypocomplementemia – in 59.2%, positive direct Coombs test – in 30.6 %. A custom panel of primers containing Illumina adapter tails was used for amplification of *HLA-A/B/C/DRB1/DQB1* exones. High-throughput sequencing was performed on the Illumina MiSeq platform with MiSeq Reagent Kit v3 (600 cycles). *HLA-A/B/C/DRB1/DQB1* alleles were determined using SpecHLA. Statistical data processing, including comparison of the frequencies of HLA alleles in the group of pts with jSLE and in the control group, was carried out in the Python software environment using the Numpy, Pandas, and scikit-learn libraries. The risk score was calculated as an odds ratio with 95% confidence intervals (OR [95%CI]).

**Results:** jSLE is positively associated with high statistical significance with *HLA* class I (A*01:01:01 (OR=3.28 [1.74-6.18], p=0.000), B*08:01:01 (OR=4.28 [2.01-9.11], p=0.000*,*) and class II (DRB1*03:01:01 (OR=3.74 [2.01-6.96], p=0.000), DRB1*15:01:01 (2.96 [1.70-5.14], p= 0.000), DQB1*02:01:01 (OR=4.23 [2.23-8.05], р=0.000) alleles. At the same time the *07:01:01 allele reduces the susceptibility to developing jSLE (OR=0.36 [0.19- 0.69], p= 0.002).

**Conclusion:** Our preliminary study in a group of jSLE pts revealed that HLA class I (A*01:01:01, B*08:01:01,) and class II (DRB1*03:01:01, DRB1*15:01:01, DQB1*02:01:01) alleles are associated with susceptibility to jSLE, although allele DRB1*07:01:01 has a protective effect.

**Patient Consent**

Yes, I received consent

**Disclosure of Interest**

None Declared

### Genetics, genomics and environment

#### P148 SLCO1B1 polymorphisms influence methotrexate adverse events in juvenile idiopathic arthritis

##### Jimena Garcia-Silva^1^, Beatriz Silva-Ramirez^2^, Ana V. Villarreal- Treviño^1^, Viviana Mata-Tijerina^2^, Nadina E. Rubio-Perez^1^, Fernando García-Rodríguez^1^ and Colaborativa de Investigación en Beneficio de la Reumatología Infantil (COLIBRI)

###### ^1^Pediatrics, Universidad Autónoma de Nuevo León; ^2^Departamento de Inmunogenetica, Centro de Investigación Biomédica del Noreste, Instituto Mexicano del Seguro Social, Monterrey, Mexico

####### **Correspondence:** Fernando García-Rodríguez

*Pediatric Rheumatology 2024*, **22(2):**PReS24-ABS-1353

**Introduction:** Methotrexate (MTX) is the most used disease-modifying antirheumatic drug (DMARD) for juvenile idiopathic arthritis (JIA), inducing disease remission in over 70% of the patients. The most frequent adverse events (AEs) of MTX are gastrointestinal alterations and hepatotoxicity, although these AEs have been well-described, we have not been able to predict whether a patient will develop them. There have been evidence associating genetic variants of the SLOC1B1 gene, codes for a liver protein (OATP1B1) that is responsible for drug transportation, with altered pharmacokinetic profiles resulting in delayed MTX clearance and increased toxicity.

**Objectives:** To determine the association between SNPs in the SLCO1B1 gene (rs4149056, rs2306283) with the presence of AEs in patients with JIA being treated with MTX.

**Methods:** We conducted a retrospective cohort study including pediatric patients with JIA, who had received MTX for at least 3 months. The presence of AEs were evaluated during the patient visits, these were manually collected from the medical records and were only included when the clinician directly attributed the symptom to MTX. Single nucleotide polymorphism genotyping was performed using genomic DNA isolated from peripheral blood samples.

**Results:** Thirty Mexican children diagnosed with JIA participated in the study, 22 girls and 8 boys, with a mean age of 11 (8.3-156) years. Twenty patients (66.7%) presented any AEs, being gastrointestinal alterations the most frequent (19, 95%). The distribution of genotypes was found in Hardy-Weinberg equilibrium. The SLCO1B1*1B was associated with the presence of more AEs in the response to MTX in patients with JIA (OR=3.89, 95% CI=1.23 -12.29, p=0.03).

**Conclusion:** The *1B haplotype was the most frequent one in our patients, 16 patients (53.3%) presented this haplotype in either homozygous or heterozygous form. This variant was significantly associated with a higher odds ratio of MTX AEs. SLCO1B1 genotyping is a promising way to identify patients at higher risk of getting AEs during treatment with MTX, although further studies are needed to verify our findings.

**Date of birth:** mars 27, Y

**Patient Consent**

Not applicable (there are no patient data)

**Disclosure of Interest**

None Declared

### Genetics, genomics and environment

#### P149 Hyperuricemia with C.421C>A ABCG2 gene variant in a male patient with von willenbrand disease responding to febuxostat

##### Katerina Bouchalova^1^, Zuzana Pytelova^1^, Dagmar Pospisilova^1^, Blanka Stiburkova^2^

###### ^1^Dpt. of Pediatrics, Palacky University, Olomouc; ^2^Institute of Rheumatology, Prague, Czech Republic

####### **Correspondence:** Katerina Bouchalova

*Pediatric Rheumatology 2024*, **22(2):**PReS24-ABS-1772

**Introduction:** Gouty arthropathy is a rare disease in children. The etiology is hyperuricemia, which might be familial. Major clinical manifestations include acute monoarthritis, associated with tophi and nephrolithiasis. There are no validated recommendations for treatment in childhood. The treatment is tailored to a particular patient, starting with NSAID therapy with the addition of allopurinol. Rarely biologic therapy (IL-1 blockade canakinumab) is required. *ABCG2* dysfunction is a strong independent risk for pediatric-onset hyperuricemia, and genetic data in those pediatric patients can contribute to more accurate disease prognoses, help personalized lifestyle advice, and improve therapy (urate-lowering therapy choice).

**Objectives:** A case report of a patient with von Willebrand disease and symptomatic hyperuricemia, effectively treated using febuxostat.

**Methods:** A patient was treated and followed by Pediatric Rheumatology at Univesity Hospital in Olomouc. A genetic analysis was performed at Institute of Rheumatology, Prague.

**Results:** A 14-year-old male patient with a positive family history of gout was sent in 2018 year by a hematologist due to hyperuricemia, and foot pain, predominantly in heels. No arthritis was present. Von Willebrand disease, type II was diagnosed at the age of 2 years. First, the pain resolved after substitution therapy for the primary hematology disease. However, he stopped regular substitution therapy. Borderline hyperuricemia (415 mmol/l) was first detected at the age of 12 years and 8 months, and he was treated by a general practitioner using allopurinol since February 2017 with no effect. Hyperuricemia (up to 506 mmol/l) was proved by our laboratory, while CRP, ESR, blood count, and liver and kidney tests were normal. A low level of 25-OH vitamin D was detected (39.7 nmol/l). Allopurinol did not affect on both clinical symptoms and uric acid levels. Vitamin D and diet were recommended, too. Due to positive family history, we asked for the genetic analysis, which revealed homozygous variant rs72552713 in *ABCG2* gene variant (c.421C>A, p.Q141K) in proband. Later, with knowledge of the gene variant, we started febuxostat therapy with an effect on both symptoms and uric acid levels. The patient underwent a transition to adult rheumatology. His status at the time of abstract submission is good.

**Conclusion:** This is the first published child patient with c.421C>A *ABCG2* gene variant in uric acid transporter and von Willebrand disease. This common dysfunctional variant, p.Q141K, results in a 53% reduction in urate transport and has been reported to be a major genetic cause of hyperuricemia/gout in the European population.

This research was supported by the Czech Ministry of Health (DRO FNOl, 00098892).

**Patient Consent**

Yes, I received consent

**Disclosure of Interest**

None Declared

**References**

1. Foster HE *et* Rabinowich CE: Musculosceletal manifestation of systemic diseases. In Petty RE, et al. Textbook of Pediatric Rheumatology 2021.
2. Stiburkova B, *et* al. The impact of dysfunctional variants of ABCG2 on hyperuricemia and gout in pediatric-onset patients. Arthritis Res Ther 2019.

### Genetics, genomics and environment

#### P150 Fecal microbiome in new-onset juvenile idiopathic arthritis – results from the pilot study

##### Justyna Roszkiewicz^1^, Jakub Lach^2,3^, Monika Baranowska^2^, Dominik Strapagiel^2,3^, Elzbieta Smolewska^1^

###### ^1^Department of Pediatric Cardiology and Rheumatology, Medical University of Lodz; ^2^Biobank Lab, Faculty of Biology and Environmental Protection, University of Lodz, Lodz; ^3^BBMRI.pl Consortium, Wroclaw, Poland

####### **Correspondence:** Elżbieta Smolewska

*Pediatric Rheumatology 2024*, **22(2):**PReS24-ABS-1206

**Introduction:** Rheumatic diseases result from the interplay between genetic and environmental factors. There is growing evidence that intestinal dysbiosis may have an impact on gut permeability and in consequence lead to immunological imbalance and autoinflammation. Gut dysbiosis is also gaining interest as an import ant factor in the pathogenesis of Juvenile Idiopathic Arthritis (JIA), but has not been studied in Polish population yet.

**Objectives:** The aim of our study was to compare the composition of intestinal microbiome and concentration of gut permeability marker – zonulin, in children with JIA and In healthy controls.

**Methods:** This was a pilot, single centre, prospective cohort study. Fecal and full blood samples were collected from 15 treatment-naive JIA patients and 15 sex-and-age-matched healthy controls. Gut microbiome composition was determined by 16S ribosomal RNA-based metagenomics. Alpha, β-diversity and ratios of relative abundance were compared between patients and the control group. Zonulin concentration was determined using ELISA method.

**Results:** We found no difference in microbiom biodiversity and relative species abundance between JIA children and healthy controls. We also report no statistically significant difference between the concentration of serum zonulin between these two groups.

**Conclusion:** Our results suggest that gut dysbiosis may not be the leading mechanism of immunological imbalance in Polish JIA patients. Due to relatively small study group size our results should be interpreted with caution and future research on larger groups of JIA patients is encouraged to verify our findings.

**Patient Consent**

Not applicable (there are no patient data)

**Disclosure of Interest**

None Declared

### Genetics, genomics and environment

#### P151 Type I interferonopathies in pediatric rheumatology. Presentation of three clinical cases of Uruguay

##### Rodrigo Suárez^1^, Alfredo Cerisola^2^

###### ^1^Rheumatology; ^2^Neuropediatrics, CHPR, Montevideo, Uruguay

####### **Correspondence:** Rodrigo Suárez

*Pediatric Rheumatology 2024*, **22(2):**PReS24-ABS-1662

**Introduction:** Type I interferonopathies are a heterogeneous group of inherited autoinflammatory diseases characterized by an overproduction of type I interferons (IFN).

Aicardi-Goutières syndrome is a progressive encephalopathy, in which expressions of autoimmunity are common.

**Objectives:** Report and describe the cases of three patients with Type I interferonopathies who presented with rheumatic pathology in childhood.

**Methods:** Review of the medical records of three uruguayan patients with Aicardi-Goutières syndrome (AGS) under follow-up by Pediatric Rheumatology.

**Results:** CASE I: Male carrier of Aicardi-Goutières syndrome (AGS), type 7 due to mutation in IFIH1. At 11 years old, diagnosis of Systemic Lupus Erythematosus: fever, malar erythema, and erosive polyarthritis, antinuclear antibodies (ANA): 1/320, anti DNA, Smith positive, hypocomplementemia. Evolves without renal or neuropsychiatric involvement, prolonged requirement for low-dose predisone and clinical-serological dissociation. SLICC/ACR: 2.

CASE II: Sister of patient I, diagnosis of Aicardi-Goutières syndrome (AGS), type 7 due to mutation in IFIH1. At 8 years old, diagnosis of Systemic Lupus Erythematosus, SLE 2019 EULAR/ACR criteria: 17: fever, polyarthritis, livedo reticularis in the lower limbs, anemia, ANA and anti DNA (1000 IU), anti Smith positive, and hypocomplementemia.

Proteinuria-creatininuria ratio of 0.17. Renal biopsy: Class II lupus glomerulonephritis. Flair with thrombocytopenia with hemorrhagic syndrome of rapid resolution

Evolution with persistence of arthritis with requirements for prednisone at low doses.

CASE III: Male with Aicardi-Goutières syndrome type 6, with mutation in the ADAR1 gene. Debut at 8 years of age with Polyarticular Juvenile Idiopathic Arthritis. Elevated sedimentation rate, Rheumatoid Factor of 1300 IU/ml, ANA:1/160 and Anti citrulinated positives. Treatment with methotrexate 12.5 mg weekly orally, persisting with polyarthritis Intolerance to oral sulfazalacine. Adalimumab s/c with partial improvement, low activity with weekly Etanercpt s/c.

**Conclusion:** We present three patients carrying Aicardi-Goutières syndrome, who developed multisystem inflammatory symptoms, with multiple positive antibodies.

Two brothers, who share a genetic mutation, presented with monogenic Systemic Lupus Erythematosus, with frecuent flairs and corticosteroid dependence. The third patient also presented, in addition to the cortico-dependent polyarticular symptoms and polyarthritis, systemic inflammation and multiple antibodies.

Type I interferonopathies are associated with systemic autoimmune pathologies, in these cases, with high levels of systemic inflammation and difficult control with remissives. The possibility of using anti-JAK drugs in these patients opens a door of possibilities for a better evolution.

**Date of birth:** décembre 1

**Patient Consent**

Yes, I received consent

**Disclosure of Interest**

None Declared

**Reference**

1. Giovanni Battista Dell’Isola, Gianluca Dini, Clinical spectrum and currently available treatment of type I interferonopathy Aicardi–Goutières syndrome. World Journal of Pediatrics (2023) 19:635–643.

### Imaging

#### P152 Superb microvascular imaging versus conventional power doppler ultrasound in scoring knee and wrist synovitis in juvenile idiopathic arthritis

##### Faekah Gohar^1^, Daniel Windschall^1,2^

###### ^1^Pediatric Rheumatology, St.Josef-Stift Sendenhorst, Sendenhorst; ^2^Medical Faculty, University of Halle-Wittenberg, Halle (Saale), Germany

####### **Correspondence:** Daniel Windschall

*Pediatric Rheumatology 2024*, **22(2):**PReS24-ABS-1317

**Introduction:** Superb Microvascular Imaging (SMI) is a novel ultrasound (US) Power Doppler technology (PD) that is more sensitive for low microvascular blood flow compared to conventional PD (CPD). Limited studies compare SMI with CPD in Juvenile idiopathic arthritis (JIA).

**Objectives:** To investigate the sensitivity of SMI vs CPD in the US-evaluation of synovial inflammation in patients with JIA.

**Methods:** JIA patients with clinically determined knee or wrist synovitis were investigated using B-Mode (BM), SMI and CPD. US settings were standardised. For knees, six standard views were analysed per knee joint (suprapatellar longitudinal and transverse, parapatellar medial and lateral and longitudinal medial and lateral) and for two for wrists (transverse view for the radioulnar joint and midline longitudinal view for radiocarpal, midcarpal and carpometacarpal joints). A semi-quantitative grading scale (OMERACT Score) ranging from 0 (no intrasynovial blood flow signals) to 3 (blood flow signals in >50% of the synovium) was used to grade the SMI and CPD signals in each individual image. BM was also graded 0-3, depending on the extent of synovial effusion or hypertrophy. The highest individual BM, CPD and SMI grade per single joint were taken for the calculation of the novel Musculoskeletal Ultrasound Sum Score (‘MUSS’, maximum score 6), calculated as the single highest BM grade + the single highest CPD or SMI grade for any single image for each individual joint.

**Results:** 16 knees (patient n=15) and 9 wrist joints (radioulnar x 4, radiocarpal x2, midcarpal x3, patient n=5) with clinical synovitis were analysed with US. In total 288 images for knees and 27 for wrists (all joints) were reviewed (individual images for BM, CPD and SMI were recorded per joint per individual view). The mean and standard deviation (sd) for BM grade was 2.6, 0.5 (knees) and 1.7, 0.5 (wrists). Mean (sd) CPD and SMI were 0.9 (0.7) and 2.3 (0.6) respectively in knees, p<0.001, and in wrists 0.9 (0.7) and 2.3 (0.8), p<0.001. Using the highest BM, CPD/SMI scores per individual joint, the mean MUSS using SMI+BM (mean 4.9, sd 0.8, range 3-6) was higher than when calculated using CPD (mean 3.6, sd 0.9, range 2-5), p<0.001. For wrists, mean MUSS using SMI rather than CPD was also higher with a mean 4.0 (sd 1.2, range 2-5) vs 2.6 (1.1, 1-4), p=0.02.

**Conclusion:** SMI was more sensitive than CPD for the detection of synovial hypervascularity in knees and wrists with synovitis. SMI could therefore represent a more precise imaging tool for diagnosing and monitoring intrasynovial hypervascularization, which could play a role in the treat-to-target management of JIA.

**Patient Consent**

Yes, I received consent

**Disclosure of Interest**

None Declared

### Imaging

#### P153 Spectrum of imaging assessement in patients with fibrodysplasia ossificans progressiva

##### Valeria Matkava^1^, Irina Nikishina^1^, Svetlana Arsenyeva^1^, Aliya Arefieva^1^, Leonid Blank^2^, Emil Gasymov^2^

###### ^1^Paediatric; ^2^Radiology, V.A. Nasonova Research Institute of Rheumatology, Moscow, Russian Federation

####### **Correspondence:** Valeria Matkava

*Pediatric Rheumatology 2024*, **22(2):**PReS24-ABS-1586

**Introduction:** Fibrodysplasia ossificans progressiva (FOP) is an orphan genetic condition characterized by progressive replacement of muscle and connective tissue with bone due to mutation in the ACVR1 gene. Uncontrolled heterotopic ossification (HO) leads to severe disability in patients. It is crucial to use different informative monitoring techniques for control of HO progression.

**Objectives:** To evaluate available imaging methods and their importance in FOP patients (pts)

**Methods:** From 1998 to 2024 we identified 54 pts with FOP. For routine examination and evaluation of different «flare-up» stages we used wide spectrum of imaging including X-Ray, MRI, CT and low-dose whole-body CT (WBCT).

**Results:** In our cohort X-Ray scan was performed to identify the structure of malformated thumbs and great toes. In all cases we observed clinodactyly with deformity of the 1st metatarsal bones with gradual ankylosis of the 1st interphalangeal joint during follow-up. In one-third pts we verified shortening of metacarpal bones. Also X-Ray scan revealed abnormalities of the cervical spine – ankylosis of facet joints and spinous processes. WBCT with 3D modelling allowed us to measure volume of HO and visualize non-visible ossification and peripheral osteochondromas in all pts. We performed WBCT in 25 pts and in 7 pts we did it twice in 1-year period. We compared HO volume: in 3 cases we identified low progression of HO, in 3 pts – stable status of HO, in 1 case we recognized decreasing of HO volume due to resolving of small ossificates in thoracic area. MRI was effective in visualizing diffuse soft tissue edema during the active phase of a “flare-up” especially in deep localizations such as piriform muscle. In 1 pt we detected subtotal stenosis of the spinal canal without any neurologic symptoms. Also we used MRI for identification of rheumatic signs such as synovitis in large joints and active sacroiliitis which observed in FOP pts very often. We could not perform MRI in some pts due to their inability and installed metallic constructions.

**Conclusion:** All of imaging methods are important for management of FOP patients. X-ray and CT are preferred methods when the enchondral pathway of ossification is complete and visualized. It seems that WBCT plays huge role for FOP patients and may be extrapolated in rheumatological practice while MRI as classical rheumatological imaging may be important method for FOP patients to detect inflammatory signs of FOP nodules, synovitis, sacroiliitis etc.

**Date of birth:** 08.06.1995

**Patient Consent**

Not applicable (there are no patient data)

**Disclosure of Interest**

None Declared

### Imaging

#### P154 Can we examine less fingers without losing sensitivity for detection of abnormal nailfold capillary patterns in juvenile systemic sclerosis, juvenile dermatomyositis and childhood-onset systemic lupus erythematosus?

##### R.A.S. Schutte, S.C. Bergkamp, G. Biesbroek, M.P. Gruppen, A. Nassar-Sheikh Rashid, J.M. van den Berg, D. Schonenberg-Meinema

###### Department of Pediatric Immunology, Rheumatology and Infectious Diseases, Emma Children’s Hospital, Amsterdam University Medical Centres (AUMC), University of Amsterdam, Amsterdam, Netherlands

####### **Correspondence:** R.A.S. Schutte

*Pediatric Rheumatology 2024*, **22(2):**PReS24-ABS-1498

**Introduction:** nailfold capillaroscopy is used in the diagnostic process of systemic autoimmune diseases. International guidelines advise to examine eight fingers (excluding the thumbs). It is unknown if we can examine less fingers while not lowering the sensitivity of detecting abnormal capillary changes.

**Objectives:** what is (per finger) the sensitivity for detection of an abnormal capillary pattern in patients with juvenile systemic sclerosis (jSSc), -dermatomyositis (JDM) and childhood-onset systemic lupus erythematosus (cSLE).

**Methods:** videocapillaroscopy images from eight fingers (n=2464), of which 188 non-evaluable were scored (consensus by 2 experts) from patients with jSSc, JDM and cSLE. Sensitivity for the most abnormal capillary pattern was scored per finger (separately per disease). A scleroderma pattern was defined according to the fast-track algorithm.^1^ Microangiopathy (only in cSLE) was defined as an abnormal capillary pattern but not fulfilling the criteria for a scleroderma pattern. Fingers were numbered by side (2-5L for left and 2-5R for right).

**Results:** in jSSc (n=8), six fingers showed sensitivity of >85% for detection of a scleroderma pattern, except for 5L and 4R (75%). In JDM (n=15), 3L had a sensitivity of 80% for a capillary scleroderma pattern, while it was 46.7-73.3% in the other fingers. In cSLE (n=8), sensitivity for a capillary scleroderma pattern was 75% in 4L, 3R and 4R, but lower in other five fingers (28.6-62.5%). For capillary microangiopathy in cSLE (n=46), sensitivity was 85.7% in 4R and 57.9-78.6% in other fingers.

**Conclusion:** in jSSc the sensitivity of capillary scleroderma pattern was high in most fingers validating examination of less (2-4) fingers in this disease. In JDM and cSLE examination of 4-8 fingers seems necessary for accurate sensitivity of abnormal patterns because these severe capillary changes might otherwise be missed.

**Patient Consent**

Yes, I received consent

**Disclosure of Interest**

None Declared

**Reference**

1. Smith V, Vanhaecke A, Herrick AL, Distler O, Guerra MG, Denton, CP, et al. Fast track algorithm: How to differentiate a “scleroderma pattern” from a “non-scleroderma pattern”. Autoimmun Rev. 2019 Nov;18(11):102394

### Imaging

#### P155 Evaluation of children with sacroiliitis with low-dose computed tomography

##### Yunus E. Bayrak^1^, Törehan Özer^2^, Yonca Anık^2^, Nihal Sahin^1^, Hafize E. Sönmez^1^

###### ^1^Pediatric Rheumatology; ^2^Radiology, Kocaeli University, Kocaeli, Türkiye

####### **Correspondence:** Nihal Sahin

*Pediatric Rheumatology 2024*, **22(2):**PReS24-ABS-1030

**Introduction:** Recent findings from adult studies suggest that low-dose computed tomography (ldCT) may offer a viable alternative for diagnosing sacroiliitis. ldCT examinations, administered at radiation rates comparable to those used in radiography, have been proven to be superior to radiography in detecting structural lesions.

**Objectives:** This study investigated the utility of low-dose computed tomography (ldCT) compared with magnetic resonance imaging (MRI) in diagnosing sacroiliitis in enthesitis-related arthritis (ERA) patients.

**Methods:** For each patient, we assessed the density changes on ldCT at corresponding locations, employing the signal intensity observed on MRI across each joint surface as a reference. The density of regions exhibiting bone marrow edema (heightened signal intensity) on MRI was lower on CT, whereas the density of regions devoid of signal (no bone marrow edema) on MRI was higher on CT.

**Results:** Thirty patients (11 girls/19 boys) diagnosed with ERA were enrolled. The median (IQR) age was 14.44 (6.08) years and the median (IQR) follow-up time was 1.47 (2.05) years. During radiologic evaluation, eighteen patients (60%) exhibited clinical sacroiliitis, and 10 (33.3%) experienced morning stiffness. The median JSpADA was 2.5 (1.3). MRI revealed bilateral bone marrow edema in 22 (73.3%) patients. We assessed the density changes on ldCT at the corresponding locations of the areas of bone marrow edema on MRI. On ldCT evaluation of the right iliac crest, lower density was found on ldCT in regions exhibiting heightened signal intensity in MRI for 20 (66.6%) patients. On the right sacral side, lower density was observed on ldCT in regions exhibiting heightened signal intensity in MRI for 22 (73.3%) patients. On the left iliac crest, lower density was observed in 18 (60%) patients. On the left sacral side, lower density was observed on ldCT in regions exhibiting heightened signal intensity in MRI for 22 (73.3%) patients. A correlation was found between the density measurement in ldCT and the signal intensity in MRI. Erosion was detected in 23 (76.6%) patients on ldCT while erosion was detected in only 11 (36.7%) patients on MRI.

**Conclusion:** This study suggests that ldCT is superior to MRI for early structural change detection. Pixel-based density evaluation in ldCT aligns with MRI findings for bone marrow edema.

**Date of birth:** 21.09.1987

**Patient Consent**

Yes, I received consent

**Disclosure of Interest**

None Declared

### Imaging

#### P156 Ultrasound findings of hands joins in patients with juvenile idiopathic arthritis

##### Olga Alekseeva, Olga Borodacheva, Alexander Volkov, Irina Nikishina

###### V.A. Nasonova Research Institute, Moscow, Russian Federation

####### **Correspondence:** Irina Nikishina

*Pediatric Rheumatology 2024*, **22(2):**PReS24-ABS-1751

**Introduction:** Ultrasound (US) is an important imaging tool for the primary diagnosis of juvenile idiopathic arthritis (JIA), useful for monitoring the disease and evaluating the treatment response. Its main advantages include the safety of the method and accessibility. Hand joints involvement is not as common as in adults with rheumatoid arthritis, and US diagnosis differs in a number of features peculiar to children. US can be easily used to assess joint fluid and synovial membrane proliferation. Power doppler (PD) can detect hypervascularization of the synovial membrane and periarticular soft tissues, which is especially useful when evaluating small joints.

**Objectives:** to evaluate the spectrum of inflammatory changes in the joints of the hands in patients with JIA according to US.

**Methods:** The study included 61 JIA patients (mean age 12.0 [11.0; 15.0] years), who underwent US of hand joints in our center in the past six months. US was performed on expert–class devices using a multi-frequency linear sensor (10-18 MHz) with power Doppler (PD) technique. US signs of synovitis were intraarticular effusion and proliferation of the synovial membrane according to the “gray” scale (B-mode, GS) and hypervascularization of synovia in the PD mode according to the criteria of OMERACT (the Outcome Measures in Rheumatology Clinical Trials). US signs of tenosynovitis were abnormal anechoic or hypoechoic tendon sheath widening, which can be related to both the presence of tenosynovial abnormal effusion or hypertrophy. US examination included the articular zones of the wrist, metacarpophalangeal (MCP), proximal interphalangeal (PIP) joints. This US examination was performed in real clinical practice in patients who according to their physician, had a suspicion of hand joints involvement.

**Results:** The study evaluated 1342 joints in patients with JIA. US signs of synovitis in the GS mode were detected in the wrist joints in 47 patients. Of these, 6 patients showed US signs of increased vascularization (PD+). In 38 patients, US signs of synovitis were detected in the GS mode in MCP joints, and increased vascularization was observed in the PD mode in 4 patients. In 29 patients, signs of synovitis were detected in the PIP joints in the GS mode, of which one patient showed increased vascularization in the PD mode. Additionally, US signs of tenosynovitis were detected in 58 patients.

**Conclusion:** Our data confirmed the significance of US examination of the hand joints in patients with JIA, as the identification of inflammatory changes in these joints can lead to timely diagnosis and the choosing of appropriate therapy.

**Patient Consent**

Yes, I received consent

**Disclosure of Interest**

None Declared

### Imaging

#### P157 Pulmonary hypertension in rheumatic diseases in children - a one-year study

##### Alexander Volkov, Irina Nikishina, Natalia Yudkina

###### V.A. Nasonova Research Institute of Rheumatology, Moscow, Russian Federation

####### **Correspondence:** Irina Nikishina

*Pediatric Rheumatology 2024*, **22(2):**PReS24-ABS-1755

**Introduction:** Pulmonary hypertension is a well-known and potentially dangerous complication of rheumatic conditions, most often seen in adults. Recent reports in medical journals have described isolated cases of pulmonary hypertension in children with rheumatic disease. Given that rheumatic conditions are considered a risk factor for the development of pulmonary hypertension, screening for this condition is essential. Echocardiography, a non-invasive and relatively inexpensive test, can be used to screen for pulmonary hypertension in both adults and children.

**Objectives:** The aim of the study is to investigate the detectability of echocardiographic signs of pulmonary hypertension in children with rheumatic diseases.

**Methods:** A retrospective analysis of medical data from patients admitted to the institute between March 1st, 2023 and March 1st, 2024 was conducted. During this period, a total of 1,567 hospitalizations occurred. Most of these patients were diagnosed with juvenile idiopathic arthritis (43%) and systemic connective tissue disease accounted for 18% of all admissions. During this same time period, 1,281 echocardiograms were performed. These echocardiograms included the measurement of tricuspid regurgitation velocity and the identification of indirect signs of pulmonary hypertension.

**Results:** Only 4 cases of pulmonary hypertension were identified, in one of which the diagnosis of a rheumatic disease could not be confirmed. Two patients, aged 11 and 10, had juvenile idiopathic arthritis. In the first case, pulmonary arterial hypertension was considered a comorbidity (idiopathic pulmonary arterial hypertension), while in the second case it was associated with a congenital heart disease in a patient with Down syndrome. This was a residual condition after surgical treatment for an interventricular septal defect. The third case involved a patient with systemic lupus erythematosus, aged 17, who had mitral and aortic insufficiency as a result of endocarditis. The diagnosis of systemic lupus erythematosus could not be made in a 9-year-old child with pulmonary arterial hypertension, who only had an isolated decrease in C3 complement component. Interestingly, in patients with systemic sclerosis (9% of the total), no signs of pulmonary hypertension were detected. All patients with pulmonary arterial hypertension were treated with a combination of PAH-specific medications.

**Conclusion:** Pulmonary hypertension is a rare condition and a complication of rheumatic diseases. However, it can occur, so it is important for pediatric-rheumatologists to be aware of this condition.

**Patient Consent**

Yes, I received consent

**Disclosure of Interest**

None Declared

**Reference**

1. Humbert M, Kovacs G, Hoeper MM, Badagliacca R, Berger RMF, Brida M, Carlsen J, Coats AJS, Escribano-Subias P, Ferrari P, Ferreira DS, Ghofrani HA, Giannakoulas G, Kiely DG, Mayer E, Meszaros G, Nagavci B, Olsson KM, Pepke-Zaba J, Quint JK, Rådegran G, Simonneau G, Sitbon O, Tonia T, Toshner M, Vachiery JL, Vonk Noordegraaf A, Delcroix M, Rosenkranz S; ESC/ERS Scientific Document Group. 2022 ESC/ERS Guidelines for the diagnosis and treatment of pulmonary hypertension. Eur Respir J. 2023 Jan 6;61(1):2200879. 10.1183/13993003.00879-2022.

### Imaging

#### P158 Can zero echo time magnetic resonance imaging enhance our ability to assess erosion in sacroiliitis more effectively?

##### Yunus E. Bayrak^1^, Yonca Anik^2^, Törehan Özer^2^, Duygu Aydin^1^, Nihal Şahin^1^, Hafize E. Sönmez^1^

###### ^1^Department of Pediatric Rheumatology; ^2^Department of Radiology, Kocaeli University, Kocaeli, Türkiye

####### **Correspondence:** Duygu Aydin

*Pediatric Rheumatology 2024*, **22(2):**PReS24-ABS-1652

**Introduction:** Enthesitis-related arthritis (ERA), a subgroup of JIA, accounts for 18.9% of JIA cases in our region, with a higher prevalence of sacroiliac (SI) joint involvement. At the outset of the disease, approximately 30% of individuals with ERA exhibit symptomatic sacroiliitis, a rate that escalates to 70% over time. Although magnetic resonance imaging (MRI) is the most sensitive radiological method for detecting sacroiliitis, identifying early erosive changes remains still challenging. Conventional MRI pulse sequences have limitations in reliably depicting structures such as osseous and calcified tissues.

**Objectives:** Recently, novel MRI sequences, such as zero echo time (ZTE) MRI utilizing ultrashort echo times (UTEs), have emerged as a viable method to produce computed tomography (CT)-like images of ossified or calcified structures. This study aims to evaluate the utility of ZTE MRI to identify erosion.

**Methods:** All MRI scans were performed using a 1.5 T scanner. The MRI protocol included a fat-suppressed axial T2-weighted sequence, axial T1-weighted sequence, coronal STIR sequence, and axial T2-weighted sequence. Additionally, a ZTE sequence was conducted. The low-dose CT scan was performed using a 640-MSCT device, with patients exposed to an average effective dose of 0.615 mSv per sacroiliac CT. Structural lesions, including erosions, sclerosis, and joint space changes, were compared using ZTE and low-dose CT. Structural lesions, including erosions, sclerosis, and joint space changes were scored based on a previously described method.

**Results:** A total of 20 patients were evaluated (13 boys, 7 girls). Sixteen patients had a family history of rheumatic disease. The median age at diagnosis was 14.44 years, and the median follow-up time was 17 months. At the time of diagnosis, 18 (90%) patients experienced lower back pain, 18 (90%) had hip pain, 15 (75%) reported morning stiffness, 14 (70%) had peripheral arthritis, 8 (40%) experienced heel pain, 7 (35%) had enthesitis, and 5 (25%) had back pain. HLA-B27 was positive in 14 (70%) patients. The median JSpADA at diagnosis and at the time of MRI was 3.7 and 2.7, respectively. During radiologic evaluation, the median counts of active joint and enthesitis were 2 and 0, respectively. Fourteen patients (70%) exhibited clinical sacroiliitis, and 9 (45%) experienced morning stiffness. The mean erosion score on low-dose CT was 9.4, on ZTE-MRI was 8.8, and on MRI 4. The mean sclerosis score on low-dose CT was 9.15, on ZTE-MRI was 9.15, and on MRI 6.15. The mean joint space changes on low-dose CT was 5.1, on ZTE-MRI was 4.95, and on MRI 3.8. The kappa value for the detection of erosions on the quadrant level compared with low-dose CT was 0.514 (p < 0.001), for sclerosis it was 1 (p < 0.001), and for joint space changes, it was 0.814 (p < 0.001).

**Conclusion:** ZTE imaging may produce a visualization of the sacroiliac joints that closely resembles low-dose CT scans, thereby enhancing the detection of subtle erosions and sclerosis in the sacroiliac joints.

**Date of birth:** juin 15, Y

**Patient Consent**

Yes, I received consent

**Disclosure of Interest**

None Declared

### Imaging

#### P159 Validation of the wrist-specific pediatric arthritis ultrasound scoring system in juvenile arthritis

##### Patricia Vega-Fernandez^1^, Kelly Rogers^1^, Pinar Avar-Aydin^1^, Daniel Lovell^1^, Hermine I. Brunner^1^, Mekibib Altaye^2^, Amy Cassedy^2^, Arthur Meyers^3^, Tracy Ting^1^

###### ^1^Rheumatology; ^2^Biostatistics; ^3^Radiology, Cincinnati Children’s Hospital Medical Center, Cincinnati, United States

####### **Correspondence:** Patricia Vega-Fernandez

*Pediatric Rheumatology 2024*, **22(2):**PReS24-ABS-1389

**Introduction:** Wrist involvement in juvenile idiopathic arthritis (JIA) is associated with a high risk of damage and a recommendation for more aggressive treatment(1). The interobserver agreement for clinical assessment of wrist arthritis is low(2). Musculoskeletal ultrasound (MSUS) is an objective imaging technique that can complement the clinical assessment of joint inflammation.

**Objectives:** To validate wrist-specific Pediatric Arthritis Ultrasound Scoring System (PAUSS-wrist)(3) against physical examination (PE) and contrast-enhanced magnetic resonance imaging (ceMRI).

**Methods:** JIA patients with either provider-reported wrist arthritis or patient-reported wrist pain were eligible for this study. At the time of the study visit wrist PE findings for arthritis (presence/absence) by an experienced pediatric rheumatologist, a comprehensive wrist MSUS examination, and a ceMRI of the target wrist were collected. MSUS images of the radiocarpal, intercarpal, and distal radioulnar joints were collected by an American College of Rheumatology MSUS certified pediatric rheumatologist. B-mode and PD-mode images were scored per PAUSS-wrist by pediatric MSUS experts, who were blinded to PE and ceMRI findings. For the current report, MSUS was recorded abnormal if any of the B-mode images had a score of 2 or 3, or if PD images had a score of 1-3. ceMRI scoring was performed according to Rheumatoid Arthritis MRI scoring system (RAMRIS)-wrist as previously done for JIA(4). Associations between the findings on PE and PAUSS-wrist were investigated. Test characteristics of the PAUSS-wrist were evaluated with ceMRI as reference.

**Results:** Sixteen children (mean age of 14.1 years) with JIA were included in the current report. Seven children (44%) had active arthritis per PE. Given scarcity of PD findings, B-mode findings were only included in the analysis. Although a weak correlation between PE and the PAUSS-wrist score was found (correlation (r)= 0.12, p 0.65), the PAUSS-wrist score had a strong correlation with the RAMRIS-wrist score (r=0.73, p<0.001). When using ceMRI-wrist as reference standard, PE and PAUSS-wrist had a sensitivity of 0.48 (95% confidence interval, CI, 0.2-0.7) and 0.94 (CI 0.7-1.0) respectively, for the detection of wrist arthritis. Specificity analysis was limited as all subjects had an abnormal MRI for arthritis.

**Conclusion:** The high sensitivity of the PAUSS-wrist in detecting wrist synovitis per ceMRI and strong correlation of the PAUSS-wrist score with the RAMRIS-wrist, suggest that MSUS provides an objective bedside assessment of wrist arthritis that is superior to PE. Larger studies examining the specificity of PAUSS-wrist are desirable.

**Patient Consent**

Yes, I received consent

**Disclosure of Interest**

P. Vega-Fernandez Grant / Research Support with: Dr. Vega-Fernandez was supported by the National Institutes of Arthritis and Musculoskeletal Skin Diseases [P30AR076316 and K23AR081424A]. , K. Rogers: None Declared, P. Avar-Aydin: None Declared, D. Lovell: None Declared, H. Brunner: None Declared, M. Altaye: None Declared, A. Cassedy: None Declared, A. Meyers: None Declared, T. Ting: None Declared

**References**

1. Ringold S, Angeles-Han ST, Beukelman T, Lovell D, Cuello CA, Becker ML, et al. 2019 American College of Rheumatology/Arthritis Foundation Guideline for the Treatment of Juvenile Idiopathic Arthritis: Therapeutic Approaches for Non-Systemic Polyarthritis, Sacroiliitis, and Enthesitis. Arthritis Rheumatol. 2019;71(6):846-63.
2. Guzman J, Burgos-Vargas R, Duarte-Salazar C, Gomez-Mora P. Reliability of the articular examination in children with juvenile rheumatoid arthritis: interobserver agreement and sources of disagreement. J Rheumatol. 1995;22(12):2331-6.
3. Vega-Fernandez P, Ting TV, Oberle EJ, McCracken C, Figueroa J, Altaye M, et al. Musculoskeletal Ultrasound in Childhood Arthritis Limited Examination: A Comprehensive, Reliable, Time-Efficient Assessment of Synovitis. Arthritis Care Res (Hoboken). 2023;75(2):401-9.
4. Mazzoni M, Pistorio A, Magnaguagno F, Viola S, Urru A, Magnano GM, et al. Predictive Value of Magnetic Resonance Imaging in Patients With Juvenile Idiopathic Arthritis in Clinical Remission. Arthritis Care Res (Hoboken). 2023;75(1):198-205.

### Imaging

#### P160 Beyond enthesitis: unraveling tarsal involvement in familial Mediterranean fever

##### Federica Anselmi^1,2^, Sandrine Chilloh^3^, Linda Rossi-Semerano^1^, Valérie Merzoug^3^, Perrine Dusser^1,2^, Isabelle Kone- Paut^1,2^

###### ^1^Department of Pediatric Rheumatology, National Reference Centre for Auto-inflammatory Diseases and Amyloidosis of Inflammatory origin (CEREMAIA), Bicêtre hospital, Assistance Publique-Hôpitaux de Paris (AP-HP); ^2^Paris Saclay University; ^3^Department of Paediatric Radiology, Bicêtre hospital, Assistance Publique-Hôpitaux de Paris (AP-HP),, Paris, France

####### **Correspondence:** Federica Anselmi

*Pediatric Rheumatology 2024*, **22(2):**PReS24-ABS-1502

**Introduction:** Talalgias and plantalgias are common in patients with Familial Mediterranean Fever (FMF). It is therefore difficult to clinically differentiate these symptoms from those of spondyloarthropathy (SpA), which is often associated with FMF.

**Objectives:** The aim of our study was to evaluate tarsal involvement on MRI in patients with FMF in order to assess the role of this technique in the differential diagnosis with SpA.

**Methods:** we analyzed the records of 167 of our patients with FMF and we selected those who complained of disabling talalgia and underwent an MRI scan. The images were analyzed by expert pediatric radiologists, integrated into the clinical context and compared with the literature.

**Results:** 12/167 patients complained about talalgias. We were able to analyze 3 MRI images (2 girls/1 boy, all M694V/ M694V). The images showed a bone swelling (T2 hypersignal/T1 hyposignal) in the lower part of the body of the calcaneus, at a distance from the physis, with no involvement of the enthesis of the superficial plantar fascia.

It is worth to mention that all of these patients were resistant to colchicine and anti-TNF drugs and required treatment with anti-IL-1 (Canakinumab) to control symptoms.

Discussion: MRI abnormalities of the tarsus in FMF patients complaining with plantalgia or talagia are distinct from those of SpA patients, characterized by a T2 thickening/ hypersignal of the enthesis of the affected tendon with swelling of the adjacent tendon insertion and sometimes bursitis. Inflammatory involvement of the calcaneus on MRI in patients with FMF is therefore a unique entity that differs from enthesitis in patients with SpA.

**Conclusion:** MRI is a useful tool for investigating talalgia in patients with FMF in order to guide the differential diagnosis and adapt the treatment towards blocking IL-1 rather than TNF.

**Patient Consent**

Yes, I received consent

**Disclosure of Interest**

None Declared

### Imaging

#### P161 Innovative use of infrared thermography and computer vision for evaluating inflammatory joints in pediatric rheumatology

##### Meraj A. Siddiqui^1^, Burak Baskın^2^, Esra Baskın^3^

###### ^1^Department of Pediatrics, Baskent University Faculty of Medicine; ^2^Association of Artificial Intelligence and Data Science; ^3^Department of Pediatric Rheumatology, Baskent University Faculty of Medicine, Ankara, Türkiye

####### **Correspondence:** Meraj A. Siddiqui

*Pediatric Rheumatology 2024*, **22(2):**PReS24-ABS-1421

**Introduction:** Pediatric rheumatic diseases pose diagnostic challenges due to their complex array of symptoms and systemic manifestations (1). Timely and precise diagnosis is critical for effectively addressing arthritis-related symptoms such as joint swelling and pain. However, existing diagnostic tools have limitations, highlighting the need for safer and more efficient alternatives (2).

**Objectives:** This study presents an innovative approach using computer vision techniques for the infrared detection of joint inflammation in pediatric rheumatology, aiming to enhance diagnostic accuracy of arthritis.

**Methods:** This single-center, prospective study was conducted at the Başkent University Pediatric Rheumatology Department from December 2022 to June 2023. The study included 18 pediatric patients diagnosed with acute knee arthritis and 19 healthy controls. Thermography was performed under controlled room conditions (Room temperature: 21°C±2°C, Humidity: 50%±5) using the FLIR ONE Edge Pro thermal camera. Thermal images were analyzed using a custom-developed Python software and OpenCV, assessing temperature and color distributions. Statistical analysis was performed using SPSS v25.0, and diagnostic accuracy was evaluated through ROC analysis.

**Results:** The study demonstrated significant differences in maximum and average temperatures between inflamed and non-inflamed joints. The average maximum temperature (T max) in the arthritic patients’ right knees was significantly higher compared to healthy children (33.37±1.57 vs. 31.73±1.23, p = 0.001). Temperature differences between the right and left knees were statistically significant (p < 0.05), with effect sizes (Cohen’s d) ranging from medium to large. In color spectrum analysis, median pixel values in the red spectral band, indicating increased heat due to inflammation, were significantly higher in patients compared to controls [Right knee: 227.5 (170.8-253.1) vs. 175.4 (28.3-242.4), p = 0.006; Left knee: 228.2 (124.7-242.5), p < 0.05] and showed significant effect sizes (r). ROC analysis demonstrated high diagnostic accuracy with AUC values of 0.822 for T max and 0.797 for average temperature (T avg). In this analysis, the Positive Predictive Value (PPV) and Negative Predictive Value (NPV) for T max were calculated as 86.1% and 71.1% respectively. For T avg, the PPV and NPV values were determined as 75.0% and 76.3% respectively. These values indicate that the test has a strong predictive capacity for diagnosing pediatric knee arthritis.

**Conclusion:** This study demonstrates that combining infrared thermography and computer vision techniques in pediatric rheumatology is an effective and non-invasive method for detecting joint inflammation in children. Our research shows that the data obtained using these innovative technologies significantly enhance the accuracy and sensitivity in diagnosing and monitoring joint inflammation. Early detection of disease symptoms, accurate diagnosis, and continuous monitoring can contribute to optimizing treatment processes and improving patients’ quality of life.

**Patient Consent**

Not applicable (there are no patient data)

**Disclosure of Interest**

None Declared

**References**

1. Giancane G, Consolaro A, Lanni S, et al. Juvenile Idiopathic Arthritis: Diagnosis and Treatment. Rheumatol Ther. 2016;3(2):187-207.
2. Garner AJ, Saatchi R, Ward O, et al. Juvenile Idiopathic Arthritis: A Review of Novel Diagnostic and Monitoring Technologies. Healthcare (Basel). 2021;9(12):1683.

### Imaging

#### P162 Nailfold capillaroscopic findings in healthy Indian children

##### Jitendra S. Oswal, Sushma Shree B C

###### Bharati Vidyapeeth Medical College, Pune, India

####### **Correspondence:** Jitendra S. Oswal

*Pediatric Rheumatology 2024*, **22(2):**PReS24-ABS-1456

**Introduction:** Nailfold capillaroscopy (NFC) is a simple diagnostic method used to evaluate morphology of capillary network in nailfold area.

**Objectives:** To describe NFC parameters in a cohort of healthy children in terms of capillary density, capillary dimension and morphology. The study also aimed to assess differences in NFC parameters with age and sex.

**Methods:** The healthy children were recruited from nearest two schools as part of the pilot study. A digital capillaroscope (with 200× magnification) was used to capture nailfold capillary images as per predetermined protocol. These images were analysed with the help of INSPECTIS brand software.

**Results:** Among 248 children screened, 57 were excluded as their NFC images were difficult to analyse due to nail bed hyperpigmentation. A total of 1528 images were assessed from 191 children (mean age 13.3 years), comprising of 136 male and 55 female. The capillary density ranged from 6 to 10, with mean of 7.5 capillaries per mm and mean Standard Deviation (SD) of 1.42. The mean capillary diameter was 17.17μm (range 12 to 30) with a SD of 4.34. Tortuous and ramified capillaries were observed in 41(21%) children. Microhaemorrhages were seen in 9(5%) in the absence of nail bed trauma. There was no difference in NFC changes with age and sex.

**Conclusion:** Our study provides the normal nailfold capillaroscopic parameters in healthy children. The isolated tortuous and ramified capillaries should be considered as non-specific abnormality. The study findings add to the normative NFC data for formulating diagnostic evaluation of children for autoimmune rheumatic diseases.

**Patient Consent**

Not applicable (there are no patient data)

**Disclosure of Interest**

None Declared

### Imaging

#### P163 Tenosynovitis as clue for a diagnosis of Blau syndrome in a child

##### Alessandra Di Nora^1^, Serena Pastore^2^, Andrea Taddio^2,3^, Irene bruno^2^, Martina Girardelli^2^, Erica Valencic^3^, Alberto Tommasini^2,3^

###### ^1^Department of Clinical and Experimental Medicine, Catania; ^2^Institute for Maternal and Child Health IRCCS “Burlo Garofolo”; ^3^University of Trieste, Trieste, Italy

####### **Correspondence:** Serena Pastore

*Pediatric Rheumatology 2024,* **22(2):**PReS24-ABS-1529

**Introduction:** Blau syndrome (BS) is a rare disease characterized by the triad of granulomatous polyarthritis, dermatitis and uveitis.

**Objectives:** To describe peculiar features that cans raise the suspicion of the BS even in the absence of the typical triad.

**Methods:** We reviewed the laboratory tests, the ultrasound (US) and the genetic test of a 8-year-old female with a history of swelling of the wrists and ankles since the age of two years. No significant family history.

**Results:** The patient was referred to our reumatological outpatient at the age of 8 years for strength reduction and stiffness. She presented fluctuant swellings over the dorsal aspect of the wrists and ankles joints with no pain. She underwent US that diagnosed abnormal fat distribution at age of 2 years old. No history of skin lesions. Blood tests showed lymphopenia with high inflammation rates (erythrocyte sedimentation rate, serum immunoglobulin G level and siglec-1 expression). Anti-nuclear antibodies (ANA) were positive with a titer of 1:1280, and rheumatoid factor was negative. No eye involvement was detected upon ophthamological examination.The US revealed marked tenosynovitis of the extensor tendons of the wrist and ankle joints and of the flexor tendons of the fingers and flexors of the ankles, with no Doppler signal. 15 days after the first examination, the child was admitted to our hospital with fever and important limitation of motion of the wrists, fingers and ankles. The laboratory tests revealed worsening inflammation indices. US showed a marked tenosynovitis with strong power Doppler signal. The prominent power Doppler positive synovial hypertrophy of tendons with buggy articular aspect was considered highly suspicious of BS. Indeed, the diagnosis was confirmed by the finding of the *de novo* variant p.R334W in NOD2. We started a brief treatment with oral prednisone (2 mg/Kg/day) with a gradually improvement of the general conditions, and a long term therapy with adalimumab and methotrexate.

**Conclusion:** A prominent tenosynovitis with rapidly changing power Doppler activity, due to the peculiar autoinflammatory nature of the disease, can be a specific clue to the diagnosis of BS, even in the absence of the characteristic triad of clinical manifestations, as reported in our case.

**Patient Consent**

Yes, I received consent

**Disclosure of Interest**

None Declared

**References**

1. 1 Wouters CH, Maes A, Foley KP, Bertin J, Rose CD. Blau syndrome, the prototypic auto- inflammatory granulomatous disease. Pediatr Rheumatol Online J. 2014;12:33.
2. 2 Banday AZ, Mukherjee S, Kumrah R, Suri D, Jindal AK. Prominent tenosynovitis on ultrasonography: A useful sign for the diagnosis of Blau syndrome. Eur J Rheumatol. 2022 Jan;9(1):58-59. 10.5152/eurjrheum.2020.20187. PMID: 33687830; PMCID: PMC10089145.
3. 3 Wouters CH, Maes A, Foley KP, Bertin J, Rose CD. Blau syndrome, the prototypic auto-inflammatory granulomatous disease. Pediatr Rheumatol Online J. 2014 Aug 6;12:33. 10.1186/1546-0096-12-33. PMID: 25136265; PMCID: PMC4136643.
4. 4 Brown, W., Bonar, S.F., McGuigan, L. et al. Blau syndrome: a rare cause of exuberant granulomatous synovitis of the knee. Skeletal Radiol 49, 1161–1166 (2020). 10.1007/s00256-020-03376-1.

### Imaging

#### P164 Assessment of the nailfold microvasculature in pediatric patients with primary and secondary raynaud’s phenomenon

##### Yolanda Macías Huerta, Ana V. Villarreal Treviño, Fernando García Rodriguez, Ana Karen Leos Leija, Nadina Rubio Perez, Lourdes Aldana Galvan

###### Department of Pediatric Rheumatology, University Hospital “Dr. José Eleuterio González”., Nuevo León , Mexico

####### **Correspondence:** Ana V. Villarreal Treviño

*Pediatric Rheumatology 2024*, **22(2):**PReS24-ABS-1592

**Introduction:** Periungual capillaroscopy has proven to be a useful tool in Rheumatology, being non-invasive and safe, allowing the evaluation of morphological characteristics and alterations of the nailfold capillaries. Raynaud’s Phenomenon (RP), a clinical condition commonly found in patients with Rheumatic Diseases, requires capillaroscopic evaluation to differentiate primary (pRP) from secondary Raynaud’s Phenomenon (sRP), and helps monitor disease activity, assess treatment response, and prognosis. To date, there is limited data in the literature regarding capillaroscopic characteristics in pediatric populations.

**Objectives:** Describe qualitative, semi-quantitative, and quantitative capillaroscopic characteristics and differences among pediatric patients with Primary and Secondary Raynaud’s Phenomenon.

**Methods:** A cross-sectional, observational, and descriptive study in which were included patients from the Pediatric Department under Pediatric Rheumatology follow-up at a tertiary center in Monterrey, Nuevo León, Mexico, from July 2017 to October 2023. Eight fingers, excluding the thumb, were analyzed, obtaining 4 images per finger in quadrants A, B, C, and D of both hands, using a x200 videocapillaroscope (Optilia). Demographic data were collected to describe diagnosis, age, and presence RP. Qualitative and semiquantitative data included capillaroscopic pattern (Cutolo), density, architecture, arborized capillaries, microhemorrhages, and avascular areas; for quantitative characteristics of each finger, length, apical, afferent, and efferent diameter were obtained.

**Results:** 1088 images from 34 patients were evaluated, consisting of 25 girls and 9 boys, with an average age of 13 years. Of the total patients, 11 had a diagnosis of primary Raynaud’s Phenomenon and 23 had secondary Raynaud’s Phenomenon due to SLE (26%), JIA (24%), systemic sclerosis (6%), mixed connective tissue disease (6%), and IgA vasculitis (6%). Among qualitative characteristics, we found a higher frequency of non-specific alterations (55.8%), mainly represented by the sRP group, in comparison to the pRP group in which the normal patron was more frequent (81%). In the quantitative evaluation, significant differences were observed in the capillary density, length, apical and arterial diameter values in both groups in the 4th finger of the right and left hand. Additionally, measurements obtained in patients with SLE and JIA were compared, where statistically significant differences (p <0.05) were found in apical and arterial diameter in the fourth finger of both hands.

**Conclusion:** Significant qualitative and semi-quantitative changes were observed in the sRP group. The quantitative characteristics of nailfold microvasculature morphology were identified in patients with pRP and sRP, where statistically significant differences were observed in the fourth finger of the left hand in both groups, highlighting the apical diameter and arterial branch width. Despite finding alterations in patients’ images, it would be useful to conduct studies with controls in healthy children to reach more solid conclusions about the characteristics present in patients with Raynaud’s Phenomenon.

**Patient Consent**

Not applicable (there are no patient data)

**Disclosure of Interest**

None Declared

**References**

1. Smith V, Herrick AL, Ingegnoli F, Damjanov N, De Angelis R, Denton CP, et al. Standardisation of nailfold capillaroscopy for the assessment of patients with Raynaud’s phenomenon and systemic sclerosis. Autoimmun Rev. marzo de 2020;19(3):102458.
2. Smith V, Ickinger C, Hysa E, Snow M, Frech T, Sulli A, et al. Nailfold capillaroscopy. Best Pract Res Clin Rheumatol. marzo de 2023;37(1):101849.

### Imaging

#### P165 Imaging in CACP syndrome: clues for differential diagnosis from juvenile idiopathic arthritis

##### Federica Anselmi^1,2^, Charlotte Borocco^3^, Mouna Barat^4,5^, Raphaele Seror^6,7^, Linda Rossi-Semerano^1^, Isabelle Kone- Paut^1,2^

###### ^1^Department of Pediatric Rheumatology, National Reference Centre for Auto-inflammatory Diseases and Amyloidosis of Inflammatory origin (CEREMAIA), Bicêtre hospital, Assistance Publique-Hôpitaux de Paris (AP-HP); ^2^Paris Saclay University., Paris; ^3^Department of Pediatrics, Centre hospitalier Aix-en-Provence, Aix-en-provence; ^4^Department of Molecular Genetics and Cytogenomics, Rare and Auto Inflammatory Diseases Unit, , CHU Montpellier; ^5^university of montpellier, montpellier; ^6^Department of Rheumatology, Hôpitaux Universitaires Paris-Sud, Assistance Publique-Hopitaux de Paris; ^7^Université Paris-Sud, INSERM U1012 , Paris, France

####### **Correspondence:** Federica Anselmi

*Pediatric Rheumatology 2024*, **22(2):**PReS24-ABS-1410

**Introduction:** Camptodactyly-Arthropathy-Coxa vara-Pericarditis syndrome(CACPs) is a rare genetic disorder commonly misdiagnosed as juvenile idiopathic arthritis(JIA). It is characterized by early onset non-inflammatory arthropathy of large joints, coxa vara deformity, sterile pericarditis and camptodactily. Unlike the arthropathy, not all the clinical features are present in childhood. Including the search for diagnostic clues for CACPs in diagnostic workup of JIA is crucial in order to distinguishing between these conditions.

**Objectives:** Here we describe two girls with CACPs, initially referred for JIA, and highlight some imaging findings that should alert clinicians to a diagnosis of CACPs

**Results:** Both patients were referred to our centre at the age of 12. Of note, the first symptoms (mechanical pain, progressive stiffness) appeared at the ages of 5 and 7 years respectively. At the initial assessment they exhibited bilateral extensive polyarticular involvement with joint swelling, limited range of motion and bilateral camptodactily of the 5^th^(patient 1) and of the 1^st^ finger(patient 2) and of several toes. Neither patient had elevated inflammatory markers nor autoantibodies. Synovial fluid analysis revealed non-inflammatory fluid and presence of red blood cells, lymphocytes and macrophage synoviocytes, with absent/very low count of neutrophils. Several joint injections as well as different immunomodulatory treatments(methotrexate, anti-TNF-alpha, anti-IL-6,JAK-inhibitors, anti-CTLa4) were tested in both patients with no efficacy. Due to the atypical presentation for JIA, several imaging examinations were performed. X-RAYs showed flattening of the epiphyses (radius, ulna, femoral condyles) with a progressive joint narrowing (wrist and fingers) in both patients. Neither patient had coxa vara. Nevertheless, they had hip abnormalities, such as flattening of the femoral heads and short necks. Diffuse osteopenia was also present. Ultrasound (US) revealed huge effusion in the joints and synovial sheaths with hyperechoic spots and mild synovial hypertrophy and no or few doppler signals. MRI (of the knee in patient 1 and of the wrist in patient 2) showed significant effusion and enhanced synovial uptake. Genetic testing showed homozygous mutation in the PRG4 gene in both patients, thus confirming the diagnosis of CACPsyndrome.

**Conclusion:** In children with joint swelling, stiffness and limited range of motion that may mimic JIA, with non-inflammatory pain and no response to JIA treatments, imaging findings such as shortened femoral necks, epiphyseal flattening of long bones and the distinctive US features described above should prompt consideration of a CACPsyndrome.Larger studies are needed to validate the suggestive radiological features of CACP observed in our patients.

**Patient Consent**

Yes, I received consent

**Disclosure of Interest**

None Declared

### Immunodeficiency and infection related arthritis

#### P166 Immunodeficiency due to a novel variant in PIK3CD

##### Reza Shiari^1^, Niloofar Shashaani^1^, Azadeh Zeinab Mirzaee^2^, Khosro Rahmani^1^, Vadood Javadi Parvaneh^1^, Mehrnoush Yeganeh^1^, Fatemeh Freshteh Mehregan^1^

###### ^1^Pediatric Rheumatology, Mofid Children Hospital; ^2^Pediatric Rheumatology, Masih Daneshvari Hospital, Tehran, Iran, Islamic Republic Of

####### **Correspondence:** Niloofar Shashaani

*Pediatric Rheumatology 2024*, **22(2):**PReS24-ABS-1765

**Introduction:** Abstract

Background Primary immunodeficiencies are immunological disorders caused by gene mutations involved in immune system development and activation. Recently, activated phosphoinositide 3-kinase delta syndrome (APDS) due to mutations in the phosphoinositide 3-kinase (PI3K), phosphatidylinositol-4, 5-bisphosphate 3-kinase, catalytic subunit delta gene (PIK3CD), and phosphoinositide 3-kinase regulatory subunit 1 (PIK3R1) genes have been reported to induce a combined immunodeficiency syndrome leading to senescent T cells, lymphadenopathy, and immunodeficiency. The exact diagnosis of these deficiencies is essential for treatment and prognosis. In recent years, targeted treatment with selective PI3Kd inhibitors has had a significant effect on controlling the symptoms of these patients

**Objectives:** whole blood sample. Variant interpretation of interested variants was accomplished through the American College of Medical Genetics and Genomics (ACMG). A novel heterozygous variant (c.1429 G>A; p.Glu477Lys) was found in the PIK3CD gene (Table 2). The variant was validated in the patient, and segregation analysis showed the mother is the carrier for the variant. According to the ACMG guideline, this variant can be classified as a Variant of Unknown Significance (VUS).

**Methods:** .

**Results:** In this report, we described a girl with a novel mutation in the PIK3CD gene. She had a recurrent fever, and erythema nodosum with the manifestations of combined immunodeficiency in immunological investigations. She experienced several episodes of viral and bacterial infections, autoimmune disorders (hypothyroidism and type 1 diabetes mellitus), and auto-inflammatory manifestations (left knee arthritis, pericardial effusion).

**Conclusion:** Conclusion The genetic analysis found a novel variant of PIK3CD (c.1429 G>A) in the patient. Following daily antibiotic prophylaxis and monthly IV therapy, the patient’s frequent infections and fevers were controlled.

**Date of birth:** décembre 3

**Patient Consent**

Not applicable (there are no patient data)

**Disclosure of Interest**

None Declared

**References**

1. Tangye SG, Al-Herz W, Bousfiha A, Chatila T, Cunningham-Rundles C, Etzioni A, et al. Human inborn errors of immunity: 2019 update on the classification from the International Union of Immunological Societies Expert Committee. J Clin Immunol. 2020;40(1):24–64.
2. Chi CY, Lin CH, Ho MW, Ding JY, Huang WC, Shih HP, et al. Clinical manifestations, course, and outcome of patients with neutralizing anti-interferon-γ autoantibodies and disseminated nontuberculous mycobacterial infections. Medicine. 2016;95(25):e3927.
3. Notarangelo LD. Primary immunodeficiencies. J Allergy Clin Immunol. 2010;125(2 Suppl 2):182–94.

### Immunodeficiency and infection related arthritis

#### P167 Childhood onset Sjogren disease as an immunodisregulative process: high frequency of immunodeficiency in a single cohort

##### Edoardo Marrani^1^, Marco Renni^1^, Valerio Maniscalco^1^, Ilaria Maccora^1^, Maria Vincenza Mastrolia^1^, Ilaria Pagnini^1^, Gabriele Simonini^1,2^

###### ^1^Pediatric Rheumatology Unit, Meyer Children’s Hospital IRCCS; ^2^NEUROFARBA, University of Firenze, Firenze, Italy

####### **Correspondence:** Maria Vincenza Mastrolia

*Pediatric Rheumatology 2024*, **22(2):**PReS24-ABS-1673

**Introduction:** Childhood-onset Sjogren Disease (coSjD) is an underdiagnosed disease which present unique diagnostic and therapeutic challenges. Once considered an inflammatory disorder limited to salivary and lacrimal glands, recent evidence uggests that in children and young adults SjD lead to a systemic inflammatory state. Immunodisregulative processes are associated with higher disease burden and high risk of malignancies. In adults an overlap between Common Variable Immunodeficiency and Sjogren Disease is reported. However, no studies address the frequency of immunodeficiency within coSjD cohorts remain scarce.

**Objectives:** This study aimed to investigate the prevalence of immunodeficiency in a single cohort of pediatric patients with coSjD. Specifically, we sought to determine the frequency of immunodeficiency among patients diagnosed with coSjD and explore its implications for disease management and prognosis.

**Methods:** We conducted a retrospective analysis of medical records from a cohort of pediatric patients diagnosed with coSjD and in follow-up at our unit between 2022 and 2024. Demographic data, clinical presentations, laboratory findings, and immunological profiles were extracted and analyzed. Immunodeficiency was defined based on established criteria, including quantitative and qualitative abnormalities in immunoglobulin levels, lymphocyte subsets, functional assays or genetic confirmatory tests.

**Results:** Among the cohort of pediatric patients diagnosed with coSjD (n = 20), we identified a strikingly high frequency of immunodeficiency, with 20% (4 out of 20) of patients meeting criteria for immunodeficiency. One patient has a chromosomal disorder associated with immunodeficiency, one patient suffers of genetical confirmed Iper-IgE syndrome, one patient has a genetical confirmed APECED syndrome due to mutation of AIRE gene and the last patient suffers of a complex phenotype characterized by reduced IgG, T- and CD19 deficiency, chronic skin disorder, interstial lung disease suggestive of an *PI3K* deficiency (exome analysis ongoing). The diagnosis of SjD followed the diagnosis of immunodeficiency in 3 cases and relies on a combination of minor salivary glands biopsy and on ultrasounds of the salivary glands. Due to the limited sample size, no significant differences between immunodeficiency associated SjD and coSJD without immunodeficiency was found. Immunosuppressive treatment was required in three patients (mycophenolate mofetil in two, rituximab in combination with sirolimus in one patient) while the other patient received hydroxicloroquine without significant benefit (persistence of parotiditis).

**Conclusion:** Our study highlights the significant prevalence of immunodeficiency as a comorbidity in pediatric patients with coSjD, emphasizing the need for heightened awareness and proactive management strategies. Early identification of immunodeficiency in these patients is crucial for mitigating infectious risks and optimizing long-term outcomes. Clinicians should maintain a high index of suspicion for coSjD in immunodeficient patients presenting with recurrent parotitis, connective tissue disease symptoms, or unexplained elevations in inflammatory markers. Salivary gland ultrasound emerges as a promising screening tool in this context. Further studies in larger cohorts are warranted to elucidate the underlying mechanisms linking immunodisregulation and coSjD,

**Patient Consent**

Yes, I received consent

**Disclosure of Interest**

None Declared

### Immunodeficiency and infection related arthritis

#### P168 Differences in presentation between children with septic and non-septic arthritis: a retrospective multicenter study

##### Najma Abdullahi^1,2^, Moniek Corcoran^3^, Yvonne Koopman-Keemink^4^, Jeroen Noordzij^5^, Olivier Somers^2^, Ilse Tromp^3^, Anneloes de Vries^4^, Sylvia Kamphuis^1^, Xandra van den Tweel^1, 2^

###### ^1^Pediatric Rheumatology/Immunology, Sophia Children’s Hospital, Erasmus University Medical Center; ^2^Pediatrics, Maasstad Hospital, Rotterdam; ^3^Pediatrics, Albert Schweitzer Hospital, Dordrecht; ^4^Pediatrics, Haga Hospital, The Hague; ^5^Pediatrics, Reinier de Graaf Hospital, Delft, Netherlands

####### **Correspondence:** Najma Abdullahi

*Pediatric Rheumatology 2024*, **22(2):**PReS24-ABS-1290

**Introduction:** A common cause of arthritis in children is reactive arthritis, which most often is self-limiting. On the contrary, septic arthritis, which is less common, demands immediate treatment with intravenous antibiotics to prevent long-term joint damage. At first presentation, it often is a diagnostic challenge to differentiate between septic and non-septic arthritis.

**Objectives:** To compare characteristics of children aged 6 months to 18 years old with septic arthritis and non-septic arthritis.

**Methods:** We conducted a retrospective, multicenter cohort study at four general hospitals in the Netherlands, including children with a first-onset arthritis lasting less than 6 weeks between March 1, 2011, and March 1, 2021. Eligible subjects were retrieved using International Classification of Diseases (ICD) codes. We collected clinical, laboratory and imaging data of these patients. Patients with immunodeficiency, acute rheumatic fever, IgA vasculitis and other arthritis related diseases were excluded. Group comparisons were performed using Chi-square/Fisher’s exact tests and Mann-Whitney U test.

**Results:** A total of 352 patients, with a median age of 4 years (IQR 2-8), were included: 48 with septic arthritis (14%) and 304 with non-septic arthritis (86%). Among the septic cases, 46% showed positive cultures, 38% negative cultures, 8% involved Kingella kingae cases, and in 8% the diagnosis was made clinically without cultures taken. Diagnoses in non-septic arthritis cases included reactive arthritis (81%, including 27 cases of post-streptococcal reactive arthritis), JIA (12%), and other diagnoses (7%). Septic arthritis patients differed significantly from non-septic patients regarding the presence of fever (72% vs. 38%, p<0.001), number of affected joints (median 1 (IQR 1-1) vs. median 1 (IQR 1-2), p <0.001), and limited range of motion (LROM) (93% vs. 73%, p=0.008). The knee joint was more affected in the non-septic group compared to the septic group (58% vs. 35%, p=0.004). Inflammatory markers were higher in children with septic arthritis: CRP (median 76 mg/dl vs. 13 mg/dl, p<0.001) and ESR (median 43 mm/h vs. 25 mm/h, p<0.001) and ultrasound abnormalities were seen more frequently in septic arthritis (92% vs. 72%, p=0.008).

**Conclusion:** In this study, we observed significant differences in the presence of fever, LROM, and inflammatory markers between children with septic and non-septic arthritis. Further research is planned to develop a prediction tool at presentation that helps in distinguishing septic arthritis from non-septic arthritis.

**Patient Consent**

Not applicable (there are no patient data)

**Disclosure of Interest**

None Declared

### Immunodeficiency and infection related arthritis

#### P169 Challenges in diagnosing children with Poststreptococcal Reactive Arthritis (PSRA): a retrospective multicenter cohort-study

##### Najma Abdullahi^1,2^, Estrella Both^1^, Ilse Tromp^3^, Olivier Somers^2^, Anneloes de Vries^3^, Yvonne Koopman-Kemink^4^, Jeroen Noordzij^5^, Moniek Corcoran^3^, Xandra van den Tweel^2^, Sylvia Kamphuis^1^

###### ^1^Rheumatology, Erasmus University Medical Center; ^2^Pediatrics, Maasstad Hospital, Rotterdam; ^3^Pediatrics, Albert Schweizer Hospital, Dordrecht; ^4^Pediatrics, Haga Hospital, The Hague; ^5^Pediatrics, Reinier de Graaf Hospital, Delft, Netherlands

####### **Correspondence:** Estrella Both

*Pediatric Rheumatology 2024*, **22(2):**PReS24-ABS-1496

**Introduction:** Poststreptococcal Reactive Arthritis (PSRA) is a reactive arthritis caused by group A streptococcal (*GAS*) pharyngitis.Currently there is a lack of knowledge and consensus regarding its clinical characteristics and prognosis. Available studies are hampered by low sample size and the lack of a generally accepted definition of PSRA. Proofing the antecedent *GAS* infection is challenging as *GAS* carriers may also have positive throat cultures and show elevated anti-streptococcal titers. Lastly, there’s no consensus regarding the potential risk of cardiac sequelae in PSRA.

**Objectives:** To demonstrate how PSRA is diagnosed in clinical practice and to identify knowledge gaps regarding the ability to accurately diagnose PSRA.

**Methods:** We performed a ‘real-life’ retrospective multicenter cohort study. Clinical and laboratory data including streptococcal serology (ASO, anti-DNAse) was collected from children aged 0,5-18 years presenting between 01/03/2011 and 01/03/2021 with first-onset arthritis < 6 weeks. Patients fulfilling criteria for acute rheumatic fever were excluded. Eligible subjects were retrieved using International Classification of Diseases (ICD) codes.

**Results:** We included 352 patients and identified two groups of patients defined by whether ASO with or without anti-DNAse was performed or not. Fifteen patients with anti-DNASe but no ASO were excluded. Patients (n=186) with ASO +/- anti-DNAse had a slightly higher frequency of pharyngitis and lower frequency of mono-arthritis, when compared to patients (n=151) who were not tested for streptococcal serology. Initial ASO titers were positive in 32% of patients. Only in a minority (19%) streptococcal serology was repeated over time. Twenty-seven patients of 186 patients (14%) were diagnosed with PSRA. Only 6/27 patients had a recorded pharyngitis prior to the onset of arthritis. Mono-arthritis was the most frequent presentation (53%) with the knee being most commonly affected. Median arthritis duration was 5 days (range 1-59). Initial ASO/anti-DNase titers were only slightly higher in patients diagnosed with PSRA (708 and 653 IU/ml respectively) compared to patients not diagnosed with PSRA despite positive serology (541 and 508 IU/ml respectively). None of the patients (n=352) developed cardiac abnormalities.

**Conclusion:** In our study it was not possible to distinguish children with PSRA from children without PSRA based on clinical characteristics or the results of streptococcal serology. This underlines the diagnostic challenges of PSRA, yet confirms that children with first onset arthritis and positive streptococcal serology may have a negligible risk of developing cardiac abnormalities after acute rheumatic fever is excluded.

**Patient Consent**

Yes, I received consent

**Disclosure of Interest**

None Declared

### Immunodeficiency and infection related arthritis

#### P170 Mycoplasmal septic arthritis, central nervous system vasculitis and the unveiling of a late-onset agammaglobulinemia: a case report

##### Monthira Chowichian

###### Pediatric, Phramongkutklao Hospital, Bangkok, Thailand

*Pediatric Rheumatology 2024*, **22(2):**PReS24-ABS-1511

**Introduction:** *Mycoplasma pneumoniae* is a common respiratory pathogen in school-age children and rarely fatal. Extrapulmonary involvement in musculoskeletal and central nervous system is expected to be found but is unusual. Furthermore, septic arthritis and central nervous system (CNS) vasculitis without preceding respiratory symptoms are limitedly reported, even in immunodeficiency patients, especially in cases of X-linked agammaglobulinemia (XLA)

**Objectives:** To describe the rare manifestation in a case of late-onset XLA with *M. pneumoniae* septic arthritis and CNS vasculitis presents a challenge in diagnosis.

**Methods:** A case report

**Results:** A 6-year-old boy presented with acute fever and asymmetrical polyarthritis for 7 days prior to admission. Initially, he experienced fever with joint pain at right elbow, right 2^nd^ proximal interphalangeal joint (PIP), hip and left knee. He had no recent history of respiratory tract infection. In his past medical history, he was diagnosed incomplete Kawasaki disease three years ago and acute disseminated encephalomyelitis after receiving the vaccine two years ago. Currently, he is wheelchair-bound with self-sitting support. On admission, his temperature was 39.2°C, pulse rate 160 bpm, blood pressure 110/70 mmHg and respiratory rate 24/min. Physical examination demonstrated tenderness in the right elbow, 2^nd^ PIP, hip and left knee with warmth and mild swelling. The investigation showed hemoglobin 10.2 g/dl, white blood cell count 21.7 x 10^9^/L (neutrophil 81%, lymphocytes 11%) and platelets 969 x 10^9^/L. The erythrocyte sedimentation rate was 26 mm/hr. C-reactive protein was 186.19 mg/L. Ultrasound-guided right hip arthrocentesis was performed and joint fluid analysis showed turbid fluid with white blood cell count 6.32 x 10^9^/L (neutrophil 97%) and no organism. Cloxacillin was prescribed intravenously. At the 5^th^ day of admission, he developed alteration of consciousness. An MRI brain showed hyperdense lesion at brainstem and scatter lesions at cortex, suggestive of multiple sclerosis (MS). A lumbar puncture was done and cerebrospinal fluid (CSF) analysis was normal, with no autoimmune encephalitis detected. The patient received antibiotics and underwent plasmapheresis as a treatment for autoimmune encephalitis. A few days later, he developed seizure, a CT angiography(CTA) of the brain showed vasculitis affecting medium to small vessel. Subsequent 16S rRNA gene sequencing of the joint fluid from both the right hip and left knee identified *M. pneumoniae.* Following further evaluation, the patient’s clinical presentation and investigation findings were consistent with XLA.

**Conclusion:** Common organisms, if present in atypical presentations and organ involvement, should be evaluated to determine the cause of the uncommon. In children, concerns regarding immunodeficiency disorders should arise when encountering atypical infections and manifestations.

**Date of birth:** septembre

**Patient Consent**

Yes, I received consent

**Disclosure of Interest**

None Declared

### Immunodeficiency and infection related arthritis

#### P171 Acute rheumatic fever preceding enthesitis related juvenile idiopathic arthritis

##### Paivi Miettunen^1,2^, Wisoo Shin^1^, Samarjeet Bhandal^3^

###### ^1^Pediatrics, University of Calgary; ^2^Pediatric Rheumatology, Alberta Children’s Hospital; ^3^Diagnostic Imaging, University of Calgary, Calgary, Canada

####### **Correspondence:** Paivi Miettunen

*Pediatric Rheumatology 2024*, **22(2):**PReS24-ABS-1261

**Introduction:** Acute rheumatic fever (ARF) is an inflammatory reaction secondary to group A streptococcal pharyngitis, diagnosed by modified Jones’ criteria^1^. Painful, migratory arthritis/arthralgia is common, it typically affects the large joints, lasts < 3 weeks, and responds to salicylates. Enthesitis related juvenile idiopathic arthritis (ERA) is a type of juvenile idiopathic arthritis (JIA), diagnosed by ILAR criteria^2^ ,requiring > 6 weeks of arthritis/enthesitis. We now present a pediatric patient who developed ERA shorty after ARF diagnosis.

**Objectives: Case:** An 8-year-old boy developed fleeting musculoskeletal complaints. One week later he presented to family doctor with a sore throat and migratory hip and knee pain with unremarkable radiographs and a negative throat swab. Two weeks later, he presented in ER with a history of fever (>38.5C), worsening migratory arthralgia with a normal musculoskeletal (MSK) exam and symptom resolution with one dose of ibuprofen (afebrile in ER). Viral infection was considered. One week later he re-presented in ER with ongoing joint complaints. Investigations revealed positive ASOT (716 IU/mL, normal (N)<536 IU/mL), elevated ESR (70 mm/hr, N<10mm/hr) and CRP (15.1 mg/L, N<8mg/L), normal hemoglobin ( Hgb) and white blood cells ( WBC).

**Methods:** Rheumatology was consulted. On exam, he had painful and decreased range of motion (ROM) of shoulders, hips, and left wrist. He met the Jones’ criteria for ARF (one major criteria (arthritis) and 2 minor criteria (fever and increased ESR/CRP) with positive ASOT. He was started on Aspirin and amoxicillin (x 10 days); had an ECG and ECHO, which were normal. One week later he re-presented to ER with reduced and painful ROM of the R hip, with resolution of all other MSK complaints. Ultrasound demonstrated a moderate R hip effusion with synovial thickening. Bloodwork revealed ESR 69 mm/hr, CRP 13.3 mg/L, normal WBC and Hgb, and rising ASOT of 762 IU/mL. Septic joint, infectious osteomyelitis, transient synovitis of the hip or evolving JIA were queried. HLA-B27 and an urgent MRI were ordered.

**Results:**

**HLA-B27:** negative

**MRI bilateral hips and pelvis revealed:** a) Right hip synovitis, no imaging evidence of osteomyeltitis; b) bilateral sacroiliitis; c) marrow edema of bilateral femoral trochnaters and right ischio-pubic ramus and d) no evidence of myositis/subperiosteal abscess.

His R hip symptoms persisted at 7 weeks, ruling out transient synovitis. He now met the ILAR criteria for ERA (Arthritis/enthesitis > 6 weeks with bilateral sacroiliitis). Aspirin was switched to Naproxen (15 mg/kg/day) with steady improvement in his symptoms. He requires ongoing penicillin prophylaxis for ARF.

**Conclusion:** The atypical articular course for ARF triggered a concern for a potential infection or evolving JIA. MRI was important in ruling out osteomyelitis or abscess and diagnosing sacroiliitis/enthesitis. This case highlights the importance of clinical follow-up of ARF patients with persisting joint symptoms.

**Patient Consent**

Yes, I received consent

**Disclosure of Interest**

None Declare

**References**

1. Michael H. Gewitz, Robert S. Baltimore, Lloyd Y. Tani, Craig A. Sable, Stanford T. Shulman, Jonathan Carapetis, Bo Remenyi, Kathryn A. Taubert, Ann F. Bolger, Lee Beerman, Bongani M. Mayosi, Andrea Beaton, Natesa G. Pandian and Edward L. Kaplanand on behalf of the American Heart Association Committee on Rheumatic Fever, Endocarditis, and Kawasaki Disease of the Council on Cardiovascular Disease in the Young. *Revision of the Jones Criteria for the Diagnosis of Acute Rheumatic Fever in the Era of Doppler Echocardiography. A Scientific Statement From the American Heart Association.* Circulation. 2015;131:1806–1818
2. Youn-Soo Hahn. *Enthesitis-related Arthritis.* J Rheum Dis 2018; 25(4): 221-230

### Immunodeficiency and infection related arthritis

#### P172 Kingella Kingae – silent cause of the osteoarticular infections in pediatric population

##### Gabriela Živković^1^, Benjamin Vukalović^2^, Mandica Vidović^3^

###### ^1^Division for Neonatology, Department of Obstetrics and Gynecology, University Hospital Center Sestre milosrdnice; ^2^Community Health Center of Zagreb County; ^3^Division for Pediatric Reumathology, Department of Pediatrics, University Hospital Center Sestre milosrdnice, Zagreb, Croatia

####### **Correspondence:** Mandica Vidović

*Pediatric Rheumatology 2024*, **22(2):**PReS24-ABS-1515

**Introduction:** *Kingella kingae* is a β-hemolytic member of the *Neisseriaceae* family and a very common cause of osteoarticular infections in the 6 months to 4 years age group. Initially asymptomatic colonisation of the posterior pharyngeal mucosa and excretion of a repeat-in-toxin (RTX) may contribute to development of mild clinical manifestations as a first sign of invasive infection.

**Objectives:** We present a case report of a child who was treated at our tertiary care pediatric center due to suspected osteomyelitis caused by *K. kingae*.

**Methods:** We retrospectively analyzed the patient’s medical records.

**Results:** A two years old girl was hospitalized due to pain in lower extremities, limited mobility and an inflammatory markers’ increase. Parenteral administration of clindamycin and cefazolin was initiated at the admission. Scintigraphy showed discrete uptake of radioisotope in the right hip with no elements for osteomyelitis. However, in the pharyngeal mucosa *K. kingae* was isolated, and septic arthritis with possible complication of osteomyelitis was suspected. Screening for celiac disease, antinuclear antibodies and rheumatoid factor were negative. Initially started antibiotic therapy was changed to cefixime per os, and regression of symptoms was observed. Antibiotic therapy was administered for 14 days in total. Control laboratory findings were normal, and ultrasound of the hip at the discharge showed regression of pericapsular edema.

**Conclusion:** A *K. kingae* osteoarticular infection (OAI) is often characterized by a mild clinical presentation and a limited inflammatory response, whilst pyogenic infection caused by a more aggressive pathogen (e.g., streptococci and methicillin-sensitive or methicillin-resistant *Staphylococcus aureus*), are easier to detect, with affected children appearing ill, with high fever and an elevated white blood cell count. Despite mild clinical presentation of *K. kingae* OAI, parenteral administration of penicilinase-stable β-lactam antibiotic and a broad-spectrum second or third-generation cephalosporin should be initiated in suspected cases, especially in the 6 months to 4 years age group, due to possible development of complications and long-term impaired function. A good clinical response and decrease of CRP levels may lead to switching to oral antibiotics. Antibiotics can be administered for a total of 2-3 weeks for arthritis and 3-6 weeks for osteomyelitis.

**Patient Consent**

Yes, I received consent

**Disclosure of Interest**

None Declared

**References**

1. Yagupsky P. Kingella kingae: carriage, transmission, and disease. Clin Microbiol Rev. 2015;28:54–79.
2. Cochard B, De Marco G, Bazin L, Vazquez O, Di Laura Frattura G, Steiger CN, Dayer R, Ceroni D. Biological Predictors of Osteoarticular Infection Due to *K. kingae*-A Retrospective Cohort Study of 247 Cases. Microorganisms. 2023;11(9):2130.
3. Yagupsky P. Kingella kingae: from medical rarity to an emerging paediatric pathogen. Lancet Infect Dis. 2004;4:32–41.

### Immunodeficiency and infection related arthritis

#### P173 Osteomyelitis in immunocompromised neonate - a case report

##### Eka Nakhutsrishvili, Julieta Kikvadze, Tinatin Kutubidze

###### Tbilisi State Medical University, Tbilisi, Georgia

####### **Correspondence:** Eka Nakhutsrishvili

*Pediatric Rheumatology 2024*, **22(2):**PReS24-ABS-1356

**Introduction:** An immunocompromised state describes individuals with an impairment of the immune system which can arise from primary or secondary immunodeficiencies. OM is the infection of bone by haematogenous, direct or contiguous invasion. Osteomyelitis in immunocompromised children can present differently from immunocompetent children and can cause devastating sequelae if treated inadequately.

**Objectives:** We aim to review the clinical profile, treatment and outcome of immunocompromised child with osteomyelitis.

**Methods:** Review of immunocompromised neonate admitted with osteomyelitis in our hospital

**Results:** Patient P2G2, gestation 39 weeks, birth weight - 3000 g, length - 49 cm, Apgar score - 7/8. Immediately after birth, the newborn had fractures of both clavicles. Later, icterus of the skin and visible mucous membranes was detected - phototherapy, infusion therapy were performed under the supervision of neonatologist and traumatologist. The newborn started to be irritated, later, swelling of the right knee, fever, severe pancytopenia was observed, due to marked thrombopenia, LP could not be performed. X-rays of the knee joint revealed increased periarticular soft tissues, periostitis on the lateral surface of the lower third of the right femur, distended bone tissue in the distal epiphysis area, disturbed integrity of the epiphysis contours; fluid was observed in the shoulder joints, left elbow joint and right hip joint. Despite broad-spectrum combined antibiotic therapy, the general condition of the patient worsened in clinical and paraclinical aspects, the need for oxygen increased, and the patient was transferred to mechanical ventilation with SIMV mode, inotropic support was started to correct hypotension and hypoperfusion. Examination of bone marrow cells was performed - no abnormal cells were observed. The patient was consulted by a pediatric phthisiatrist. According to the ultrasound examination of the joints, fluid was still detected in the right knee joint with suppuration, a surgical intervention was performed - phlegmon excision, bacteriological examination of the obtained material revealed Staphylococcus aureus10^8^. The general condition of the patient remained severe, subcutaneous edema was expressed all over the body, an increase in ascitic fluid was noted in dynamics, peritoneal drainage was performed, transudate was obtained - leukocytes were mainly represented by lymphocytes on the background of erythrocytes. It was planned to conduct an immunological study, and severe immunodeficiency was detected - the treatment scheme included intravenous immunoglobulin (IVIG).

**Conclusion:** Although uncommon, osteomyelitis in immunocompromised children and neonates can occur with devastating effects. Treatment involves prolonged administration of antibiotics and surgery. Immune recovery seems to play an important role in bone healing and recovery, and treatment for immunodeficiency should be attempted where possible.

**Date of birth:** août 15, Y

**Patient Consent**

Yes, I received consent

**Disclosure of Interest**

None Declared

### Immunoregulation and basic science

#### P174 Childhood-onset sjögren’s disease has an activated peripheral blood CD4+ memory and T-follicular helper cell phenotype which could drive B-cell dysregulation

##### Junjie Peng^1,2^, Lucia Martin-Gutierrez^1^, Elizabeth C. Jury^1^, George Robinson^2^, Coziana Ciurtin^2^

###### ^1^Centre for Rheumatology Research, Division of Medicine; ^2^Centre for Adolescent Rheumatology Versus Arthritis, University College London, London, United Kingdom

####### **Correspondence:** Junjie Peng

*Pediatric Rheumatology 2024*, **22(2):**PReS24-ABS-1241

**Introduction:** Childhood-onset Sjögren Disease (cSjD) is a rare and poorly understood autoimmune rheumatic disease with onset before 18 years of age. Differences in clinical presentation compared to the adult phenotype, combined with a lack of evidence-based management strategies, warrants research into the pathogenesis of the disease.

**Objectives:** To investigate the in-depth peripheral blood immunological landscape of children and adolescents with cSjD using 30-marker spectral flow cytometry.

**Methods:** A pilot cross-sectional analysis was performed investigating the peripheral blood immune phenotype of children and adolescents with cSjD (N=10; mean age=18 years; age range=16-21 years; all females; mean disease duration=4 years; disease duration range=2-6 years; off steroids and biologic treatment naïve) compared to age and sex-matched healthy controls (cHCs; N=10; mean age=18 years; age range=15-25 years; all females). The identified phenotype was further explored using 30-feature spectral flow cytometry in a larger cohort of cSjD (mean age=21 years; age range=17-30 years; all females; mean disease duration=7 years; disease duration range=2-18 years) and cHCs (mean age=19 years; age range=15-27 years; all females) (N=19/group) using unsupervised dimensionality reduction (UMAP) and clustering analysis (FlowSOM in “Spectre” R package^1^). Serum cytokines assessed by Cytometric Bead Array and Luminex.

**Results:** CD4+ T-cell populations were significantly dysregulated in young people with cSjD compared with matched cHCs, characterised by elevated effector memory (EM, p=0.01) and terminally differentiated EMRA populations (p=0.0002) and reduced central memory T-cells (p=<0.0001). An unsupervised high-parameter analysis of T-cell phenotype using spectral flow cytometry identified twenty CD4+ T-cell clusters, of which four clusters were significantly elevated in young people with cSjD compared with cHCs (p<0.004) all characterised as CD45RA- memory T-cells. Two of these clusters expressed high levels of PD-1, CXCR3 and ICOS, suggesting an activated T-follicular helper-1 phenotype; one cluster expressed elevated PD-1 and ICOS and one cluster was characterised by elevated PD-1 only. This pro-inflammatory and activated T-cell phenotype was supported by increased production of IL-6 by CD4+ T-cells (p=0.04) and increased serum IL-6 levels (p=0.02) in young people with cSjD vs cHCs. Finally, cytokines associated with B-cell activation, IL-10 (p=0.01) and APRIL (p=0.0007), and immune cell trafficking, CCL8 (p=0.03), were also increased in cSjD supporting an ongoing systemic inflammatory environment.

**Conclusion:** While adult-onset SjD is characterised by peripheral CD4+ T-cell lymphopenia^2^, we detected an opposite trend towards significant expansion of CD4+ T-cell subset frequencies in cSjD. This could have significant therapeutic implications, suggesting that cSjD may recapitulate the early phase of the corresponding adult disease phenotype, when treatments with T-cell targeted (such as abatacept, low dose IL2) or broader (such as leflunomide or cyclosporine A) effects could be beneficial.

**Patient Consent**

Yes, I received consent

**Disclosure of Interest**

None Declared

**References**

1. Ashhurst TM, Marsh‐Wakefield F, Putri GH, Spiteri AG, Shinko D, Read MN, Smith AL, King NJ. Integration, exploration, and analysis of high‐dimensional single‐cell cytometry data using Spectre. Cytometry Part A. 2022 Mar;101(3):237-53.
2. Mandl T, Bredberg A, Jacobsson LT, Manthorpe R, Henriksson G. CD4+ T-lymphocytopenia--a frequent finding in anti-SSA antibody seropositive patients with primary Sjögren’s syndrome. The Journal of rheumatology. 2004 Apr 1;31(4):726-8.

### Immunoregulation and basic science

#### P175 Rheumatic masks of cancer. One center experience

##### Sania Valieva, Seda Kurbanova, Dmitry Rossinsky

###### Morozov City Children’s Clinical Hospital , Moscow, Russian Federation

####### **Correspondence:** Sania Valieva

*Pediatric Rheumatology 2024*, **22(2):**PReS24-ABS-1301

**Introduction:** Multiple types of cancer can manifest with musculoskeletal symptoms. When these symptoms are predominant early in the disease course, it’s essential to consider a differential diagnosis that includes JIA, SLE, and septic or reactive arthritis, among other rheumatic diseases.

**Objectives:** The goal was to determine the prevalence and primary clinical features of cancer diagnosed in children initially presumed to have rheumatological conditions, drawing on the clinical data from the Morozov City Children’s Clinical Hospital in Moscow.

**Methods:** A retrospective examination of medical records was conducted on patients identified from a pool of 1452 new cases seen at the tertiary Morozov City Children’s Clinical Hospital in Moscow, spanning from 2020 to 2023, where the final diagnosis turned out to be cancer.

**Results:** Among 1452 children (861 girls and 591 boys) hospitalized during the above period, 24 patients were diagnosed with cancer (1.7% of studying patients). The average age of onset was 6 years old. The average time from the moment of onset to the establishment of the final diagnosis of oncopathology was 4,2 months. The most common diagnoses upon admission to the hospital in patients with cancer pathology detected later were JIA (n=8) and reactive arthritis (n=8). In isolated cases, diagnoses such as sJIA, including with secondary hemophagocytic syndrome, chronic osteomyelitis, septic arthritis, autoinflammatory syndrome were suggested. All children with cancer determined as a result of the examination complained of joints pain, while active arthritis was detected in 20 of them (83%). Ossalgia occurred in half of the patients. 17 patients (70.9%) had fever, 7 (29.2%) had weight loss. Blood test results were assessed and 23 patients (96%) had at least one abnormality. The most frequently observed significant increases in the levels of CRP (n=16; 66.7%), LDH (n=15; 62.5%). Cytopenia was observed in only 7 patients (29.2%). To make a diagnosis, methods such as СT of internal organs, MRI of the brain, examination of red bone marrow punctate and other were used. Before diagnosis, patients received the following therapy: antibiotics (n=21; 87.5%), NSAIDs (n=18; 75%), glucocorticosteroids (n=4; 16.7%), 1 child took immunosuppressants.

In the structure of diagnosed malignant diseases, the largest share was acute lymphoblastic leukemia (n=16 ;66.7%), the remaining diseases were chronic myeloblastic leukemia (n=2; 8.3%), T-cell lymphoma (n=2; 8.3%), neuroblastoma (n=2; 8.3%), one was diagnosed with Sarcoma Ewing, another 1 had a neoplasm of the pineal region.

**Conclusion:** Damage to the musculoskeletal system in combination with constitutional symptoms may indicate the course of both rheumatic and oncological diseases. The results of standard laboratory tests are not always informative. If a neoplastic process is suspected, diagnosis is necessary, including examination of a bone marrow biopsy, computed tomography, magnetic resonance imaging, and dynamic monitoring of the patient. Therapy with glucocorticosteroids complicates this process, erasing clinical and laboratory manifestations, and therefore, until a final diagnosis is established, it is strongly recommended to neglect glucocorticosteroids.

**Patient Consent**

Yes, I received consent

**Disclosure of Interest**

None Declared

### Immunoregulation and basic science

#### P176 Lectin histochemistry of the normal articular cartilage of the hip joint during the postnatal period

##### Andrii Fedotchenko

###### Zaporizhzhia State Medical and Pharmaceutical University, Zaporizhzhia, Ukraine

*Pediatric Rheumatology 2024*, **22(2):**PReS24-ABS-1053

**Introduction:** The distribution of lectins in the intact articular cartilage is still not fully understood.

**Objectives:** The hip joint of intact laboratory rats (from birth to the 90th day) was studied.

**Methods:** The samples were stained with lectins conjugated with horseradish peroxidase (HRP): peanut (PNA), soybean (SBA), Helix pomatia (HPA), wheat germ (WGA), Perca fluviatilis (PFA), Sambucus nigra (SNA), Lens culinaris (LCA), and Vicia sativa (VSA).

**Results:** In newborns, the matrix of the tangential zone revealed moderate (++) reaction with the LCA, VSA, and PFA, but no staining with the PNA, SBA, HPA, WGA, and SNA. Subsequently, it showed no affinity to the HPA, WGA, and SNA. On the seventh day, it stained weakly (+) with the SBA, LCA, VSA, and PFA, but showed no reaction with the PNA. After that, the affinity to the PFA did not vary significantly. On the 14th day, almost no reaction with the LCA and VSA, weak staining with the SBA, and a strong affinity to the PNA could be seen. A strong expression of the PNA, SBA, LCA, and VSA on the 30th-45th day and a weak reaction from the 60th day could be observed.

In newborns, the matrix of the middle zone revealed moderate affinity to the LCA, VSA, WGA, and PFA; but weak reaction with the PNA, SBA, and HPA. After that, the galactose- and mannose-specific lectins as well as the WGA and PFA showed mainly weak affinity. No staining with the SNA was observed. The single foci of irregular polygonal shape with strong lectin affinity could be traced among the weakly stained matrix of the middle zone (SNA, WGA, PNA, SBA, and HPA from the 30th day; PFA, LCA, and VSA from the 60th day). From the 90th day, similar phenomena could be observed also in the tangential zone.

The matrix of the basal zone showed till the 14th day mostly a weak reaction with the PNA and SNA; and a strong reaction with other lectins. From the 30th day, the proliferative and hypertrophic zones of the growth plate, and calcified cartilage spicule revealed strong expression to all studied lectins. The resting zone and primary spongiosa expressed no affinity. The tidemark as the continuous demarcation line was not observed.

The marginal cartilage revealed no affinity to the HPA, WGA, and SNA. Till the seventh day, it showed mainly weak reaction with the PFA, LCA, and VSA, but no staining with the PNA and SBA. On the 14th day, it expressed strong affinity to the PNA and SBA, but almost no staining with LCA, VSA, and PFA. After that, no reaction with the PFA could be traced. From the 30th day, the marginal cartilage showed a robust expression of the PNA, SBA, LCA, and VSA.

The nuclei of chondrocytes expressed a different affinity (from moderate to strong). The capsule of the lacunae of chondrocytes revealed a strong reaction. No significant differences between caput femoris and acetabulum were observed.

**Conclusion:** The glycophenotype of the normal articular cartilage undergoes significant changes throughout the postnatal period, reflecting its morphogenesis. The obtained data are proposed as a pattern to compare norm with pathology.

**Patient Consent**

Not applicable (there are no patient data)

**Disclosure of Interest**

None Declared

